# Benzenoid Aromatics from Renewable Resources

**DOI:** 10.1021/acs.chemrev.4c00087

**Published:** 2024-09-17

**Authors:** Shasha Zheng, Zhenlei Zhang, Songbo He, Huaizhou Yang, Hanan Atia, Ali M. Abdel-Mageed, Sebastian Wohlrab, Eszter Baráth, Sergey Tin, Hero J. Heeres, Peter J. Deuss, Johannes G. de Vries

**Affiliations:** † Leibniz Institut für Katalyse e.V., Albert-Einstein-Strasse 29a, 18059 Rostock, Germany; ‡ State Key Laboratory of Heavy Oil Processing, College of Chemical Engineering and Environment, China University of Petroleum (Beijing), 102249 Beijing, China; # Joint International Research Laboratory of Circular Carbon, Nanjing Tech University, Nanjing 211816, PR China; ⊥ Green Chemical Reaction Engineering, Engineering and Technology Institute Groningen, University of Groningen, Nijenborgh 4, 9747 AG Groningen, The Netherlands

## Abstract

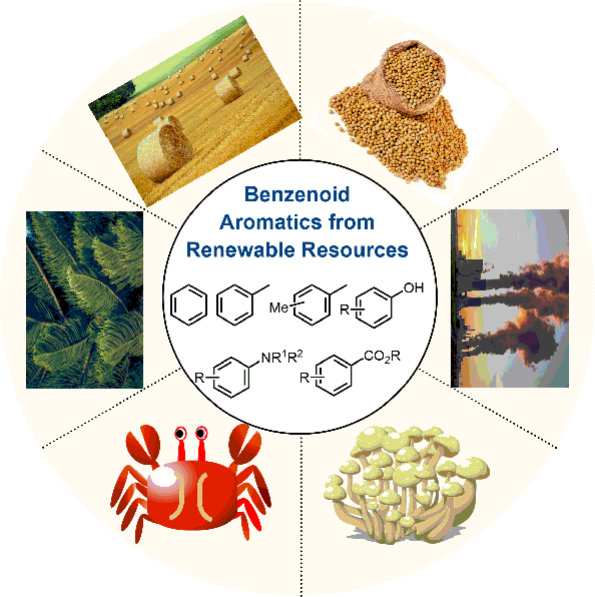

In this Review, all known chemical methods for the conversion of renewable resources into benzenoid aromatics are summarized. The raw materials that were taken into consideration are CO_2_; lignocellulose and its constituents cellulose, hemicellulose, and lignin; carbohydrates, mostly glucose, fructose, and xylose; chitin; fats and oils; terpenes; and materials that are easily obtained via fermentation, such as biogas, bioethanol, acetone, and many more. There are roughly two directions. One much used method is catalytic fast pyrolysis carried out at high temperatures (between 300 and 700 °C depending on the raw material), which leads to the formation of biochar; gases, such as CO, CO_2_, H_2_, and CH_4_; and an oil which is a mixture of hydrocarbons, mostly aromatics. The carbon selectivities of this method can be reasonably high when defined small molecules such as methanol or hexane are used but are rather low when highly oxygenated compounds such as lignocellulose are used. The other direction is largely based on the multistep conversion of platform chemicals obtained from lignocellulose, cellulose, or sugars and a limited number of fats and terpenes. Much research has focused on furan compounds such as furfural, 5-hydroxymethylfurfural, and 5-chloromethylfurfural. The conversion of lignocellulose to xylene via 5-chloromethylfurfural and dimethylfuran has led to the construction of two large-scale plants, one of which has been operational since 2023.

## 1 Introduction

The build-up of largely man-made greenhouse gases in the atmosphere has led to global warming due to the greenhouse effect. Practically all nations have ratified the Paris agreements, which seek to limit global warming to 1.5 °C by the year 2050. Unfortunately, the agreements do not stipulate how that should be achieved, but it is clear to everyone that the use of fossil fuels will have to be phased out. This also means that the chemicals we depend on in our daily lives will have to be produced from renewable resources. The number of useful renewable resources is rather limited, but the supply is rather abundant and more than enough to cover the current and future need for chemicals. Lignocellullose is by far the largest supply of renewable carbon; it is available in the form of wood, leaves, and grasses, as waste from the agro and paper industries, as well as in the form of municipal waste. Other resources may come in lower supplies but have proven their usefulness. Chitin, mostly available from shells of crustaceans, is a vastly underutilized renewable resource. Starches are plentiful, available from corn and cereals; they are easily hydrolyzed to monomeric carbohydrates. The use of starches for fuel could potentially threaten the food supply, but their use for chemicals production would lay a much lower claim and will not threaten food production. Other much smaller resources are oils and fats and terpenes, such as pinenes and limonene. CO_2_ can also be considered as a source of renewable carbon. It can be reduced to CO, formic acid, methanol, or methane using either electrons from renewable (solar or wind) energy or hydrogen made via electrolysis from water with renewable electricity. Finally, there is biogas, from the anaerobic fermentation of biomass.

In the past decades, much progress has been made in the conversion of renewable resources to aliphatic chemicals, but conversion to benzenoid aromatics has lagged somewhat behind, although this is slowly turning around, and the first factory for large-scale production of xylene based on renewables started production as this manuscript was being written. Thus, this is a good moment to take stock and critically review all methods that have been published so far to see promising directions and to find out what is still missing.

**Scheme 1:**
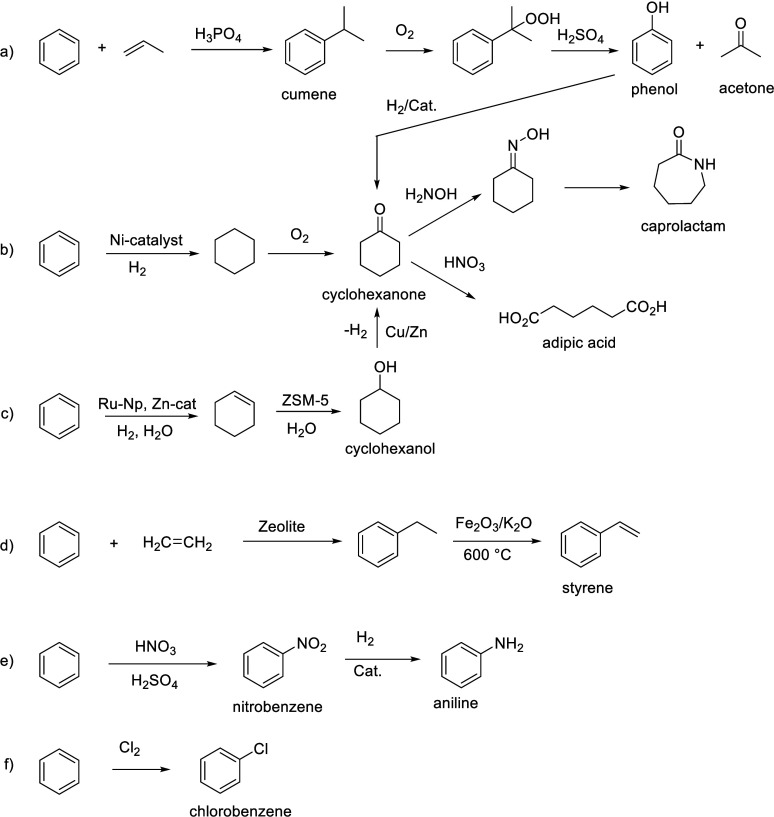
Bulk Chemicals Produced from Benzene

**Scheme 2:**
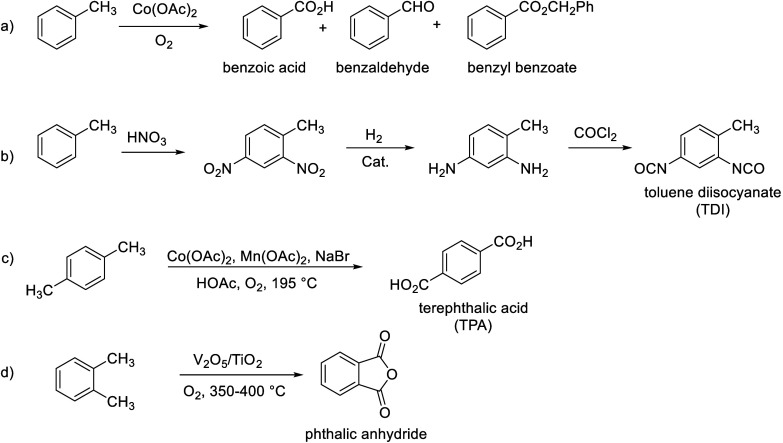
Bulk Chemicals Produced from Toluene and Xylenes

Benzenoid aromatics are widely used as solvents and as building blocks for the production of polymers, pharmaceuticals, agrochemicals, flavors, and fragrances and many other fine chemical applications. Benzene, toluene, and xylenes are produced by naphtha cracking, and from these basic building blocks a host of other important bulk chemicals, such as phenol, aniline, chlorobenzene, terephthalic acid, and others, are made (Schemes 1 and 2).

In this Review, we will comprehensively summarize all chemical methods that have been developed for the conversion of renewable resources to benzenoid aromatics.

A limited number of aromatic compounds have also been produced via fermentation, mostly from glucose. These fall outside the scope of this Review, although for completeness’ sake we have included a list of aromatic compounds that can be produced by fermentation (Table 1). Of these, only the amino acids l-phenylalanine, l-tryptophan, and l-tyrosine (Table 1, entries 1–3) are produced on large scale.^1^ It is generally possible to obtain reasonably high titers and productivities with aromatic compounds that are natural metabolites. These fermentations all proceed via the Shikimate pathway. Non-natural aromatic compounds could, in principle, also be produced via fermentation, but these compounds—styrene is a good example (Table 1, entry 22)—are often toxic, and usually titers remain too low for economical production.

We found only a single overall review on aromatics from renewables,^5^ although many reviews exists on the separate topics. The chemistry we review here is highly diverse. The low-temperature methods usually proceed in good yields, and the products are isolated in pure form. The high-temperature methods, on the other hand, usually result in char, gases, and an oil which is a mixture of chemicals that are usually only analyzed by GC. For these publications we have focused on studies that reach a threshold of >10% isolated yield or >20% yield for mixtures of aromatics.

## 2 Aromatics from Sugars

### 2.1 Introduction

Carbohydrates constitute between 65 and 75% of lignocellulosic biomass. They are produced from carbon dioxide and water by plants and by some microorganisms via photosynthesis.^6^ Monosaccharides, the simplest carbohydrates, include glucose, fructose, and xylose. In this section, we will summarize all reports on the chemical conversion of monosaccharides to benzenoid aromatics.

A number of methods are used industrially to obtain sugars.^7^ Currently the major source is from the hydrolysis of starch, an amorphous polymer of glucose, which is relatively easy. This is mostly done enzymatically using the α-amylase enzyme, although it is also possible using dilute acid. This is currently the major source of glucose. Glucose is converted enzymatically to fructose, and the mixture is separated by simulated moving bed (SMB) on a very large scale. Lignocellulose can be pretreated to remove the protective lignin layer, which allows the precipitation and isolation of its main component, cellulose, which is a highly stable polymer of glucose. Cellulose can be hydrolyzed enzymatically using a mixture of enzymes, which is a relatively slow and expensive procedure. This method is used if the sugars will be used for fermentation, as in the production of cellulosic bioethanol, since it does not result in unwanted side products that could inhibit the fermentation. However, if the sugars are to be used for chemical conversion, it is also possible to hydrolyze lignocellulose directly to sugars using concentrated HCl, a method known as the Bergius process.^8,9^

### 2.2 Aromatics from Catalytic Fast Pyrolysis of Sugars

Pyrolysis is a method to convert sugars into chemicals by heating to 400–600 °C under an inert atmosphere. The products are a range of small molecules in the form of gas or liquid; in addition, char is formed. Fast pyrolysis usually delivers a reasonable yield of liquid products, which is known as bio-oil, containing more than 400 organic species, including aromatic and aliphatic hydrocarbons. If the pyrolysis is carried out in the presence of a catalyst, usually a zeolite, the product spectrum dramatically changes, and the reaction can be tuned to either deliver mostly aromatics or mostly alkenes.

A number of reviews exist on this topic focusing on catalysts,^10^ techniques,^11^ chemistry,^12^ or processes.^12^ In this section, we will describe the aromatic compounds that can be obtained via catalytic pyrolysis of sugars and derivatives including yields and/or selectivity.

The literature on catalytic fast pyrolysis (CFP) of sugars is summarized in Table 2. The formation of aromatics from sugars through catalytic pyrolysis was traced back to the 1980s, when Chen and co-workers subjected a glucose solution in methanol to a fixed-bed reactor containing a zeolite catalyst at 510 °C.^13^ Aromatics were produced in a yield of around 9%. Around the same time, Dao and Haniff observed trace amounts of aromatics (<1%) from the pyrolysis of glucose and fructose in aqueous solution catalyzed by 80 wt% ZSM-5 in bentonite at 450 °C.^14−16^ When methanol was used as co-feed for the pyrolysis of glucose, the aromatics yield was increased to 2.7–10.0 wt%, which was further elevated to 14.6 wt% by replacing bentonite with SiO_2_–Al_2_O_3_.^16^ An uncharacterized glucose-isopropylidene derivative, obtained by reacting glucose with excess acetone, was co-fed with methanol in the CFP catalyzed by H-ZSM-5, resulting in 6.9–26.0 wt% yield of aromatics.^16^ Modification of ZSM-5 with either Mn or Zn significantly reduced the aromatics yields in the pyrolysis of glucose to <1 wt%, whereas the yields of aromatics from fructose were increased to 20.4 wt%.^16^ The aromatic distribution was not described.^16^

In 2008, Huber and co-workers studied the CFP of xylitol, glucose, and cellobiose at 600 °C and produced unstable bio-oils containing various aromatic chemicals.^17,18^ Using ZSM-5 as catalyst, aromatics yields of 22.9–47.5% were observed.^17,18^ Under these conditions, all feedstocks produced similar aromatic distributions with high selectivities (40–45%) to naphthalene. The key factors to improve aromatic yields include fast heating rates, high catalyst to feed ratios, and the proper choice of catalyst.^17^

**Scheme 3:**
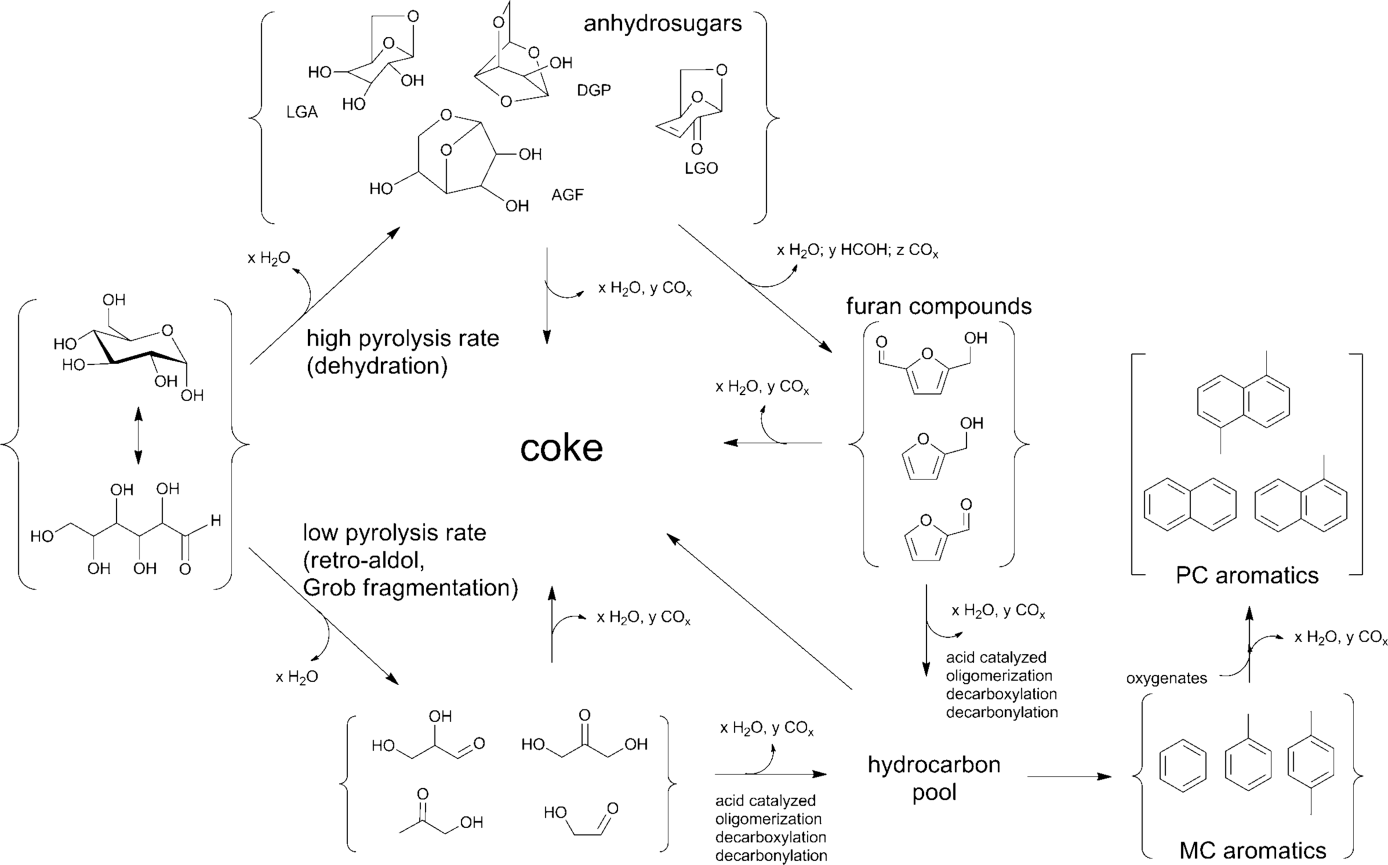
Proposed Reaction Mechanism for the CFP of Glucose over ZSM-5 Reproduced with permission from ref (19). Copyright 2010 Elsevier.

A mechanistic study on the catalytic fast pyrolysis of glucose over ZSM-5 based on isotopic studies and various other technologies suggested two major pathways, as shown in Scheme 3.^19,20^ Two pathways are involved in the formation of aromatics during glucose pyrolysis. The first pathway is the rapid thermal decomposition of glucose to small oxygenates at low temperature through retro-aldol and Grob fragmentation reactions. The second pathway proceeds via anhydrosugars and furans at high temperature via dehydration reactions. The use of ZSM-5 as catalyst significantly decreased the temperature required for both decomposition pathways. These dehydrated sugars and decomposition products are transformed to aromatics inside the pores of the catalysts, the rate of which was significantly slower. Coke formation on the catalyst surface via furan polymers is the major competitive reaction to aromatic production. The selectivity to aromatics was dependent on the temperature and the catalyst/feed ratios.^19^

**Table 1:** Aromatic Compounds That Can Be Produced by Fermentation (from Glucose, unless Mentioned Otherwise)

Entry	Name	Titer (g/L)	Ref
1	l-Phenylalanine	46	(1)
2	l-Tryptophan	30–50	(1)
3	l-Tyrosine	55	(1)
4	Vanillin^a^	4.3	(2)
5	Salicylic acid	11.5	(2)
6	*p*-Hydroxybenzoic acid	36.6	(2)
7	2-Phenylethanol	1.0	(2)
8	*p-*Coumaric acid	12.5	(3)
9	Caffeic acid	2.8	(4)
10	Anthranilic acid	14	(4)
11	*p*-Aminobenzoic acid	43	(4)
12	Gallic acid	20	(4)
13	Quinic acid	49	(4)
14	Pyrogallol	1	(4)
15	Cinnamic acid	6.9	(4)
16	Salvianic acid A	7.1	(4)
17	Salidroside (a glucoside of Tyrosol)	6.9	(4)
18	4-Vinyl-phenol	17.6	(4)
19	Indigo	18	(4)
20	Violacein	5.4	(4)
21	Deoxy-violacein	1.6	(4)
22	Styrene	0.26	(4)

a From ferulic acid.

**Table 2:** Catalytic Fast Pyrolysis of Sugars—Products Analyzed by GC and GC-MS

								Aromatic selectivity (%)			
Entry	Feed	Catalyst (Si/Al ratio)	Feed/cat or WHSV (h^–1^)^a^	Reactor	Reaction time (s)	*T* ( °C)	Aromatic carbon yield (%)	Benzene	Toluene	Xylene	Ref
1	Glucose-isopropylidene derivative^b^	H-ZSM-5 (19.3)	1.11^a^	Flow-microreactor	3 h	450	26.0 wt	–	–	–	(16,26)
2	Fructose^b^	H-ZSM-5 (19.3)	0.22^a^	Flow-microreactor	3 h	450	20.4 wt	–	–	–	(16,26)
3	Fructose^b^	Zn-ZSM-5 (20.9)	0.19^a^	Flow-microreactor	3 h	450	20.4 wt	–	–	–	(16,26)
4	Fructose-isopropylidene derivative^b^	Mn-ZSM-5 (19.5)	0.157^a^	Flow-microreactor	3 h	450	20.8 wt	–	–	–	(16,26)
5	Xylitol	ZSM-5 (15)	0.05	Pyroprobe	240	600	47.5	5.7^d^	9.0^d^	8.5 C^c^^,^^d^	(17,18,27)
6	Glucose	ZSM-5 (15)	0.05	Pyroprobe	240	600	31.4	3.6^d^	5.7^d^	4.1 C^c^^,^^d^	(17,18,27)
7	Cellobiose	ZSM-5 (15)	0.05	Pyroprobe	240	600	28.2	3.8^d^	6.3^d^	4.2 C^c^,^d^	(17,18,27)
8	Glucose	ZSM-5 (15)	0.05	Pyroprobe	240	600	23.6	–	–	–	(17,18,27)
9	Glucose	ZSM-5 (15)	0.05	Pyroprobe	240	600	29.4	–	–	–	(17,18,27)
10	Glucose	ZSM-5 (15)	0.11	Pyroprobe	240	600	27.2	–	–	–	(17,18,27)
11	Glucose	ZSM-5 (15)	0.25	Pyroprobe	240	600	22.9	–	–	–	(17,18,27)
12	Glucose	ZSM-5 (15)	0.05	Pyroprobe	240	600	35.5	14.2	27.1	17.3^c^	(21)
13	Glucose	ZSM-11 (15)	0.05	Pyroprobe	240	600	25.3	12.8	18.5	12.9^c^	(21)
14	Glucose	ZSM-5 (11.5)	0.05	Semibatch Pyroprobe	240	600	29^h^	17.5^h^	24.0^h^	16.5^h^	(22)
15	Glucose	ZSM-5 (15)	0.05	Semibatch Pyroprobe	240	600	43^h^	13.0^h^	20.5^h^	15.0^h^	(22)
16	Glucose	ZSM-5 (25)	0.05	Semibatch Pyroprobe	240	600	33^h^	11.5^h^	20.5^h^	15.5^h^	(22)
17	Glucose	ZSM-5 (40)	0.05	Semibatch Pyroprobe	240	600	28^h^	10.0^h^	21.3^h^	18.5^h^	(22)
18	Glucose	MicZSM-5 (15.2)^e^	0.05	Semibatch Pyroprobe	240	600	31^h^	15.0^h^	28.0^h^	19.0^h^	(22)
19	Glucose	MicZSM-5 (15.2)^e^^,^^f^	0.05	Semibatch Pyroprobe	240	600	31^h^	14.0^h^	27.0^h^	19.5^h^	(22)
20	Glucose	MesZSM-5 (14.4)^g^	0.05	Semibatch Pyroprobe	240	600	32^h^	9.5^h^	21.5^h^	19.0^h^	(22)
21	Glucose	MesZSM-5 (14.4)^f^^,^^g^	0.05	Semibatch Pyroprobe	240	600	30^h^	9.5^h^	20.5^h^	18.5^h^	(22)
22	Glucose	H-ZSM-5 (15)	0.05	Tandem microreactor	–b	500–600	27.0	24.5	36.5	12.5	(23)
23	Sorbitol	Ni-H-ZSM-5/SBA-15 (38)	0.75^a^	Fixed-bed reactor	–b	320	28.2 wt	–	1.9^d^	8.2^d^	(24)
24	Cellobiose	Fe-H-ZSM-5 (11.5)	0.10	Micropyrolyzer	30	500	25.8	12.1	39.4	14.1	(25)

a WHSV = weight hourly space velocity.

b Co-feeding with methanol.

c Ethylbenzene included.

d Carbon yield.

e Microporous ZSM-5.

f Treated with tartaric acid.

g Mesoporous ZSM-5.

h No precise data available, estimated from figures.

Huber’s group investigated the effects of pore size and shape on the production of aromatics from glucose by testing a range of zeolites with different pore sizes.^21^ Use of zeolites with medium pore sizes in the range of 5.2–5.9 Å as catalysts produced the highest aromatic yields, while small-pore zeolites produced CO, CO_2_, and coke, and large-pore zeolites enhanced coke formation. The internal pore space and steric hindrance had decisive effects on aromatics production from glucose. The highest aromatic yields (35.5%) were realized by ZSM-5 and ZSM-11, containing medium size pores with moderate internal pore size and steric hindrance.^21^ The effects of Si/Al ratios of ZSM-5 on the aromatic production from glucose pyrolysis was also studied by Huber and Lobo.^22^ An optimum ratio was found at 30, indicating the critical role of the concentration of the acidic sides inside the zeolites. The hierarchical mesopores within the zeolites and the external surface acid sites were investigated but these had a very limited effect on the conversion of glucose to aromatics.^22^

**Figure 1:**
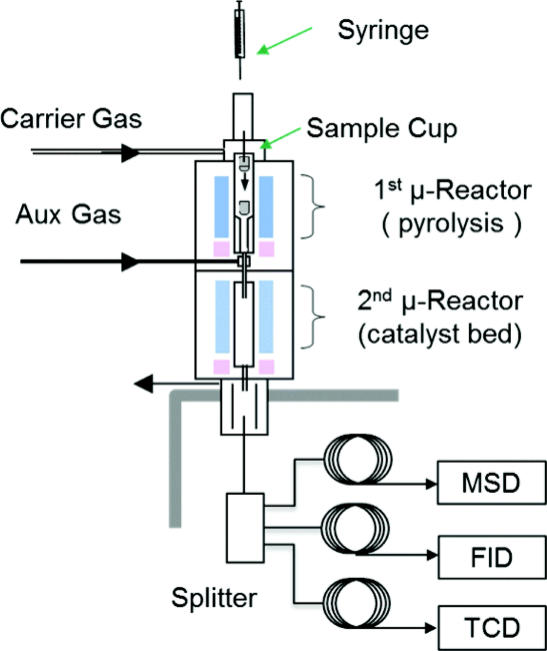
Diagram of tandem microreactor system for furfural pyrolysis. Reproduced with permission from ref (23). Copyright 2015 The Royal Society of Chemistry.

Brown’s group introduced the use of a tandem microreactor for the CFP of glucose, wherein glucose first passed through a pyrolysis reactor before entering into a fixed-bed reactor with H-ZSM-5 as catalyst (Figure 1).^23^ The obtained aromatics yield (27%) and product distribution were consistent with the weight sum of products that were produced from the individual oxygenates, suggesting no significant interactions between the oxygenated species released from the pyrolysis of glucose.^23^

Wang and co-workers prepared a Ni-H-ZSM-5/SBA-15 catalyst, containing both microporous (H-ZSM-5) and mesoporous (SBA-15) structures, for the CFP of sorbitol in a fixed-bed reactor and produced aromatic compounds in 28.2 wt% yield at 320 °C under 40 bar H_2_.^24^ The large sorbitol molecule was firstly hydrodeoxygenated to small oxygen-containing intermediates catalyzed by Ni-SBA-15. The small oxygen-containing intermediates entered into the H-ZSM-5 micropores and converted to aromatic molecules.^24^

Mullen reported the use of Fe-modified H-ZSM-5 as catalyst for the pyrolysis of cellobiose and observed a 25.8% aromatic carbon yield with 1.4 wt% Fe loading, compared to 17.3% carbon yield with parent H-ZSM-5 as catalyst.^25^ Use of zeolites with an increased Fe loading led to a slightly decreased aromatic carbon yields (15.5–15.6%). With a catalyst loading of 10 times the weight of cellobiose, Fe-H-ZSM-5 with 1.4 wt% Fe favored the formation of benzene (12.1%) and naphthalene (28.9%) rather than *p*-xylene (14.1%) and other alkyl benzenes, while the use of H-ZSM-5 as catalyst produced benzene, naphthalene, and *p*-xylene in 10.9%, 22.4%, and 17.3% selectivity, respectively.^25^

### 2.3 Aromatics from Fermentation Products from Sugars

A wide range of products can be obtained from sugars, mostly glucose, via fermentation. Some of these processes are performed on very large scale, such as bioethanol, and these products may form interesting raw materials for the production of benzenoid aromatics.

#### 2.3.1 Aromatics from Bioethanol

Although ethanol was produced in the past via hydration of ethylene, today most ethanol is produced via fermentation of sugars, stemming from corn starch or sugar cane, using yeasts.^28^ The annual global bioethanol production reached 27.2 million gallons per year until 2021.^29,30^ The main driver for this is its use as fuel additive, which is mandatory in many countries. The United States and Brazil are the major producers of bioethanol in the world. Since it was feared that the extensive use of corn for bioethanol production would jeopardize its availability for food (food *vs* fuel), other sources of sugars have been investigated, with a particular focus on inedible lignocellulose.^31^ Several companies have started production of lignocellulosic bioethanol in the past 10 years, but in the meantime almost all have ceased production. Catalytic conversion of ethanol to hydrocarbons (ETH), mainly aromatics and paraffins, has been reviewed with emphasis on the catalysts and hydrocarbon yields.^32−35^ In this section, we will focus on the formation of aromatics from ethanol.

**Figure 2:**
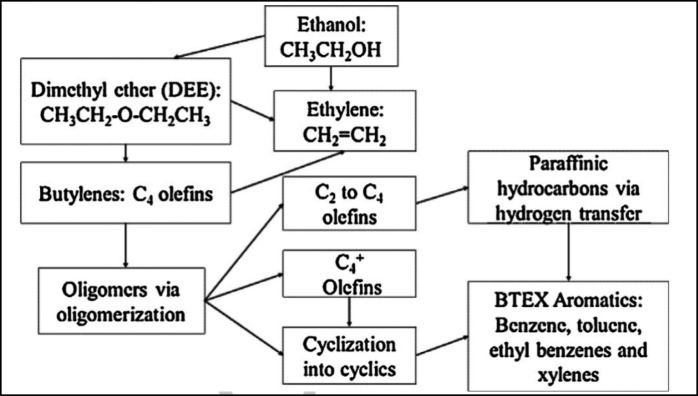
Proposed pathway of ethanol conversion to hydrocarbons. Reproduced with permission from ref (33). Copyright 2015 Elsevier.

The proposed main pathway of ethanol conversion to aromatic and aliphatic hydrocarbons is shown in Figure 2. Below 300 °C, ethanol is dehydrated to ethylene or diethyl ether, which are converted over Brønsted acids into C_4_ olefins and higher oligomers.^33^ Cracking of these oligomers produces C_2_, C_3_, and C_4+_ olefins. Cyclization reactions of these olefins form BTEX aromatics, whereas hydrogen transfer processes yield paraffinic hydrocarbons.^36^ Aromatics can also be produced by bimolecular hydrogen transfer between cyclic hydrocarbons and light olefins.^33,36^

Most studies on the conversion of ethanol to BTX have been performed in a fixed bed reactor. H-ZSM-5-type zeolites, featuring both acidity and porosity, are the most commonly used catalysts for the conversion of ethanol to hydrocarbons. The earliest record on pyrolysis of ethanol was tracked to 1983, when Mutharasan and co-workers used ZSM-5 for ethanol conversion in a fixed bed reactor at 300–400 °C under various pressures and produced ≤36 wt% yield of aromatics.^37^ Later, Costa and co-workers comprehensively studied the ZSM-5-catalyzed conversion of ethanol at 300–500 °C and evaluated the effects of Si/Al ratios, water composition, pressure, temperature and WHSV on product distribution.^38^ A maximum 41% yield of aromatics was obtained at 400 °C using ZSM-5 (Si/Al = 85) as catalyst (Table 3, entry 1).^38^

**Table 3:** Catalytic Conversion of Ethanol to BTX over Different Catalysts^a^

						Aromatic distribution (wt%)			
Entry	Catalyst (Si/Al ratio)	WHSV (h^–1^)	TOS (h)	*T* (°C)	Aromatics yield (wt%)	Benzene	Toluene	Xylene	Ref
1	ZSM-5 (85)	0.25	2	400	41	1.5	12.3	14.0	(38)
2	Nano ZSM-5 (30)	2.0–3.0	–	500	50.6	2.5	17.0	27.5	(36)
3	Micro ZSM-5 (30)	2.0–3.0	–	500	36.3	1.1	11.7	22.3	(36)
4	Micro ZSM-5 (100)	2.0–3.0	–	500	31.8	1.8	11.5	14.7	(36)
5	H-ZSM-5 100-500 nm (70-73)	1.58	1	500	23–28^b^	–	–	–	(39)
6	H-ZSM-5 (15)	4.73	6	300–400	48–53^c^	–	–	–	(40)
7	H-ZSM-5 (15, 40, 140)	7.9	6	360	41–56^c^	–	–	–	(40)
8	ZSM-5 (41, 136)	7.9	6	360	40–62^c^	–	–	–	(41)
9	ZSM-5-HTS (48, 120)	7.9	6	360	53–57^c^	–	–	–	(41)
10	H-MFI (25)	1.43	3.5	352	30	–	–	–	(42)

a All reactions were carried out in a fixed bed reactor. Ethanol was fully converted. Product was analyzed by GC.

b Aromatics selectivity.

c Yields were calculated based on weight of ethanol minus the weight of water.

Viswanadham and co-workers used nano-sized ZSM-5 with 30 nm sized crystallites for the catalytic pyrolysis of ethanol at 500 °C and produced a 50.6% yield of aromatics (Table 3, entry 2), significantly higher than that obtained using microcrystalline ZSM-5 catalysts (31.8–36.3 wt%, entries 3 and 4). The authors ascribed the high yield of aromatics from nano ZSM-5 to the enhanced adsorption–desorption properties of the catalyst and consequently the increased diffusion of hydrocarbon species.^36^ H-ZSM-5 with different crystallite sizes were tested for the conversion of ethanol to propylene and aromatics at 500 °C.^39^ The aromatics selectivity changed from 28% to 24% when the crystallite sizes was decreased from 500 to 100 nm. However, these catalysts deactivated rapidly, showing a sharp decrease in aromatic yields from the onset (entry 5). Ramasamy investigated the conversion of ethanol to hydrocarbons over H-ZSM-5 zeolites with varying Si/Al ratios (15, 40, and 140) at 300–400 °C, observing 41–56% yields of aromatics (entries 6 and 7).^40^ The group also prepared hierarchical mesoporous nano-sized ZSM-5 (ZSM-5-HTS) by hydrothermal treatment that showed an increased catalyst lifetime and afforded 53–57% yields of aromatics from ethanol at 360 °C (entries 8 and 9).^41^

H-MFI zeolite was also used as catalyst for the pyrolysis of ethanol at 352 °C and produced a 30% molar yield of aromatic chemicals (Table 3, entry 10).^42^ H-Beta zeolites and metal-modified H-Beta showed high selectivity toward gaseous products and only produced <6.5 wt% yield of hydrocarbons with high selectivity to C_9_–C_10+_ aromatics.^43^

The effect of doping zeolites with metals has also been investigated in the conversion of ethanol (Table 4). Ga-modified ZSM-5 is highly selective to aromatics formations, while P-, Ni-, or Fe-modified H-ZSM-5 usually favor the formation of gaseous olefins and only produce <19% yield of aromatics.^44,45^

**Table 4:** Catalytic Conversion of Bioethanol over Metal-Modified Zeolites^a^

							Aromatic distribution (selectivity) (%)			
Entry	Catalyst (Si/Al ratio)	WHSV (h^–1^)	TOS (h)	*T* (°C)	Ethanol concn (%)	Aromatics sel. (%)	Benzene	Toluene	Xylene	Ref
1	H-ZSM-5 (14.5)	7.3	–	400	92.2	52.9^b^	3.1	18.9	30.9	(46)
2	Metal-H-ZSM-5 (14.5)^c^	7.3	–	400	91.5–98.4	4.5–75.6^b^	0.9–4.3	5.4–24.5	17.0–46.6	(46)
3	H-ZSM-5 (14.5)^d^	60^e^	–	400	100	53.7–53.9	–	–	–	(47)
4	Fe-H-ZSM-5 (14.5)^d^	60^e^	–	400	92.3–100	8.5–41.8	–	–	–	(47)
5	2% Mo_2_C/ZSM-5 (40)	40^e^	–	500–600	100	20.2–23.6	4.3–8.8	7.4–9.2	3.4–7.7	(48)
6	2% Ga_2_O_3_/ZSM-5 (40)	40^e^	–	500–600	100	35.0–43.2	9.3–22.4	14.7–16.1	4.0–9.1	(48)
7	2% ZnO/ZSM-5 (40)	40^e^	–	500	100	21.6	5.8	9.2	4.9	(48)
8	ZSM-5 (40) + 2% Mo_2_C/ZSM-5 (40)	40^e^	–	500	100	24.8	3.8	9.8	8.5	(48)
9	ZSM-5 (40) + 2% Ga_2_O_3_/ZSM-5 (40)	40^e^	–	500–600	100	44.9–46.7	4.4–11.0	17.9–20.5	12.6–18.6	(48)
10	ZSM-5 (40) + 2% ZnO/ZSM-5 (40)	40^e^	–	500	100	36.3	10.2	14.6	9.6	(48)
11	1.7% Ga-ZSM-5 (11.5)	1.6	–	300–500	100	5.5–40.9^f^	–	–	–	(49)
12	H-ZSM-5 (11.5)	0.4–3.2	–	450	100	16.8–26.0^f^	–	–	–	(49)
13	6.2% Ga-ZSM-5 (11.5)	0.4–3.2	–	450	100	29.5–55.3^f^	10–20	18–24	11–14	(49)
14	H/ZSM-5 (21)^g^	0.5–3.5	90	300–400	84–88	17–43	–	–	–	(50)
15	1% Ni/ZSM-5 (21)^g^	0.5–3.5	90	300–400	90–92	17–41	–	–	–	(50)
16	H-ZSM-5 (21)^h^	1.0	60	360^j^	–	23.2^i^	1.1^i^	7.5^i^	11.7^i^	(52)
17	3 wt% Zn-ZSM-5 (21)^h^	1.0	140	360^j^	–	30.6^i^	1.5^i^	11.5^i^	13.8^i^	(52)
18	3 wt% Ga-ZSM-5 (21)^h^	1.0	240	360^j^	–	27.5^i^	1.1^i^	8.9^i^	13.8^i^	(52)
19	CaO-H-ZSM-5 (25)	3.0	1	300–500^k^	100	9–32^i^	–	–	–	(51)
20	MgO-H-ZSM-5 (25)	3.0	1	300–500^k^	100	8–25^i^	–	–	–	(51)
21	Ni^2+^-ZSM-5 (25)	3.0	1	400^k^	100	42.5^i^	–	–	–	(51)
22	Cr^3+^-ZSM-5 (25)	3.0	1	400^k^	100	34.1^i^	–	–	–	(51)
23	0.7% Pd-0.4% Zn/MFI/Al_2_O_3_ (30)^l^	0.6	50	330^m^	100	42^i^	–	–	–	(53)
24	0.1% Au-0.06% Pd/MFI/Al_2_O_3_^l^	0.6	4	330^m^	100	30^i^	0.8	3.7	8.2	(54)

a All reactions were carried on a fixed bed reactor; product was analyzed by GC.

b Carbon selectivity of BTX.

c H-ZSM-5 (14.5) incorporated with various metals.

d With or without water.

e Flow rate (mL/min).

f BTX yield (wt%).

g Products were analyzed by GC and GC-MS.

h Blended on an extra 50 wt% of Al_2_O_3_.

i Yield (wt%).

j Pressure = 10 bar.

k ρ(ethanol) = 0.2 bar.

l Reactions were performed in a fixed bed microreactor; products were analyzed by GLC.

m Pressure = 5 bar.

Inaba and co-workers evaluated different zeolites, including H-Beta, H-ZSM-5, USY, and H-Mordenite, for the conversion of ethanol to aromatics at 400 °C.^46^ Among the tested zeolites, H-ZSM-5 produced BTX at selectivity of 53.9% at 92.4% ethanol conversion (Table 4, entry 1). H-ZSM-5 was then impregnated with different metals, including Cr, Fe, Ni, Ru, Ag, Ir, Pt and Au, and investigated as catalysts for ethanol conversion at 400 °C affording aromatics with 4.5–73.6% BTX selectivity at 91.5–98.4% conversion (entry 2). Copper and magnesium impregnation resulted in high selectivity for ethylene. Doping with non-noble metals usually led to worse results, whereas doping with noble metals resulted in BTX selectivities that were similar to the results with the parent ZSM-5 catalyst. Best results were obtained with gallium impregnation (73.6%). Interestingly, Ni- and Fe-impregnated H-ZSM-5 as catalysts also yielded BTX in 38.8% selectivity at 96% ethanol conversion and 51.2% selectivity at 97% ethanol conversion, respectively, different from Machado and Phung’s observations.^44,45^ Later, the same group prepared Fe-impregnated Fe-H-ZSM-5 with various Fe source for this reaction.^47^ The addition of Fe reduced the selectivity toward aromatics, but still resulted in 8.5–41.8% selectivity toward aromatic chemicals at close to 100% ethanol conversion (entries 3 and 4). The authors presumed that addition of Fe caused a reduction of acidic sites that are required for aromatics formation.^47^

Impregnation of ZSM-5 with Mo_2_C, Ga_2_O_3_, and ZnO showed positive effects on aromatics formation from ethanol and afforded 20.2–46.7% yield of aromatics (Table 4, entries 5–7).^48^ Among all these catalysts, Ga_2_O_3_-ZSM-5 stands out and produced 35.0–43.2% yields of aromatics. This was attributed to the ability of metal promoters to convert ethylene that formed by ethanol dehydration over pure ZSM-5 to aromatic chemicals. The use of a two-bed reactor, where ZSM-5 in the first bed effectively dehydrated ethanol to ethylene and metal-promoted ZSM-5 in the second bed converted ethylene to aromatics, further improved aromatic yields to 24.8–46.7% (entries 8–10).^48^ Narula and co-workers prepared ZSM-5 catalysts with different Ga loadings (0.5–6.2 wt%) and examined their activity on ethanol conversion to BTXs under different reaction conditions.^49^ Under all conditions, Ga-modified ZSM-5 produced higher yields of BTX than the parent H-ZSM-5 and reached to a maximal 55.3% yield (entries 11–13). The authors attributed this result to the promoted oligomerization and dehydrocyclization of propylene and butene to aromatics.^48^

Wu and co-workers studied the effect of 1% Ni on H-ZSM-5 in the conversion of both ethanol and real bio-ethanol stemming from fermentation.^50^ Ni/H-ZSM-5 has altered acid properties compared to the parent H-ZSM-5, enhancing the catalytic conversion of ethanol from 84–88% to 90–92% under the same reaction conditions, while the selectivity toward aromatics remained the same (Table 4, entries 14 and 15). For the conversion of a real 72% bioethanol aqueous solution at 350 °C, Ni/ZSM-5 exhibited a good stability over 168 h with a 90% ethanol conversion. However, the selectivity toward aromatics was 30% during the first 20 h, but then it decreased and more gaseous products were produced. The effect of water was not significant on the product distribution but it slightly decreased the ethanol conversion, as was also found by Schulz.^51,50^

Sivasanker modified H-ZSM-5 extrudates (SiO_2_/Al_2_0_3_ = 82):Al_2_0_3_ (50:50 wt%) with 3 wt% Zn and Ga for ethanol conversion at 360 °C.^52^ Compared to the parent H-ZSM-5 extrudates, incorporation of Zn and Ga increased the aromatic yields to 27.5–30.6% from 23.2% (Table 4, entries 16–18). Additionally, Zn- and Ga-ZSM-5 exhibited longer catalyst lifetime.^52^

Zeolites exchanged with alkali metals, alkaline earth metals and transition metals were also utilized for ethanol conversion.^51^ Both alkali and alkaline earth metals exchanged zeolites resulted in the preferential formation of ethene, rather than aromatics. In contrast, MgO- and GaO-impregnated H-ZSM-5 were found to be selective toward aromatics and produced 8–32 wt% yields of aromatics at 300–500 °C (Table 4, entries 19 and 20). Transition metal exchanged ZSM-5 as catalysts for ethanol conversion at 400 °C yielded 12.7–42.5 wt% of aromatics. Among them, Ni^2+^ and Cr^3+^-ZSM-5 stand out and produced 42.5 wt% and 34.1 wt% yield of aromatics respectively (entries 21 and 22), due to their high activity for ethene oligomerization.

A bimetallic catalyst supported on MFI and γ-Al_2_O_3_ that contained 0.7 wt% Pd and 0.4 wt% Zn was prepared for ethanol conversion at 330 °C and showed a stable activity over 50 h time on stream (TOS) with a yield of aromatics of 42 wt% (Table 4, entry 23) at full ethanol conversion.^53^ Under the same conditions, the use of 0.1 wt% Au and 0.06 wt% Pd on MFI and γ-Al_2_O_3_ produced a 30 wt% yield of aromatics (entry 24).^54^

In conclusion, a series of zeolites has been used as catalysts for the catalytic fast pyrolysis of ethanol and aromatics could be obtained in up to 57% yields.^41^ One main drawback of these reactions is the quick catalyst deactivation due to coke formation and the dealumination of zeolites by water. Another negative aspect is the high weight loss in a process where three molecules of ethanol form one molecule of benzene (from 138 to 78). Considering the rather poor aromatics selectivity and the multistep conversion of lignocellulose to bio-based aromatics via ethanol, the practicality of this approach to produce aromatics is low. In addition, the use of the obtained hydrocarbon oil as fuel or as fuel additive is not competitive with the direct use of ethanol.^33^

#### 2.3.2 Aromatics from Ethylene

Compared to methane (see section 8), the production of aromatics from the cyclization of alkenes such as ethylene is much more facile and can be practically achieved at significantly lower temperatures (down to 400 °C) with conversions exceeding 90% and reasonable selectivity, up to 67%, to aromatics.^55,56^ Ethylene is one of the most important base chemicals used in the chemical industry for the production of a wide range of commodities. The production of ethylene is currently based on steam cracking of fossil hydrocarbons. Three approaches can be considered for the production of ethylene from renewables. The first route involves the acid-catalyzed dehydration of bioethanol, generated by the fermentation of renewable sugars (see section 2.3.2).^57^ This is currently produced on industrial scale by Braskem GmbH,^58^ as well as by India Glycols Ltd.^59^ It is also possible to produce ethylene fermentative. This is was recently proposed as an alternative approach, although its yield and productivity are still far removed from what is needed for large-scale application.^60^

**Figure 3:**
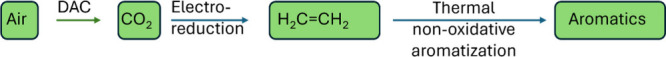
Schematic illustration of the sequence of converting CO_2_ to ethylene and further to benzenoid aromatics.

In the anticipated circular economy concepts the use of aromatics to make chemicals or as components of fuels such as gasoline should eventually lead to the release of CO_2_ once again into atmosphere. Therefore, coupling direct-air-capture (DAC) technologies with electrocatalytic conversion of CO_2_ to C_2+_ chemicals and its further valorization to aromatics and other chemicals can be considered as an important part of the future cycle of sustainable use of renewables to make chemicals (Figure 3). In this case CO_2_ is basically regarded as the building block for making these chemical commodities. The electrochemical reduction of CO_2_ to ethylene has been researched extensively. However, the methodology is currently not economic and major improvements are necessary to bring the electrolyzer potential down from above 3 V to the range of H_2_O electrolyzers (1.4–2.5 V) and to increase the stability from to-date 190 h approaching the stability of H_2_O electrolyzers (60,000 – 90,000 h).^61^ In addition, the electricity cost is too high. Even in an idealized case, the cost of ethylene produced in this way would be almost three times higher than the current (fossil-based) production costs.^62^

Lanzatech has recently announced that they can produce ethylene in a fermentation process using a modified organism that is fed with a mixture of CO_2_, H_2_, O_2_, and N_2_. However, the amounts of produced ethylene in the gas stream were in the order of 2000 ppm, which seems very far removed from an industrial process.^63^

There are several reports in the literature about the conversion of ethylene to aromatics which are summarized in Table 5.

**Table 5:** Catalytic Conversion of Ethylene to Aromatics

Entry	Catalyst (metal loading)	Structural features of the catalyst	Reaction conditions (gas mixture, temperature, space velocity, or flow rate)	C_2_H_6_ conversion (%)	Product selectivity	Analysis	Ref
1	Ga/H-ZSM-5 (0.025 wt%)	Si/Al = 60	100% C_2_H_6_; 500 °C	96.7 ± 0.4	Benzene: ∼24.4%	GC	(56)
					Aromatics: 67.1%		
2	Ga/ZSM-5	Si/Al = 24	12.7% C_2_H_6_/Ar; 3000 mL g^–1^ h^–1^; 1 atm; 400 °C	84.0	Benzene: ∼6%	GC	(64)
		Si/Ga = 57			Toluene: ∼25%		
					Xylene: ∼19		
					BTX: ∼50%		
3	Ga/H-ZSM-5 (1.4/4.7 wt%)	Si/Al = 35	5 mol% C_2_H_4_/N_2_; 3100 mL g^–1^ h^–1^; 1 atm; 400 °C	65/58	Aromatics: 30.1%/20	GC	(65)
		Si/Ga = 80.4/24.1					
4	Mo/H-ZSM-5	Si/Al = 24	5 mol% C_2_H_4_/N_2_; 1.21 h^–1^; 700 °C	95 (50 min)/35 (400 min)	Benzene: ∼25 to >6%	GC	(66)
					Aromatics: 18 to >15%		

Ethylene dehydroaromatization requires acidic zeolitic material or a bifunctional catalyst made of acidic support, basically zeolitic material and a metal center. Earlier reports by Dufresne et al. demonstrated the potential of using additive-free H-ZSM-5 for the non-oxidative dehydroaromatization of ethylene.^55^ On this zeolite, ethylene conversion at 500 °C is about 92.5% with a product selectivity for aromatics of around 34.5%, while the selectivity for the C_1_–C_4_ paraffins is 39.5%. The rest of the products is a mixture of olefins and C_5+_ aliphatic hydrocarbons. These authors showed also that the use of ZnO/Al_2_O_3_ catalysts can achieve a similar ethylene conversion at 500 °C but with much higher selectivity toward aromatics (between 54 and 65%).

**Figure 4:**
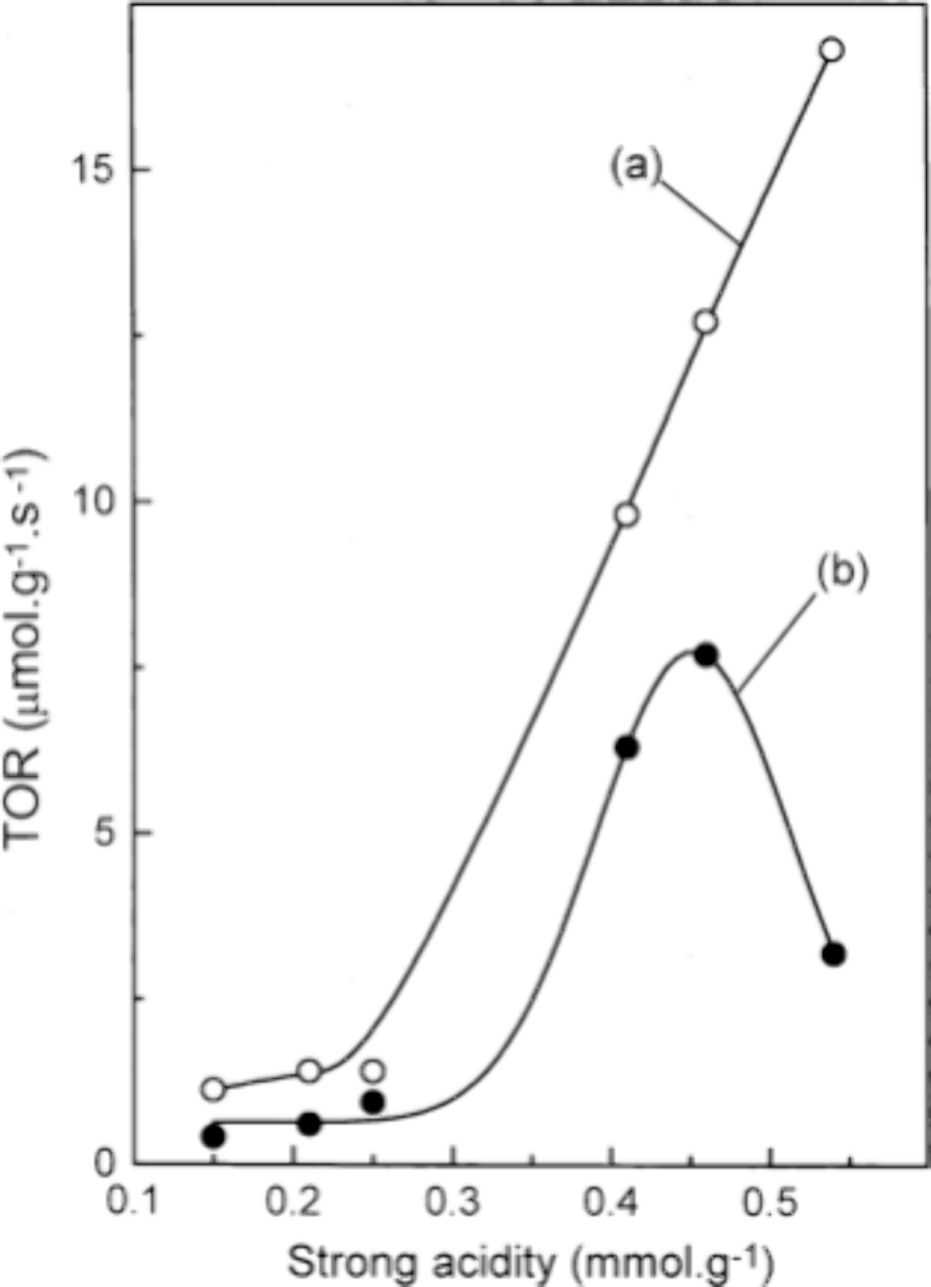
Dependence of turnover reaction rate (TOR) for (a) ethylene conversion and (b) ethylene-to-aromatics conversion in the ethylene aromatization over the Ga-modified ZSM-5 type zeolites at the ethylene iso-conversion of 60% on their strong acidity. Reproduced with permission from ref (65). Copyright Elsevier 2001.

The combination of Ga with H-ZSM-5 showed in particular high potential as catalyst for ethylene dehydroaromatization (Table 5, entry 2).^64^ Choudhary et al. studied Ga supported on H-ZSM-5, HMFI, or HAlMFI zeolitic frameworks for ethylene dehydroaromatization at a significantly lower temperature (400 °C) (Table 5, entry 3),^65^ than that reported by Dufresne et al.^55^ These authors found out that the use of either H-GaMFI or H-GaAlMFI as catalysts resulted in higher selectivity toward aromatics than the use of Ga-H-ZSM-5M as catalyst. At this temperature conversions >90% could be achieved at a GHSV lower than 5000 cm^3^ g^–1^ h^–1^ for both H-GaAlMFI catalysts with selectivity to aromatics exceeding 65%. Both H-GaMFI and Ga-H-ZSM-5 showed conversions up to 65% ethylene under the same conditions. The selectivity toward aromatics is however much more limited on the latter ZSM-5 supported Ga catalyst (about 30% total aromatics) compared to the H-Ga-MFI (65% total aromatics obtained). In this study, these authors also quantified the impact of acid sites on catalytic activity and the selectivity toward aromatics. Results showed that ethylene conversion increases continuously with the increase of acidity while the selectivity toward aromatics depends on an optimum concentration of acid sites (Figure 4).

Zhou et al. showed that the selectivity toward aromatics can be varied between 15 to about 52% depending on the nature of the Ga species and how they are dispersed and bound to the ZSM-5 framework.^64^ While the lowest aromatics selectivity is related to the lack of Lewis acid sites in the catalysts (or specifically the extra-framework Ga), the catalysts characterized by the highest aromatics selectivity were based on highly dispersed Ga oxo species binding to or stabilized on Brønsted acidic sites in the ZSM-5 framework. These catalysts showed a continuous deactivation with TOS, with varying rates depending also on the nature of the support material. In this regard the extra-framework Ga-based catalyst showed the highest deactivation rates, deactivation, essentially decaying from 80% ethylene conversion to about 40% over 22 h TOS. Considering a 40% conversion and a selectivity toward aromatics of 52% these catalysts have aromatics yield of 20%. Lu et al. also studied the activity of Ni supported into MWW zeolites (MCM-22 and ERB-1) for ethylene oligomerization and aromatization but at significantly lower temperatures than that used in studies discussed above.^67^ A conversion of about 33% was observed at 300 °C. At this temperature mostly ethylene oligomers were formed and no aromatics. Interestingly this catalyst showed almost no deactivation for over 4 h on stream which is different from the standard behavior observed during methane dehydroaromatization on Mo supported by the MMW type zeolite.^68^ Aromatics were only formed at 500 °C, but conversion (from 12% to 2%) and selectivity (from 30% to 8%) rapidly decayed, even after 2 h.

Volmer et al. investigated the conversion of ethylene under identical conditions to that used in the methane dehydroaromatization (i.e., at 700 °C at atmospheric pressure) over a Mo/HZS-5 catalyst (Table 5, entry 4).^66^ These authors aimed at answering the open question of whether methane is aromatized to benzenoids via ethylene as intermediate or if it proceeds via a direct pathway to benzenoids. Based on this study the outcome of the reaction products indicated much lower selectivity toward aromatics from ethylene compared to the methane MDA reaction on the same catalyst under identical reaction conditions (see, e.g., entry 5, Table 48),^69^ which suggests that the reaction has a different mechanism and probably also proceeds via different intermediates. To the best of our knowledge there is no systematic experimental study which clarifies the reaction mechanism for this reaction.

**Figure 5:**
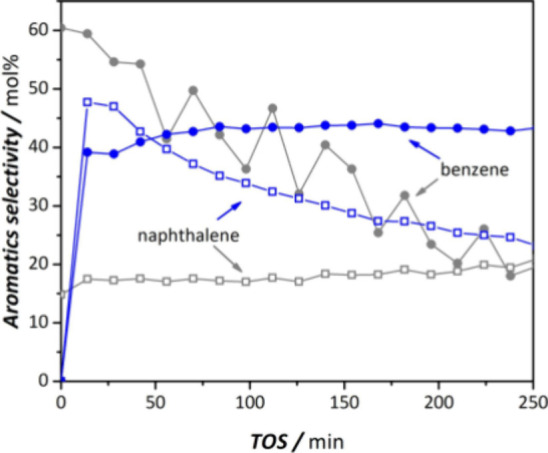
Comparison of selectivity to aromatics when feeding mixtures of 95 mol%CH_4_/5 mol%N_2_ over Mo/H-ZSM-5 (blue symbols) or 5 mol% C_2_H_4_/95 mol% N_2_ over H-ZSM-5 (gray symbols) at ambient pressure and at a reaction temperature of 700 °C for both mixtures (benzene: solid circles; naphthalene empty squares). Total flow rate in both cases 15 mL/min. Reproduced with permission from ref (66). Copyright 2020 Wiley.

Interestingly, the reaction behavior with TOS (i.e., activation/deactivation) is different for both catalyst (Figure 5), which is further evidence for the different reaction intermediates/mechanisms. The authors observed the formation of significant amounts of naphthalene from methane under these conditions whereas feeding ethylene resulted in lower yields to naphthalene. It can thus be inferred that it is less likely that ethylene is an intermediate in the methane dehydroaromatization, in spite of the fact that its addition can enhance the dehydroaromatization of methane itself as discussed in section 8. It can be concluded that the aromatization of mixtures of methane and ethylene would be a more practical option for the production of aromatics both in terms of selectivity toward aromatics as well as allowing a lower reaction temperature.

#### 2.3.3 Aromatics from 1-Butanol

Most 1-butanol is produced via hydroformylation of propene to butyraldehyde, which is hydrogenated to 1-butanol. 1-Butanol can also be obtained from renewables via fermentation using a clostridium microorganism in the so-called ABE process which produces acetone, 1-butanol, and ethanol.^70^ The process has been in production since 1912, mainly for the acetone that was necessary to make explosives. The 1-butanol found application in paints. In the 1950s most of these fermentation processes were ceased, as production based on fossil fuels was cheaper. Today, there is an increased interest in 1-butanol as a biofuel as it has better properties than bioethanol. For this reason, the ABE fermentation has been started up again in China.

Several groups have investigated the conversion of 1-butanol into aromatics, often in an attempt to convert biobutanol into a mixture of hydrocarbons suitable to be used as fuel. All reactions were performed in a fixed-bed reactor. In some cases, the effect of pressure was investigated, but in all cases it was found that best selectivities to BTX were obtained at 1 bar. In all cases 1-butanol conversion was >99% (Table 6).

**Table 6:** Conversion of 1-Butanol to Aromatics by Catalytic Fast Pyrolysis

Entry	Catalyst	Si/Al	*T* ( °C)	WHSV (h^–1^)	TOS (h)	Selectivity to BTX (%)	B:T:X	Ref
1	H-ZSM-5	55	400	0.75	1	27^a^	4:13:10^a^	(71)
2^b^	H-ZSM-5	55	350	0.75	28	29	3:12:14	(72)
3^b^	γ-Al_2_O_3_	–	350	0.75	28	0	–	(72)
4^b^	H-β	30	350	0.75	28	12	–	(72)
5	H-ZSM-5	20	400	0.3	1–8	35^a^^,^^c^	–	(73)
6	H-ZSM-11	20	400	0.3	1–8	33^a^^,^^c^	–	(73)
7	H-L	2.9	400	0.3	1–8	13^a^^,^^c^	–	(73)
8	H-Y	–	400	0.3	1–8	14^a^^,^^c^	–	(73)
9	5Zn-H-ZSM-5	55	500	0.75	6	68	12:32:24	(74)
10	5Ga-H-ZSM-5	55	600	0.75	1	69^d^	31:29:8	(75)
11	Cu_20_MMO	–	600	Not given	3	12	3:6:9	(76)
12^e^	H-ZSM-5	36	450	Not given		29	2:11:16	(77)

a GC data without internal standard.

b Pressure = 20 bar.

c Aromatics selectivity determined by NMR.

d 1 mol% of ethylbenzene was also formed.

e A mixture of 1-butanol and acetone was used.

Shee and co-workers examined the catalytic fast pyrolysis (CFP) of 1-butanol over ZSM-5 (80).^71^ Below 300 °C no aromatics were formed. At 300 °C, initially mostly C9 is formed and smaller amounts of BTX. At higher temperatures up to 400 °C this ratio changes and more BTX is formed. Product ratios at 5 different WHSVs (14.96–0.75 h^–1^) were measured and the highest BTX selectivity was obtained at the lowest WSVH (Table 6, entry 1). The same group tested the H-ZSM-5 (55) catalyst at 20 bar and 350 °C and compared the results (29% selectivity to BTX) with those obtained with ZSM catalysts with lower and higher Si/Al ratios (Table 6, entries 2–4).^72^ Both led to somewhat lower selectivities to BTX. The results were also compared to those obtained with γ-Al_2_O_3_, where no aromatics were formed and H-β were 12% BTX was obtained.

Varvarin and co-workers tested 4 different zeolites, H-ZSM-5, H-ZSM-11, H-L, and H-Y, in the CFP of 1-butanol.^73^ Highest aromatic yields were obtained with H-ZSM-5 (35%) and H-ZSM-11 (33%) (Table 6, entries 5–8).

Shee and co-workers examined the effect of doping the H-ZSM-5 catalyst with different amounts of zinc and found that 5% of Zn gave optimum results.^74^ The doping with Zn resulted in a shift from aliphatic to aromatic hydrocarbons and under the optimum conditions a 68% selectivity was obtained to BTX (Table 6, entry 9). The same group also investigated the effect of doping with gallium and found that the catalyst with 5 mol% of gallium gave the best results.^75^ At a temperature of 550 °C a BTEX selectivity of 69% was obtained (Table 6, entry 10). The authors explained these effects by assuming that these metals catalyzed the dehydrogenation of cycloaliphatic compounds to aromatics.

Metzker and co-workers used copper–magnesium–aluminum mixed metal oxide, derived from the hydrotalcite precursor in which 20 mol% of magnesium was replaced by Cu^2+^ ions (Cu_20_MMO), which is a known catalyst for the Guerbet reaction, for the conversion of 1-butanol at 500 and 600 °C (Table 6, entry 11).^76^ This results in a mixture of alkenes, alcohols, aldehydes, and aromatics. At 600 °C a selectivity 0f 25% to aromatics was achieved with 17.5% selectivity to BTX. Co-feeding the reaction with methanol led to a lower yield of only 13% aromatics and methyl ethers were formed in addition to the products found earlier. The authors assume that the reaction follows the initial steps of the Guerbet reaction, but the expected intermediates were not found. In addition, the substitution pattern of the aromatics cannot be explained via a Guerbet mechanism.

Aguado and co-workers investigated the CFP of a mixture of acetone and 1-butanol using H-ZSM-5 as catalyst (Table 6, entry 12). This approach offers a direct conversion of the two components from the ABE process and obviates the need for their separation. The results (29% BTX) were not greatly different from those obtained without acetone.^77^

Once 1-butanol becomes available on large scale from renewables, this could be an interesting route in view of the relatively high aromatic selectivities and the low oxygen content.

#### 2.3.4 Aromatics from Isobutanol/Isobutanal/Isobutylene

Isobutanol is mostly used as solvent for organic chemicals. The production of isobutanol from carbohydrates has been achieved by fermentation in high efficiency.^78−81^ The American company Gevo has upgraded its ethanol production plant in Luverne (USA) for the “side-by-side” production of both ethanol and isobutanol from corn on a scale of 5.7 million liters per year.^79,82^ Isobutanol can be used as a fuel additive and as precursor for the synthesis of valuable chemicals, including isobutene, butene, and aromatics.^79,81^ A Review from 2021 has summarized the conversion of isobutanol to hydrocarbons.^79^

**Scheme 4:**
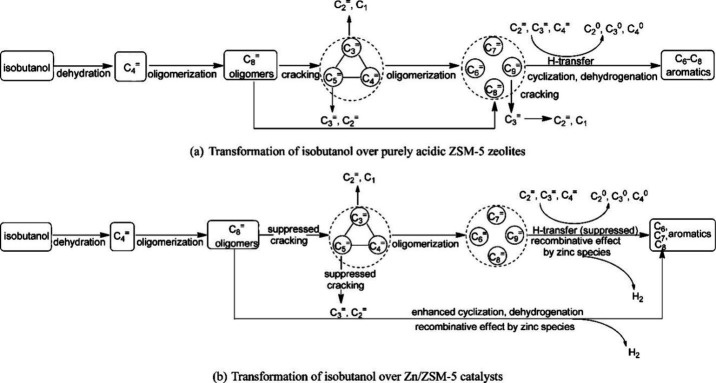
Transformation of Isobutanol to Aromatics over Zeolites Reproduced with permission from ref (84). Copyright 2012 American Chemical Society.

Bio-based aromatics can be obtained from zeolite-catalyzed pyrolysis of isobutanol. Table 7 summarizes the reports on aromatics production in >20% yield via the CFP of isobutanol. In 1989, Le Van Mao studied the transformation of isobutanol catalyzed by H-ZSM-5 and Zn-incorporated H-ZSM-5 at 390–470 °C and observed the formation of 13.5–22.9 wt% aromatic products.^83^ Both the addition of Zn (Zn/H-ZSM-5) and the use of chrysotile asbestos (Zn/Hasb-ZSM-5) as catalyst slightly improved the aromatic yields and the BTX selectivity.^83^ Xu and Huang investigated the transformation of isobutanol to aromatics by employing acidic zeolites with or without metal promoters.^84^ ZSM-5 with different Si/Al ratios (13.3–42.7) was used as catalysts for the pyrolysis of isobutanol at 450 °C. This resulted in the production of aromatics in 41.6–42.3 wt% yields, along with 20.7–25.3 wt% yields of propane and 19.0–21.4 wt% yields of isobutane as main side products. The authors proposed a pathway for the transformation of isobutanol into aromatics (Scheme 4a). Under acidic conditions, isobutanol dehydrates to isobutene and butene. The formed C4 oligomerizes to C8 species, which were converted to aromatics at high temperature through a series of reactions, including multiple oligomerization–cracking reactions, cyclization, and dehydrogenation. The incorporation of Zn to ZSM-5 raised the aromatics yield to 54.2–59.3 wt%, and lowered the formation of propane (4.3–10.0 wt%) and isobutane (10.0–15.6 wt%) by suppressing the oligomerization-cracking reactions due to reduced Brønsted acidity (Scheme 4b).^84^ It was proposed that the Zn species promote the dehydrogenation of C7 and C8 intermediates, resulting in higher yields of toluene and xylene.^84^ The impregnation of Ga, Mo, La, and Ag to ZSM-5 showed very limited effects on the aromatics formation.^84^

**Table 7:** Catalytic Fast Pyrolysis of Isobutanol to Aromatics—Products Analyzed by GC and GC-MS

							Aromatic distribution (wt%)			
Entry	Catalyst (Si/Al ratio)	WHSV (h^–1^)	TOS (h)	*T* (°C)	*P* (bar)	Aromatics yield (wt%)	Benzene	Toluene	Xylene	Ref
1	H-ZSM-5 (19)^d^	2.5	–b	470	6.9	21.1		16.3^c^		(83)
2	H-ZSM-5 (19)^d^	2.5	–b	470	17.2	21.8		15.5^c^		(83)
3	Zn/H-ZSM-5 (19)^d^	2.5	–b	470	17.2	21.2		15.1^c^		(83)
4	Zn/Hasb-ZSM-5 (14)^d^	2.5	–b	470	6.9	20.2		16.2^c^		(83)
5	Zn/Hasb-ZSM-5 (14)^d^	2.5	–b	470	17.2	22.9		17.4^c^		(83)
6	ZSM-11 (26.6)	3.88	–b	450	1.0	41.9	3.9	14.3	13.1	(84)
7	ZSM-5 (13.3)	3.88	–b	450	1.0	41.6	4.2	15.2	13.5	(84)
8	ZSM-5 (34.3)	3.88	–b	450	1.0	42.3	4.3	15.7	14.3	(84)
9	ZSM-5 (42.7)	3.88	–b	450	1.0	42.3	4.3	15.5	13.9	(84)
10	2.3% Zn/ZSM-5 (34.3)^e^	3.88	–b	450	1.0	59.3	3.8	24.3	21.2	(84)
11	2.1% Ga/ZSM-5 (34.3)^e^	3.88	–b	450	1.0	42.9	4.6	16.6	14.4	(84)
12	2.4% La/ZSM-5 (34.3)^e^	3.88	–b	450	1.0	43.6	4.2	16.2	18.4	(84)
13	2.1% Mo/ZSM-5 (34.3)^e^	3.88	–b	450	1.0	42.0	4.5	15.8	13.7	(84)
14	2.3% Ag/ZSM-5 (34.3)^e^	3.88	–b	450	1.0	43.2	4.5	16.1	14.4	(84)
15	0.4% Pt/ZSM-5-R (34.3)^f^	3.88	–b	450	1.0	42.5	4.6	16.2	14.0	(84)
16	2.1% Ga/ZSM-5-R (34.3)^f^	3.88	–b	450	1.0	43.7	4.5	16.6	14.6	(84)
17	2.1% Mo/ZSM-5-R (34.3)^f^	3.88	–b	450	1.0	46.2	4.9	17.5	15.4	(84)
18	0.8% Zn/ZSM-5 (34.3)^e^	3.88	–b	450	1.0	54.2	4.5	22.9	18.1	(84)
19	5.1% Zn/ZSM-5 (34.3)^e^	3.88	–b	450	1.0	61.4	4.1	25.9	22.0	(84)
20	9.0% Zn/ZSM-5 (34.3)^e^	3.88	–b	450	1.0	58.7	3.7	24.6	21.2	(84)
21	2% Ga/ZSM-5 (12.5)	1.25	–b	350	1.01	21.8^g^	0.9	6.0	11.6	(85)
22	2% Ga/ZSM-5 (12.5)	1.25	–b	400	1.01	29.7^g^	3.0	11.4	14.2	(85)
23	2% Ga/ZSM-5 (12.5)	1.25	–b	450	1.01	38.3^g^	8.8	16.8	12.1	(85)
24	2% Ga/ZSM-5 (12.5)	1.25	–b	500	1.01	51.2^g^	22.8	20.1	7.7	(85)
25	2% Ga/ZSM-5 (12.5)	1.25	–b	550	1.01	56.2^g^	32.2	18.7	4.2	(85)
26	ZSM-5 (12.5)	1.25	–b	400	1.01	19.4^g^	2.4	6.7	9.2	(85)
27	0.5% Ga/ZSM-5 (12.5)	1.25	–b	400	1.01	24.5^g^	2.6	9.0	11.8	(85)
28	2% Ga/ZSM-5 (12.5)	1.25	–b	400	1.01	27.7^g^	2.8	10.5	13.4	(85)
29	4% Ga/ZSM-5 (12.5)	1.25	–b	400	1.01	32.4^g^	3.1	12.3	15.7	(85)
30	6% Ga/ZSM-5 (12.5)	1.25	–b	400	1.01	32.5^g^	3.0	12.2	15.9	(85)
31	8% Ga/ZSM-5 (12.5)	1.25	–b	400	1.01	28.7^g^	2.6	10.9	13.9	(85)
32	2% Ga/ZSM-5-reduced (12.5)	1.25	–b	400	1.01	32.2^g^	3.2	12.3	15.6	(85)
33	2% Ga/ZSM-5-reduced (12.5)	1.25	–b	400	1.01	35.1^g^	3.2	13.4	17.0	(85)
34	MFI/MCM-41	1.9	–b	400	1.01	25	0.5	3	5	(87)
35	MFI/MCM-41	1.9	–b	500	1.01	24	0.5	3	5	(87)
36	ZnCrMFI/MCM-41	2.3	2 h	400	1.01	26	–	–	5	(88)
37	ZnCrMFI/MCM-41	2.3	2 h	450	1.01	27	–	–	9	(88)
38	ZnCrMFI/MCM-41	2.3	2 h	500	1.01	26	–	–	8	(88)
39	ZnCrMFI/MCM-41	2.3	2 h	550	1.01	29	–	–	7	(88)
40	MFI-136 (68)	3.3^a^	2 h	400–450	1.01	25–31	–	–	–	(89)
41	ZnCrMFI-136 (68)	2.4^a^	2 h	450–550	1.01	25–29	–	–	–	(89)
42	ZnCrMFI-40 (20)	2.1^a^	2 h	400–500	1.01	26–36	–	–	–	(89)
43	ZnCrMFI-40 (20)	2.1^a^	2 h	550	1.01	40	9	21	9^h^	(89)
44	H-ZSM-5 (25)	1.74^a^	3 h	400	1.01	44.3	2.7	14.8	17.7	(90)
45	H-ZSM-5 (25)	1.74^a^	3 h	400	1.01	47.2	3.2	16.4	18.6	(90)
46	Zn/ZSM-5 (25)	1.74^a^	3 h	400	1.01	45.9	3.3	16.7	18.6	(90)
47	Zn/ZSM-5 (25)	1.74^a^	3 h	400	1.01	53.5	3.4	18.6	22.2	(90)
48	Ga/ZSM-5 (25)	1.74^a^	3 h	400	1.01	51.0	2.1	16.2	22.2	(90)
49	Ga/ZSM-5 (25)	1.74^a^	3 h	400	1.01	59.4	2.9	20.9	26.0	(90)

a LHSV (liquid hour space velocity) was given.

b Not reported.

c Yield of BTX mixture.

d Diluted with 20 wt% bentonite.

e Pretreated at 60 °C.

f Pretreated at 100 °C.

g Total yields of benzene, toluene, xylene, ethylbenzene, and styrene.

h Ethylbenzene included.

In 2019, Du and Li prepared a series of Ga impregnated H-ZSM-5 catalysts for the conversion of isobutanol to olefins and aromatics which they tested at various temperatures. At ≤300 °C, the H-ZSM-5-catalyzed CFP of isobutanol only produced very small amounts of aromatics (<10 wt%) and olefins were the major products (>45 wt%) with a dominant amount of butene (>31 wt%). The aromatic yields increased to 17.6 wt% at 400 °C and then decreased when the temperature was raised further. Compared to H-ZSM-5, the use of Ga-loaded ZSM-5 had a positive effect on the formation of aromatics (24.5–32.5 wt%). Increasing the amount of Ga from 0.5% to 8% showed limited influence on the formation of aromatics, suggesting that Ga-Brønsted acid site pairs were the actual active sites for the aromatics formation, which is limited by the fixed number of Brønsted acid sites inside the zeolites. Higher temperatures raised the aromatic yields, which was attributed to the enhanced dehydrogenation by the Ga-Brønsted acid sites.^85^

Only zeolites with MFI and MEL (The codes and related structures can be found in the Database of Zeolite Structures.)^86^ topologies effectively catalyze aromatic formation from isobutanol.^84^ Moiseev studied MFI-types zeolites for the formation of aromatics from isobutanol via pyrolysis.^87−89^ Loktev and Moiseev used a micro-mesoporous MFI/MCM-41 catalyst for isobutanol pyrolysis at 400 °C and obtained 25 wt% aromatics.^87^ Karavaev and Moiseev modified the micro-mesoporous MFI/MCM-41 catalyst with Zn and Cr, which catalyzed the aromatics formation from isobutanol in 7–29 wt% yields at 320–550 °C.^88^ An improved aromatics yield (40 wt%) was obtained from isobutanol when ZnCrMFI-40 with a Si/Al ratio of 20 was used as catalyst.^89^

Inert gases, including nitrogen and helium, are often used as carrier gas for the CFP of isobutanol in fixed-bed reactors. In 2017, Park used CO_2_ as carrier gas for isobutanol pyrolysis catalyzed by H-ZSM-5 and Zn- or Ga-promoted ZSM-5.^90^ Compared to the reaction performed under He, CO_2_ as carrier gas enhanced the aromatics formation with all tested zeolites. Ga/ZSM-5 showed a superior behavior compared to untreated H-ZSM-5 and Zn/ZSM-5 in the aromatics production, which was inconsistent with Xu’s work.^84^ The author proposed that the Ga species increased the Lewis acidity and decreased the Brønsted acidity of the catalyst. The formation of monodentate and bidentate carbonates, which was detected by FT-IR and XPS, confirmed the interaction of CO_2_ with the Ga species. It was proposed that CO_2_ reacts with hydrogen in the water gas shift reaction, thus advancing the dehydrogenation of cycloalkanes to aromatics.^90^

Isobutanal can be obtained from glucose or CO_2_ by fermentation.^91,92^ The transformation of isobutanal to bio-based aromatics was demonstrated by Palkovits and co-workers.^93^ Y zeolite, BEA zeolite, and Mordenite were tested for isobutanal conversion at 400 °C. Only H-ZSM-5 delivered high aromatics yields with BTX as major components, consistent with Xu’s conclusion that the MFI and MEL types of zeolite give high yields of aromatics.^84^ Optimization of temperature, WHSV, and Si/Al ratios provided 78.8–93.3 mol% yields of aromatics along with 61.5–78.9% yields of BTX from isobutanal catalyzed by H-ZSM-5 with medium Si/Al ratios (15–130) at 400 °C (Table 8, entries 1–9).^93^

**Table 8:** Aromatics Production by CFP of Isobutanal or Isobutene

								Aromatic distribution (yield%)			
Entry	Feed	Catalyst (Si/Al ratio)	WHSV (h^–1^)	TOS (h)	*T* (°C)	Analytical method	Aromatics yield (mol%)	Benzene	Toluene	Xylene	Ref
1	isobutanal	H-ZSM-5 (15)	3.0	2.5	400	GC-MS	93.3	11.8	36.3	30.8	(93)
2	isobutanal	H-ZSM-5 (33)	3.0	2.5	400	GC-MS	88.2	9.8	33.2	30.8	(93)
3	isobutanal	H-ZSM-5 (40)	3.0	2.5	400	GC-MS	90.1	8.2	31.4	34.6	(93)
4	isobutanal	H-ZSM-5 (49)	3.0	2.5	400	GC-MS	89.3	7.9	30.7	32.3	(93)
5	isobutanal	H-ZSM-5 (130)	3.0	2.5	400	GC-MS	78.8	6.4	26.3	28.8	(93)
6	isobutanal	H-ZSM-5 (40)	3.0	1.0	400	GC-MS	62.3	7.7	25.2	17.2	(93)
7	isobutanal	H-ZSM-5 (40)	3.0	1.5	400	GC-MS	89.8	10.2	33.8	30.4	(93)
8	isobutanal	H-ZSM-5 (40)	3.0	2.0	400	GC-MS	84.6	8.9	31.2	30.3	(93)
9	isobutanal	H-ZSM-5 (40)	3.0	3.0	400	GC-MS	79.3	5.7	25.3	32.6	(93)
10	isobutene	3 wt% AlH_3_/SiO_2_ (4)	600^a^	–	500	–	20.6	3.8^b^		9.0	(94)
11	isobutene	H-ZSM-5 (30)	–	1.5	400	GC	25.4	6.6^c^	31.4^c^	26.4^c^	(95)
12	isobutene	H-ZSM-5 (30)	–	1.5	500	GC	32.9	12.9^c^	36.3^c^	29.2^c^	(95)
13	isobutene	(1/2) Ga_2_O_3_/H-ZSM-5 (30)	–	1.5	500	GC	31.3	11.9^c^	37.6^c^	26.8^c^	(95)
14	isobutene	(1/1) Ga_2_O_3_/H-ZSM-5 (30)	–	1.5	500	GC	33.5	10.2^c^	37.5^c^	28.2^c^	(95)
15	isobutene	(2/1) Ga_2_O_3_/H-ZSM-5 (30)	–	1.5	500	GC	33.6	11.5^c^	36.3^c^	29.4^c^	(95)
16	isobutene	(1/2) ZnO/H-ZSM-5 (30)	–	1.5	500	GC	38.9	10.4^c^	39.4^c^	25.8^c^	(95)
17	isobutene	(1/1) ZnO/H-ZSM-5 (30)	–	1.5	500	GC	33.2	10.4^c^	39.2^c^	26.7^c^	(95)
18	isobutene	(2/1) ZnO/H-ZSM-5 (30)	–	1.5	500	GC	23.5	10.3^c^	41.3^c^	28.5^c^	(95)
19	isobutene	(1/2) CuO/H-ZSM-5 (30)	–	1.5	500	GC	25.1	17.0^c^	45.4^c^	29.5^c^	(95)
20	isobutene	(1/1) CuO/H-ZSM-5 (30)	–	1.5	500	GC	29.3	14.7^c^	43.6^c^	28.5^c^	(95)
21	isobutene	(2/1) CuO/H-ZSM-5 (30)	–	1.5	500	GC	20.8	13.5^c^	44.9^c^	33.1^c^	(95)

a GHSV (gas hour space velocity).

b Yield of benzene and toluene.

c Selectivity was given.

Isobutene can be produced by dehydration of isobutanol. It is presumably the key intermediate for aromatics formation from isobutanol.^84,85^ The conversion of isobutene to aromatics (Table 8 entries 10–21) can be traced back to 1982, when Slaugh reported 20.6% aromatics yield at 500 °C using a catalyst that was prepared by treating silica with 3 wt% AlH_3_ followed by heating in a flow of N_2_ at up to 700 °C.^94^ In 2010, Hutchings synthesized a range of catalysts based on the combination of metal oxides, including Ga_2_O_3_, ZnO, and CuO, with H-ZSM-5. These catalyzed aromatics formation from isobutene in 20.8–38.9% yields.^95^ ZnO/H-ZSM-5 delivered the highest aromatics yields (23.5–38.9%), followed by Ga_2_O_3_/H-ZSM-5 (31.3–33.6%). Interestingly, an increased loading of ZnO (1/2 to 2/1) on H-ZSM-5 reduced the aromatics yields (38.9% to 23.5%), whereas increased ratios of Ga_2_O_3_ (1/2 to 2/1) enhanced the aromatics formation (31.3 to 33.6%).^95^

Diisobutylene, typically a mixture of 2,4,4-trimethyl-1-pentene and 2,4,4-trimethyl-2-pentene, can be obtained by dimerization of isobutene using an ion exchange resin as catalyst.^96,97^ Conversion of diisobutylene to aromatics was investigated with non-supported metal catalysts.^98^ A Cr-Mg-Al-O catalyst, prepared by stepwise impregnation of 5% Cr and 0.75–3% Mg to Al_2_O_3_, converted diisobutylene at 500 °C to aromatics in 19.8–19.9% yields with 98% selectivity to *p*-xylene.^98^

**Scheme 5:**
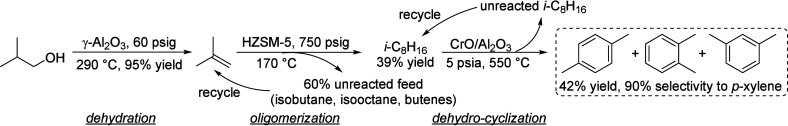
Gevo’s Integrated System to Convert Isobutanol to *p*-Xylene^99^

The American company Gevo, Inc. patented a catalytic route for the conversion of isobutanol to *p*-xylene via isooctene as intermediate (Scheme 5).^99^ In a fixed-bed reactor, 15 wt% of isobutanol as aqueous solution was dehydrated over γ-Al_2_O_3_ at 290 °C under a pressure of 60 psig to isobutene in 95% yield. H-ZSM-5-catalyzed oligomerization of isobutene, which was combined with recycled isobutane, isooctane, and butenes, at 170 °C under a pressure of 750 psig produced 39% yield of isooctene. Dehydrocyclization of isooctene, combined with recycled isooctene, over CrO-doped Al_2_O_3_ at 550 °C under a pressure of 5 psia gave a 42% yield of xylenes with 90% selectivity to *p*-xylene.^99^

#### 2.3.5 Aromatics from Acetone

The most important source of renewable acetone is the acetone–butanol–ethanol (ABE) fermentation process.^100−102^ During the (preferential) production of butanol,^103^ considerable amounts of acetone are produced, which can be made available by various separation methods^104,105^ and subjected to further use, also for aromatization. Microorganisms important for this belong to the genus *Clostridium*, such as *C. acetobutylicum*, *C. beijerinckii*, *C. saccharoacetobutylicum*, *C. aurantibutyricum*, and *C. sporogenes*. Interestingly, a plethora of substrates can be used,^100^ such as starch- and sugar-containing materials (first generation), but also lignocellulose (second generation), although the latter feedstock must undergo pretreatment. In this process, the structure is made accessible to the microorganisms and hydrolytically pretreated to gain soluble sugars. After distillative processing, ethanol, butanol and also acetone are available separately.

**Scheme 6:**
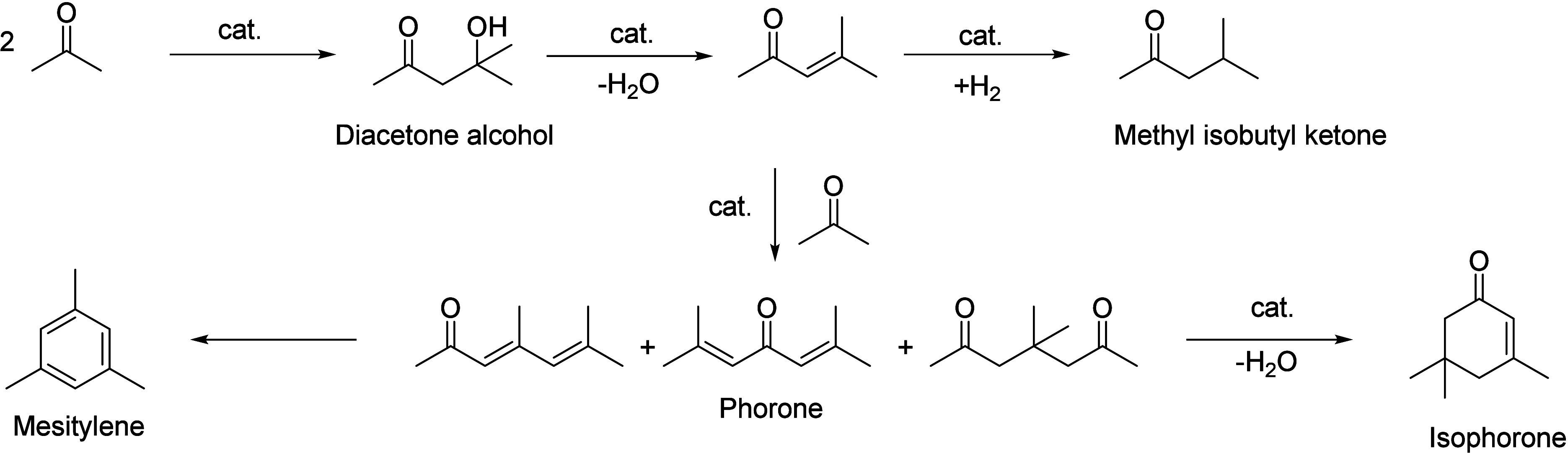
Self-Aldol Condensation of Acetone to Mesitylene and Isophorone

Generally, ketones can be converted into longer-chain molecules by C–C bond linkage through aldol condensation. Of particular interest in this reaction is that oxygen is removed from the organic compounds in the form of water, thus increasing the C/O ratio. In this way, unsaturated ketones are formed which can be further converted to aromatics. Thus, acetone from the ABE process can also be converted into aromatics.^106−109^ In the process, different partial reactions take place, leading to a complex overall picture of the reaction. A detailed description of the underlying processes was already provided by Salvapati et al.^106^ in 1989. The main reaction pathways are depicted in Scheme 6 following a paper by Wu et al.^110^

In this process, two molecules of acetone react over acidic/basic catalysts to form diacetone alcohol, which after dehydration can be converted to mesityl oxide. Mesityl oxide is a pivotal intermediate, the formation of which is of high interest for the subsequent reactions for aromatics formation. Therefore, this reaction has been extensively investigated; the main literature data are listed in Table 9. It can be obtained preferably at lower temperatures over solid or liquid acid/base catalysts. It is important to note that this reaction is an equilibrium reaction and tends to occur at lower temperatures compared to the following steps. Water withdrawal promotes product formation, and any hydrogen additions (as in deoxygenations) are rather unfavorable, as these promote the formation of alkanes^111,112^ and thus these fractions are partially lost to aromatization. Mesityl oxide reacts further with acetone to form a mixture of phorone, 4,4-dimethyl-2,6-heptadione, and 4,6-dimethyl 3,5-heptadien-2-one. 4,4-Dimethyl-2,6-heptadione can react further to form isophorone, whereas 4,6-dimethyl 3,5-heptadien-2-one can ring-close and dehydrate to form mesitylene. Mesitylene is an interesting aromatic compound which is used as a solvent, particularly in the electronics industry. It is obtained at higher temperatures of aldol condensation using mineral or solid acid catalysts (Table 10). Isophorone is a precursor for 3,5-xylenol; hence, the reported methods for its production have been listed in Table 11, and its conversion to 3,5-xylenol in Table 12. The data in the tables are taken from ref (106) and papers which appeared after the appearance of this review.

**Table 9:**
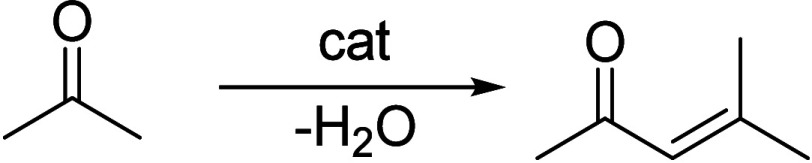
Condensation of Acetone to Mesityl Oxide

Entry	*T* ( °C)	*P* (bar)	Reactor	Time	Catalyst	Conv (%)	Sel (%)	Yield (%)	Ref
1	RT		batch	n.a.	H_2_SO_4,conc._			25.0	(106)
2	RT		batch	12 h	POCl_3_			17.5	(106)
3	Reflux				H_3_PO_4_		100		(106)
4	RT		batch	21 d	HCl_anhydr._			75.0	(106)
5	RT		batch	n.a.	AlCl_3_			62.0	(106)
6	150		batch	5 h	BeCl_3_			19–27	(106)
7	0–10		batch	16 h	ZnCl_2_			49.1	(106)
8	RT		batch	0.8 h	SiCl_4_ on Zn		60	25.9	(106)
9	110	6	Batch/continuous	n.a.	Sulfonic cation exchanger		27.1	6.6	(106)
10	55		Fixed-bed	20–40 min contact time	Anion exchange resin			55.0	(106)
11	120		Fixed-bed		Pd-Zn over Zr phosphate			33.4	(106)
12	550		Fixed-bed	LHSV 0.1–5.0 h^–1^	ZnO			15.0	(106)
13	450		Adiabatic	LHSV 0.7 h^–1^	82% ZnO–18% ZrO_2_		77.2	17.9	(106)
14	370		Fixed-bed	LHSV 0.5 h^–1^	MgO–CaCO_3_–SiO_2_ (27.5:70.5:2.0)		71.7	7.1	(106)
15	100	2.7	Fixed-bed		γ-Alumina		78.2	13.0	(106)
16	255		Fixed-bed		Al_2_O_3_–MoO_3_		18.4	9.5	(106)
17	300		Fixed-bed	LHSV 0.3 h^–1^	ZnO–Cr_2_O_3_ with Fe_2_O_3_		87.7	14.2	(106)
18	280		Fixed-bed		ZnO–Cr_2_O_3_–CaO–Fe_2_O_3_		80.9	9.8	(106)
19	120		Batch	1 h	ZrO_2_			28.1	(106)
20	200		Batch	2 h	ZrO_2_–Cr_2_O_3_			15.5	(106)
21	120	20	Fixed-bed		Zr phosphate + ZnCl_2_		98	19.9	(106)
22	500		Fixed-bed	1000 h^–1^	Pd on MgO			38.5	(106)
23	350–450		Fixed-bed		MgO (94%)–V_2_O_5_ (6%)			11.4	(106)
24	100	3	Batch	0.5 h	KOH			11.8	(106)
25	115	5	Batch	4 h	Group IV metal phosphates			99.0	(106)
26	115	5	Fixed-bed	LHSV 4.0 h^–1^	Titanium phosphate		98.3	10.0	(106)
27	120	4.3	Batch	1 h	Ti, Zr, Hf, Sn phosphate		95.3	28.1	(106)
28	250	100	Batch	12 h	NH_4_Cl, NH_4_Br		38.8	8.3	(106)
29	300	1	Flow Reactor	1.2 h^–1^	Al_2_O_3_	59	20	12	(113)
30					Mg_0.7_AlO	72	17	12	(113)
31					Mg_1.0_AlO	78	7	5	(113)
32					Mg_3.5_Al0	68	10	5	(113)
33					Mg_6.5_AlO	58	12	7	(113)
34					MgO	39	71	28	(113)
35	300	1	Flow Reactor	1.19 h^–1^	MgO	16.9	67		(114)
36					Ca/MgO	17.9	66		(114)
37					Sr/MgO	16.2	68		(114)
38					Ba/MgO	17.4	67		(114)
39					Li/MgO	14.2	49		(114)
40					Na/MgO	14.8	70		(114)
41					K/MgO	13.5	71		(114)
42					Cs/MgO	12.6	71		(114)
43	210		Flow Reactor		Mo_2_N	25	49		(111)
44					USY zeolite	25	60		
45	150		Batch		Sulfated zirconia	34 (1 h)	100		(115)
						68 (3 h)	82		
						83 (9 h)	49		

**Table 10:**
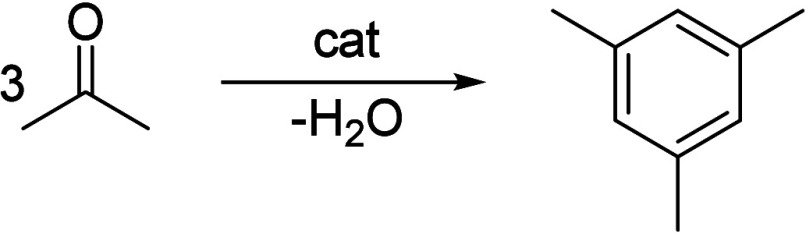
Condensation of Acetone to Mesitylene

Entry	*T* ( °C)	*P* (bar)	Reactor	Time	Catalyst	Conv (%)	Sel (%)	Yield (%)	Ref
1	175–80		Batch		HCl			36.0	(106)
2	600		Batch		HCl		31.0	39.0	(106)
3	20		Batch	20 h	Conc. H_2_SO_4_			9.2	(106)
4	–10 to 15		Batch		H_2_SO_4_ + 10% H_3_PO_4_			17.5	(106)
5	200		Fixed-bed	LHSV 0.2 h^–1^	Aluminosilicate		100.0	30.0	(106)
6	253	28	Fixed-bed	LHSV 0.99 h^–1^	Al_2_O_3_ + MoO_3_		74.0	38.0	(106)
7	204–426	0–67	Fixed-bed	LHSV 0.1–5.0 h^–1^	Cr_2_O_3_–B_2_O_3_–Al_2_O_3_			24.2	(106)
8	200–500		Fixed-bed		Nb_2_O_5_			35–45	(106)
9	450		Fixed-bed		Mg-Zr on graphite	49	19		(116)
						54	26		
						52	32.5		
10	200		Fixed-bed	1.45 h^–1^	Tantalum phosphate	91	87.8		(117)
11	130	25	Batch	6 h	Purolite CT275DR			12.5	(109)
	160							16	
	190							16.1	

**Table 11:**
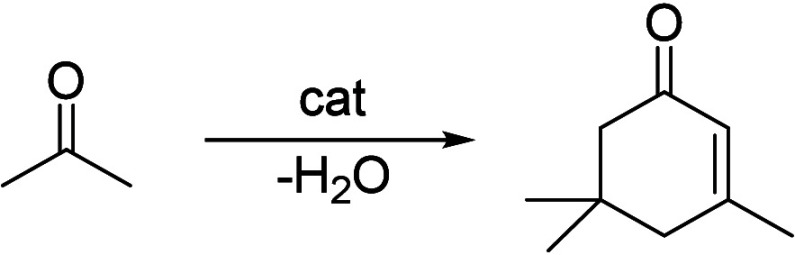
Condensation of Acetone to Isophorone

Entry	*T* (°C)	*P* (bar)	Reactor	Time	Catalyst	Conv (%)	Sel (%)	Yield (%)	Ref
1	120–130	30	Batch		Dilute alkali			42.0	(106)
2	150–200		Batch	37 min	Dilute alkali hydroxide (15–35%)			78.0	(106)
3	215		Batch	0.5 h	45% aqueous alkali solution		43.9	17.4	(106)
4	200	30	Batch	1.0 h	0.2–0.25% NaOH + Water:Acetone (30:40)			36.0	(106)
5	110	1.6	Batch	1.5 h	Alkali and dimethyl sulphoxide			22.1	(106)
6	424		Fixed-bed	LHSV 0.36 h^–1^	Alkali or alkaline earth metal salts		42.8	28.6	(106)
7	150		Fixed-bed		BaO, feed = mesityl oxide			10.0	(106)
8	300–520		Fixed-bed	LHSV 1.0 h^–1^	γ-Alumina		35.2	19.3	(106)
9	440–560		Fixed-bed	LHSV 1.0 h^–1^	Magnesia		35.0	13.7	(106)
10	300–540		Fixed-bed	LHSV 2.0 h^–1^	Chromia–alumina		64.5	21.8	(106)
11	300	1	Flow Reactor	1.2 h^–1^	Al_2_O_3_	59	64	37	(113)
12					Mg_0.7_AlO	72	71	51	(113)
13					Mg_1.0_AlO	78	78	61	(113)
14					Mg_3.5_Al0	68	80	54	(113)
15					Mg_6.5_AlO	58	71	41	(113)
16					MgO	39	22	9	(113)
17					Li/Mg_1.0_AlO	73	72	53	
18					Na/Mg_1.0_AlO	83	77	64	
19					K/Mg_1.0_AlO	85	78	66	
20	210		Fixed-bed		MgO	25	61		(111)

**Table 12:**
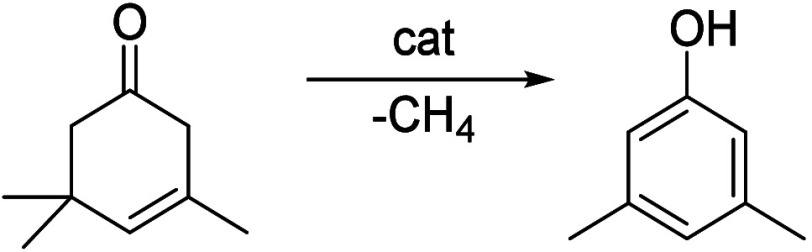
3,5-Xylenol from Isophorone

Entry	*T* ( °C)	Reactor	Time	Catalyst	Conv (%)	Sel (%)	Yield (%)	Ref
1	575	Fixed-bed	135 s	Cr-Ni alloy			77.6	(106)
2	600	Fixed-bed	LHSV 0.5 h^–1^	Fe-Ni-Cr alloy		83.1	79.6	(106)
3	400–700	Fixed-bed		Al_2_O_3_			51.0	(106)
				Al_2_O_3_ + 2.1% CaO			74.0	
				Al_2_O_3_ + 5% MgO			78.0	
4	500	Fixed-bed		Al_2_O_3_ + Fe_2_O_3_			33.6	(106)
5	450–600	Fixed-bed		Al_2_O_3_ + Fe_2_O_3_			50.0	(106)
6	625	Fixed-bed	86 v/v h^–1^	Cu_2_O–Cr_2_O_3_–BaO–graphite (45:36:2.5:11)		67.8	63.7	(106)
7	625	Fixed-bed	86 v/v h^–1^	Cu_2_O–Cr_2_O_3_ (58:38)		72.6	66.0	(106)
8	630	Fixed-bed	77 v/v h^–1^	Al_2_O_3_–Cr_2_O_3_–K_2_O–CeO_2_ (76:18:4:2)		71.1	71.1	(106)
9	560	Fixed-bed	LHSV 0.5 h^–1^	γ-Al_2_O_3_		38.4	49.4	(106)
10	560	Fixed-bed	LHSV 0.5 h^–1^	Cr_2_O_3_–Al_2_O_3_		89.8	87.7	(106)
11	530	Fixed-bed		Lithium phosphate		80.7	75.4	(106)
12	200–600	Fixed-bed	172 v/v h^–1^	FeAl_7_(PO_4_)_8_		74.9	73.1	(106)
				CoCa(PO_4_)_5_		71.2	69.5	
				Co_2_Al_3_Fe(PO_4_)_5_		59.0	54.6	
13	550–570	Flow reactor	0.5 v/v h^–1^	CH_3_I		85.0	85.0	(106)
14	600	Flow reactor	0.5 v/v h^–1^	*n*-Butyl bromide		80.4	76.0	(106)
15	600	Flow reactor	0.33 v/v h^–1^	CCl_4_		65.0	62.4	(106)
16	570	Flow reactor	0.8 v/v h^–1^	Allyl bromide		81.0	60.7	(106)
17	570	Flow reactor	0.3 v/v h^–1^	Phenyl iodide		86.0	85.0	(106)
18	400	Fixed-bed	3 h^–1^	10% Cr_2_O_3_/SiO_2_			19.1	(118)
19	440						24.9	(118)
20	480						32.1	(118)
21	520						34.6	(118)
22	500	Fixed-bed	1 h^–1^	15% Cr_2_O_3_/Al_2_O_3_	84		43.2	(119)
23				2% K, 15% Cr_2_O_3_/Al_2_O_3_	76		48.9	(119)
24	440	Fixed-bed	WHSV 1 h^–1^	Alumina	74		14.4	(120)
25				Carbon-covered alumina	32		16.9	(120)
26	520	Fixed-bed	WHSV 1 h^–1^	Alumina	86		49.3	(120)
27				Carbon-covered alumina	92		66.4	(120)
28	450	Fixed-bed		Mg-Zr on graphite	49	2.5		(116)
29					52	20.7		
30					51	17.0		
31	527	Flow reactor	1.0 h^–1^	Cr_2_O_3_–K_2_O/carbon-covered alumina	59	98		(121)
32	544			Cr_2_O_3_-K_2_O/Al_2_O_3_	76	78		
33	546			Carbon-covered alumina	87	60		
34	547			Al_2_O_3_	92	65		
35	450–580		0.5–4.0 h^–1^	Cr_2_O_3_-Al_2_O_3_		90		(122)

Mesityl oxide can be obtained from the aldol condensation/dehydration of acetone. Both batch and continuous approaches have been described, using dehydrating reagents or preferably oxides. For the latter class of substances, there were various modifications of the catalysts. MgO and alkali (Li, Na, K, Cs) or alkaline earth (Ca, Sr, Ba) magnesium oxides give significantly lower mesityl oxide yields as shown by Di Cosimo et al.^114^ Thus, only conversions between 12.6 and 17.9% with mesityl oxide as the main product (selectivity between 66 and 71%) could be found. Isophorone, on the other hand, was formed only in the case of using Li-doped magnesium oxide with a selectivity of 38%.^114^ Ma et al. confirmed this trend but described significant efficiency improvements with systematically different compositions of Mg-Al oxides as well as the binary archetypal phases MgO and Al_2_O_3_ from a citrate method (Tables 9 and 11). They found that by adding an alkali promoter, the efficiency could be further increased. For example, K/Mg_1.0_AlO provides an isophorone yield of 66% at 300 °C in a flow reactor at a GHSV of 1.2 mL h^–1^ (Table 11).^113^

Particularly noteworthy is the selective production of mesitylene directly from acetone. Without a doubt, this conversion is a focal point in the aromatics production from these sources. Suitable catalysts for the production of mesitylene from acetone are solids bearing acidic (and basic) groups on their surface. Of these candidates, tantalum phosphate stands out.^117^ The material is obviously equipped with abundant strongly acidic sites and was shown to be extremely active even at a relatively low temperature of 200 °C, so that an acetone conversion of 91.0% was observed with a mesitylene selectivity of 87.8%. In addition, a relatively small amount of coke forms on this catalyst compared to other catalysts, which can be removed by regeneration through calcination in oxygen, thus making the catalyst usable again.

The formation of isophorone and the derived production of 3,5-xylenol must be considered together. Isophorone as a precursor to 3,5-xylenol is available under a wide variety of experimental conditions (Table 11). Aromatization of isophorone can be realized at high temperatures using various catalysts (Table 12). Raju et al. found a significant 3,5-xylenol yield of 34.6% at a temperature of 520 °C when isophorone was converted over 10% Cr_2_O_3_/SiO_2_ at 3 h^–1^. In the same run, there was also a remarkable formation of cresols and 2,3,5-trimethylphenol but in the lower percentage range.^118^ By changing the support to Al_2_O_3_ with a K promoter, the yield to 3,5-xylenol was increased to 48.9%.^119^ Interestingly, surface coating of commercial alumina with carbon further increased the selectivity to 3,5-xylenol and yields of up to 66.4% were obtained. This improvement was explained by suppressed decomposition reactions as a result of blocking the acid sites.^120^ That both blocking of the acid sites and Cr as a catalyst could play a role in the 3,5-xylenol synthesis from isophorone was attempted to be shown in the combination of both strategies. However, no unambiguously clear trend was found with respect to the different catalyst components.^121^

In many of the aforementioned examples, the outcome can be a mixture of many condensation products with diacetone alcohol, mesityl oxide, phorone, mesitylene, isophorone as main components.^106^ Such products can also be seen as valuable intermediates, as the aromatization of isophorone itself to products like 3,5-xylenol, 2,3,5 trimethylphenol, *m*-cresol, *m*-xylene, mesitylene, toluene, or even jet fuel aromatics is possible.^122−124^ The range of possible products can also be influenced by admixture of hydrogen or hydrogenating co-substrates.^125^ In this regard, an interesting approach is the *in situ* conversion of such intermediates obtained from acetone condensation possible by a tandem reaction in a one-pot process with CaC_2_ as reaction partner which leads to methyl-substituted aromatics. In the best case, 48% methyl-substituted naphthalenes and 20% 3,5-xylenol are produced in addition to mesitylene.^126^

There are other reactions possible for acetone aromatization. H-ZSM-5 catalysts can be used to convert pure acetone to benzene, toluene, ethylbenzene and xylenes (BTEX).^127^ At a temperature of 400 °C at almost complete acetone conversion, a liquid main fraction is formed which, in addition to saturated hydrocarbons, mainly contains aromatics, mostly BTEX. In addition, besides gaseous products, such as CO_*x*_ and C_1–4_ saturated and unsaturated hydrocarbons were formed. Shorter residence times are conducive to high BTEX yields, as increasing them promotes the formation of more C_9/10_ aromatics. The maximum aromatics selectivity (*S* ≈ 71%) was found at full conversion at a space velocity of 4 h^–1^. Interestingly, the BTEX fraction can be increased somewhat by the presence of methane instead of nitrogen. For example, on Zn-Ga- and Zn-Pd-functionalized H-ZSM-5, Austin et al. found a selectivity increase of acetone aromatization at 5 bar in batch from 48 to 54% in the presence of methane (liquid yield about 70%). At this point, it is important to mention that the reaction temperature of 400 °C is significantly lower than that for methane dehydroaromatisation (cf. chapter 8), so that a reaction involving both compounds must be assumed here. It was further demonstrated by NMR spectroscopy and isotopic labelling that methane was incorporated into the product.^128^ Wang et al. investigated the most important parameters influencing the conversion of acetone to BTX over 5% Ni_2_P/H-ZSM-5 catalyst in a continuous flow reactor. They found that temperature and contact time have a crucial influence on the product spectrum. Other important parameters are reaction pressure and H_2_ partial pressure. At 450° C and a contact time of 0.54 s, they found the highest BTX selectivity of 49% with almost complete acetone conversion in the presence of added hydrogen.^129^ In addition, longer-chain ketones can also be converted in the same way, although the examples of this are incomparably fewer.

Based on the reaction of hemicellulose or cellulose and the corresponding sugars xylose and glucose, respectively, various reaction routes can lead to higher ketones. Alternatively 2-butanone can be produced either by dehydration of 2,3-butanediol, which can be obtained via fermentation or via hydrogenolysis of sorbitol (Dacheng process)^130^ or from levulinic acid by decarboxylation.^131,132^ Via the alkylation of acetone using bio-based alcohols, 2-pentanone and 2-heptanone can be accessed.^133,134^ The former can also be produced from the ring-opening hydrogenolysis of 2-methylfuran over xylose. 2-Hexanone can produced from 2,5-dimethylfuran by hydrogenation.^135^

**Scheme 7:**
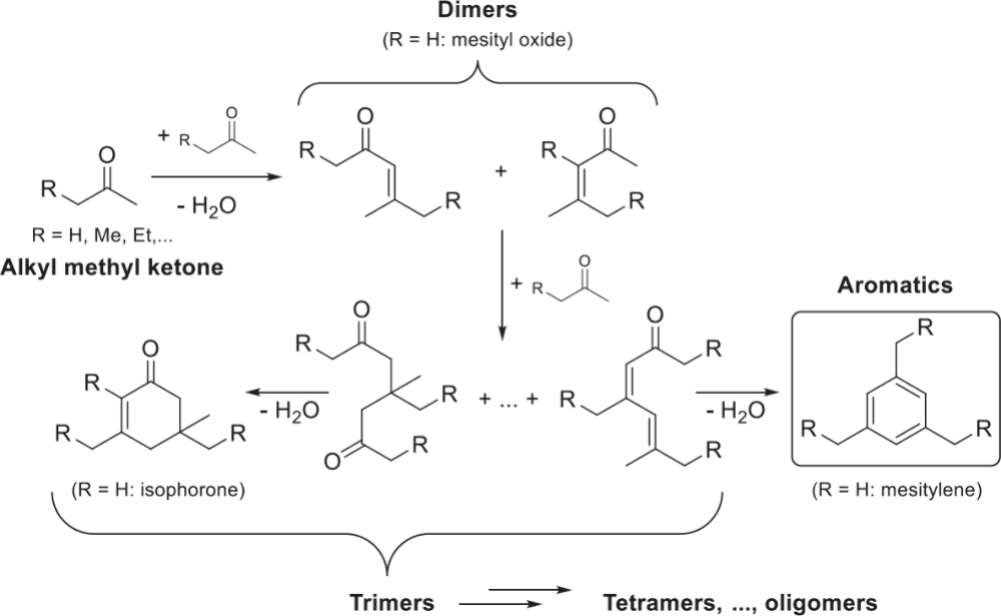
Proposed Reaction Scheme for the Acid-Catalyzed Condensation of Alkyl Methyl Ketones to Aromatics Reproduced with permission from ref (137). Copyright 2022 Elsevier.

Such higher ketones can also be converted to aromatics using ZSM-5-based catalysts^107^ whereby even the ketonisation of the corresponding acids of an upstream step via titanium dioxide have already been considered.^136^Scheme 7 shows the comparable conversion of various alkyl methyl ketones to such structures. Reif et al. were able to show that at least the possibility of such a conversion exists (aromatics yields of less than 1%).^137^ This is therefore only a perspective outlook at this point. However, Bell and co-workers showed that the conversion of higher C_4_-C_6_ ketones appears to be quite comparable to acetone and tracked the conversions and selectivities to dimers and trimers. Compared to the conversion of acetone (24%), only 2-hexanone showed lower conversions (11%) in favor of increased dimer selectivities (82%). For the C_3–5_-ones, the trimer selectivities were between 75 and 84%. Such production of unsaturated or even aromatic trimers is also discussed in the frame of renewable fuel generation.^135^

#### 2.3.6 Aromatics from Other Fermentation Products

##### 2.3.6.1 From Muconic Acid

**Figure 6:**
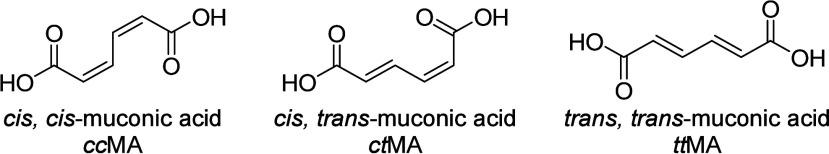
Structures of muconic acid isomers.

Muconic acid is an important bio-derived chemical which may exist in three isomeric forms (Figure 6), *cis,cis*-muconic acid (*cc*MA), *cis,trans*-muconic acid (*ct*MA), *trans,trans*-muconic acid (*tt*MA). Its importance is highlighted by its ready conversion into a variety of monomers, including adipic acid (nylon-6,6 monomer), ε-caprolactam (nylon-6 monomer), and terephthalic acid (PET monomer).^138,139^ The formation of *cc*MA by fermentation has been reported multiple times as has its chemical synthesis from lignin; this has been reviewed extensively.^138−141^ A few studies demonstrated its production from glucose by fermentation.^139−143^ The synthesis of *tt*MA has been achieved by deoxydehydration of sugar-based mucic acid, multi-step conversion of adipic acid, or isomerization of *cc*MA.^139,144^ Diels–Alder (DA) reactions of *tt*MA and olefins followed by dehydrogenation produces aromatic chemicals, while *cc*MA or *ct*MA have to isomerize to *tt*MA prior to DA reactions. None of these routes have been commercialized though.

**Scheme 8:**
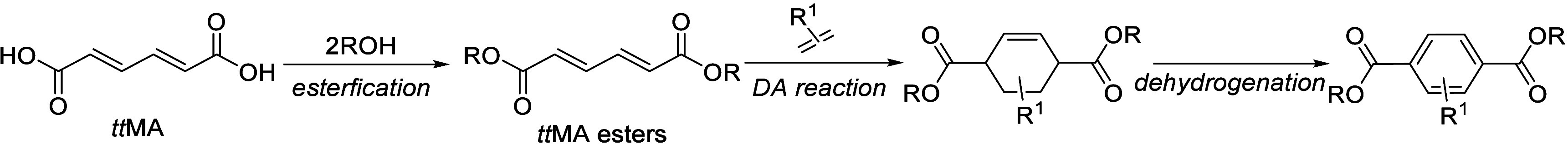
Dield–Alder Reactions of *tt*MA Esters and Olefins to Terephthalic Esters Followed by Dehydrogenation

*tt*MA contains two electron-withdrawing carboxylic acid groups and has been rarely used in a DA to deliver cyclized products. Instead, *tt*MA esters, readily obtained from *tt*MA and alcohols under acidic conditions and possessing better solubility in most organic solvents, are often used in DA reactions to form cyclic compounds, which can be further dehydrogenated to terephthalic esters (Scheme 8).

**Scheme 9:**
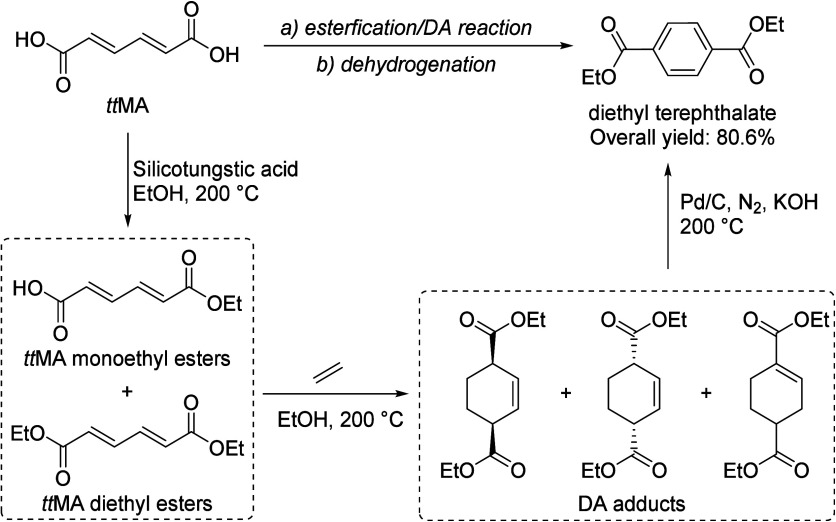
Cascade Process for Diethyl Terephthalate Production from *tt*MA^145^

In 2016, Xu and Lu first described this esterification/DA/dehydrogenation strategy to produce diethyl terephthalate from *tt*MA in 80.6% overall yield (Scheme 9), wherein esterification and DA reaction were integrated into one step. Using 1 mol% silicotungstic acid as catalyst, *tt*MA in ethanol was converted to diethyl muconate in 92% yield under 1 bar ethylene at 200 °C after 4 h. Increased ethylene pressure enhanced the formation of the DA adducts which reached >99% yield under 30 bar ethylene. Under inert atmosphere (N_2_), the resulting oily products were dehydrogenated to diethyl terephthalate in 80.6% yield at 200 °C catalyzed by Pd/C with addition of KOH to neutralize the silicotungstic acid which remained from the first step.^145^

**Scheme 10:**
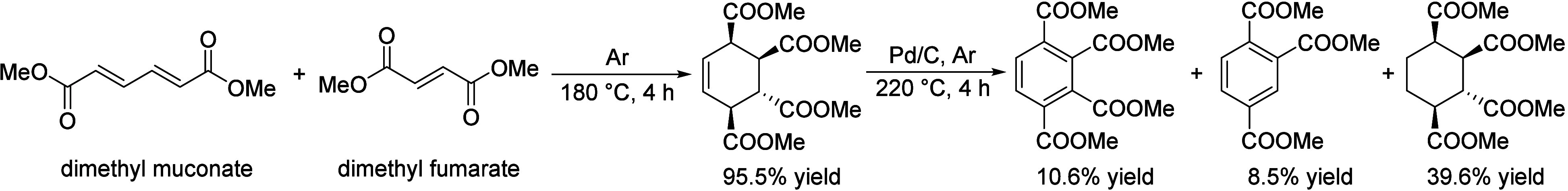
Reaction of Dimethyl Muconate and Dimethyl Fumarate to Bio-Based Aromatics^146^

Xu and Lu extended this strategy to other bio-based dienophiles, including dimethyl fumarate,^146^ methyl acrylate,^147^ and *p*-benzoquinone.^148^ Dimethyl muconate reacted with dimethyl fumarate under Ar at 180 °C to give 95.5% yield of the DA adduct, which was converted to tetra- or tri-methoxycarbonyl-benzene in 10.6% and 8.5% yield, along with 39.6% hydrogenated product (Scheme 10).^146^

**Scheme 11:**
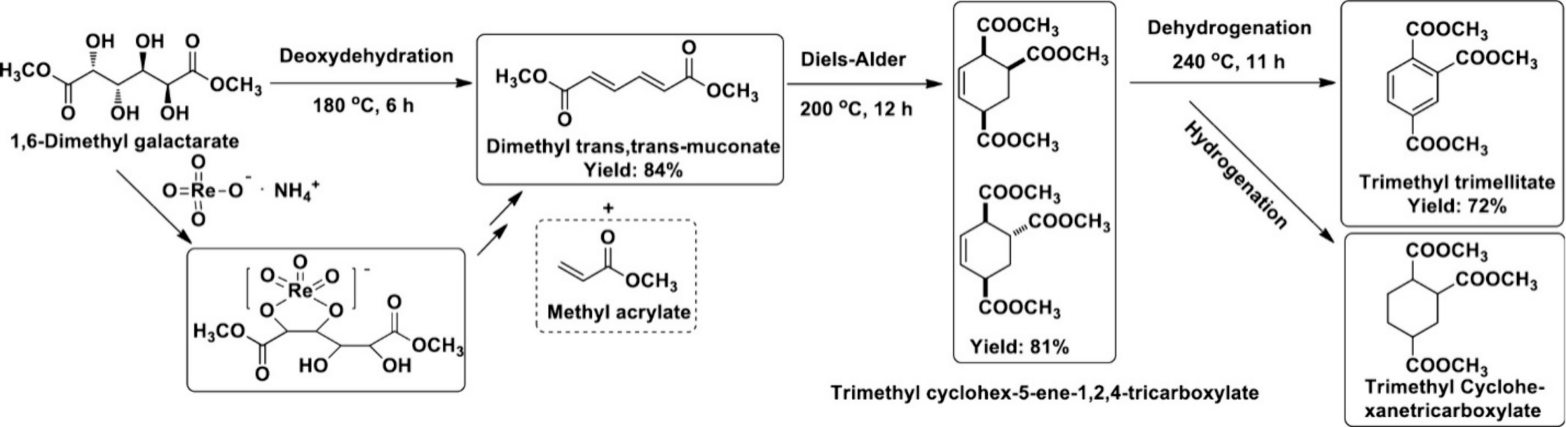
Formation of Trimethyl Trimellitate from 1,6-Dimethyl Galactarate Reproduced with permission from ref (147). Copyright 2021 American Chemical Society.

The same authors reported the *de novo* synthesis of trimethyl trimellitate from dimethyl galactarate (Scheme 11).^147^ NH_4_Re-catalyzed deoxydehydration of bio-based 1,6-dimethyl galactarate at 180 °C using PPh_3_ as reductant produced dimethyl muconate in 84% yield. This was reacted with methyl acrylate at 200 °C to give trimethyl cyclohex-5-ene-1,2,4-tricarboxylate in 81% yield.^147^ Methyl acrylate can be obtained from renewable resources by dehydration of lactic acid^149,150^ or dehydration and oxidation of glycerol.^150,151^ Pd/C-catalyzed dehydrogenation of trimethyl cyclohex-5-ene-1,2,4-tricarboxylate at 240 °C resulted in the formation of trimethyl trimellitate in 72% yield.^147^ Prolonged reaction times decreased the yield of trimethyl trimellitate due to the formation of hydrogenated product – trimethyl cyclohexanetricarboxylate (Scheme 11).^147^

**Scheme 12:**
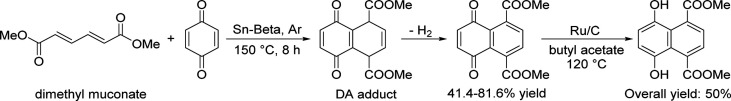
Reaction of Dimethyl Muconate with *p*-Benzoquinone to Aromatics^148^

*p*-Benzoquinone served as a dienophile in the DA reaction with dimethyl muconate catalyzed by Sn-Beta zeolite at 150 °C (Scheme 12).^148^ The use of 1.0 equiv of *p*-benzoquinone produced the DA adduct as main product in 44.2% yield. When the amount of *p*-benzoquinone was increased to 2.0, 3.0, or 4.0 equiv, the yield of dehydrogenated product was improved to 41.4%, 76.5%, and 81.6%, respectively. The excess amount of *p*-benzoquinone facilitated the removal of hydrogen from the DA adduct, and hence raised the aromatic yields, meanwhile *p*-benzoquinone was hydrogenated to hydroquinone. Further dehydrogenation with Ru/C at 120 °C produced the 1,4-naphthohydroquinone derivative in 50% yield (Scheme 12).^148^

**Scheme 13:**
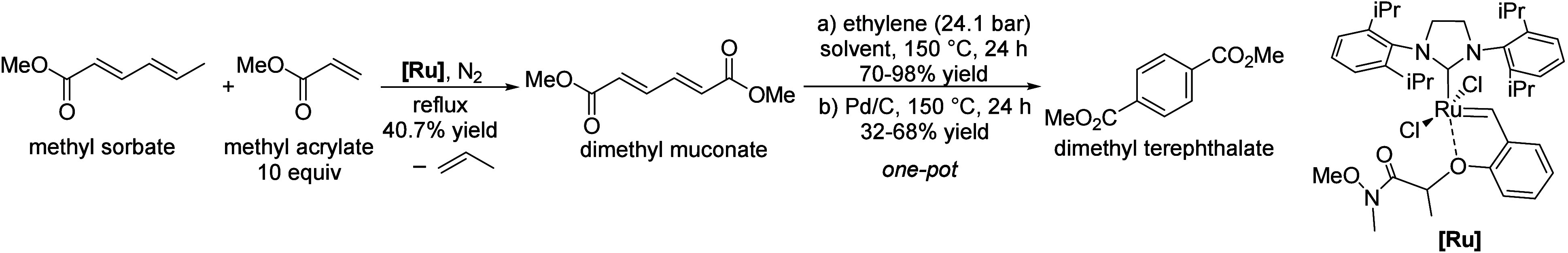
Metathesis of Methyl Sorbate and Methyl Acrylate to Dimethyl Muconate and Its Conversion to Dimethyl Terephthalate^152^

Lobo and co-workers reported a metathesis pathway to synthesize dimethyl muconate in 40.7% yield from methyl sorbate and methyl acrylate catalyzed by a Ru-NHC complex ([Ru]).^152^ The product was subjected to a one-pot DA/dehydrogenation reaction at 150 °C, and produced dimethyl terephthalate in 32–68% yield, depending on the solvent used (Scheme 13).^152^

##### 2.2.6.2 From Sorbic Acid

Sorbic acid (*trans,trans*-2,4-hexadienoic acid) can be isolated by extraction from non-edible mountain ash berries (*Sorbus Aucuparia*).^153^ It can be also prepared from bio-based 4-hydroxy-6-methyl-2-pyrone, a fermentative product from glucose.^153,154^ Sorbic acid is extensively used for food preservation;^153^ preparing bio-based aromatics from it will probably be too expensive and its use for this purpose has only been reported to a limited extent.

**Scheme 14:**
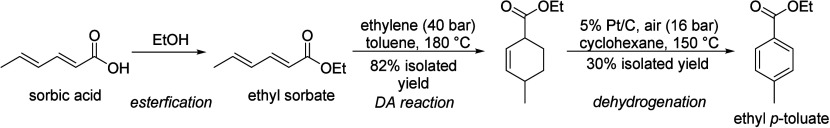
Conversion of Sorbic Acid to Ethyl *p*-Toluate through Esterification/DA Reaction/Dehydrogenation^155^

Delcroix synthesized ethyl *p*-toluate from sorbic acid through esterification, DA reaction, followed by dehydrogenation (Scheme 14).^155^ Ethyl sorbate, obtained from sorbic acid and ethanol, reacted with ethylene (40 bar) through DA reaction in toluene at 180 °C to produce 4-methylcyclohex-2-enoate in 82% isolated yield. Pt/C-catalyzed dehydrogenation of 4-methylcyclohex-3-enoate at 150 °C led to the formation of ethyl *p*-toluate in 30% isolated yield.^155^

**Scheme 15:**
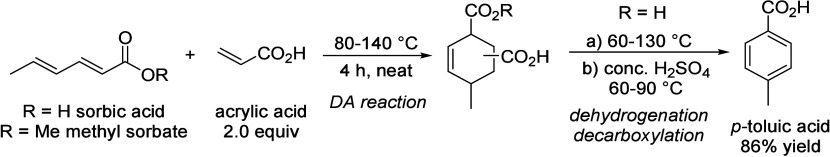
Production of *p*-Toluic Acid from Sorbic Acid and Acrylic Acid^157^

In 1950, Alder described the DA reaction of sorbic acid with acrylic acid, which afforded the two isomers of the DA adduct, which were further dehydrogenated by sulfur to the aromatic products.^156^ The yields were not mentioned.^156^ In 2016, Gioia re-investigated acrylic acid as dienophile for the DA reaction of sorbic acid and sorbate (Scheme 15).^157^ Sorbic acid or methyl sorbate reacting with acrylic acid under neat conditions produced a mixture of cyclic adduct isomers in 79% conversion at 80 °C and 100% conversion at 140 °C. The obtained isomer mixture was directly converted to *p*-toluic acid in 86% yield through a one-pot dehydrogenation/ decarboxylation.^157^

##### 2.3.6.3 From Succinic Acid

Succinic acid is one of the top 10 platform chemicals originating from carbohydrates listed by the U.S. Department of Energy (DOE) in their report.^158,159^ It can be converted into a number of valuable products, such as maleic anhydride (MA), 1,4-butanediol, tetrahydrofuran, etc.^6,159,160^ Bio-based succinic acid can be produced by fermentation, but also via oxidation of furfural or levulinic acid or via reduction of tartaric acid.^161,162^ At some point no less than four companies were producing succinic acid via fermentation: Succinity, Myriant, BioAmber, and Reverdia;^6,161^ however, the market for succinic acid is rather small and is growing only marginally each year. For this reason, three of these companies have stopped production and only Roquette, one of the partners in Reverdia is still producing succinic acid via fermentation.

**Scheme 16:**
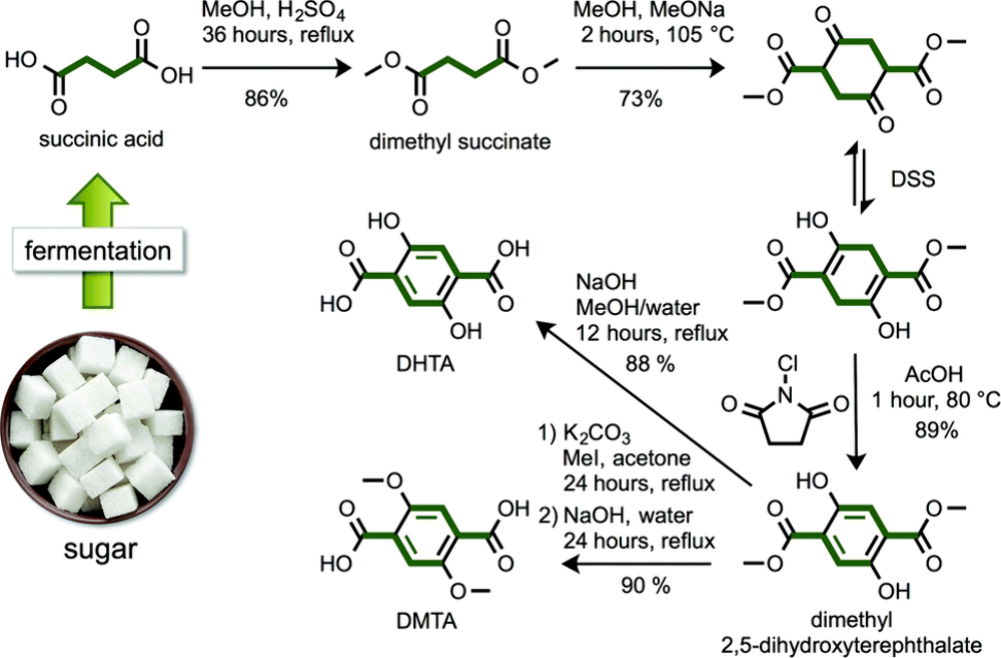
Preparation of Aromatic Monomers from Succinic Acid Reproduced with permission from ref (163). Copyright 2018 The Royal Society of Chemistry.

Miller reported a synthesis of aromatic monomers from succinic acid by a four-step approach (Scheme 16).^163^ Esterification of succinic acid with methanol in the presence of H_2_SO_4_ led to the formation of dimethyl succinate in 86% yield, which was cyclodimerized in methanol using 1 equiv of MeONa at 105 °C to produce dimethyl succinyl succinate (DSS) in 73% yield. Dehydrogenation of DSS with *N*-chlorosuccinimide in AcOH resulted in the production of dimethyl 2,5-dihydroxyterephthalate in 89% yield. Dimethyl 2,5-dihydroxyterephthalate was further converted to aromatic monomers, depending on the conditions to 2,5-dihydroxyterephthalic acid (DHTA) in 88% yield or 2,5-dimethoxyterephthalic acid (DMTA) in 90% yield.^163^

##### 2.3.6.4 From Quinic Acid/Shikimic Acid

**Scheme 17:**
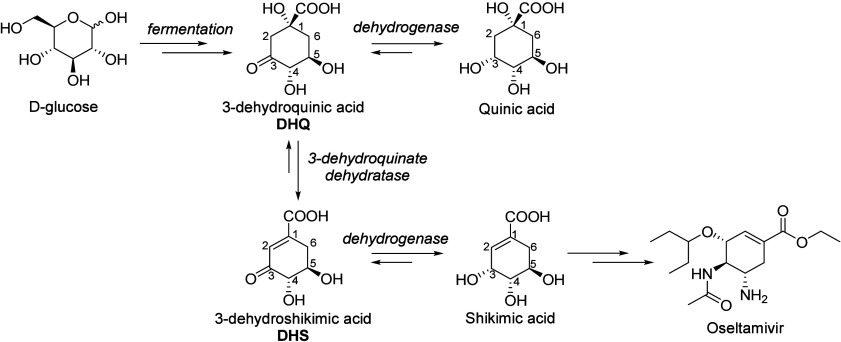
Simplified Shikimate Pathway to Quinic Acid, Shikimic Acid, DHQ, and DHS

The shikimate pathway is a major pathway in microorganisms and plants for the synthesis of aromatic amino acids from glucose, named after its main precursor, shikimic acid. This pathway involves several cyclohexenecarboxylic acid intermediates containing 2–4 hydroxy groups (Scheme 17), including quinic acid, 3-dehydroquinic acid (DHQ), shikimic acid, 3-dehydroshikimic acid (DHS).^164−167^ The biosynthesis of aromatic chemicals via this pathway aided by metabolic engineering has been reviewed.^4,168−170^ Herein, we will focus on the chemical conversions of these intermediates that can be obtained from fermentation. Metabolic engineering has been used to partially block the pathway to synthesize these chemicals, especially shikimic acid, which is largely used for the production of the anti-influeza medicine oseltamivir (Tamiflu).^164−166^ In addition, the chemical syntheses of shikimic acid from sugars has been realized through various methods.^164^ Quinic acid can be obtained from the bark of the Cinchona tree.

Due to the presence of carboxylic acid and hydroxy groups in these structures, the chemical syntheses of aromatics from DHQ, DHS, quinic acid, and shikimic acid mainly involve the following three pathways: (i) decarboxylation and dehydration to phenols; (ii) dehydration to benzoic acid or esters; and (iii) condensation with amines and dehydration to anilines, especially for DHQ and DHS.

**Scheme 18:**
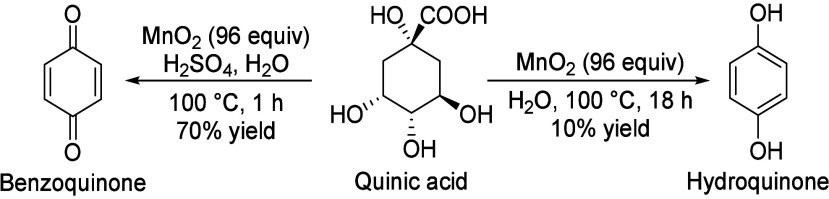
Conversion of Quinic Acid to Benzoquinone and Hydroquinone by Reaction with MnO_2_^171^

**Scheme 19:**
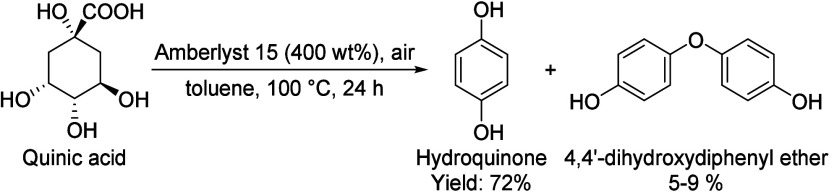
Synthesis of Hydroquinone from Quinic Acid in the Presence of Amberlyst-15^173^

Decarboxylation of quinic acid followed by the removal of two hydroxyl groups at C3 and C5 through dehydration results in the formation of hydroquinone. In 1992, Frost first observed 10% hydroquinone from the reaction of an aqueous solution of quinic acid with MnO_2_ at 100 °C for 18 h, whereas 70% of benzoquinone was obtained under the same conditions in the presence of H_2_SO_4_ (Scheme 18).^171^ In 2001, Frost tested various oxidants for the one-pot conversion of quinic acid to hydroquinone in water (Table 13), involving oxidative decarboxylation and dehydration.^172^ Use of stoichiometric amounts of NaOCl, (NH_4_)_2_Ce(SO_4_)_3_, or V_2_O_5_ as oxidants led to the formation of trihydroxycyclohexanone, which was further dehydrated by heating under reflux to afford hydroquinone in 87%, 91%, and 85% yields, respectively.^172^ Ag_3_PO_4_-catalyzed oxidation of quinic acid with 1.2 equiv of K_2_S_2_O_8_ as oxidant produced hydroquinone in 51–85% yields followed by dehydration.^172^ Recently, Candeias discovered an air-oxidized conversion of quinic acid to hydroquinone in the presence of 400 wt% of dry Amberlyst 15 using toluene as solvent, which afforded hydroquinone in 72% yield along with 5–9% yields of 4,4′-dihydroxydiphenyl ether (Scheme 19).^173^

**Table 13:**
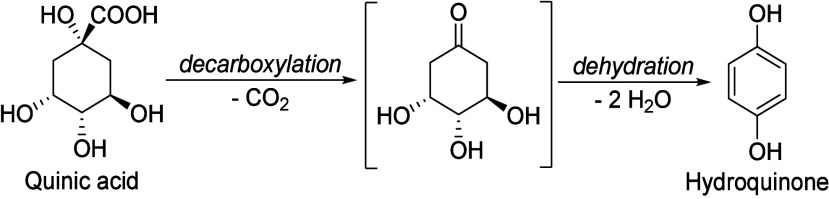
One-Pot Conversion of Quinic Acid to Hydroquinone with Various Oxidants^172^

	Conditions		
Oxidant/catalyst (equiv)	Oxidative decarboxylation	Dehydration	Yield (%)
NaOCl (4.6)	i) H_2_SO_4_ (1.2), RT, 3 h	reflux, Ar, 10 h	87
	ii) 2-propanol^a^		
(NH_4_)_2_Ce(SO_4_)_3_ (2.4)	RT, 30 min	reflux, Ar, 10 h	91
V_2_O_5_ (1.1)	50 °C, 4 h	reflux, Ar, 8 h	85
K_2_S_2_O_8_ (1.2)/Ag_3_PO_4_ (0.10)	50 °C, 4 h	reflux, Ar, 8 h	85
K_2_S_2_O_8_ (1.2)/Ag_3_PO_4_ (0.02)	50 °C, 4 h	reflux, Ar, 8 h	74
K_2_S_2_O_8_ (1.2)/Ag_3_PO_4_ (0.01)	50 °C, 4 h	reflux, Ar, 8 h	51

a Isopropanol was added to quench excess NaOCl.

**Scheme 20:**
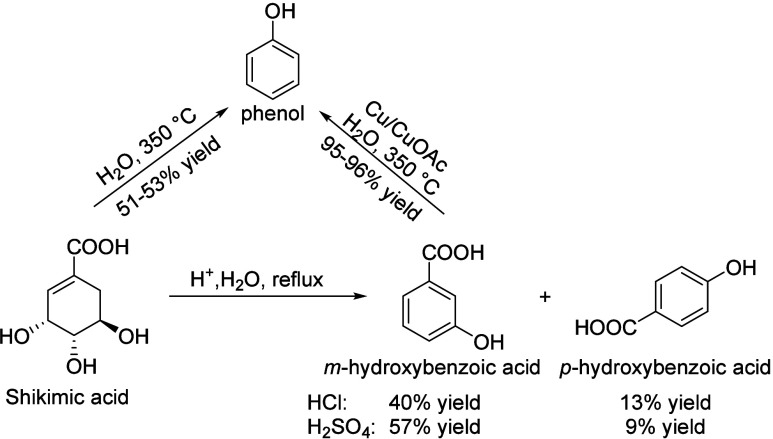
Conversion of Shikimic Acid to *m*-Hydroxybenzoic Acid and Phenol^174^

Dehydration of shikimic acid with acids (HCl or H_2_SO_4_), produced 40% and 57% yield of *m*-hydroxybenzoic acid along with 13% and 9% yield of *p*-hydroxybenzoic acid, respectively.^174^ Decarboxylation of *m*-hydroxybenzoic acid at 350 °C with 1.0 equiv of Cu or CuOAc yielded 95–96% of phenol. The direct synthesis of phenol from shikimic acid was achieved in water at 350 °C in 51–53% yield (Scheme 20).^174^

**Scheme 21:**
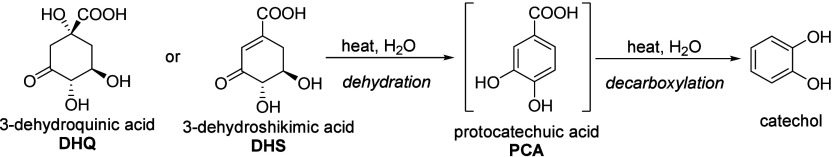
Production of PCA and Catechol from DHQ and DHS

DHQ and DHS, containing one keto group at C3, were converted to catechol by dehydration at C5, C6 followed by decarboxylation (Scheme 21).^175^ Frost reported the transformation of DHQ and DHS in aqueous solution to protocatechuic acid (PCA) at 190 °C in 68% and 82% yield respectively with 4% and 3% catechol as side product.^175^ Elevated temperatures increased catechol formation due to the decarboxylation of PCA to catechol at high temperature. At 250–310 °C, catechol was produced from the aqueous solution of DHQ and DHS in 40–54% and 86–90% yields, respectively.^175^

**Scheme 22:**
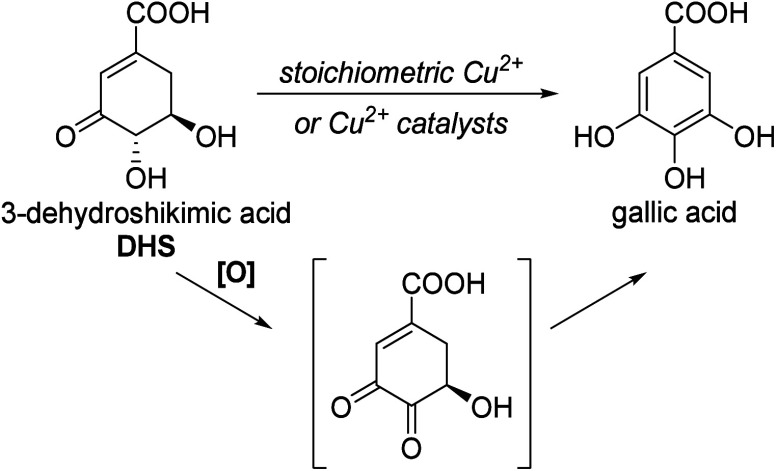
Dehydrogenation of DHS to Gallic Acid with Cu^2+^ ^176^

Dehydrogenation of DHS and shikimic acid, leaving the hydroxy and carboxylic acid groups untouched, leads to the formation of gallic acid, which is used as building block for pharmaceuticals and as anti-oxidant. In 2000, Frost and co-workers reported the conversion of DHS to gallic acid in the presence of Cu^2+^ (Scheme 22).^176^ In aqueous phosphate buffer solution, stoichiometric amounts of CuCO_3_Cu(OH)_2_ or Cu_*x*_(H_3–*x*_PO_4_)_2_ (prepared by dissolving CuSO_4_ in H_2_O followed by addition of NaHPO_4_ resulting in a blue-colored precipitate) oxidized DHS to gallic acid in in 31–51% yields. When AcOH was used as solvent, gallic acid was obtained in 74% yield at 40 °C in the presence of 2.2 equiv of Cu(OAc)_2_. The use of Cu(OAc)_2_ as catalyst in AcOH provided 21–48% yields of gallic acid at 40 °C with NH_4_NO_3_, H_2_O_2_, or air as oxidants. The addition of 0.1–0.5 equiv of Zn to the Cu(OAc)_2_-catalyzed oxidation of DHS in AcOH at 50 °C improved the yields of gallic acid to 46–72% under air or oxygen, while reaction in the absence of Cu(OAc)_2_ gave the dehydrated product, PCA.^176^

**Scheme 23:**
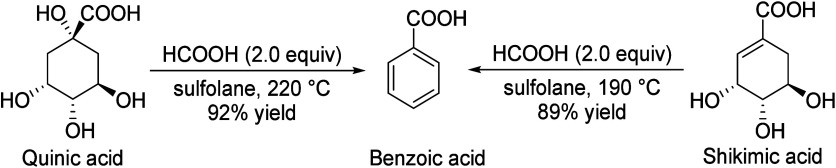
HCOOH-Mediated Deoxydehydration of Quinic Acid and Shikimic Acid to Benzoic Acid^177^

A deoxydehydration (DODH) reaction at C3 and C4 followed by dehydration on quinic acid or shikimic acid forms benzoic acid, which was achieved by using 2.0 equiv of HCOOH in sulfolane, affording benzoic acid in 92% and 89% yields respectively from quinic acid and shikimic acid (Scheme 23).^177^

**Scheme 24:**
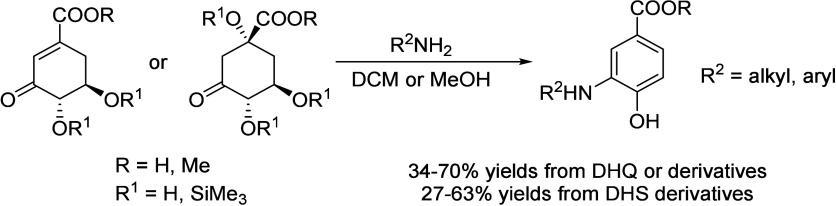
Transformation of DHQ and DHS Derivatives to Arylamines^178^

DHS and DHQ both contain one carbonyl group at C3, allowing its reaction to amines through condensation followed by dehydration to form arylamines. In 1993, Gorrichon first observed the formation of 3-aminobenzoates and 3-amino-benzoic acids in 34–70% yields from the reaction of primary amines to DHQ or its derivatives using dichloromethane (DCM) or MeOH as solvent (Scheme 24).^178^ The reaction of primary amines with DHS derivatives also produced the corresponding arylamines in 27–63% yields.^178^

**Scheme 25:**
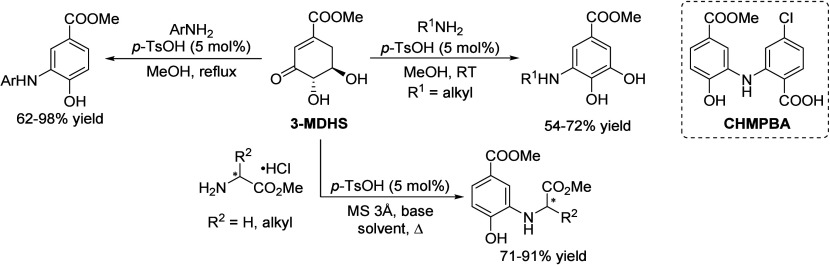
Reaction of 3-MDHS with Arylamines or Alkylamines and the Structure of CHMPBA^179−181^

**Scheme 26:**
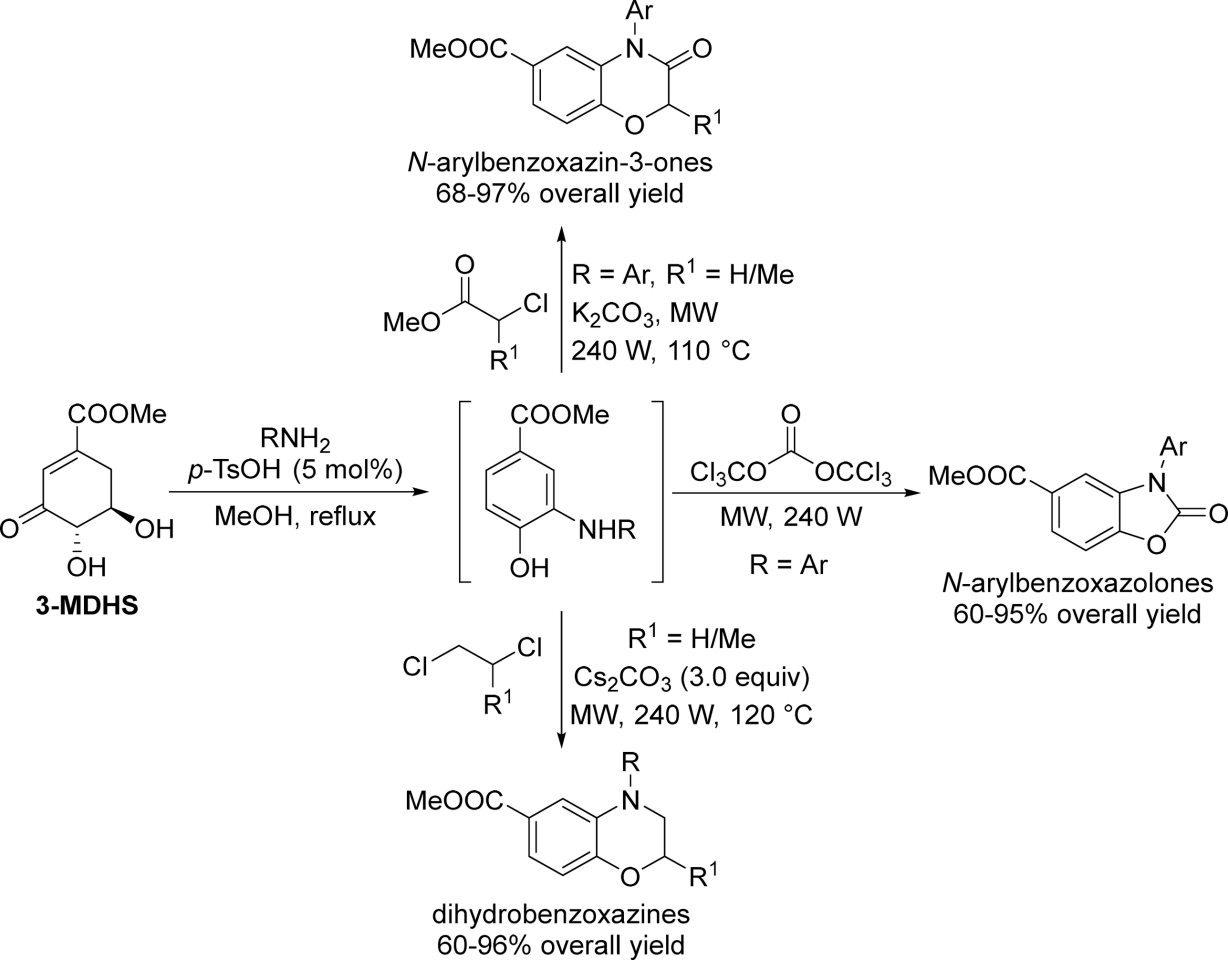
Conversion of 3-MDHS to Heterocyclic Products^182−184^

Zou’s group revisited this topic by focusing on methyl 3-dehydroshikimate (3-MDHS). Catalyzed by 5 mol% *p*-TsOH, 3-MDHS reacted with arylamines in MeOH and then dehydrated to methyl 3-arylamino-4-hydroxybenzoate in 62–98% yields at reflux (Scheme 25).^179^ When alkylamine reacted with 3-MDHS at room temperature, methyl 3-alkylamino-4,5-hydroxybenzoates, rather than methyl 3-alkylamino-4-hydroxybenzoate, were obtained in 54–72% yields due to the dehydrogenation reaction under air.^179^ This hypothesis was verified by the reaction of 3-MDHS and arylamine oxidized by Cu(OAc)_2_, instead of *p*-TsOH, from which methyl 3-phenylamino-4,5-hydroxybenzoates were produced.^179^ This synthetic approach was used to prepare a bio-based fluorescent sensor, CHMPBA, in 83% yield, which finds use as ammonia vapor detector, fluorescent invisible ink, or anti-false trademark ink (Scheme 25).^180^ Amino acid esters reacted with 3-MDHS catalyzed by *p*-TsOH in the presence of 3Å MS to give the corresponding substituted methyl 3-amino-4-hydroxybenzoates in 71–91% yields (Scheme 25).^181^ In addition, the group utilized the same strategy to convert 3-MDHS to *N*-arylbenzoxazolones,^182^*N*-arylbenzoxazin-3-ones,^183^ and *N*-substituted dihydrobenzoxazines via methyl 3-amino-4-hydroxybenzoates as intermediates (Scheme 26).^184^

**Scheme 27:**
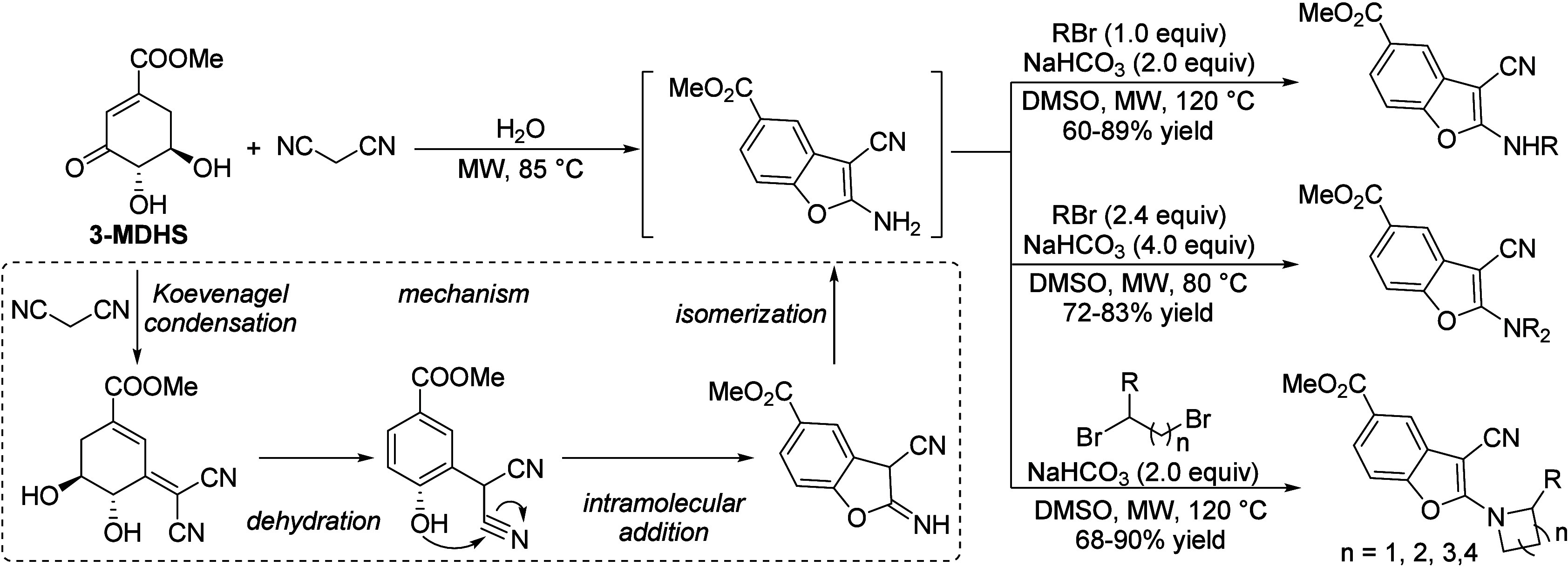
Conversion of 3-MDHS to Benzofuran and Derivatives^185^

Reaction of 3-MDHS with malononitrile in water using microwave irradiation led to the formation of a benzofuran ring via Knoevenagel condensation at C3 followed by dehydration and intramolecular addition (Scheme 27).^185^ The obtained methyl 2-amino-3-cyanobenzofuran-5-carboxylate was further alkylated to produce highly substituted benzofurans.^185^

##### 2.3.6.5 From 2-Pyrone and Derivatives

**Scheme 28:**
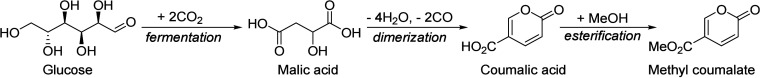
Production of Coumalic Acid and Methyl Coumalate from Glucose via Malic Acid

2-Pyrone is a well-studied unit for the construction of aromatic rings through Diels–Alder (DA) reactions followed by decarboxylation and/or dehydrogenation.^186,187^ Coumalic acid is a 2-pyrone-containing compound that can be prepared by dimerization of malic acid, an important “top 10” carbohydrate-originating platform molecule (Scheme 28).^159,188,189^ Esterification of coumalic acid under acidic conditions lead to the formation of methyl coumalate, a popular coumalic acid derivative for aromatic synthesis.

**Scheme 29:**
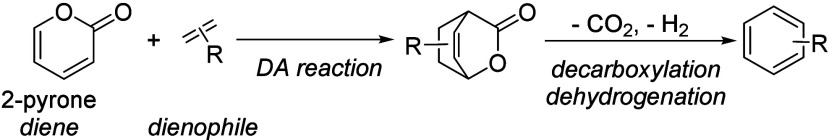
DA Reaction of 2-Pyrone and Olefins Followed by Dehydrogenation and Decarboxylation to Aromatics

Olefins react with 2-pyrones to oxabicyclo[2.2.2]octene intermediates, which can then be converted to aromatics after loss of one molecule CO_2_ and one molecule H_2_ (Scheme 29).

**Scheme 30:**
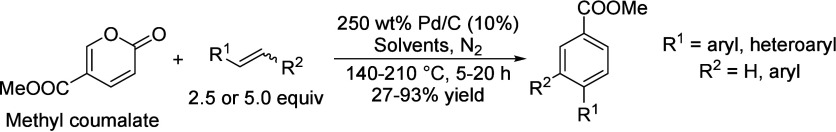
Methyl Benzoates from Reactions of Methyl Coumalate with Aryl-Substituted Olefins^193^

Although the reaction of methyl coumalate with olefins to generate the DA cycloadducts was traced back to 1969,^190−192^ the first aromatics production was reported in 1994 by Matsui, who prepared a range of substituted methyl benzoates using aryl-substituted alkenes (Scheme 30).^193^ In the presence of 250 wt% Pd/C (10%), methyl coumalate reacted with the olefins at 140–210 °C in nonpolar solvents, including *m*-xylene, mesitylene, and dodecane, generating the corresponding methyl benzoates in 27–93% yields.^193^

**Scheme 31:**
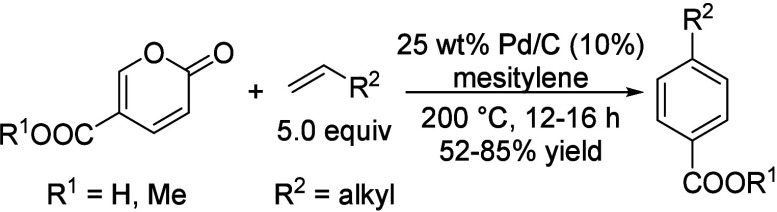
Reactions of Methyl Coumalate or Coumalic Acid with Terminal Alkenes to *Para*-Substituted Benzoates^194,199^

**Scheme 32:**
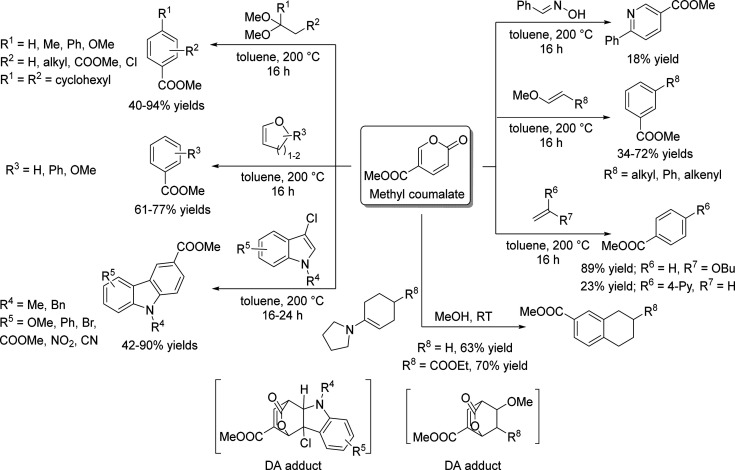
DA/Decarboxylation of Methyl Coumalate and Substituted Olefins or Analogs^195−198^

In 2011, Kraus extended this approach to coumalic acid and α-alkylated alkenes and he decreased the amount of Pd/C (10%) to 25 wt% (Scheme 31).^194^ Only *para*-substituted methyl benzoates or benzoic acids were formed in 52–85% yields.^194^ Shortly after, Kraus reported the same transformation without catalyst in toluene at 200 °C by employing a range of olefins containing a leaving group in the form of a halide or an alkoxy group (Scheme 32), including vinyl ethers,^195,196^*N*-alkyl-3-chloroindoles,^197^ saturated acetals,^195,196^ and benzaldehyde oxime (leading to the formation of the pyridine).^196^ A number of bio-based methyl benzoates with various substituents were synthesized in moderate to high yields.^195−197^ The DA addition of vinyl ethers to methyl coumalate gave 8-alkoxy-oxabicyclo[2.2.2]octenes as products, which were readily converted to the aromatic products by extruding 1 equiv of CO_2_ and 1 equiv of alcohol.^195,196^ The elimination of the alcohol was much easier than dehydrogenation using Pd/C as catalyst.^195,196^ Saturated acetals can eliminate one alcohol molecule to generate vinyl ethers, and hence, also react with methyl coumalate to produce bio-based aromatics.^195,196^ Similarly, *N*-alkyl-3-chloroindoles as dienophiles deliver 7-chloro-oxabicyclo[2.2.2]octenes as DA adduct, which readily eliminate 1 equiv of HCl and 1 equiv of CO_2_ to generate the aromatic product.^197^ Pyrrolidine enamines were also used for the DA reaction of methyl coumalate at room temperature to form aromatics by extruding CO_2_ and pyrrolidine.^198^

**Scheme 33:**
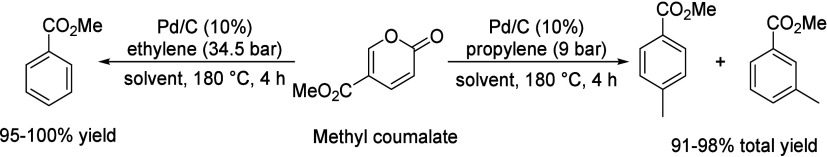
Syntheses of Methyl Benzoate and Methyl Toluate from Methyl Coumalate^200,201^

Although the reaction of various dienophiles with methyl coumalate were comprehensively studied by Kraus, the use of cheap gaseous ethylene and propylene as dienophiles was only described by Shanks in recent years (Scheme 33).^200,201^ At 180 °C, methyl coumalate was converted to methyl benzoate in 95–100% yields under 34.5 bar of ethylene in the presence of Pd/C (10%) catalyst in toluene, 1,4-dioxane, or γ-valerolactone (GVL) as solvent,^200^ while a mixture of methyl *p*-toluate and methyl *m*-toluate was obtained in 91–98% yield under 9 bar propylene (Scheme 33).^201^

**Scheme 34:**
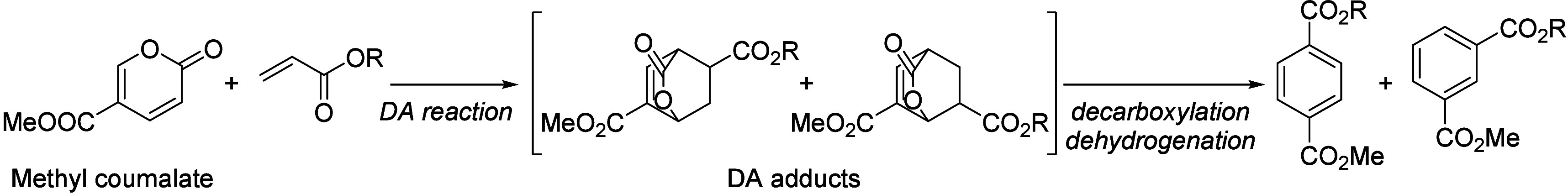
Terephthalate and Isophthalate from DA Reaction of Methyl Coumalate and α,β-Unsaturated Carbonyl Compounds (Acrylate as Example)

In view of the irreplaceable role of terephthalic acid in polymers, its synthesis from renewable resources is of great interest. DA reactions of methyl coumalate and α,β-unsaturated carbonyl compounds form oxabicyclo[2.2.2]octene intermediates containing one methoxycarbonyl group and one carbonyl group (For a general example see Scheme 34). The ensuing selective decarboxylation and dehydrogenation leads to the formation of mixtures of terephthalates and isophthalates.

**Scheme 35:**
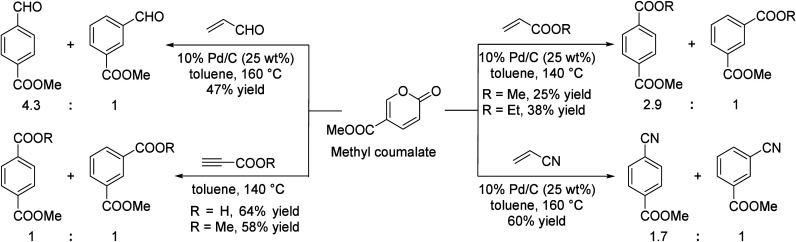
Preparation of Terephthalic Acid and Isophthalic Acid Precursors from Methyl Coumalate^202^

Kraus tested acrylates, acrylonitrile, and acrolein as dienophiles in the DA/decarboxylation/dehydration reaction with methyl coumalate, providing mixtures of terephthalic acid and isophthalic acid precursors in 25–60% yields (Scheme 35).^202^ The ratios of *p*-substituted to *m*-substituted products were only 1.7–4.3.^202^ Kraus also reported the preparation of terephthalates and isophthalates from the DA/decarboxylation reaction of methyl coumalate with propiolic acid or methyl propiolate, providing the aromatic mixtures in 64% and 58% yields at 140 °C using toluene as solvent without catalyst (Scheme 35).^202^

**Scheme 36:**
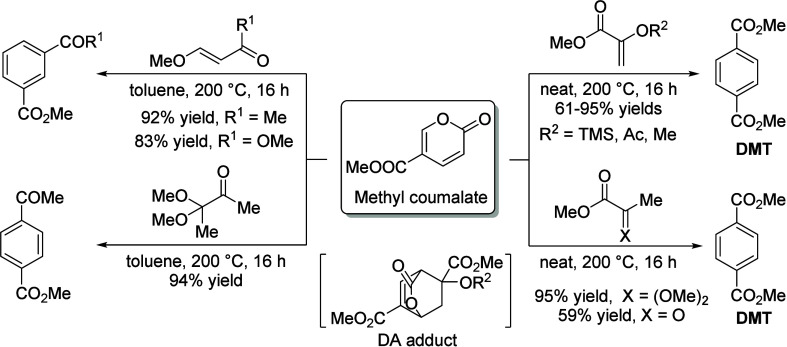
Conversion of Methyl Coumalate to Terephthalate and Isophthalates^203^

As discussed before, vinyl ethers and acetals reacting as dienophiles with methyl coumalate readily produced aromatics without any catalyst.^195,196^ The reaction of 3,3-dimethoxybutan-2-one with methyl coumalate in toluene at 200 °C produced methyl 4-acetylbenzoate in 94% yield (Scheme 36). The use of *trans*-3-methoxyacrylate as dienophile in the same reaction produced dimethyl isophthalate in 83% yield.^203^ Under neat conditions, methyl 2-hydroxyacrylates with *o*-protecting groups reacted with methyl coumalate via DA addition followed by decarboxylation and elimination of alcohols, forming dimethyl terephthalate (DMT) in 61–95% yields.^203^ Methyl pyruvate and methyl 2,2-dimethoxypropanoate convert *in situ* to methyl 2-hydroxy- or methoxy-acrylates by isomerization or elimination of methanol and react with methyl coumalate under neat conditions, affording DMT in 59% and 95% yield, respectively.^203^

**Scheme 37:**
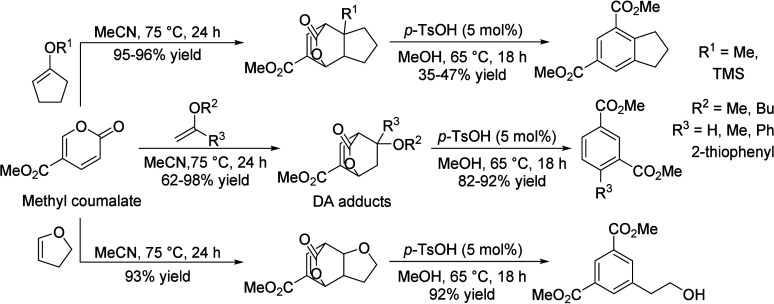
Transformations of Methyl Coumalate to Substituted Isophthalates^204^

Aromatization of the DA adduct from methyl coumalate and olefins without elimination of CO_2_ led to the formation isophthalates, thus preserving all carbon atoms from methyl coumalate (Scheme 37).^204^ At 75 °C, cycloadducts were produced from methyl coumalate and vinyl ethers in MeCN and then converted to a series of substituted isophthalates by elimination of alcohols and lactone transesterification catalyzed by 5 mol% *p*-toluenesulfonic acid (*p*-TsOH) in methanol.^204^ Reaction of 2,3-dihydrofuran with methyl coumalate under these conditions produced dimethyl 5-hydroxyethyl-isophthalate in 86% yield.^204^

**Scheme 38:**
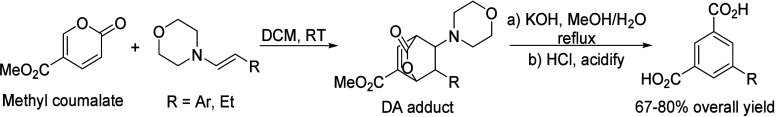
Syntheses of 5-Substituted Isophthalic Acids from Methyl Coumalate and Piperidine Enamines^198^

Piperidine enamines with *trans*-substituents reacted with methyl coumalate in dichloromethane at room temperature to form the bicyclic lactones. After ring-opening and elimination of piperidine, these afforded the 5-substituted isophthalates, which were hydrolysed to the 5-substituted isophthalic acids (Scheme 38).^198^ The overall yields were 67–80%.^198^

**Scheme 39:**
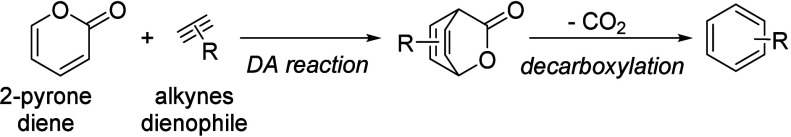
Alkynes as Dienophiles for the DA Reaction with 2-Pyrones^205^

**Scheme 40:**
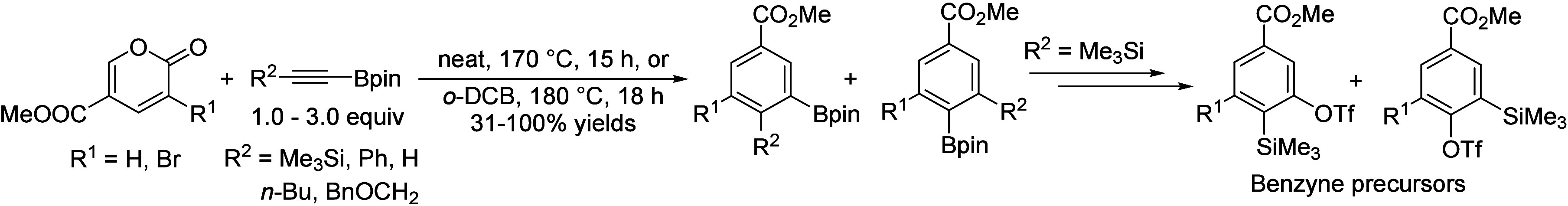
Conversion of Methyl Coumalate or Methyl 3-Bromocoumalate to Phenylboronic Esters (206)

Alkynes are good dienophiles in the DA reaction of 2-pyrones to generate aromaticity upon decarboxylation (Scheme 39).^205^ In 2008, Harrity reported the reaction of methyl coumalate with substituted alkynylboronates in *o*-dichlorobenzene (*o*-DCB) or neat to synthesize aromatic boronic esters in 31–100% yields (Scheme 40). Methyl 3-bromocoumalate was also demonstrated to react with alkynylboronates in *o*-DCB generating bromo-substituted phenylboronic esters in 77–82% yields.^205^ Use of trimethylsilyl alkynylboronate as dienophile in the reaction produced *o*-trimethylsilylphenylboronic esters in 77–100% yields, which were further converted to functionalized *o*-trimethylsilylphenyl triflates, valuable benzyne precursors.^206^

**Scheme 41:**
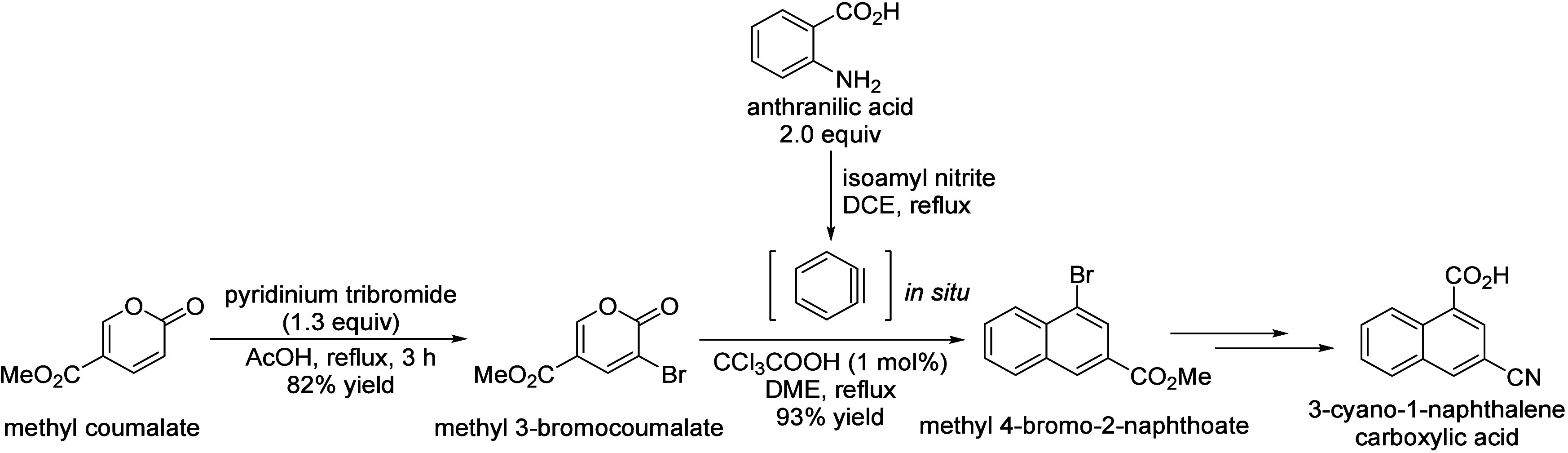
Synthesis of Methyl 4-Bromo-2-naphthoate from Methyl Coumalate via Methyl 3-Bromocoumalate^207^

**Scheme 42:**
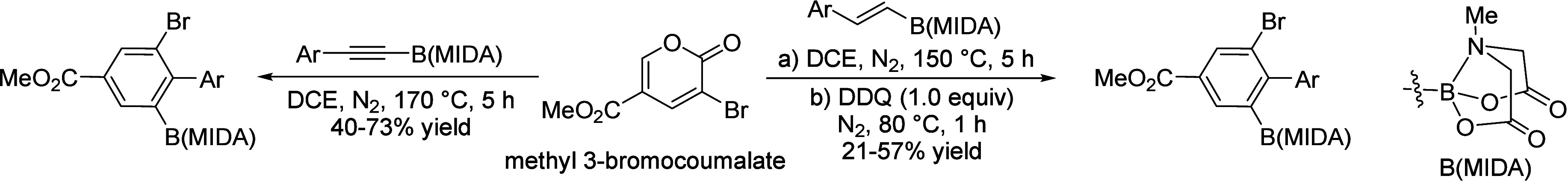
Conversion of Methyl 3-Bromocoumalate to Aromatic Boronates^208^

Bromination of methyl coumalate with pyridinium tribromide in acetic acid formed methyl 3-bromocoumalate in 82% yield. This was reacted with benzyne, formed *in situ* from anthranilic acid and isoamyl nitrite, catalyzed by 1 mol% trichloroacetic acid in ethylene glycol dimethyl ether (DME) to furnish methyl 4-bromo-2-naphthoate in 93% yield (Scheme 41).^207^ Methyl 4-bromo-2-naphthoate was further converted to 3-cyano-1-naphthalenecarboxylic acid, an intermediate for drug syntheses.^207^ Methyl 3-bromocoumalate reacted with alkynyl MIDA (*N*-methyliminodiacetyl) boronates in 1,2-dichloroethane (DCE) at 170 °C to give aromatic boronates in 40–73% yields. Its reaction with alkenyl MIDA boronates followed by oxidation with DDQ (2,3-dichloro-5,6-dicyano-1,4-benzoquinone) at 80 °C in a one-pot procedure produced aromatic boronates in 21–57% yields (Scheme 42).^208^

**Scheme 43:**
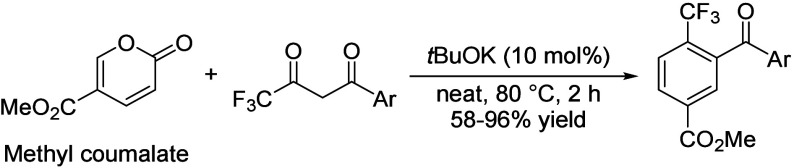
Syntheses of Methyl *p*-CF_3_-Benzoates from Methyl Coumalate^209^

**Scheme 44:**
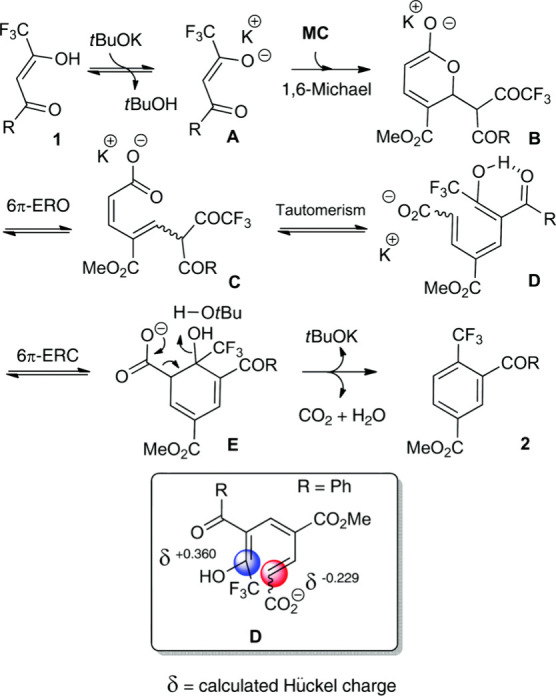
Proposed Mechanism of Formation of Dimethyl 4-(trifluoromethyl)isophthalate from Methyl Coumalate Reproduced with permission from ref (209). Copyright 2018 The Royal Society of Chemistry.

Thorimbert reported an interesting conversion of methyl coumalate to *p*-CF_3_-substituted methyl benzoates involving a 6π-electrocyclic ring closure mechanism. Catalyzed by 10 mol% *t*BuOK, *p*-CF_3_-substituted methyl benzoates were obtained in 58–96% yields from methyl coumalates and trifluoromethyl-β-diketones under neat condition at 80 °C (Scheme 43).^209^ The authors suggested that this transformation involves a 1,6-Michael addition, a 6π-electrocyclic ring opening (6π-ERO), tautomerization, and a 6π-electrocyclic ring closure to generate the aromatic ring (Scheme 44).^209^

**Scheme 45:**
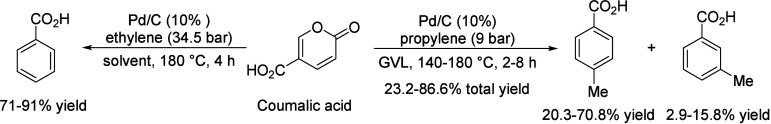
Syntheses of Benzoic Acid and Toluic Acids from Coumalic Acid^200,210^

In 2011, Kraus reported the direct use of coumalic acid as diene to prepare *p*-substituted benzoic acids in 65–85% yields from reactions with substituted alkenes catalyzed by Pd/C at 200 °C (Scheme 31).^194^ After that, the direct syntheses of aromatics from coumalic acid was rarely reported until 2017, when Shanks and co-workers synthesized benzoic acid and toluic acids from coumalic acid catalyzed by Pd/C (10%) using gaseous ethylene and propylene as dienophiles (Scheme 45).^200,210^ Under 34.5 bar ethylene pressure, coumalic acid was converted to benzoic acid in 71–91% yields at 180 °C using toluene, 1,4-dioxane, γ-valerolactone (GVL), or acetone as solvent.^200^ Methyl coumalate subjected to the same conditions afforded methyl benzoate in 95–100% yield.^200^ Using GVL as solvent, coumalic acid reacted with 9 bar propylene at 140–180 °C and produced 20.3–70.8% yields of *p*-toluic acid along with 2.9–15.8% yields of *m*-toluic acid.^210^ When toluene and 1,4-dioxane were used as solvent at 180 °C, *m-* and *p*-toluic acids were produced in 51% and 88% yield, respectively.^201^

**Scheme 46:**
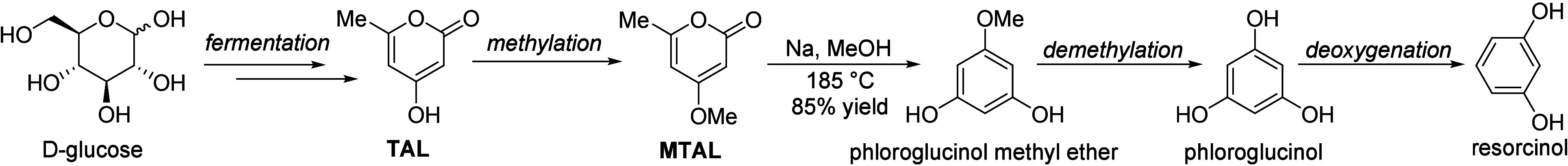
Synthesis of Phloroglucinol Methyl Ether from Glucose via TAL and MTAL^215,216^

Triacetic acid lactone (**TAL**, 4-Hydroxy-6-methyl-2*H*-pyran-2-one) is a fermentation product of glucose and commonly used for polyketide synthesis.^211−213^ It can also be obtained by deacetylation of dehydroacetic acid (3-Acetyl-2-hydroxy-6-methyl-4*H*-pyran-4-one), but this entails a multi-step synthesis starting from acetic acid or acetone.^214^ Methylation of **TAL** under basic conditions results in the formation of the methyl ether of **TAL** (**MTAL**), which was converted to phloroglucinol methyl ether in 85% yield by reaction with Na in methanol as solvent at 185 °C (Scheme 46).^215,216^ The obtained phloroglucinol methyl ether was demethylated to phloroglucinol, which was deoxygenated to resorcinol under H_2_.^215^

**Scheme 47:**
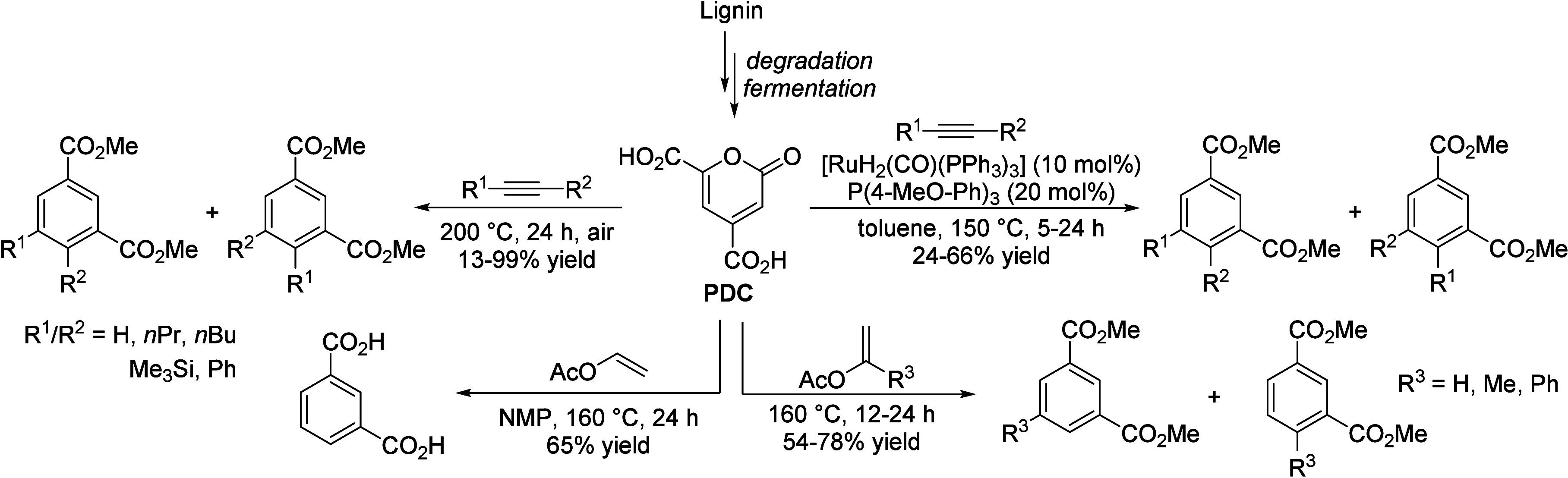
Bio-Based Aromatics from Lignin-Derived PDC^218^

2-Pyrone-4,6-dicarboxylic acid (PDC) can be obtained by fermentation of protocatechuate which may be obtained by lignin degradation.^217^ Its transformation to isophthalates is also presented in this section for comparison (Scheme 47). DA reactions of PDC with alkynes at 200 °C produced 4,5-disubstituted isophthalates in 13–99% yields, while the same reaction catalyzed by a ruthenium complex with tris-*p*-anisylphosphine as ligand generated 24–66% yields in toluene at 150 °C.^218^ Reaction of PDC with substituted vinyl acetates at 160 °C delivered 4- and 5-subtituted isophthalates in 54–78% yields. Addition of *N*-methylpyrrolidone (NMP) to the reaction produced isophthalic acid in 65% yield directly from the reaction between PDC and vinyl acetate.^218^

**Scheme 48:**
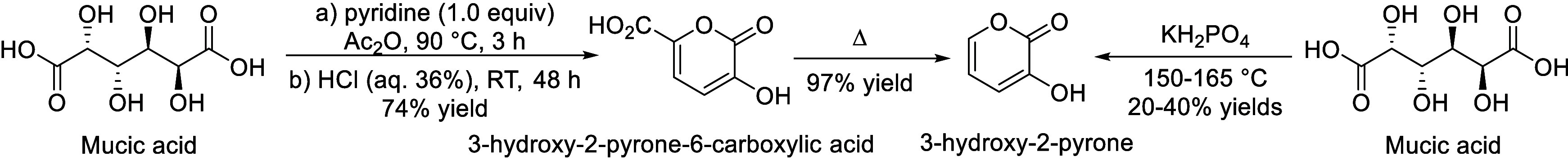
Preparation of 3-Hydroxy-2-pyrones from Mucic Acid^219−221^

3-Hydroxy-2-pyrone can be prepared in 20–40% yield by heating mucic acid at 150–165 °C in the presence of KH_2_PO_4_ in a distillation apparatus (Scheme 48).^219,220^ Sebastiano and co-workers reported the transformation of mucic acid in a two-step reaction to 3-hydroxy-2-pyrone-6-carboxylic acid (Scheme 48) in 74% yield. This compound was decarboxylated to 3-hydroxy-2-pyrone in 97% yield by sublimination.^221^

**Scheme 49:**
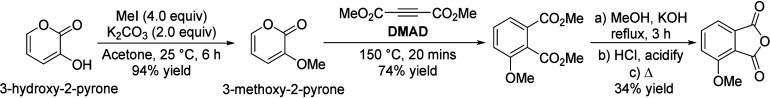
Synthesis of 3-Methoxyphthalic Anhydride from 3-Hydroxy-2-pyrone^220^

In 1975, Watt reported a methylation/DA reaction/decarboxylation reaction sequence to dimethyl 3-methoxyphthalate from 3-hydroxy-2-pyrone (Scheme 49).^220^ 3-Methoxy-2-pyrone, obtained from 3-hydroxy-2-pyrone in 94% yield by reaction with methyliodide under basic conditions, reacted with dimethyl acetylenedicarboxylate (DMAD) at 150 °C to form dimethyl 3-methoxyphthalate in 74% yield.^220^ Dimethyl 3-methoxyphthalate was converted to 3-methoxyphthalic anhydride in 34% yield via ester hydrolysis followed by acid-catalyzed dehydration.^220^

**Scheme 50:**
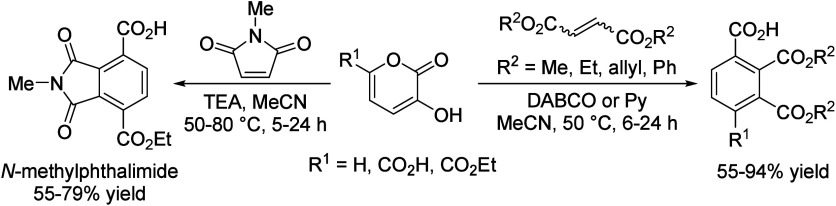
Syntheses of Aromatic Carboxylates and *N*-Methyl Phthalimide from 3-Hydroxy-2-pyrones^222^

Truscello reported the reaction of substituted 3-hydroxy-2-pyrones with fumarates and maleates at 50 °C to substituted aromatic carboxylates in 55–94% yields in the presence of DABCO (1,4-diazabicyclo[2.2.2]octane) or pyridine (Scheme 50).^222^ Ethyl 3-hydroxy-2-pyrone-6-carboxylate reacted with *N*-methylmaleimide in acetonitrile catalyzed by triethylamine (TEA) to form the substituted *N*-methylphthalimide in 55% yield at 50 °C and 79% yield at 80 °C.^222^

##### 2.3.6.6 From Myo-inositol

**Figure 7:**
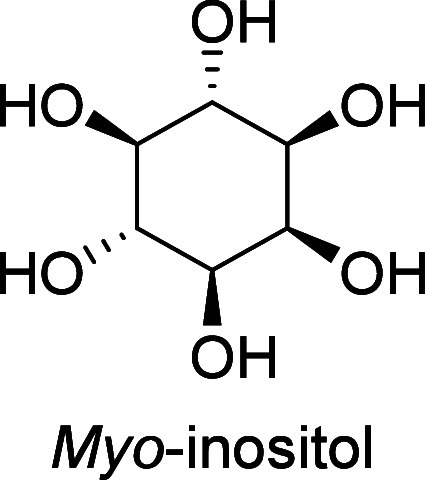
Structure of myo-inositol

Inositol is a naturally existing carbocyclic sugar with nine theoretical stereoisomers which finds use as food supplement. *Myo*-inositol is the most abundant isomer and is produced by acid-catalyzed hydrolysis of phytate (inositol hexakisphosphate) obtained from corn, although fermentative and biocatalytic approaches are also known.^223^ In view of its cyclohexane ring with six hydroxyl groups (Figure 7), its conversion to aromatics is mainly achieved by two pathways: dehydration to phenols and deoxydehydration (DODH) to benzene.

**Scheme 51:**
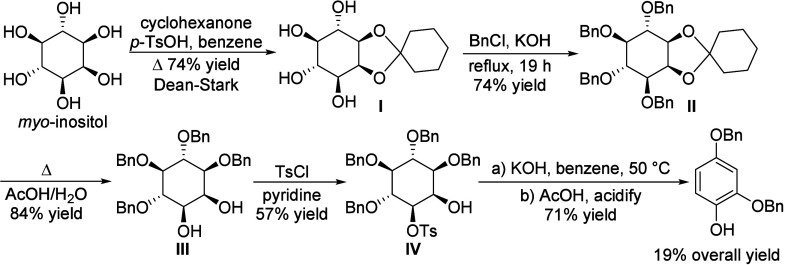
Conversion of Myo-inositol to Aromatics^224,225^

In 1965, Tate reported the conversion of *myo*-inositol to 2,4-dibenzyloxyphenol via inositol derivatives as intermediates through a five-step pathway (Scheme 51).^224,225^ Selective protection of *myo*-inositol with cyclohexanone formed acetal **I** (1,2-O-cyclohexylidene-*myo*-inositol) in 74% yield, which then reacted with benzyl chloride (BnCl) at reflux under basic conditions, delivering 1,4,5,6-tetra-O-benzyl-2,3-cyclohexylidene-*myo-*inositol (**II**) in 74% yield. Compound **II** was selectively hydrolyzed to 1,4,5,6-tetra-O-benzyl-*myo*-inositol (**III**) under acidic conditions in 84% yield. Tosylation of **III** with *p*-toluenesulfonyl chloride (TsCl) afforded **IV** in 57% yield, which was converted to 2,4-dibenzyloxyphenol in 71% yield via a threefold elimination.^224,225^ The overall yield of 2,4-dibenzyloxyphenol from *myo*-inositol was 19%.^224,225^

**Scheme 52:**
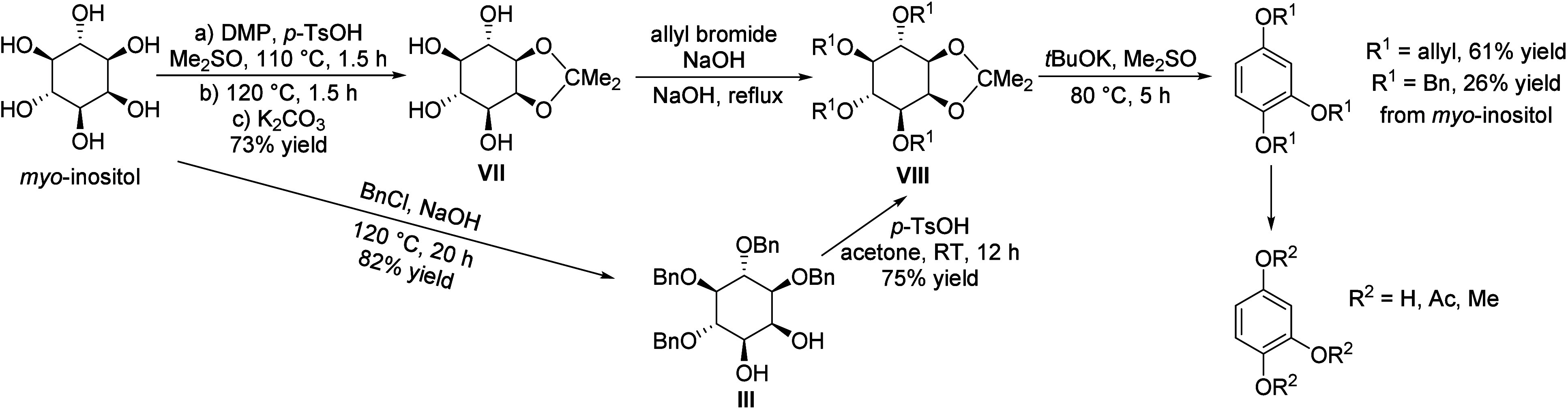
Syntheses of 1,2,4-Trisubstituted Benzene from *Myo*-inositol^226,227^

Gigg improved this approach via 1,2-*O*-isopropylidene-*myo*-inositol (**VII**) as intermediate and shortened the pathway to 3–4 steps (Scheme 52).^226,227^ 1,2,4-Trisbenxyloxybenzene and 1,2,4-tris(prop-1-enyloxy)benzene were obtained in 26% and 61% yields, respectively, and further converted to 1,2,4-trisalkoxybenzenes and 1,2,4-trihydroxybenzene.^226,227^

**Scheme 53:**
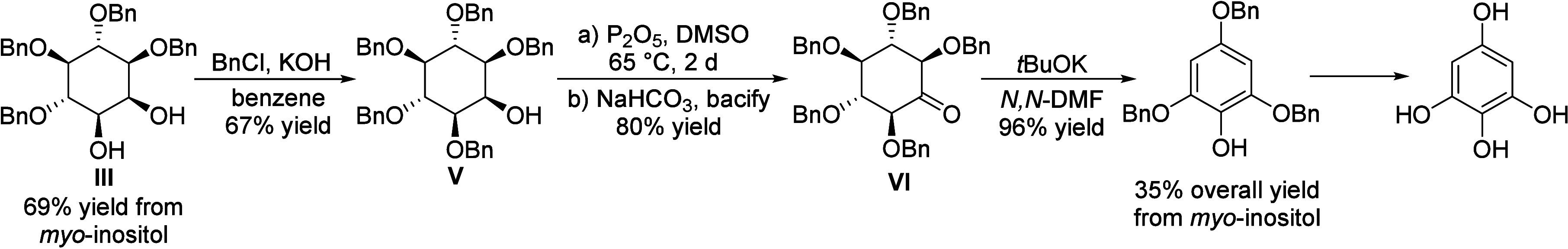
Production of 2-Hydroxy-1,3,5-tribenzyloxybenzene from *Myo*-inositol^228^

Reckendorf utilized the same approach as Tate and synthesized **III** from *myo*-inositol in 69% yield (Scheme 53).^228^ Penta-O-benzyl-*myo*-inositol (**V**) was obtained by etherification of **III** with BnCl and oxidized to inosose **VI** by P_2_O_5_/DMSO. The elimination of two benzyl alcohol units from **VI** under basic conditions delivered 2-hydroxy-1,3,5-tribenzyloxybenzene. The overall yield of 2-hydroxy-1,3,5-tribenzyloxybenzene from *myo*-inositol was 35%.^228^ 2-Hydroxy-1,3,5-tribenzyloxybenzene was deprotected to 1,2,3,5-tetrahydroxybenzene.^228^

**Scheme 54:**
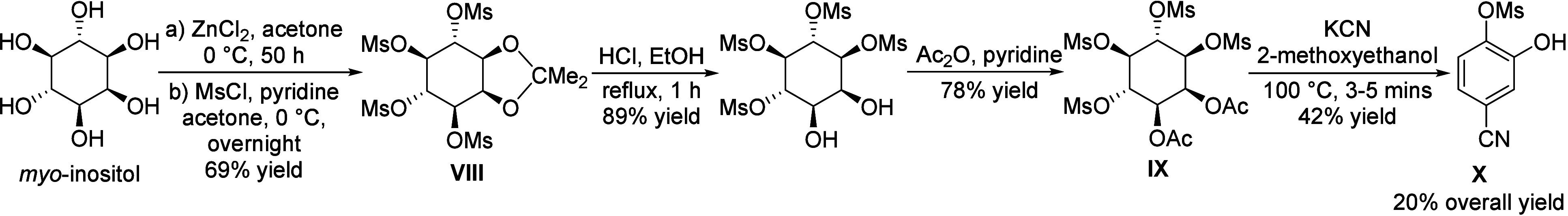
Production of 4-Cyano-2-hydroxyphenyl Methanesulfonate (X) from *Myo*-inositol^229,230^

Guided by the same protection-deprotection strategy, Cadenas prepared 2,3-di-*O*-acetyl-1,4,5,6-tetra-*O*-methylsufonyl-myoinositol (**IX**) from *myo*-inositol and addressed its transformation to 4-cyano-2-hydroxyphenyl methanesulfonate (**X**) by nucleophilic attack of cyanide followed by elimination of alcohols (Scheme 54).^229,230^ The overall yield of **X** from *myo*-inositol was 20%.^229,230^

**Scheme 55:**
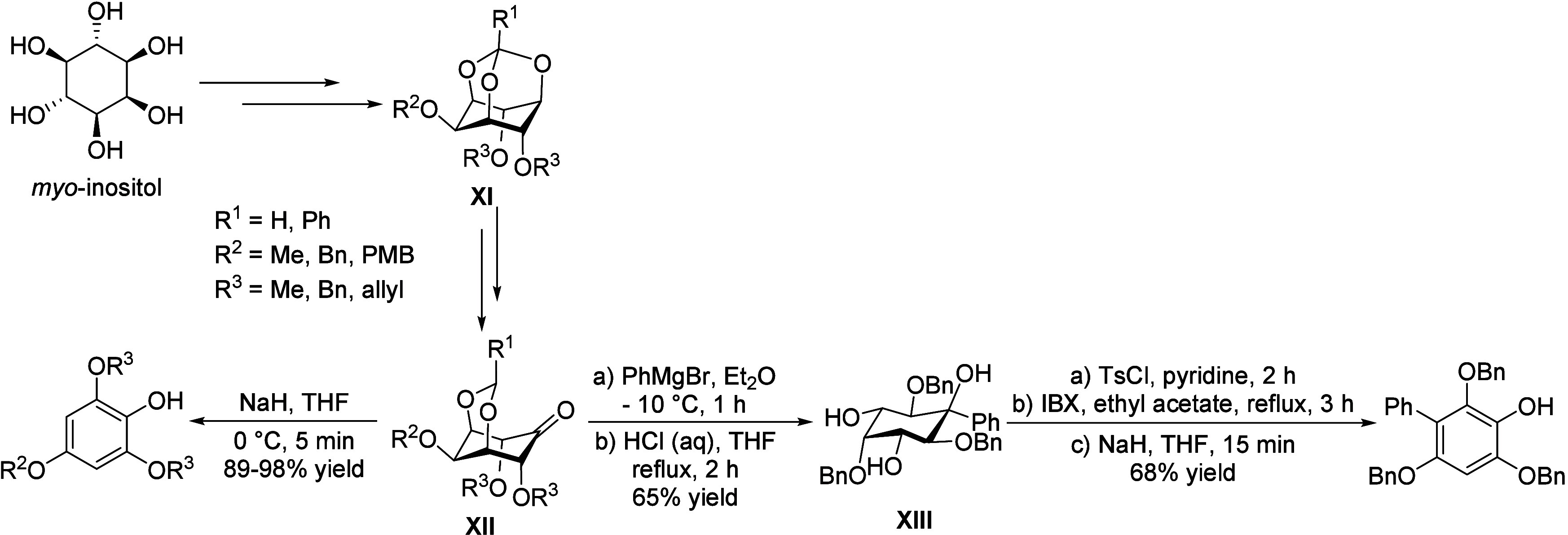
Schashidhar’s Approach to Polyoxygenated Aromatics from *Myo*-inositol^231,232^

Schashidhar reported the preparation of poly-oxygenated aromatics from *myo*-inositol via *myo*-inositol 1,3,5-orthoformates (**XI**) as intermediates through five or eight steps reactions (Scheme 55).^231,232^ Selective ring opening of **XI** followed by oxidation led to the formation of ketone **XII**, which was converted to O-protected polyoxygenated benzenes through elimination of alcohols and isomerization.^231,232^

Although the overall yields of aromatics from *myo*-inositol are satisfactory, this protection-deprotection approach suffers from lengthy procedures with very low atomic economy, making it unlikely these methods will be used for large-scale production.

**Scheme 56:**
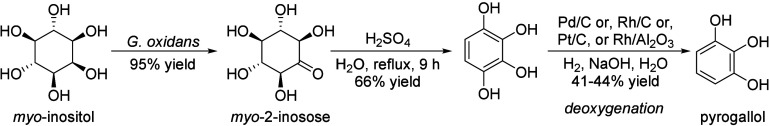
Direct Conversion of *Myo*-inositol to 1,2,3,4-Tetrahydroxybenzene and Pyrogallol^215,233^

Frost found that *Gluconobacter oxidans ATCC 621* can oxidize *myo*-inositol to *myo*-inosose in 95% yield, which was further converted to 1,2,3,4-tetrahydroxybenzene through acid-catalyzed dehydration in 66% yield (Scheme 56).^233^ Deoxygenation of the obtained 1,2,3,4-tetrahydroxybenzene under H_2_ catalyzed by Pd/C, Rh/C, Pt/C, or Rh/Al_2_O_3_ afforded pyrogallol in 41–44% yields.^215^ Compared to the protection-deprotection approach, this method is superior because of its high atom-economy, less waste generated, fewer steps, and comparable aromatics yield (63% from *myo*-inositol).

**Scheme 57:**
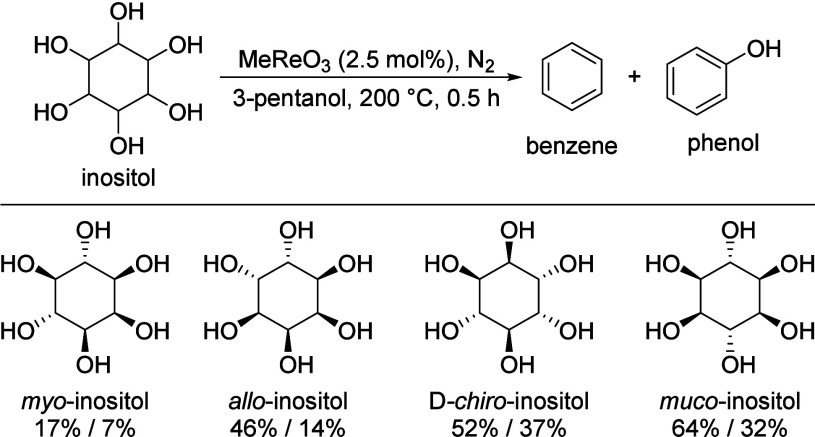
DODH of Inositols to Benzene and Phenol^144^

**Scheme 58:**
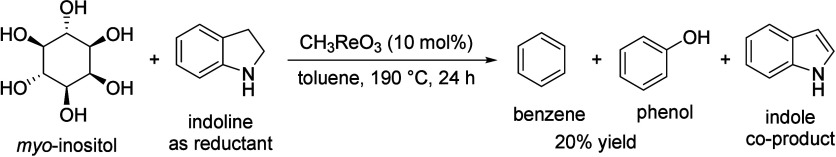
DODH of *Myo*-inositol to Benzene and Phenol with Indoline as Reductant^236^

Deoxydehydration (DODH) of vicinal diols to olefins attracted a lot of attention as methodology for the upgrade of bio-based polyols.^234,235^ The removal of three vicinal diol groups in inositol generates benzene as product, while phenol is formed by eliminating two vicinal diol groups and one water molecule. In 2012, Toste reported the DODH reaction of inositol catalyzed by 2.5 mol% MeReO_3_ with 3-pentanol as reductant (Scheme 57).^144^ At 200 °C, benzene and phenol were obtained from *myo*-inositol in 17% and 7% yield respectively.^144^ As DODH only eliminates *cis*-diol groups, the isomerization of *myo*-inositol must happen during the reaction. Other isomers, such as *allo*-, d-*chiro*-, and *muco*-inositol were all tested under the same conditions, affording benzene and phenol in 46–64% and 14–32% yields respectively.^144^ Indoline can also serve as reductant in the DODH of *myo*-inositol to benzene and phenol giving indole as co-product.^236^ Catalyzed by 10 mol% MeReO_3_, a mixture of benzene and phenol in 20% yield was obtained at 190 °C in toluene as solvent (Scheme 58).^236^

In view of the high cost of myo-inositol and the lengthy synthetic procedures, large scale application of the methods described above to bulk aromatic products, such as phenol and benzene, seems highly unlikely.

### 2.4 Aromatics from Hydrogenation Products of Sugars

#### 2.4.1 Aromatics from Sorbitol

**Scheme 59:**
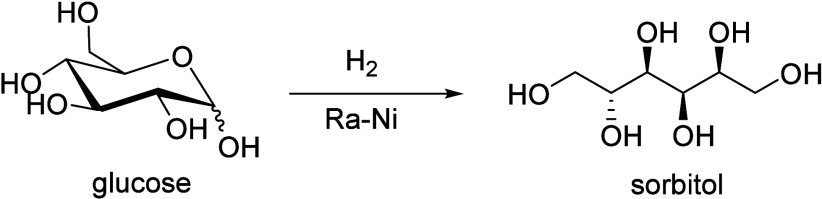
Hydrogenation of Glucose to Sorbitol

Sugars are relatively easily hydrogenated to their poly-hydroxy analogues. Hydrogenation of glucose gives sorbitol, a process practiced on very large scale (Scheme 59). World-wide production is probably in excess of 500 000 ton/y.

A number of publications exist on the CFP of sorbitol to aromatics, although many of these studies were aimed at producing fuels,^237^ rather than just aromatics (Table 14).

**Table 14:** Catalytic Fast Pyrolysis of Aqueous Sorbitol Solutions

Entry	Catalyst	Si/Al	*T* ( °C)	WHSV (h^–1^)	TOS	Selectivity to BTX (%)	B:T:X	Ref
1	H-ZSM-5	–	500		30 s	20^a^	–	(238)
2	H-ZSM-5	30	600	11.7	20 min	13	4:6:3	(239)
3^b^	Ni(3)H-ZSM-5 /MCM-41	–	320	0.75	–	34^a^	–	(240)
4^b^,^c^	Ni(10)H-ZSM-5 /MCM-41	38	300	1.25	–	27^a^	–	(241)
5^b^	Ni(10)H-ZSM-5	38	280	1.5	–	32^a^	–	(242)
6^b^	Ni(10)H-ZSM-5	38	280	2.25	3 h	25^d^	–	(243)
7^b^	Ni(10)H-β	25	280	2.25	3 h	5^d^	–	(243)
8^b^	Ni(10)-H-ZSM-5/SBA-15	38	320	0.75	70 h	10	0:2:8	(24)
9^e^	H-ZSM-5/SiO_2_^f^	–	450	1.0	–	17	1:4:11	(244)

a Selectivity to aromatics.

b At 40 bar hydrogen pressure.

c A mixture of 60% sorbitol and 40% xylitol in water.

d Selectivity to substituted benzenes.

e Sorbitol/MeOH (1:3).

f 15% SiO_2_.

Corma and co-workers screened a range of catalysts for the CFP of a range of renewable materials including sorbitol as 50% aqueous solution. The reaction was performed at a small scale at a relatively high temperature of 500 °C. Using ZSM-5 as catalyst an overall selectivity to aromatics of 20% was obtained (Table 14, entry 1).^238^ Huber and co-workers performed similar chemistry on a 15% aqueous solution of sorbitol at the even higher temperature of 600 °C and a high WHSV of 11.7 h^–1^. They recorded a selectivity of 13% to BTX (Table 14, entry 2).^239^

The use of nickel-doped zeolites was investigated by Wang, Ma and co-workers. They used catalysts which are based on a mixture of H-ZSM-5 and MCM-41 (3:2) doped with different amounts of nickel. The reactions were performed on aqueous solutions of sorbitol over a fixed-bed reactor at a hydrogen pressure of 40 bar. The highest aromatics yield (34%) was obtained with 3 wt% of Ni doping at 320 °C (Table 14, entry 3).^240^ The authors noted that at lower temperatures substantial amounts of isosorbide where found. A similar catalyst with 10 mol% of Ni doping was used on an aqueous mixture of sorbitol and xylitol which presumably could be obtained from lignocellulose by hydrolytic hydrogenation. Here a selectivity to aromatics of 27% was reached (Table 14, entry 4).^241^ Good results were also obtained with nickel (10%) doped H-ZSM-5 under similar conditions were a selectivity to aromatics of 32% was found (Table 14, entry 5).^242^ A direct comparison between Ni-doped H-ZSM-5 and Nickel doped H-β showed that use of the latter resulted in a poor yield of substituted benzenes of only 5% (Table 14, entry 7).^243^ A Ni-doped catalyst based on a mixture of ZSM-5 and SBA-15 was rather stable and could be used for 70h TOS during which the conversion of sorbitol remained at 100% and the oil yield stayed between 35% and 40%.^24^ The selectivity to BTX was rather low at 10% (Table 14, entry 8). A large part of the aromatic fraction also consisted of naphthalenes.

Li and co-workers modified the ZSM-5 surface with silica to enhance the amount of xylenes in the aromatic fraction. They investigated different loadings of silica in the CFP of sorbitol/methanol mixtures and although the amount of aromatics decreased from 20 to 17% with increasing amount of silica, within the aromatics fraction, the selectivity to *p*-xylene increased from 5% at 0% silica to 11% at 15% of silica (Table 14, entry 9).^244^

Overall, the CFP of sorbitol leads to the formation of mixtures of alkanes and aromatics, which would not be easy to separate. These mixtures could be used as fuel, but isolation of the individual aromatics from these mixtures will be too costly.

#### 2.4.2 Aromatics from Hexane

Hexane can be generated from renewable resources. The production of hexane directly from cellulose in 83% yield was reported by Tomishige and co-workers.^245^ In this process the same catalyst catalyzes the hydrolysis of cellulose to glucose, the hydrogenation of glucose to sorbitol, and finally the hydrogenation of sorbitol to hexane. This opens up a route from cellulose via hexane to aromatics via CFP of hexane for which a number of reports exist (Table 15).

**Table 15:** Catalytic Fast Pyrolysis of *n*-Hexane

Entry	Catalyst (Metal loading)	Structural features	Reaction conditions (gas mixture, temperature, space velocity, or flow rate)	*n*-hexane conversion	Product selectivity	Ref
1	Galloalumino silicate	Si:Al:Ga = 78:1:1.3	100% *n*-hexane; 500 °C; 2 h^–1^	–	63% yield of aromatics	(246)
	H-Si-Al-Ga					
2	Zn/H-ZSM-5 (1–3%)	Si:Al = 96:1	*n*-hexane/methanol = 7:3, 1:1, and 3:7; 430–470 °C; 0.5–2 h^–1^	100%	42–48% benzene	(247)
	ZnxCry/H-ZSM-5 (Zn/Cr = 1:1 to 1:6)			82–60%	25–37% benzene	
3	Pt/MgO (0.52%)	220 m^2^g^–1^	6% *n*-hexane/He; 477 °C	47% (1 min) to 18% (30 h)	benzene: 45% to 23%	(248)

Aromatization of *n*-hexane was studied by Kanai and Kawata on Ga-based silicates or aluminosilicates.^246^ The authors showed that galloaluminosilicate and gallosilicate catalysts have significantly higher catalytic activity for the conversion of *n*-hexane to aromatics than Ga^3+^-exchanged ZSM-5 or Ga_2_O_3_/H-ZSM-5 catalysts, with a total aromatics yield of H-Si-Al-Ga of 63% at 500 °C (Table 15, entry 1). These authors concluded that for the ZSM-5-based catalysts the active Ga species is extra-framework (non-framework Ga). Kanai also studied the impact of the annealing temperature on the activity of Ga exchanged H-ZSM-5 and found that increasing the annealing temperature results in a pronounced increase in the activity of these cataylts.^249^ Lahna et al. studied the same reaction on gallosilicates with MFI structure.^250^ These authors demonstrated that activity for *n*-hexane conversion increases continuously with increasing the Ga content in the gallosilicates framework.

**Figure 8:**
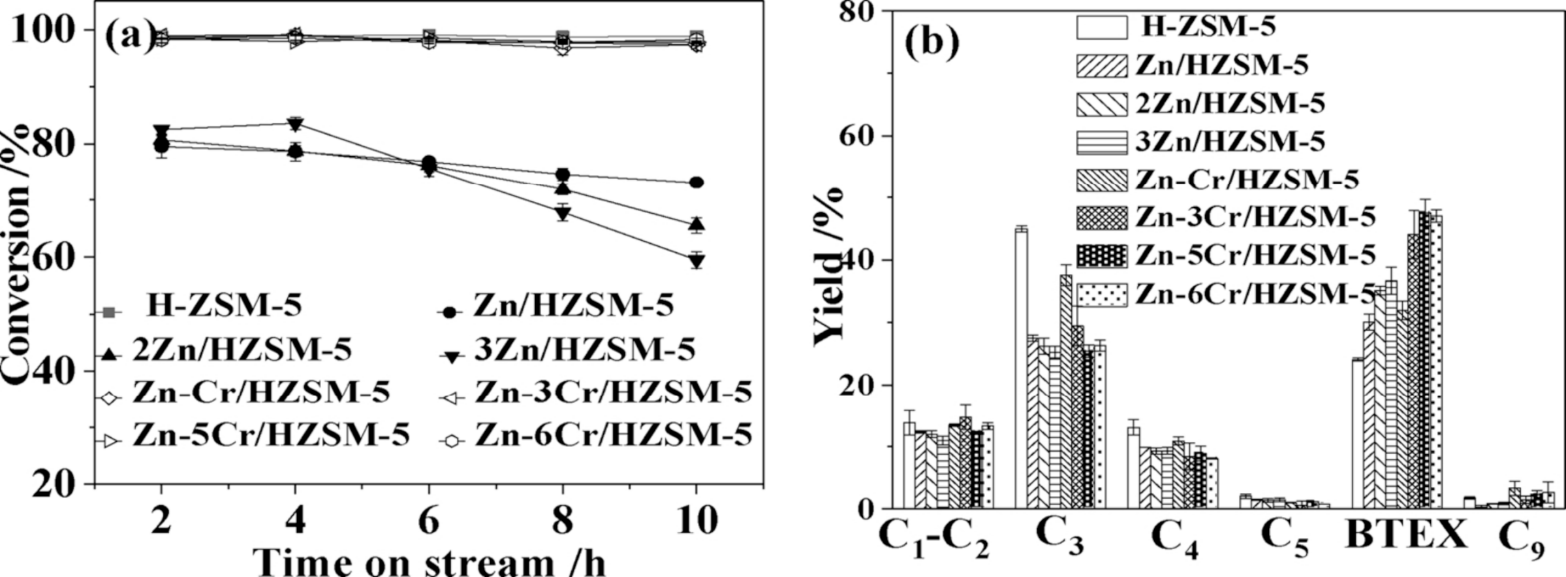
Conversion and product distribution of the CFP of *n*-hexane depending on type of catalyst. Reaction conditions: *n*-hexane feed, *T* = 470 °C, hourly gas space velocity = 2 h^–1^. Reproduced with permission from ref (247). Copyright 2020 Elsevier.

Doping H-ZSM-5 with a mixture of Zn and Cr was also recently reported (Table 15, entry 2).^247^ This study showed a decisive role of Cr on the activity of these catalysts for the conversion of hexane and its cofeeds with methanol. At a 470 °C (gas space velocity = 2 h^–1^) conversion of *n*-hexane is essentially close to 100% on all Cr/Zn doped samples, while at the same time the Zn loaded H-ZSM-5 catalysts showed *n*-hexane conversion between 80 and 70%. Interestingly the Zn/Cr-doped samples showed almost no deactivation with TOS which was visibly pronounced for the Zn-doped samples. Although the undoped H-ZSM-5 sampled showed a similar high conversion close to the Zn/Cr catalysts and rather high stability with TOS, this catalyst is much less selective to aromatics and BTX and has higher selectivity toward C3-C5 hydrocarbons (Figure 8). For the dehydroaromatization of a reaction feed of *n*-hexane:methanol (ratio =3:7) and at a temperature of 450 °C (gas space velocity = 2 h^–1^) the aromatic yield was 40.1%, with xylene selectivity of 40.4%. An increase of the methanol concentration in the feed gas resulted in an increase of the selectivity toward xylene.

Gui et al. studied ZnNi/H-ZSM-5 for the aromatization of *n*-hexane using both thermal and microwave-assisted heating.^251^ The aromatics yield, in particular the BTX yields, were higher when using the microwave-assisted heating when compared to the conventional thermal heating. Solymosi and Barthos also studied the aromatization of *n*-hexane and 1-hexene on supported and unsupported Mo_2_C catalyst.^252^ Silica supported Mo_2_C showed significantly higher activity compared to the unsupported carbide. Upon varying the reaction temperature between 450 °C and 550 °C, the conversion of *n*-hexane over the 10 wt% Mo_2_C/SiO_2_ catalyst varied between about 5% and 35%, while selectivity for benzene varied between 18 and 65%, depending on the temperature range. The maximum benzene selectivity (65%) on this best performing catalyst was observed at 500 °C, whereas the maximum conversion (35%) was observed at 600 °C. Based on their results it could be assumed that the production of benzene passes through the formation of hexene, and eventually its final dehydrogenation and cyclization to benzene.

Pt catalysts are thus far the most intensively studied catalysts for the aromatization of hexane.^253^ The reason for the comparatively intensive studies of Pt for *n*-hexane activation is related to the industrial use of Al_2_O_3_-supported Pt in the reforming of naphtha to convert heavy naphtha (C7-C10) into benzenoid aromatics.^254^ These catalysts are also bifunctional in nature, where the metal is necessary for the adsorption and dehydrogenation of the alkane and the acid sites (on Al_2_O_3_) are needed for oligomerization and cyclization toward aromatics. Tamm and co-workers at Chevron showed for the first time that Pt clusters encaged into the micropores of the L-type zeolites active for the aromatization of hexane as well as heptane with rather high selectivity toward aromatics.^255^ They named this catalyst AROMAX, which compared to non-acidic metal catalysts (e.g., Pt/carbon) showed significantly higher stability with time on stream. Structural features of these commercial catalysts are, however, not clearly reported. Davis and Derouane showed that Pt clusters supported on MgO, a basic oxide support, also showed good activity toward the aromatization of light naphtha including hexane (Table 15, entry 3).^248^ However, these catalysts show an initial decline in the catalytic activity and then remain stable with TOS. Interestingly, it can be concluded that basic sites can activate the cyclization of the dehydrogenated alkanes in a similar way as the acid sites.

Azzam et al. focused on mechanistic understanding of the dehydroaromatization of hexane on KL-zeolite supported Pt catalysts by employing kinetic isotope effect (KIE) measurements.^256^ Running the reaction using a gas mixture containing equimolar amounts of *n*-C_6_H_14_ and *n*-C_6_D_14_ on Pt/KL catalyst at 500 °C resulted in almost no KIE, i.e., the rates of hydro-cyclization of C_6_H_14_ and C_6_D_14_ are equal. This means that the dehydrogenation step cannot be considered as rate-limiting step. Instead, these authors concluded that the entry into the pores (diffusion) of hexane is the rate determining step.

**Figure 9:**
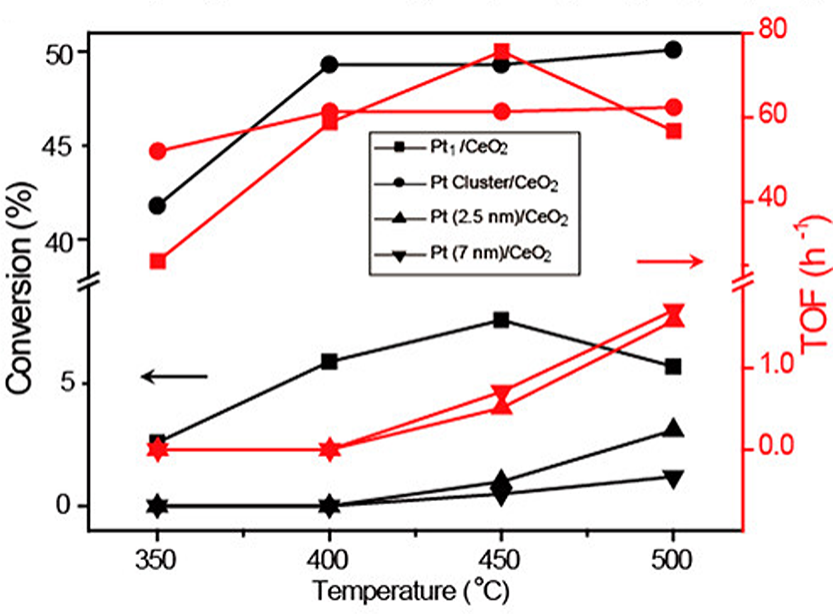
Temperature dependence of the conversion of *n*-hexane on Pt catalysts having different Pt particle sizes (in addition to Pt single-atom catalyst) and corresponding turnover frequency rates. Reproduced with permission from ref (257). Copyright 2020 American Chemical Society.

Recently, Zhang et al. studied the potential of heterogeneous Pt single-atom-based catalysts for the dehydroaromatization of *n*-hexane to benzene and compared their behavior to nanoparticle or Pt-clusters supported on CeO_2_ in the temperature range from 350 to 500 °C.^257^ These authors found a monotonic increase in the rate of conversion of *n*-hexane upon going from Pt nanoparticles to smaller clusters and eventually to the atomic dispersion level (i.e., Pt single-atom c) (see results in Figure 9). At 500 °C where selectivity to aromatics is highest, the single-atom Pt/CeO_2_ catalyst is about one order of magnitude more active than the nanoparticle-based Pt/CeO_2_ catalysts. This comparison is based on both total conversion and also the turnover frequencies (see results in Figure 9).

It was recently reported that a metal-free catalyst, made by the grafting of activated carbon using phosphorous compounds, can achieve relatively high activity and benzene-selectivity.^258^ At a temperature of 525 °C these catalysts achieved up to 90% conversion with a selectivity for benzene slightly higher than 90% after 15 min of starting the reaction. This catalyst showed, however, deactivation with time on stream for hexane conversion, decaying in 300 min from 90 to about 45% hexane conversion. The selectivity for benzene decayed also after 300 min to about 40%. Loss of selectivity of benzene is correlated with increase of selectivity toward olefins, paraffins and other C6 hydrocarbons (non-benzenoids).

In view of the relatively high selectivities obtained in the CFP of hexane we may conclude that this is a methodology that would merit further research toward large scale implementation.

### 2.5 Aromatics from Bio-derived Furans

**Figure 10:**
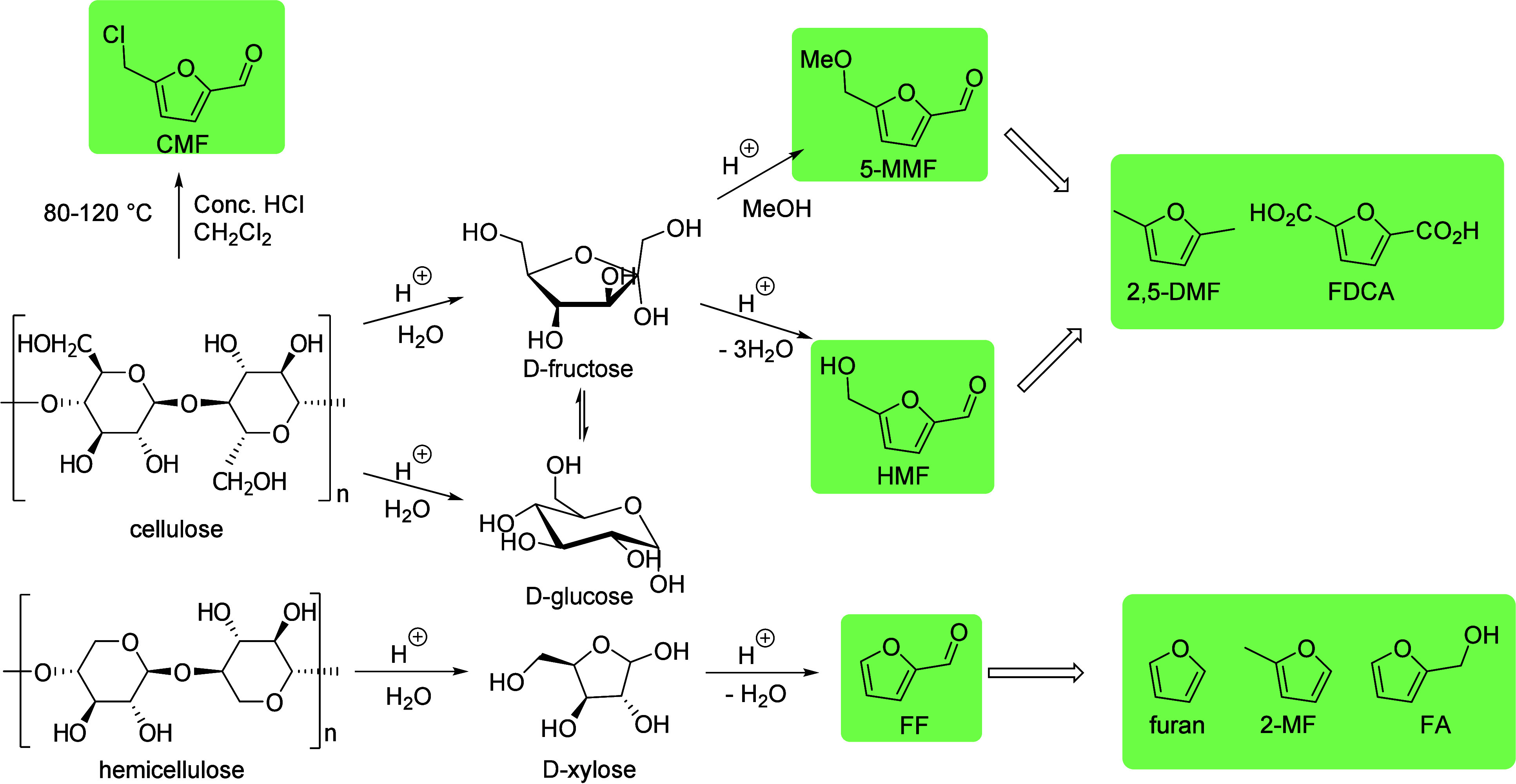
Sugar-derived furan platform chemicals.

One main approach to utilize carbohydrates is through their dehydrated products (Figure 10). Dehydration of fructose or glucose under acidic conditions produces 5-hydroxymethylfurfural (HMF), while dehydration of pentose led to the formation of furfural (FF). Bozell has listed HMF and FF as “top 10+4” chemicals originated from sugars.^159^ Although many methods have been reported to produce HMF from sugars,^6,259^ HMF itself is rather unstable, which makes it hard to isolate it pure in high yields. In addition, the required high dilution or the use of buffer solutions increases the cost and inhibit its actual industrial production. In 2014, AVA Biochem started a plant in which HMF is produced on ton-scale.^260,261^ In their process, HMF is a side product of the production of biochar from sugars. They announced 400 ton production per year and expect to reach 5000–10000 tonnes per year in the near future.^260,261^ The price of HMF is thus still rather high. As a consequence, HMF analogues with better stability have attracted more attention, such as 5-methoxymethylfurfural (5-MMF) and 5-chloromethylfurfural (CMF) (Figure 10).^262^ Dehydration of fructose in methanol forms 5-MMF, which is oxidized to 2,5-furandicarboxylic acid (FDCA). The Dutch company Avantium is constructing the first FDCA plant in Delfzijl, the Netherlands, and are expected to start operation in 2024 with a predicted 5000 tonnes production per year.^263^ Mascal and co-workers developed a process to dehydrate cellulose or even lignocellulose to CMF in >80% yield using concentrated HCl and a chlorinated solvent.^262,264^ Origin Materials will bring this process to large-scale production. They built a plant in Ontario, Canada, which has started production in June 2023 and has an estimated capacity to process 25000 dry metric tons of biomass.^265^ The second plant in Geismar, Louisiana is planned to open in 2025 and has a capacity of 1 million tons.^266^ They will use CMF for the production of xylene and terephthalic acid.

Unlike HMF, the industrial production of FF from biomass, such as empty corn cobs, has been well developed, and is also economically preferred over fossil-based processes.^6^ The estimated FF production in the world is around 450 000 tons per year, most of which is produced in China.^130^ More than 60% of FF is used to produce furfuryl alcohol (FA) through hydrogenation.^6,267^ FA is used for the production of furan resins, which find application as foundry binders.

Hydrogenolysis of HMF or FF leads to the formation of 2,5-dimethylfuran (2,5-DMF) or 2-methylfuran (2-MF) respectively, while palladium-catalyzed decarbonylation of FF produces furan.^268,269^ The latter has been an industrial process as Dupont has used furan as raw material for the production of adiponitrile in their first process for this nylon intermediate. However, the process was abandoned when a new process based on butadiene was developed. Furan, 2-MF, and 2,5-DMF are recognized as potential bio-based fuels.^268,269^ Moreover, furan also serves as solvent.

In this section, we summarize the research on the syntheses of bio-based aromatics from furans through three approaches: (a) catalytic pyrolysis of furans; (b) Diels–Alder/dehydration of furans with dienophiles; and c) aldol-condensation reactions.

#### 2.5.1 Aromatics from Catalytic Pyrolysis of Furans

The earliest reports on the catalytic pyrolysis of furans can be traced back to the 1990s, when Schulz-Ekloff and Chantal detected trace amounts of BTX and benzofuran in the liquid oil obtained from pyrolysis of furan and furfural (FF) at 300–450 °C using H-ZSM-5 or Pt-ZSM-5 as catalysts.^270,271^ After that, pyrolysis of furans did not gain much attention until 2008, when Huber realized the key roles of furans as intermediates in aromatics formation during pyrolysis of glucose (section 2.2).^17,20,27^ Since then, catalytic pyrolysis of furans attracted extensive attention, especially as model reaction for lignocellulose pyrolysis. Tables 16–18 summarize the published reports with more than 20% aromatics yields on the CFP of furans and furfural (FF).

**Table 16:** Zeolite-Catalyzed Pyrolysis of Furan to Aromatics—Products Analyzed by GC and GC-MS

										Aromatic distribution (selectivity) (%)			
Entry	Cat. (Si/Al ratio)	WHSV (h^–1^)	Carrier gas	*P*_furan_ (Torr)	*T* (°C)	TOS	Furan conv. (%)	Aromatic sel. (%)	Aromatic carbon yield (%)	Benzene	Toluene	Xylene	Ref
1	H-ZSM-5 (15)	21.86	air	6	600	4.5 min	28	32.1	9.0	22.1	19.9	4.5	(272)
2	H-ZSM-5 (15)	10.35	air	6	600	4.5 min	48	31.01	14.9	25.9	23.6	4.4	(272)
3	H-ZSM-5 (15)	1.95	air	6	600	4.5 min	97	31.42	30.5	36.9	34.0	8.7	(272)
4	H-ZSM-5 (15)	10.4	air	6	450	4.5 min	22	37.7	8.3	9.5	11.1	4.0	(272)
5	H-ZSM-5 (15)	10.4	air	6	500	4.5 min	32	27.3	8.7	17.9	19.8	4.8	(272)
6	H-ZSM-5 (15)	10.4	air	6	650	4.5 min	60	26.8	16.1	35.1	24.6	3.4	(272)
7	Mic ZSM-5 (15)	10.4	He	6	600	270 s	35.9	44.7	16.1	21.0	18.6	8.8^b^	(22)
8	Mic ZSM-5^a^ (15)	10.4	He	6	600	270 s	40.3	40.5	16.3	20.7	18.1	8.1^b^	(22)
9	Mes ZSM-5 (15)	10.4	He	6	600	270 s	36.3	35.8	13.0	18.3	17.7	8.7^b^	(22)
10	Mes ZSM-5^a^ (15)	10.4	He	6	600	270 s	29.5	37.0	10.9	17.8	18.2	8.7^b^	(22)
11	Meso ZSM-5 (30)	9.5	He	5.5	600	3 min	27.3	40.9	11.2	28.0	26.3	2.4	(274)
12	100 nm ZSM-5 (27)	6.2	He	5.5	600	3 min	27.3	36.3	9.9	28.9	24.6	2.8	(274)
13	800 nm ZSM-5 (28)	3.2	He	5.5	600	3 min	23.8	26.9	6.4	27.6	23.2	2.5	(274)
14	ZSM-5 (15)	10.4	He	6	600	30 min	48	31.0	14.9	25.9	23.6	4.3	(275)
15	Ga/H-ZSM-5^c^ (15)	10.4	He	6	600	30 min	53	37.8	20.0	38.8	21.2	3.1	(275)
16	Ga/H-ZSM-5^d^ (15)	10.4	He	6	600	30 min	50	39.7	19.9	35.6	17.5	1.9	(275)
17	Ga/H-ZSM-5^e^ (15)	10.4	He	6	600	30 min	47	43.5	20.5	33.7	15.1	1.5	(275)
18	ZSM-5 (15)	9.3	He	6	600	4.5 min	46.1	20.8	7.9	40.4	34.8	3.3	(276)
19	0.5 wt% Zn/ZSM-5 (15)	9.3	He	6	600	4.5 min	49.8	23.9	10.8	46.8	21.1	4.3	(276)
20	1.0 wt% Zn/ZSM-5 (15)	9.3	He	6	600	4.5 min	66.9	31.0	16.8	67.0	14.6	5.0	(276)
21	2.0 wt% Zn/ZSM-5 (15)	9.3	He	6	600	4.5 min	53.0	29.8	13.2	64.1	14.7	4.2	(276)
22	5.0 wt% Zn/ZSM-5 (15)	9.3	He	6	600	4.5 min	69.5	37.1	19.6	73.9	12.3	3.0	(276)
23	2.0 wt% Ga/ZSM-5 (15)	9.3	He	6	600	4.5 min	52.5	26.7	11.0	54.7	26.0	2.0	(276)
24	5.0 wt% Ga/ZSM-5 (15)	9.3	He	6	600	4.5 min	46.5	28.7	12.1	48.5	23.3	2.2	(276)
25	H-ZSM-5 (13)	0.05	Ar	1.5	450	–	–	39.7	–	25.9	25.7	5.8	(277)
26	4 wt% Ga/H-ZSM-5 (13)	0.05	Ar	1.5	450	–	–	49.8	–	34.5	25.1	3.2	(277)

a Catalysts were treated with tartaric acid.

b Includes ethylbenzene, styrene.

c ion-exchanged ZSM-5.

d incipient-wetness ZSM-5.

e modified ion-exchange ZSM-5.

##### 2.5.1.1 BTX and Other Alkylated Benzenes from Furans

In 2011, Huber investigated the H-ZSM-5-catalyzed pyrolysis of furan and studied the effects of space velocity and temperatures on aromatics formation (Table 16, entries 1–6).^272^ Lower space velocity (from 21.86 to 1.95) at 600 °C enhanced furan conversion (from 28% to 97%), increased benzene selectivity (from 22.1% to 36.9%) and toluene selectivity (from 19.9% to 34.0%). Benzofuran and coke were the main products at 450 °C. At medium temperatures (500–600 °C), the selectivity toward benzene and toluene were higher. An even higher temperature (650 °C) enhanced the formation of olefins and CO.^272^ The catalyst deactivated rapidly, due to coking. After 30 min TOS, only coke was formed.^272^ Mesoporous (Mes) ZSM-5-catalyzed CFP of furan resulted in slightly lower selectivity (35.8%) of aromatics than microporous (Mic) ZSM-5 (44.7%) at 600 °C, due to coke formation in the mesopores (Table 16, entries 7–10).^22^ Microporous ZSM-5-catalyzed furan pyrolysis favored the formation of small monoaromatics (BTX), while mesoporous ZSM-5 showed higher selectivity to larger alkylated monoaromatics.^22^ Surface dealumination of both mesoporous and microporous ZSM-5 with a tartaric acid solution had limited effect on aromatics production.^22^ Increased H_2_O pressure on ZSM-5-catalyzed furan pyrolysis at 600 °C significantly improved furan conversion (from 44% to 85%), but continuously decreased aromatic selectivity (from 8.1% to 5.2%), which was ascribed to the enhanced formation of CO_2_ and propylene from furan hydrolysis.^273^

Fan and Gou prepared ZSM-5 catalysts with various morphologies and crystallite sizes (100 nm and 800 nm) and tested them for the CFP of furan at 600 °C (Table 16, entries 11–13).^274^ Increased mesoporosity and decreased crystallite size both facilitated furan conversion (27.3% with mesoporous ZSM-5 or 100 nm ZSM-5, 23.8% using 800 nm ZSM-5) and improved aromatic selectivity.^274^ The reduced aromatics yield found upon use of 800 nm ZSM-5 as catalyst was due to coke formation inside the micropores.^274^

To improve the aromatics yield in these reactions, metal-doped zeolites were investigated. In 2012, Huber synthesized Ga-promoted H-ZSM-5 catalysts which were tested in the CFP of furan at 600 °C (Table 16, entries 14–17).^275^ Ion-exchanged and incipient-wetness Ga/H-ZSM-5 showed similar furan conversions as H-ZSM-5 (47–53%), but higher aromatic selectivity (37.8–43.5%), compared to H-ZSM-5 (31%).^275^ However, H-ZSM-5 with Ga inside the framework, synthesized by a hydrothermal method, reduced both furan conversion and aromatic selectivity, due to the lack of Brønsted acid sites.^275^ The group also investigated the use of Zn-promoted ZSM-5 as catalyst in the CFP of furan at 600 °C and compared the results with those obtained with the Ga/ZSM-5 catalyst (Table 16, entries 18–22).^276^ The addition of Zn or Ga to ZSM-5 both enhanced furan conversion to 69.5% and 52.5% respectively, and increased the aromatics formation with a maximum 19.6% yield using 5.0 wt% Zn-promoted ZSM-5 catalyst.^276^ Meanwhile, the selectivity to benzene was improved to 73.9%.^276^

Hensen and co-workers prepared H-ZSM-5 containing 1–4 wt% Ga, which was used as catalyst in the CFP of furan (Table 16, entries 25–26) and 2,5-dimethylfuran (2,5-DMF) at 450 °C with low WHSV (0.4 or 0.05) (Table 17). Olefin yields increased rapidly after catalyst deactivation, indicating that olefins were generated in the earlier stages and were intermediates for aromatics formation. Increased Ga content improved aromatics formation, especially BTX and reduced olefins formation, suggesting that olefins were converted to aromatics by Ga species. At lower temperatures (<300 °C), no gaseous products were observed with full 2,5-DMF (2,5-dimethylfuran) conversion catalyzed by 4 wt% Ga/H-ZSM-5, which was ascribed to the oligomerization of 2,5-DMF in the zeolite micropores. At 300 °C, BTX started to appear; the yields of which maximized at 375–525 °C. At higher temperatures (>525 °C), aromatic yields were decreased because of coke formation. The improved aromatics selectivity obtained with Ga/H-ZSM-5 was ascribed to the substitution of Lewis acid sites for Brønsted acid sides, which may inhibit cracking processes.^277^ As biomass may contain alkali metals, such as potassium, the use of ZSM-5 tainted with different potassium sources was tested as catalysts at 600 °C. The addition of potassium decreased both 2-methylfuran (2-MF) conversion and aromatic yields (≤17.9 wt%), compared to the parent ZSM-5, due to the inhibition of the decarbonylation of 2-MF.^278^

**Table 17:** H-ZSM-5 or Ga-Promoted H-ZSM-5-Catalyzed Pyrolysis of 2-MF and 2,5-DMF^a^^277^

				Aromatics distribution (selectivity) (%)		
Entry	Feed	Catalyst (Si/Al ratio)	Aromatics Sel. (%)	Benzene	Toluene	Xylene
1	2-MF	H-ZSM-5 (13)	45.2	19.7	28.5	13.3
2	2-MF	4 wt% Ga/H-ZSM-5 (13)	54.9	29.1	24.6	6.0
3	2,5-DMF	H-ZSM-5 (13)	46.7	23.3	32.5	10.1
4	2,5-DMF	1 wt% Ga/H-ZSM-5 (13)	45.3	39.7	28.3	4.9
5	2,5-DMF	2 wt% Ga/H-ZSM-5 (13)	51.4	44.7	26.5	4.1
6	2,5-DMF	4 wt% Ga/H-ZSM-5 (13)	52.7	46.5	24.7	4.0

a Conditions: WHSV = 0.05 h^–1^; Ar as carrier gas; furan pressure = 1.5 Torr at 450 °C. Product distribution was analyzed by GC-MS.

Furfural has been mostly employed as model chemical to study biomass pyrolysis. In 2017, Lin investigated the use of H-ZSM-5, 0.5 wt% Zn/H-ZSM-5, and 1.5 wt% Zn/H-ZSM-5 in the CFP of furfural and studied the effects of temperature as well as space velocity on product distribution (Table 18, entries 1 and 2).^279^ H-ZSM-5 as catalyst provided a 15.7% carbon yield of aromatics at 500 °C with high toluene distribution (62.2% selectivity).^279^ Addition of 0.5 wt% and 1.5 wt% Zn to H-ZSM-5 raised the aromatic carbon yields to 20.7% and 26.3% at 500 °C and altered the selectivity to benzene to 70.5% and 71.6%, respectively.^279^ Increased space velocity reduced aromatics formation using either H-ZSM-5 or Zn/H-ZSM-5 as catalysts.^279^

**Table 18:** Catalytic Fast Pyrolysis of Furfural (FF) to Aromatics^a^

											Aromatics distribution (selectivity) (%)			
Entry	Feed	Catalyst (Si/Al ratio)	GHSV (h^–1^)	Carrier gas	*P*_furfural_ (Torr)	*T* (°C)	TOS (min)	Conv. (%)	Aromatics Sel. (%)	Aromatics carbon yield (%)	Benzene	Toluene	Xylene	Ref
1	FF	0.5 wt% Zn/ H-ZSM-5 (40)	2412	air	5.6	500	13	100	20.7	20.7	70.5	26.5	0	(279)
2	FF	1.5 wt% Zn/ H-ZSM-5 (40)	2412	air	5.6	500	13	100	26.3	26.3	71.6	25.0	0	(279)
3	FF	H-ZSM-5 (23)	0.5^b^	N_2_^d^	–	500	–	100 wt	50.3 wt	50.3 wt	10.0	16.8	19.6	(281)
4	FF	0.3 H-ZSM-5 (23)^c^	0.5^b^	N_2_^d^	–	500	–	100 wt	34.4 wt	34.4 wt	10.2	14.1	16.5	(281)
5	FF	0.5 H-ZSM-5 (23)^c^	0.5^b^	N_2_^d^	–	500	–	100 wt	35.2 wt	35.2 wt	16.1	7.4	22.8	(281)
6	FF	0.7 H-ZSM-5 (23)^c^	0.5^b^	N_2_^d^	–	500	–	100 wt	45.9 wt	45. 9 wt	12.6	17.7	20.7	(281)
7	FF	0.9 H-ZSM-5 (23)^c^	0.5^b^	N_2_^d^	–	500	–	100 wt	53.9 wt	53.9 wt	23.6	20.6	16.8	(281)
8	FF	1.1 H-ZSM-5 (23)^c^	0.5^b^	N_2_^d^	–	500	–	100 wt	45.7 wt	45.7 wt	18.6	22.8	18.9	(281)
9	FF	0.5 wt% Mg–1 wt% Cu/BEA (100)	1.2^f^	Ar	–	700–750	60	100	∼45	∼45	100	0	0	(282)
10	FF	H-ZSM-5 (15)^e^	–	He	–	300, 600	–	–	–	35.1	24.2	30.2	8.7	(23)
11	HMF	H-ZSM-5^e^	–	He	–	300, 600	–	–	–	25.5	24.8	29.9	11.5	(23)

a Products were analyzed by GC and GC-MS (FID).

b Feeding rate (mL/h).

c Pretreated with HCl (the number in front indicates the concentration).

d N_2_ (10 mL/min).

e In a tandem microreactor.

f WHSV.

Wang and co-workers studied the effects of mesopores on the CFP of furfural at 550 °C.^280^ Compared to microporous ZSM-5, the use of mesoporous ZSM-5 as catalyst improved the selectivity to benzene and toluene by 45.2% and 55.3% respectively and decreased the naphthalene selectivity by 72.1%, while the total aromatics yield did not change (around 10%).^280^

Zhao and co-workers evaluated the effects of temperature, gas flow, feeding rate, and Si/Al ratios on aromatics formation from the H-ZSM-5-catalyzed pyrolysis of furfural (Table 18, entries 3–8).^281^ The optimum conditions were 10 mL/min N_2_ flow and 0.5 mL/h feed rate with H-ZSM-5 in Si/Al ratio 23 as catalyst, producing 58.5 wt% aromatics yield.^281^ Pretreatment of H-ZSM-5 with a 0.3–1.1 mol/L of HCl solution reduced the aromatics yield to 34.4–45.9 wt%, except 0.9 mol/L HCl, which slightly improved the aromatics yield to 53.9 wt%.^281^ In addition, the use of HCl-pretreated H-ZSM-5 as catalyst decreased the selectivity to naphthalene in the CFP of furfural, compared to pure H-ZSM-5.^281^

Guan and co-workers prepared a bifunctional Mg-Cu loaded BEA zeolite for furfural pyrolysis, providing >40% carbon yield of aromatics with high selectivity to monoaromatics at 600 °C (Table 18, entry 9).^282^ The addition of Cu-species to the zeolite created Lewis acidic sites and thus facilitated the deoxygenation of furan intermediates and their aromatization.^282^ Mg species prohibited the polymerization, resulting in high selectivity to monoaromatics. Using 0.5 wt% Mg–1 wt% Cu on β-zeolite as catalyst, only benzene was produced as the sole liquid product at temperatures >700 °C. This catalyst showed high stability for 10 recycles.^282^

Brown and co-workers reported a tandem microreactor, where furfural was pyrolyzed in the first reactor and the resulting liquid vapor entered into the second reactor equipped with a catalyst bed (Figure 1).^23^ Furfural was first pyrolyzed at 300 °C and the produced liquid vapor was pyrolyzed at 600 °C with H-ZSM-5 as catalyst in the second reactor, affording a 35.1% carbon yield of aromatics (Table 18, entries 10 and 11).^23^ HMF treated under the same conditions delivered a lower aromatic carbon yield (25.5%), due to coke formation.^23^ An isotopic study of the HMF conversion suggested that decarbonylation of HMF to small furan molecules was essential for entering into H-ZSM-5 for the following transformations but also resulted in high coke formation.^23^

##### 2.5.1.2 Catalytic Fast Pyrolysis of Furans with Co-feeds

Helium, nitrogen, and air are frequently used as carrier gas for the CFP of biomass materials. When gaseous carbon-containing chemicals were added to the carrier gas, the product distributions were significantly altered. In 2012, Huber compared the product distribution of ZSM-5-catalyzed pyrolysis of furans with or without olefins as co-feed (Table 19).^283^ Use of 2% propylene as co-feed in the CFP of furan at 600 °C enhanced both furan conversion and aromatic selectivity, and led to an altered aromatic distribution, wherein toluene selectivity increased from 25.1% to 58.6% and benzene selectivity decreased from 27.5% to 15.3%.^283^ Reduced temperature (from 600 °C to 450 °C) decreased toluene selectivity from 58.6% to 38.9%, suggesting that the DA reaction of furan was less favored at lower temperature (Table 19, entries 1–6).

**Table 19:** Effects of Co-feeds (Propylene and Ethylene) on Catalytic Fast Pyrolysis of Furans over ZSM-5^a^^,^^283^

			WHSV (h^–1^)				Conv. (%)			Aromatics distribution (%)		
Entry	Feed	Propylene (in He)	Furan	Olefin	*P*_furan_ (Torr)	*T* (°C)	Furan	Olefin	Aromatics sel. (%)	Benzene	Toluene	Xylene
1	Furan	–	5.9	–	6.0	600	64	–	42.6	24.3	21.8	3.9
2	Furan	–	10.4	–	6.0	600	48	–	29.2	27.5	25.1	4.6
3	Furan	1.92%^b^	10.4	11.1	6.0	600	50	1	40.2	28.4	30.1	7.9
4	Furan	1.94%	10.4	15.6	6.0	600	65	16	53.8	15.3	58.6	13.6
5	Furan	2%	10.4	16.0	6.0	550	59	25	65.6	8.4	53.7	18.3
6	Furan	2%	10.4	16.0	6.0	450	54	22	60.2	5.1	38.9	35.1

7	2-MF	–	5.7	–	4.9	600	98	–	47.3	23.8	24.5	9.2
8	2-MF	2%	5.7	9.1	4.9	600	99	31	59.6	24.4	28.6	26.9
9	2-MF	2%	5.7	9.1	4.9	450	92	42	66.1	6.8	17.5	48.9
10	2-MF	2%	5.7	2.9	4.9	450	86	43	71.1	6.9	17.1	46.5
11	2-MF	2%	5.7	1.0	4.9	450	79	45	60.4	7.4	17.3	35.7

12	FF	–	9.0	–	7.0	600	100	–	16.7	35.5	28.6	6.9
13	FF	2%	9.0	9.1	7.0	600	100	53	42.7	21.0	57.9	14.2
14	FF	2%	9.0	9.1	7.0	450	100	64	38.6	6.1	38.5	38.5
15	FA	–	3.3	9.1	2.5	600	100	–	42.4	9.1	13.1	13.3
16	FA	2%	3.3	9.1	2.5	600	100	29	34.4	23.4	38.2	21.7

a Continuous-flow fixed-bed reactor; ZSM-5 (Si/Al = 15) as catalyst; reaction time: 45 s. Products were analyzed by GC-MS and GC-FID-TCD.

b Co-feeding with ethylene.

Addition of propylene as co-feed for the CFP of 2-methylfuran (2-MF) at 600 °C increased aromatic selectivity from 47.3% to 59.6% and xylene selectivity from 9.2% to 26.9% due to the DA reaction.^283^ At 450 °C, the CFP of 2-MF with propylene resulted in 66.8% selectivity toward aromatics with 48.9% of xylene. Lower temperatures reduced 2-MF conversion, propylene conversion, and aromatic formation, revealing an optimum temperature range for the DA product from CFP of 2-MF at 450–600 °C (Table 19, entries 7–11).

The CFP of furfural (FF) with 2% propylene as co-feed raised aromatic selectivity from 16.7% to 42.7% and especially increased toluene selectivity from 28.6% to 57.9% due to the decarbonylation of furfural to furan before its DA reaction to propylene.^283^ Co-feeding of furfuryl alcohol (FA) with propylene slightly decreased the total aromatic selectivity from 42.4% to 34.4%, but increased the selectivity toward benzene, toluene, and xylene, especially toluene (from 13.1% to 38.2%), suggesting that FA was also first decomposed to furan before its reaction with propylene (Table 19, entries 15–16).^283^ This pioneering work revealed the importance of DA reactions for aromatics formation in the CFP of lignocellulosic biomass.^283^

Hensen and Weckhuysen reported the Ga-promoted H-ZSM-5-catalyzed pyrolysis of 2,5-DMF with ethylene as co-feed at 450 °C and produced around 50% BTXE (benzene, toluene, xylenes and ethylbenzene) selectivity with full conversion.^284^ An increase of the Ga-loading improved both benzene and toluene selectivity. Regeneration experiments up to 10 cycles highlighted the high stability of Ga-H-ZSM-5 for catalyzing this transformation.^284^

Methanol has also been used as co-feed for the catalytic pyrolysis of furans (Table 20). In 2014, Zhao and co-workers studied methanol as co-feed for the H-ZSM-5-catalyzed pyrolysis of 2-MF at 550 °C and observed enhanced furan conversion (48.3–96.5% vs 39.8%) and improved aromatic selectivity (31.0–50.8% vs 19.8%) along with increased xylene selectivity and reduced benzene selectivity (Table 20, entries 1–5).^285^ Investigation of methanol/2-MF ratios, temperature, and WHSV maximized the aromatic selectivity to 56.7% with full 2-MF conversion at a MeOH/2-MF ratio of 2 at 550 °C. 2,5-DMF (Table 20, entries 6 and 7), FF (Table 20, entry 8), and FA (Table 20, entry 9) were also pyrolyzed with methanol as co-feed at 550 °C and delivered 55.0%, 29.4%, and 24.2% yields of aromatics, respectively.^285^

**Table 20:** Methanol as Co-feed for Catalytic Pyrolysis of Furans^a^

							Conv. (%)				Aromatics distribution (%)			
Entry	Feed	MeOH/feed ratio	Catalyst (Si/Al ratio)	Furan WHSV (h^–1^)	*T* (°C)	Reaction time (min)	Furan	MeOH	Aromatics sel. (%)	Aromatics carbon yield (%)	Benzene	Toluene	Xylene	Ref
1	2-MF	–	ZSM-5 (25)^b^	6	550	15	39.8	–	19.8	7.9	35.4	46.9	15.9	(285)
2	2-MF	1	ZSM-5 (25)^b^	6	550	15	48.3	100	31.0	15.0	20.9	49.1	25.6	(285)
3	2-MF	2	ZSM-5 (25)^b^	6	550	15	94.8	100	50.8	48.2	22.5	47.5	27.5	(285)
4	2-MF	3	ZSM-5 (25)^b^	6	550	15	96.5	100	37.8	36.5	22.9	47.1	25.9	(285)
5	2-MF	2	ZSM-5 (25)^b^	1	550	15	100	100	56.7	56.7	24.2	49.9	23.0	(285)
6	2,5-DMF	2	ZSM-5 (25)^b^	6	550	15	100	100	55.0	55.0	20.3	46.2	29.7	(285)
7	2,5-DMF	2	ZSM-5 (25)^b^	6	450	15	76.2	100	48.6	37.0	14.6	37.7	38.7	(285)
8	FF	2	ZSM-5 (25)^b^	6	550	15	100	100	29.4	29.4	31.9	48.5	17.8	(285)
9	FA	2	ZSM-5 (25)^b^	6	550	15	100	100	24.2	24.2	24.6	44.1	28.7	(285)
10	2-MF	5	H-ZSM-5 (25)	2	500	60	100	94.6	43.5	43.5	8.1	13.2	23.6	(286)
11	2-MF	–	H-ZSM-5 (25)	2	500	60	64.7	–	20.5	13.3	9.2	6.6	9.9	(286)
12	2-MF	2	H-ZSM-5 (25)	2	500	60	90.8	88.0	31.8	28.9	9.3	9.4	17.6	(286)
13	2-MF	3	H-ZSM-5 (25)	2	500	60	96.5	92.4	40.0	38.6	8.8	12.0	26.0	(286)
14	2-MF	10	H-ZSM-5 (25)	2	500	60	100	94.9	39.6	39.6	5.3	10.2	38.7	(286)
15	2,5-DMF	5	H-ZSM-5 (25)	2	500	60	100	94.7	41.4	41.4	8.8	11.7	30.0	(286)
16	FF	5	H-ZSM-5 (25)	2	500	60	100	94.1	29.0	29.0	10.3	12.5	25.3	(286)
17	FA	5	H-ZSM-5 (25)	2	500	60	100	94.2	27.5	27.5	8.1	10.0	29.5	(286)
18	2-MF	2	15% La/10% Ce/ZSM-5 (80)	1	400	–	–	–	–	25.6	6.2	23.3	42.4	(287)
19	2-MF	2	15% La/10% Ce/ZSM-5 (80)	1	450	–	–	–	–	45.2	6.5	26.8	41.7	(287)
20	2-MF	2	15% La/10% Ce/ZSM-5 (80)	1	500	–	–	–	–	47.6	8.1	33.8	37.8	(287)
21	2-MF	2	15% La/10% Ce/ZSM-5 (80)	1	550	–	–	–	–	40.7	12.3	41.8	34.7	(287)

a Reactions were performed in a fixed-bed reactor with N_2_ as carrier gas; products were analyzed by GC-FID-TCD and GC-MS.

b Ar as carrier gas.

In 2019, Ma reported the H-ZSM-5-catalyzed pyrolysis of 2-MF with methanol as co-feed in a self-designed quartz tube reactor (Table 20, entries 10–14).^286^ At 500 °C, co-feeding with methanol enhanced the 2-MF conversion from 64.7% to 90.8–100% and increased aromatic selectivity from 20.5% to 31.8–43.5% using H-ZSM-5 as catalyst. Increased methanol ratios improved xylenes selectivity and slightly decreased selectivity to benzene. Unlike Zhao’s observation,^285^ the selectivity to toluene was also increased from 6.6% to 9.4–12.0% with methanol as co-feed. Studies on 2-MF pyrolysis at different temperatures, WHSV, MeOH/2-MF ratios, and Si/Al ratios maximized the aromatic selectivity to 45.1% with full 2-MF conversion. Co-feeding with methanol, H-ZSM-5-catalyzed pyrolysis of 2,5-DMF (Table 20, entry 15), FF (Table 20, entry 16), and FA (Table 20, entry 17) at 500 °C produced 41.4%, 29.0%, and 27.5% aromatic selectivity, respectively, with full conversion.^286^

A bimetallic-modified catalyst 15% La/10% Ce/H-ZSM-5 was prepared and studied by Li and co-workers for 2-MF pyrolysis at 450 °C (Table 20, entries 18–21).^287^ With methanol as co-feed (molar ratio to 2-MF is 2), a 45.2% yield of aromatics was obtained and *p*-xylene was generated in 17.8% yield with 94.5% *p*-xylene/xylene ratio. In the absence of MeOH, only 24.9% yield of aromatics was formed with 7.8% yield of *p*-xylene (90.1% *p-*xylene/xylene ratio).^287^ Pyrolysis of furan, 2,5-DMF, and FF with 15%La/10%Ce/H-ZSM-5 as catalyst co-feeding with MeOH at 450 °C resulted in 12.4–22.5% yields of *p*-xylene (94.9–95.6% *p-*xylene/xylene ratios).^287^ The high selectivity to *p*-xylene was attributed to the micropores inside the modified zeolite, which suppressed the isomerization of *p*-xylene to *m*-xylene and *o*-xylene (*d*_c_ = 7.437 and 7.345 Å respectively).^287^ Moreover, this modified catalyst possesses very low external acidic sites, preventing the isomerization of *p*-xylene to *m*-xylene or *o*-xylene.^287^

Methane is the main composition of natural gas, which was employed by Song as co-feed for the CFP of furfural in a batch reactor.^288^ At 400 °C, a 76.7% carbon yield of aromatics was obtained with a 52.7% carbon yield of BTX under 25 bar methane catalyzed by a Zn-Ga-modified H-ZSM-5 (Si/Al = 140).^288^ The absence of methane resulted in a reduced aromatics yield (54.3%) and a lower BTX yield (38.6%) under same conditions.^288^ With the parent H-ZSM-5 as catalyst, 56.7% and 51.2% yield of aromatics under 25 bar methane or N_2_ were produced, respectively.^288^ The improved catalytic behavior of Zn-Ga/ZSM-5 for aromatics formation was attributed to its high dispersion of Zn and Ga species on ZSM-5, the proper weak and strong acidic sites, and the stable metal species.^288^

**Figure 11:**
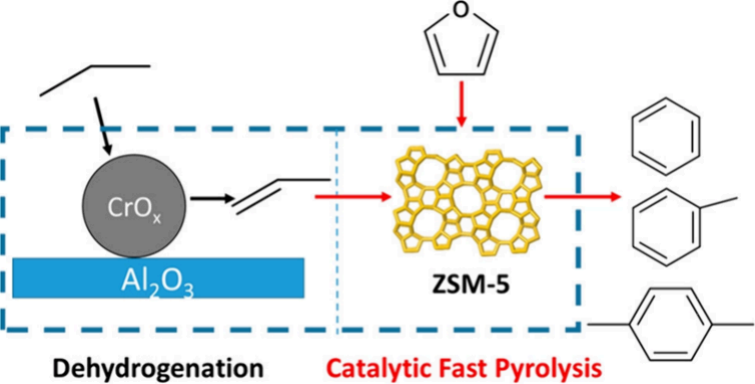
CrO_*x*_/Al_2_O_3__ZSM-5-catalyzed furan pyrolysis co-feeding with propane. Reproduced with permission from ref (289). Copyright 2019 American Chemical Society.

Around 5% of natural gas is composed of propane.^289^ Fan designed a bifunctional catalyst consisting of both CrO_*x*_/Al_2_O_3_ for dehydrogenation of propane to propylene and ZSM-5 for furan pyrolysis.^289^ Three catalysts with different ratios (0.5, 1.0, 2.0) of CrO_*x*_/Al_2_O_3_ to ZSM-5 were tested at 550 °C in a fixed-bed reactor, delivering 12.0–20.9% and 10.8–13.4% aromatics yield, respectively, with or without propane as co-feed.^289^ Propane as co-feed with ZSM-5 as catalyst showed less improvement on aromatics formation than CrO_*x*_/Al_2_O_3__ZSM-5 catalysts, due to the deficiency of propane dehydrogenation to propylene.^289^ Toluene was produced in 37% selectivity using ZSM-5 as catalyst with propane as co-feed, while 33% toluene selectivity was observed in the absence of propane. Increased ratios of CrO_*x*_/Al_2_O_3_ to ZSM-5 further improved toluene selectivity to 43–50%. Mechanistic studies suggested a two-step reaction pathway over CrO_*x*_/Al_2_O_3__ZSM-5. Dehydrogenation of propane led to the formation of propylene, facilitated by CrO_*x*_/Al_2_O_3_ species. The *in situ* formed propylene reacted with furan over ZSM-5 through Diels–Alder reaction and further converted to aromatics, resulting in high toluene selectivity (Figure 11).^289^

##### 2.5.1.3 Catalytic Fast Pyrolysis of Furans with Ammonia as Co-feed

**Scheme 60:**
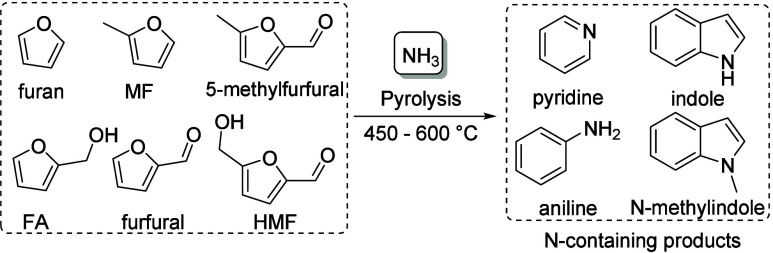
Catalytic Pyrolysis of Furans to N-Containing Chemicals with NH_3_ as Co-feed

Ammonia (NH_3_) was employed by Fu and Zhang as carrier gas for CFP of furans, generating a range of *N*-containing aromatic chemicals (Scheme 60, Table 21).^290−292^ In particular, indole was produced in good yields. A range of catalysts, including BEA zeolite, MCM-41, ZSM-5, and H-ZSM-5 with different Si/Al ratios were tested using furan and furfural as feeds, of which H-ZSM-5 (Si/Al = 25) exhibited the best activity for indole formation due to its proper pore structure and acidity.^290,291^ The use of H-ZSM-5 (Si/Al = 25) as catalyst for pyrolysis of furan with varying temperatures, WHSV, ammonia to feed molar ratios produced indoles in 3.5–35.7% carbon yields with 68.7–90.9% selectivity toward indole, along with 0.5–10.5% yields of pyridines and 2.1–5.5% yield of anilines (Table 21, entries 3–5).^290^ H-ZSM-5 (Si/Al = 25)-catalyzed pyrolysis of furfural generated 3.2–20.8% carbon yields of indoles with 67–92.9% selectivity to indole, depending on the conditions, when pyridines and anilines were detected in 1.1–8.9% and 0.5–3.4% carbon yields, respectively (Table 21, entries 11–14).^291^ Dilution of NH_3_ with 25% N_2_ for the CFP of furfural at 600 °C with WHSV 1.5 h^–1^ improved the indole carbon yield to 33.0% (Table 21, entry 20). Using 75% NH_3_–25% N_2_ as carrier gas, the CFP of FA, 5-methylfurfural, and HMF delivered indoles in 6.8%, 11.6%, and 6.9% yields, respectively, while pyridines were produced in 12.1%, 8.1%, and 1.4% carbon yields (Table 21, entries 21–26).^292^ On the contrary, the CFP of furan with 75% NH_3_–25% N_2_ as carrier gas at 600 °C produced 47.0% carbon yield of indoles, and 5.4% pyridines (Table 21, entries 27 and 28).^292^

**Table 21:** Co-feeding with Ammonia in the Catalytic Fast Pyrolysis of Furans to (Hetero)aromatics^a^

						Carbon yield (%)				Indoles distribution (%)		
Entry	Feed	NH_3_/feed ratio	Catalyst (Si/Al ratio)	WHSV (h^–1^)	*T* (°C)	Benzenoid aromatics	Pyridines	Anilines	Indoles	Indole	Methylindoles	Ref
1	Furan	8/1	ZSM-5 (50)	0.5	500	0.7	2.1	2.4	20.3	73.6	24.9	(290)
2	Furan	8/1	H-ZSM-5 (25–80)	0.5	500	0.4–1.4	2.0–2.7	2.1–4.3	22.6–31.7	74.2–87.0	10.8–24.3	(290)
3	Furan	8/1	H-ZSM-5 (25)	0.5	450–650	0.5–9.1	0.5–10.5	2.1–3.2	12.7–27.2	68.7–90.9	9.7–31.3	(290)
4	Furan	8/1	H-ZSM-5 (25)	0.25–1.0	500	0.5–1.7	2.6–3.0	3.5–5.4	19.3–35.7	72.1–79.5	20.5–27.9	(290)
5	Furan	2–12/1	H-ZSM-5 (25)	0.5	500	0.6–0.8	1.6–3.1	1.9–5.5	21.4–32.5	72.5–79.8	20.1–27.5	(290)
6	2-MF	8/1	H-ZSM-5 (25)	0.5	500	1.7	2.3	0.9	24.3	15.7	47.1	(290)
7	2-MF	8/1	H-ZSM-5 (25)	0.5	600	5.2	10.0	1.9	17.6	52.0	34.8	(290)
8	2-MF/FF (1/1)	8/1	H-ZSM-5 (25)	0.5	500	1.4	2.4	1.0	26.4	32.5	46.6	(290)
9	2-MF/FF (1/1)	8/1	H-ZSM-5 (25)	0.5	600	2.9	6.8	1.7	22.8	58.7	19.1	(290)
10	FF	2/1	ZSM-5 (50)	1.0	650	3.6	9.8	3.1	11.0	89.6	10.4	(291)
11	FF	2/1	H-ZSM-5 (25)	1.0	500–700	1.1–4.7	1.1–8.3	0.1–3.3	3.2–20.8	67.0–91.6	8.4–33.0	(291)
12	FF	2/1	H-ZSM-5 (25–80)	1.0	650	1.9–4.7	4.3–8.3	1.8–3.3	10.7–20.8	87.0–91.6	8.4–13.0	(291)
13	FF	2/1	H-ZSM-5 (25)	0.50–1.25	650	2.4–6.7	7.9–8.9	2.5–3.3	10.4–20.8	89.3–91.6	8.4–10.6	(291)
14	FF	2–15/1	H-ZSM-5 (25)	1.0	650	2.9–4.7	7.8–8.3	3.3–3.4	18.7–20.8	90.0–92.9	8.4–10.0	(291)
15	FF	25–100%^b^	H-ZSM-5 (25)	1.0^c^	600	3.0–4.5	2.7–3.8	1.0–1.6	9.3–19.4	84.0–86.5	13.5–16.0	(292)
16	FF	75%^b^	H-ZSM-5 (25)	1.0^c^	550–650	2.2–5.0	1.8–3.8	0.4–2.4	10.2–19.6	71.7–88.1	11.9–28.3	(292)
17	FF	75%^b^	H-ZSM-5 (25)	1.0–2.0^c^	600	2.0–3.2	2.6–3.8	1.0–1.5	16.6–20.1	84.6–86.5	13.5–15.4	(292)
18	FF	75%^b^	H-ZSM-5 (25)	1.0–2.0^d^	600	3.2–4.0	2.1–3.6	1.1–2.5	23.1–33.0	83.9–87.2	12.8–16.1	(292)
19	FF	75%^b^	H-ZSM-5 (25)	1.0–2.0^e^	600	5.5–5.9	4.7–6.9	1.9–2.3	15.6–16.9	87.5–89.3	10.7–12.5	(292)
20	FF	75%^f^	H-ZSM-5 (25)	1.5^d^	600	2.7–3.7	2.4–7.8	1.2–2.1	18.7–30.3	84.3–90.1	14.8–17.9	(292)
21	FA	100%^b^	H-ZSM-5 (25)	1.5^d^	600	4.0	9.2	–	4.3	42.3	57.7	(292)
22	FA	75%^b^	H-ZSM-5 (25)	1.5^d^	600	5.1	12.1	–	6.8	42.7	57.3	(292)
23	5-methylfurfural	100%^b^	H-ZSM-5 (25)	1.5^d^	600	7.0	6.6	–	8.6	33.2	66.8	(292)
24	5-methylfurfural	75%^b^	H-ZSM-5 (25)	1.5^d^	600	7.1	8.1	–	11.6	41.2	58.8	(292)
25	HMF	100%^b^	H-ZSM-5 (25)	1.5^d^	600	3.0	0.8	–	4.0	46.2	53.8	(292)
26	HMF	75%^b^	H-ZSM-5 (25)	1.5^d^	600	3.2	1.4	–	6.9	60.4	39.6	(292)
27	Furan	100%^b^	H-ZSM-5 (25)	1.5^d^	600	2.2	5.1	3.2	40.5	68.9	31.1	(292)
28	Furan	75%^b^	H-ZSM-5 (25)	1.5^d^	600	2.7	5.4	2.3	47.0	73.0	27.0	(292)

a Continuous-flow reactor; products were analyzed by GC-FID.

b NH_3_ concentration in N_2_; diluted NH_3_ flow rate = 40 mL/min.

c Residence time = 2.6 s.

d Residence time = 3.3 s.

e Residence time = 4.0 s.

f Diluted NH_3_ flow rate = 20–80 mL/min.

#### 2.5.2 1,2,4-Benzenetriol from HMF

**Scheme 61:**
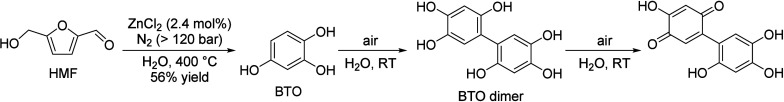
Conversion of HMF to BTO and BTO Dimerization^295,296^

In the 1990s, van Bekkum observed the formation of 1,2,4-benzenetriol (BTO) in >25% yields from an aqueous HMF solution kept at 350 °C or 330 °C under 280 bar autonomous pressure.^293,294^ In 2018, Deuss and co-workers re-investigated this conversion by employing Lewis acids as catalysts (Scheme 61).^295^ Catalyzed by 2.4 mol% ZnCl_2_ under N_2_ (> 120 bar), a 56% yield of BTO was obtained from HMF in H_2_O after reaction at 400 °C. Dimerization of BTO in aqueous solution under air atmosphere at room temperature led to formation of the BTO dimer, which could be subsequently oxidized under air to the hydroquinone-containing dimer.^295^

**Scheme 62:**
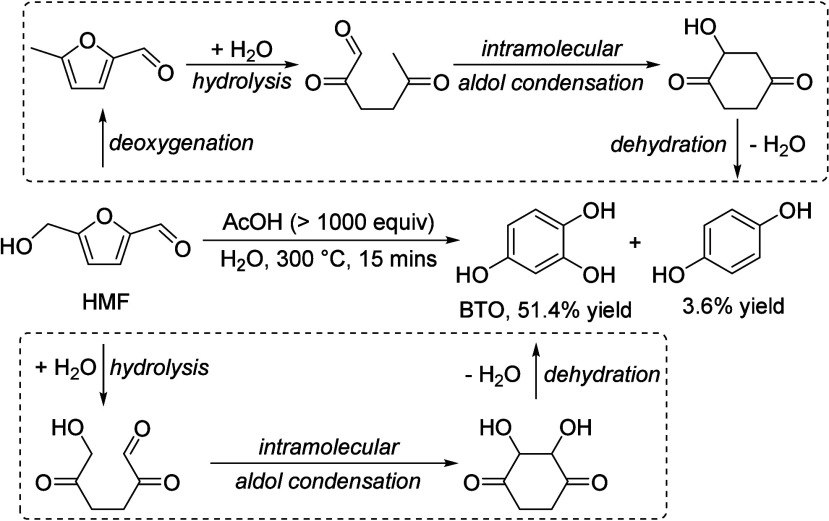
Conversion of HMF to BTO and Hydroquinone with Excess Acetic Acid^297^

In 2020, Deng’s group reported the use of an excess amount of acetic acid (> 1000 equiv) for the conversion of HMF to BTO at 300 °C.^297^ In addition to 51% BTO, 3.6% hydroquinone was also observed (Scheme 62).^297^ A plausible mechanism for the formation of BTO is shown in Scheme 62. Ring-opening of HMF by hydrolysis produces 6-hydroxy-2,5-dioxohexanal, which undergoes intramolecular aldol condensation and dehydration to form BTO. Formation of hydroquinone was ascribed to the deoxygenation of HMF to 5-methylfurfural prior to ring-opening.^297^ At 100 °C, a 94% yield of BTO dimer was produced from the aqueous solution of BTO under air.^297^

**Scheme 63:**
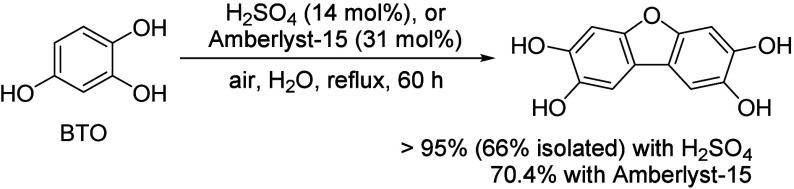
Conversion of BTO to 2,3,7,8-Tetrahydroxy-dibenzofuran^295^

Conversion of BTO to 2,3,7,8-tetrahydroxy-dibenzofuran was achieved in >95% yield by employing 14 mol% of H_2_SO_4_ in aqueous solution (Scheme 63).^295^ The use of recyclable Amberlyst-15 generated 2,3,7,8-tetrahydroxy-dibenzofuran in 70.4% yield.^298^

#### 2.5.3 Aromatics Synthesized via Diels–Alder/Dehydration Reactions of Furans

**Scheme 64:**
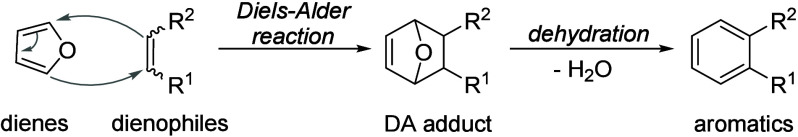
Diels–Alder/Dehydration of Furan and Olefins

**Scheme 65:**
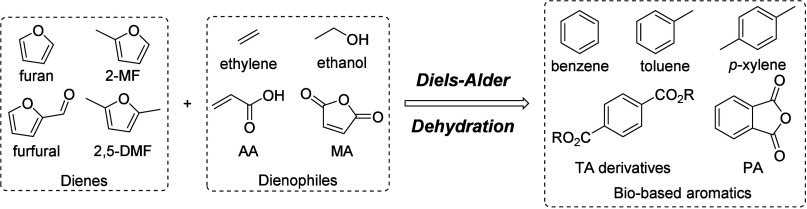
Diels–Alder Reactions of Furans with Various Dienophiles to Bio-Based Aromatics

Diels–Alder reaction of furans with bio-derived or fossil-based dienophiles, followed by dehydration has been extensively exploited for the production of bio-based aromatics (Scheme 64), in particular for the formation of *p*-xylene, the precursor to terephthalic acid (TA), one of the monomers for the formation of polyethylene terephthalate (PET). A number of reviews have appeared on this approach^299−302^ mainly focusing on mechanism,^299^ aromatics from C_6_-furans,^301^ and syntheses of *p*-xylene.^300^ In this section, we will discuss the conversion of furans to bio-based aromatics through Diels–Alder reactions with various dienophiles, including ethylene, alkynes, and maleic anhydride (Scheme 65).

##### 2.5.3.1 Benzene, Toluene, *p*-Xylene, and Alkylbenzenes Using Ethylene as Dienophile

**Scheme 66:**
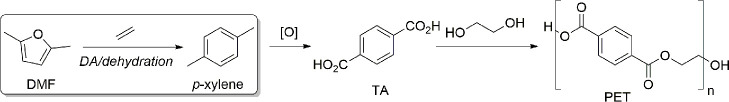
DA/Dehydration of 2,5-DMF and Ethylene to TA

Ethylene, which can be obtained from bio-ethanol by dehydration, is the simplest dienophile for the DA reactions of furans, delivering benzene, toluene, *p*-xylene, or other alkylbenzenes (BTX) as products, depending on the structure of the furans. Inspired by the many-fold industrial applications of BTX, this reaction has attracted extensive attention. In particular, the use of *p*-xylene as TA precursor for PET production brought its renewable production from 2,5-DMF into sharp focus (Scheme 66). The low price of ethylene and the 100% carbon atom economy of this strategy have accelerated the rapid development in this field. Table 22 summarizes the reported conditions for the DA/dehydration reaction of 2,5-DMF and ethylene.

**Table 22:**
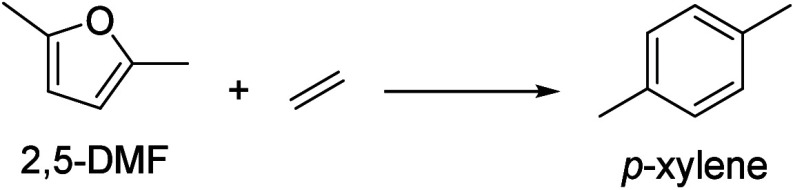
Reported Diels–Alder Reactions of 2,5-DMF and Ethylene to *p*-Xylene

Entry	*P*_ethylene_ (bar)	Catalyst (Si/Al ratio)	Sub/cat. (wt)	Solvent (conc)	*T* (°C)	*t* (h)	Conv (%)	Sel. (%)	Yield (%)	Ref
1	62	H-Y (30)	44	–	300	24	95	51	48	(303)
2	62	H-Y (30)	44	*n*-heptane (25 vol%)	300	24	95	75	71	(303)
3	62	H-BEA (12.5)	21	*n*-heptane (1.0 M)	250	24	>99	90	90	(304)
4	62	H-BEA (19)	21	*n*-heptane (1.0 M)	250	24	78	80	62	(304)
5	62	H-Y (2.6)	21	*n*-heptane (1.0 M)	250	24	52	74	38	(304)
6	62	H-ZSM-5 (19)	21	*n*-heptane (1.0 M)	250	24	54	70	38	(304)
7	62	Niobic acid	21	*n*-heptane (1.0 M)	250	24	45	76	34	(304)
8	62	γ-Al_2_O_3_	21	*n*-heptane (1.0 M)	250	24	52	58	30	(304)
9	62	H-BEA (12.5)	–a	*n*-heptane (1.35 M)	250	24	55	55	30	(307)
10	62	Sn-BEA (126)^d^	–a	*n*-heptane (1.35 M)	250	24	63	68	43	(307)
11	62	Zr-BEA (168)^d^	–a	*n*-heptane (1.35 M)	250	24	83	73	61	(307)
12	62	Ti-BEA (128)^d^	–a	*n*-heptane (1.35 M)	250	24	60	68	41	(307)
13	62	P-BEA (1471) (27.1)^e^	100^c^	*n*-heptane (1.35 M)	250	24	99	98	97	(308)
14	62	P-SPP (27.3)^e^	100^c^	*n*-heptane (1.35 M)	250	24	100	97	97	(308)
15	62	P-celite (13.3) (5.0)^e^	100^c^	*n*-heptane (1.35 M)	250	24	96	94	90	(308)
16	62	P-BEA (1471) (27.1)^e^	498^c^	*n*-heptane (1.35 M)	250	24	99	96	95	(308)
17	62	P-SPP (27.3)^e^	498^c^	*n*-heptane (1.35 M)	250	24	97	89	86	(308)
18	62	H_3_PO_4_	100^c^	*n*-heptane (1.35 M)	250	24	96	43	41	(308)
19	62	H-BEA (12.5)	4 mM^f^	*n*-heptane (1.35 M)	250	24	98	65	64	(308)
20	62	Zr-BEA (168)^d^	4 mM^f^	*n*-heptane (1.35 M)	250	24	99	73	72	(308)
21	62	H-BEA (12.5)	1 mM^f^	*n*-heptane (1.35 M)	250	24	55	55	30	(308)
22	62	Zr-BEA (168)^d^	1 mM^f^	*n*-heptane (1.35 M)	250	24	84	77	65	(308)
23	50	NSP-BEA (15)^b^	111	*n*-heptane (25 vol%)	250	24	99	80	79	(309)
24	50	NSP-BEA (25)^b^	111	*n*-heptane (25 vol%)	250	24	98	70	68	(309)
25	50	C-BEA (12.5)	111	*n*-heptane (25 vol%)	250	24	98	75	73	(309)
26	50	Si-Al-O (0.5)	111	*n*-heptane (25 vol%)	250	24	98	67	66	(309)
27	50	NS-2.5 (48–340)	111	*n*-heptane (25 vol%)	250	24	92–96	63–72	58–70	(310)
28	50	NS-7.5 (47)	111	*n*-heptane (25 vol%)	250	24	89	64	57	(310)
29	50	ZSM-5 (42)	111	*n*-heptane (25 vol%)	250	24	50	62	31	(310)
30	60	K-Y (2.6)	9.6	*n*-hexane (0.5 M)	250	15	50	42	21	(311)
31	55	H-ZSM-5 (36)	46.8	*n*-hexane (25 vol%)	250	24	16	51	8	(312)
32	55	Desilicated ZSM-5 (25)	46.8	*n*-hexane (25 vol%)	250	24	51	59	30	(312)
33	30	H-BEA (7)	–	*n*-heptane (1.56 M)	200	20	61	–	54	(305)
34	30	H-BEA (7)	–	*n*-heptane (1.56 M)	300	20	89	–	77	(305)
35	30	H-BEA (7)	–	*n*-heptane (0.78 M)	300	20	98	–	78	(305)
36	30	H-BEA (7)	–	*n*-heptane (3.12 M)	300	20	60	–	50	(305)
37	40	H-BEA (22)	–	*n*-heptane (1.56 M)	300	20	99	–	97	(305)
38	40	H-BEA (30)	–	*n*-heptane (1.57 M)	300	20	100	94	94	(306)
39	40	H-BEA (30)^g^	–	*n*-heptane (1.57 M)	300	20	100	94–97	94–97	(306)
40	40	γ-Al_2_O_3_	–	*n*-heptane (1.57 M)	300	20	72	58	42	(306)
41	52	BEA (11.8)	46.8	*n*-heptane (0.52 M)	240	20	45	46	21	(313)
42	52	DS-ITQ-2_L (10.9)	46.8	*n*-heptane (0.52 M)	240	20	64	50	32	(313)
43	52	DS-ITQ-2_H (11.6)	46.8	*n*-heptane (0.52 M)	240	3	49	44	22	(313)
44	52	DS-ITQ-2_H (11.6)	46.8	*n*-heptane (0.52 M)	240	20	78	55	43	(313)
45	30	SAA (1)	2.6	1,4-dioxane (10 wt%)	250	6	90	67	60	(314)
46	20	H-BEA (12.5)	2.6	1,4-dioxane (10 wt%)	250	4	68	68	47	(314)
47	20	H-BEA (12.5)	2.6	1,4-dioxane (10 wt%)	300	4	88	60	53	(314)
48	45	SiO_2_–SO_3_H	4	*n*-heptane (1.04 M)	250	6	67	89	60	(316)
49	45	H-BEA (25)	4	*n*-heptane (1.04 M)	250	6	99	82	81	(316)
50	45	H-BEA (100)	4	*n*-heptane (1.04 M)	250	6	58	72	42	(316)
51	45	SiO_2_–SO_3_H	4	*n*-heptane (0.35 M)	250	6	77	89	69	(316)
52	45	SiO_2_–SO_3_H	4	*n*-heptane (0.52 M)	250	6	70	85	60	(316)
53	45	SiO_2_–SO_3_H	4	*n*-heptane (2.08 M)	250	6	51	89	45	(316)
54	20	HPW	69.4	1,4-dioxane (11.5 vol%)	250	1	41	50	21	(315)
55	20	HPW	23.1	1,4-dioxane (11.5 vol%)	250	1	68	53	36	(315)
56	20	15% HPW/SiO_2_	23.1	1,4-dioxane (11.5 vol%)	250	1	47	58	27	(315)
57	20	15% HPW/SiO_2_	23.1	1,4-dioxane (11.5 vol%)	250	6	81	73	59	(315)
58	20	HSiW	69.4	1,4-dioxane (11.5 vol%)	250	1	43	52	22	(315)
59	20	HSiW	23.1	1,4-dioxane (11.5 vol%)	250	1	72	46	33	(315)
60	20	15%-HSiW/SiO_2_	23.1	1,4-dioxane (11.5 vol%)	250	1	42	70	30	(315)
61	20	15% HSiW/Al_2_O_3_	23.1	1,4-dioxane (11.5 vol%)	250	1	45	50	22	(315)
62	20	H-BEA (12.5)	23.1	1,4-dioxane (11.5 vol%)	250	1	49	58	28	(315)
63	20	H-BEA (19)	23.1	1,4-dioxane (11.5 vol%)	250	1	49	57	28	(315)
64	20	SAA (1)	23.1	1,4-dioxane (11.5 vol%)	250	1	37	57	21	(315)
65	30	5–20% HSiW/SiO_2_	23.1	1,4-dioxane (11.5 vol%)	250	6	24–94	59–85	14–80	(315)
66	30	15% HSiW/SiO_2_	23.1	1,4-dioxane (11.5 vol%)	200–300	6	35–96	53–85	18–82	(315)
67	30	SiP^m^	–	*n*-dodecane + *n*-tridecane (11.1 wt%)	250	6	84–85	61–69	51–59	(317)
68	30	TiP^n^	–	*n*-dodecane + *n*-tridecane (11.1 wt%)	250	6	80	71	57	(317)
69	30	SiP or TiP^n^	–	*n*-dodecane + *n*-tridecane (11.1 wt%)	250	12	100	∼80	∼80	(317)
70	30	H_3_PO_4_	–	*n*-dodecane + *n*-tridecane (11.1 wt%)	250	6	72	39	28	(317)
71	20	SiO_2_/Al_2_O_3_ (3.4)	13.3	hexadecane (10 wt%)	250	–	60	40	24	(318)
72	20	H-Y (2.55)	13.3	hexadecane (10 wt%)	250	–	60	52	31	(318)
73	20	WO_*x*_–ZrO_2_^i^	13.3	hexadecane (10 wt%)	250	–	60	77	46	(318)
74	20	Niobic acid	13.3	hexadecane (10 wt%)	250	–	60	57	34	(318)
75	20	TFA	13.3	hexadecane (10 wt%)	250	–	60	40	24	(318)
76	20	WO_*x*_–ZrO_2_^i^	13.3	hexadecane (10 wt%)	250	1.5–6	>99	80	80	(318)
77	50	WO_*x*_–ZrO_2_^j^	111.3	*n*-heptane (25 vol%)	250	24	66–95	56–91	37–85	(319)
78	54	NbO_*x*_-based catalyst	8.3	*n*-heptane (1 M)	250	6	87	93	81	(320)
79	54	Sn-BEA (−)	8.3	*n*-heptane (1 M)	250	6	82	74	61	(320)
80	54	NbO_*x*_-based catalysts^k^	8.3	*n*-heptane (1 M)	250	6	20–89	84–92	17–82	(320)
81	40	NbO_*x*_-based catalysts	28	1,4-dioxane (4 M)	250	2	16–50	89–96	7–41	(321)
82	40	8Nb-MCM^o^	28	1,4-dioxane (2.5–4 M)	250	2	56–61	95–96	48–55	(321)
83	40	8Nb-MCM^o^	28	solvent (4 M)	250	2	25–61	95–97	21–55	(321)
84	40	8Nb-MCM^o^	28	–	250	2	>99	78	66	(321)
85	40	8Nb-MCM^o^	28	1,4-dioxane (4 M)	250	10	>99	96	92	(321)
86	40	2–16Nb-MCM^o^	28–210	1,4-dioxane (4 M)	250	2	45–58	89–96	41–48	(321)
87	40	2Nb-MCM^o^^,^^p^	28–76	1,4-dioxane (4 M)	250	2	51–65	79–87	29–34	(321)
88	20	H-BEA (12.5)	260^a^	*n*-heptane (1.04 M)	250	6	62	59	36	(322)
89	20	Sn-BEA (530)	260^a^	*n*-heptane (1.04 M)	250	6	66	71	46	(322)
90	20	P-BEA (530)	260^a^	*n*-heptane (1.04 M)	250	6	77	77	66	(322)
91	20	SnPO^l^	260^a^	*n*-heptane (1.04 M)	250	6	68–94	72–92	49–85	(322)
92	20	SnPO (1.75)^l^	260^a^	*n*-heptane (1.04 M)	250	18	>99	93	93	(322)
93	20	ZrP_1.5_-SBA	4 wt%^q^	*n*-heptane (2.35 M)	250	24	91	96	87	(323)
94	35	CF_2_ClCOOH	10	1,4-dioxane (0.2 M)	200	24	72	69	50	(324)
95	35	Sc(OTf)_3_	100	1,4-dioxane (0.2 M)	200	24	77	70.1	54	(324)
96	34.5	CuCl_2_	10–100	1,4-dioxane (5 wt%)	250	7	65–97	91–101	64–90	(325)
97	34.5	Cu(OTf)_2_	20–200	1,4-dioxane (5 wt%)	250	7	95–100	86–101	86–100	(325)
98	34.5	Y(OTf)_3_	20–100	1,4-dioxane (5 wt%)	250	7	98–100	84–94	84–92	(325)

a Substrate/catalyst acid site.

b Nanosponge-like mesoporous BEA zeolite.

c Molar ratio of DMF to P.

d Si/metal ratios.

e Si/P ratio.

f Concentration of acid sites on the catalyst.

g Modified with different alumina.

h Phosphotungstic acid.

i 15 wt% WO_3_.

j 16 wt% WO_3_. Calcinated at 650–950 °C for 1–3 h.

k Prepared at different pH (1, 2, and 3).

l Different P/Sn molar ratios (1.00–2.00).

m Phosphated SiO_2_ (calcination at 300 °C or 500 °C).

n Phosphated TiO_2_ (calcination at 500 °C).

o *x*Nb/MCM indicates *x* wt% of Nb.

p Addition of phosphoric acid (0.2, 0.5, or 1 M).

q Catalyst concentration.

In 2012, Dauenhauer screened various zeolites with different Si/Al ratios for the DA/dehydration reaction of 2,5-DMF with ethylene (Table 22, entries 1 and 2).^303^ The results suggested that the DA addition of 2,5-DMF and ethylene did not take place on the active sites inside the zeolite, whereas the following dehydration reaction of the DA adduct was strongly affected by the Brønsted acidic sites within the zeolite. Elevated temperatures and addition of aliphatic solvents significantly helped to prevent side reactions, such as the hydrolysis to 2,5-hexanedione and polymerization.^303^ A 75% selectivity toward *p*-xylene with 95% 2,5-DMF conversion was obtained under 62 bar ethylene using *n*-heptane as solvent and H-Y (Si/Al = 30) as catalyst at 300 °C.^303^

Various heterogeneous catalysts were studied by the same group for *p*-xylene formation at 250 °C under 62 bar ethylene, including H-BEA, H-ZSM-5, H-Y, niobic acid, and γ-Al_2_O_3_ (Table 22, entries 3–8).^304^ Among all these catalysts, H-BEA showed an exceptional activity and provided 90% selectivity to *p*-xylene with full 2,5-DMF conversion.^304^ Under the same conditions, toluene was produced in 46% selectivity from 2-MF with full conversion and benzene was generated in 35% selectivity from furan with 70% conversion (Table 23, entries 1 and 2).^304^ This work significantly brought the use of BEA zeolites into focus. In 2018, Zhang and co-workers studied the use of BEA zeolites with different Si/Al ratios for *p*-xylene production from 2,5-DMF under 40 bar ethylene at 300 °C (Table 22, entries 33–37).^305^ Dealuminated H-BEA with a Si/Al ratio of 22, obtained by treating H-BEA (Si/Al=19) with an aqueous HNO_3_ solution, was found to be the best catalyst, which delivered *p*-xylene in 97% yield.^305^ After five recycles, the catalyst was still sufficiently active to deliver *p*-xylene in 85% yield.^305^ Toluene and benzene were synthesized in 50% selectivity at 92% 2-MF conversion and 28% selectivity at 68% furan conversion, respectively (Table 23, entries 3 and 4).^305^ Further dealumination of H-BEA by treatment with an HCl solution increased the Si/Al ratio to 30; this catalyst produced *p*-xylene in 94% yield with full 2,5-DMF conversion (Table 22, entry 38).^306^ Modification of dealuminated H-BEA with various alumina sources, such as Al(NO_3_)_3_, Al(O*i*Pr)_3_, and γ-Al_2_O_3_, had a limited effect on the reaction (Table 22, entries 39 and 40).^306^

**Table 23:**
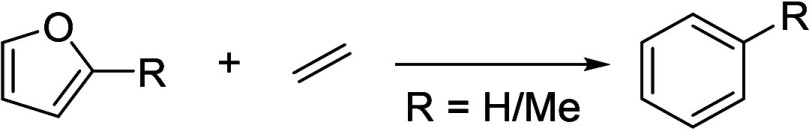
Conversion of 2-MF to Toluene and Furan to Benzene under Ethylene

Entry	Diene	*P*_ethylene_ (bar)	Catalyst (Si/Al ratio)	Sub/cat. (wt)	Solvent (conc)	*T* (°C)	*t* (h)	Conv (%)	Sel. (%)	Yield (%)	Ref
1	2-MF	62	H-BEA (12.5)	21	*n*-heptane (1.0 M)	250	48	>99	46	46	(304)
2	Furan	62	H-BEA (12.5)	21	*n*-heptane (1.0 M)	250	72	70	35	25	(304)
3	2-MF	40	H-BEA (22)		*n*-heptane (1.56 M)	300	20	92	50	46	(305)
4	furan	40	H-BEA (22)		*n*-heptane (1.56 M)	300	20	68	28	19	(305)
5	2-MF	20	WO_*x*_-ZrO_2_^a^	13.3	hexadecane (10 wt%)	250	1.5–6	>99	34	34	(318)
6	Furan	20	WO_*x*_-ZrO_2_^a^	13.3	hexadecane (10 wt%)	250	1.5–6	>99	18	18	(318)
7	2-MF	40	8Nb-MCM^b^	87	1,4-dioxane (4 M)	250	8	∼80	-	∼68	(321)
8	Furan	40	8Nb-MCM^b^	87	1,4-dioxane (4 M)	250	8	∼70	-	∼52	(321)
9	2-MF	35	CF_2_ClCOOH	10	1,4-dioxane (0.2 M)	200	24	65	36	23	(324)
10	furan	35	CF_2_ClCOOH	10	1,4-dioxane (0.2 M)	200	24	43	11	5	(324)
11	2-MF	35	Sc(OTf)_3_	100	1,4-dioxane (0.2 M)	200	24	76	46	35	(324)
12	Furan	35	Sc(OTf)_3_	100	1,4-dioxane (0.2 M)	200	24	48	17	8	(324)
13	2-MF	30	H-BEA (12.5)	23.2	1,4-dioxane (11.1 vol%)	250	8	94	27	25	(326)
14	2-MF	30	AlCl_3_	23.2	1,4-dioxane (11.1 vol%)	250	8	82	55	45	(326)
15	2-MF	30	AlCl_3_	23.2	1,4-dioxane (11.1 vol%)	250	24	99	70	70	(326)
16	2-MF	30	VCl_3_	23.2	1,4-dioxane (11.1 vol%)	250	8	82	51	42	(326)
17	2-MF	30	CrCl_3_	23.2	1,4-dioxane (11.1 vol%)	250	8	76	48	37	(326)
18	2-MF	30	SnCl_4_	23.2	1,4-dioxane (11.1 vol%)	250	8	92	34	31	(326)
19	2-MF	30	YbCl_3_	23.2	1,4-dioxane (11.1 vol%)	250	8	74	37	28	(326)
20	2-MF	30	ZnCl_2_	23.2	1,4-dioxane (11.1 vol%)	250	8	92	33	30	(326)
21	2-MF	30	Na-Y (2.5)	23.2	1,4-dioxane (11.1 vol%)	250	8	54	69	38	(326)
22	2-MF	30	Li-Y (2.5)	23.2	1,4-dioxane (11.1 vol%)	250	8	55	62	34	(326)
23	2-MF	35	Na-Y (2.5)	11.6	1,4-dioxane (11.1 vol%)	250	4–24	32–96	62–68	22–64	(326)

a 15 wt% WO_3_.

b 8 wt% of Nb.

**Scheme 67:**
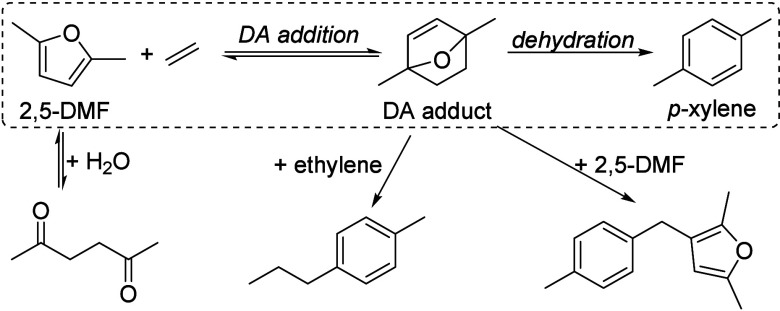
Reaction of 2,5-DMF with Ethylene to *p*-Xylene and Side Reactions

Lewis acidic zeolites, including Zr-, Sn-, and Ti-BEA, were prepared and tested for the DA/dehydration of 2,5-DMF and ethylene (62 bar) (Table 22, entries 9–12).^307^ Compared to H-BEA, Lewis acidic zeolites remained active for a longer period of time.^307^ In particular, Zr-BEA exhibited excellent catalytic performance resulting in high *p*-xylene selectivity (90%), full 2,5-DMF conversion, and slow catalyst deactivation.^307^ The superior behavior of Zr-BEA was due to its lower activity to hydrolysis and its weakly acidic sites which prevented polymerization of 2,5-hexanedione.^307^ The *p*-xylene formation rate was dependent on the amount of Lewis acidic sites at low catalyst loading but independent at high catalyst loading.^307^ P-containing zeolites, both BEA zeolite (P-BEA) and self-pillared pentasil zeolite (P-SPP), converted 2,5-DMF under 62 bar ethylene to *p*-xylene in 97% yield (Table 22, entries 13 and 14). These excellent results were attributed to the improved catalytic performance in the dehydration reaction and the lack of side reactions – alkylation and oligomerization (Scheme 67).^308^ Non-zeolitic P-celite as catalyst also delivered a high yield (90%) of *p*-xylene (Table 22, entry 15).^308^ Re-used P-BEA gave 94% selectivity to *p*-xylene at 98% 2,5-DMF conversion after the third recycling test. Reuse of P-SPP resulted in the formation of *p*-xylene in 65% yield at 76% 2,5-DMF conversion.^308^

In 2016, Kim and co-workers prepared nanosponge-like mesoporous beta zeolites (NSP-BEA) with various Si/Al ratios and used these for the DA/dehydration reaction of 2,5-DMF to ethylene (Table 22, entries 23–26).^309^ At 250 °C, the use of NSP-BEA (Si/Al = 15) under 50 bar ethylene resulted in a 79% yield of *p*-xylene. The authors ascribed these good results to the presence of the mesopores and the large amount of Brønsted acidic sites.^309^

Other types of zeolites have also been investigated. In 2017, Kim synthesized a range of mesoporous MFI zeolite nanosheets in different crystal sizes and Si/Al ratios (NS-2.5 and NS-7.5), for the DA/dehydration reaction of 2,5-DMF (Table 22, entries 27–29).^310^ The prepared MFI-type zeolites exhibited superior performance, compared to the parent ZSM-5, producing *p*-xylene in 63–72% selectivity with 89–96% 2,5-DMF conversion.^310^ Ion-exchanged faujasite zeolites, including Li-Y, Na-Y, K-Y, Rb-Y, and Cs-Y, however, showed very poor activity for this conversion and only produced a maximum 21% yield of *p*-xylene using K-Y (Si/Al = 2.6) as catalyst after 15 h reaction at 250 °C under 60 bar ethylene (Table 22, entry 30).^311^

ZSM-5, desilicated by treating with NaOH, produced 59.4% selectivity of *p*-xylene at 51% 2,5-DMF conversion using hexane as solvent under 55 bar ethylene at 250 °C, while use of the parent ZSM-5 resulted in 50.7% selectivity of *p*-xylene at 16% 2,5-DMF conversion (Table 22, entries 31 and 32).^312^

In 2020, Corma and co-workers prepared DS-ITQ-2 zeolites, which is a MWW-type zeolite with a large external surface area made by external hemicavities or cups in which the oxanorbornene structure could be stabilized by using hexamethyleneimine (HMI) and *N*-hexadecyl-*N*’-methyl-DABCO, and tested these catalysts for *p*-xylene production from 2,5-DMF and ethylene (Table 22, entries 41–44).^313^ At 240 °C, the DS-ITQ-2 zeolites showed better catalytic behavior than the BEA and FAU zeolites for the DA/dehydration reaction of 2,5-DMF under 52 bar ethylene, producing *p*-xylene with 55% selectivity at 78% 2,5-DMF conversion.^313^

Instead of well-structured zeolites, Jae prepared silica-alumina aerogels (SAAs) with different Si/Al ratios as catalysts for the DA reaction of 2,5-DMF and ethylene (30 bar) at 250 °C, delivering *p*-xylene in up to 60% yield (Table 22, entries 45–47).^314^ The authors also found that the *p*-xylene yield was correlated to the concentration of Brønsted acidic sites, suggesting that the dehydration of the DA adduct is the rate-limiting step under these conditions.^314^

In 2016, Jae screened a range of phosphotungstic acid (HPW) and silicotungstic acid (HSiW) catalysts supported on oxides, including SiO_2_, Al_2_O_3_, ZrO_2_, and TiO_2_, for the DA/dehydration of 2,5-DMF to synthesize *p*-xylene (Table 22, entries 54–66).^315^ HSiW supported on SiO_2_ with 15% loading was selected as the best catalyst, which produced *p*-xylene in 80% yield with 94% 2,5-DMF conversion after 6 h reaction at 250 °C under 30 bar ethylene.^315^

Tan and co-workers prepared a mesoporous silica-supported sulfonic acid (SiO_2_-SO_3_H) for the conversion of 2,5-DMF to *p*-xylene (Table 22, entries 48–53).^316^ At 250 °C, 85–89% selectivity of *p*-xylene at 50–77% 2,5-DMF conversion was obtained using *n*-heptane as solvent, depending on concentration.^316^ Phosphated SiO_2_ and phosphated TiO_2_ were prepared and utilized for DA/dehydration of 2,5-DMF under 30 bar ethylene (Table 22, entries 67–70). Phosphated SiO_2_ and TiO_2_ calcined at 773 K were identified as the most active and selective catalysts, exhibiting a high *p*-xylene selectivity of 70% at a 2,5-DMF conversion of 80% at 250 °C after 6 h.^317^

In 2013, Dumesic and co-workers prepared WO_*x*_-ZrO_2_ catalysts, containing both Brønsted and Lewis acidic sites, and compared their catalytic activity in the DA/dehydration of 2,5-DMF under 20 bar ethylene to H-Y, niobic acid, γ-Al_2_O_3_, amorphous SiO_2_/Al_2_O_3_, and other acids (Table 22, 71–76). At 60% 2,5-DMF conversion, WO_*x*_-ZrO_2_ showed the highest selectivity (70%) to *p*-xylene, while only 10–57% selectivity to *p*-xylene was observed using the other acidic catalysts.^318^ At full conversion of 2,5-DMF, 2-MF, and furan, *p*-xylene, toluene, and benzene were produced in 80%, 34%, and 18% yields respectively under 20 bar ethylene catalyzed by WO_*x*_-ZrO_2_ at 250 °C (Table 23, entries 5 and 6).^318^ Both calcination temperature and time affect the activity of WO_*x*_-ZrO_2_ for the *p*-xylene production, which was ascribed to the Brønsted acidic sites on the surface of the catalysts.^318,319^

In 2018, Shen prepared a NbO_*x*_-based catalyst and investigated their catalytic performance for the DA reaction of 2,5-DMF with ethylene varying the reaction time, amount of acid, and reaction temperature (Table 22, entries 78–80).^320^ Under 54 bar ethylene, *p*-xylene was obtained in 92.7% selectivity at 87.2% 2,5-DMF conversion at 250 °C.^320^ After five recycles, the catalyst still produced *p*-xylene with the same selectivity and only a 3.7% decreased 2,5-DMF conversion.^320^ In 2022, Cao and co-workers supplemented the research on NbO_*x*_-based catalysts for *p*-xylene production from 2,5-DMF and ethylene (Table 22, entries 81–87).^321^ A series of non-zeolitic NbO_*x*_-based catalysts were synthesized and tested for DA/dehydration reaction of 2,5-DMF under 40 bar ethylene, generating *p*-xylene in 7–41% yield.^321^ Atomically dispersed NbO_*x*_ supported on MCM-type silica with mesoporous structure (Nb/MCM) as catalyst delivered up to 92% yield of *p*-xylene at 250 °C under optimized conditions.^321^ Toluene was obtained from 2-MF in ∼68% yield catalyzed by Nb/MCM with 8% Nb loading under 40 bar ethylene, while benzene was produced from furan in ∼52% yield (Table 23, entries 7 and 8).^321^ Recycling experiments of the Nb/MCM catalyst for *p*-xylene formation revealed its good reusability, showing only slightly decreased activity after having been reused five times without regeneration and fully restored catalytic activity after calcination.^321^

Tan’s group synthesized SnPO catalysts with different P/Sn ratios and tested their catalytic activity for the conversion of 2,5-DMF to *p*-xylene at 250 °C under 20 bar of ethylene (Table 22, entries 88–92).^322^ Compared to H-BEA and Sn-BEA, the SnPO catalyst showed good activities and generated *p*-xylene in up to 85% yields after 6 h reaction.^322^ Using SnPO (P/Sn = 1.75) as catalyst, a prolonged reaction time of 18 h maximized the *p*-xylene yield to 93% with near full 2,5-DMF conversion.^322^ Bae and co-workers prepared mesoporous zirconium phosphates (ZrP) grafted on silica for *p*-xylene production from 2,5-DMF (Table 22, entry 93). Under 20 bar of ethylene, a 96% *p*-xylene selectivity with 91% 2,5-DMF conversion was obtained using ZrP grafted on SBA-15 as catalyst with a P/Zr ratio 1.5 at 250 °C.^323^

The liquid Brønsted acid CF_2_ClCOOH and the Lewis acid Sc(OTf)_3_ were also exploited for the DA/dehydration of 2,5-DMF and ethylene at 200 °C, affording *p*-xylene in 50% and 54% yields respectively (Table 22, entries 94 and 95).^324^ Toluene was obtained from 2-MF and ethylene catalyzed by CF_2_ClCOOH and Sc(OTf)_3_ in 23% and 35% yield respectively, while benzene was produced in 5% and 8% yield respectively (Table 23, entries 9–12).^324^

**Scheme 68:**
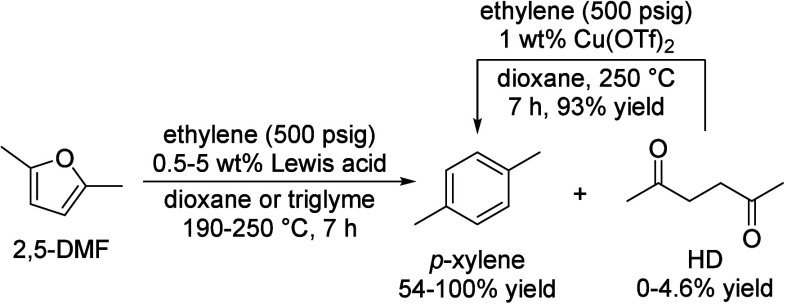
Micromidas’ Conversion of 2,5-DMF and HD to *p*-Xylene^325^

Micromidas inc. developed a technique to convert 2,5-DMF to *p*-xylene under 34.5 bar of ethylene at 190–250 °C in dioxane catalyzed by 0.5–5 wt% Lewis acids, producing *p*-xylene in 54–100% yields (Scheme 68 and Table 22, entries 96–98).^325^ In addition, 2,5-hexanedione (HD) was produced as side product in up to 4.6% yield. As HD can be converted to 2,5-DMF, they also convert HD to *p*-xylene in 93% yield using Cu(OTf)_2_ under the same conditions.^325^

**Scheme 69:**
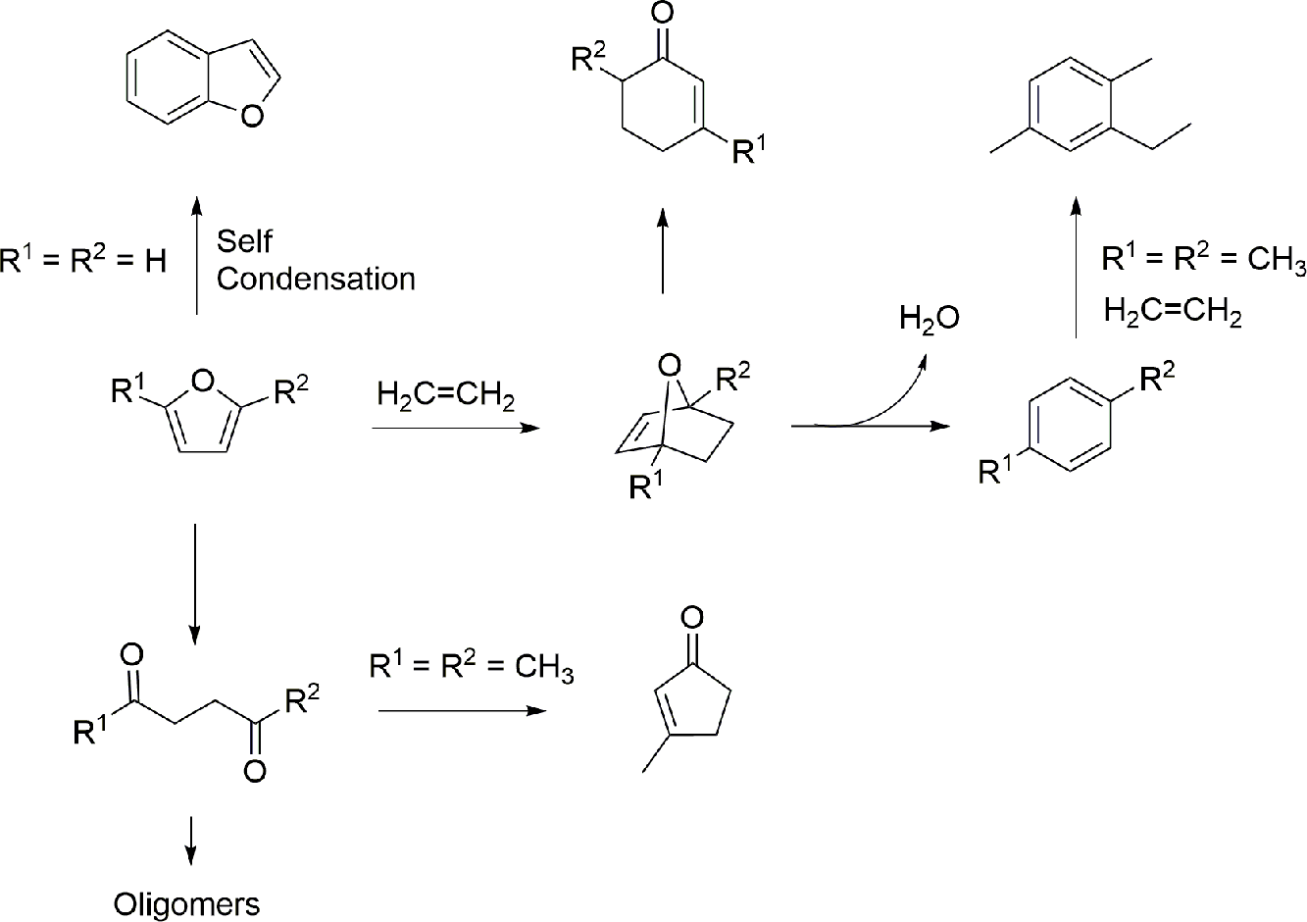
Reaction Pathways of Furans with Ethylene to Aromatics Reproduced with permission from ref (318). Copyright 2013 Wiley.

The methyl substituents enhance the reactivity of the furan rings in [4+2] cycloaddition reactions and stabilize the intermediates in the dehydration of the DA adducts, hence, the yields of toluene from 2-MF and benzene from furan are usually much lower.^304,305,318,321,324^ Dumesic elucidated the reason of the low aromatic selectivity in DA reaction of 2-MF and furan with ethylene (Scheme 69).^318^ Compared to 2,5-DMF, the ring-opening products of 2-MF and furan oligomerize quickly, driving the equilibrium toward the ring-opening products. Isomerization of DA adducts result in the formation unsaturated cyclohexanones. Benzofuran was also detected in solid acid-catalyzed reaction of furan. Table 23 summarizes reported approaches for the conversion of 2-MF and furan to toluene and benzene, respectively, by reaction with ethylene.

Jae reported the DA/dehydration reactions of 2-MF and ethylene for toluene production catalyzed by a series of catalysts, including metal chlorides, cation-exchanged Y zeolites, and metal-modified BEA zeolites (Table 23, entries 13–23).^326^ Under 30 bar of ethylene, the use of AlCl_3_ and VCl_3_ as catalysts produced toluene in 42–45% yields, while CrCl_3_, SnCl_4_, YbCl_3_, and ZnCl_2_ afforded toluene in more moderate yields (28–36%).^326^ The increased Lewis acidity of the metal chlorides raised the toluene yield. With AlCl_3_ as catalyst, the DA/dehydration reaction of 2-MF and ethylene produced toluene in 45% yield after 8 h and 70% yield after 24 h reaction at 250 °C.^326^ The increased selectivity over time probably is an indication that the dehydration is the rate-limiting step. Among all tested cation-exchanged Y zeolites, Li-Y and Na-Y showed superior activity, affording 34% and 38% yield of toluene, respectively, after 8 h reaction under 30 bar of ethylene at 250 °C. Increasing the ethylene pressure to 35 bar improved the toluene yield to 63% with Na-Y as catalyst after 24 h reaction.^326^

**Scheme 70:**
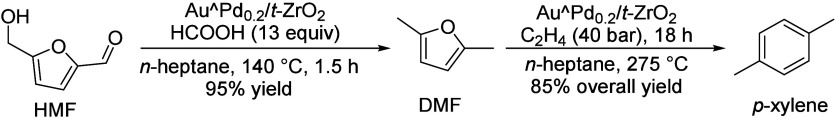
One-Pot Conversion of HMF to *p*-Xylene^328^

Whereas a variety of catalysts have been identified for the synthesis of *p*-xylene from 2,5-DMF through the DA/dehydration reaction with ethylene, the direct use of HMF and furfural has never been reported, probably due to the electron-withdrawing substituent.^327^ In 2018, Cao and Li prepared a bimetallic Pd-modified Au-clusters anchored on tetragonal-phase zirconia (Au^^^Pd_0.2_/*t*-ZrO_2_) as catalyst for both HMF hydrogenation to 2,5-DMF and DA/dehydration of 2,5-DMF under 40 bar ethylene and achieved a one-pot two-stage conversion of HMF to *p*-xylene in 85% overall yield (Scheme 70).^328^

**Scheme 71:**
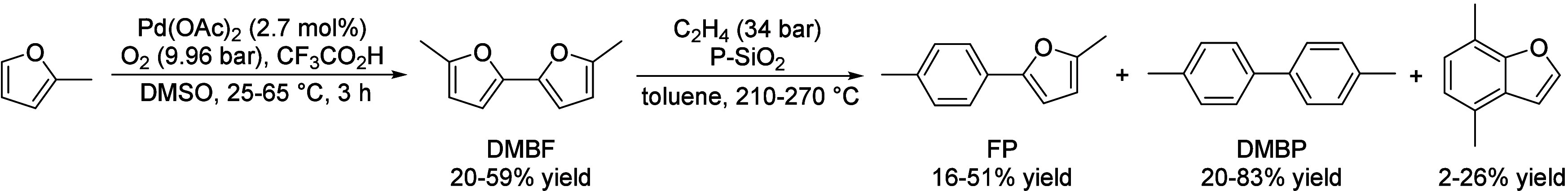
Synthesis of DMBP from 2-MF through Oxidative Coupling and DA/Dehydration Reactions^329^

4,4′-Dimethylbiphenyl (DMBP) has been synthesized by the DA/dehydration reaction of 5,5′-dimethyl-2,2′-bifuran (DMBF), obtained by Pd-catalyzed oxidative coupling of 2-MF in 20–59% yields with the aid of trifluoracetic acid and O_2_ (Scheme 71).^329^ P-SiO_2_ catalyzed DA/dehydration of DMBF under 34 bar of ethylene at 210–270 °C produced DMBP in 20–83% yields, along with 16–51% yields of 2-methyl-5-(*p*-tolyl)furan (FP) and 2–26% yields of 4,7-dimethylbenzofuran.^329^

**Scheme 72:**
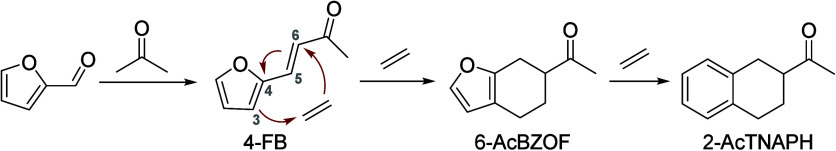
DA/Dehydration of 4-FB under Ethylene to 6-AcBZOF and 2-AcTNAPH^331^

4-(2-Furyl)-3-butene-2-one (4-FB), produced from bio-based furfural and acetone through aldol-condensation,^330^ reacts with ethylene through [4+2] addition to the 3,5-diene instead of the furan diene, affording 6-acetyl-4,5,6,7-tetrahydrobenzofuran (6-AcBZOF).^331^ The furan ring in 6-AcBZOF reacts with ethylene and forms 2-acetyl-1,2,3,4-tetrahydronaphthalene (2-AcTNAPH). In the absence of catalyst, an 86% yield of 6-AcBZOF was obtained under 35 bar ethylene. At 275 °C, H-BEA and Sn-BEA catalyzed the DA/dehydration of 4-FB and ethylene and delivered 2-AcTNAPH in 54% and 69% yields, respectively, while H-ZSM-5 and α-Al_2_O_3_ as catalyst only produced 25% and 20% yields of 2-AcTNAPH (Scheme 72).^331^

##### 2.5.3.2 Benzene, Toluene, *p*-Xylene, and Alkylbenzenes Using Other Dienophiles

**Scheme 73:**
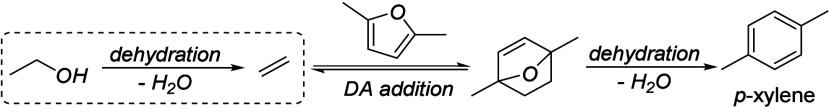
Ethanol as Ethylene Precursor for *p*-Xylene Production from 2,5-DMF

Although ethylene has been extensively used as dienophile in the DA reaction with 2,5-DMF for the high-yielding production of *p*-xylene, the use of high-pressure ethylene causes extra cost and challenges in large-scale production. Ethanol, a cheap liquid which can be obtained from renewables, can be dehydrated to ethylene at high temperatures. It has been employed as ethylene precursor for the DA/dehydration reaction with 2,5-DMF to synthesize 100% bio-based *p*-xylene (Scheme 73).

**Figure 12:**
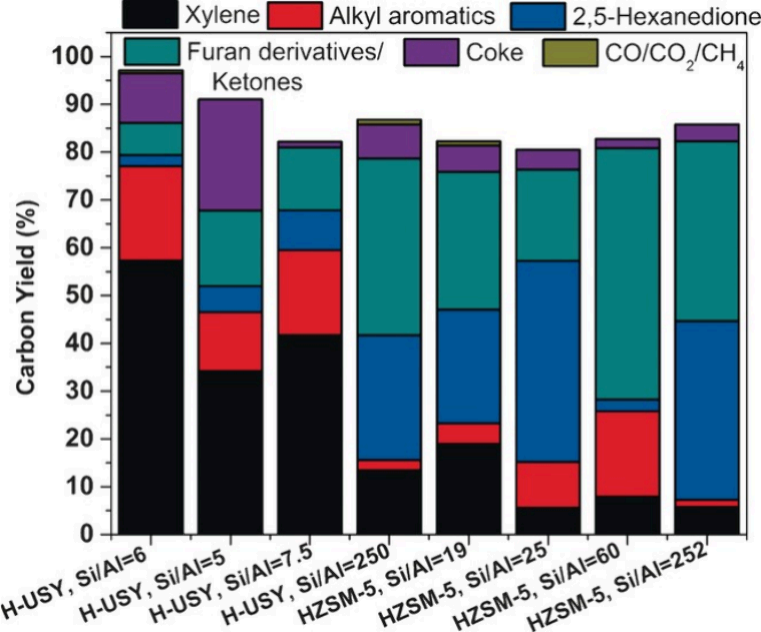
Conversion of 2,5-DMF to aromatics with ethanol catalyzed by different zeolites. Reproduced with permission from ref (332). Copyright 2016 Wiley.

In 2016, Tsang and Mpourmpakis tested HUSY and H-ZSM-5 zeolites with different Si/Al ratios for the conversion of 2,5-DMF and ethanol to *p*-xylene and alkyl aromatics at 300 °C (Figure 12).^332^ HUSY with (Si/Al = 6) exhibited the best performance, due to its intermediate acidic content and preferred pore size. Compared to ethylene, the use of ethanol resulted in a lower selectivity to *p*-xylene (67% v.s. 77%) but a higher selectivity to alkylated aromatics (23% v.s. 5%) and a higher selectivity to total aromatics (90% v.s. 82%).^332^ DFT calculations suggested that ethanol was first protonated and then dehydrated to ethylene which enabled the DA reaction of 2,5-DMF and ethylene.^332^ In 2021, Zhang’s group synthesized sulfonic acid functionalized MCM-41 catalysts (MCM-41-SO_3_H) with adjusted acidity by treatment with ammonia and used these catalysts for the same conversion.^333^ The optimization on the amount of sulfonic groups and the N content resulted in a maximum 80% selectivity to *p*-xylene at 79% 2,5-DMF conversion using *n*-heptane as solvent after 30 h reaction at 300 °C.^333^

**Figure 13:**
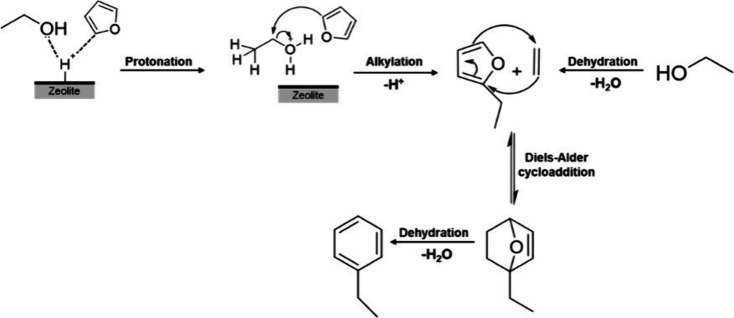
Formation of ethylbenzene from furan and ethanol through alkylation/DA/dehydration. Reproduced with permission from ref (334). Copyright 2018 American Chemical Society.

In 2018, Tsang and Mpourmpakis reported the DA/dehydration reaction of furan with ethanol as dienophile precursor and compared this reaction to the one with ethylene catalyzed by HUSY (Si/Al = 250) at 300 °C.^334^ The use of ethanol instead of ethylene significantly improved the aromatic selectivity from 36% to 67% and reduced the benzofuran selectivity from 63% to 23%. Whereas benzene was produced from furan and ethylene with 35% selectivity, the use of ethanol as pre-dienophile only generated trace amounts of benzene. Instead, ethylbenzene was observed as the main aromatic product which was formed with 45% selectivity. DFT calculations suggested that alkylation of furan by ethanol under acidic conditions produced 2-ethylfuran, which reacted with the in situ formed ethylene through DA/dehydration reaction, forming ethylbenzene as the main product (Figure 13).^334^

**Scheme 74:**
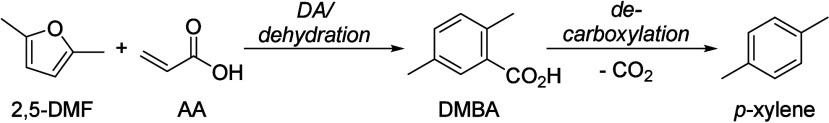
DA/Dehydration of 2,5-DMF and AA Followed by Decarboxylation to Form *p*-Xylene

Acrolein and acrylic acid (AA), obtained from renewable glycerol, both contain one vinyl group directly connected to an electron-withdrawing group (-COH, or -COOH) and serve as excellent dienophiles for Diels–Alder reactions. Addition of 2,5-DMF to AA followed by dehydration delivered 2,5-dimethylbenzoic acid (DMBA), which could then be decarboxylated to *p*-xylene (Scheme 74).

**Scheme 75:**
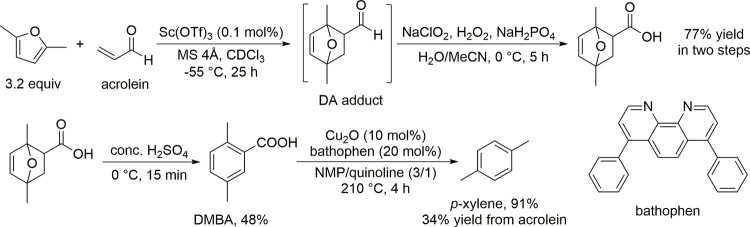
Formation of *p*-Xylene from 2,5-DMF and Acrolein by DA Addition, Oxidation, Dehydration, and Decarboxylation^335^

Toste reported a multi-step synthesis of *p*-xylene from 2,5-DMF and acrolein (Scheme 75).^335^ DA addition of acrolein and 2,5-DMF (3.2 equiv) catalyzed by Sc(OTf)_3_ in CDCl_3_ at -55 °C produced a mixture of 7-oxabicyclo[2.2.1]hept-5-ene-2-carbaldehyde isomers, which were oxidized by NaClO_2_ and H_2_O_2_ to the corresponding acids in 77% overall yield. Dehydration of the obtained acids with concentrated H_2_SO_4_ led to the formation of DMBA in 48% yield. Cu-catalyzed decarboxylation of DMBA, in the presence of 20 mol% of bathophenanthroline (bathophen), produced *p*-xylene in 91% yield. The overall yield of this multi-step strategy to *p*-xylene from acrolein was 34%.^335^

**Scheme 76:**
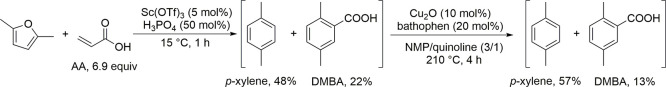
One-Pot Conversion of 2,5-DMF and AA to *p*-Xylene and DMBA^336^

**Scheme 77:**
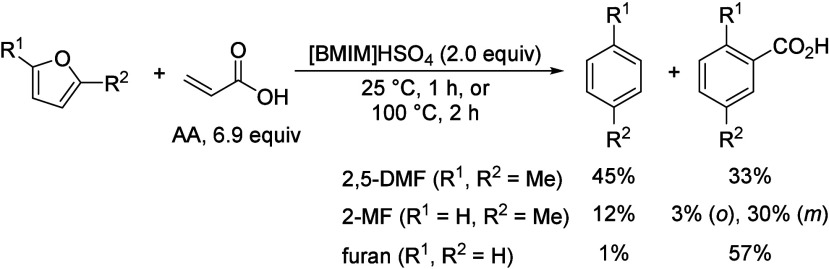
Conversion of Furans and AA to Aromatics in [BMIM]HSO_4_^337^

Zhang’s group reported the use of ionic liquids for the DA/dehydration of 2,5-DMF and acrylic acid (AA).^336^ In neat conditions, 2,5-DMF and 6.9 equiv of AA were first reacted in the presence of different metal triflates (5 mol%) at varying temperatures which produced 11–39% yields of *p*-xylene along with 13–18% yields of DMBA, delivering 24–55% total aromatic yields.^336^ The addition of 1.5 equiv of [EMIM]NTf_2_ as solvent and 50 mol% of H_3_PO_4_ improved the yield of *p*-xylene to 48% along with 22% of DMBA. To further improve the *p*-xylene selectivity, a one-pot Cu-catalyzed decarboxylation process was integrated, which improved the *p*-xylene yield to 57% (Scheme 76).^336^ The reaction of 2-MF and AA under optimized conditions afforded 12% of toluene along with *o*- and *m*-methylbenzoic acid in 2% and 9% yield, respectively. Reaction of furan with AA only produced 4% of benzene and 22% of benzoic acid.^336^ In 2018, the same group further improved this reaction by employing various ionic liquids (Scheme 77).^337^ In the presence of 2 equiv of [BMIM]HSO_4_, a 45% yield of *p*-xylene and a 33% yield of DMBA were obtained after 1 hour reaction at 25 °C.^337^ Addition of H_2_SO_4_ decreased the *p*-xylene yield to 20–42% and improved the yield of DMBA to 36–58%. The reaction of 2-MF with 6.9 equiv of AA in [BMIM]HSO_4_ at 100 °C produced toluene, *o*- and *m*-methyl-benzoic acid in 12%, 3%, and 30% yields, respectively, whereas furan was converted to benzoic acid in 57% yield under the same conditions.^337^

Zeolites, including ZSM-5, BEA, and Y zeolite, were tested as catalysts for the *p*-xylene production from 2,5-DMF and AA at 200 °C in a continuous flow reactor.^338^ ZSM-5 (Si/Al = 280) as catalyst delivered a 31.5% yield of *p*-xylene along with a 1.2% yield of DMBA, while Y zeolite (Si/Al = 80) afforded *p*-xylene in 66.9% yield and DMBA in 32.3% yield respectively. Use of a BEA zeolite with a Si/Al ratio of 150 gave a 83% yield of *p*-xylene and a 17% yield of DMBA.^338^ The good performance of the BEA zeolite was due to its catalytic ability for dehydration and decarboxylation originating from its microporous structure and medium to high acidity.^338^ Increasing the residence time from 3.04 min to 10.1 min raised the *p*-xylene yield from 59.3% to 80% and reduced the yield of DMBA from 31.7% to 14%, suggesting that *p*-xylene was formed by decarboxylation of DMBA. When a batch reactor was used instead of the flow reactor, only 40% of 2,5-DMF conversion was observed with 34% of *p*-xylene and 4% of DMBA, due to the rapid catalyst deactivation.^338^

Wu’s group prepared a heterogenous metal-organic framework Bi-BTC catalyst from bismuth(III) nitrate pentahydrate and trimesic acid. In a pressure tube, Bi-BTC catalyzed the reaction of 2,5-DMF and AA at 160 °C with acetone as solvent, resulting in 92% yield of *p*-xylene along with 4% yield of DMBA.^339^ Under the same conditions, the reaction of 2-MF with AA produced 65% yield of toluene and 23% yield of 2-methylbenzoic acid.^339^ When furan was subjected to the same conditions, benzene and benzoic acid were obtained in 37% and 43% yield respectively. Furfuryl alcohol (FA) and HMF could not be converted under these conditions.^339^

##### 2.5.3.3 Terephthalic Acid Derivatives

**Figure 14:**
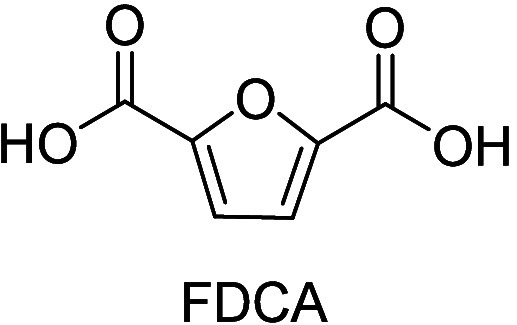
Structure of 2,5-furandicarboxylic acid (FDCA)

2,5-Furandicarboxylic acid (FDCA, Figure 14) is an outstanding bio-based chemical as it serves as an analogue of terephthalic acid (TA) and it will be used for the production of bio-based polymers, in particular poly-ethylene furan-2,5-dicarboxylate (PEF). Avantium has announced the construction of the first FDCA plant in Delfzijl, the Netherlands, and are expected to start operation in 2024 with a predicted 5000 tonnes production per year.^263^ DA/dehydration of FDCA and ethylene, expected to form TA directly, however failed due to the high electro-deficiency of FDCA. HMF, as a top “10+4” carbohydrate-originating platform chemical,^159^ has rarely been reported in DA reactions because of its instability and electron-withdrawing groups, making it an unreactive diene for DA reactions. Instead, derivatives of FDCA (FDCA esters) and HMF provided better reactivity in DA reactions with ethylene, delivering TA analogues as major product.

**Scheme 78:**
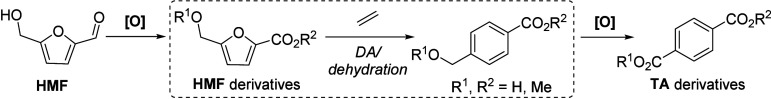
Davis’ Strategy to Synthesize TA from HMF^327,340,341^

**Scheme 79:**
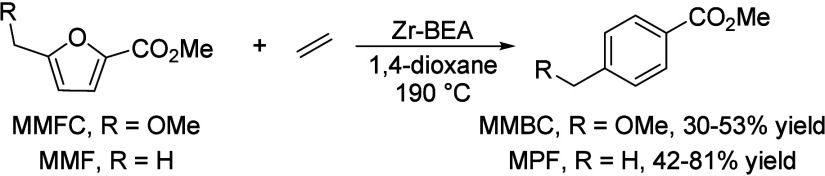
Zr-BEA-Catalyzed Conversion of MMFC to MMBC and MMF to MPF^340,341^

In 2014, Davis proposed an oxidation/DA/dehydration/oxidation strategy to synthesize TA from HMF (Scheme 78).^327^ HMF was first oxidized to 5-hydroxymethylfuroic acid which was also esterified. These two compounds were reacted with ethylene to afford 4-substituted benzoic acid or ester. Further oxidation of the obtained 4-substituted benzoic acid or ester led to the formation of TA or its half ester. However, this method only provided 5–24% yields of 4-substituted benzoic acid or esters when these substrates were reacted with 70 bar of ethylene catalyzed by Sn-BEA zeolite at 190 °C.^327^ Later, improved yields (30–53%) of methyl 4-(methoxymethyl)benzene carboxylate (MMBC) were reported by the same group from the reaction of methyl 5-methoxymethylfuran-2-carboxylate (MMFC) with 70 bar of ethylene catalyzed by Zr-BEA at 190 °C (Scheme 79).^340^ Under the same conditions, methyl *p*-toluate (MPF) was obtained from methyl 5-methyl-2-furoate (MMF) in 42–81% yields.^340^ Use of Zn-BEA as catalyst resulted in up to 28.4% yield of MMBC from MMFC in heptane after reaction at 170 °C for 18 h under 35 bar of ethylene.^341^

**Scheme 80:**
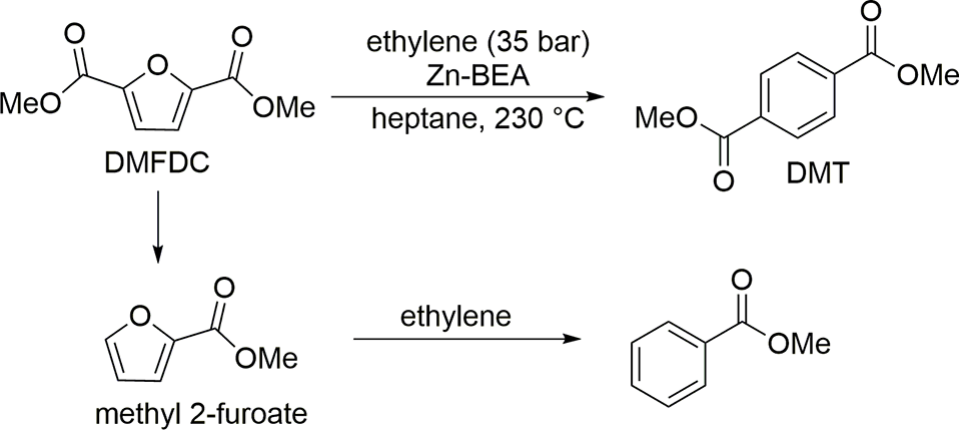
Zn-BEA-Catalyzed Conversion of DMFDC to DMT^341^

Zn-BEA catalyzed DA/dehydration of dimethyl 2,5-furandicarboxylate (DMFDC) under 35 bar ethylene and produced <15% yield of dimethyl terephthalate (DMT) along with 0–9% yield of methyl benzoate.^341^ The authors suggested that the formation of methyl benzoate was due to the decarboxylation of DMFDC to methyl 2-furoate, which reacted with ethylene and underwent dehydration (Scheme 80).^341^ However, decarboxylation of methyl esters is an unknown reaction. Methyl 2-furoate was indeed detected and no methyl benzoate was produced from DMT under the same conditions.^341^ A more plausible reaction pathway could be the hydrolysis of DMFDC to monomethyl FDCA, since water was produced during the DA/dehydration of DMFDC and ethylene. Decarboxylation of monomethyl FDCA leads to the formation of methyl 2-furoate.

Jae prepared silica-supported phosphotungtic acids (HPM-SiO_2_) with different HPW loading and studied their catalytic activity for the conversion of DMFDC to terephthalates, including DMT, diethyl terephthalate, and methyl ethyl terephthalate.^342^ Under 35 bar of ethylene, 12% HPM-SiO_2_ provided ∼50% yield of terephthalates after 6 h reaction at 225 °C. Increasing the HPW loading to 25% improved the terephthalate yield to ∼60%.^342^

**Scheme 81:**
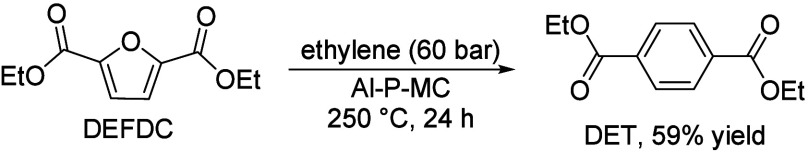
Al-P-MC-Catalyzed Conversion of DEFDC and Ethylene to DET^343^

Farmer and co-workers reported the DA/dehydration of diethyl 2,5-furandicarboxylate (DEFDC) to diethyl terephthalate (DET) catalyzed by different catalysts under 60–80 bar ethylene at 150–250 °C.^343^ Comparing all catalysts, including Al-Y, titanium silicate, and TiO_2_, use of Al-pillared montmorillonite (Al-P-MC) resulted in the highest DET yield (59%) at 250 °C under 60 bar ethylene (Scheme 81).^343^

**Scheme 82:**
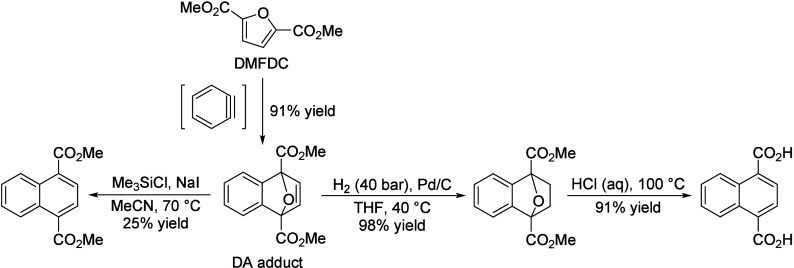
Conversion of DMFDC to Naphthalene-1,4-Dicarboxylic Acid and Dimethyl Ester^344^

**Scheme 83:**
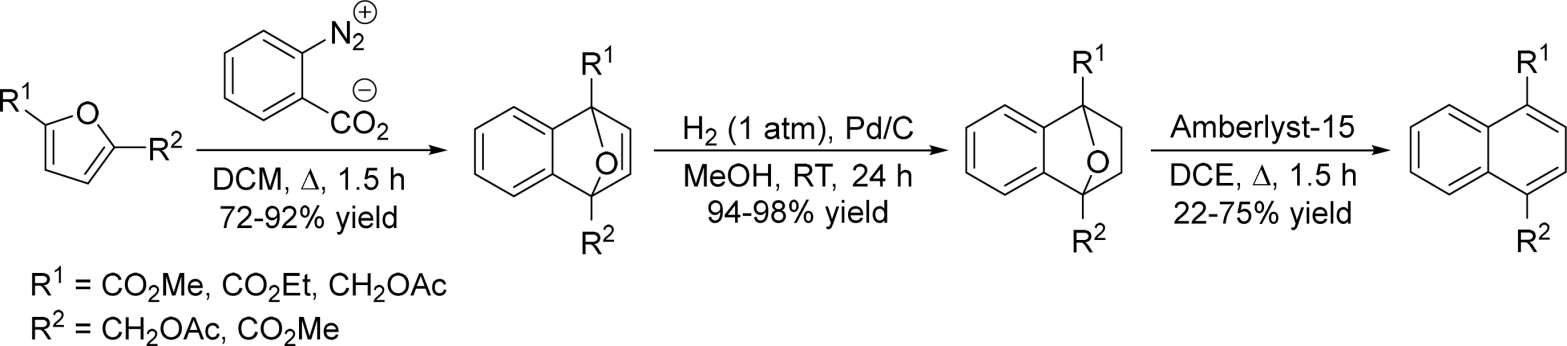
DA/Hydrogenation/Dehydration of Bio-Based Furans to Naphthalenes^345^

DA reaction of DMFDC with benzyne followed by deoxygenation at 70 °C with iodotrimethylsilane, which was formed *in situ* by reacting chlorotrimethylsilane (Me_3_SiCl) with NaI, generated dimethyl naphthalene-1,4-dicarboxylate in 25% yield (Scheme 82).^344^ Hydrogenation of the DA adduct with Pd/C under H_2_ at 40 °C followed by dehydration in an aqueous HCl solution at 100 °C produced naphthalene-1,4-dicarboxylic acids in 81% total yield (over three steps). Guided by the same strategies, 2,5-DMF was converted to 1,4-dimethylphthalene in 89% yield.^344^ The group extended the scope of this DA/hydrogenation/dehydration strategy to other bio-based furan derivatives and improved the dehydration conditions by using a catalytic amount of Amberlyst-15 (Scheme 83).^345^

##### 2.5.3.4 Benzoic Acids, Benzoates, and Benzonitriles

**Scheme 84:**
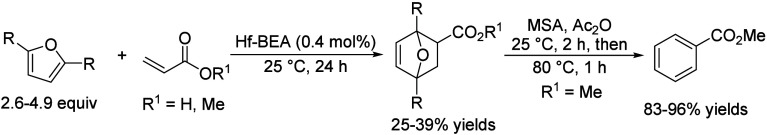
Hf-BEA-Catalyzed DA/Dehydration of Furans with AA and the Production of Methyl Benzoate^346^

Benzoic acids and benzoates are ubiquitous in pharmaceuticals, polymers, and natural products. Bio-based benzoic acids can be accessed by DA reaction/dehydration of furans and acrylic acid if decarboxylation can be prevented. As discussed in section 2.5.3.2, DMBA, toluic acid, and benzoic acid were generated along with *p*-xylene, toluene, or benzene as side products. The ratios of the benzoic acids and the corresponding decarboxylated aromatic hydrocarbons are highly dependent on the dienes, while less substituted furans show higher selectivity to benzoic acids. Lobo and co-workers reported DA reactions of furans with acrylic acids or methyl acrylate catalyzed by Hf-BEA zeolite, affording DA adducts in 25–39% yields at 25 °C after 24 h reaction (Scheme 84).^346^ Methyl benzoate was obtained in 83–96% yields by the dehydration of the DA adduct of furan and methyl acrylate, through reaction with acetyl methanesulfonate, in situ formed from methanesulfonic acid (MSA) and acetic anhydride.^346^

**Scheme 85:**
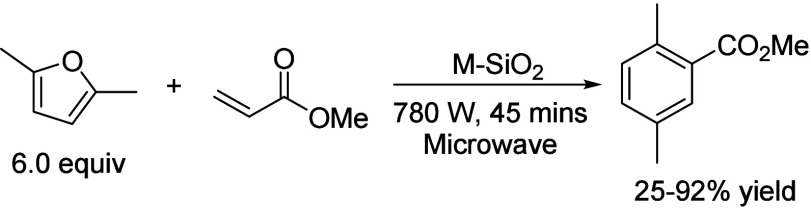
Silica-Supported Lewis Acid-Catalyzed Reaction of 2,5-DMF and Methyl Acrylate with Microwave Irradiation^347^

**Scheme 86:**
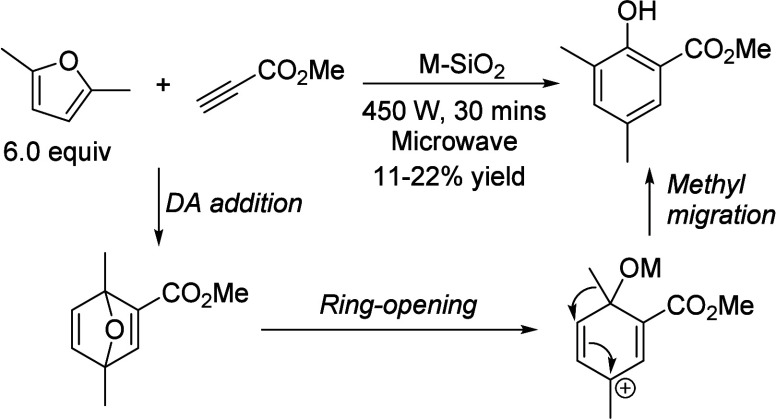
Silica-Supported Lewis Acid-Catalyzed Reaction of 2,5-DMF and Methyl Propiolate with Microwave Irradiation^348^

**Scheme 87:**
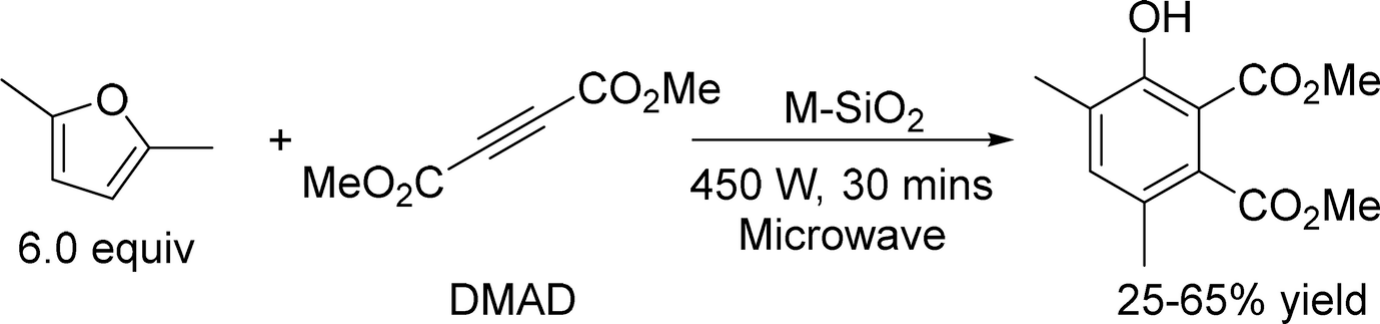
Microwave-Irradiated DA Reaction of 2,5-DMF and DMAD Catalyzed by Silica-Supported Lewis Acids^348^

Moreno reported the use of microwave irradiation and silica-supported Lewis acid catalysts, including ZnCl_2_, Et_2_AlCl, and TiCl_4_ for the DA/dehydration of 2,5-DMF and methyl acrylate under solvent-free conditions which resulted in the formation of methyl 2,5-dimethylbenzoate in 25–92% yields (Scheme 85).^347^ Reaction of 2,5-DMF with acrylonitrile under the same conditions produced 2,5-dimethylbenzonitrile in 23–50% yields.^347^ The same approach was also applied for benzoate production from 2,5-DMF and methyl propiolate. Interestingly, methyl 3,5-dimethyl-2-hydroxybenzoate was obtained in 11–22% yields, probably due to methyl group migration (Scheme 86).^348^ Similarly, reaction of 2,5-DMF and DMAD under these conditions delivered dimethyl 3-hydroxy-4,6-dimethylphthalate in 25–65% yields (Scheme 87).^348^

**Scheme 88:**
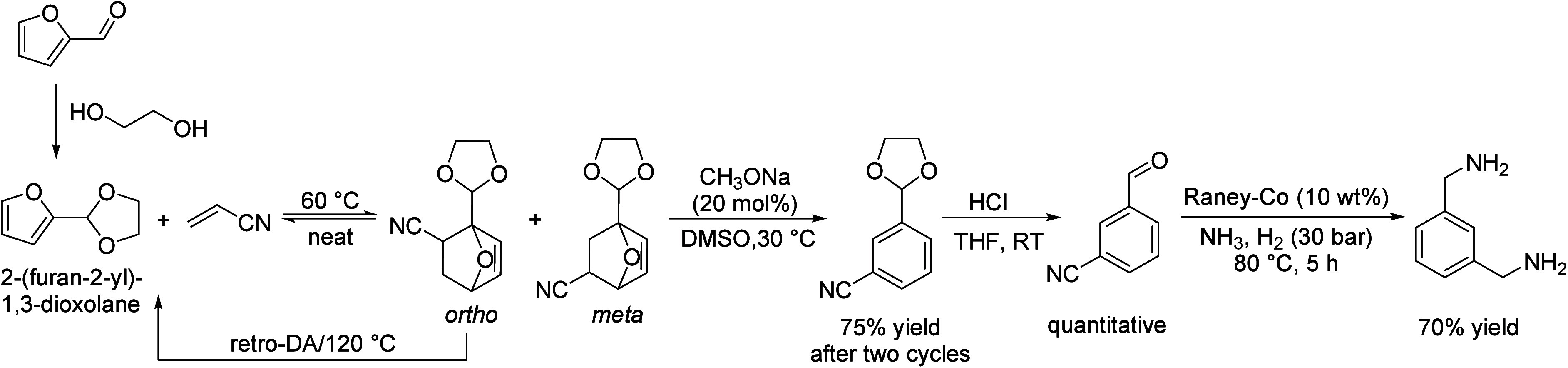
Production of *m*-Xylylenediamine from Furfural and Acrylonitrile^349^

*m*-Xylylenediamine is frequently used in industry as curing agent and polymer precursor. In 2018, Jérôme reported its production from the DA reaction of ethylene glycol-protected furfural, 2-(furan-2-yl)-1,3-dioxolane, with acrylonitrile (Scheme 88).^349^ 2-(Furan-2-yl)-1,3-dioxolane reacted with acrylonitrile at 60 °C under neat conditions to produce both *ortho*- and *meta*-adducts. A kinetic study of the dehydration reaction under basic conditions indicated that the *ortho*-adducts started to react only after the *meta*-adduct was almost consumed. Thus, the authors quenched the dehydration reaction after 50% conversion and converted the remaining *ortho*-adduct at 120 °C under vacuum to 2-(furan-2-yl)-1,3-dioxolane and acrylonitrile, which were re-subjected to the DA and dehydration conditions. After two cycles, *m*-(1,3-dioxolan-2-yl)benzonitrile was obtained in 75% yield and deprotected by HCl to *m*-formylbenzonitrile in quantitative yield. Reductive amination of *m*-formylbenzonitrile catalyzed by Raney-Co at 80 °C afforded *m*-xylylenediamine in 70% yield.^349^

**Scheme 89:**
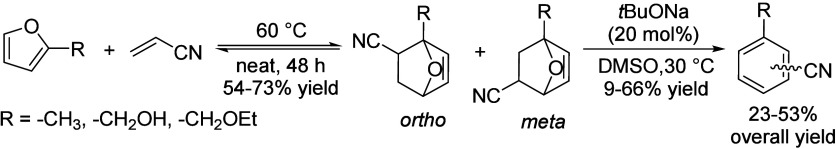
DA/Dehydration Reactions of Furans and Acrylonitrile to Benzonitriles^349^

Reactions of bio-based furans and acrylonitrile under neat conditions followed by dehydration catalyzed by 20 mol% of *t*BuONa produced *m*-and *o*-substituted benzonitriles in 23–53% overall yields (Scheme 89).^349^ Other diols and ethane-1,2-dithiol where also used to protect furfural. These compounds underwent the DA reactions with acrylonitrile catalyzed by 10 mol% ZnCl_2_, affording the corresponding DA adducts in 67–85% yields.^350^ The aromatization reaction was not described.^350^

##### 2.5.3.5 Phthalic Acids, Phthalic Anhydride, Phthalimide, and Phthalides

**Scheme 90:**
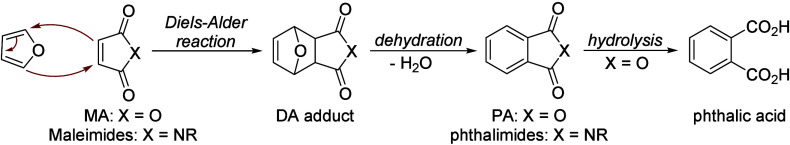
PA or phtahlimides from DA/dehydration reactions of furan with MA or maleimides

Phthalic anhydrides (PAs) and phthalic acids are important industrial chemicals for the large-scale production of plasticizers, plastics, and polymers. The production of bio-based PAs can be achieved through the Diels–Alder reaction of furans with maleic anhydride (MA), which can be obtained in renewable form via oxidation of furfural.^351,352^ Hydrolysis of the PAs gives the corresponding phthalic acids (Scheme 90). The use of maleimides as dienophile instead of MA, leads to the formation of phthalimides.

**Scheme 91:**
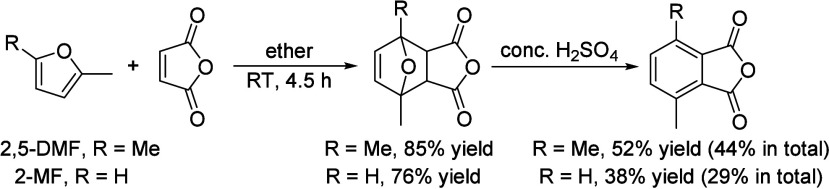
DA reaction of MA with 2,5-DMF or 2-MF followed by dehydration with H_2_SO_4_^356^

The first report on the DA addition between furan and MA can be traced back to 1929, when Diels and Alder found 7-oxobicyclo[2.2.1]hept-5-ene to be the main product of this reaction.^353^ A paper in 1933 reported the formation of 3-methylphthalic anhydride (3-methyl-PA) from the reaction of 2-MF and MA followed by dehydration with HBr and acetic acid. However, no yield was given.^354^ In 1944 and 1964, Newman reported the use of concentrated sulfuric acid for the dehydration of the DA adduct from the reaction between MA and 2,5-DMF or 2-MF, affording 3,6-dimethyl-PA and 3-methyl-PA in 52% and 38% yields, respectively (Scheme 91).^355,356^ The yield of 3-methyl-PA was further improved to 66% by mixing the DA adduct with the solution of H_2_SO_4_ in sulfolane at low temperature (-55 to -45 °C) and reacting 3 h at −55 to −45 °C and 3 h at −45 to 26 °C.^357^ Increasing the mixing temperature to 0 °C produced a 25% yield of 3-methylphthalic acid along with 1% of 3-methyl-PA.^357^

**Scheme 92:**
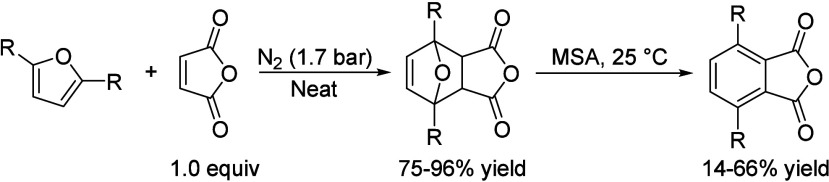
DA reaction of furans and MA, followed by dehydration with MSA^358^

Reacting furans with 1.0 equiv of MA neat under 1.7 bar of N_2_, generated DA adducts in 75–96% yields.^358^ Dehydration of DA adducts in methanesulfonic acid (MSA) at 25 °C provided 3,6-dimethyl-PA, 3-methyl-PA, and PA in 66%, 48%, and 14% yields respectively (Scheme 92).^358^ Addition of acetic anhydride raised the PA yield to 80% (along with 7% of phthalic acid) after 2 h reaction at 25 °C and 4 h reaction at 80 °C.^358^

**Scheme 93:**
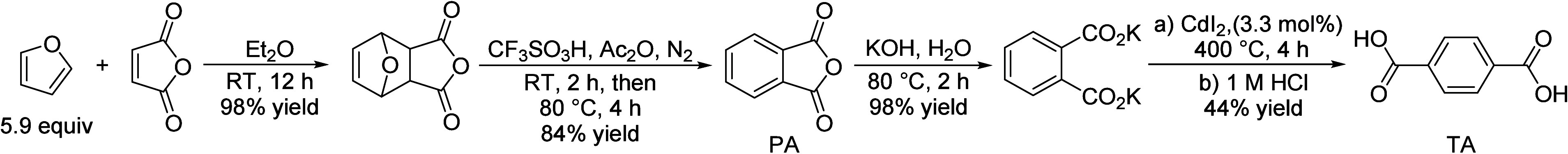
Synthesis of TA from Furan and MA via PA and Dipotassium Phthalate^359^

Kasuya’s group described the use of CF_3_SO_3_H and Ac_2_O to dehydrate the DA adduct of furan and MA, producing PA in 84% yield.^359^ Hydrolysis of PA with KOH formed dipotassium phthalate in 98% yield, which was converted to terephthalic acid in 44% yield catalyzed by CdI_2_ at 400 °C followed by acidification (Scheme 93).^359^

**Scheme 94:**
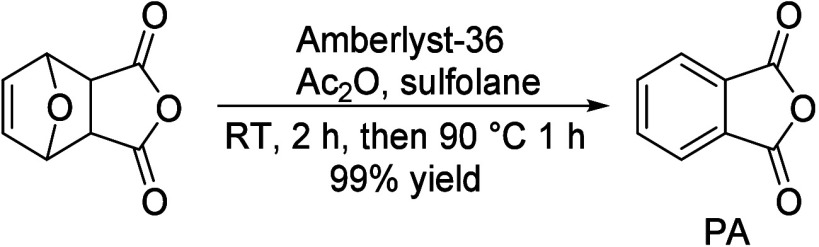
Amberlyst-36-Catalyzed Dehydration to PA^360^

Deng and co-workers achieved a 99% yield of PA from the DA adduct by treatment with Amberlyst-36 as catalyst for 2 h reaction at room temperature followed by 1 hour reaction at 90 °C (Scheme 94).^360^ Regenerated Amberlyst-36 showed a decreased yield (from 99% to 48%) in the second recycling test due to the destruction of the resin skeleton.^360^

**Scheme 95:**
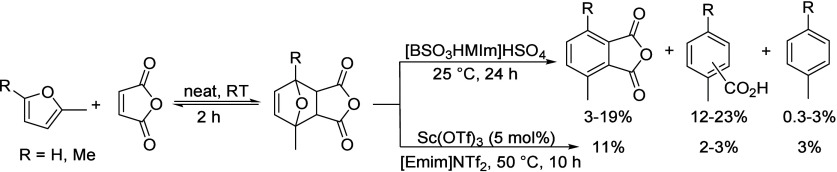
Dehydration of DA Adducts with [BSO_3_HMIm]HSO_4_ as Solvent^336,337^

Use of ionic liquids as solvent for the dehydration of DA adducts obviates the need for strong mineral acids.^336,337^ Using Sc(OTf)_3_ as catalyst for the reaction between 2,5-DMF and MA in [Emim]NTf_2_ at 50 °C afforded an 11% yield of 3,6-dimethyl-PA and 3-methyl-PA from 2,5-DMF and 2-MF respectively.^336^ Use of [BSO_3_HMIm]HSO_4_ (1-butylsulfonate-3-methylimidazolium hydrogen sulfate) as solvent at 25 °C, resulted in formation of 3,6-dimethyl-PA from 2,5-DMF in 19% yield and 3-methyl-PA from 2-MF in 3% yield, respectively (Scheme 95).^337^ In addition to benzoic acids, small amounts of *p*-xylene and toluene were also observed as side products.^337^

**Scheme 96:**
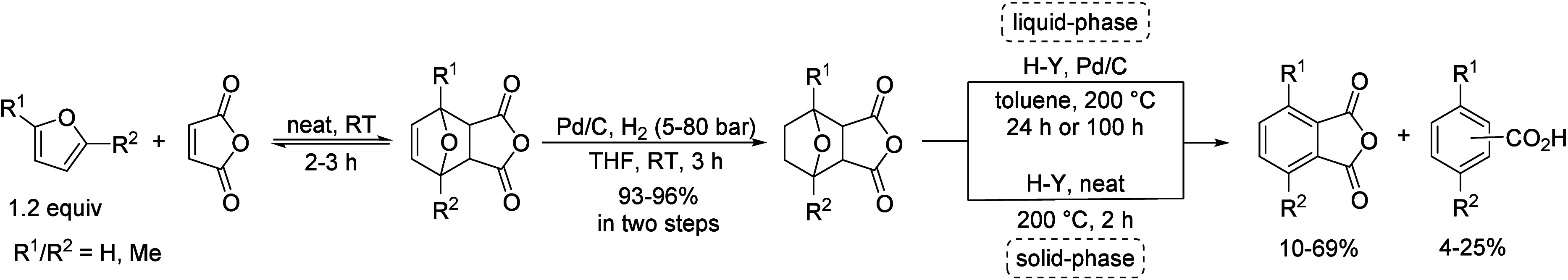
Three-Step Strategy to PAs from Furans and MA^361,362^

**Scheme 97:**
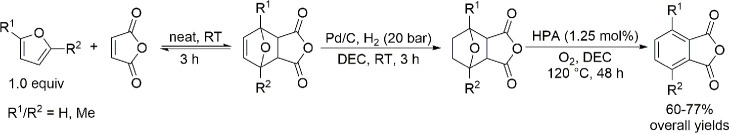
HPA-Catalyzed One-Step Dehydration and Dehydrogenation to PA^363^

To address the general issue of the reversible nature of the intermediate DA addition step, van Es and Bruijnincx reported a modified three-step strategy, wherein the DA cycloadducts were first hydrogenated to the stable oxabicyclo[2.2.1]heptane intermediates catalyzed by Pd/C. These intermediates were then converted to PAs and benzoic acids in a one-pot dehydration and dehydrogenation reaction (Scheme 96).^361,362^ The last step was achieved under both liquid-phase and solid-phase conditions at 200 °C catalyzed by H-Y zeolite with Pd/C, affording PAs in 10–69% yields along with 4–25% of benzoic acids.^361,362^ In 2020, Li’s group further improved this three-step strategy by employing heteropoly acid H_4_SiMo_12_O_40_ (HPA) and O_2_ with diethyl carbonate (DEC) as solvent for the last step which led to the production of PAs in 60–77% overall yields (Scheme 97).^363^ 2,5-DMF and *N*-methylmaleimides treated under the same conditions, using dibutyl carbonate as solvent at 150 °C resulted in the formation of 3,6-dimethylphthalimide in 92% overall yield.^363^

**Scheme 98:**
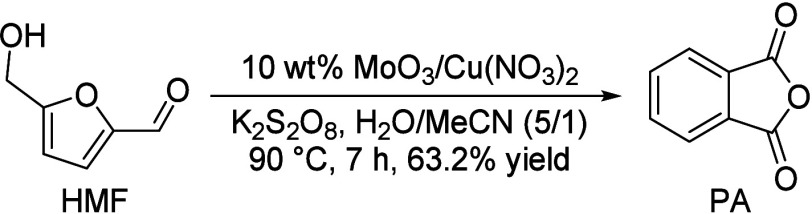
Direct Conversion of HMF to PA^364^

As furan and MA both can be produced from HMF, Sun’s group developed a direct conversion of HMF to PA with MoO_3_ and Cu(NO_3_)_2_ as catalysts in 63.2% yield at 90 °C using K_2_S_2_O_8_ as oxidant (Scheme 98).^364^ 2,5-Diformylfuran (DFF) under the same conditions produced a 77.2% yield of PA.

**Scheme 99:**
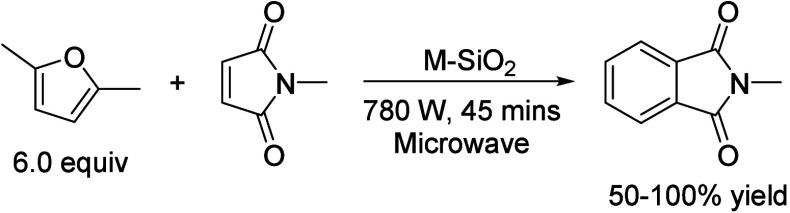
Microwave-assisted DA/dehydration of 2,5-DMF and *N*-methylmaleimides^347^

**Scheme 100:**
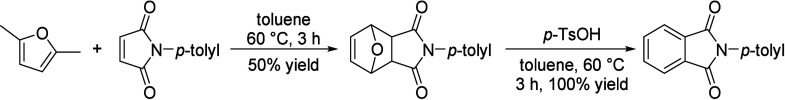
Synthesis of *N*-(*p*-tolyl)phthalimide from 2,5-DMF and N-(*p*-tolyl)maleimide^365^

Replacing MA with maleimides as dienophile in the DA/dehydration reaction with furans produces phthalimides. Microwave-irradiated DA/dehydration reaction between 2,5-DMF and *N*-methylmaleimide catalyzed by silica-supported Lewis acid catalysts, such as ZnCl_2_, Et_2_AlCl, and TiCl_4_, produced 50–100% yields of *N*-methyl phthalimides (Scheme 99).^347^ The DA reaction of *N*-(*p*-tolyl)maleimide with 2,5-DMF in toluene followed by dehydration with *p*-toluenesulfonic acid gave a 50% yield of *N*-(*p*-tolyl)phthalimide (Scheme 100).^365^

**Scheme 101:**
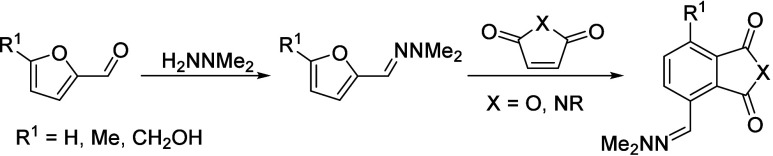
DA reactions of furfural dimethylhydrazines with MA or maleimides

Furfural, HMF, 2-methylfurfural, and furoic acid are usually less active in DA reactions, due to their electro-deficient nature. Protecting the aldehyde groups in furfural and HMF with 1,1-dimethylhydrazine allows its DA reaction with dienophiles (Scheme 101). Potts and Walsh used chloroform as solvent for the reactions of furfural dimethylhydrazine with MA or *N*-substituted maleimides and produced a range of 4-(2,2-dimethylhydrazineylidene)phthalic anhydrides or phthalimides in 65–94% yields.^366^ A microwave-irradiated approach with [BMIM]Cl as solvent was developed by Sheppard and Kamimura for this conversion which afforded a range of *N*-substituted phthalimides in 34–97% yields after 2 h reactions.^367^

**Scheme 102:**
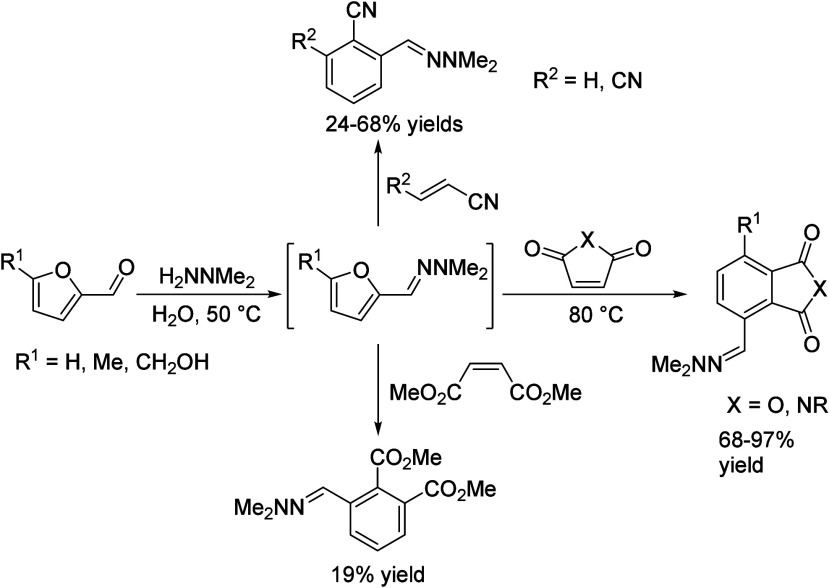
One-pot conversion of furfurals to aromatics with dimethylhydrazine as protecting group^368^

In 2016, Hailes and Sheppard achieved one-pot conversions following this strategy by the direct addition of various *N*-substituted maleimides to the reaction mixture of furfurals and 1,1-dimethylhydrazine in water after reaction at 50 °C for 30 min, affording the corresponding phthalimides in 68–97% yields (Scheme 102).^368^ Fumaronitrile, acrylonitrile, and dimethyl maleate were used as dienophiles in this reaction, which produced the corresponding aromatics in 19–68% yields.^368^

**Scheme 103:**
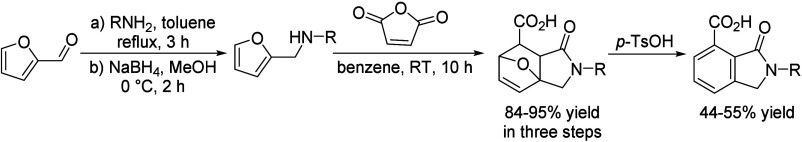
Synthesis of 3-oxoisoindoline-4-carboxylic acids from furfural^369^

**Scheme 104:**
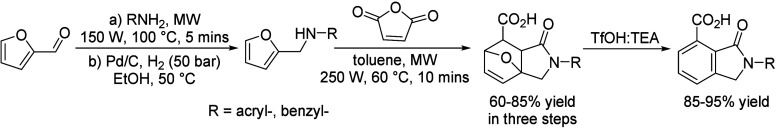
Synthesis of 3-oxoisoindoline-4-carboxylic acids from furfural (372)

*N*-Alkyl or *N*-aryl furfural amines, produced by the reductive amination of furfural with alkylamines, reacted with MA at room temperature via DA addition and a subsequent intramolecular condensation reaction produced cycloadducts in 84–95% yields (Scheme 103).^369,370^ Dehydration of the cycloadducts with *p*-TsOH delivered *N*-substituted 3-oxoisoindoline-4-carboxylic acids in 44–55% yields,^369^ while 22–56% yields were obtained using H_3_PO_4_.^370^ The use of aqueous NaOH solution enabled the conversion of the cycloadducts to *N*-substituted 3-oxoisoindoline-4-carboxylic acids in 30–65% yields.^371^ Microwave-irradiated reductive amination followed by reaction to MA in one-pot produced the cycloadducts in 60–85% yields.^372^ Dehydration of the cycloadducts with protic ionic liquid TfOH:TEA generated *N*-substituted 3-oxoisoindoline-4-carboxylic acids in 85–95% yields (Scheme 104).^372^

**Scheme 105:**
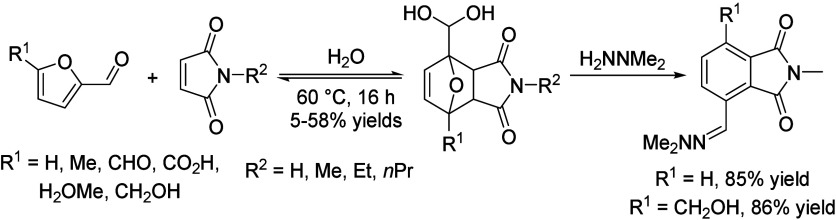
DA reactions of furfurals and *N*-substituted Maleimides in Water^373^

Bruijnincx reported the DA reaction of renewable furfurals with *N*-alkyl-maleimides in water at 60 °C to form 5–58% yields of substituted DA adducts, wherein the aldehyde group was present in its hydrate form (Scheme 105).^373^ Treating the DA adducts with 1,1-dimethylhydrazine delivered *N*-methyl-phthalimides in 85–86% yields.^373^

**Scheme 106:**
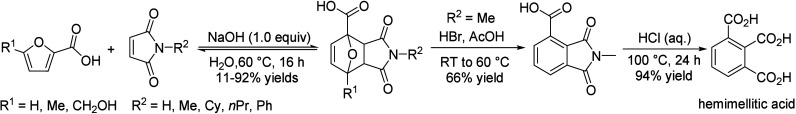
Conversion of furoic acids to hemimellitic acid via 3-carboxy-*N*-methylphthalimide^374^

Furoic acids reacted with maleimides in aqueous solution in the presence of 1.0 equiv of NaOH to produce a range of cycloadducts in 11–92% yields.^374^*N*-methyl-1,3-dioxoisoindoline-4-carboxylic acid was obtained in 66% yield by dehydration of the corresponding DA adduct with HBr in AcOH. This product was further converted to hemimellitic acid in aqueous HCl at 100 °C in 94% yield (Scheme 106).^374^

**Scheme 107:**
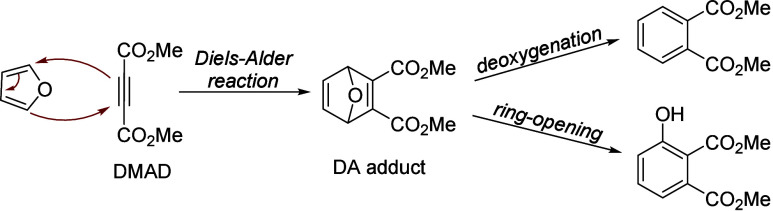
DA reactions of furans and DMAD to produce phthalates by deoxygenation of ring-opening

**Scheme 108:**
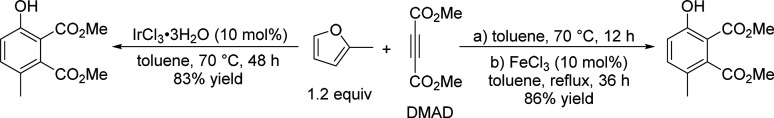
IrCl_3_ and FeCl_3_-Catalyzed Raction of 2-MF and DMAD^378^

**Scheme 109:**
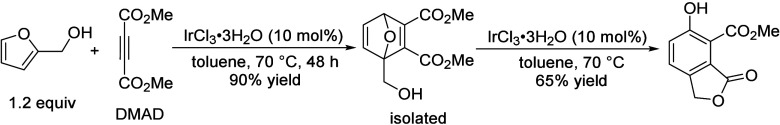
Formation of 6-Hydroxyphthalide from Furfuryl Alcohol and DMAD^378^

The DA reaction of furan with dimethyl acetylenedicarboxylate (DMAD) generates dimethyl 7-oxabicyclo[2.2.1]hepta-2,5-diene-2,3-dicarboxylate, which was converted to dimethyl phthalate through deoxygenation or to dimethyl 3-hydroxyphthalate via ring-opening aromatization (Scheme 107).^375−377^ In 2011, Sonoda reported the DA reaction of 2-MF and DMAD catalyzed by IrCl_3_ in toluene at 70 °C, which produced dimethyl 6-methyl-3-hydroxy-phthalate in 83% yield (Scheme 108).^378^ A one-pot conversion with FeCl_3_ as catalyst provided an 86% yield of dimethyl 6-methyl-3-hydroxy-phthalate.^378^ Furfuryl alcohol reacted with DMAD in toluene catalyzed by IrCl_3_ to produce the cycloadduct in 90% isolated yield. Methyl 5-hydroxy-3-oxo-1,3-dihydroisobenzofuran-4-carboxylate was obtained in 65% yield by subjecting this pure cycloadduct to the same conditions (Scheme 109).^378^

**Scheme 110:**
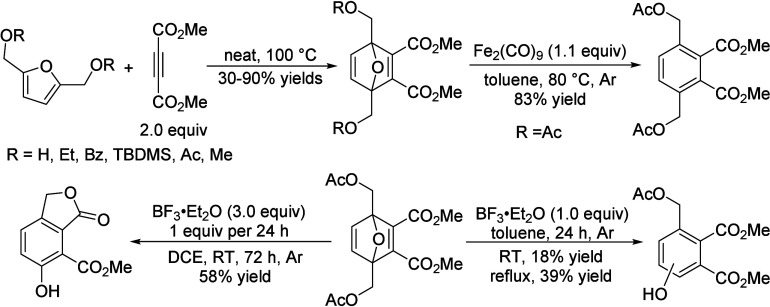
DA reactions of FA or ethers with DMAD and the conversion to phthalates or phthalide^379^

Ananikov’s group reported the DA reaction between DMAD and 2,5-bis(hydroxymethyl)furan and ethers and esters thereof under neat conditions in 30–90% isolated yields (Scheme 110).^379^ The aromatization was exemplified with the cycloadduct of DMAD with 2,5-bis(hydroxymethyl)furan diacetate, which was reduced with a stoichiometric amount of Fe_2_(CO)_9_ to dimethyl 3,6-bis(acetoxymethyl)phthalate in 83% yield.^379^ Treating the cycloadduct with a stoichiometric amount of BF_3_·Et_2_O under Ar in DCE at RT formed 6-hydroxyphthalide in 58% yield, while hydroxyl-substituted dimethyl phthalates were obtained in 18% yield at room temperature and in 39% yield under reflux using toluene as solvent.^379^

**Scheme 111:**
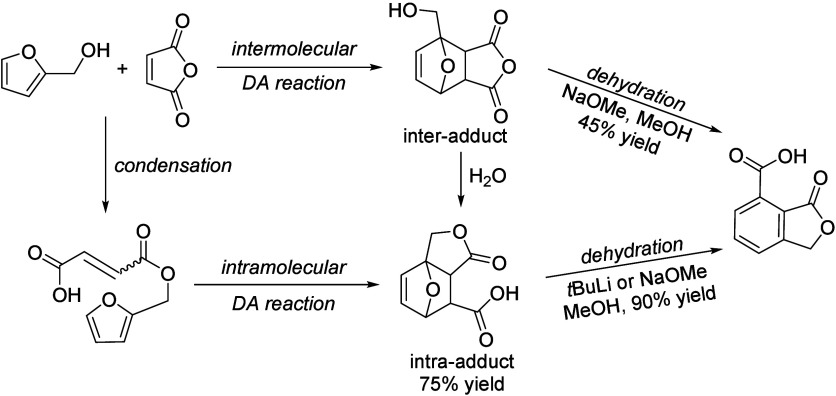
Reactions of furfuryl alcohol and MA via two pathways^380^

Reaction of furfuryl alcohol (FA) and MA can produce two possible products, the intermolecular DA adduct or the condensation product, which undergoes an intramolecular DA reaction to form the intra-adduct (Scheme 111).^380^ Moisture catalyzes the conversion of the inter-adduct into the intra-adduct, resulting in a 75% yield of the intra-adduct from FA and MA. Dehydration of the intra-adduct under basic conditions leads to 90% yield of phthalide-4-carboxylic acid, while only 45% yield could be obtained from the inter-adduct.^380^

**Scheme 112:**
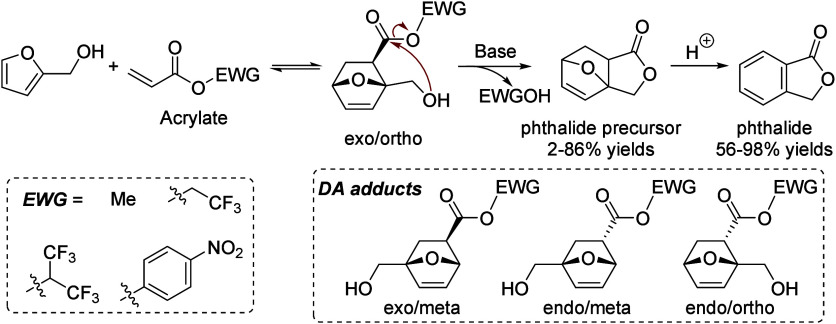
Formation of phthalides from furfuryl alcohol and activated acrylates^381^

Bruijnincx designed a route to phthalide from furfuryl alcohol (FA) and electron-withdrawing activated esters of acrylic acid (Scheme 112).^381^ Of the four possible stereoisomeric DA adducts from FA and acrylates, only the exo/ortho isomer is capable of an intramolecular reaction under basic conditions which releases one molecule of alcohol and produces the phthalide precursor. The irreversible removal of alcohol funneled the equilibrium to the exo/ortho isomer and provided up to 86% yield of the phthalide precursor, which was dehydrated with acids to phthalide in 56–98% yields.^381^ 7-Hydroxymethylphthalide was obtained from HMF in 36% yield using the same strategy.^381^

##### 2.5.3.6 Diels–Alder/Dehydration Reactions of Furans and Other Dienophiles

**Scheme 113:**
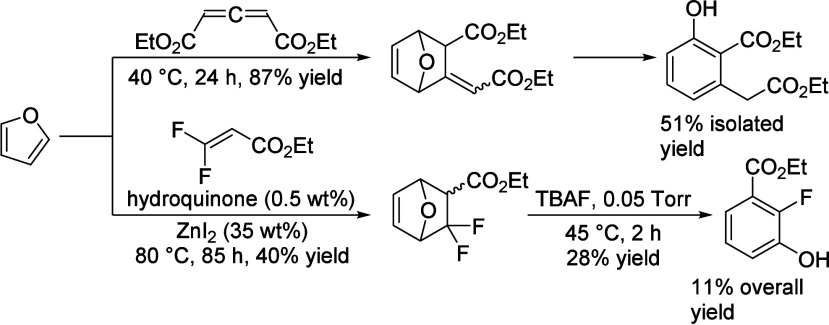
DA reactions of furan to generate aromatics^382,383^

In 1977, Kozikowski isolated ethyl 2-(2-ethoxy-2-oxoethyl)-6-hydroxybenzoate in 51% yield from the reaction of furan and 1,3-diethoxycarbonylallene at 40 °C after column chromatography (Scheme 113).^382^ Ethyl 2-fluoro-3-hydroxybenzoate was obtained from the reaction of furan and ethyl 3,3-difluoroacrylate in the presence of hydroquinone followed by treatment with tetra-*n*-butylammonium fluoride (TBAF).^383^ The addition of ZnI_2_ in the first step improved the isolated yield of DA adduct from 20% to 40%.^383^

**Scheme 114:**
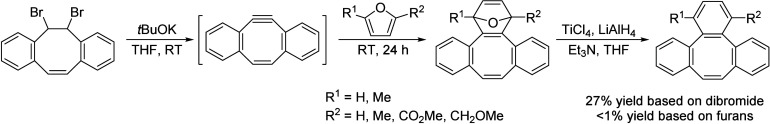
Production of tribenzo[a,c,e]annulenes from furans^384^

**Scheme 115:**
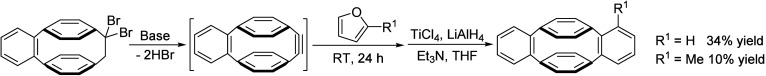
Production of dibenzo[2,2]paraclophanes from furans via DA/deoxygenation^385^

DA/dehydrations of 5,6-didehydrodibenzo[a,e]cyclooctatetraene, which was formed *in situ* by double dehydrobromination of 5,6-dibromo-5,6-dihydrodibenzo[a,e][8]annulene, with 2,5-disubstituted furans was reported to form tribenzo[*a,c,e*]cyclo-octatetraenes in 27% overall yield. However, the yields based on the furans were less than 1% due to the excess amount of furans required in these reactions (Scheme 114).^384^ Likewise, the double dehydrobromination of 9,9-dibromo-9,10-dihydro-5,8:11,14-diethenobenzo[12]annulene under basic conditions generated the alkyne containing cyclophane, which reacted with furan or 2-MF to give the cycloadducts, which were deoxygenated with TiCl_4_ and LiAlH_4_, affording dibenzo[2,2]paraclophanes in 34% and 10% overall yields respectively (Scheme 115).^385^

##### 2.5.3.7 Fine Aromatic Chemicals through Intramolecular Diels–Alder/Dehydration Reactions

**Scheme 116:**
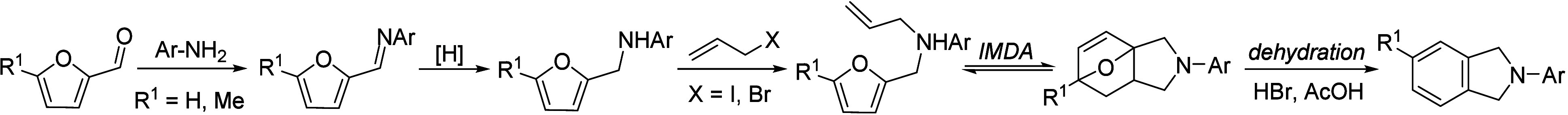
*N*-aryl-isoindolines from *N*-aryl-furfuralamines^386−390^

Intramolecular Diels–Alder reaction (IMDA) followed by dehydration can be a valuable approach for the construction of oxygen-containing six-membered ring systems and aromatic chemicals. Hahn and Jakopčić described the formation of *N*-aryl-isoindolines from *N*-aryl-furfurylamines by reaction with 3-iodopropene followed by an IMDA reaction and acid-catalyzed dehydration (Scheme 116).^386−390^ However, yields were not given.

**Scheme 117:**
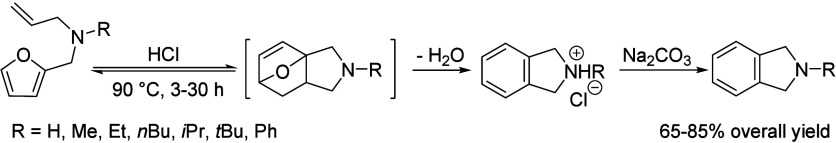
Production of dihydroisoindolines from *N*-allyl-2-furfurylamines^391^

In the presence of HCl, *N*-allyl-2-furfurylamines were converted in one step at 90 °C to dihydroisoindolinium chlorides, which were neutralized by Na_2_CO_3_ to form dihydroisoindolines in 65–85% overall yield (Scheme 117).^391^

**Scheme 118:**
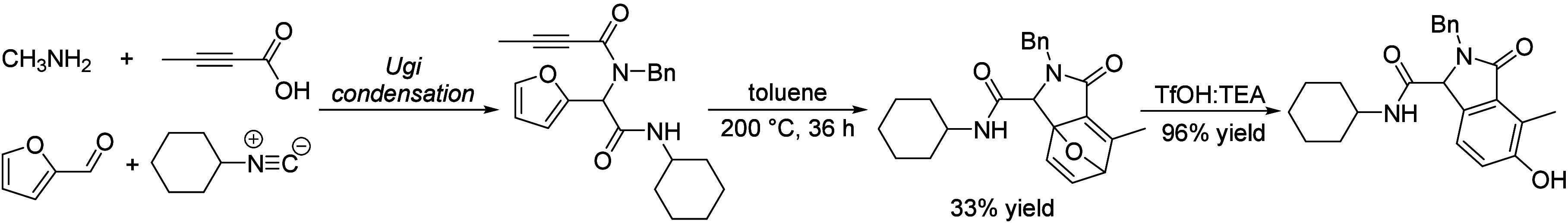
Synthesis of highly functionalized 5-hydroxy-3-oxoisoindolone from furfural through Ugi condensation followed by IMDA^372^

Ugi condensation of furfural, benzylamine, 2-butynoic acid, and cyclohexyl isocyanide delivered the Ugi condensation product, which is nicely set up for the IMDA to the cycloadduct that was obtained in in 33% yield (Scheme 118).^372^ Ring-opening of the cycloadduct in a protic ionic liquid TfOH:TEA produced the substituted 5-hydroxy-3-oxoisoindolone in 96% yield.^372^

**Scheme 119:**
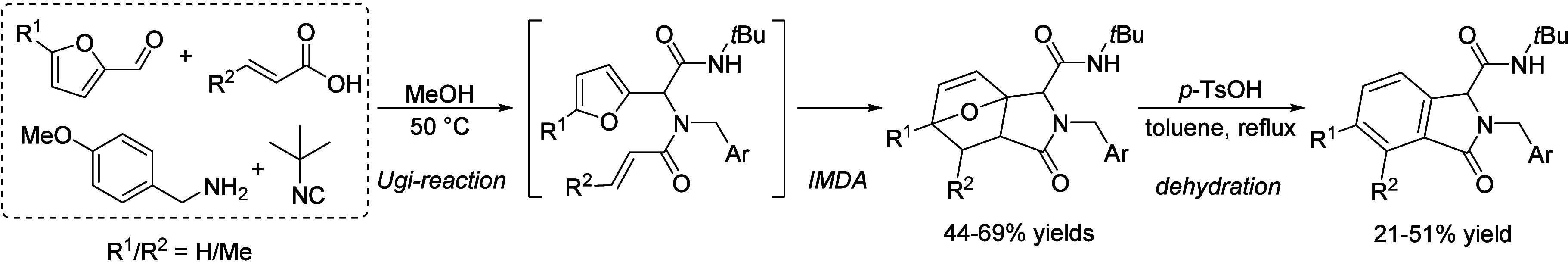
Tandem Ugi-IMDA reactions of furfurals to substituted isoindolinones^392^

Tandem Ugi-IMDA reactions of furfurals, *p*-methoxy-benzylamine, acrylic acid, and *tert*-butyl-isocyanide in methanol at 50 °C afforded 44–69% yields of the cyclo-adducts, which were dehydrated to highly substituted isoindolinones by *p*-TsOH in 21–51% yields with toluene as solvent (Scheme 119).^392^ Ivachtchenko reported a similar conversion for 5-methylfurfural, wherein microwave-assisted dehydration with BF_3_·Et_2_O as catalyst was utilized for the dehydration step, which gave the corresponding product in 75% yield.^393^

**Scheme 120:**
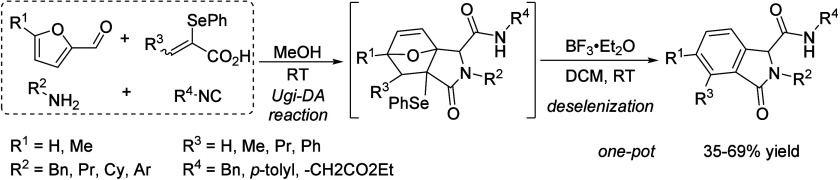
One-pot conversion of furfurals to isoindolinones through Ugi/DA/deselenization^394^

Ugi/DA reactions of furfural, amines, isocyanides, and 2-(phenylselenyl)acrylic acids in methanol followed by deselenization with BF_3_·Et_2_O produced the substituted isoindolinones in 35–69% yields (Scheme 120).^394^

**Scheme 121:**
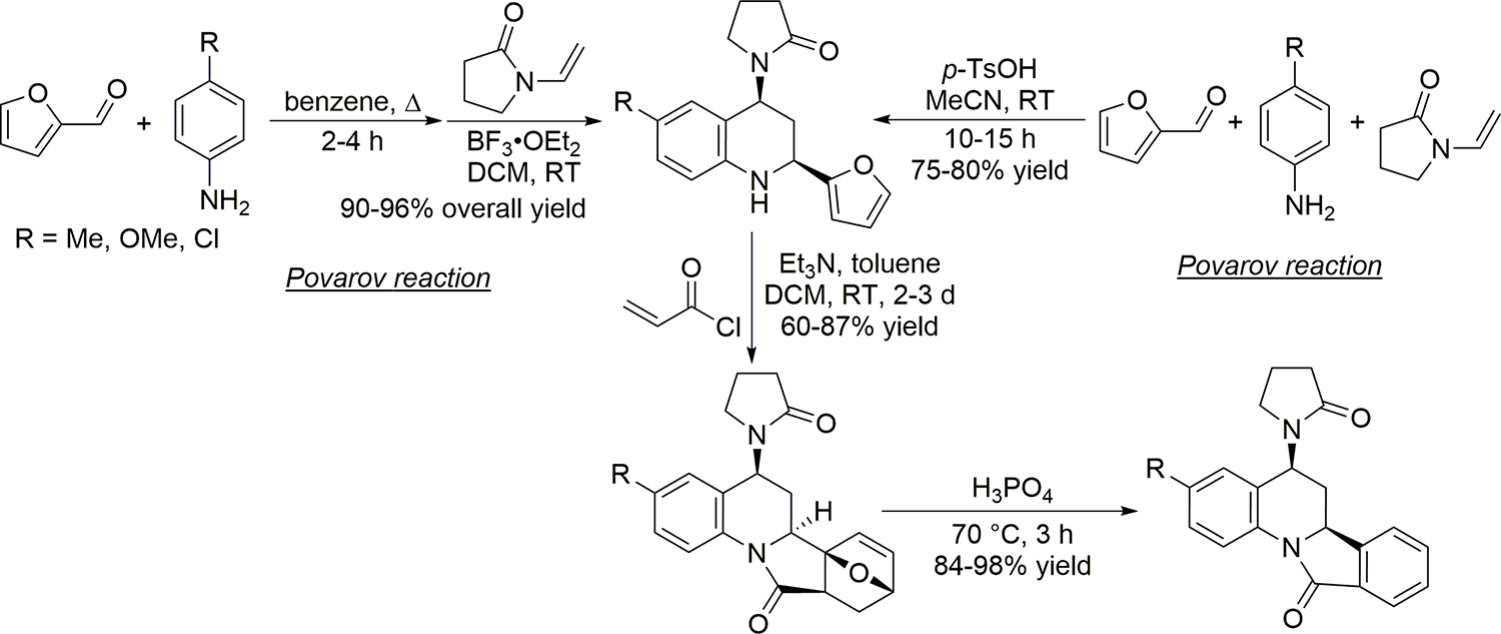
Syntheses of isoindolo[2,1-a]quinoline derivatives from furfural^395^

Povarov reaction of furfural with amines and *N*-vinylpyrrolidinone under acidic conditions afforded substituted tetrahydroquinolines in 90–96% yield using a two-step approach or in 75–80% yield from a one-step reaction.^395^ These susbtituted tetrahydroquinolines were reacted with acryloyl chloride in the presence of Et_3_N resulting in 60–87% yields of the IMDA adducts. Dehydration of the IMDA adducts with H_3_PO_4_ at 70 °C afforded the substituted isoindolo[2,1-*a*]quinolines in 84–98% yields (Scheme 121).^395^

**Scheme 122:**
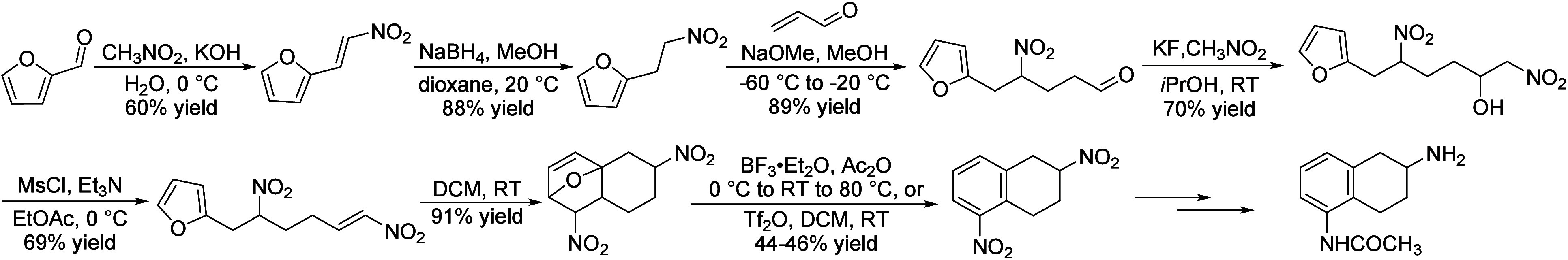
Synthesis of 2,5-dinitro-tetrahydronaphthalene from furfural^396^

A multi-step reaction to 2,5-dinitro-tetrahydronaphthalene was reported with furfural, nitromethane, and acrolein as starting materials by multiple Henry reactions, IMDA, and dehydration reactions in 9% overall yield (Scheme 122).^396^ 2,5-Dinitro-tetrahydronaphthalene could be further converted to 5-acetylamino-2-amino-tetrahydronaphthalene.^396^

**Scheme 123:**
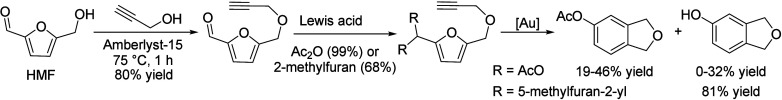
Syntheses of phthalanes from HMF by gold-catalyzed IMDA/dehydration^397^

**Scheme 124:**
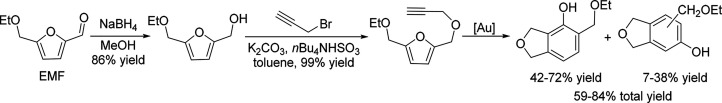
Conversion of EMF to phthalanes^397^

Hashmi described the preparation of 5-hydroxyphthalane from HMF. Amberlyst-15-catalyzed etherification of HMF in propargyl alcohol as solvent at 75 °C formed the HMF-propargyl ether in 80% yield (Scheme 123).^397^ In a flow-reactor, the reaction of HMF with 10 equiv of propargyl alcohol in ethyl acetate, produced 78% yield of HMF-propargyl ether along with 15% of EMF (5-ethoxymethylfurfural).^397^ Protection of the aldehyde group in the HMF propargyl ether with Ac_2_O catalyzed by La(NO_3_)_3_, or with 2-methyl-furan (2-MF) catalyzed by AuCl_3_ delivered the products in >99% and 68% yield respectively. Gold-catalyzed IMDA/dehydration of 5-bis(acetyloxy)-HMF propargyl ethers produced mixtures of 5-hydroxy- and 5-acetoxylphthalane in 0–32% and 19–46% yields, respectively, depending on the catalysts. IPrAuNTf_2_ as catalyst converted 5-bis(5-methylfuran-2-yl)methyl-HMF propargyl ether to 5-hydroxyphthalane in 81% yield. EMF was converted to the EMF propargyl ether through hydrogenation and etherification. This product was subjected to the same gold catalysis to produce an isomeric mixture of phthalanes via IMDA/dehydration (Scheme 124).^397^

##### 2.5.3.8 Polymers

Diels–Alder reaction of furans is an attractive tool for polymer syntheses, as the reversibility of this reaction imparts a self-healing character to the polymers. The aromatization of the 7-oxabicyclo[2.2.1]hept-2-ene moiety prevents the retro-DA reactions, hence, improves the thermal stability of the polymers but also eliminates the self-healing ability.

**Scheme 125:**
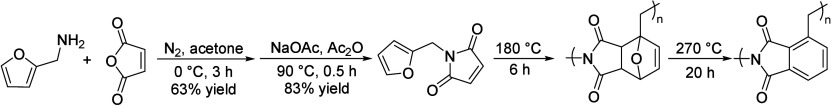
Polymers from DA reaction of *N*-(2-furylmethyl)maleimide^398^

Reaction of bio-based furfurylamine with maleic anhydride (MA) in acetone at 0 °C followed by dehydration in acetic anhydride produced *N*-(2-furylmethyl)maleimide as monomer. DA reactions of *N*-(2-furylmethyl)maleimide at 180 °C led to the 7-oxabicyclo[2.2.1]hept-2-ene-containing polymer, possessing very poor thermal stability (Scheme 125).^398^ The aromatized polymer was obtained at 270 °C from *N*-(2-furylmethyl)maleimide and had a lower molecular weight due to the dehydration. This aromatized polymer exhibits high thermal stability.^398^

**Scheme 126:**
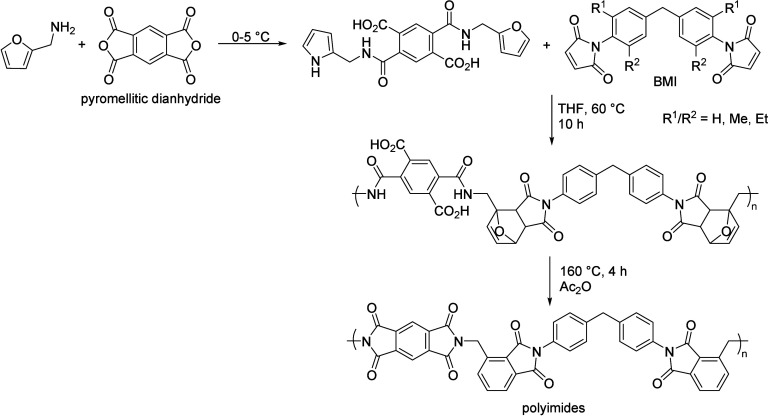
Polyimides from bio-based furfurylamine^399^

Furfurylamine was reacted with pyromellitic dianhydride at 0–5 °C. The resulting product was subjected to a DA reaction with BMI (1,1′-(methylenedi-4,1-phenylene)-bismaleimide) or derivatives thereof which resulted in the formation of 7-oxabicyclo[2.2.1]hept-2-ene-containing polymers. Dehydration of these polymers in acetic anhydride at 160 °C generated the polyimides (PIs) with better thermal stability (Scheme 126).^399^

**Scheme 127:**
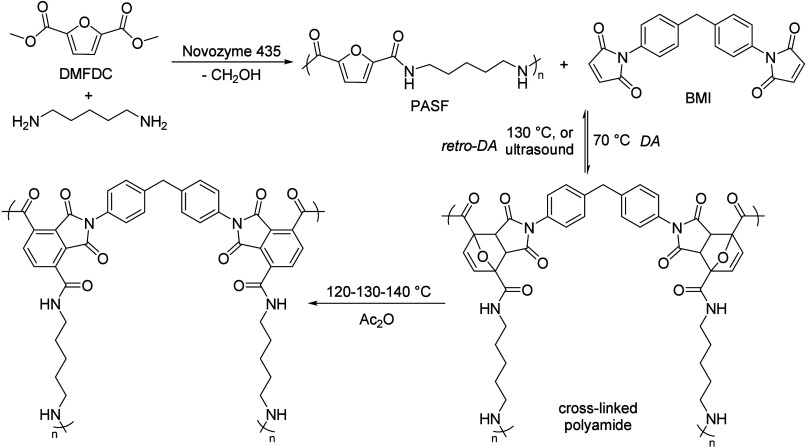
Preparation of cross-linked polyamide from bio-based DMFDC and its aromatization^400^

In 2020, Guo prepared bio-based furan-containing polyamides (PASF) by enzyme-catalyzed condensation of DMFDC and 1,5-pentanediamine. The DA reaction of PASF with BMI at 70 °C produced a cross-linked polyamide, which could be decomposed to PASF and BMI by ultrasound or by heating at 130 °C (Scheme 127).^400^ This cross-linked polymer was solidified by aromatization through a three-step increased temperature process. The aromatized polymer exhibited better thermal stability.^400^

#### 2.5.4 Aromatics Synthesized by Aldol-Condensation Reaction

The DA/dehydration reaction is currently the most promising approach to synthesize bio-based aromatics from furans. However, only a limited number of aromatic compounds have been produced through this approach.

**Scheme 128:**
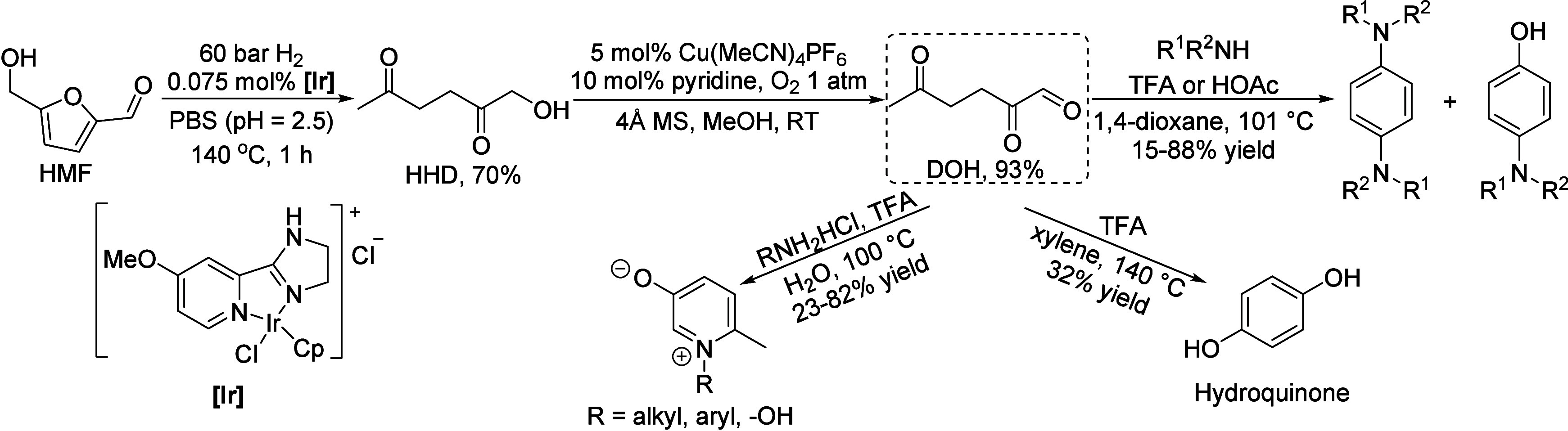
Synthesis of Benzenoid Aromatics and Pyridinium Salts via HHD and DOH as Intermediates

To extend the scope of bio-based aromatics, de Vries and co-workers reported a new strategy to prepare bio-based aromatics and pyridines from HMF (Scheme 128). Catalyzed by 0.075 mol% homogeneous Ir catalyst, HMF was converted to 1-hydroxyhexane-2,5-dione (HHD) under 60 bar H_2_ in 70% isolated yield.^401^ HHD has been identified as an important HMF-derived multifunctional building block for the synthesis of bio-based molecules.^402−404^ Fu has reported its synthesis from HMF in 98% yield catalyzed by an Ir catalyst.^405^ Cu-catalyzed of oxidation of HHD in the presence of 1 atm of O_2_ selectively converted HHD to 2,5-dioxohexanal (DOH) in 93% yield.^406^ DOH contains three carbonyl groups and was converted to aromatics through intramolecular aldol-condensation. Reaction of DOH and pyrrolidines in the presence of 1 equiv of TFA produced a range of 4-pyrrolidino-substituted phenols.^407^ When TFA was replaced by 2 equiv of HOAc, reactions of DOH and substituted pyrrolidines produced a number of 1,4-dipyrrolidinylbenzenes, interesting compounds that may serve as Wurster’s blue analogues. The total aromatics yields from DOH was 15–88%. In the absence of amines, DOH was converted to hydroquinone by acid catalysis in 32% yield at 140 °C using xylene as solvent. When H_2_O was used as solvent, the yield of hydroquinone showed a positive correlation with the temperature, and up to 32% yield was achieved at 265 °C under 56 bar N_2_.

Reaction of DOH and primary amine hydrochlorides in the presence of 2 equiv of TFA resulted in the formation of a range of *N*-substituted 3-hydroxypyridinium salts in 23–82% yields.^408^

This strategy could possibly be further improved by (a) the direct production of HHD from sugars, which has been studied by Jérôme who achieved a 55% yield from fructose, catalyzed by Pd/C and Amberlyst-15;^409,410^ (b) integrated conversion of HHD to aromatics, either one step or one-pot reaction; or (c) the use of strong heterogenous acids instead of TFA or HOAc.

**Scheme 129:**
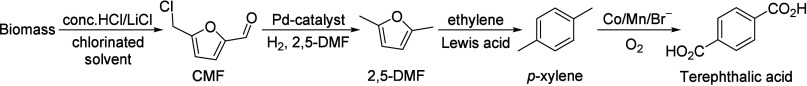
Origin Materials’ process for the production of terephthalic acid from biomass

In conclusion, although many aromatic compounds have been produced from furans on laboratory scale, most methods reviewed here suffer from shortcomings that make their large-scale production unlikely. Catalytic pyrolysis of furans delivers a mixture of aromatics in relatively low selectivity, even with co-feeds. Compared to biomass, the use of furans is rather expensive. Indeed, they are often used as probes for studying the mechanism of biomass pyrolysis. The research on intramolecular aldol-condensation of HMF-derived DOH is only at an early stage. The Diels–Alder/dehydration reaction of furans is probably the most promising method. Aromatics can usually be produced in high yield and high selectivity. The commonly obtained aromatics, such *p*-xylene, phthalic acids, and others, find broad application. The DA/dehydration of 2,5-DMF and ethylene is performed in a fixed-bed reactor and produces *p*-xylene in high selectivity. Origin Materials has commercialized this approach. They oxidize the *p*-xylene to terephthalic acid for the production of polyethylene terephthalate (PET). The 2,5-DMF is produced by hydrogenolysis of CMF that is obtained from biomass using concentrated HCl and a chlorinated solvent (Scheme 129).^130,265,411^

## 3 Aromatics from Lignin

**Figure 15:**
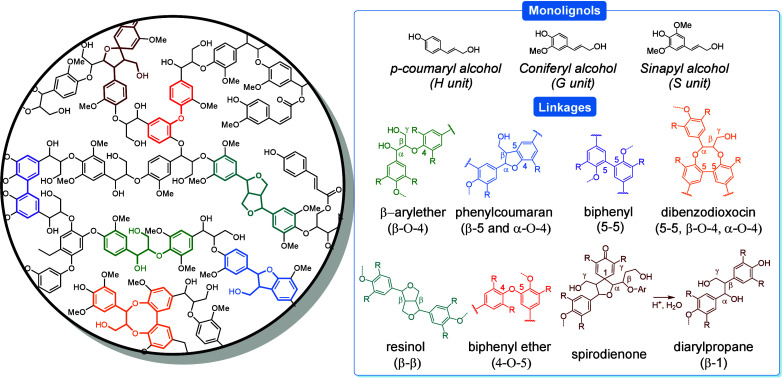
Monolignols, lignin structure and representative linkages in lignin

As a major plant cell-wall component lignin is a highly abundant biopolymer that is part of lignocellulosic biomass and foreseen to play an important role in a future influx of biogenic carbon in the chemical industry.^412,413^ Its major role in attaining aromatic chemicals from biomass comes from the fact that this biopolymer consists of aromatic subunits and is highly abundant.^414^ The native lignin structure is made up of propylphenols with different substitution patterns, of which the three main ones are sinapyl alcohol, coniferyl alcohol, and *p*-coumaryl alcohol that are radically cross-linked during the biosynthesis leading to S, G and H units, respectively, in the lignin structure (Figure 15). The radical cross-linking gives lignin an irregular structure with different C–O and C–C bonds, among which the most abundant is β-O-4 aryl ether. The composition in terms of linkages, the ratio of subunits and molecular weight as well as further decoration of the lignin structure is highly variable between different plant species and is further dependent on the part of the plant, various environmental factors and age (Table 24).^415,416^

**Table 24:** Abundance of monolignols and linkages in softwood, hardwood and grass^417^^a^

		Percentage of total amount/linkage [%]		
Component	Type	Softwood	Hardwood	Grass
Monolignol	H (*p*-coumaryl alcohol)	<5	0–8	5–33
	G (coniferyl alcohol)	>95	25–50	33–80
	S (sinapyl alcohol)	0	46–75	20–54

Linkages	β-O-4 (phenylpropane β-aryl ether)	43–50	50–65	74–84
	α-O-4 (phenylpropane α-aryl ether)	5–7	<1	n.d.
	4-O-5 (diaryl ether)	4	6–7	n.d.
	β-5 (phenylcoumaran)	9–12	3–11	5–11
	5-5 (biphenyl and dibenzodioxocin)	5–7	<1	n.d.
	β-1 (1,2-diaryl propane)	1–9	1–7	n.d.
	β-β (resinol)	2–6	3–12	1–7

a Reproduced with permission from ref (417). Copyright 2020 Wiley.

**Scheme 130:**
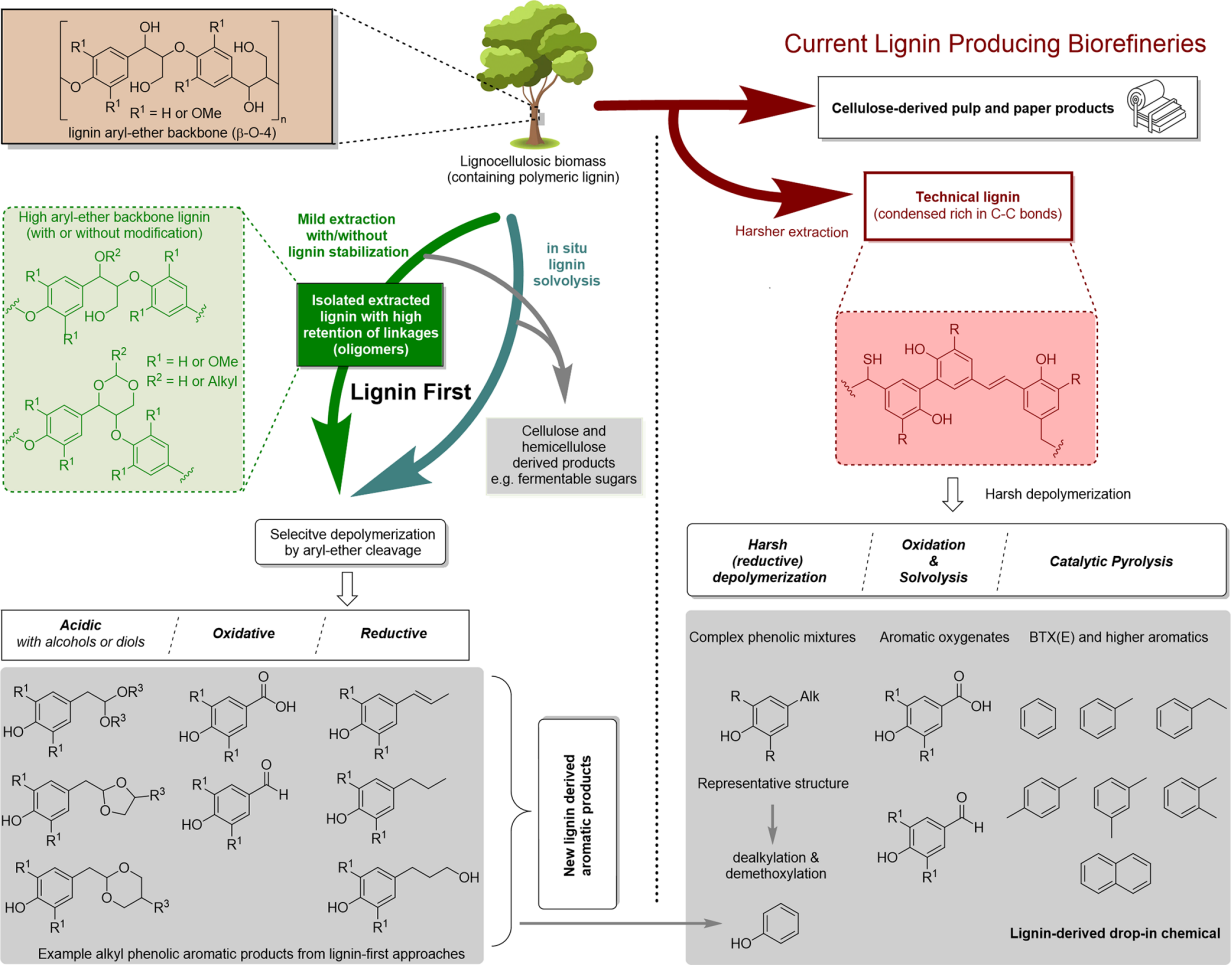
Main routes to aromatic chemical products from lignin indicating the contrast between classical routes via technical lignin and ”lignin-first” approaches

The chemical lignin structure becomes even more complex following the different pretreatments and fraction steps that are usually involved in lignocellulose processing.^418^ For instance, Kraft lignin, obtained through the Kraft pulping method employed in cellulose paper production, contains sulfide groups integrated into its structure. These groups originate from the sodium sulfide utilized in the pulping process. Moreover, Kraft lignin generally suffers from a higher degree of condensation, attributed to the breakdown of ether bonds. This breakdown results in the formation of fragments that subsequently recombine into more recalcitrant C–C bonds. Condensation reactions are similarly prevalent in various commercial fractionation processes. The diverse lignin types obtained through these commonplace procedures are usually denoted as technical lignins. Due to these changes to the chemical structure upon processing, the conversion approaches to produce aromatic chemicals can be highly dependent on the processing steps by which the lignin is obtained. In general, two main conversion strategies can be distinguished (Scheme 130): (1) Condensed technical lignins that are currently readily commercially accessible are used as starting material. However, here typically relatively harsh chemicals and/or conditions are required to crack condensed C–C bonds. (2) More selective targeted ether bond depolymerization can be applied to lignin with a higher ether content (more native-like) is referred to as “lignin-first” biomass processing.^419,420^ This “lignin-first” name arises from the vision that higher value from lignin can be attained via selective depolymerization which contrasts with processes where the main value of biomass is extracted from the carbohydrate fractions as is currently the dominant approach. Traditional pulping processes have the advantage of being well established but lignin depolymerization typically leads to more complex mixtures of aromatics which complicates downstream processing. Selective depolymerization accessible via the lignin-first approach has the potential to lead to high yields of specific (phenolic) aromatic monomers that can serve as new lignin-derived platform chemicals.^138^ These can either be funneled to current important aromatic intermediates as a drop-in approach or can serve as starting material for new emerging aromatic chemical products.^421^ A combination of more traditional lignin conversion methods and emerging lignin-first approaches is likely to coexist in the future. This balance aims to optimize profits derived from distinct value chains associated with various biomass components.

Here, an overview is provided of the conversion of lignin to phenolic monomers by both traditional and lignin-first type strategies complemented by several other strategies that can be categorized outside of these. This is followed by a collection of reactions toward diverse aromatics starting from the primary lignin depolymerization products. The phenolic nature of lignin means that phenolics are the main products encountered. Therefore, strategies to yield non-phenolic aromatic compounds directly from lignin are highlighted separately. Due to the sheer amount of relevant work in the context of the production of aromatic chemicals and especially those reporting phenolic mixtures from lignin, a complete reference overview is not feasible. We provide an overview of the main approaches and place the focus on providing an overview that includes the most important representative examples within different approaches to show the potential of each. Furthermore, we aim to give a more in-depth focus on studies where tangible good (isolated) yields of specific aromatic monomers or high yields of mixtures are attained. In the separate sections citations to the most important reviews on the different subtopics are provided for further reading.

### 3.1 Conversion of Technical Lignins to Phenolic Monomers

There are vast amounts of lignin produced as a residue in the pulp in paper industry. These are mainly available in the form of technical lignins to a total amount of around 100 Mt/y. The most prominent in terms of potential availability is Kraft lignin which is the residue recovered from black liquor from Kraft pulping, which uses sodium sulfide under alkali conditions at elevated temperatures and is the major pulping process.^422^ However, most of the black liquor is incinerated for energy and for the recovery of inorganic chemicals. This means that the main marketed lignin is lignosulphonate obtained from the sulfite process due to its applications in the construction sector. Both of these marketed technical lignins have significantly altered structures compared to native plant lignin and contain significant amounts of sulfur. Other technical lignins that are also available on smaller scale are alkali lignin and organosolv type lignins. These are low in sulfur and can also have less degraded structure if operated at mild conditions. However, it has been shown that typical available organosolv lignins are also low in β-O-4 type ether linkages and are severely condensed.^423^ Another technical lignin-derived product is pyrolytic lignin.^424^ This oily lignin-derived product constitutes the water insoluble fraction from biomass pyrolysis oil rich in small condensed phenolic structures.

**Figure 16:**
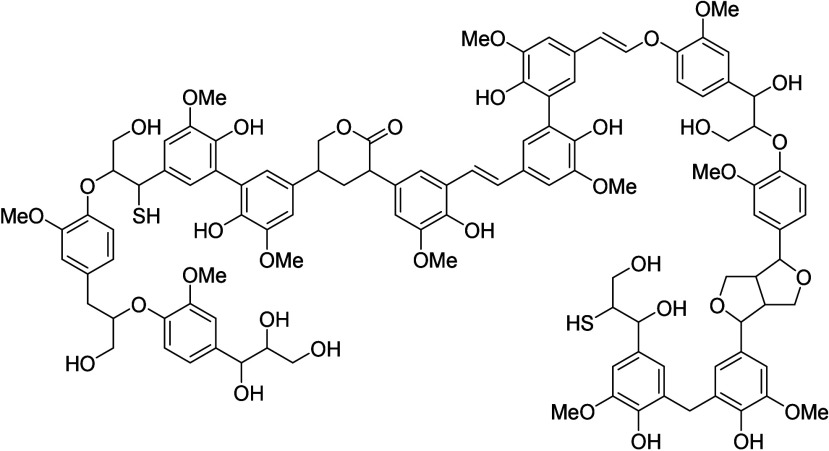
Model structure of a representative softwood Kraft lignin based on structural motifs suggested in the literature^426^

Due to the processing conditions the structures of technical lignins are extraordinarily complex and contain many carbon–carbon bonding motifs (see example structure in Figure 16).^425,426^ The complex condensed nature of technical lignins make them difficult to selectively depolymerize as bond cleavage conditions for the different motifs are highly variable. This is aggravated by the fact that at the required severity, formed fragments are often highly reactive. As the product mixtures are highly complex, depolymerization to chemicals is still challenging.^414^ Nevertheless, depolymerization can lead to relatively good yields of monomeric compounds including phenolic aromatics that can be of interest as chemical building blocks. The main strategies for depolymerization to phenolic products used are thermal, solvolytic, oxidative and reductive methods which are reviewed separately in the following sections. While the yields and selectivity for specific aromatic products are typically lower than those of emerging strategies such as those discussed in the other sections, these conversion strategies could have an important role to play in the transition to bio-based aromatics from lignin due to the sheer amounts of technical lignin that are commercially available and the push from the pulping industry to extract more value from these residues.

#### 3.1.1 Acid/Base-Mediated Solvolysis and Thermal Conversions

Important classical methodologies for the conversion of lignin are treatments leading to a liquid product. Solvolysis and pyrolysis are able to provide high amounts of organic oil. Most research in this field was aimed at obtaining oils for biofuel production. Nevertheless, it has been shown that these oils contain reasonable amounts of various aromatic monomers. In both approaches, the solid lignin is converted to a complex oil which contains a variety of monomeric and oligomeric aromatic compounds, and oxygen content is reduced by expelling water and gases such as CO_2_. In solvolysis this is achieved by degradation in a solvent often aided by reactivity of the medium (acidic or basic). In pyrolysis, direct thermal conversion in the absence of oxygen is used and the oil is obtained by collection of a liquid by condensation of gaseous fragments. Both methods rely on fragmentation leading to reactive intermediates that can readily lead to secondary reactions and undesirable solid formation.^427^ This can be overcome by stabilization or conversion of reactive fragments in the medium or by achieving short residence times. An example of stabilization is combining solvolysis with oxidative and reductive treatments such as hydrogenolysis as discussed further in sections 3.1.3 and 3.2.3. Furthermore, integrated upgrading of the oil components (or of the vapors) is often applied by the addition of catalysts yielding deoxygenated aromatics, which will be further discussed in section 3.5.1. However, due to the focus on fuel production, the analysis of monomer composition in the oil is often incomplete and/or not well-quantified for inclusion in this review. We have limited the used examples to those with significant quantified phenolic aromatic monomer yields.

##### 3.1.1.1 Solvolysis

In solvolysis (also known as Lignin-to-Liquid, LtL), the objective is to achieve fragmentation and oxygen removal to enhance the yield of organic components. Therefore, reactive media and hydrogen donor environments are employed. Typical temperatures lie between 100 and 400 °C (Table 25).^428,429^ Alkali solutions are often used due to the enhanced solubility of most technical lignins in alkali media (Table 25, entries 1–3).^430−434^ This is often referred to as base-catalyzed depolymerization (BCD). The typical monomeric products are, depending on the origin of the lignin, mixtures of phenol, guaiacol, catechol and syringol as well as alkylated versions of these. Reasonable monomer yields were obtained from reactions at pilot scale. It was also shown that when processing conditions are harsher, more demethylation occurs and consequently more catechols are formed (Table 25, entries 4 and 5).^435^ Heterogeneous base catalysts like NaX zeolite have been used as catalyst, with vanillin and vanillone identified as major components in the resultant product mixtures. (Table 25, entry 6).^436,437^ Solvolysis can also be performed under acidic conditions using mineral acids (Table 25, entry 7)^438^ or formic acid, which can play an additional role as hydrogen donor (Table 25, entries 8–11).^439−443^ These have also been combined with transition metal catalysts such as Pd to further enhance depolymerization (Table 25, entry 12).^444,445^ In addition, solid acid catalysts (Brønsted and Lewis acid) have been applied to facilitate solvolysis (Table 25, entries 13 and 14).^445−448^

**Table 25:** Overview of Solvolytic Methodologies for the Depolymerization of Lignin with Yields of Phenolic Monomers

Entry	Feedstock	Medium	Conditions	Oil yield	Composition	Notes	Ref
1	Softwood Kraft lignin (Indulin AT)	10 wt% solution in 5 wt% NaOH (aq)	270–315 °C, continuous-flow reactor with LHSV 1.4–4.0 h^–1^	Up to 19.1 wt% of a monomer-rich fraction obtained after acidification and extraction	Up to 8.4 wt% identified monomers	Main products: guaiacol and pyrocateechol; also other lignins were studied	(430,431)
2	Olive tree prunings ethanosolv lignin	5 wt% solution in 4 wt% NaOH (aq)	300 °C, 90 MPa, batch reactor, 40 min	20 wt% oil yield after acidification, extraction, and evaporation	Oil contained 10 wt% catechol and 5 wt% methyl catechol	Also neutral water, KOH, LiOH, K_2_CO_3_, and Ca(OH)_2_ were tested, as were other organosolv lignins	(432,433)
3	Kraft lignin	100 mg lignin, 25 mL aqueous 10 mM NaOH	200 °C, batch reactor, 8 h	Not provided	13 wt% monomers	>80% selectivity to guaiacol	(434)
4	Beech wood organosolv lignin	2.5 wt% NaOH (aq)	250–340 °C, 5–20 kg/h plug-flow reactor, 450–900 s residence time	Oil yield up to 80–85 wt% at 250 °C to <60 wt% above 300 °C	Guaiacol, catechol, and syringol up to about 40 wt% in the oil	Catechol component increases at higher *T*	(435)
5	Spruce wood Kraft lignin	2.5–7.5 wt% lignin and NaOH (aq)	250–300 °C, 5–20 kg/h plug-flow reactor, 450–750 s residence time	Oil yield up to 80–85 wt% at 250 °C to around 60 wt% at 300 °C	Guaiacol and catechol up to about 30 wt% in the oil	Catechol component increases at higher *T*	(435)
6	Alkali lignin	Lignin:NaX (solid base cat):EtOH:H_2_O 1:1:40:60	250 °C, batch reactor, 1000 rpm stirring	51 wt% oil yield after acidification, extraction, and solvent evaporation	About 16 wt% of the main identified monomers derived from lignin	Methyl guaiacol, vanillin, and vannilone as main products; also other supported metal catalysts and lignins were tested	(436,437)
7	Wheat straw alkali lignin	1 g lignin, 20 g ethylene glycol, and 10 wt% H_2_SO_4_	120 °C, batch microwave reactor, 40 min	97% liquified	Up to 14 wt% identified monophenolics, mainly methyl guaiacol	Showing benefit of microwave irradiation and effect of reaction conditions	(438)
8	Wheat straw soda lignin (Protobind 1000)	Different ratios of lignin, HCOOH, and EtOH	360–400 °C, batch reactor and CSTR, 15–1180 min	Approx. 60–70% liquid yield	Up to around 40 mg/g lignin combined yield of phenol, guaiacol, catechol, and alkylated phenolics	Kinetic model for the formation of main components was demonstrated	(439,440)
9	Norway spruce ethanosolv lignin	Different ratios of lignin, formic acid, and water	320–360 °C batch reactor, 2 h	78–94 wt% based on lignin input	Up to 9.5 wt% identified monomeric products	Main components are guaiacol and methyl/ethyl/propyl guaiacol	(441)
10	Pine wood lignin extracted from black liquor from acid hydrolysis	3 g lignin in 90 mL 1:1:0.4 H_2_O:EtOH:HCOOH	330 °C, batch reactor, 2 h	64.2 wt%	23.0 wt% monomers based on lignin intake	Mainly 4-*n*-proply guaiacol (5.5 wt%), methylguaiacol (3.4 wt%), catechol (3.0 wt%), ethylguaiacol (2.7 wt%), and phenol (2.1 wt%)	(442)
11	Softwood Kraft lignin (Indulin AT)	0.5 g lignin, 10 mL EtOH, 10 mL 1,4-dioxane, and 2 mL HCOOH	300 °C, batch reactor, 2 h	About 50 wt%	22.4 wt% phenolic monomers	Mainly 4-*n*-propyl guaiacol	(443)
12	Norway spruce weak acid–enzymatic hydrolysis lignin	2 g lignin, 3 g HCOOH, 5 g H_2_O, and 0.2 g cat (Rh, Ru, or Pd supported on alumina)	340–380 °C, batch reactor, 2–6 h	80–91 wt% oil yield (highest for Ru on alumina)	Up to 18.5 wt% identified monomers in the oil	Main product is cresol	(444)
13	Corn stover acetosolv lignin	1 g lignin, 100 mL acetic acid, and 0.1 g Zr-KIT-5 (Lewis acid cat)	250 °C, batch reactor, 1000 rpm stirring, 6 h	43 wt% oil yield based on lignin intake	28 wt% cumulative yield of identified phenolic monomers	Catalysts could be reused in multiple runs; GVL and MeOH/H_2_O were tested as medium with other lignins	(445−447)
14	Bagasse organosolv lignin	1.75 w/v lignin in MIBK and 1 wt% heterogeneous carbonaceaus solid acid catalyst	350 °C, batch reactor, 3 h	Over 90% lignin conversion	32% identified phenolic monomers, mainly ethyl guaiacol	Also other acidic catalysts and solvents were evaluated	(448)

##### 3.1.1.2 Pyrolysis

For pyrolysis, the rule of thumb is that the higher the heating rate, the higher the oil yields. Higher yields of aromatic (phenolic) monomers can be obtained due to the suppression of extensive charring that will happen when heated slowly. Fast pyrolysis is considered to have heating rates of over 100 °C/s.^428^ The typical optimal pyrolysis temperature is somewhere around 450 °C. Without upgrading catalysts, the oil typically consists of a variety of substituted phenols, and guaiacols. Many different reactor types and designs have been applied at various scales ranging from mg to multiple kg scale.^449^

In addition to the scale of the operation, parameters such as the experimental setup, feeding method, types of the lignin (chemical composition, level of purity, and pretreatment method), addition of additives, and additional factors like the strategy employed for product recovery can significantly influence both the yields and product distribution.^450^ For example, the processability of the lignin can be significantly affected by the amount of carbohydrate impurities.^451^ Herein, we present a chosen set of representative investigations that employed pyrolysis without gas-upgrading on a multi-gram scale. These studies quantified the yield of phenolic monomers using absolute analytical methods, providing insights into the yield and composition of products obtained from diverse lignin sources under varying conditions and technologies (Table 26). The main products derived from lignin are typically alkylphenolics (H, G, and S type) including vinyl substitutions with a combined maximum yield of up to around 10 wt% based on lignin input.

**Table 26:** Yields of Oil and Phenolic Monomers from Fast Pyrolysis of Different Lignins under Different Conditions without Oil Upgrading

Entry	Feedstock and scale	Reactor type	Conditions	Organic oil yield	Phenolic yield and composition	Notes	Ref
1	50 g soda lignin (mixed non-wood origin)	Bubbling fluidized silica sand bed	500 °C, 1–2 s, 10 g batches every 1–2 min	11 wt% based on dry feedstock	Up to 8% phenolics (5% monomeric), main: guaiacol, 4-methyl guaiacol, 4-ethyl guaiacol, and syringol	Comparative analysis of pyrolysis of same lignin samples in different laboratories	(452)
2	450 g Kraft lignin^423x^–clay (30 wt%) extrudates	Bubbling fluidized silica sand bed reactor	550 °C, atmospheric pressure	Up 31 wt% oil yield of up to 48% liquid	Up to 17 wt% phenolic in the rt electrostatic condensed fraction	Other lignins were also tested at lower temperature and smaller scale	(453)
3	300 g acetosolv lignin pretreated with Ca(OH)_2_	Bubbling fluidized bed reactor	450–600 °C, 100 g/h	Approx. 38 wt% total liquid with 34% water content	Oil contains 55–60 wt% phenolics of which 16% identified monomers (6 wt% based on lignin intake)	Vinylphenol and vinylguaiacol as main products; also phenol, guaiacol, syringol, and other alkylphenolics	(454)
4	200 g Alcell organosolv lignin	Bubbling fluidized bed reactor, continuous mode	400 °C, 100 g/h, 1 s vapor residence time	21 wt% of 40% total liquid	13 wt% phenolics in the oil	Mostly alkyl phenols, alkyl guaiacols, and alkyl syringols	(455)
5	200 g soda lignin (non-woody origin)	Bubbling fluidized bed reactor, continuous mode	400 °C, 100 g/h, 1 s vapor residence time	13 wt% of 40% total liquid	20 wt% in the oil	In addition to the above, also a significant amount of catechols was observed	(455)
6	40 g soda lignin (non-woody origin)	Bubbling fluidized bed reactor, batch mode	500 °C, 100 g/h, 1–3 s vapor residence time	47.6 wt% liquid of which 17.1% was water	11.2 wt% monomers based on lignin input	Major monomers: vinyl guaiacol, phenol, guaiacol, syringol, and alkylphenol	(456)
7	Beech wood ethanosolv	Bubbling fluidized bed reactor	497 °C, 4 g/10 min	Up to 18 wt% phenolic oil	11.6 wt% monomeric phenolics based on lignin input	4-Methylsyringol as major identified component in 3.45 wt% based on lignin input	(457)
8	300 g softwood Kraft lignin	Batch microwave reactor	627–967 °C, 3.2 kW, 800 s	Up to 18% oil and up to 53% total liquid	Up to 35.3 wt% identified monomers in the oil phase	Main components are alkylated phenols, guaiacols, and catechols	(458)

##### 3.1.1.3 Product Isolation

For both pyrolysis and the different solvolysis methods, the isolation of the valuable phenolic monomers from the complex product mixtures can be challenging and costly.^428,449^ In the case of pyrolysis there is the option to utilize a staged condensation to collect different fractions with selected composition like an aqueous and organic fraction^453^ or a heavy and a light oil, which allow for separate upgrading strategies.^459^ Beyond this, different options like liquid–liquid extraction (LLE), column chromatography (CC), membrane separation, and distillation have been investigated.^428^ The acidic nature of phenols makes base extraction effective for enrichment. Switchable hydrophilicity solvents in which the reversible reaction between an amine such as *N*,*N*-dimethyl-cyclohexylamine and CO_2_ allows the solvent to switch between lipophilic and hydrophilic have been used to separate guaiacol and methyl guaiacol from lignin pyrolysis oil.^460^ For base-catalyzed depolymerization mixtures, a combination of LLE, distillation and crystallization were utilized to isolate guaiacol, catechol, 4-methyl-catechol, 4-methoxy-catechol, and syringol separately.^428^

#### 3.1.2 Oxidative Conversions

**Scheme 131:**
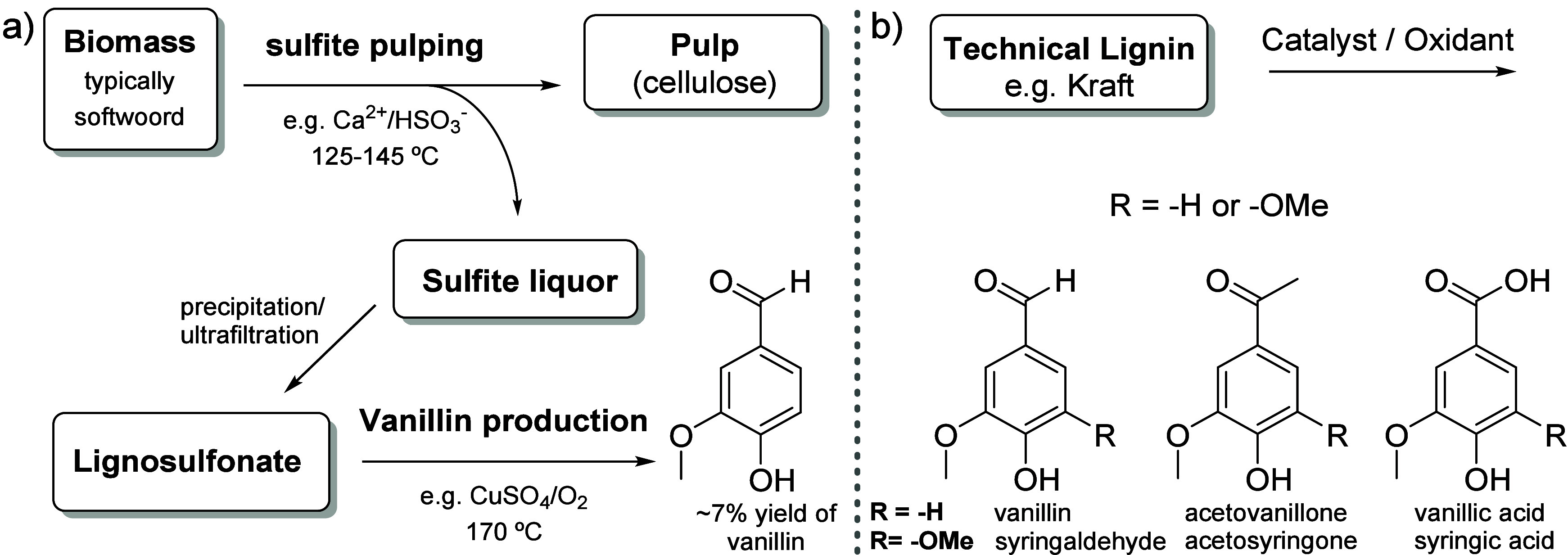
(a) Schematic Representation of the Commercial Vanillin Production Process via Sulfite Pulping; (b) Typical Products from Technical Lignin Oxidation

The most important commercially produced aromatic compound from lignin is vanillin, which can economically compete with petrochemical routes and is more suitable for bulk production compared to lignin hydrolytic extraction from other natural sources.^461^ The production of vanillin from lignin is currently done via oxidation of lignosulfonates attained from softwood pulping and makes up about 15% of the vanillin market. This process utilizes aerobic oxidation (10–12 atm air) with copper catalysts such as copper(II) sulfate at elevated temperatures in the range of 130–200 °C and has typical yields of around 7 wt% (Scheme 131a).^462,463^ Another classical method for oxidation of lignin to vanillin and derivatives is nitrobenzene oxidation (NBO),^464^ which is usually used as a benchmark to compare new (catalytic) methodologies. For the oxidation of lignins toward vanillin, many different oxidants and catalysts can be used.^465^ Other aromatic products besides vanillin can be produced such as acetovanillone (apocynin) and vanillic acid. In the case of lignin derived from biomass containing lignin structures with significant S content, the related products are syringaldehyde, acetosyringone, and syringic acid (Scheme 131b). In addition to these, numerous minor products may form, including hydroxybenzoic acids and aliphatic compounds resulting from side chain oxidation. Furthermore, secondary oxidative ring opening of the primary phenolic products can occur. When chlorine-based oxidants are used, chlorinated aromatics are obtained but mostly appear in complex mixtures in low yields.^466^ An alternative reported product are quinones, albeit again at relative low yields.^467,468^

The catalytic oxidation of technical lignins beyond lignosulfonates (in particular Kraft lignin) has received much attention and there are numerous studies evaluating specific elements such as oxidants and catalyst combinations, which have been reviewed extensively in recent years.^461,469,470^ An excerpt of manuscripts where relatively high yields were obtained is provided in Table 27. Non-catalytic methods under acidic or alkali conditions or those using iron Fenton systems only reach low yields of aromatics due to low conversions and/or low selectivities due to overoxidation. Catalytic methodologies can steer the reaction selectivity toward monomeric phenolic aldehydes or acids. Good aldehyde yields have been achieved in aerobic oxidation in alkali media using lanthanum oxide-based perovskite-type catalysts,^471,472^ in particular those containing iron and copper^473^ (Table 27, entry 1), supported molybdenum pyrophosphate catalysts (Table 27, entry 2), copper cobalt mixed oxide catalysts prepared by alginate gelation (Table 27, entry 3).^474^ Copper-manganese-based oxides with a spinel phase proven to be effective for the oxidation of Kraft lignin with H_2_O_2_ in alkaline medium at elevated temperature to give a mixture of monomers in around 17% yield (Table 27, entry 4).^475^ Patankar et al. showed that up to 28.5 wt% yield of monomers of which 20 wt% vanillin can be obtained from oxidation of a fractionated Kraft lignin with NaOCl by using a magnetically recyclable TEMPO catalyst (Table 27, entry 5).^476^ Activity and selectivity were well retained over 2 reuses after simple recovery with a magnet without any regeneration.

**Table 27:** Processes for the Catalytic Oxidation of Technical Lignins

Entry	Lignin	Oxidant	Catalyst	Conditions	Major product(s)	Yields	Ref
1	Enzymatic hydrolysis of steam explosion corn stalk	O_2_	LaFe_0.8_Cu_0.2_O_3_/ LaMnO_3_	Aqueous NaOH, 120 °C, 30–120 min	*p*-hydroxybenzaldehyde/vanillin, syringaldehyde	Up to 2.5%/5.6 /11.5, respectively	(471−473)
2	Alkali lignin	O_2_	MoPO/ CeO_2_	Aqueous NaOH, 150 °C, 3 h	Vanillin	9%	(495)
3	Wheat straw organosolv lignin	10% O_2_ in N_2_	CoFe (1:1) mixed oxides	Water, 200 °C, 4 h	Vanillin/syringaldehyde (17%/50% selectivity as part of the total monomers)	19.6% identified aromatic monomers	(474)
4	Kraft lignin	H_2_O_2_	CuMn mixed oxides (1:3)	Aqueous NaOH, 180 °C, 60 min, 26 h	*p*-hydroxybenzaldehyde /vanillin/vanillic acid /acetovanillone	About 17%	(475)
5	Softwood Kraft (Indulin AT)	NaOCl	TEMPO immobilized on iron magnetic beads	Aqueous NaBr, 25 °C, 4 h	Vanillin (78% selectivity)	Up to 28.5%	(476)
6	Milled softwood Kraft (Indulin AT)	a) *t*-BuOOH	a) TPPFeCl	a) Phosphate buffer (pH = 3): MeCN 3:1, rt	Methyl vanillate, 5-carbomethoxy-vanillate	10%	(496)
		b) H_2_O_2_	b) –	b) HCOOH/H_2_O, 50 °C, 70 h			
7	Acid milled alkali lignin	Visible light	TiO_2_	Water, 250 W/m^2^ light	Vanillin, homovanillic acid, acetovanillone, guaiacol, and vanillic acid	22.8 wt%	(480)
8	Kraft lignin (Indulin AT)	O_2_	H_3_PMo_12_O_40_·*x*H_2_O	1:4 H_2_O:MeOH, 170 °C, 20 min	Vanillin/methyl vanillate (70% of total mon.)	10% monomers	(481)
9	Organosolv lignin	O_2_	Au/CeO_2_	MeOH	Vanillin/methyl vanillate/methyl syringate	About 21 wt%	(482)
10	Softwood Kraft (Indulin AT)	O_2_	Au/Li-AlLDH	a) DMF, 120 °C, 1 h	Vanillin/vanillic acid	Around 10%	(483)
				b) 0.1 M NaOH (aq.)			
11	Sodium lignosulfonate	Air	–	Bu_4_NOH·30H_2_O, 120 °C, 43–70 h	Vanillin/vanillic acid	6.5%	(484)
12	Soda lignin	Air	–	Bu_4_NOH·30H_2_O, 120 °C, 43–70 h	Vanillin/vanillic acid	6.7%	(484)
13	Alkali lignin	[Pro]_2_[MnCl_4_]	–	Ethyl ammonium nitrate, 35 °C, 4 h	Vanillin/acetovannillone	18–20% vanillin	(485)
14	Alkali lignin	O_2_	[HC_4_im]_3_PMo_12_O_40_	([HC_4_im]HSO_4_) 80%, 150 °C, 5 h	Complex mixture of phenolic monomers	Up 15 wt%	(486)
15	Beech organosolv lignin	Air	Mn(NO_3_)_2_	[EMIM][CF_3_SO_3_], 100 °C, 24 h	Depending on the catalyst concentration, syringaldehyde or DMBQ as main product	11.5 wt% isolated yield of DMBQ	(487)
16	Softwood Kraft (Indulin AT)	Electric current	Ni electrodes	Aqueous NaOH, 160 °C	Vanillin and acetovanillone	Up to 7 wt%	(497)
17	Lignosulfonate	Electric current	Ferrate	Aqueous NaOH	Vanillic acid	7.2 wt%	(498)
18	Butanosolv lignin	Electric current	Lead/mercury electrodes	Aqueous NaOH	Phenolic carboxylic acids	23.5 wt%	(494)
19	Spruce lignosulfonate	Electric current	Nickel mesh	Aqueous NaOH	Vanillin	9.6 wt%	(493)
20	Bamboo lignin	Electric current	Cu and Pb/PbO_2_ electrode	Aqueous NaOH	Vanillin/syringaldehyde/*p*-coumaric acid	3.6/5.7/3.0 wt%, respectively	(493)

Due to the heterogeneity of the lignin structure, the exact oxidative depolymerization mechanism is hard to determine. Nevertheless, much work with model compounds has shed light onto the subject.^466,470,477,478^ Proposed mechanisms show that depolymerization occurs via hydrolysis of aryl ether bonds, facilitated or not by oxidized intermediates (depending on the presence of a phenolic end group that allows for the formation of a phenoxy radical), followed by a stepwise oxidative degradation of the side chain to the main products (discussed in more detail in section 3.2.2). The latter is known to be dominant when oxidants such as TEMPO are applied. Either way, the C_α_–C_β_ gets cleaved to form the aldehyde, which depending on the conditions can get further oxidized to the acid. The second pathway is via ketone intermediates as demonstrated in a two-step procedure by Yao et al. KOH ball-milled Kraft lignin was oxidized using *t*BuOOH as oxidant with a homogeneous iron-porphyrin catalyst to install ketone groups at the benzylic positions. This ketone-containing lignin was then subjected to Baeyer–Villiger oxidation using formic acid/H_2_O_2_ followed by hydrolysis of the formed esters to give 10 wt% monomers (Table 27, entry 6). After methylation of the products for analysis, methyl vanillate and methyl 5-methoxycarbonyl-vanillate were detected as the main components. In this study, it was also shown that the mechanochemical pretreatment with KOH was essential for effective depolymerization via this strategy as it increased chemical reactivity, likely by decreasing the particle size and an increase of the reactive surface. In addition, increased carbonyl content could be observed by IR analysis after milling, which has been seen also in other studies on the effect of milling on the chemical structure of lignin.^479^ The beneficial effect of mechanochemical treatment was also shown for the TiO_2_-catalyzed photocatalytic degradation of alkali lignin to guaiacol, vanillin, acetovanillone, vanillic acid and homovanillic acid (Table 27, entry 7).^480^ Unmilled samples gave no phenolic monomers while up to 22.8 wt% of monomers could be obtained after the photocatalytic degradation of ball-milled acid-impregnated lignin.

Oxidation in methanol as an organic medium has been reported to give decent yields of phenolic products. Aerobic oxidation yielded around 10 wt% monomers from Kraft lignin using a homogeneous polyoxometalate (H_3_PMo_12_O_40_) catalyst in a 1:4 H_2_O:MeOH mixture^481^ and about 21 wt% monomers from an organosolv lignin using a ceria supported gold catalyst in pure methanol (Table 27, entries 8 and 9).^482^ In addition to phenolic aldehydes, esters are formed, likely via oxidation of corresponding hemiacetal intermediates. Around 10% phenolic acid and aldehydes could be obtained using two-step process where Kraft lignin was first oxidized using gold nanoparticles supported on a lithium-aluminum (1:2) layer double hydroxide support in dimethylformamide (DMF) followed by hydrolysis using sodium hydroxide (Table 27, entry 10).^483^ In the first step, benzylic oxidation takes place, forming ketone structures as well as esters. These are hydrolytically cleaved in the second step to form the final products.

**Scheme 132:**
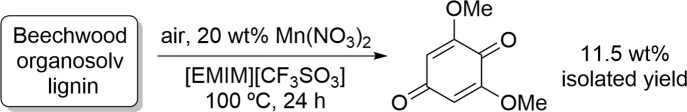
Catalytic Oxidation of Beechwood Organosolv Lignin to DMBQ^487^

Solubility of the lignin can be an issue in the conversion of technical lignins. This can be overcome by using ionic liquids (IL) which are excellent solvents for various types of lignin. Aerobic oxidation in Bu_4_NOH·30 H_2_O at relatively mild temperatures of 120 °C yielded reasonable amounts of monomers from sodium lignosulfonate and soda lignin (Table 27, entries 11 and 12).^484^ Oxidation using prolinium tetrachloromanganate in ethyl ammonium nitrate resulted in 18–20% vanillin yields from alkali lignin (Table 27, entry 13).^485^ IL-modified polyoxometalates were used for the aerobic oxidation of alkali lignin in a mixture of 80 wt% 1-butyl-3-methylimidazolium hydrogensulfate (([HC_4_im]HSO_4_)_80%_) and 20 wt% water to yield up to 15 wt% of a complex mixture of phenolics and aromatics.^486^ Bösmann and Wasserscheid et al. tested various ionic liquids for the aerobic oxidation of organosolv lignin with Mn(NO_3_)_2_. In [EMIM][CF_3_SO_3_] with 20 wt% catalyst, 6-dimethoxy-1,4-benzoquinone (DMBQ) was the main product which could be isolated from the solution in 11.5 wt% yield via extraction and crystallization (Table 27, entry 15, Scheme 132).^487^

The isolation of DMBQ, as performed here, stands out among other research as the isolation of single aromatic chemicals such as vanillin from complex mixtures obtained from the oxidation of technical lignin streams can be challenging.^469^ It was shown that vanillin can be separated from a mixture derived from alkali aerobic oxidation (content 2.16 g/L vanillin) containing also vanillone and vanillic acid by extraction and subsequent crystallization.^488^ Alternatively, ultra- and nano-filtration and extraction in combination with distillation and adsorption can be used.^489^

In addition, oxidative degradation of technical lignins can be initiated using electrochemistry (Table 27, entries 16–20). This has advantages in terms of controlling selectivity using current density, voltages, and electrode materials. Furthermore, such methods are considered to have the potential to be less energy intensive and can be combined with another process such as hydrogen generation.^490−492^ Reported aromatic products and yield vary depending on conditions and design of the setup. The studies are difficult to compare, but reasonable to good yields have been reported.^493^ An early report claimed the conversion of butanosolv lignin using lead and mercury electrodes to a mixture of phenolic carboxylic acids in up to 23.5 wt% yield, which were separated by distillation (Table 27, entry 18).^494^ Typical product mixtures contain vanillin and vanillic acid with the co-generation of aliphatic acids and CO_2_.

Regardless of the many different approaches to lignin oxidation, it has been clearly shown that probably the most important parameter is the lignin feedstock used to produce aromatic monomers such as vanillin. For example, the mild aerobic oxidation method performed at 120 °C in tetrabutylammonium hydroxide 30-hydrate (Bu_4_NOH·30 H_2_O) gave combined yields of 6.5 and 6.7 wt% of vanillic acid and vanillin from sodium lignosulfonate and soda lignin, respectively (Table 27, entries 11 and 12), while 16.3 and 22.5 wt% were obtained from milled wood lignin and wood meal, respectively.^484^ Similarly, GVL extracted lignin gave significantly higher monomer yield compared to Kraft lignin (40 vs 10 wt%) upon oxidation with gold catalysts.^484^ These examples show that the amount of cleavable bonds in the substrate likely have a large influence on the obtained yields, especially when mild oxidative conditions are used. These factors contribute significantly to the notable variations in yields observed across different studies. Insufficient details regarding the starting materials, particularly when utilizing commercial technical lignins or samples from demonstration processes as feedstock, often contribute to this variability. In section 3.2.2, the oxidation of more native-like lignin is discussed. In these studies, more emphasis is placed on high quality of the lignin as starting material in terms of cleavable bonds.

#### 3.1.3 Reductive Conversions

**Scheme 133:**
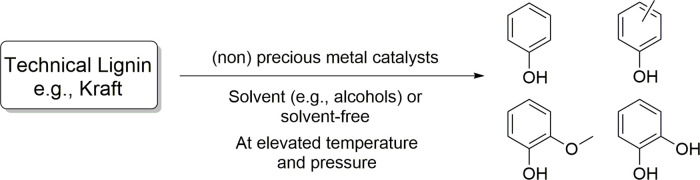
Catalytic Hydrotreatment of Technical Lignins to Phenols

Catalytic hydrotreatment is an effective way to depolymerize technical lignins.^412,417,499^ Typically, the process is conducted with molecular hydrogen or a hydrogen donor (e.g., methanol or formic acid) and a catalyst at elevated temperatures and pressures. Various reactions take place, such as hydrogenolysis, hydrogenation, hydrodeoxygenation, and demethoxylation, leading to depolymerization and hydrodeoxygenation of lignin (Scheme 133).

There is an extensive amount of literature about the hydrogenolysis of technical lignin which have been reviewed in recent years.^412,417,500^ To give an idea about the potential of different methodologies, in this part we focus on the publications on the catalytic hydrotreatment of technical lignins to phenols (e.g., phenol, cresols, guaiacol, and catechol) with high yield (>20% based on lignin intake). These studies (Table 28) can be classified into studies involving the use of an external solvent, and those without using an external solvent (solvent-free).

**Table 28:** State-of-the-Art Overview for the Catalytic Hydrotreatment of Technical Lignins to Phenols

Entry	Lignin	Solvent	Catalyst	Reaction conditions	Major products	Yield (wt%)	Ref
1	Alkali lignin	H_2_O + formic acid	Pd/C	265 °C, 0.1 Mpa N_2_, 6 h	Phenols (mainly catechol)	25.6	(503)
2	Alkali lignin	Methanol	Pd/C + CrCl_3_	280 °C, 4.0 Mpa H_2_, 5 h	Phenols	35.4	(505)
3	Enzymatic hydrolysis lignin	H_2_O + methanol	Raney Ni + H-USY	270 °C, 0.1 Mpa N_2_, 6 h	Phenols	27.9	(515)
4	Kraft lignin	Methanol	NiWS/C	320 °C, 3.5 Mpa H_2_, 24 h	Phenols	26.0	(516)
5	Alkali lignin	H_2_O	Ru/α-HfP	190 °C, 3.5 Mpa H_2_, 3 h	Phenols	25.1	(504)
6	Enzymatic hydrolysis lignin	Ethanol	WO_3_/γ-Al_2_O_3_	320 °C, 0.1 MPa N_2_, 8 h	Alkylphenols	24.5	(510)
7	Kraft lignin	Ethanol	Mo/SEP	280 °C, 0.5 MPa N_2_, 4 h	Phenols	30.6	(507)
8	Kraft lignin	Ethanol	P-Mo/SEP	290 °C, 7.5 MPa N_2_, 4 h	Guaiacols	26.5	(508)
9	Kraft lignin	Ethanol	MnMo/SEP	290 °C, 0.5 MPa N_2_, 4 h	Guaiacols	30.9	(509)
10	Alkali lignin	Isopropanol	RuCu/ZrO_2_	270 °C, 0.1 MPa N_2_, 3 h	Guaiacols	26.9	(517)
11	Pyrolytic lignin	–	Ru/C	400 °C, 10.0 MPa H_2_, 4 h	Alkylphenols	20.5	(518)
12	Kraft lignin	–	NiMoS_2_/C	300 °C, 8.0 MPa H_2_, 10 h	Alkylphenols	26.9	(512)
13	Kraft lignin	–	NiMoP/C	400 °C, 10.0 MPa H_2_, 2 h	Alkylphenols	25.0	(513)
14	Kraft lignin	–	NiMoP/SiO_2_	400 °C, 10.0 MPa H_2_, 2 h	Alkylphenols	30.6	(514)

Regarding studies using an external solvent, alcohols such as methanol and ethanol are mostly used as these have the ability to act as hydrogen donors during the reaction.^501^ The majority of work is exclusive to the use of batch set-ups. Relatively harsh conditions are applied, with temperatures ranging from 190 to 320 °C, pressure from 0.1 to 7.5 MPa, and reaction times from 3 to 24 h. Besides, both precious metal and non-precious metal catalysts have been investigated. Mono- and bimetallic precious metal catalysts have been tested, and examples are Pd- and Ru-based catalysts. For non-precious metals, catalysts in their metallic, oxide, and sulfide forms have been tested for lignin hydrotreatment. The use of sulfided catalysts (e.g., NiMoS and CoMoS) for converting Kraft lignin is reasonable as it contains significant amount of sulfur (2–3%) which maintains the sulfidation state of the sulfided catalysts while this will poison common precious metal hydrotreatment catalysts.^502^

Water, instead of commonly used alcohols, is applied as solvent in several studies, combined with hydrogen donor (e.g., formic acid) or molecular H_2_ for reductive conversions.^503,504^ Onwudili et al. found that Pd/C is active for depolymerization of alkali lignin in subcritical water combined with formic acid (Table 28, entry 1).^503^ The formic acid promotes the hydrolysis of O–CH_3_ ether bonds of guaiacol formed in the subcritical water. Pd/C further catalyzes the hydrogenolysis of aryl–O ether bonds in lignin and the hydrogenation of C=C bonds. In another study by Shu et al. (Table 28, entry 2), Pd/C was used with a Lewis acid catalyst (e.g., CrCl_3_), resulting in high yield of phenols (35.4%).^505^ However, Pd/C combined with a base catalyst (i.e., NaOH) did not lead to such high yields of phenolic monomers.^506^ Non-precious metal oxide catalysts were also investigated, resulting in the finding that tungsten oxide and molybdenum oxide catalysts performed well.^507−510^ The use of Mo/Sepiolite (SEP) led to a high yield of alkylphenols (30.6%) from Kraft lignin (Table 28, entry 7).^507^ Modification of the Mo/SEP catalyst by phosphorus shifts the selectivity to guaiacol, with a yield of 26.5% based on lignin intake (Table 28, entry 8).^508^ This is because the phosphorus addition reduces the strong Lewis acid site on Mo/SEP, and thus inhibits the alkylation reaction. A recent study by the same group (Table 28, entry 9) showed that the addition of Mn has a similar effect and a MnMo catalyst with a molar ratio of Mn: Mo = 3: 1 gave the highest yield of guaiacols (30.9%).^509^

The experimental studies discussed in the previous part were all conducted in the presence of a solvent. However, a solvent-free approach is considered advantageous from a techno-economic point of view, as much higher reactor productivities are possible (about 100 kg m^–3^ h^–1^).^511^ Although no external solvent is used, the molten lignin or liquid degradation products thereof serve as the reaction medium.

An overview of solvent-free catalytic hydrotreatment studies of technical lignins to phenols with high yield is provided in Table 28 (entries 11–14). Both precious metal and non-precious metal catalysts have been used in these exploratory catalyst studies, and all of them were performed in batch mode. Even more harsh conditions were applied compared with catalytic hydrotreatments in the presence of a solvent. Reaction temperatures were between 300 and 400 °C, a pressure between 8.0 and 10.0 MPa, and reaction time between 2 and 10 h. Non-precious metal catalysts (NiMoS and NiMoP catalysts) show a higher yield of desired phenolic products than precious metal ones, and neutral material with high surface area (i.e., active carbon and SiO_2_) were chosen as support (Table 28, entries 12, 13, and 14).^512−514^

#### 3.1.4 Other Strategies for Lignin Conversion to Phenolic Monomers

Several promising reported approaches for the conversion of lignin to phenolic aromatic monomers do not completely fall within the above categories. Among these are emerging biochemical, electrochemical and photochemical methods discussed in more detail below. These strategies include less examples that reach significant yields of aromatic mixtures and/or specific aromatic compounds but do show potential for future development also when applied in the context of lignin-first conversion as elaborated on in section 3.2 especially as in some cases significantly different product portfolio’s can be achieved compared to the above mentioned approaches.

##### 3.1.4.1 Biochemical

The selective production of phenolic chemicals via biological approach can be considered a cornerstone for future biorefining due to the mild conditions used; however, there is a long road ahead for this to become feasible from an economic perspective.^519−521^ There is a significant potential to achieve a variation of products as many natural organisms are known to degrade lignin^522^ and recent developments including those targeting in bacterial fermentation to lipids, muconic acid, pyruvate and polyhydroxyalkanoates show that monomeric phenolic metabolites are intermediates in lignin metabolism of both bacteria and fungi.^523−526^ For example, monomers like syringic acid and ferulic acid have been detected during the degradation of wheat straw lignin by *Aspergillus fumigatus*.^527^ For the lignin depolymerization, organisms have many different enzymes like laccases and peroxidases that induce oxidative degradation. Many of these have been applied for lignin conversion but without recovery of significant yields of aromatics. Enzymes involved in more specific β-O-4 cleavage have been identified that can potentially be used for selective depolymerization.^528−531^ Application of these enzymes showed increased release of monomers, like vanillin from Kraft lignin derived from softwood (Table 29, entry 1).^532^

**Table 29:** Phenolic Products Obtained from Lignin and Lignin-Derived Mixtures by Application of Isolated Enzymes and by Metabolic Engineering of Bacteria

Entry	Feedstock	Organism/enzyme	Product	Yield	Notes	Ref
1	Softwood Kraft lignin	Purified LigD, LigF, and LigG, cloned and expressed in *E. coli*	Guaiacol, ferulic acid, eugenol, acetovanillone, and vanillin	Up to 116 mg/L increase over controls with no enzyme	Hardwood Kraft lignin was tested as well but gave much lower amounts of product	(532)
2	Milled wheat straw	*R. jostii* RHA045	Vanillin, *p*-hydroxybenzaldehyde, vanillic acid, and ferulic acid	2% vanillin and 6–7% total phenolics (assuming 15–20% lignin content)	Kraft lignin was also tested but gave low yields	(534)
3	Supercritical ethanol-depolymerized alkali lignin	*P. putida* KT2440	7 mg/L protocatechuic acid	17.5 wt% of G-type monomers	The input for the biocatalytic conversion contains 77 mg/L monomers	(535)
4	Alkaline-pretreated corn stover lignin	*R. opacus* PD630	Gallate	40.7 wt%	Crude depolymerization mixture was used	(536)
5	Base-depolymerized ammonia fiber explosion (AFEX) corn stover lignin	*R. opacus* PD630	Gallate	63 wt%	Crude depolymerization mixture was used	(536)
6	Base-hydrogen peroxide depolymerized Kraft lignin	*E. coli* DH1	Pyrogallol	7.3 mg/g syringate input	Crude oxidative depolymerization mixture (after filtration) containing syringate was used	(537)
7	Alkaline liquor from corn stover	*S. cerevisiae* yPCA12	810 mg/L protocatechuic acid	From a mixture containing 2030 mg/L *p*-coumaric acid and 370 mg/L ferulic acid	Strain was optimized for *p*-coumaric acid conversion	(538)

**Scheme 134:**
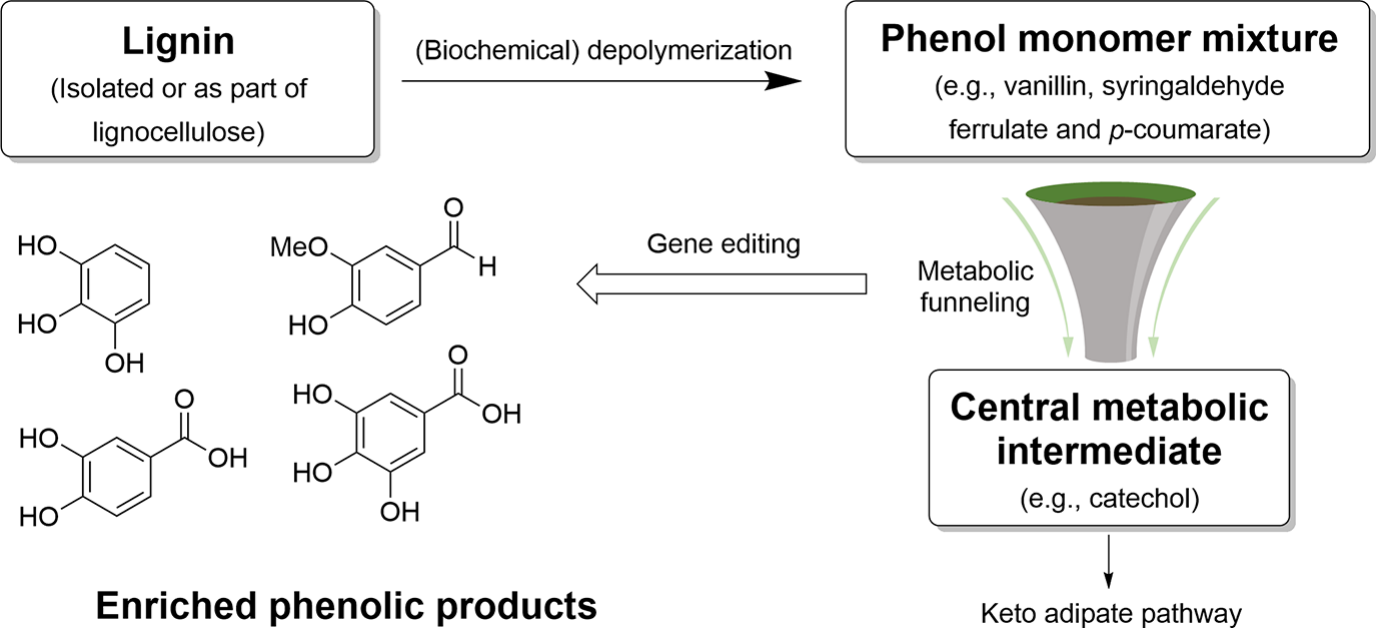
Strategy Toward Phenolic Monomers from Lignin Degradation Using Biochemical Conversion

To target phenolic degradation products, the ability of organisms to funnel the many aromatic intermediates to specific compounds can be exploited.^533^ In the metabolic pathway lignin degradation products are funneled to protocatechuate (3,4-dihydroxybenzoic acid) and catechol which can enter the β-ketoadipate pathway (Scheme 134). When this metabolic pathway is engineered by gene editing, specific intermediates can be targeted and enriched in solution. For example, knocking out a gene coding for vanillin dehydrogenase in *Rhodococcus jostii RHA1* allowed for conversion of efficiency of the lignin of wheat straw (without lignin extraction or chemical pretreatment) of 2% to vanillin and 6–7% to phenolic monomers (Table 29 entry 2).^534^ When combined with chemical pretreatment, protocatechuic acid, gallate, and pyrogallol have been shown to be accessible (Table 29, entries 3–7).^535−538^ Nevertheless, the overall yields of phenolic production using bacteria, fungi, as well as isolated and in-vitro produced enzymes are still low. The right combination of enzymes and organisms still needs to be developed, along with suitable methods for scaling them, for biotechnological approaches to become viable for the production of aromatics from lignin.^533,539^

##### 3.1.4.2 Electrochemical

The current push toward electrification of the chemical industry, as well as its relatively benign and environmentally friendly nature, make electrocatalytic conversion of lignin stand out as an attractive way for conversion to aromatic chemicals.^540^ General approaches are the direct electrooxidation of lignin via a mediator or electrolytic-chemical combination reactions. Most of these are via oxidative pathways, while limited work exists on the direct electroreduction of polymeric lignin to aromatic monomers. Direct electrooxidation of lignin to vanillin has been demonstrated by many different electrode (catalytic) materials and lignins,^541^ although most publications have limited quantification of the released monomeric compounds or report low yields.^542^ Lignin dissolution is typically required for electrooxidation and thus alkali media in which most lignins are soluble are usually applied, although alternatives like IL^543−545^ and DES media^546^ have also been explored. Several examples regarding the conversion of technical lignins have already been discussed in sections 3.1.2 and 3.2.2, with reported yields of up to 23.5 wt% (Table 27 entries 16–20).^493,494,497,498^ In addition, the oxidation of aspen lignin using a Pb/PbO_2_ anode was reported to give several phenolic products as well as 4-methyl anisole in good production rates of up to 343 g/kg lignin per hour and reaching nearly full lignin conversion.^547^ Anisole as well as other aromatics like xylenes and toluene were also reported as part of the product mixture in another study using a Cu/Ni-Mo-Co cathode and a Pb/PbO_2_ anode converting corn stover lignin and using nickel cathode and a Pb/PbO_2_ node.^548,549^ By combined oxidation on an Pb/PbO_2_ anode and reduction using a FeW_9_Cr_4_Mo_3_V cathode, wheat straw lignin could be converted to aromatic monomers associated with reductive depolymerization. A depolymerization mixture rich in alkyl phenols such as propyl guaiacol could be obtained in combination with vanillin.^550^ A combined yield of 123 g/Kg_lignin_ of vanillin syringaldehyde and *p*-coumaric acid was reported from bamboo lignin using a combination of a Cu cathode and a Pb/PbO_2_ anode.^551^ The coproduction of hydrogen and vanillin from alkali lignin by electron abstraction for water splitting mediated by phosphomolybdic acids and visible light irradiation was also shown, although the yields were only 2.5 mg/g lignin.^552^ A microbial fuel cell achieved 11 wt% vanillin production from mild alkali wheat straw lignin likely due to degradation by the formed H_2_O_2_.^553^ Regardless of the significant progress made in this field electrochemical approaches are still facing challenges such low selectivity due to overoxidation and formation of CO_2_ and the limitation in terms of electrolyte solutions.^540^

Addressing selectivity, the reactions taking place at the anode are complex and are further complicated by homogeneous reactions with reactivate oxygen species. Based on the mechanisms for traditional lignin oxidation the main pathway for electrochemical oxidative degradation of lignin is via the oxidation of the α-benzylic hydroxy of the lignin β-O-4 unit to a ketone followed by further oxidation degradation. With mediators such as TEMPO, the oxidation can be directed toward the oxidation of the primary hydroxy groups present in the lignin β-O-4 unit and other linking motifs to aldehydes that lead to further degradation or further oxidation to carboxylic acids. Selective electrochemical oxidation was exploited by Stephenson et al. for a two-step β-O-4 cleavage strategy of mild dioxosolv pine lignin.^554^ Selective benzylic oxidation of lignin was achieved using *N*-hydroxyphthalimide (NHPI)/2,6-lutidine and 1 eq. of lutidine in acetone/DMSO (98:2) to yield a lignin with a 87:13 ratio oxidized vs non-oxidized β-O-4 units. The oxidized lignin was photocatalytically cleaved to yield 1.3 wt% β-hydroxypropiovanillone and 1.1 wt% vanillone (see section 3.1.4.3). The selective oxidation of primary alcohols has been exploited in the work by Stahl et al. discussed in section 3.2.2, where, by applying TEMPO derivative 4-acetamido-TEMPO (ACT), a carboxylic acid rich lignin could be obtained that could be depolymerized to 30 wt% identified phenolic monomers using formic acid. Further developments in scaling and integration of simultaneous oxidation and reduction as well as appropriate technoeconomic assessments are required to increase viability of electochemical routes to aromatics from lignin.^555,556^

##### 3.1.4.3 Photocatalytic

**Scheme 135:**
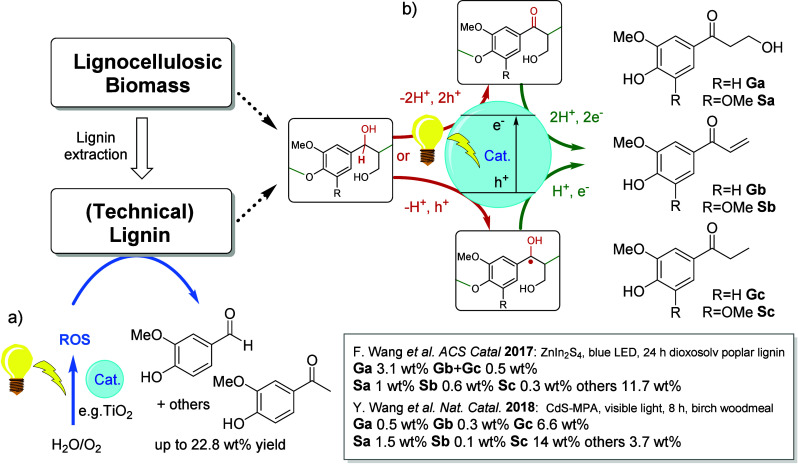
Photocatalytic Approaches to the Production of Aromatic Monomers from Lignin: Oxidative Conversion (a) by Photocatalytic Generation of Reactive Oxygen Species (ROS) and (b) by Photocatalytic Dehydrogenation and Hydrogenolysis

Photocatalysis, especially when making use of solar light, an attractive untapped energy source, can also be applied for lignin depolymerization. Sun light is inherently green and sustainable as well as abundantly available. Most photocatalytic lignin conversion studies are in the context of topics like waste-water treatment and focus simply on lignin degradation and products are small molecules like methanol, formic acid and CO_2_.^557^ Photocatalytic selective cleavage of lignin linkages to obtain aromatics is still in its infancy. Nevertheless, the amount of published studies that fall under this approach is rapidly increasing but still mostly limited to model compound studies as summarized in recent reviews.^558−560^ The studies that have applied photocatalysis do show promise, with the majority of published reports utilizing the photocatalytic generation of reactive oxygen species for oxidative degradation to vanillin and acetolvanillone (Scheme 135a). As shown in section 3.1.2, photocatalytic oxidation using visible light and TiO_2_ gave significant amounts (22.8 wt%) of a mixture of vanillin, homovanillic acid and acetovanillone from milled alkali lignin.^480^ Also, TiO_2_ mediated photooxidation of apple tree pruning ethanosolv lignin with UV light irradiation was shown to yield significant amounts of aromatic monomers.^561^ After reacting 0.5 h 14.2 wt% of syringaldehyde was reported together with smaller amounts of syringol, cathechol, vanillin, and sinapylaldehyde. Increased yields from photocatalytic degradation with TiO_2_ to up to 20 wt% vanillin were reported by combining it with sonochemistry.^562^ Recently, a 5.7 wt% yield of vanillin was reported from the visible light-induced depolymerization of cotton stem lignin using a *n*-butanol/H_2_O emulsion containing H_2_O_2_ and carbon dots immobilized on CuO nanoparticles.^563^

Hydrogen generation using photocatalysis is an important topic in green hydrogen production that has a strong parallel to biorefining. Therefore, the reported 14.7 wt% vanillin yield during hydrogen production from Kraft lignin is an interesting finding that brings these together.^564^ This was achieved using simulated sunlight and nickel coordinated N-doped graphene quantum dots-modified silicon flakes. Additionally, acetovanillone and guaiacol were produced to give a total monomer yield of up to 25.0 wt%. Transfer hydrogenolysis can also be achieved using photocatalysis (Scheme 135b). Wang and co-workers used ZnIn_2_S_4_ catalysts to depolymerize poplar mild organosolv lignin with 9.6 W blue (455 nm) LED light in a 4 to 1 mixture of acetone and isopropanol in an argon atmosphere.^565^ After 24 h at 42 °C, a set of phenolic ketone monomers was obtained with a total yield of 17.2 wt% with **Ga** and **Sa** as main components (Scheme 135). Using model compounds, it was demonstrated that the photocatalyst induces the dehydrogenation of the benzylic hydroxy of the lignin β-O-4 motif to form a hydrogen pool that is utilized to hydrolytically cleave the ether bond in the same lignin motif. A later study showed that the energy band structure could be tuned by controlling the Zn/In ratio.^566^ Here, Zn_4_In_2_S_7_ was found most effective providing a similar set of phenolic ketone monomers in 18.4 wt% yield from birch mild dioxosolv lignin after a 24 h reaction using visible light and methanol under a nitrogen atmosphere.

The photocatalytic degradation of lignin has been extended to lignin-first type biomass conversion. Zhang and Wang et al. showed that cadmium sulfide quantum dots (CdS) with 3-mercaptopropionic acid (MPA) ligand were effective for the selective depolymerization of lignin as part of the lignocellulosic matrix of birch meal.^567^ After 8 h treatment with visible light (420–780 nm) in a 1:1 mixture of MeOH:H_2_O, the product mixture contained phenolic ketone monomers in a total yield of 26.7 wt% with **Gc** and **Sc** (Scheme 135) as major components. Furthermore, the hemicellulose and cellulose remained intact in the residue for separate utilization. The same catalytic system was also able to depolymerize isolated lignins but with a lower monomer yield. A correlation was shown between the β-O-4 motif content and the obtained product yields.^567^

It is doubtful that these photochemical approaches can be scaled up to larger scale as there is no economy of scale in photochemical reactions. In order to retain the same rate, the irradiation surface should increase linearly with the amount of substrate. In addition, the quantum yield needs to be sufficiently high. Most studies do not report a quantum yield.

### 3.2 Lignin-First: Selective Chemocatalytic Lignin Depolymerization to Phenolic Monomers

**Scheme 136:**
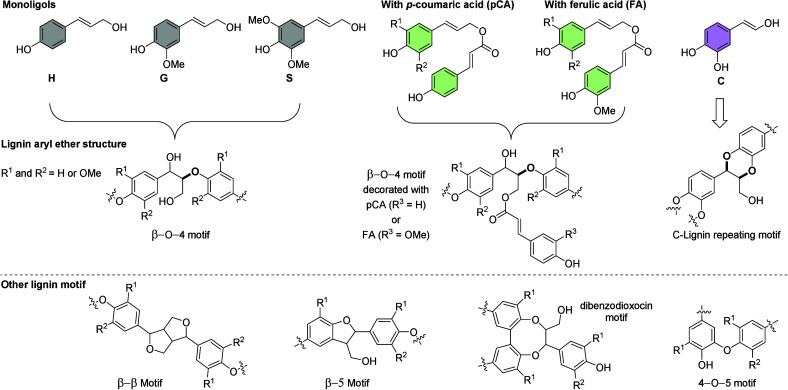
Representative Native Aryl Ether Structures in Lignin

The challenges in the selective conversion of technical lignins to (phenolics) aromatic monomers are mostly related to the complex structure resulting from the condensation processes and other chemical modifications that occur during its removal from the lignocellulose matrix.^417,418^ For this reason, the concept of lignin-first strategies was developed, wherein the objective is to depolymerize the proto or native lignin structure, consisting of aryl–ether bonds connecting monolignols (H, G, and S) (Scheme 136).^419^ The advantage of this approach is that aryl–ether bonds of which the β-O-4 motif is the most dominant, can make up of up to 90% of the bonds linking the monomers in the native lignin structure and these are much easier to cleave selectively compared to condensed C–C bond structures. This means that the actual potential for selective depolymerization to phenolics is limited by the native structure, which can be significantly different for diverse types of biomass. In general, hardwoods which are high in S monomer content have the highest β-O-4 motif content while softwoods that contain mostly G monomers contain more native C–C bonding motifs such as the β-5, the β-β, and the 5-5 motifs. Grassy lignins are constituted of a mixture of all three monolignols, along with decorating structures featuring ferulate and *p*-coumarate. On the other hand, nutshell lignins, such as those from walnuts, can exhibit a particularly high H unit content.^568^ Additionally, they may contain more exotic structures like catechyl structures (C-lignin).^569,570^

Different selective depolymerization methodologies, mainly targeting C–O bonds, are discussed in the sections below. Two types of approaches can be distinguished:^420,571^ (1) the use of a lignin that has been pre-extracted from lignocellulose and (2) the application of lignin depolymerization methodology directly on lignocellulosic material. In the former one wants to retain the aryl–ether C–O bonded structure to facilitate depolymerization. This can be achieved by mild lignin extraction, which usually results in low lignin yields, or by the application of stabilization strategies.^572,573^ In the latter, efficient lignin conversion is typically achieved through the integration of solvolysis, facilitating a catalytic depolymerization strategy that results in stable monomers. The former has the advantage that other biomass components do not interfere; while the latter means that separation and depolymerization is achieved in a single step avoiding undesired secondary reactions like condensation that can occur during the lignin pre-extraction. Overall, the success of such methodologies will depend on the combined value that can be extracted from all the different biomass components. Nevertheless, achieving high yields of a select amount of aromatics leads to a significant opportunity toward high value applications of the lignin component.

#### 3.2.1 Selective Acid-Catalyzed Lignin Depolymerization

Under acidic conditions, the most abundant β-O-4 ether bonds in lignin are prone to undergo hydrolysis resulting in the degradation of lignin. For example, acids are responsible for the β-O-4 cleavage and subsequent solvation of released fragments in sulfite and organosolv pulping. Acid-mediated lignin depolymerization or acidolysis of lignin has been historically used for the separation and elucidation of lignin structure.^574,575^ For example, thioacidolysis is a classical acidolysis method that using EtSH and BF_3_ allows for the quantitative degradation of lignin to thiolated aromatic monomers.^576^ This is used for indirect measurements of the linkage and monomer composition of lignin. Similarly, using the addition of dry HI and CDCl_3_ to ground biomass in an NMR tube, it was shown that 1,3-diiodo-1-(4-hydroxy-3-methoxyphenyl)propane could be obtained as sole depolymerization product in up to 34 wt% from Douglas fir wood and 88 wt% yield of a mixture of monomers from aspen wood, reflecting the biomass lignin monomer composition (both yields based on lignin content).^577,578^

**Scheme 137:**
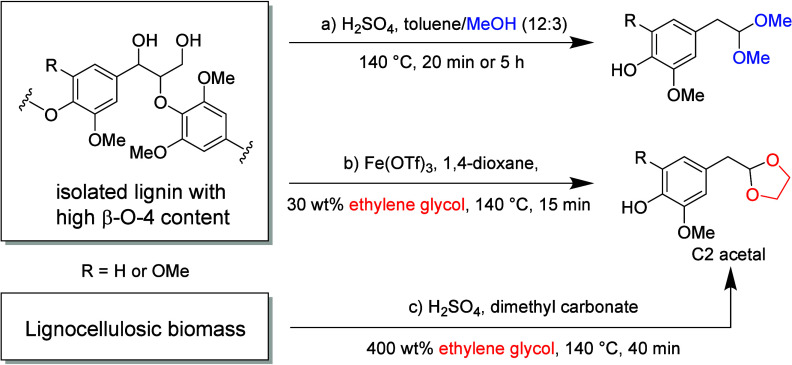
Methanol/Ethylene Glycol-Stabilized Lignin β-O-4 Acidolysis

**Scheme 138:**
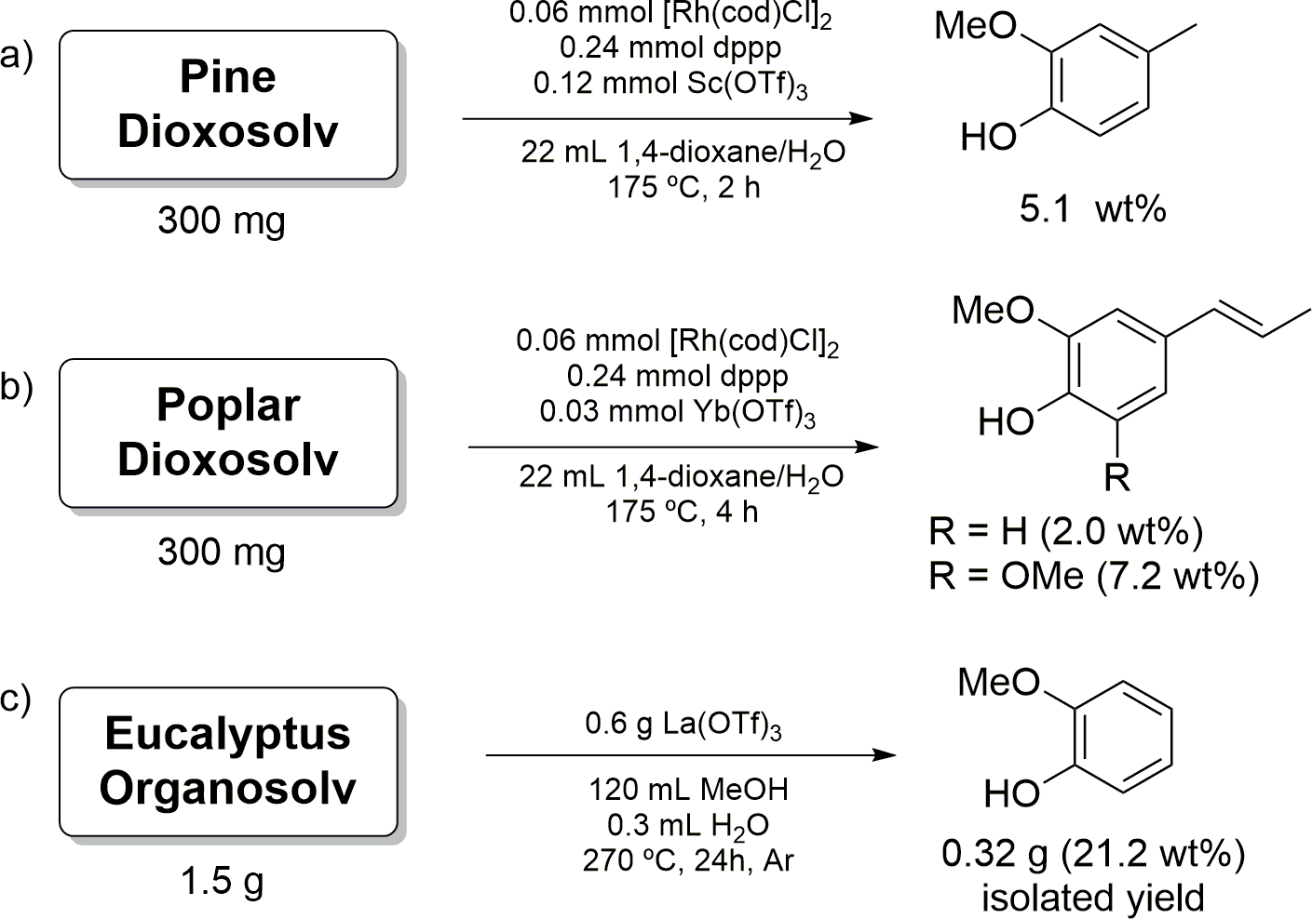
Lewis Acid-Catalyzed Depolymerization of Lignin to (a) Methylguaiacol by Combination of Rhodium-Catalyzed Decarbonylation, (b) Propenyl Phenolics, and (c) Guaiacol

Acid-catalyzed methods are nowadays being further developed for selective lignin depolymerization.^579,580^ During the process of acidolysis, the formed carbocation is very reactive resulting in inter- or intra-molecular condensation reactions via electrophilic aromatic substitution. Furthermore, cleavage products such as aldehydes and ketones are prone to self-condensation reactions, which are responsible for the typically low overall aromatic monomer yields in lignin acidolysis. This can be overcome by alcohol-stabilized acidolysis, adding, e.g., methanol or ethylene glycol during acidolysis, which dramatically suppresses the condensation reactions by *in situ* protecting the formed aldehydes to acetals. Watanabe et al.^581^ performed the acidolysis of Japanese cedar and *Eucalyptus globulus* in toluene with the presence of small amount of methanol as trapping agent, resulting in a nearly 7 wt% production (based on lignin content) of lignin monomer homovanillyl aldehyde dimethyl acetal (Scheme 137a). Depolymerizing lignin to phenolic monomers and utilizing these phenolic monomers for epoxy development is a promising route for sustainable production of epoxy. The same group modified the obtained C2-acetal structure, e.g., via transacetalization and annulation. The modified structures were further used for the synthesis of lignin-based epoxy resins with controllable thermodynamic properties.^582^ Ethylene glycol has also been used for stabilizing the aldehyde products during lignin acidolysis.^583,584^ Barta and Deuss et al.^585^ achieved an excellent yield of 19.3 ± 3.2 wt% for a set of three phenolic C2-acetals from walnut methanosolv lignin under Fe(OTf)_3_-catalyzed, ethylene glycol-stabilized acidolysis. A further thorough screening on 27 lignins, obtained from 13 different pretreatment methods, found that even a maximum of 35.5 wt% combined monomers yield from methanosolv walnut lignin and 16.5 wt% of a single C2-acetal can be achieved from a methanosolv pine lignin via the ethylene glycol stabilized acidolysis (Scheme 137b).^586^ They further combined the diol-stabilized H_2_SO_4_-catalyzed acidolysis with the concept of lignin-first fractionation, achieving the production of 9 wt% single C2-acetal directly from pinewood without prior separation of lignin (Scheme 137c).^587^ In addition, lignin acidolysis can be coupled with *in situ* hydrogenation/dehydration (using Ru/C and H_2_) or decarbonylation (using a homogeneous iridium catalyst) to remove the aldehyde functionality avoiding their condensation, thus promoting monomer yields.^583^ Bruijnincx et al.^588^ utilized water-tolerant Lewis acids (e.g., various metal triflates) for the acidolysis of lignin followed by aldehyde decarbonylation by a homogeneous Rh/dppp catalyst (dppp = 1,3-bis(diphenylphosino)propane). The yield of the decarbonylation product 4-methylguaiacol was 5.1 wt% from poplar dioxosolv lignin (Scheme 138a). Interestingly, the product selectivity can be tuned via changing the acidity of Lewis acid and the source of the lignin. For example, 4-(1-propenyl)phenols became the dominant monomers with Yb(OTf)_3_ as Lewis acid and poplar dioxosolv lignin (Scheme 138b). Han and co-workers showed that selectivity can also be tuned toward guaiacol using Lewis acid catalysts.^589^ With La(OTf)_3_ as catalysts at 270 °C in methanol/water (4/0.01 v/v), 25.5 wt% guaiacol was obtained from pinewood enzymatic mild acidolysis lignin (EMAL) after a 24 h reaction. After column purification, 21.2 wt% pure guaiacol was isolated from Eucalyptus organosolv lignin extracted using 70% isopropanol/water (Scheme 138c).

Solid acid catalysts including immobilized ionic liquids, metal salts, metal oxides, heteropoly acids, and zeolites have also been widely applied for converting lignin to aromatic monomers.^590^ Dhepe et al.^437^ applied various solid acids, including zeolites (H-USY, H-MOR, H-BEA, H-ZSM-5, and clays) and metal oxides (SiO_2_-Al_2_O_3_, sulfonated zirconia, and MoO_3_/SiO_2_). By applying SiO_2_–Al_2_O_3_ as catalyst in H_2_O/CH_3_OH (1/5 v/v) at 250 °C for 30 min, a yield of 30 wt% vanillin was observed. Zeolites have also been applied for the depolymerization of isolated lignins^591^ and Corma and Samec et al.^592^ reported a zeolite-assisted lignin-first fractionation approach for lignocellulose. Using model compounds it was shown that the acidity and the shape selectivity of zeolite are used to convert reactive monomers formed under organosolv pulping conditions to more stable derivatives. The sum yield of typical acidolysis aldehydes was 22.9% in the presence of β-1 zeolite presence compared to 0% in its absence. Under optimized conditions, a yield of 20 wt% of monomers was obtained under organosolv pulping conditions of birchwood. Han and Meng et al.^593^ claimed a phenol production strategy directly from lignin catalyzed by solid acid catalyst HY zeolite (Si/Al = 30) by cleaving both the C_sp2_–C_sp3_ and C–O bonds. A phenol yield of 10.9 wt% was achieved with a selectivity of 91.8% from poplar lignin (a large-scale reaction yielding 4.1 g pure phenol from 50.0 g lignin was also demonstrated). It is noteworthy that the mechanism of solid acid-catalyzed lignin depolymerization appears dramatically different from the lignin acidolysis reactions catalyzed by mineral acids. This discrepancy can be attributed to the diverse and multifaceted catalytic functions exhibited by the solid acid catalyst during the lignin depolymerization process.

#### 3.2.2 Selective Oxidative Lignin Depolymerization

Oxidative depolymerization of lignin yields valuable functionalized monomers. Diverse catalysts, encompassing both homogeneous and heterogeneous, have been employed in the depolymerization of dimeric lignin models as well as native lignin. A comprehensive discussion of these catalytic systems can be found in various recent reviews.^466,477,478,594−596^

Oxidative treatment of lignin is a well-known process (e.g., alkaline aerobic lignin oxidation in basic media with O_2_ or air as oxidizing agent and copper-based catalyst) operated at commercial scale by the company Borregaard (see details in Scheme 146). This is mainly limited to vanillin production from technical lignins or crude residual streams from the traditional paper and pulping industry. As demonstrated in section 3.1.2, the overall carbon yields of vanillin, acetovanillone, vanillic acid, syringaldehyde, acetosyringone, and syringic acid, (see Scheme 131b for the chemical structures and names) depend on the feedstock selection and the specific conditions.

Instead of applying technical lignin, Beckham et al.^597^ re-examined the alkaline aerobic lignin oxidation for native like lignin, approximately 20 wt% syringaldehyde and 17 wt% vanillin can be obtained from high-S popular and pine, respectively.^598^ Electrochemical methods have been reported for the direct oxidation of organosolv lignin. Utley and Viertler et al. reduced the electro oxidation temperature of acetone-soluble spruce lignin by addition of nitrobenzene in aqueous alkaline condition achieving a vanillin yield of 15%.^599^ Furthermore, Sun and co-researchers reported a combined yield of 17.5% for vanillin and syringaldehyde using lignin extracted from sweetgum using an ethanosolv method.^600^ Notably, they employed nickel foam as an economical electro-catalyst in alkaline conditions to facilitate this transformation.

**Figure 17:**
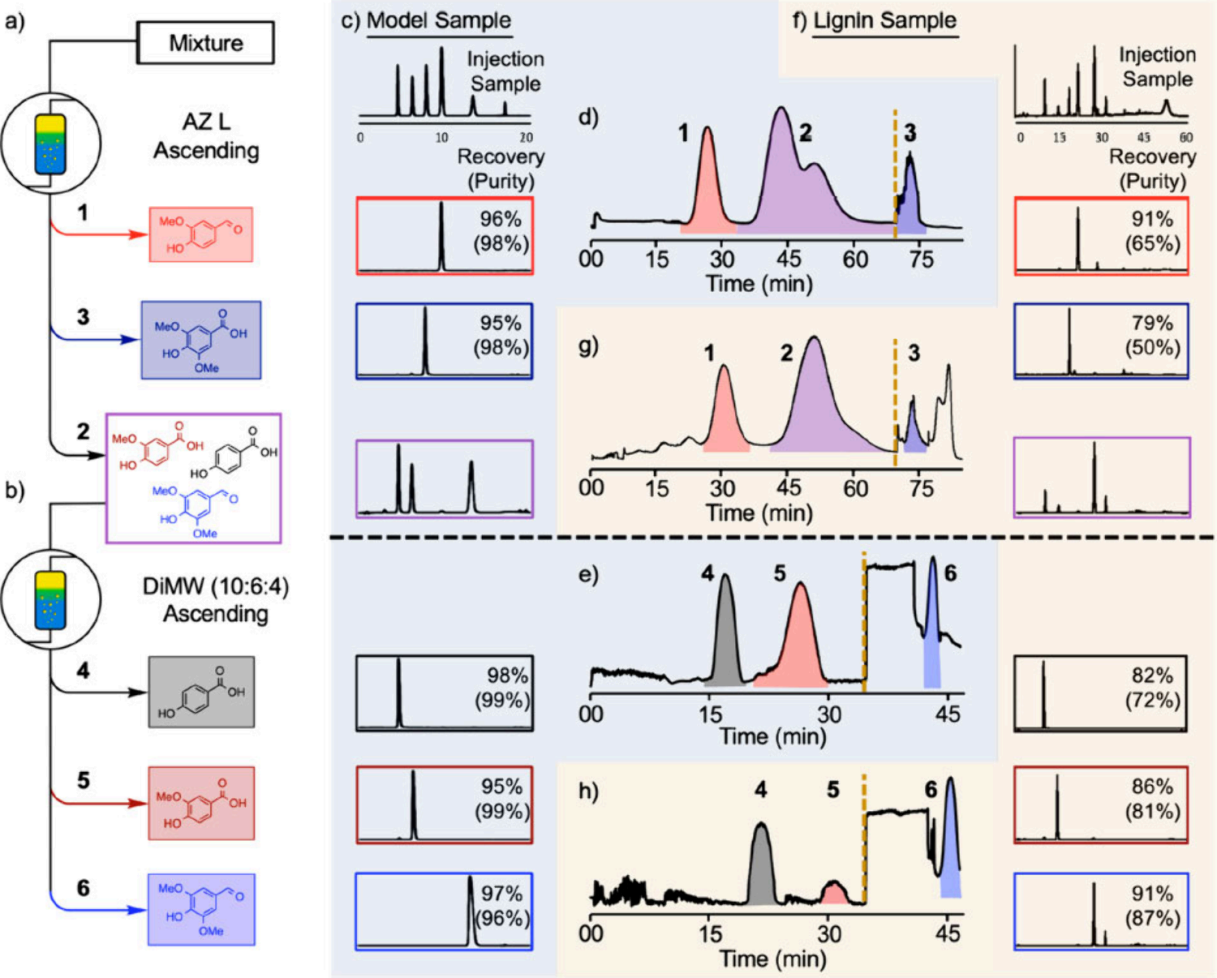
Two-stage centrifugal partition chromatography (CPC) separation sequence enabling isolation of aromatic compounds in a model sample and authentic lignin oxidative alkaline depolymerization mixture. Reproduced with permission from ref (1275). Copyright 2022 American Chemical Society.

Aqueous alkaline conditions are known to enhance the efficiency of pulp production processes and lignin depolymerization. Miyafuji et al.^484^ reported tetrabutylammonium hydroxide 30-hydrate (Bu_4_NOH·30H_2_O) as new alkaline reaction media instead of NaOH for lignin alkaline aerobic oxidative degradation without the presence of additional catalyst. The degradation of milled wood lignin in Bu_4_NOH·30H_2_O at 120 °C for 43 h yielded vanillin and vanillic acid at the yield of 11.7 and 4.1%, respectively. A further enhanced vanillin yield of 15.4% was achieved on Japanese cedar wood flour under the same reaction conditions. The process of oxidative depolymerization of lignin results in the generation of a diverse array of functionalized monomers, posing a challenge of intricate separation as also discussed in section 3.1.2. Stahl et al.^1275^ reported a two-stage centrifugal partition chromatography (CPC) strategy to isolate monomers from the lignin alkaline oxidative depolymerization product. Utilizing the resultant mixtures obtained from multiple lignin oxidative reactions within the context of this separation methodology (comprising a total mass of 1.6 grams), the composition is as follows: 25.5 wt% corresponds to aromatic monomers, encompassing 4.0 wt% of pHBA (*p*-hydroxybenzoic acid), 5.0 wt% of vanillin, 1.2 wt% of vanillic acid, 0.6 wt% of acetovanillone, 11.2 wt% of syringaldehyde, 2.1 wt% of syringic acid, and 1.4 wt% of acetosyringone. In terms of recovery, the separation strategy achieves percentages of 82% for *p*HBA, 91% for vanillin, 86% for vanillic acid, 91% for syringaldehyde, and 79% for syringic acid (Figure 17).

**Scheme 139:**
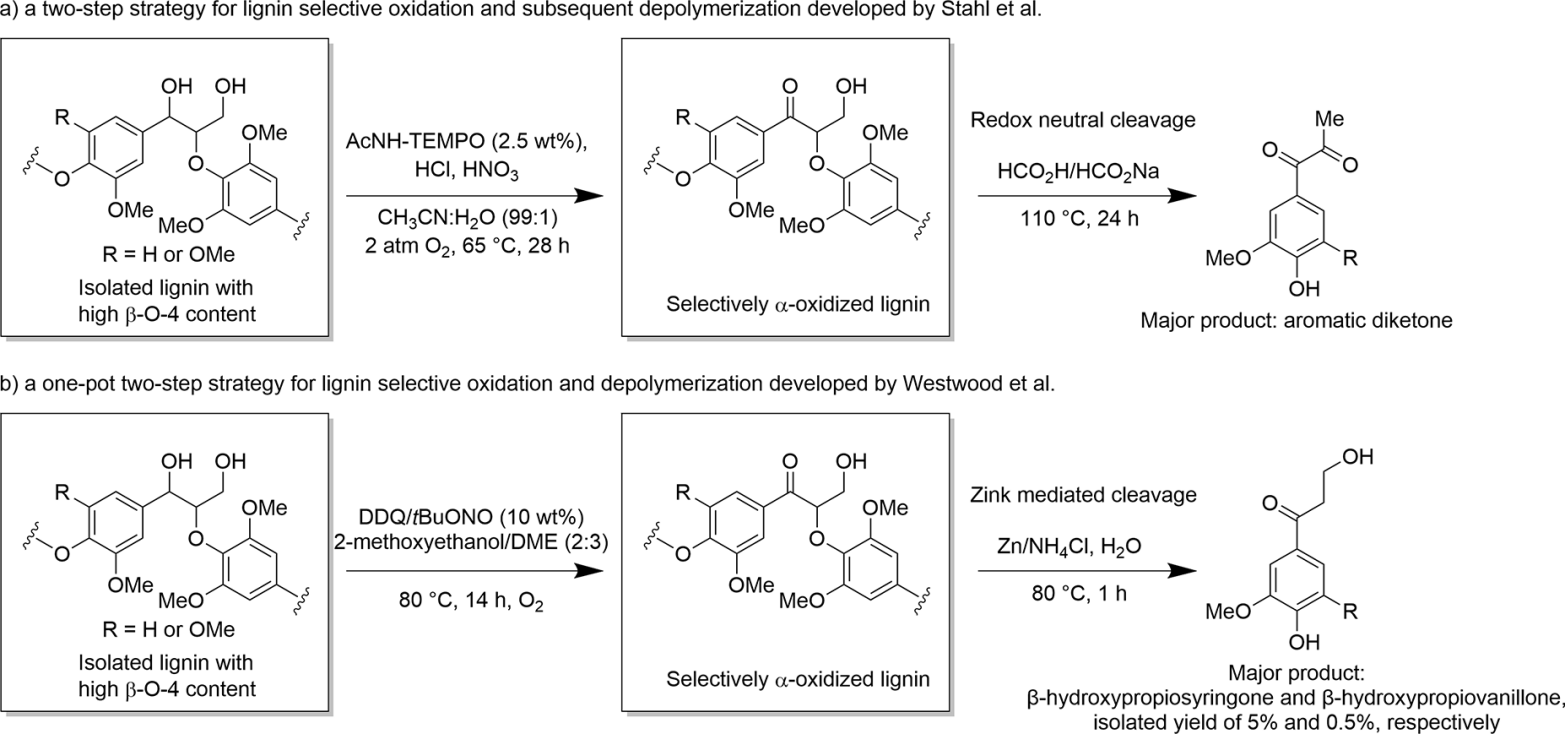
Selective Oxidation of Lignin β-O-4 Motif and Its Subsequent Depolymerization

When using lignin with a more regular structure other oxidative approaches become viable. New approaches have been developed for selective depolymerization of lignins with high aryl-ether content or lignin as part of the lignocellulosic matrix. Some two-step (separately or in one-pot) lignin valorization strategies have been reported focusing on the abundant β-O-4 linkage. Typically, the first step involves oxidizing the alcohol group of the side chain. The selective α-oxidation (2° benzylic alcohol oxidation) leads to a decrease of the dissociation energy of the C–O bond in the β-O-4 link motif, while the γ-oxidation (1° alcohol oxidation) significantly weakens the C_α_–C_β_ bond, thereby facilitating its breakdown.^594^ The oxidation of primary (γ-OH) and secondary (α-OH) alcohols can be selectively achieved, which allows different further degradation products following different reaction paths.^601,602^ Stahl et al. reported a formic-acid-induced lignin depolymerization strategy toward aromatic diketones.^603^ This relied on firstly oxidizing the secondary alcohol of the lignin β-O-4 motif using TEMPO derivatives as catalysts in combination with an oxygen atmosphere. Subsequently, the oxidized lignin was subjected to the formic acid/formate reaction conditions to facilitate the C–O bond cleavage (Scheme 139a). Applying an oxidized aspen lignin, the aromatic diketone derived from the S and G units in lignin can reach up to 13.1 wt% and 6.7 wt%, respectively, with a total monomer yield of 52.2 wt% including aromatic diketones stemming from the G and S units, vanillin, syringaldehyde, etc. Furthermore, a range of lignins obtained from this process were assessed for this oxidation-hydrolysis sequence, from which poplar lignin isolated via an acid pretreatment gave 16.8 wt% of S and G unit-derived diketones.^604^

**Scheme 140:**
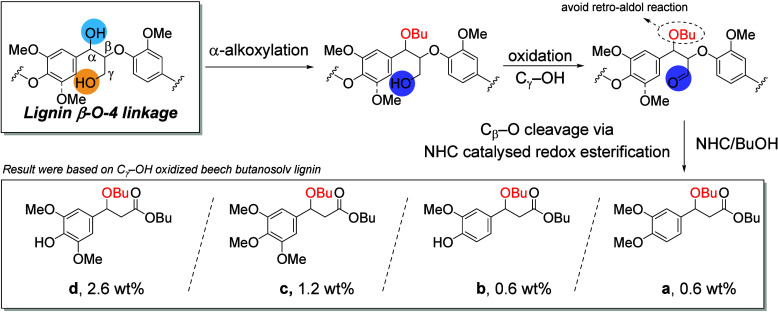
Selective Conversion of α-Butoxylated, γ-Oxidized Lignin

Luterbacher et al. reported a formaldehyde-stabilized lignin extraction method by using formaldehyde as α,γ-diol-protecting reagent to preserve the β-O-4 interunit linkage forming an acetal structure (details *vide infra*, structure see Scheme 143, structure B), as such lignin can be extracted with high β-O-4 content with the potential of achieving a high monomer yield in the following depolymerization.^605^ A further deprotection and oxidation step was developed using 2,3-dichloro-5,6-dicyano-1,4-benzoquinone (DDQ) as oxidant/catalyst to deprotect the acetal and oxidize the α-OH into a ketone, after which the deprotected and oxidized lignin was subjected to the formic acid/sodium formate system (method reported by Stahl et al. in Scheme 139a) for monomeric aromatic diketone production.^606^ With a propylidene acetal protected lignin extracted from F5H poplar (a genetically modified poplar with high syringyl content in lignin) going through the deprotection and simultaneously oxidation, and the depolymerization step, the single diketone yield released from S units reached 44 wt% based on Klason lignin basis. Westwood et al. reported a two-step one-pot strategy to selectively depolymerize lignin to functionalized phenolic monomers (Scheme 139b).^607^ The first selective secondary (α-OH) alcohols oxidation step was achieved in a the DDQ/*t*BuONO/O_2_ system at 80 °C, while the second C–O cleavage step was promoted by Zn/NH_4_Cl in water at 80 °C. Starting from a mild dioxosolv birch lignin, 5 wt% of β-hydroxypropiosyringone was isolated as major product after extraction and purification by column chromatography. In a followup report by Westwood et al.,^608,609^ this DDQ protocol to obtain lignin^OX^ was studied for its reactivity of not only β-O-4 but also β-β, β-5 and lignin-bound Hibbert’s ketones providing insight into the nature of the secondary aromatic fragments that are released. The oxidation of the secondary benzylic alcohol of the lignin β-O-4 linkages can also be achieved by a mechanochemical approach. Su et al. reported the successful selective oxidation of the benzylic alcohol of lignin β-O-4 linkages to the corresponding ketone using 2,3-dichloro-5,6-dicyano-1,4-benzoquinone (DDQ)/NaNO_2_ as oxidant under milling conditions.^610^ The subsequent base (NaOH)-catalyzed cleavage of the C_β_–O bonds and C_α_–C_β_ bonds of lignin β-O-4 ketones delivered monomeric phenols under milling conditions. With birch lignin, this two-step mechanochemical procedure achieved 4.8 wt% of syringic acid. In addition, the secondary benzylic alcohol oxidation in lignin β-O-4 can be coupled with reductive cleavage. Wang et al. developed a two-step oxidation–hydrogenolysis strategy for lignin toward phenolic monomers.^611^ The first benzylic alcohol oxidation step was performed with O_2_/NaNO_2_/DDQ/NHPI, while the second hydrogenolysis step was catalyzed by NiMo sulfide. By employing this two-step methodology on birch lignin, a 32% yield of phenolic monomers was successfully obtained including monomers such as propyl syringol/guaiacol and propenyl syringol/guaiacol. The selective oxidation of the primary alcohol of lignin β-O-4 motif can also be targeted. Deuss et al.^612,613^ achieved selective primary alcohol oxidation to the corresponding aldehyde in the lignin β-O-4 motif using an ethanolsolv lignin. Westwood et al.^614^ documented the specific γ-oxidation of the butoxylated β-O-4 units within butanosolv rice husk lignin, resulting in the formation of γ-carboxylic acids. These two approaches both rely on the masking of the benzylic alcohol by ethoxylation of the benzylic position in the lignin β-O-4 motif. Applying the γ-oxidized lignin, Westwood et al.^615^ further depolymerized it via *N*-heterocyclic carbene (NHC)-catalyzed redox esterification (Scheme 140). As a result of the latter, 4 types of novel aromatic monomers preserving the C3 side chain were obtained (compound **a**–**d** in Scheme 140), among which monomer **d** demonstrated an isolated yield of 2.6 wt%. Without protection of the α-OH, the selective oxidation of the γ-OH to γ-aldehyde in lignin β-O-4 motif can easily trigger the retro-aldol reaction cleaving the C_α_–C_β_ bond.^616,617^ This allows for one-pot conversion of lignin β-O-4 models or lignins; however, high single monomer yield was not observed upon application on isolated lignins.

**Scheme 141:**
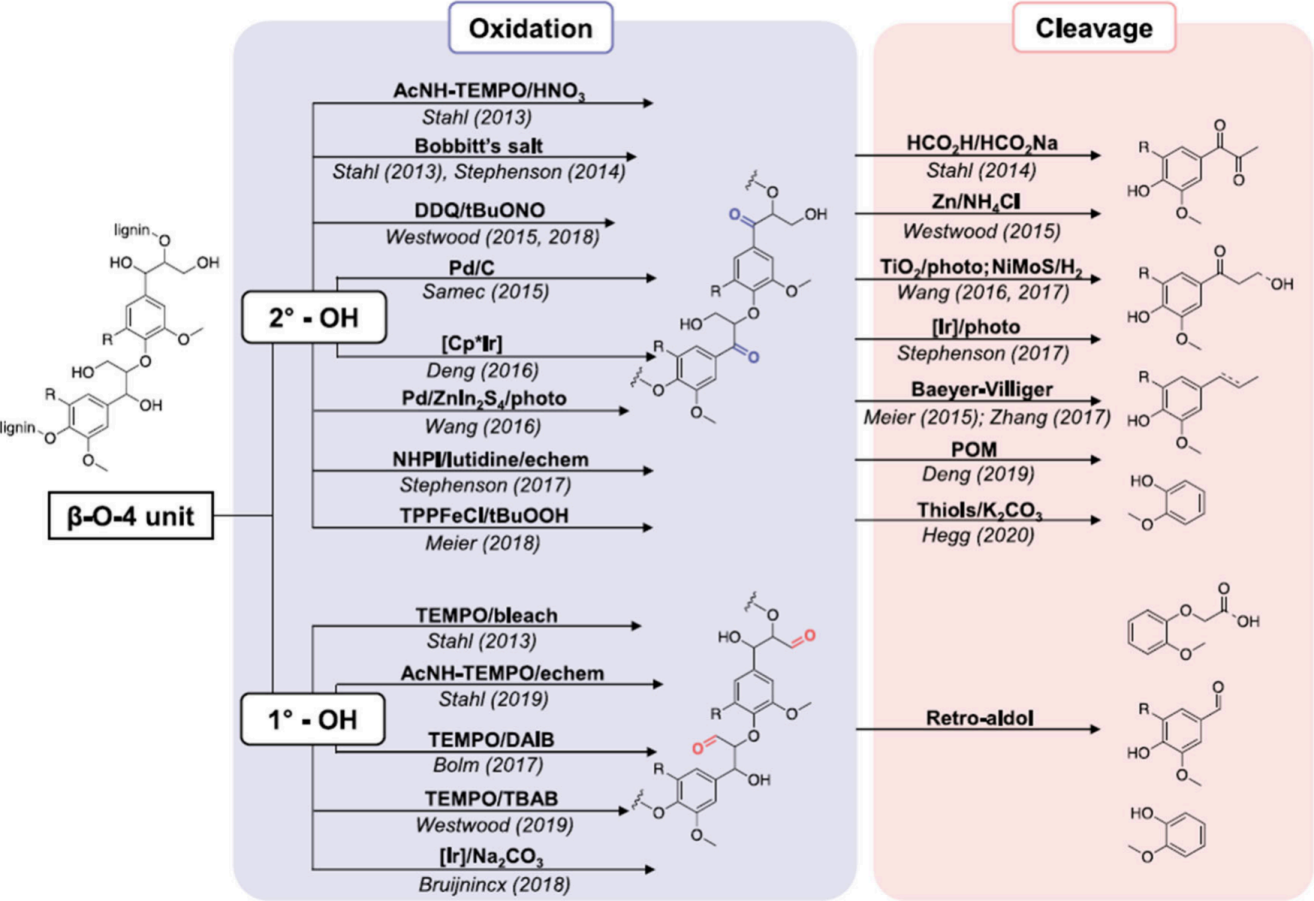
Overview of the Sequential Oxidation–Cleavage Approaches Applied on Lignin Reproduced with permission from ref (601). Copyright 2021 Elsevier.

Stahl et al.^618^ showed the selective electrochemical conversion of primary alcohols in lignin to carboxylic acids, using 2,2,6,6-tetramethyl-1-piperidine-*N*-oxyl (TEMPO) and 4-acetamido-TEMPO (ACT) as catalytic mediators under mild basic conditions. This approach was applied to poplar wood-derived lignin, yielding a distinct polyelectrolyte material with similar molecular weight but increased acid content and water solubility; further acid treatment leads to depolymerization into nearly 30 wt% of characterized aromatic monomers using H_2_O/HCO_2_H. In these obtained monomers, the yield of syringaldehyde and syringic acid reached 7.8 wt% and 6.8 wt%, respectively. In the context of sequential oxidation-depolymerization methodologies aimed at lignin conversion, Stahl et al.^601^ comprehensively outlined the oxidation techniques employed on isolated lignin samples, as indicated in Scheme 141. For a comprehensive understanding, we direct the reader to consult the detailed review provided by these authors.^470,601^

Due to the macromolecular structure of lignin, achieving good solubility of lignin is of vital importance to enhance its depolymerization efficiency. Sun and Yu et al. developed a methanol/choline chloride (MeOH–ChCl)-based deep eutectic solvent (DES) to improve the solubility of lignin and the redox potential of the catalyst Cu(OAc)_2_/1,10-phenanthroline.^619^ By employing alkaline lignin as the substrate, they claimed noteworthy yields of 40.1% acetovanillone and 40.6% acetic acid, showcasing the effectiveness of this oxidation catalytic system.

Oxidative lignin-first fractionation strategies have also been explored. Stahl et al.^598^ coined oxidative catalytic fractionation (OCF) which was performed utilizing a heterogeneous Co-N-C catalyst, along with O_2_ as the oxidizing agent and acetone as the solvent. This procedure, applied on poplar, leads to the production of phenolic compounds containing aldehyde moieties (such as vanillin and syringaldehyde) as well as carboxylic acids (including *p*-hydroxybenzoic acid, vanillic acid, and syringic acid), achieving a yield of 15 wt% (reaction conditions: 10 wt% Co-PANI-C (polyaniline (PANI) as the nitrogen source), 190 °C, 35 bar 6% O_2_ in N_2_, 12 h). Deng et al.^620^ used a polyoxometalate (POM) catalyst for the OCF of wood sawdust. Their method relies on methoxylation of the active α-OH groups of lignin in a methanol and water mixture at low temperature (100 °C), subsequently elevating the temperature to 140 °C; yielding 45.9 wt% aromatic monomers including vanillin, syringaldehyde, methyl vanillate, methyl syringate, and methylparaben (methyl 4-hydroxybenzoate). CuO nanoparticles (NPs) were also reported to be able to catalyze the OCF of lignocellulose.^621^ Under 1 MPa O_2_ in 7.5 wt% aqueous NaOH at 160 °C for 1 h, up to 48.6 wt% aromatic aldehyde monomers, mainly including vanillin and syringaldehyde, were obtained from Eucalyptus.

In addition to the aforementioned thermocatalytic oxidative cleavage processes, photocatalytic lignin-first strategies have been demonstrated as described in section 3.1.4.3 and outlined in Scheme 135b.

#### 3.2.3 Selective Reductive Lignin Depolymerization

The catalytic conversion of lignocellulosic biomass under reductive conditions is one of the oldest approaches used for lignin structure elucidation, by which the 4-*n*-propylphenol nature of the lignin got confirmed.^622−624^ A chronological overview on the reductive depolymerization of lignin is well summarized in a recent review by Korányi and Barta et al.^571^ In recent years, this approach has been rediscovered with many new developments even aiming toward commercialization. The reductive catalytic depolymerization of lignin typically yields alkyl phenolics. However, it can also be tuned toward cycloalkanes or more functionalized alkylphenolics by depolymerization of extracted lignins that are rich in cleavable aryl ether linkages such as the β-O-4 by milder depolymerization technologies or by lignin extraction with stabilization strategies. In addition, the reductive depolymerization of lignin can occur as part of the lignocellulose matrix, in accordance with the lignin-first concept. This method involves the catalytic fractionation of biomass and the subsequent reductive catalytic depolymerization of lignin in one pot. In simpler terms, it focuses on extracting lignin through solvent-based techniques (with potential aid of a catalyst), which is followed by immediate depolymerization of the extracted lignin under reductive conditions by a catalyst and a reducing agent. Lignin structural differences can be determined via the reductive process by carefully analyzing the product distribution.^572^

##### 3.2.3.1 Reductive Depolymerization of Isolated Lignins

**Scheme 142:**
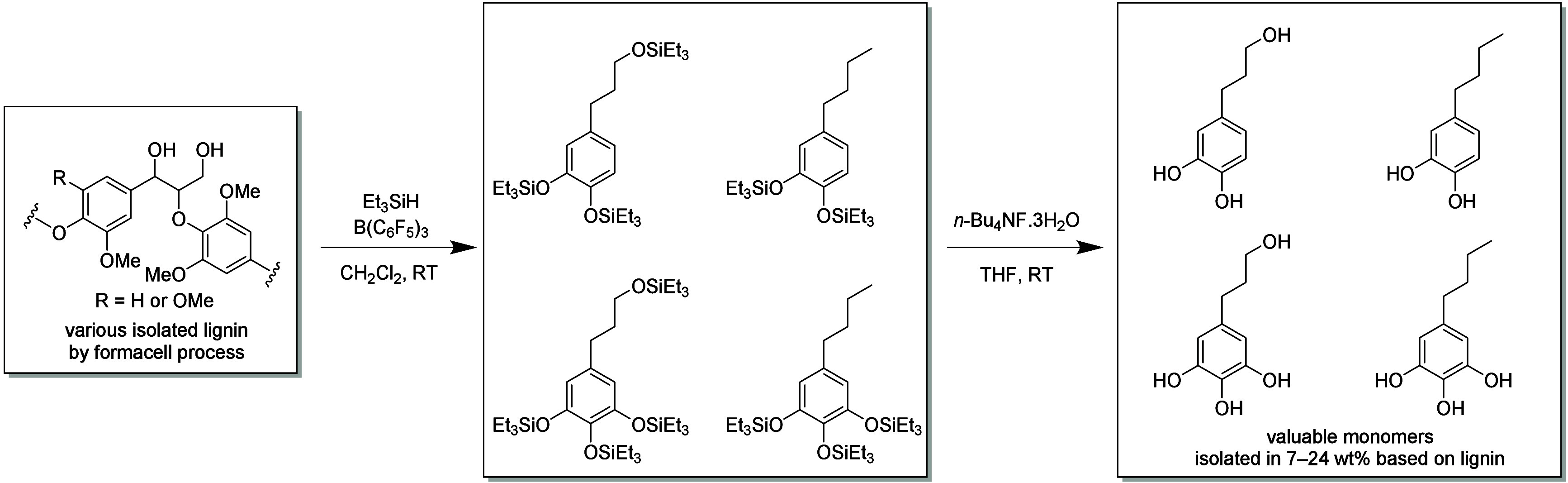
Convergent Reductive Depolymerization of Lignin to Isolated Phenol Derivatives by Metal-Free Catalytic Hydrosilylation

Different from the industrial lignin with alternated and condensed structure which has been discussed in detail in section 3.1.3, the more native-like lignin brings opportunities to obtain specific monomers in high selectivity and yield under reductive conditions. The hydroconversion of industrial condensed lignin has been extensively researched and in general necessitates harsh conditions (high temperature and high H_2_ pressure). Inert solvents, such as dodecane and 1-methylnaphthalene, can be employed to enhance interactions between solid, liquid and gas. However, these solvents have proven to be less effective in terms of depolymerization efficiency and complicate the product recovery.^596^ For isolated lignin with better preserved C-O linkages, the reductive depolymerization is usually performed in solvents able to act as hydrogen donors, e.g., alcohols, water, formic acid, and tetralin. Besides their hydrogen donating ability, these solvents have synergistic functions including solvation, catalytic effects, and participation in reactions. Barta et al.^625^ investigated the reductive depolymerization of purified organosolv candlenut lignin catalyzed by Cu-doped porous metal oxides (Cu-PMO) in methanol. A series of substituted catechols were identified with a yield of 63.7%, among which the major catechol: 4-(3-hydroxypropyl)catechol was isolated in a yield of 43.3% by column chromatography (Table 30, entry 1). The observed product distribution indicates the presence of C-lignin in the candlenut substrate used (more examples on reductive depolymerization of C-lignin are overviewed in section 3.2.3.2). Cantat et al.^626^ reported a reductive depolymerization of lignin under metal-free conditions employing hydrosilanes as reductants and B(C_6_F_5_)_3_ as a Lewis acid catalyst (Scheme 142 and Table 30, entry 2). One of the advantages of this method is the high selectivity to a limited number of species in the product mixture which allows easy separation. In addition, valuable 4-(3-hydroxypropyl)benzene-1,2,3-triol or 4-(3-hydroxypropyl)-1,2-benzenediol can be obtained. Recently, this catalytic system was applied on C-lignin achieving silylated catechol derivatives in 85% yield.^627^ Wang et al.^628^ reported a synergistic single-atom catalyst (SAC) by integrating atomically dispersed Mo centers and Al Lewis acid sites onto an MgO substrate (Mo_1_Al/MgO).^628^ The designed synergistic catalyst demonstrated high activity for Eucalyptus lignin depolymerization in the H-donor solvent methanol, without the use of external H_2_. This process delivered 46% monophenolic monomers, with a notable selectivity to coniferyl and sinapyl methyl ether at 200 °C under N_2_ (Table 30, entry 3). Interestingly, lignin can also be depolymerized without the addition of a transition-metal catalyst. Abu-Omar et al.^629^ demonstrated that (*E*)-4-propenyl syringol and isoeugenol were obtained from organolsolv poplar lignin with a yield of 33.6% at 270 °C for 4 h in 50:50 (v:v) EtOH/*i*-PrOH (Table 30, entry 4). Such a high yield is surprisingly comparable to those obtained with most transition-metal catalysts. Other reports on selective lignin reductive depolymerization, using various catalytic approaches exhibiting high reported monomer yields are summarized in Table 30, entries 5–20.

**Table 30:** Overview of the Mild Selective Catalytic Reductive Depolymerization of Isolated Lignin with High Aryl Ether Content to Phenolic Compounds

Entry	Source and scale	Catalyst and ratio of cat/substrate	Solvent	Conditions	Yield	Major product	Ref
1	Organosolv lignin from candlenut; 1 g	Cu-PMO; 0.5:1	Methanol	140 °C, 4 MPa H_2_, 20 h	Up to 63.7% catechols	43.3% Isolated yield 4-(3-hydroxypropyl)catechol	(625)
2	Formacell lignin from hybrid plane; 400 mg	305 wt% Et_3_SiH, 30 wt% B(C_6_F_5_)_3_	CH_2_Cl_2_	RT, 20 h	33% Phenolic monomers including 5-propylpyrogallol and 5-(3-hydroxypropyl)benzene-1,2,3-triol	Isolated 5-(3-hydroxypropyl)benzene-1,2,3-triol at yield of 24%	(626)
3	*Eucalyptus* lignin; 300 mg	Mo_1_Al/MgO; 0.33:1	MeOH	200 °C, N_2_ 1 MPa, 8 h	46% Phenolic monomers	92% Selectivity to coniferyl and sinapyl methyl ether	(628)
4	Organosolv poplar lignin; 180 mg	None	Ethanol:isopropanol (50:50, v/v)	270 °C, N_2_ 1 MPa, 4 h	Overall oil yield of 70 wt%	48% Selectivity to (*E*)-4-propenyl syringol and isoeugenol	(629)
5	Cellulolytic enzyme lignin from bamboo; 0.5 g	Different zeolites mixed with Raney Ni; 1.5:1	H_2_O/CH_3_OH (2:5, v/v)	270 °C, 0.1 MPa N_2_, 30 min	21.0–27.9% Phenolic monomers depending on the zeolite	12.4% of 2-(4-hydroxy-3,5-dimethoxyphenyl)acetic acid with Hβ-zeolite	(515)
6	Organosolv birch lignin; 50 mg	Ni_7_Au_3_, NaOH in 2.7 equiv	H_2_O	160 °C, 1 MPa H_2_, 4 h	10.9% Phenolic monomers	6.1% 4-*n*-Propanolsyringol	(630)
7	THFA beech lignin; 100 mg	Ni/C; 1:1	THFA^a^/1,4-dioxane (1:1, v/v)	220 °C, 2 MPa H_2_, 5 h	14.7% Phenolic monomers	9.3% 4-*n*-Propanolsyringol	(631)
8	Enzymatic mild acidolysis lignin from willow; 50 mg	Nanostructured MoOx/CNT; 0.1:1	CH_3_OH	260 °C, 3 MPa H_2_, 4 h	38.7% Phenolic monomers	Approximately 18–19% unsaturated 4-*n*-propenylsyringol	(632)
9	Empty fruit bunch lignin; 300 mg	Ru/H_Β_; 0.33:1	Ethanol/water (0.65:0.35 v/v)	250 °C, 4 MPa H_2_, 6 h	15.9% Monophenolic monomers	Primary product is 4-*n*-propylguaiacol	(633)
10	Sugarcane bagass organosolv lignin; 500 mg	Ni/MgO, Ni/ZrP; 0.2:1	Isopropanol	270 °C, 3 MPa H_2_, 4 h	15.0% Monophenolic monomers	42.3% Selectivity to 4-ethylphenol	(634,635)
11	Birch lignin; 100 mg	Ru/Nb_2_O_5_; 2:1	H_2_O	250 °C, 0.7 MPa H_2_, 20 h	35.5% C7–C9 arenes	Ethylbenzene with yield of 9.1% and propylbenzene with a yield of 8.5%	(636)
12	Organosolv poplar lignin	Ni/C with addition of H_2_SO_4_ and *p*-hydroxybenzyl alcohol as capping agent	Methanol	160 °C, 1 h	20.4% Phenolic monomers	Acetosyringone and ferulic acid methyl ester with highest peak areas	(637)
13	Birch lignin; 200 mg	Ni_50_Pd_50_/SBA-15; 0.5:1	Isopropanol/H_2_O (2:1, v/v)	245 °C, 8 h	18.5% Monophenolic monomers	8.9% 4-*n*-Propylsyringol	(638)
14	Organosolv birch lignin; 0.3 g	Ru@N-doped carbon; 0.2:1	Ethanol/H_2_O (1:1, v/v)	300 °C, H_2_ 1.0 MPa, 2 h	30.5% Phenolic monomers	Around 13% 4-*n*-propylsyringol	(639)
15	Organosolv birch lignin; 100 mg	Ni_1_-Fe_1_/Ac; 0.5:1	Methanol	225 °C, H_2_ 2 MPa, 6 h	23.2 wt% Phenolic monomers	4% *4-n*-Propylguaiacol and 14% 4-*n*-propylsyringol	(640)
16	Organosolv birch lignin; 50 mg	Ni_7_Au_3_	H_2_O	170 °C, H_2_ 1 MPa, 12 h	14.2 wt% Phenolic monomers	9.3% 4-*n*-Propanolsyringol	(641)
17	Lignin; 100 mg	Br-Ru/C; 1:1	MeOH	180 °C, H_2_ 0.5 MPa, 6 h	26 wt% Phenolic monomers	Approximately 20% selectivity to methyl *p*-hydroxy-hydrocinnamate	(642)
18	Poplar lignin; 1 g	Ni_0.5_Co_0.5_/C; 0.5:1	MeOH	260 °C, N_2_ 1 MPa, 4 h	55.2% Monophenolic monomers	Approximately 40% selectivity to guaiacol	(643)
19	Corncob enzymatic hydrolysis lignin; 50 mg	None	9,10-Dihydroanthracene	400 °C, N_2_ 0.1 MPa, 10 min	Up to 20.2 wt%	Alkylphenols as major product	(644)
20	Eucalyptus enzymatic mild acidolysis lignin; 50 mg	Pd/C; 0.1:1	MeOH	180 °C, H_2_ 3 MPa, 4 h	44.1%	76% Selectivity to propanol-guaiacol/syringol	(645)

a THFA = tetrahydrofurfurylalcohol

##### 3.2.3.2 Reductive Depolymerization of Isolated Lignin with a Modified Structure

**Scheme 143:**
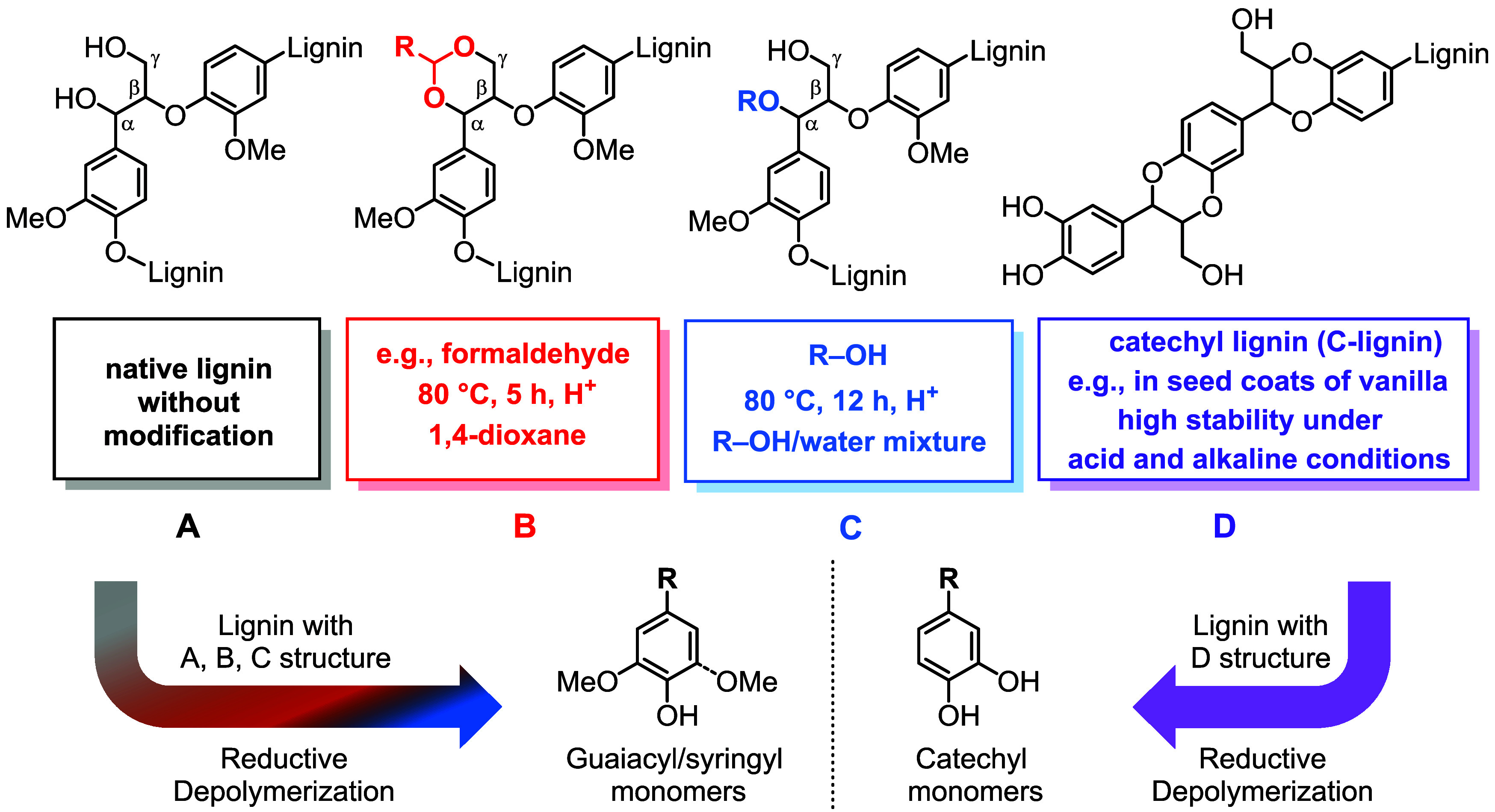
Lignin Structures with Preserved High C–O Bonds Content and Their Depolymerized Products

During the extraction of lignin or the pretreatment of biomass, the formation of recalcitrant C–C bonds poses a challenge for further depolymerization. Stabilization strategies can be applied to preserve the most abundant β-O-4 linkages.^646,647^ Luterbacher et al.^605^ applied formaldehyde as the stabilization reagent to react with 1,3-diols (α- and γ-hydroxyl groups on the β-O-4 motif) forming a six-membered 1,3-dioxane structure (Scheme 143, structure B).^646,647^ This approach avoids severe condensation of the β-O-4 linkage by preventing formation of the reactive carbocation intermediate. Subsequent hydrogenolysis of the extracted lignin under formaldehyde stabilization using Ru/C and 40 bar H_2_ delivered guaiacyl and syringyl aromatic monomers at near theoretical yields. Specifically, for the extracted beech lignin, a monomeric yield of 47% was attained (Table 31, entry 1). In contrast, the gene-modified F5H poplar lignin, which exhibited a high syringyl unit content (98.3%) along with reduced native interunit C–C linkages, yielded a remarkable 78% of phenolic monomers. The same group further extended the lignin extraction procedure with other diol protecting reagents with the potential formation of cyclic acetals, ketals, carbonates, and boronates by reacting with the α,γ-diol in lignin, respectively. In particular, lignins protected by acetaldehyde and propionaldehyde were found to reach high selectivity to aromatic monomers upon hydrogenolysis without the aromatic alkylation observed with formaldehyde.^648^ Facilitated by Pd/C catalysis, lignin monomers were obtained with yields approaching theoretical values based on Klason lignin. The monomeric yields were 48% for birch, 20% for spruce, and an impressive 70% for the high-syringyl transgenic poplar (Table 31, entry 2). Notably, a high 80% selectivity toward a singular 4-*n*-propanolsyringol product was achieved in the case of the F5H-poplar. Furthermore, hydrogenolysis of propanal-stabilized lignin was demonstrated for endocarp waste biomass^649^ as well as in a continuous flow reactor (Table 31, entry 3, 4).^650^ Monophenolic monomers were produced in 45% and 40% yield with Ni/C and Ru/C as catalyst, respectively (yields were based on Klason lignin content). During the acidic organosolv lignin extraction, the stabilization of the β-O-4 motif can also be achieved via α-alkoxylation by adding various alcohols or diols (Scheme 143, structure C),^651−653^ allowing for lignin extracted at mild conditions with high β-O-4 content. Barta et al.^654^ developed a ternary DES system (consisting of choline chloride, oxalic acid, and ethylene glycol) for stabilized and efficient lignocellulose fractionation. The resulting ethylene glycol incorporated lignin, characterized by its high β-O-4 content, underwent facile depolymerization under reductive conditions (employing Ru/C, 4 MPa H_2_, 220 °C in methanol for 18 h). This process yielded 24% monophenolic monomers with a selectivity of 49% toward 4-*n*-propylsyringol (Table 31, entry 5).

**Table 31:** Overview of Reductive Depolymerization of Isolated Lignin with a Modified Structure

Entry	Source and scale	Catalyst and ratio of cat./substrate	Solvent	Conditions	Yield	Major products	Ref
1	Beech and F5H-poplar lignin under formaldehyde stabilization; lignin solution 20 mL	5 wt% Ru/C; 100 mg cat.	THF	250 °C, H_2_ 4 MPa, 15 h	47% Phenolic monomers for beech lignin and 78% for F5H-poplar lignin	4-*n*-Propylsyringol and 4-*n*-propanolsyringol	(605)
2	Birch spruce and F5H-poplar lignin extracted with acetaldehyde (AA) and propionaldehyde (PPA) protection; lignin solution 20 mL	Pd/C; 250 mg Ru/C; 100 mg Ni/C; 100 mg	1,4-dioxane	200 °C, H_2_ 4 MPa, 20 h	48% Phenolic monomers for birch lignin, 70% for F5H-poplar lignin, and 20% from spruce lignin; the yields listed here are based on Pd/C	High selectivity (80%) to 4-*n*-propanolsyringol for poplar lignin	(648)
3	Endocarp lignin under propanal-assisted fractionation; 100 mg	Ru/C; 1:1	THF	250 °C, H_2_ 4 MPa, 3 h	Up to 120 g monomers/kg biomass	Ca. 75% selectivity to propylguaiacol/syringol	(649)
4	Propylidene acetal-stabilized lignin from birch, 2.5mg/mL in 1,4-dioxane/methanol mixture	Ni/C, Ru/C	1,4-dioxane/methanol (2:8, v/v)	180 °C, H_2_ 6 MPa, 20 h; flow reactor: lignin, feed: 0.1mL/min, lignin solution, flow rate of H_2_: 50 mL/min	45% Phenolic monomers over Ni/C and 40% phenolic monomers over Ru/C	Approximately 39% 4-*n*-propylsyringol over Ni/C and approximately 15–20% 4-*n*-propylsyringol over Ru/C	(650)
5	Ethyl glycol-incorporated lignin (α-alkoxylated lignin); 200 mg	Ru/C; 1:1	MeOH	220 °C, H_2_ 4 MPa, 18 h	24% Monophenolic monomers	49% Selectivity to 4-*n*-propylsyringol	(654)
6	Catechyl lignin from enzymatic hydrolysis (C-lignin) and C-lignin containing biomass; 200 mg	Pt/C, Pd/C, Ru/C; 0.5:1	MeOH, 1,4-dioxane, or THF	200 °C, H_2_ 4 MPa, 15 h	Up to 85 mol% catechyl monomers with isolated C-lignin	Approximately 60 mol% catechylpropane with Pd/C; 55% mol catechylpropanol with Ru/C	(655)
7	Isolated C-lignin by ChCl/LA; 50 mg	Pd/C, Ru/C; 0.3:1	MeOH	200 °C, H_2_ 3 MPa, 4 h	Catechyl monomers in 29.6 wt%	24 wt% Catechylpropanol with Pd/C	(656)
8	C-lignin sample from endocarp of castor seed coats; coats extracted by enzymatic and mild acidolysis treatment; 50 mg	Ru/ZnO/C; 0.3:1	MeOH	200 °C, H_2_ 3 MPa, 4 h	Catechyl monomers in 66 mol%	77% High selectivity to propenylcatechol	(657)

Despite the progress achieved in enhancing stabilization methods for lignin extraction to prevent condensation reactions,^646^ as well as the progress made in catalyst innovation,^138,499^ a diverse range of compounds continues to be produced through the process of reductive depolymerization. This gives rise to challenges in the subsequent separation steps. Ralph et al.^569,655^ reported the utilization of a noteworthy form of lignin, denoted as C-lignin. This specific lignin variant is formed almost solely by β-O-4 coupling of caffeyl alcohol. The growing polymer chain is characterized by a dominant benzodioxane homopolymer structure (Scheme 143, structure D), notably lacking higher condensed units and high acid-resistance. Therefore, the extraction of C-lignin can be accomplished while preserving a notable fraction of C-O linkages and mitigating undesired condensation reactions, which typically occurred during acid pretreatment or the extraction stage. This unique composition imparts to it the capacity for selective depolymerization into valuable catechyl-type monomers. The C-lignin is known to naturally occur in the seed coats of vanilla (*Vanilla planifolia*) and various plant species belonging to the *Melocactus* genus within the Cactaceae family.^569^ Hydrogenolysis of acidic LiBr pretreated C-lignin was tested with various combination of catalyst (i.e., Pt/C, Pd/C, and Ru/C) and solvent (i.e., MeOH, 1,4-dioxane, and THF). Pd/C and Ru/C in the presence of MeOH showed different selectivities yielding catechylpropane and catechylpropanol, as the primary products, in approximately 60 mol% and 55 mol%, respectively (Table 31, entry 6). Song et al.^656^ developed a protocol to extract C-lignin from castor seed coats by deep eutectic solvents (DESs) with high purity. Catalyzed by Pd/C in methanol under 3 MPa H_2_, a 29.6 wt% (equivalent to 81 mol%) yield of monomeric catechol derivatives was successfully achieved, demonstrating a notable 86% selectivity toward catechylpropanol (Table 31, entry 7). The same group investigated selective hydrogenolysis of C-lignin over an atomically dispersed ruthenium catalyst, 66% catechyl-type monomers were obtained with high 77% selectivity to unsaturated propenylcatechol (Table 31, entry 8).^657^ Román-Leshkov et al.^658^ applied reductive catalytic fractionation (RCF) to vanilla seeds to explore the depolymerization of C-lignin occurring naturally (see section 3.2.3.3). Given the beneficial attributes of C-lignin,^659^ genetic engineering presents itself as a viable strategy for augmenting the production of tailored lignins such as C-lignin within plants.^660,661^ The presence of α-OH and γ-OH in the lignin β-O-4 motif provides opportunities for β-O-4 activation and cleavage; however, this reactivity can also be problematic due to enhanced condensation and catalyst deactivation.^662^ Lignin structure simplification could be selectively achieved via dehydrogenative decarbonylation of the α-alkoxylated lignin to remove the γ-CH_2_OH in lignin β-O-4 motif.^613^ The resulting defunctionalized lignin without γ-OH might offer opportunities for selective conversion of lignins toward functionalized monomers, e.g., acetophenone derivatives under hydrogen-transfer conditions.^612^

**Scheme 144:**
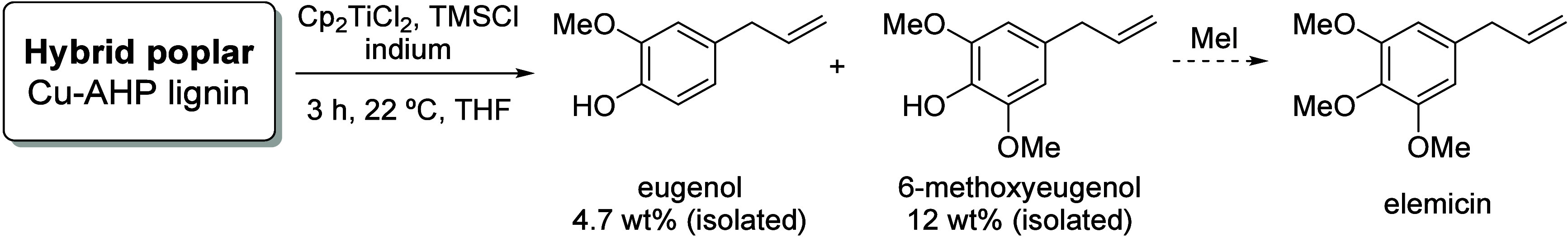
Depolymerization of Lignin via Microscopic Reverse Biosynthesis Combined with Reduction^663^

In addition to these, Diao et al. utilized microscopic reverse biosynthesis.^663^ The idea behind this is that lignin is depolymerized to the phenolpropanoid monomers from which it was originally formed. They reported depolymerization of high β-O-4 containing lignin, obtained by copper-catalyzed alkaline hydrogen peroxide (Cu-AHP)-treated hybrid poplar. This was done by treatment with Cp_2_TiCl_2_, TMSCl and Indium in THF for 3 h at room temperature (Scheme 144). Eugenol and 6-methoxyeugenol could be isolated as clear oils in 4.7 and 12 wt% yield, respectively. The latter could be converted quantitatively to elemicin, a flavours & fragrance (F&F) compound, after alkylation. Mechanistic investigations using model compounds showed that depolymerization occurs via β-scission of the benzylic radical intermediate after hydroxy abstraction and reduction. This was presented as a reverse reaction to the biosynthesis, forming monolignol radicals that subsequently lead to the formation of isolated allyl phenolics via subsequent dehydration and reduction steps.

##### 3.2.3.3 Reductive Catalytic Fractionation of Lignocellulose

**Figure 18:**
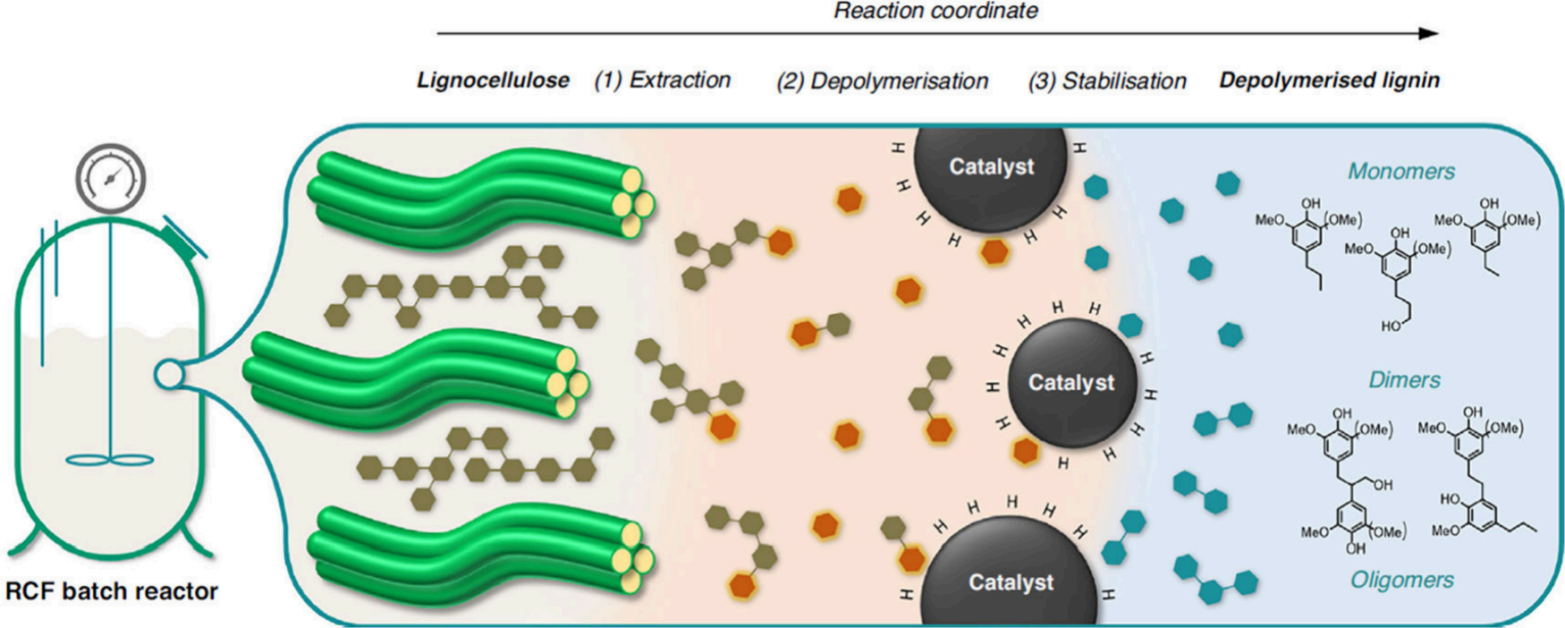
Schematic of RCF in batch mode, illustrating three key elementary steps: lignin extraction, depolymerization (via solvolytic or catalytic, like hydrogenolysis), and stabilization, yielding low-Mw lignin oil with monomers, dimers, oligomers, and carbohydrate pulp. Green hexagons: native lignin monomers. Orange: reactive units. Blue: stabilized units. Reproduced with permission from ref (664). Copyright 2018 Elsevier.

Reductive catalytic fractionation (RCF) represents an emerging domain for the selective conversion of lignin and is the main approach that falls under the lignin-first umbrella. In contrast to the prevailing prioritization of cellulose and hemicellulose utilization within lignocellulosic biomass, this approach aims to maximize lignin valorization by preventing lignin from being obtained as condensed C–C enriched structure. Reductive catalytic fractionation is centered on valorizing lignin as the first step, coupling three elementary steps, i.e., lignin extraction, depolymerization (solvolytic and catalytic), and stabilization of reactive intermediates (Figure 18).^664^

This approach thus can achieve the initial conversion of lignin in its “native” form, characterized by a higher proportion of C–O bonds, prior to the conversion or utilization of cellulose and hemicellulose. As such, a higher total monomer yield can be expected from the RCF approach as condensation and partial depolymerization in the lignin extraction step can be avoided. In a typical RCF reaction, solvolytic solvents such as short-chain alcohols (C1–C4) or cyclic ethers (e.g., 1,4-dixoane) are typically employed to facilitate the lignin extraction with or without water addition, resembling the organosolv lignin extraction.^665^ Simultaneously, redox catalysts usually containing supported metal catalysts (e.g., Ni, Ru, and Pd) are applied to facilitate the depolymerization and stabilization step. It is worthy to note that lignin is not merely catalytically depolymerized under RCF conditions, solvolytic depolymerization of lignin via homolytic cleavage of β-O-4 can also play an important role depending on the process conditions (e.g., reaction temperature and solvent applied, as well as pH).^664^ The reductive conditions are predominantly induced by the introduction of pressurized hydrogen gas. Alternatively, these reductive conditions can be generated using hydrogen-donating solvents or even from constituents within the biomass itself, such as hemicellulose.^666^ RCF products include a low molecular weight lignin oil consisting of various monophenolics (such as 4-*n*-propylguaiacol/syringol, 4-*n*-propanolguaiacol/syringol and 4-*n*-propenylguaiacol/syringol), oligomeric fragments and carbohydrate-derived compounds leaving a residual solid carbohydrate-rich pulp. The historical inception of reductive catalytic fractionation, aimed at structurally analyzing lignin, dates back to the 1930s and 1940s.^623,667^ In conjunction with the alkaline oxidative depolymerization and alcoholysis applied to lignocellulosic biomass, these investigations empirically validated the intricate aromatic nature of lignin. This validation substantiated the foundational insights initially postulated by Klason, Fredenberg, and Hibbert, ultimately laying the cornerstone for contemporary lignin chemistry.^668^ Over the past decade, substantial progress has been made in rapidly advancing this approach toward the production of phenolic and aromatic monomers, low molecular weight lignin oils and carbohydrate pulp, which can be further valorized, respectively.^665,668−671^ In the following part, studies toward high monomer yields are highlighted.

In 2008, Kou et al.^672^ employed a range of noble metals (Pd, Pt, Ru, and Rh) supported by carbon to degrade birch sawdust (Table 32, entry 1). The emphasis was on converting lignin to monomers and dimers, which were followed by subsequent conversion into alkanes and methanol by hydrodeoxygenation and hydrogenation. The highest total monomers yield (including 4-*n*-propylguiacol/syringol and 4-*n*-propanolguaiacol/syringol) was 46% with Pt/C as catalyst. The prospect of producing bio-ethylene glycol from cellulose holds substantial promise.^673^ Nonetheless, its feasibility hinges upon the development of energy-efficient and cost-effective pretreatment methods to access cellulose as a substrate. In response to this challenge, in 2012, Zhang et al.^674^ documented a one-pot catalytic hydrocracking process using Ni-W_2_C/AC as a catalyst, enabling the direct conversion of raw woody biomass into monophenols and aliphatic diols (Table 32, entry 3). This method achieves simultaneous conversion of cellulose, hemicellulose, and lignin, effectively bypassing the need for pretreatment. This investigation yielded an intriguing observation: the substitution of H_2_O with methanol or ethylene glycol as solvents impedes the transformation of cellulose and hemicellulose into diols. Concurrently, the proportion of monophenols derived from lignin rises from 36.9% to 42.2% and 46.5%, respectively. This phenomenon underscores the substantial potential to customize the catalytic process for distinct objectives through straightforward solvent modifications. Xu and Wang et al.^675^ demonstrated that Ni/C can chemoselectively convert lignin in birch wood to 4-*n*-propylguaiacol/syringol in 90% selectivity (Table 32, entry 4). With methanol as hydrogen donor, this process achieved 50% conversion of lignin. A stepwise mechanism was postulated, comprising initial alcoholysis-based extraction and fragmentation, followed by subsequent depolymerization. Analysis with MALDI-TOF and NMR demonstrated that the methanol facilitated the extraction and fragmentation of lignin with a molecular weight of *m/z* ca. 1100 to 1600, while the isotope studies confirmed that methanol was involved in providing hydrogen for C–O bond cleavage. Rinaldi et al.^676^ proposed a biorefining strategy using Raney Ni-catalyzed lignin depolymerization to convert plant biomass. This approach yields lignin bio-oil (in which 60% of its components have a molecular weight < 350 Da), suitable for subsequent hydroprocessing to produce chemicals and fuels, along with a pulp that is amenable to downstream enzymatic hydrolysis (as shown in Table 32, entry 5). This is one of the first examples where the name Early-stage Catalytic Conversion of Lignin (ECCL) was coined; which is another name for the lignin-first concept as used here.^677,678^

**Table 32:** Overview of the Reductive Catalytic Fractionation of Biomass to Alkyl Phenolics

Entry	Source and scale	Catalyst and cat.:substrate ratio	Setup	Solvent	Conditions	Total monomer yield	Major products	Carbohydrate retention	Year and ref
1	Birch; 4 g	Pt/C, Pd/C, Ru/C, Rh/C and 1% H_3_PO_4_; 0.075:1	Batch reactor	1,4-dioxane/H_2_O (1:1, v/v)	200 °C, H_2_ 4 MPa, 4 h	Up to 46.4%	4-*n*-Propanol-guaiacol/syringol	Not reported	2008 (672)
2	Pine, 2.2 g	Pd/C; 0.08:1	Batch reactor	1,4-dioxane/water (1:1 v/v)	195 °C, H_2_ 3.45 MPa, 24 h	22.0%	4-*n*-Propyl-guaiacol and 4-*n*-propanolguaiacol	Not reported	2011^713^
3	Birch, corn stalk, pine, poplar, beech, etc.; 1 g	Ni-W_2_C/AC Na_2_SO_4_ and NaCl could be added; 0.4:1	Batch reactor	H_2_O	235 °C, H_2_ 6 MPa, 4 h	Up to 46.5%	4-*n*-Propyl-guaiacol/syringol	Converted to diols; total yield up to 76.1% and mainly ethylene glycol	2012 (674)
4	Birch; 2 g	Ni/C; 0.05:1	Batch reactor	MeOH	200 °C, Ar 0.1 MPa, 6 h	48.6%	4-*n*-Propyl-guaiacol/syringol	Not reported	2013 (675)
5	Poplar; 16 g	Raney Ni; 0.625:1	Batch reactor	2-PrOH/H_2_O (7:3, v/v)	180 °C, Ar 0.1 MPa, 3 h	25% bio-oil based on initial weight of biomass	Mostly alkylphenolics as shown by 2D-GC	71%	2014 (676)
6	Birch; 10 g	Pd/C; 0.13:1	Batch reactor	Ethanol/H_2_O (1:1, v/v)	195 °C, Ar 0.4 MPa, 1 h	49% isolated yield	2,6-Dimethoxy-4-(prop-1-enyl)-phenol	Not reported	2014 (679)
7	Poplar; 1 g	ZnPd (1:0.1)/C	Batch reactor	MeOH	220 °C, H_2_ 3.4 MPa, 12 h	Up to 54%	4-*n*-prolyl-guaiacol/syringol	74%	2014 (681)
8	Birch; 2 g	Ru/C; 0.15:1	Batch reactor	MeOH	250 °C, H_2_ 3 MPa, 3–6 h	Up to 52% monophenols	4-*n*-prolyl-guaiacol/syringol in 41%	92%	2015 (683)
9	Pine, corn stalk, corncob, wheat straw, rice straw; 1 g	Ru/C + LiTaMo_6_; 0.2:0.2:1	Batch reactor	Aqueous H_3_PO_4_ solution (0.1–0.3 M)	230 °C, H_2_ 6 MPa, 24 h	Up to 35.4% phenols	Mostly alkylphenolics	Converted to gasoline alkanes with the yield up to 82.4%	2015 (714)
10	Birch; 2 g	Ru/C and Pd/C; 0.2:1	Batch reactor	MeOH	250 °C, H_2_ 3 MPa, 3 h	48% and 49% for Ru/C and Pd/C, respectively	Selectivity change from 4-*n*-prolyl-guaiacol/syringol (75%) to 4-*n*-propanol-guaiacol/syringol (91%)	85% and 89% for Ru/C and Pd/C, respectively	2015 (715)
11	Miscanthus; 1 g	Ni/C; 0.15:1	Batch reactor	MeOH	225 °C, H_2_ 3.5 MPa, 12 h	68%	Methyl dihydroferulate and methyl *p*-hydroxyhydrocinnamate in 28%; 4-*n*-prolylguaiacol/syringol in 40%	88.5%; further converted to furfural and levulinic acid	2016 (682)
12	Corn stover; 1 g	Ru/C, Ni/C; 0.1:1	Batch reactor	MeOH	200–250 °C, H_2_ 3 MPa, 1–6 h	Up to 38% monophenolic monomers	65% selectivity to methyl coumarate/ferulate	>90%	2016 (716)
13	Poplar; 2 g	Pd/C; 0.1:1	Batch reactor	MeOH with H_3_PO4 or NaOH at 1.25–5 g/L	200 °C, H_2_ 2 MPa, 3 h	Up to 42% monophenols	35% 4-*n*-Propanolsyringol	Approximately 65–90%	2016 (685)
14	Poplar; 2 g	Pd/C; 0.1:1	Batch reactor	MeOH or EtOH with varying water content from 0 to 100%	200 °C, H_2_ 2 MPa, 3 h	Up to 45% monophenols	4-*n*-Propanol-guaicol/syringol as major products	Up to 98%	2016 (686)
15	Birch, poplar, spruce, pine; 10 g	Pd/C; 0.1:1	Batch reactor	Ethanol–water (1:1, v/v)	210 °C, 15 h	Up to 36% monophenolics	4-*n*-Propyl/propenyl-syringol as major products	Up to 64%	2016 (666)
16	Beech; 1 g	Ni/C; 0.1:1	Batch reactor	MeOH–water (6:4, v/v)	200 °C, H_2_ 2 MPa, 5 h	Up to 51.4%	75.3% selectivity to 4-*n*-propanol-guaiacol/syringol	Not reported	2016 (717)
17	Birch, oak, Douglas fir; 2 g	Pd/C+Al(OTf)_3_; 0.1:1HCl; H_2_SO_4_, H_3_PO_4_, CH_3_COOH; *p*-TsOH was also tested as co-catalyst	Batch reactor	MeOH	180 °C, H_2_ 3 MPa, 2 h	Up to 48% monophenolics	Mixture of alkylmethoxyphenols	Up to 96%	2016, 2017, 2018^697−700^
18	Birch; 2 g	Ni/Al_2_O_3_ in basket; 0.1:1	Batch reactor	MeOH	250 °C, H_2_ 3 MPa, 3 h	More than 40% monophenols	70% selectivity to 4-*n*-propanol-guaiacol/syringol	93% glucose and 83% xylose retention	2017 (688)
19	Eucalyptus; 2 g	Ru/C; 0.1:1	Batch reactor	*n*-Butanol/water (1:1, v/v)	200 °C, H_2_ 3 MPa, 3 h	Up to 50% monophenols	4-*n*-Propanol-guaiacol/syringol as major products	Hemicellulose to polyols with yield of 49%; cellulose 96% yields as pulp	2018 (689)
20	Pine, walnut, poplar, oak, beech, etc.; 1 g	Cu20-PMO; 0.2:1	Batch reactor	MeOH	180 °C, H_2_ 4 MPa, 18 h	Up to 36%	4-*n*-Propanolguaiacol/syringol were isolated at yield of with selectivity of 67%	to aliphatic alcohols at elevated conditions (320 °C, 6 h)	2018 (703)
21	Birch; 0.2 g	Cobalt on phenanthroline/carbon catalyst (Co-phen/C); 0.15:1	Batch reactor	EtOH/H_2_O (1:1, v/v) and HCOOH + HCOONa as hydrogen donor	200 °C, 4 h	34%	56% selectivity to 4-*n*-propyl/propenyl-syringol	32%	2018 (680)
22	Apple wood, pine; 0.5 g	MoxC/CNTs for hardwood; Ru/CMK-3 for softwood and grass	Batch reactor	MeOH	250 °C, H_2_ 1 MPa, 3 h	Up to 42% monophenolics for hard wood, 20% for soft wood	Mostly alkylphenolics	For Mo-based cat. C5 and C6, retention of 89 and 98%, respectively; for Ru-based cat., 52.9%	2018 (718)
23	Birch; 2 g	Pt/γ-Al_2_O_3_; 0.25:1	Batch reactor	MeOH/water (1:2, v:v)	230 °C, N_2_ 3 MPa, 3 h	49.5% monophenolics	46.1% Propylguaiacol/syringol	41%	2019 (719)
24	Beech; 1 g	Pd_70_Pt_30_ NPs	Batch reactor	MeOH	250 °C, H_2_ 3 MPa, 3 h	45%	Sum of 4-*n*-propyl-guaiacol/syringol and 4-*n*-propanol-guaiacol/syringol	Not reported	2019 (720)
25	Pine; 1 g	α-HfP NPs; 0.2:1	Batch reactor	H_2_O	190 °C, H_2_ 3.5 MPa, 3 h	19.8%	18.5% 4-*n*-Propylguaiacol	Not reported	2019 (504)
26	Eucalyptus; 0.5 g	Ni@ZIF-8; 0.1:1	Batch reactor	MeOH	260 °C, H_2_ 3 MPa, 8 h	44.3%	55% selectivity to 4-*n*-propyl-guaiacol/syringol	Not reported	2019 (721)
27	Eucalyptus; 1 g	Pd/C; 0.1:1	Batch reactor	MeOH	240 °C, H_2_ 3 MPa, 4 h	49.8 wt%	4-*n*-Propanolguaiacol 12.9% and 4-*n*-propanolsyringol 31.9%	Retention C5 of 67.8% and C6 of 82.5%; to furfural catalyzed by FeCl_3_ in another step	2020 (722)
28	Birch; 2 g	Ni_50_Pd_50_/SBA-15; 0.075:1	Batch reactor	*i*-PrOH:H_2_O (2:1, v/v)	245 °C, N_2_ 1 MPa, 4 h	37.2%	18.9% 4-*n*-Propylsyringol	Not reported	2020 (723)
29	Hemp hurd; 0.2 g	Pd/C; 0.1:1	Batch reactor	MeOH/H_2_O (7:3, v:v)	200 °C, 4 h	38%	High selectivity to 4-*n*-propyl/propanolsyringol	Approximately 43%	2021 (724)
30	Corn stover; 1 g	Co/AC-N; 0.1:1	Batch reactor	*i*-PrOH/water/ formic acid (10:1:1, v:v:v)	235 °C, N_2_ 0.1 MPa, 200 min	24%	High selectivity to 4-*n*-ehtylguaiacol and 4-*n*-ethylphenol	8%	2021^725^
31	Triploid poplar; 0.5 g	Ru/C, Cs_2_CO_3_ (4 wt%); 0.1:1	Batch reactor	MeOH	220 °C, H_2_ 3 MPa, 4 h	30.2%	High selectivity to propyl/propanol guaiacol/syringol	Retention of C5 53% and C6 80%	2022 (726)
32	Pine; 10 g	CuO/C; 0.1:1	Batch reactor	MeOH	240 °C, H_2_ 3 MPa, 4 h	12.1% isolated yield	Isolated 4.8% for propylguaiacol and 7.5% for propanolguaiacol	Not reported	2022 (727)
33	Various types of hardwood, softwood, and herbaceous planta; 0.5 g	Ru/CNT; 0.1:1	Batch reactor	MeOH	220 °C, H_2_ 3 MPa, 4 h	Up to 46% in general hardwood > herbaceous plant > softwood	For hard wood, high selectivity to propanol/propylsyringol/guaiacol	Not reported	2022 (728)
34	Poplar; 1 g	Ru/C, Pd/C, Pt/C, Ni/C; 0.1:1	Batch reactor	Ethylene glycol, 1,2-propanediol, 2,3-butanediol, and glycerol	225 °C, 3 h, flushed with He before reaction	Up to 21%	High selectivity to propyl/propanol guaiacol/syringol	Not reported	2023 (695)
35	Poplar; 1 g	0.3 g Ni/C catalyst mixed with SiO_2_ (50/50) dispersed inSiC	Flow reactor	Methanol with flow rate of 0.5 mL/min	190 °C for both beds, 6 MPa H_2_ with a flow rate of 50 mL/min, 3 h	Up to 18%	High selectivity to propylguaiacol/syringol	Not reported	2017 (706)
36	Birch; 0.15 g	Pd/C; 0.15 g	Flow reactor	2.8 g L^–1^ H_3_PO_4_ in MeOH–H_2_O 7:3 v/v; flow rate 0.3 mL/min	Solvolysis bed = 200 °C; catalytic bed = 180 °C, BPR 3 MPa H_2_, 3 h	37%	71% selectivity toward 4-*n*-propylguaiacol/syringol and their corresponding methyl ether derivatives	92%	2017 (711)
37	Birch; 1 g	β-zeolite; 2 g	Flow reactor	Ethanol/H_2_O (9:1, v/v); flow rate 0.5 mL/min	220 °C, 5 MPa, 3 h	16%	Ca. 60% selectivity to unsaturated aromatics, namely eugenol and isoeugenol	Not reported	2021 (708)
38	Poplar; 300 mg	Ru/C; 0.15:1	Batch reactor	2-Butanol 5 mL and 25 wt% aq. NH_3_ 1 mL	255 °C, H_2_ 3 MPa H_2_, 3 h	65.6% monophenolics	26.6% 4-[3-(2-Butyl)amino]propyl]phenol	C6 98%, C5 86%	2023 (712)

**Figure 19:**
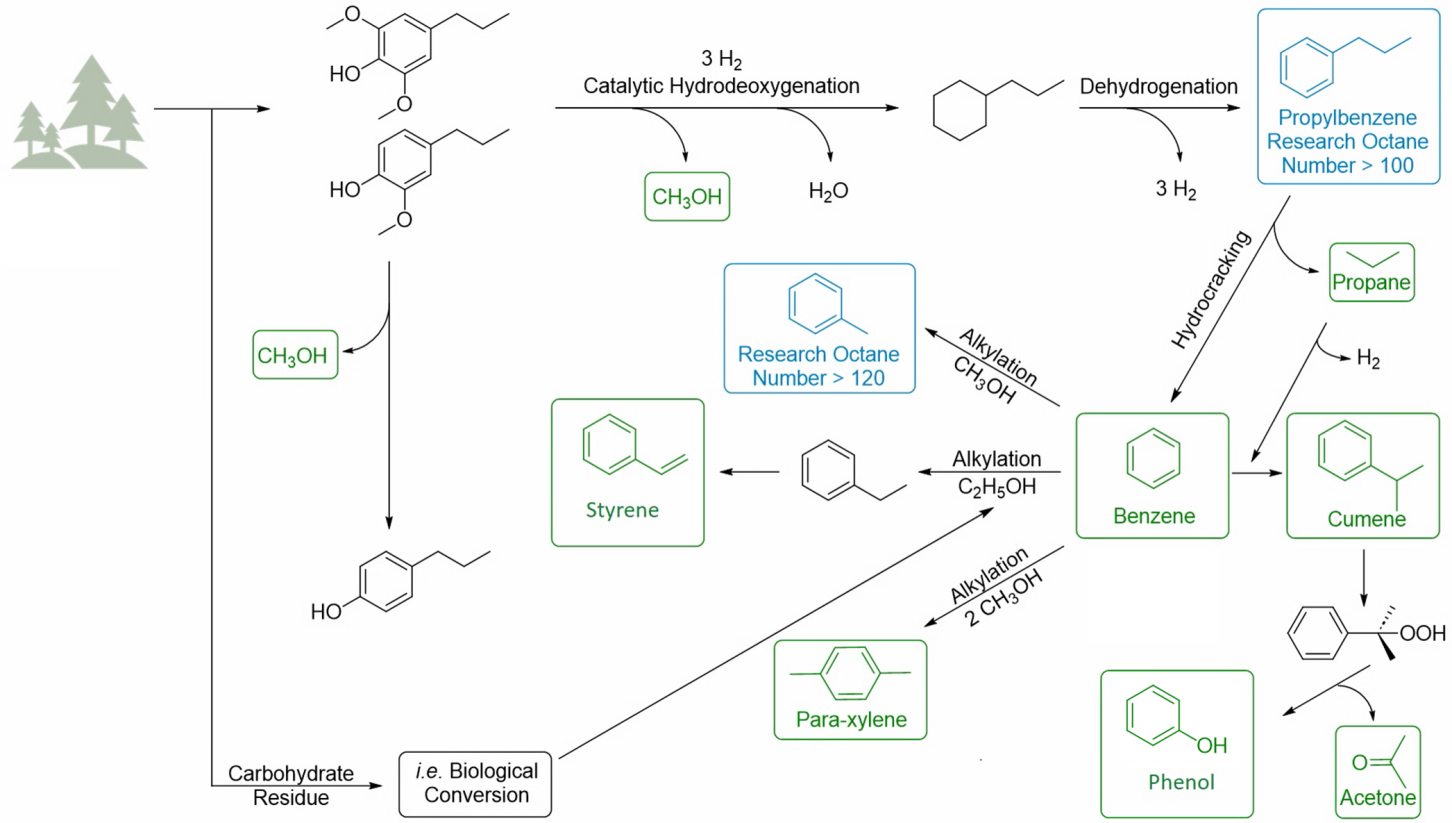
Pathways for renewable fuel (blue) and chemicals (green) production from lignin. Methoxypropylphenols can be used directly in the fragrance industry (as dihydroeugenol) or modified catalytically for fuels (e.g., propylbenzene, toluene) or chemicals (e.g., propane, methanol, benzene, cumene, *p*-xylene, ethylbenzene, styrene, phenol, acetone). Reproduced with permission from ref (681). Copyright 2015 Royal Society of Chemistry.

**Scheme 145:**
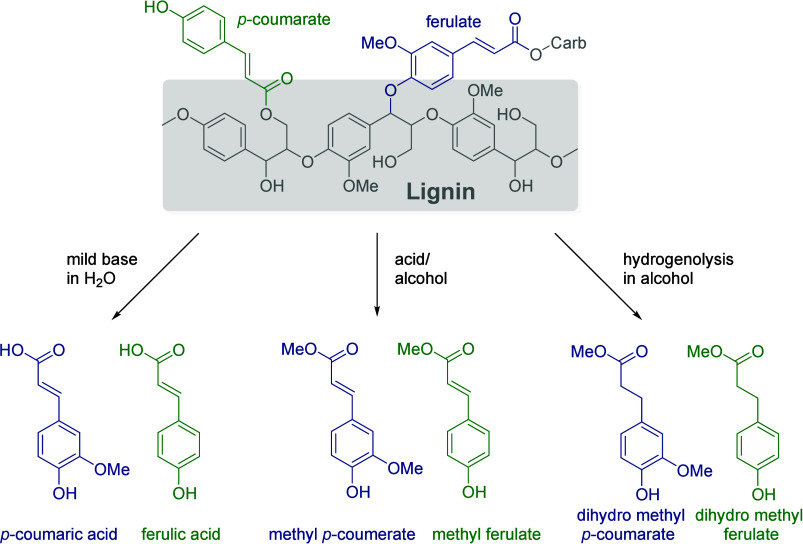
Strategies for the Recovery of pHCAs from Lignin

Samec et al.^679^ reported an approach involving one-pot fragmentation-depolymerization to convert wood into unsaturated 4-*n*-propenylguaiacol/syringol. This process hinged upon an initial step of organosolv pulping in an ethanol/water medium, followed by a Pd-catalyzed transfer hydrogenolysis. The latter process was claimed to utilize H_2_ liberated through the decomposition of hydrogen donors such as formic acid and polyols known to be released during the organosolv fractionation process. In addition, the dehydrogenation of α-OH within the lignin β-O-4 motifs could also contribute H_2_. Application of this methodology to birch wood yielded 49% isolated 4-*n*-propenylsyringol (Table 32, entry 6). The same group showed that hydrogen necessary for the RCF process can be supplied by the hemicellulose fraction,^666^ and the noble metal catalyst can be substituted with cobalt on a phenanthroline/carbon support (Table 32, entries 15 and 21).^680^ Abu-Omar et al.^681^ demonstrated a synergistic biorefinery based on bimetallic Zn/Pd/C-catalyzed lignin conversion prior to cellulose starting from lignocellulosic biomass. Starting from poplar, 54% monomers containing 4-*n*-propylguaiacol and 4-*n*-propylsyringol were obtained with a selectivity of 45% and 55%, respectively (Table 32, entry 7). In this study, lignin-derived methoxyphenols were further demonstrated to be converted to, e.g., propylbenzene as a valuable platform chemical (Figure 19). The same group further reported a method for total utilization of miscanthus biomass upon conversion with Ni/C under hydrogen atmosphere, in which the carbohydrate residue was converted to furfural and levulinic acid catalyzed by iron(III) chloride in yields of 55% and 76%, respectively (Table 32, entry 11).^682^ In addition to 4-*n*-propylguaiacol/syringol, the obtained monomers included methyl ferulate ester and its derivatives (for detailed structures see Scheme 145 in section 3.3). These products were generated from bound ferulate groups commonly found in grasses.

**Figure 20:**
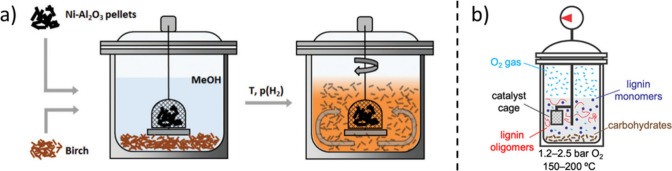
Catalyst baskets used for (a) RCF and (b) OCF process facilitating catalyst–product separation and recovery. Reproduced with permission from ref (688), copyright 2017 Royal Society of Chemistry, and ref (598), copyright 2021 American Chemical Society.

In 2015, Sels et al.^683^ presented a study describing a methodology employing Ru/C as a catalyst within methanol under a hydrogen pressure of 3 MPa. This process effectively transformed lignin into predominantly monomeric compounds with >50% yield, notably 4-*n*-propylguaiacol and 4-*n*-propylsyringol, but also dimers and oligomers (Table 32, entry 8). Concurrently, it yielded carbohydrate pulp at a theoretical yield, making it well-suited for subsequent applications. Within the context of this investigation, the terminology “lignin-first” strategy was introduced for the first time to describe this approach, which was further developed and refined as described at the start of this section.^684^ The same group also conducted additional investigations to assess the impact of various process parameters on the efficiency of the lignin-first process, including the effects of introducing acid (H_3_PO_4_), alkaline (NaOH) additives,^685^ the incorporation of water in reaction media,^686^ and solvent (Table 32, entry 13&14).^687^ The primary role of the metal catalyst in the RCF process is to depolymerize and largely stabilize the reactive intermediate produced during the solvolysis step. The same group reported an approach whereby catalyst in pellet form was strategically positioned within a basket within the reactor, simplifying the subsequent separation process (Figure 20a, catalyst basket was reported to be used in the OCF process as well shown in Figure 20b).^688^ Furthermore, by changing the catalyst from Ru/C to Pd/C, it was possible to maintain the hydroxy group on the propyl unit of monophenolics. Altering the solvent from methanol to *n*-butanol/H_2_O enables the conversion of hemicellulose into polyols, ultimately yielding a pure cellulose pulp (Table 32, entry 19).^689^ The lignin oil derived from the RCF process comprises not only monomers but also includes dimers, trimers, and oligomers, collectively constituting approximately 50% of the total lignin content.^690,691^ The isolation of monomers from this complex lignin oil poses a considerable challenge. Addressing this, Sels et al.^692^ employed silicon-based membranes for nanofiltration within the RCF process. By employing a two-stage filtration approach, they achieved a separation factor of 25.4 and obtained enrichment in monomers (mixture of alkyl phenols) up to a purity of 95% in the permeate. Notwithstanding the challenges associated with the separation, it is important to note that the operational pressure, stemming from the external hydrogen utilization, and the vapor pressure of methanol, pose significant challenges to the capital cost of the reactor.^693^

**Figure 21:**
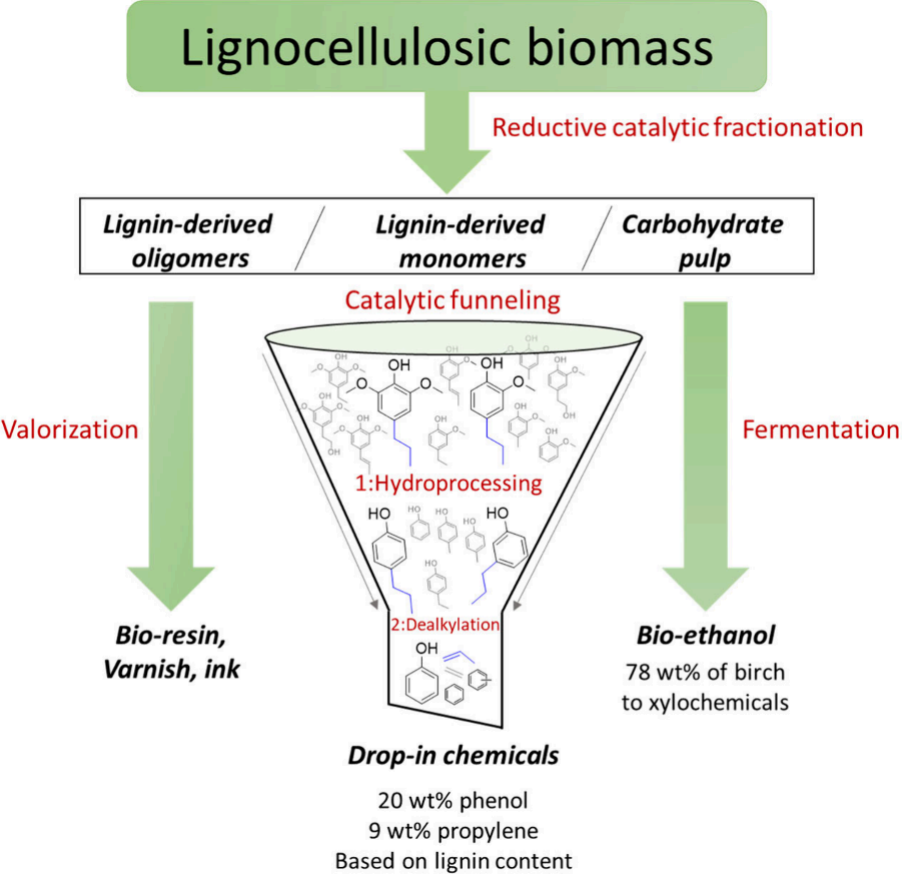
RCF oriented wood refinery proposed by Sels et al.^694^

Based on the concept of lignin first, Sels et al.^694^ proposed a sustainable and economically-viable wood conversion biorefinery (Figure 21). The obtained RCF lignin alkylphenolic monomers were transformed to phenol and propylene by catalytic funneling, while bio-ink can be obtained from phenolic oligomers. The carbohydrate pulp could be used for bioethanol production.

Overall, high carbon and mass efficiency can be achieved, and 78 wt% of wood (in this case birch) was converted to valuable xylochemcials. In a recent investigation conducted by Beckham and Román-Leshkov et al., a hydrogen-free RCF process was showcased by employing low vapor pressure hydrogen-donating solvents, namely ethylene glycol, 1,2-propanediol, 2,3-butanediol, and glycerol. Comparable monophenolic yields (17–19%) were attained using 2,3-butanediol under various catalysts (including Ru/C, Pd/C, Pt/C) as to the utilization of pressurized external H_2_ (Table 32, entry 34).^695^ Similarly, He et al.^696^ demonstrated a selective RCF process at atmospheric pressure without hydrogen, in which acidified ethylene glycol (0.2–0.8 g L^–1^ sulfuric acid) was used as solvent without using high-pressure reactors. At a temperature range of 185–195 °C, this hydrogen-free process yielded 24.1% monomers, whereas the typical RCF process achieved 31.6% monomer production at a higher temperature range of 220–250 °C. Hensen et al. conducted a series of investigations wherein they employed homogeneous acids (i.e., metal triflates, HCl, H_2_SO_4_, H_3_PO_4_, CH_3_COOH, *p*-TsOH) as a co-catalyst to enhance both the solvolysis step and the subsequent lignin depolymerization step within the context of the RCF concept (Table 32, entry 17).^697−700^ The addition of acids is well-established for enhancing the efficacy of organosolv delignification, as well as facilitating C–O bond cleavage. When trace amounts of Al(OTf)_3_ were introduced during the RCF process, it promoted the disruption of lignin-carbohydrate interlinkages, thereby expediting the release of the lignin fraction during the solvolysis phase and aiding in the removal of hemicellulose, ultimately yielding pure cellulose pulp. Consequently, this approach can significantly reduce both reaction time and temperature requirements, achieving nearly 50% monophenolics within a 2 h reaction period at 180 °C.^697^ Barta and co-workers^625,701,702^ pioneered the Cu-PMO-catalyzed reductive catalytic fractionation. In 2018, they demonstrated a two-step LignoFlex process catalyzed by the same noble-metal-free catalyst, Cu-PMO, which achieved complete lignocellulose conversion (Table 32, entry 20).^703^ The first step selectively converted lignin to mainly 4-*n*-propanolguaicol, while the second step generated aliphatic alcohols by catalytically converting the remaining carbohydrate pulp. Both products from the two steps can be further upgraded to high-value chemicals by divergent or convergent pathways. For example, the isolated 4-*n*-propanol-guaiacol/syringol could be transformed to amines, while the aliphatic alcohols can be tuned to fuels via chain elongation and hydrodeoxygenation. Under reductive conditions, employing a supported noble metal catalyst (e.g., Ru/C), the typical product profile primarily comprises 4-*n*-propyl-substituted phenols. This outcome is attributed to the high reactivity of these catalysts in reducing C=C bonds and deoxygenating alcohols. Interestingly, Song et al.^704^ reported that MoO_*x*_/SBA-15 exhibited the ability to depolymerize lignin, yielding 43.4% monophenolic compounds with an impressive 86% selectivity toward side chain unsaturated monolignols and their derivatives. This distinctive outcome arises from the catalyst’s pronounced capability to cleave C–O bonds while displaying limited hydrogenation activity. In the RCF process, the typical outcome involves the preservation of hemicellulose and cellulose within the pulp, or the non-selective conversion of hemicellulose and cellulose pulp. To optimize the utilization of lignocellulosic biomass, a method involving the pretreatment of biomass with FeCl_3_ as a catalyst has been documented.^705^ This pretreatment selectively converts hemicellulose into pentose sugars, allowing the remaining cellulose-lignin pulp to subsequently undergo the RCF process for the selective production of monophenolics from both lignin and cellulose pulp. However, it is worth noting that this pretreatment step may potentially lead to lignin condensation or partial cleavage of C–O bonds. Given the extensive body of research in this domain, we have compiled further specific examples with high reported monomer yields, which are outlined in Table 32, entries 21–34.

**Figure 22:**
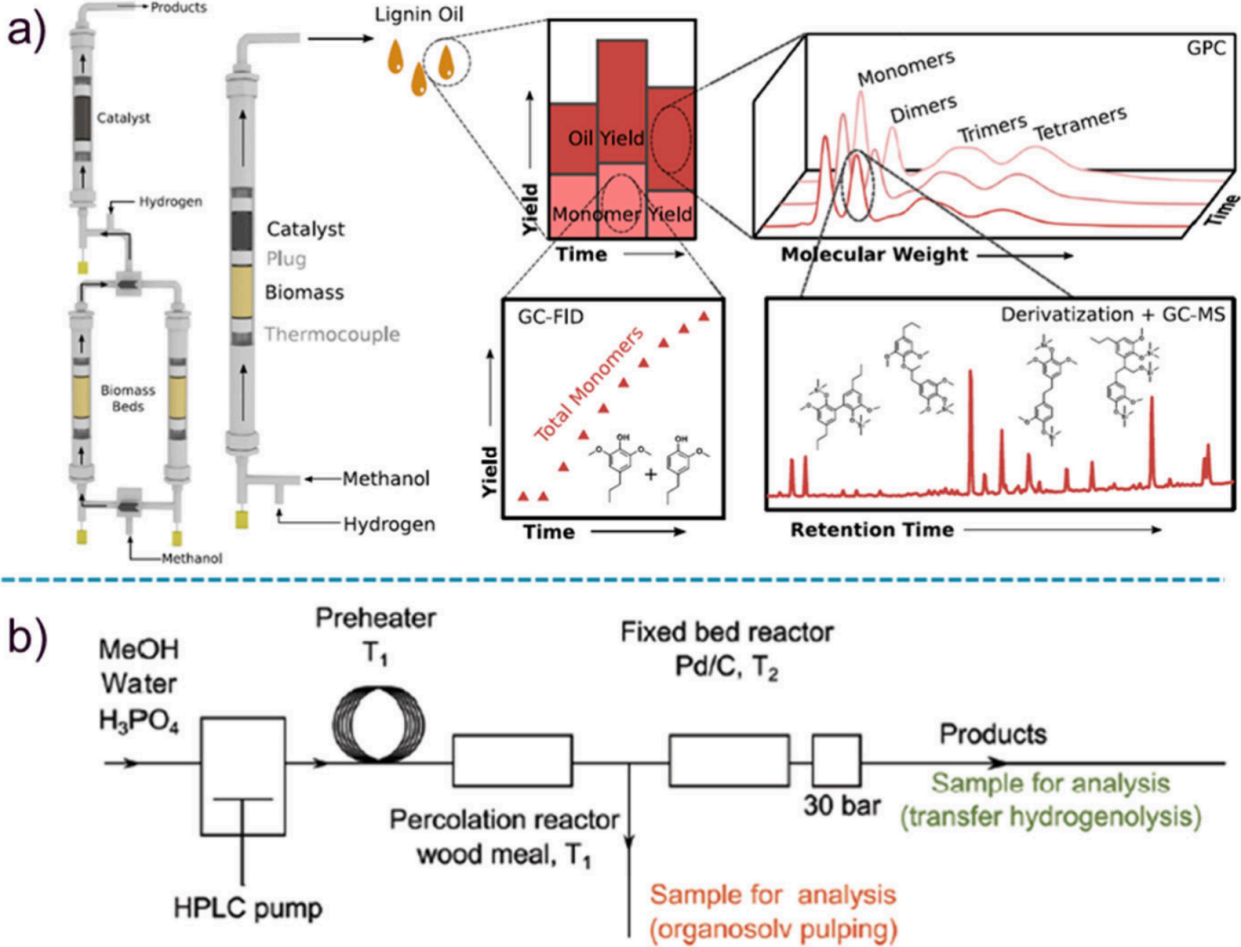
(a) Illustration of the flow-through single-bed reactor for RCF from the Román-Leshkov group, in combination with the different analysis techniques for characterizing lignin oil at different extraction times. (b) Schematic representation of the flow-through system used for RCF in the Samec group. Reproduced with permission from ref (706), copyright 2017 Elsevier, and ref (711), copyright 2017 Royal Society of Chemistry.

In terms of reactor configuration, transitioning from a batch reactor to a semiflow reactor and ultimately to a continuous-flow reactor represents a favorable strategy for scaling up the process.^706^ For instance, a flow reactor offers a valuable advantage by enabling the segregation of the solvolysis step from the depolymerization and stabilization step, which tend to be intertwined in batch RCF studies. Consequently, it presents significant opportunities for conducting independent investigations of the solvolysis and catalytic stages, such as kinetic studies,^707^ catalyst deactivation studies,^708^ and intermediates separation for mechanistic understanding, as well as exploring relationships between lignin structure and performance relationships (Table 32, entries 35–38).^709^ In addition, it provides an efficient way of extracting “native-like” lignin for mechanistic or material studies by solely applying the decoupled flow through solvolysis step.^710^ Román-Leshkov and Beckham et al. demonstrated a flowthrough RCF concept (Figure 22a and Table 32, entry 35), enabling the identification of time-resolved lignin intermediates involved with either the solvolysis or the hydrogenolysis steps, serving as a great tool for understanding RCF fundamentals.^706^ Samec et al. demonstrated a flow-through system tailored for conducting RCF investigations, notable for its capacity to independently adjust reaction parameters for both the solvolysis and reduction stages (Figure 22b and Table 32, entry 36).^711^ When utilizing birch in this flow-through reactor, they obtained a 37% yield of monophenolic compounds, with an impressive 71% selectivity toward 4-*n*-propylguaiacol/syringol and their corresponding methyl ether derivatives.

**Figure 23:**
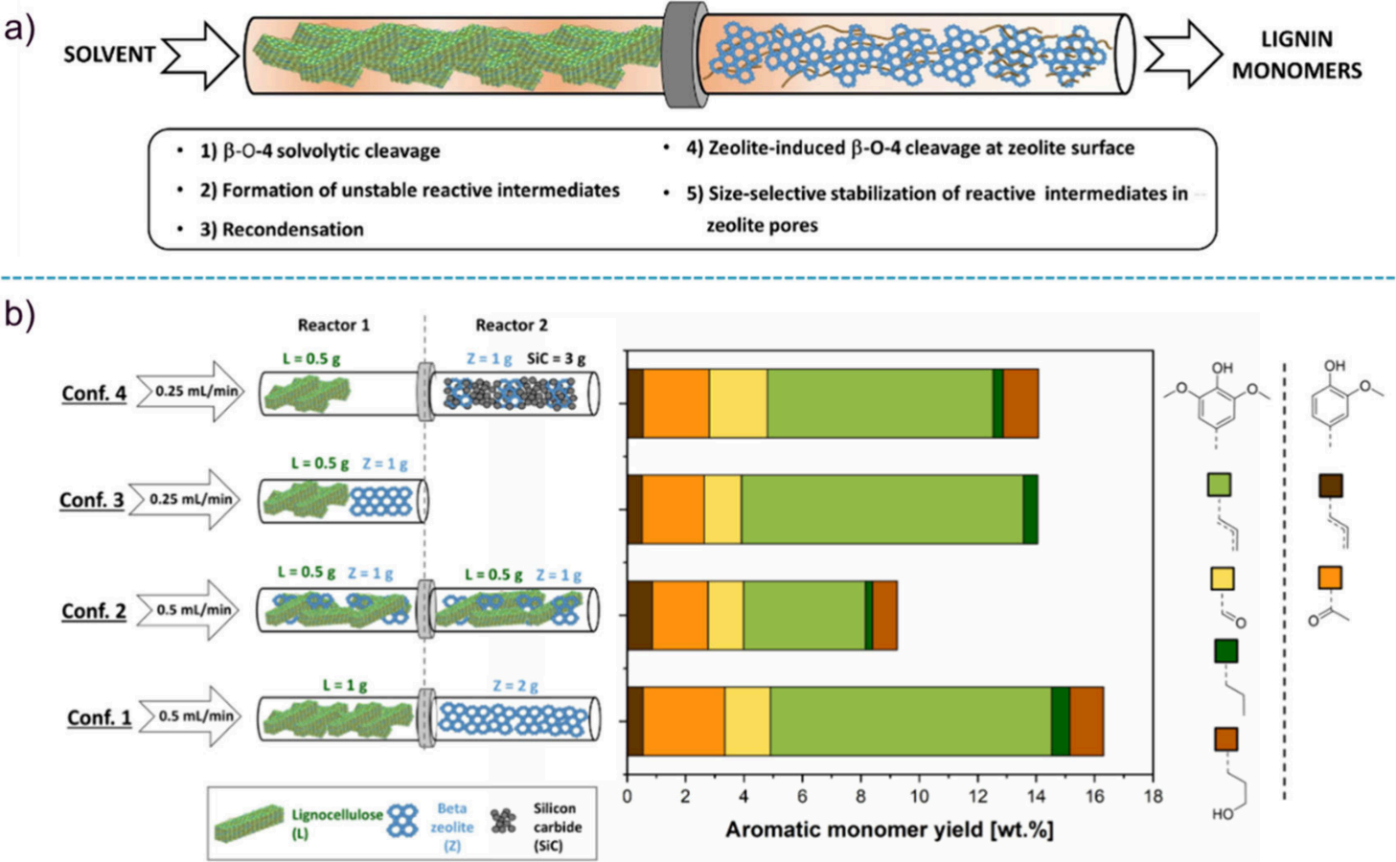
(a) Illustration of the flow-through β-zeolite-assisted lignocellulose fractionation. (b) Effect of different reactor configurations in the aromatic monomer yield. Reproduced with permission from ref (708). Copyright 2021 John Wiley & Sons, Inc.

Instead of using a redox catalyst for the reduction/stabilization step for the lignin-first process, D’Angelo et al. reported a flow-through reactor applying a β-zeolite to stabilize the reactive intermediate released from the solvolysis step under hydrogen-free conditions.^708^ The acidity of the β-zeolite helps to cleave the C–O bonds, meanwhile it can also prevent intermediate condensation due to its size-selective properties. Different configurations of the flow reactor (e.g., a separated bed for biomass and catalyst, mixing catalyst with biomass for a single bed, diluted catalytic bed, etc.) were tested for their influence on monomers yield (Figure 23). Approximately 16% monophenolics were obtained from birch under reactor configuration 1 (Figure 23 and Table 32, entry 37).

Incorporating heteroatoms, such as nitrogen, into the monomeric compounds derived from lignin depolymerization holds significant promise for enhancing the value of these monomers, which currently have limited market applications. Rather than following the conventional two-step approach involving depolymerization and subsequent functionalization to introduce heteroatoms into lignin-derived monomers, Yan and colleagues^712^ demonstrated an NH_3_-assisted RCF (Ru/C facilitated) process. This innovative method achieved the direct synthesis of *N*-alkylated phenolic amines. Using 2-butranol as solvent a mixture of monomeric alkylated amines was obtained in 65.6% yield of which 4-[3-(2-butyl)amino]propyl]phenol was the main derivative, in 26.6% yield from poplar sawdust (Table 32, entry 38).

### 3.3 Strategies for *p*-Hydroxycinnamic Acid

*p*-Hydroxycinnamic acids (pHCAs) are found as part of the chemical structure of lignocellulosic material (Scheme 145). In particular, *p*-coumaric acid (pCA) and ferulic acid (FA) can be commonly found in many grassy biomass sources where these are bound to lignin via ester and ether bonds, and are considered to be potential crosslinking sites between lignin and carbohydrate polymers.^729−731^ The contents are low, up to 2 wt%, but typically well below 1 wt%. The standard method to release pHCAs is using a mild version of base-catalyzed depolymerization (BCD).^538,732,733^ At 120 °C using 2% NaOH for 40 min 6.1 g/L pCA could be obtained from corn stover lignin.^734^ This report involved a lignin-rich residue obtained after enzyme hydrolysis and thus a lower amount of FA was obtained (<180 mg/L) as FA is typically more associated with the hemicellulose part of the lignocellulosic material. Alternatively, the use of other bases like lime have been reported yielding both FA and pCA in up to 0.4 g/kg rice straw (which represents 78% and 56% of the total ester bound FA and pCA in the rice straw feedstock) after treatment at 95 °C for 2 h.^735^

Metal chlorides in the presence of alcohols (typically MeOH) have been used for selective release of hydroxycinnamic acids in the form of alkyl esters from lignin. Li and co-workers reported 10.6 wt% (74.1% of total pCA content) and 11.7 wt% methyl-*p*-coumarate upon depolymerization at 160 °C for 8 h in methanol using ChCl[FeCl_3_]_2_ as catalyst from bagasse and corncob lignin, respectively.^736^ Wu et al. reported 12.7 wt% yield of methyl *p*-coumarate from bagasse lignin after treatment with catalytic CuCl_2_ in methanol at 155 °C for 4 h.^737^ Other biomass sources for the lignin could be used as well as other alcohols leading to the respective coumarate esters although in lower yield.

Another method is by catalytic hydrogenolysis or RCF where pHCAs or their various double-bond reduced forms often show up in the product mixtures (see section 3.2.3) usually as esters.^716,738−740^ It has been shown that by using Ni/C and lowering the hydrogen pressure during the hydrogenolysis of miscanthus biomass, the product selectivity can be steered from the double-bond saturated forms to the double-bond containing pHCA esters such as methyl *p*-coumarate and methyl ferulate.^682^ Alternatively, the selectivity can be steered by using different catalysts such as ZnMoO_4_/MCM-41 with which 25.5 wt% and 37.8 wt% of a combined yield of methyl coumarate and methyl ferulate based on lignin content could be obtained from enzymatic mild acidolysis lignin and corncob sawdust, respectively.^741^

However, none of these extraction methods is deemed “natural” for use in the cosmetics and food industries. Therefore, enzymatic approaches have been explored.^742^ Direct extraction from the lignocellulosic material is, however, inefficient and requires a physicochemical pretreatment and/or the addition of (hemi)cellulases. Using a hemicellulosic cocktail, 21.8% FA recovery was achieved based on the original FA content from wheat bran including purification by an ion-exchange resin.^743^

Overall, the principal challenge in the recovery of hydroxycinnamic acids lies in their low concentration in the extraction liquor, attributed to their low content in the parent biomass. This challenge is exacerbated by the multistep recovery process involving solid-liquid extraction, separation, and subsequent purification steps.^742,744^

### 3.4 Conversion of Important Lignin-Derived Phenols

In the section above the focus was on the depolymerization of lignin to specific types of phenolics or mixtures thereof. However, these propyl phenols and vanillin are not base chemicals. Therefore, new conversion routes need to be developed to access other important aromatics. An example is phenol, which is a much-desired chemical.^745^ In addition, the further upgrading of complex mixtures that are rich in aromatic monomers is an important topic. This allows for funneling to less complex mixtures.^412,525^ On the other hand, the unique monomer structures also allow for the conversion to new complex valuable compounds such as pharmaceuticals. Below a selection of important publications on these topics are reviewed.

#### 3.4.1 Funneling to Phenol

Phenol is an important bulk chemical and is among others used to produce caprolactam, phenolic resins and surfactants.^746,747^ Converting lignin to phenol usually involves multiple steps, for example depolymerization and subsequent funneling steps which typically are demethoxylation and dealkylation.^694,748,749^ Yan et al. demonstrated an efficient method for converting lignin-derived monomers into phenol. This process involved hydro-demethoxylation and dealkylation reactions, catalyzed by Pt/C and H-ZSM-5, respectively. Owing to the compatible reaction conditions for these two transformations, the optimal results were obtained by simply physically mixing the two catalysts. When applied to 4-*n*-propylguaiacol, this approach yielded phenol in over 60% yield, with methanol and alkenes as the main side products.^750^ An integrated biorefinery, including reductive catalytic fractionation, extraction, and catalytic funneling (gas-phase hydroprocessing and dealkylation), was developed by Sels and co-workers to valorize lignin to obtain a high yield of phenol and propylene (20 wt% and 9 wt% based on lignin content, Figure 21).^694^ The catalytic funneling can also be achieved by one-pot process, in which MoP/SiO_2_ and H-ZSM-5 (in pellet form, mixed and loaded in a fixed-bed reactor) used for demethoxylation and transalkylation (to benzene), respectively.^751^ The process resulted in 9.6 mol% of phenol based on the lignin content in a pinewood. Catalytic funneling of lignin to phenol can also be realized by an oxidation–hydrogenation strategy. Wang and co-workers developed this strategy for upgrading an organosolv poplar lignin to phenol with high yield (13 wt%) under relatively mild conditions (200 °C, 3 MPa of H_2_, 6 h).^752^

#### 3.4.2 To Non-phenolic Aromatic Products

As seen in earlier sections, phenolic products make up the majority of products obtained from lignin depolymerization strategies. However, these are usually a starting point for diversification into a plethora of other products.^138,412^ In this section a concise overview is provided for the valorization routes, highlighting those that use actual depolymerization mixtures as a starting point.

##### 3.4.2.1 HDO of Depolymerization Mixtures

The depolymerization of lignin by pyrolysis delivers a mixture of various compounds (bio-oil). It consists mainly of alkoxy-phenols and oxygenated aromatics (e.g., guaiacol, methyl guaiacol, syringol, methyl syringol, vanillin, syringaldehyde, vinyl syringol, vinyl guaiacol, and 1,2,3-trimethoxybenzene). Thus, obtaining hydrocarbons (e.g., aromatics and (cyclo)alkanes) necessitates bio-oil upgrading by downstream catalytic hydrodeoxygenation (HDO). HDO of bio-oil involves the presence of a catalyst and hydrogen (50–100 bars) and/or hydrogen donor solvents at moderate temperature (300–600 °C). The oxygen is mostly removed in the form of water. Various catalytic systems have been reported for the HDO of bio-oil: (1) catalyst systems that are used in the petroleum industry, e.g., industrial HDO and hydrodesulfurization (HDS) catalysts including bimetallic Co–Mo or Ni–Mo/Al_2_O_3_, supported and unsupported metal sulfide (CoMoS and NiMoS), transition metal phosphides (Ni_2_P and Co_2_P), and Mo_2_C; (2) various transition metal catalysts, e.g., Ni, Pd, Pt, Ru, etc., catalysts supported on carbon, SiO_2_, Al_2_O_3_, TiO_2_, ZrO_2_, CeO_2_, Nb_2_O_5_, zeolites, etc., preferably with oxophilicity. As a result of the structural units (i.e., *p*-coumaryl alcohol, coniferyl alcohol and sinapyl alcohol), after depolymerization by cleaving C–O and C–C bonds, phenolic hydroxy group and methoxy groups contribute to high content of oxygen in lignin depolymerization mixtures. Therefore, a series of studies have been performed on the removal of phenolic hydroxyl groups and/or methoxy groups.^753^

**Figure 24:**
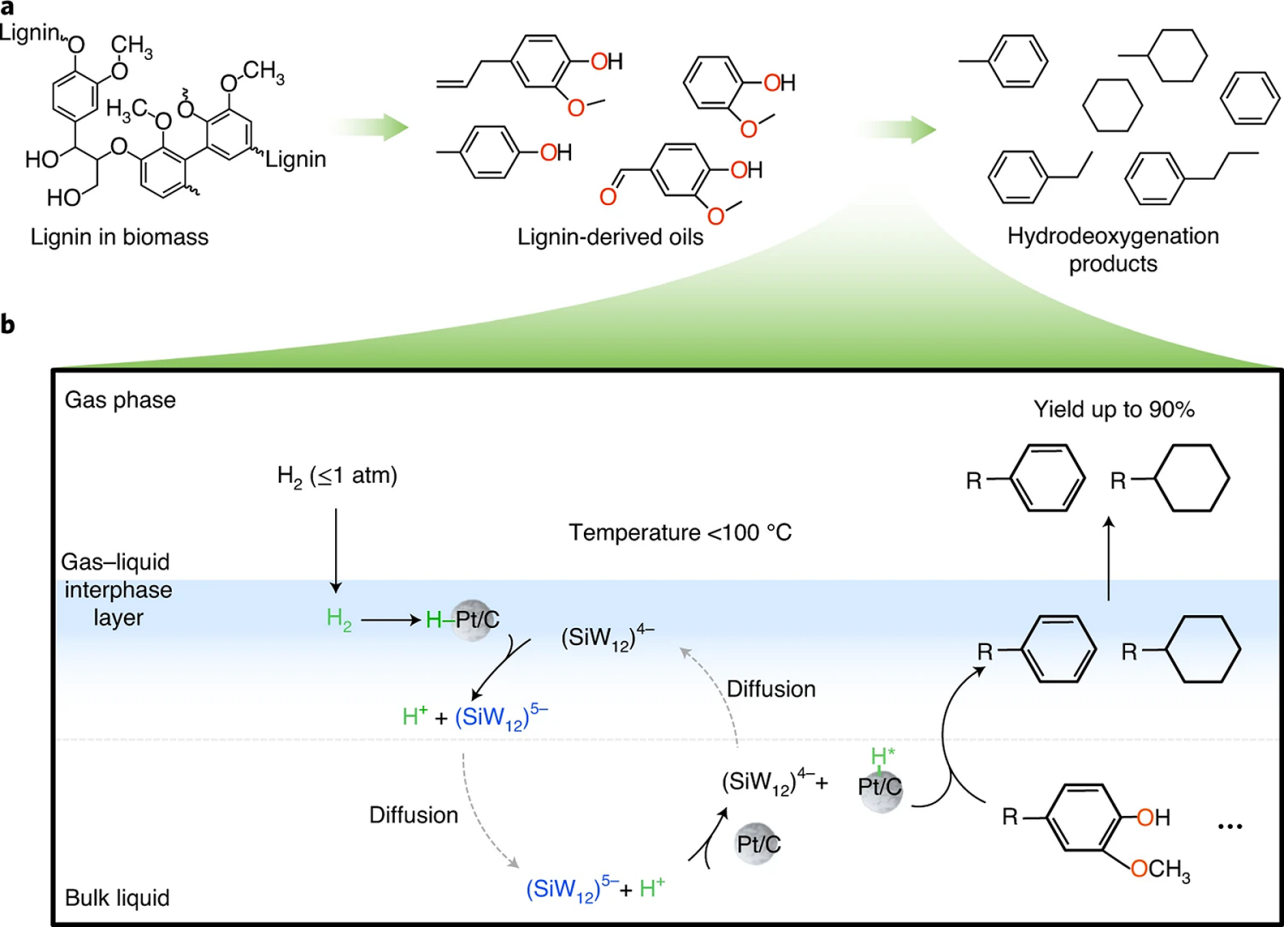
Illustration of hydrogen-buffer-improved bio-oil upgrading. (a) The common structures of lignin, lignin-derived bio-oil, and hydrocarbon products after upgrading. (b) Illustration of the proposed reaction of SiW_12_ hydrogen buffer, which transfers hydrogen gas into the solution over a Pt/C catalyst for HDO. Reproduced with permission from ref (754). Copyright 2023 Springer Nature Limited.

**Figure 25:**
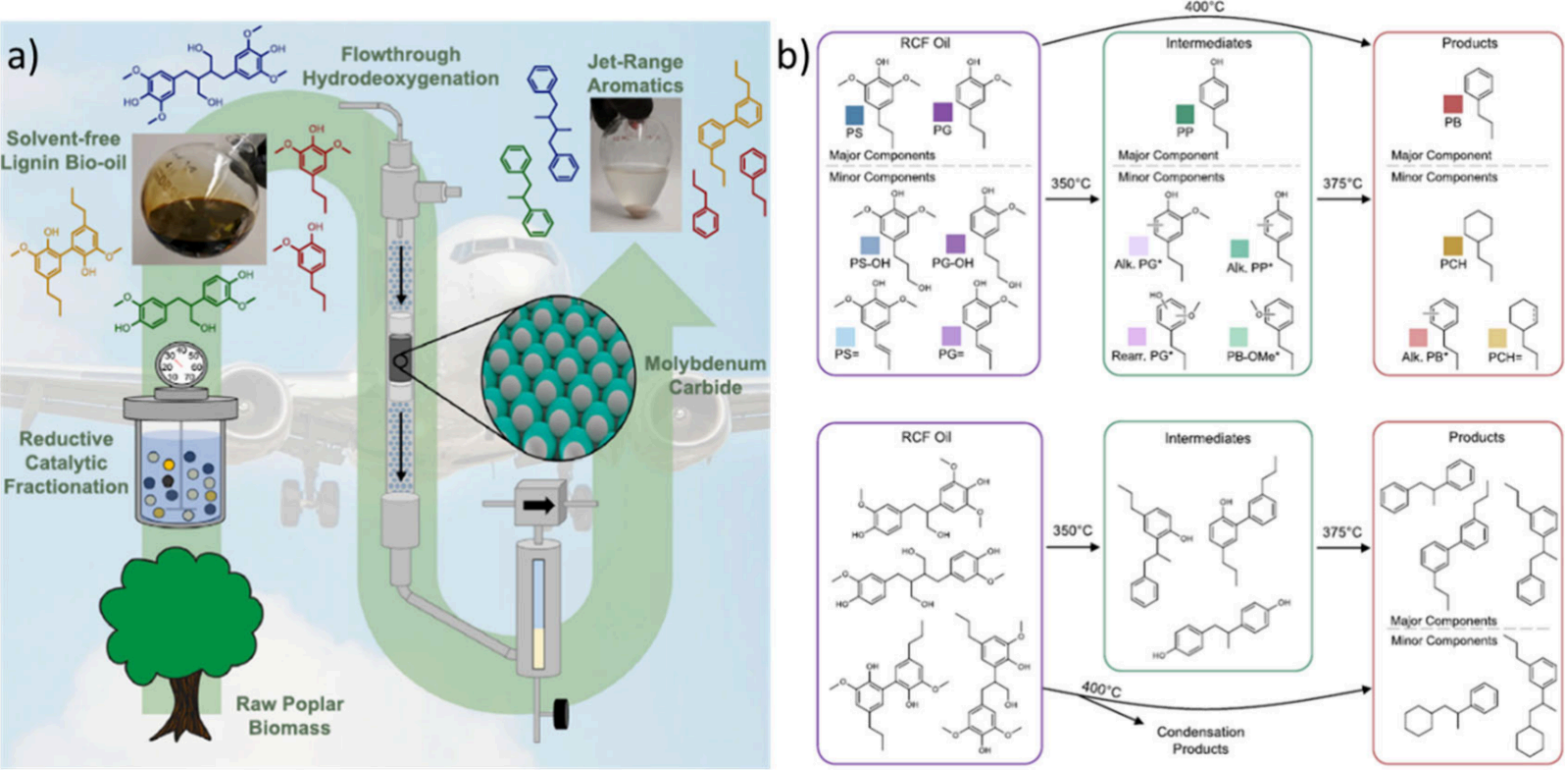
(a) Illustration of continuous hydrodeoxygenation of lignin to jet-range aromatic hydrocarbons. (b) Monomers and dimers identification in in the feed, intermediates, and products during the RCF oil HDO. Reproduced with permission from ref (762). Copyright 2022 Elsevier.

The catalysts, mechanism, and kinetics etc. for lignin oil HDO have been extensively discussed by recent reviews.^1276^ However, the high pressures/temperature required as well as the low selectivities obtained when applying the developed catalysts to real lignin oil instead of model phenols may limit the scalability and economics of the reaction. These obstacles encourage research toward mild and selective approaches for lignin oil upgrading. In the HDO process, hydrogenation metals and Brønsted acidic sites/preferably also with oxyphilic sites are typically active in transforming phenolics to hydrocarbons. Much elegant research has focused on developing catalytic systems with these two functions via, e.g., physical mixing, bimetallic/bifunctional designing, or catalytic system designing. Deng et al. developed a hydrogen buffer synergistic catalytic system (consisting of a low redox potential H_4_SiW_12_O_40_ (SiW_12_) and suspended Pt-on-carbon (Pt/C) particles, achieving HDO of bio-oil-based phenolics to hydrocarbons under ambient pressure at low temperatures (Figure 24).^754^ The SiW_12_ facilitates the bio-oil upgrading by three critical roles, i.e., (i) oxidizing the H_2_ gas to form reduced SiW_12_ in the presence of Pt/C; (ii) transferring both electrons and H^+^ ions to the bulk phase to form active H* or H_2_ on the Pt/C surface; and (iii) formation of oxonium ion in a SiW_12_ superacid solution reduces the deoxygenation energy, which is evidenced by the DFT calculations. Applying this mild HDO method on phenol or phenol derivatives, up to 90% yield of hydrocarbons (benzene, cycloalkane and relevant derivatives) was obtained. However, how this catalytic system performs on real lignin oil remains a question. Ouyang et al. reported a non-noble-metal bifunctional ZrP_2_O_7_-Ni_12_P_5_ catalyst for hydrodeoxygenation of lignin-derived bio-oils to hydrocarbons under relatively mild conditions (i.e., 3 MPa H_2_ and 250 °C).^755^ A highly selective conversion of guaiacol to cyclohexane was achieved (yield of 95.8% at 97.3% conversion) while 36.1% of hydrocarbons can be obtained from HDO of lignin-derived bio-oil. This was attributed to the introduction of a suitable amount of Zr increasing the specific area, pore volume, number of acidic sites, and the adsorption and activation capacity of hydrogen species on the surface of the catalysts. The morphology and mesoporous structure of the catalyst remained even after 5 catalytic cycles, holding promise for further development. Highly selective HDO of lignin monomers to C9 hydrocarbons was reported by Abu Omar et al. under low hydrogen pressure applying a physical mixture of Ru/C and Nb_2_O_5_.^756^ As a result of the synergistic effect of Nb_2_O_5_ (providing acidic sites) and Ru/C (hydrogenolysis, dehydration and hydrogenation), 100% conversion of a simulated lignin oil (a mixture of dihydroeugenol, isoeugenol and 4-allylsyringol) to propyl cyclohexane (76%) and propyl benzene (24%) was achieved. Wang et al. reported a reaction system consisting of a highly stable bifunctional catalyst (i.e., Ni-WO_*x*_/NiAl_2_O_4_), which the crystalline and highly dispersed Ni^0^ species providing the hydrogenation sites with W enhancing the oxophilicity.^757^ Here, a highly stable NiAl_2_O_4_ spinel support was used as a solid acid. This catalytic system not only achieved a high cycloalkane yield of 83.8% from guaiacol (250 °C, 5 MPa H_2_, and 4 h), but also decreased the relative content of phenolics in bio-oil from 58.3% to 20.3%. One of the applications of HDO of lignin-derived bio-oil is tailoring it toward jet fuel (a blended mixture of different hydrocarbon molecules in the range of C9-C16, including paraffins, aromatics, and cycloalkanes). Many reviews have summarized the recent advances in transforming lignin-derived bio-oil to jet fuel.^758−761^ Román-Leshkov et al. developed a continuous, two-stage catalytic process using molybdenum carbide to deoxygenate lignin from poplar into aromatic hydrocarbons at high 86% of the theoretical carbon recovery (Figure 25).^762^ This process relies firstly on the solvent-free RCF of biomass delivering lignin-oil, subsequently the lignin oil is hydrodeoxygenated by earth-abundant Mo_2_C. Besides the two-step conversion of lignin (i.e., lignin to bio-oil and HDO of bio-oil), converting lignin directly to benzene at mild condition represents a promising approach. Han et al. claimed that 18.8 wt% benzene was obtained from lignin catalyzed by high-silica HY zeolite supported RuW alloy catalyst in water.^763^ Shanks and co-workers demonstrated integration of MoO_3_-catalyzed HDO with lignin pyrolysis to attain products with up to 55% carbon yield aromatics of which a majority was non-phenolic.^764^

##### 3.4.2.2 Aromatics by Further Selective Conversion of One or Several Monomeric Compounds Obtained from Lignin

**Scheme 146:**
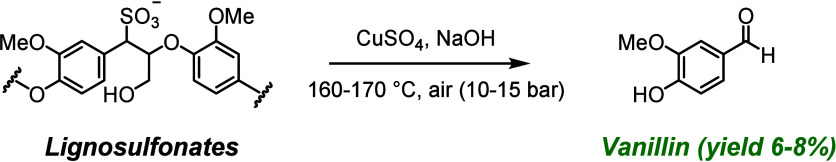
Borregaard Process for the Oxidative Depolymerization of Lignosulfonate to Vanillin

Apart from converting lignin-derived bio-oil as a whole for fuel or bulk chemical applications, separating a single monomer from lignin oil mixtures then applying for fine chemical synthesis can be another alternative way of valorizing lignin-derived products. Vanillin is currently the only monomer manufactured on an industrial scale from lignosulfonates.^765^ The production of vanillin from lignin accounts for around 15% of the annual production (20 000 tons/year).^766^ Vanillin can be derived from oxidative depolymerization of lignin and this has also been proven commercially successful: Norwegian company Borregaard produces bio-based vanillin from lignosulfonate since 1962, for which a Cu^II^ catalyst is applied under alkali conditions in the presence of oxygen (Scheme 146).^463^

**Scheme 147:**
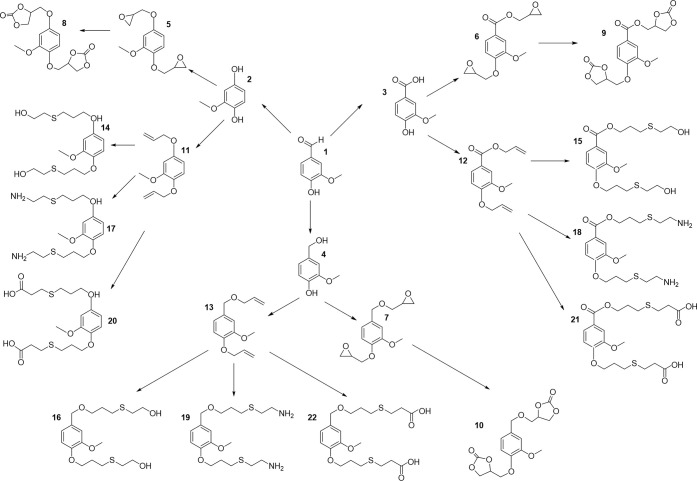
Vanillin Platform for Polymer Synthesis Reproduced with permission from ref (765). Copyright 2014 Royal Society of Chemistry.

This allows for the formation of vanillin with a significantly lower CO_2_ footprint compared to its production from crude oil. Apart from its widespread application in the flavoring industry, vanillin is a bifunctional compound due to the occurrence the phenolic hydroxy and aldehyde groups, and thus can be used for preparing thermoplastic polymers. Caillol et al. applied vanillin as a renewable building block demonstrating a platform of 22 bio-based compounds for polymer chemistry (Scheme 147).^767−769^

By firstly converting vanillin to vanillic acid **3** or even to a methoxyhydroquinone **2** (in the case of overoxidation) and vanillyl alcohol **4**, various functionalities can be built in (i.e., epoxy, cyclic carbonates, allyl, amine, alcohol and carboxylic acid moieties). These vanillin-derived monomers can be used for the synthesis of epoxy resins, polyesters, polyurethanes (PU), and even non-isocyanate polyurethanes (NIPU).

**Figure 26:**
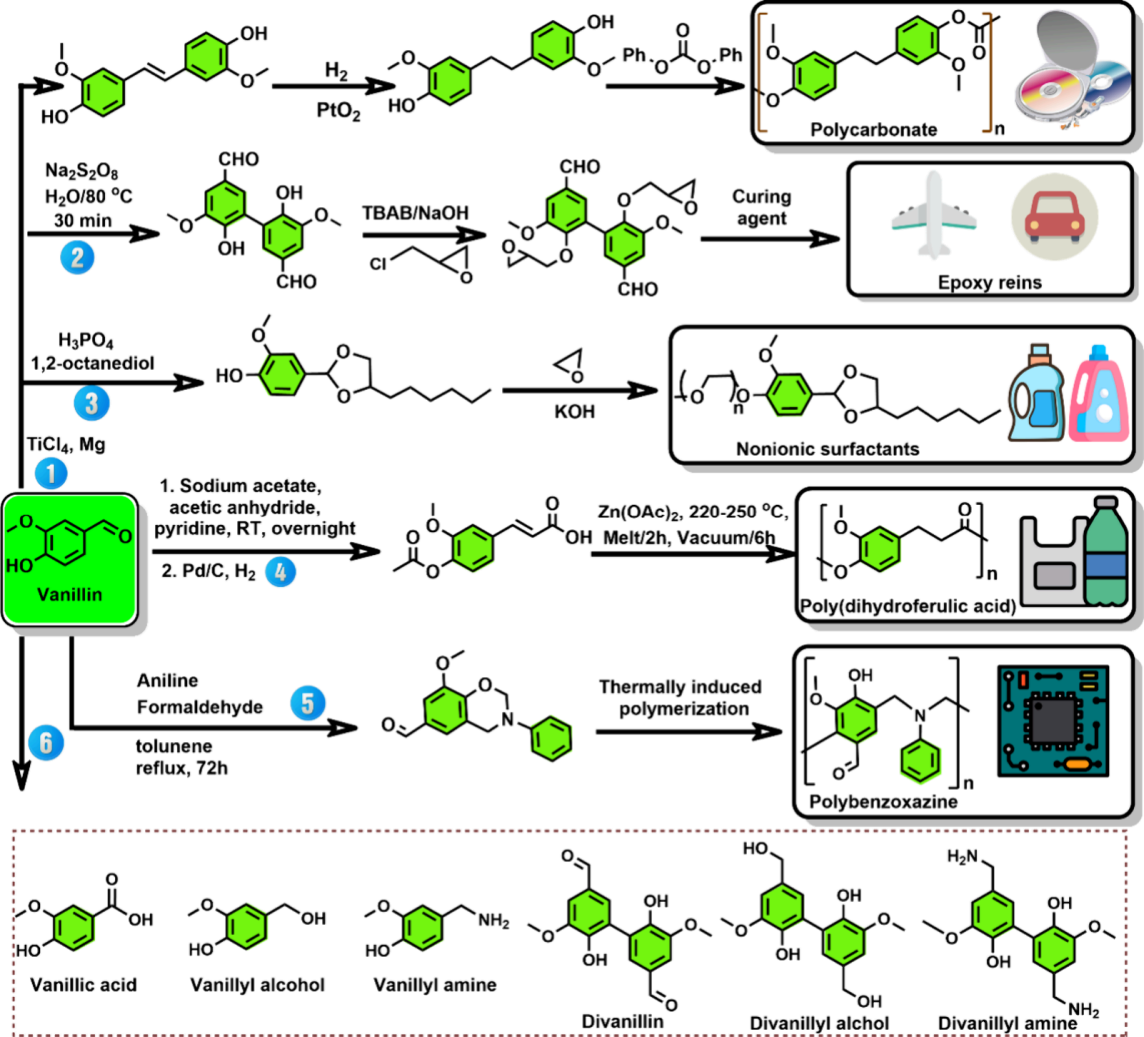
Downstream transformations of vanillin into chemicals, polymers, and surfactants. Reproduced with permission from ref (771). Copyright 2023 by Royal Society of Chemistry.

Zingerone, a key component of the pungency of ginger, was recently reported to be synthesized from vanillin via a two-step approach (i.e., firstly the aldol condensation of vanillin and acetone over recyclable AlPO_4_ as catalyst delivering dehydrozingerone, subsequently, selective C–C double-bond hydrogenation of dehydrozingerone over a Ni/LRC catalyst).^770^ In a recent review from Barta et al.,^771^ the elucidation of the downstream conversion of vanillin into chemicals, surfactants, and polymers has been outlined in detail, as depicted in Figure 26. For a more comprehensive understanding of this topic, we encourage readers to consult this review for detailed information.

**Scheme 148:**
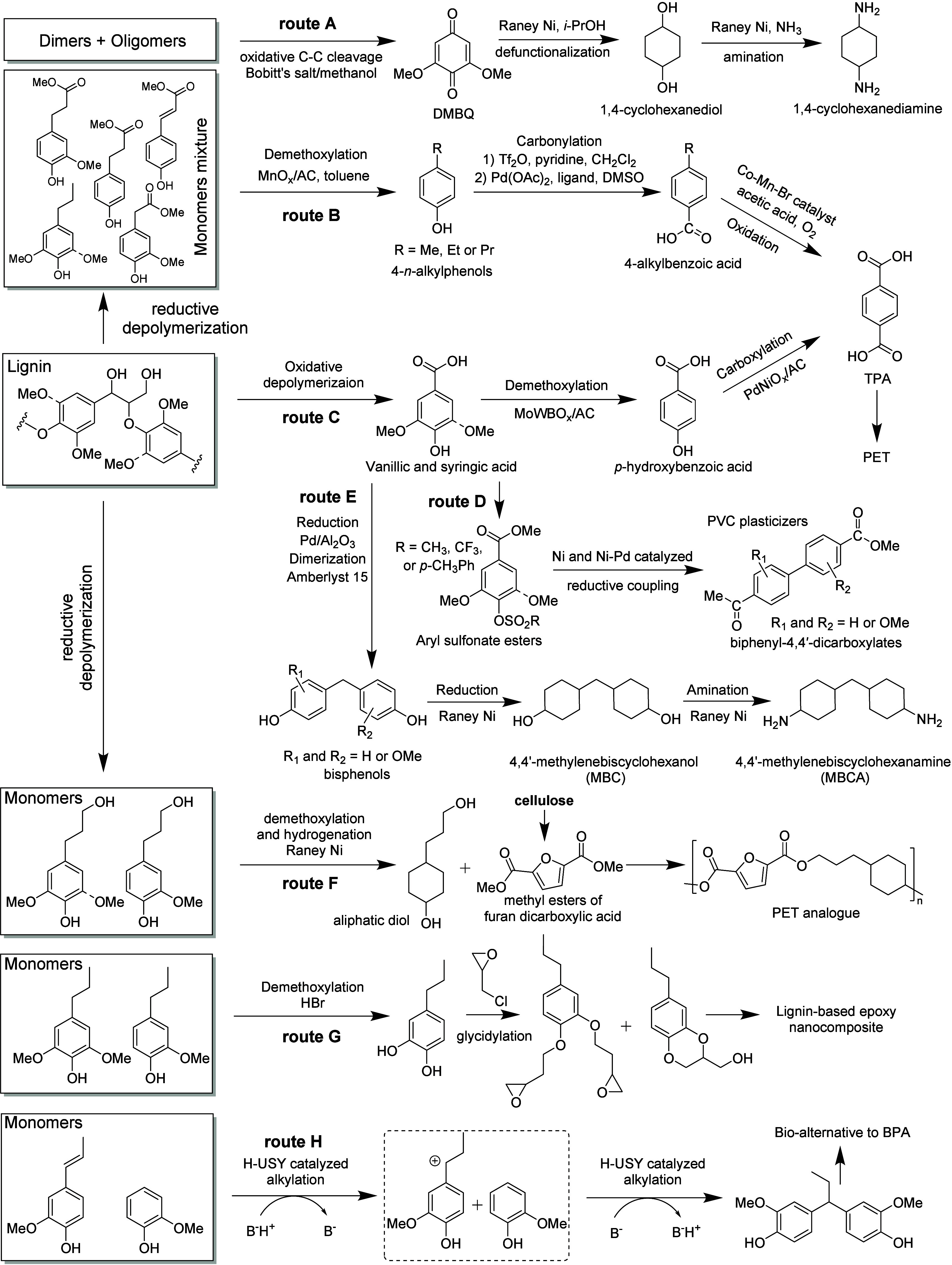
Routes for the Subsequent Transformation of Depolymerized Lignin Monomers, Dimers, and Oligomers for Their Utilization in Polymer Applications

**Figure 27:**
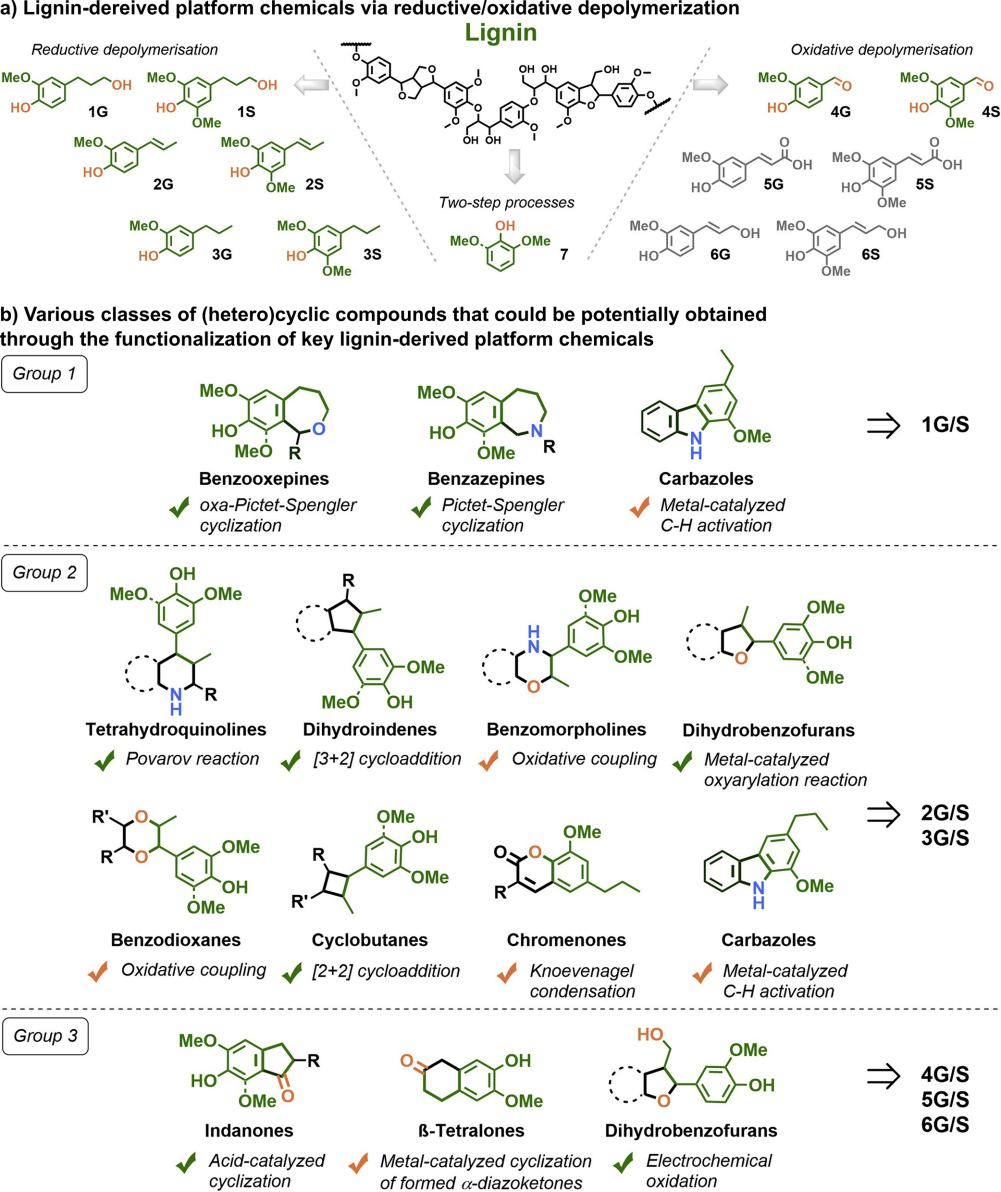
(a) Lignin-derived platform chemicals via reductive/oxidative depolymerization. (b) Various classes of (hetero)cyclic compounds that could be potentially obtained through the functionalization of key lignin-derived platform chemicals. Reproduced with permission from ref (784). Copyright 2021 Elsevier.

Under the RCF scheme (see section 3.2.3.3), the highest monomer yield is around 36 wt%, leaving C–C bond-enriched dimers and oligomers. Aiming at generating value from side-stream, Samec et al. developed an oxidative protocol for the production of additional monomers from the mixture of lignin dimers and oligomers in the RCF oil with Bobbitt’s salt (a tetramethylpiperdine-1-oxoammonium salt) to give 2,6-dimethoxybenzoquinone (DMBQ).^772^ This strategy can for example deliver an extra 18 wt% of monomeric DMBQ (representing a near-quantitive cleavage of C–C bonds inside the dimmers and oligomers) apart from the phenolic monomers from RCF of biomass (Scheme 148, route A). The reduced form of Bobbitt’s salt could be reoxidized electrochemically 5 times. Barta et al.^773^ utilized the acquired monomeric DMBQ, subjecting it to a conversion process that yielded 1,4-cyclohexanediol and 1,4-cyclohexanediamine. These latter two compounds, hold significant industrial promise, serving as possible monomers for polymers and as building blocks for pharmaceuticals. Starting from the monomers fraction in lignin oil from corn stover oxidative depolymerization, terephthalic acid (TPA) was obtained via a three-step strategy (Scheme 148, route B).^774^ The first step relies on MoO_*x*_/AC-catalyzed de-methoxylation. The 4-alkylphenols produced were converted to their triflates which underwent carbonylation through reaction with carbon monoxide, resulting in the formation of 4-alkylbenzoic acids. Subsequently, employing a Co-Mn-Br catalyst, the various alkyl chains within the 4-alkylbenzoic acid mixture underwent oxidation to yield carboxylic acid groups. This transformation ultimately converges the mixture of 4-alkylbenzoic acids into a sole product, namely, TPA (terephthalic acid). This three-step funneling and functionalization conversion achieved a 15.5 wt% yield of TPA with 99% purity based on the lignin content in corn stover. Similarly, Zhu et al. illustrated a TPA conversion pathway originating from vanillic and syringic acids, claiming a two-step transformation process catalyzed by MoWBOx/AC and PdNiOx/AC, respectively (Scheme 148, route C).^775^ Biaryl dicarboxylate esters, serving as bio-based and safer alternatives to phthalate plasticizers, have been shown to be synthesized from oxidative degradation products obtained from lignin, namely, 4-hydroxybenzoic acid, vanillic acid, and syringic acid. Initially, these monomers are converted into aryl sulfonates, and subsequently, an electrochemical method employing Ni- and Ni/Pd-catalyzed reductive coupling is employed to further transform them to dicarboxylate ester (Scheme 148, route D).^776^ A catalytic method was documented for the synthesis of the industrially important compound 4,4′-methylenebiscyclohexanamine (MBCA), utilizing lignin oxidation mixtures as the initial substrate (Scheme 148, route E).^777^ Initially, the oxidative depolymerization monomers from lignin were converted into their corresponding alcohols through the use of a Pd/Al_2_O_3_ catalyst. Subsequently, these obtained products were coupled with phenol to produce methylenebisphenols with the assistance of Amberlyst 15. These methylenebisphenol mixtures were then further hydrogenolyzed to yield 4,4′-methylenebiscyclohexanol (MBC). Finally, the transformation to MBCA was achieved through amination over Raney Ni. The same group also demonstrated that the aliphatic diol 4-(3-hydroxypropyl) cyclohexan-1-ol in high isolated yield can be obtained (11.7 wt% on lignin basis) by funneling the RCF oil (from a process catalyzed by Cu20-PMO). This aliphatic diol was used in the co-polymerization with cellulose-derived methyl esters of furan dicarboxylic acid, developing a PET analogue (Scheme 148, Route F). A bio-based expoxy thermoset was also developed from lignin-based platform chemicals. Abu-Omar et al. utilized the propylguaiacol/syringol monomer mixture derived from the RCF process for bio-based epoxy applications.^778−780^ This firstly relied on the *O*-demethylation to obtain the diphenol or triphenol structure. The obtained phenol groups were next glycidylated to epoxy monomers (Scheme 148, route G). Recently, Sels et al. recently reported a bisguaiacol production from lignin-derived monomers, e.g., isoeugenol and guaiacol, which they achieved by H-USY-catalyzed selective alkylation (Scheme 148, route H), providing renewable and safer bisphenol A substitutes.^781^ Alternatively, such a bisguaiacol can also be obtained from lignin-derived 4-*n*-propylguaiacol by acid-catalyzed condensation.^782^ Synthesizing pharmaceutically significant (hetero)cyclic compounds and natural products using monomers derived from lignin presents a compelling research domain.^783−786^ In particular, with the advancement in lignin depolymerization involving heteroatoms delivering functional aromatics,^787^ the potential for the production of diverse pharmaceutical-related chemicals has been considerably expanded. Barta et al. summarized a series of attractive heterocyclic and/or polycyclic compounds obtained from lignin for application in medicinal chemistry (Figure 27). These compounds could be potentially synthesized from lignin-derived platform chemicals utilizing the inherent chemical functionality.^784^

**Scheme 149:**
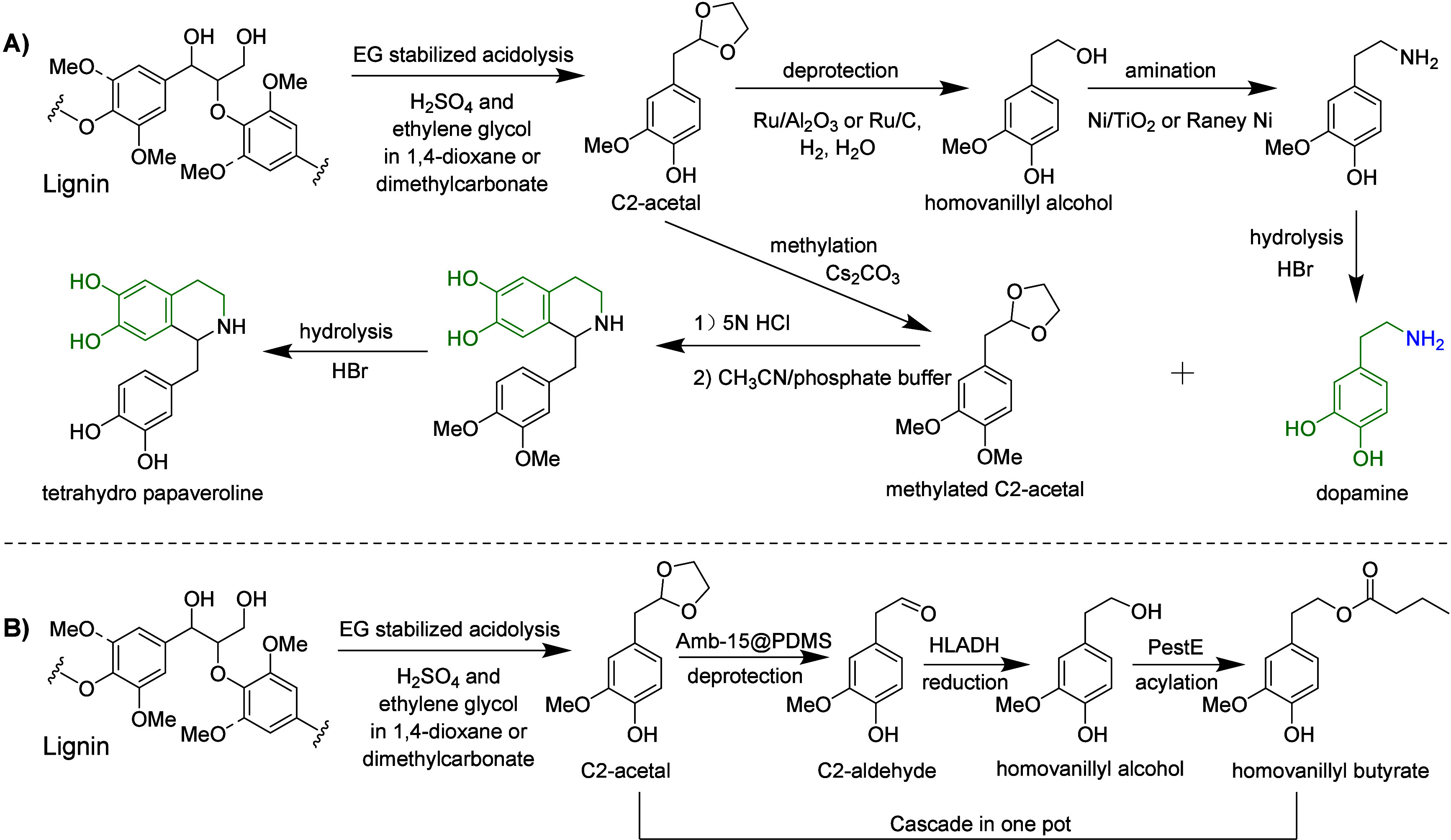
(A) Dopamine Synthesis and (B) Homovanillyl Butyrate Synthesis from Lignin-Derived C2-Acetal

Compounds of Group 1 and Group 2 can be synthesized from the platform chemicals resulting from reductive methods, while platform chemicals from oxidative methods can contribute to the synthesis of compounds of Group 3. The same group achieved the synthesis of a series of seven-membered *N*-heterocycles from lignin RCF depolymerization mixtures taking advantage of the inherent structure of lignin-derived monomers.^788^ These compounds showed promising antibacterial or anticancer activities, demonstrating potential for drug discovery. In another recent work from the Barta group,^789^ a library of dopamine-based biologically active molecules was synthesized from the acetal product of ethylene glycol-assisted lignin acidolysis. Dopamine is an important neurotransmitter and thus this is an interesting example for the defossilization of the pharmaceutical industry by utilizing the innate structural features of the aromatic moiety of lignin. Independently, the groups of Barta and Chen have reported its synthesis from an ethylene glycol (EG)-stabilized lignin acidolysis product known as C2-acetal (as depicted in Scheme 149A).^789,790^ Following lignin acidolysis, a three-step process involving combined acetal deprotection and reduction of the aldehyde group, borrowing hydrogen type amination, and hydrolysis was employed, resulting in the production of dopamine with an overall yield of 6.2 wt% based on lignin.^789^ Furthermore, starting from dopamine, compounds like tetrahydropapaveroline, a natural product, can be synthesized with a yield of 5.6% by coupling dopamine with methylated C2-acetal, which was deprotected to its aldehyde form. Bornscheuer and Deuss et al.^791^ established a chemoenzymatic cascade process for the conversion of the C2-acetal into homovanillyl butyrate, which is a lipophilized derivative of homovanillyl alcohol, with antioxidant properties (Scheme 149B). The deprotection of the acetal moiety was efficiently accomplished using PDMS-protected Amberlyst-15, facilitating subsequent coupling with enzymatic reduction and acylation reactions. This cascade process enables the generation of up to 57% homovanillyl butyrate from C2-acetal through catalytic transformations.

**Scheme 150:**
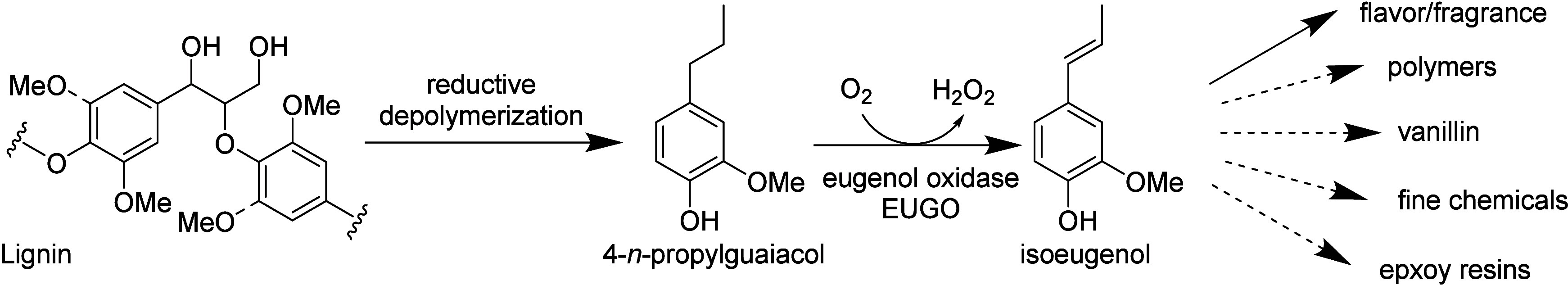
Catalytic Conversion of 4-*n*-Propylguaiacol to Isoeugenol by Engineered EUGO

Fraaije et al. achieved selective dehydrogenation of 4-*n*-propylguaiacol, a typical product from the reductive depolymerization of lignin.^792^ As a result, isoeugenol, a valuable flavor and fragrance molecule and versatile precursor compound, was obtained in a reaction catalyzed by a redesigned natural enzyme (Scheme 150).

**Scheme 151:**
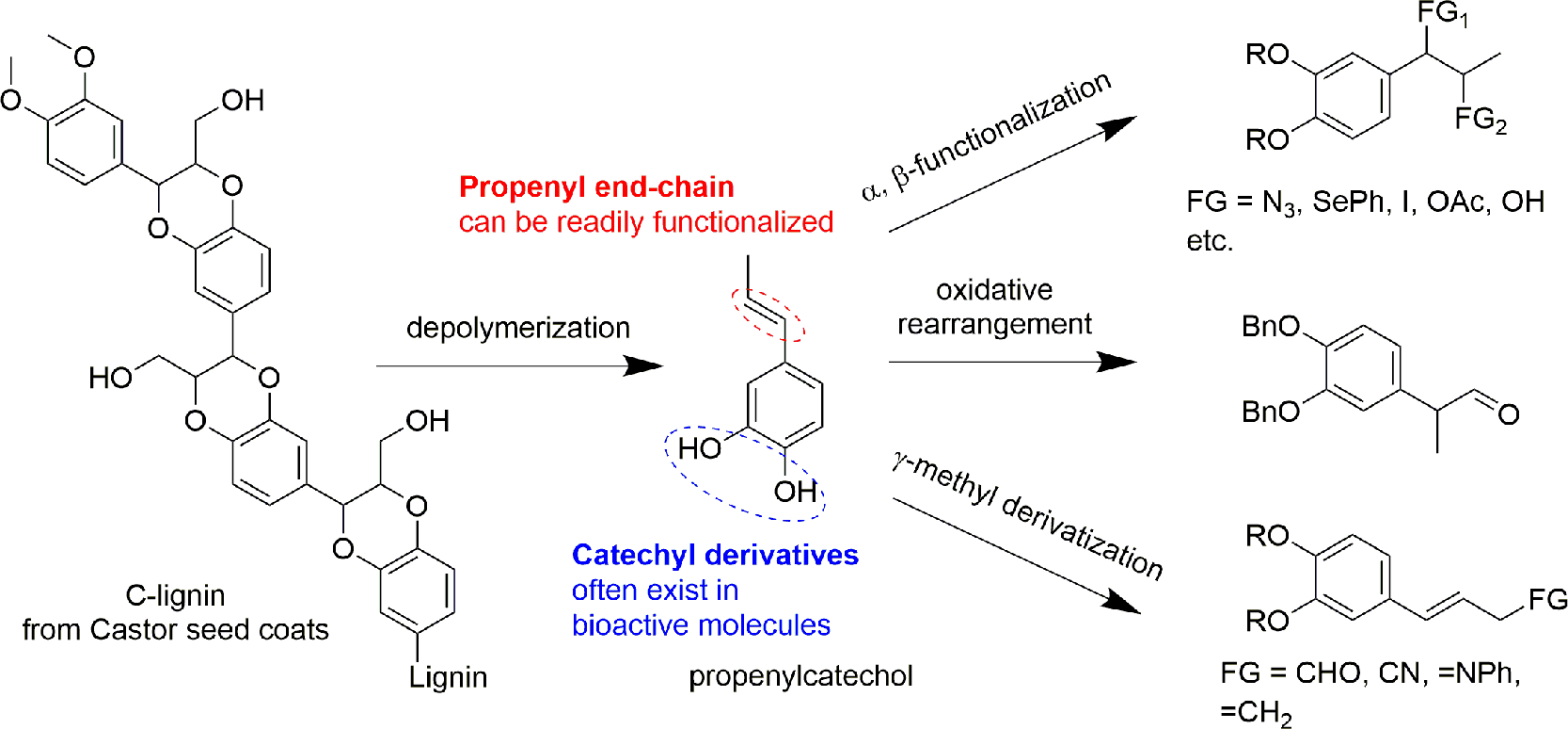
Synthesis of Bioactive Compounds from C-Lignin-Derived Product

As previously mentioned, C-lignin possesses a distinctive and abundant feature in its benzodioxane linkages that can yield monomers with catechol structures. Bioactive compounds have also been derived from the depolymerization product of C-lignin, specifically propenylcatechol. Song et al.^793^ aimed to modify both the catechyl moiety and the propenyl chain of propenylcatechol (Scheme 151). They accomplished this through C=C bond difunctionalization, β-modification, β,γ-rearrangement, and γ-methyl derivatization, resulting in a series of bioactive compounds.

**Scheme 152:**
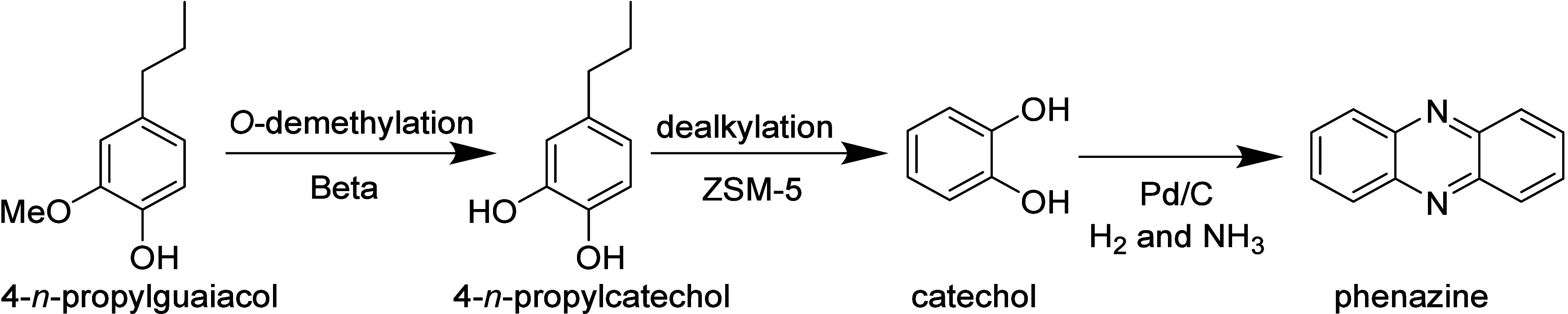
Valorization of RCF-Derived Product 4-*n*-Propylguaiacol to Catechol and Its Further Valorization to Phenazine

Beyond these, there are an increasing number of innovative applications of lignin-derived monomers. For example, Sels et al.^794^ reported the synthesis of bio-renewable catechol from 4-*n*-propylguaiacol, a typical product resulting from the reductive depolymerization of lignin. This synthesis was accomplished through a two-step procedure, involving *O*-demethylation catalyzed by Beta-zeolite, followed by dealkylation mediated by acidic ZSM-5 zeolite (Scheme 152). This approach yielded 56% catechol from 4-*n*-propylguaiacol through the two-step transformation process. Further, Yan et al. demonstrated phenazine can be obtained from lignin-derived catechol.^795^ Phenazine was synthesized in 67% yield and was obtained as high-purity crystals with a purity exceeding 97%, following a one-pot-two-stage reaction conducted over a Pd/C catalyst (Scheme 152).

**Scheme 153:**
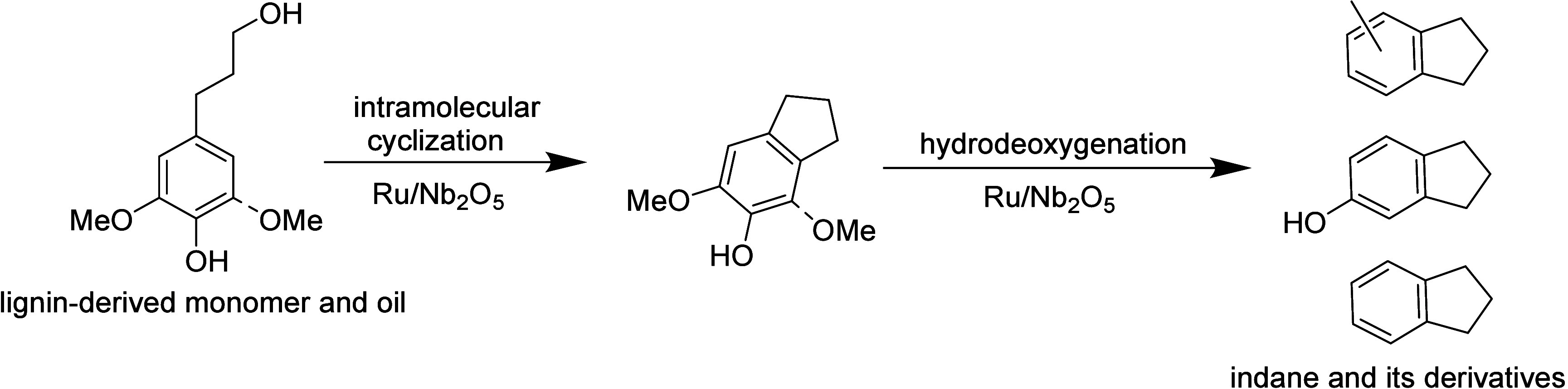
Indane Synthesis from Lignin-Derived Monomers and RCF Lignin Oil

An effective catalytic system to produce indane and its derivatives from lignin-derived monomers and oil has been reported (Scheme 153).^796^ Using a Ru/Nb_2_O_5_ catalyst modified with CH_2_Cl_2_, the intramolecular cyclization and subsequent hydrodeoxygenation of lignin-derived 4-*n*-propanolguaiacol/syringol was achieved by one-pot synthesis. Among various lignin sources, birch-wood lignin oil yielded the highest product yield with 33%, including 23.7% indane and its derivatives.

**Scheme 154:**
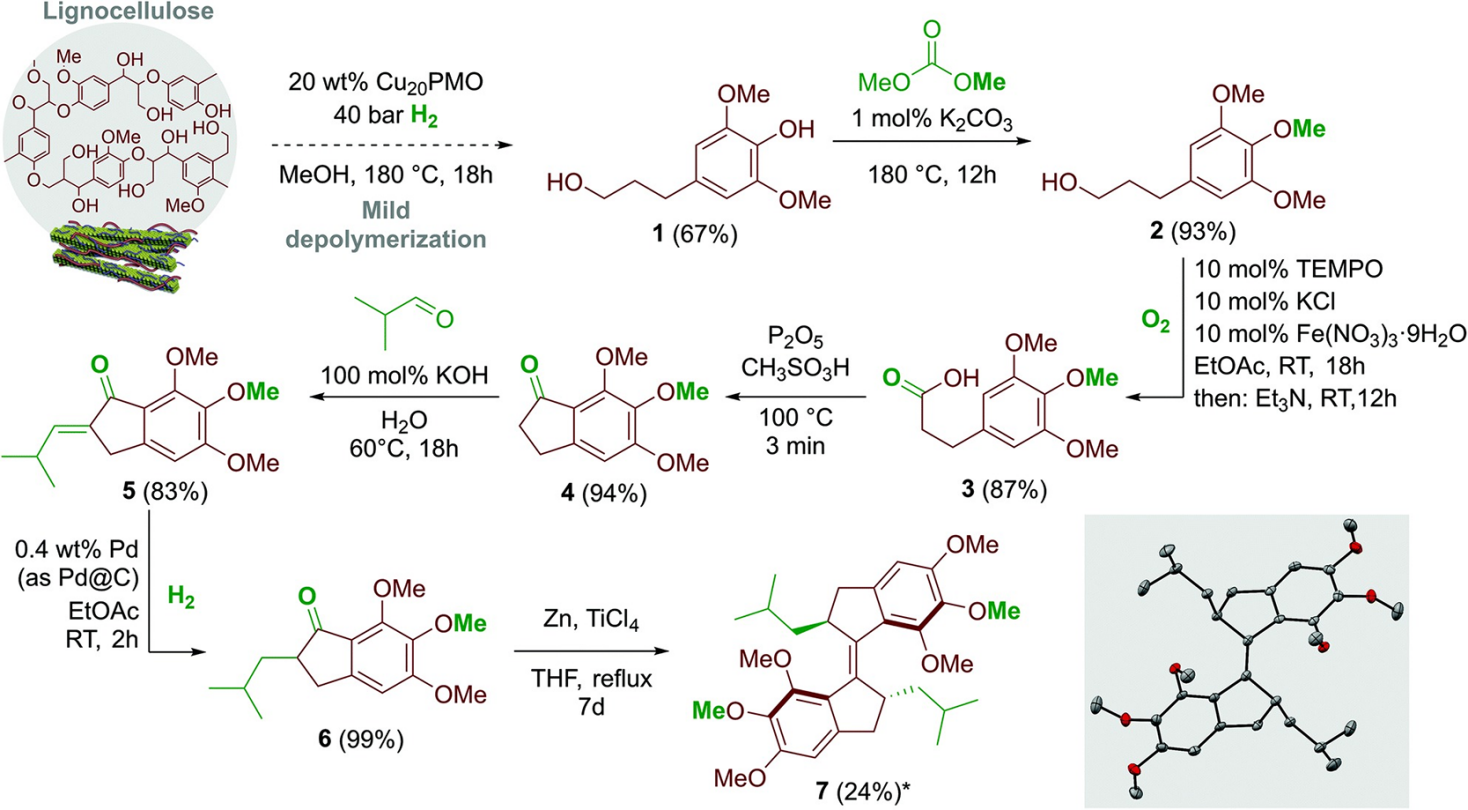
Molecular Motor Developed from Lignocellulose Rcf Monomer: 4-*n*-Propanolsyringol Reproduced with permission from ref (797). Copyright 2022 Royal Society of Chemistry.

With the aim to maintain the intrinsic functionality of the lignin-derived platform molecule a synthesis of a molecular motor was developed from the lignin-derived RCF product 4-*n*-propanolsyringol (Scheme 154).^797^

**Scheme 155:**
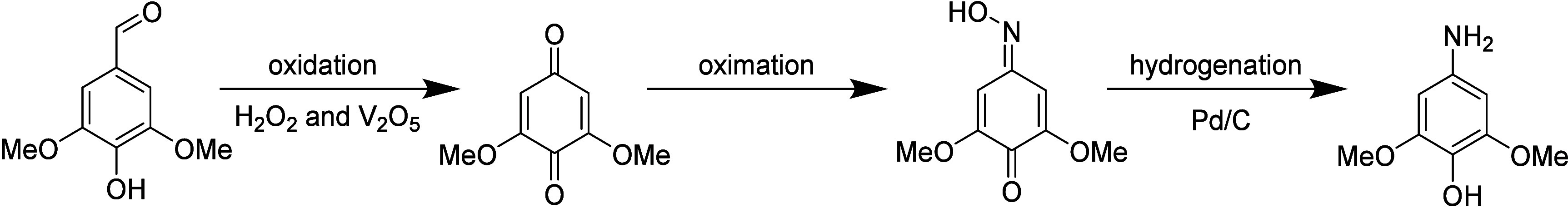
Dimethoxy-*p*-Aminophenol Synthesis from Lignin Oxidative Depolymerization Product

Similarly, to utilizing the inherent functional groups from lignin oxidative depolymerization, 2,6-dimethoxy-*p*-aminophenol was synthesized from lignin-derived oxidative depolymerization products: syringaldehyde.^798^ This was achieved via a three-step strategy, i.e., oxidation, oximation, and hydrogenation (Scheme 155). Applying this method on lignin, an overall yield of 19.8 wt% 2,6-dimethoxy-*p*-aminophenol was achieved on the basis of used lignin.

### 3.5 Non-phenolic Aromatics Directly from Lignin

As shown in the previous sections, the main aromatic products obtained from lignin depolymerization are phenolic in nature. Follow up reactions to other types of aromatics were demonstrated as well. However, significant effort has also been put in direct conversion of lignin to non-phenolic aromatic compounds like BTX. These are of particular interest as these are the base chemicals that lay at the foundation of the current chemical industry and can serve as drop-in chemicals. Catalytic pyrolysis is the most well known, but also several other approaches are discussed in the section below. Furthermore, other catalytic methods and biochemical approaches that can yield amines and more complex aromatic products directly from lignin are highlighted.

#### 3.5.1 Catalytic Pyrolysis

Thermal pyrolysis of technical lignin normally delivers phenolics (*vide supra*). Targeting at non-phenolics (aromatic benzenoid hydrocarbons), catalytic pyrolysis (including both *in situ* and *ex situ* mode^799^) is applied. A large portion of studies have focused on converting lignin to BTEX (i.e., benzene, toluene, ethylbenzene, and xylene, respectively).^800^ These studies are hard to compare as these are performed at a variety of scales and most report the selectivity for a desired component (BTX) in the product oil, as carbon% selectivity or as selectivity of detectable products by GC-MS. Furthermore, lignin sources vary a lot regarding impurities, which may also play a role in the yield. For example, it is known that higher BTX yields can be achieved from carbohydrates compared to a representative native-like lignin like milled wood lignin (roughly around 30 wt% aromatics vs up to 10 wt%, which corresponds to carbon yields up to 25 wt%).^801,802^ This is reflected in the observations that feedstocks with higher H/C ratios give higher BTX yields.^803^ This H/C ratio can be improved by co-pyrolysis with other biomass waste streams like spent cooking oil,^804,805^ waste plastics,^806^ tetralin,^807^ and others.^808^ Other parameters are the various reactor setups and heating rates as well as condensation setups that affect product yields.

One aspect that is certain, however, is that the catalyst is critical in steering selectivity toward BTX.^800,809^ To achieve this, zeolites are the popular choice among other catalysts (carbon-based, and metal-based catalysts), for lignin catalytic pyrolysis because of their accessibility, affordability, thermal stability, acidity, porosity, and superior surface properties. Microporous H-ZSM-5 was found to give the highest yield of aromatic hydrocarbons due to high deoxygenation activity derived from its suitable pore structure (tubular micropores of moderate size (5.5 Å diameter), slightly wider spherical intersections (∼10 Å diameter), and suitable Brønsted acidity. Several studies address the optimization of catalyst parameters such as Si/Al ratio’s, incorporation of other metals, and porosities.^799,810−815^ Alternatively, mesoporous catalysts including MCM-41, (modified) MCM-48,^816^ SBA-15,^817^ and Y-zeolite^818^ have also been applied for the catalytic pyrolysis of lignin toward aromatic hydrocarbons. These are well summarized in recent reviews.^819^ However, generally lower yields of aromatic hydrocarbons are observed than that of ZSM-5, this is due to the lower acidity as well as the lower deoxygenation capability of the mesoporous zeolites, thus more oxygenated aromatics (i.e., phenolics) are formed.

**Scheme 156:**
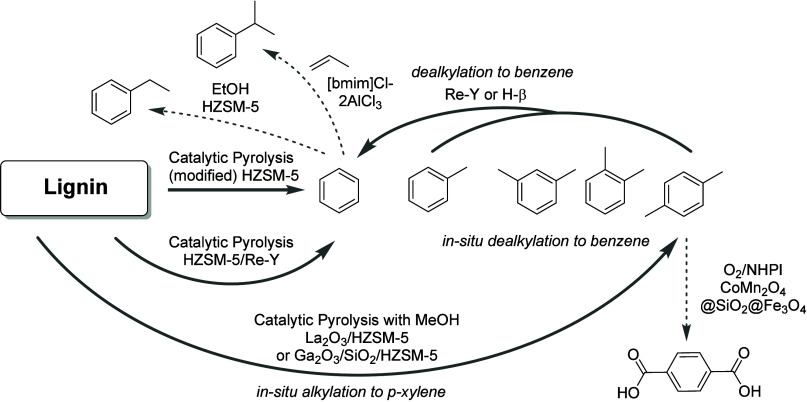
Steering toward Different Aromatic Products Using Catalytic Pyrolysis

The distribution of benzene, toluene and xylenes in the BTX oil also depends on many factors but in general higher toluene and xylene are obtained compared to benzene.^803^ When expressed as selectivity as part of the aromatic mixture the ranges are typically in the range of benzene 5–15%, toluene 20–35%, and xylenes 15–30%.^803,820^ This selectivity can for example be affected by the temperature where it was seen that an increase would shift selectivity to toluene.^821^ Li and co-workers showed that cumene could be produced by a three-step strategy (Scheme 156).^822^ Following the catalytic pyrolysis of sulfur-free wheat straw lignin utilizing 1% Zn/H-ZSM-5, the resulting oil underwent dealkylation over an Hβ-zeolite. This process yielded 93.6 wt% benzene, which was subsequently alkylated using propylene in [bmim]Cl-2AlCl_3_. A yield of 175 g_benzene_/kg_lignin_ was reported by sequentially applying a Ni/H-ZSM-5 catalyst for the catalytic pyrolysis followed by treatment with a Re-Y zeolite for dealkylation. It was also shown to be possible to combine the two catalysts in one step.^823^ Utilizing a Re-Y/HZM-5(25) catalyst, the selectivity of the catalytic pyrolysis could be directly shifted toward benzene with carbon yields of over 20%.^824^ The obtained bio-oil could be alkylated with ethanol using an H-ZSM-5(25) catalyst resulting in a 72.3% carbon yield of ethylbenzene. The same group has also shown that selectivity of the catalytic pyrolysis of the same lignin can be shifted toward p-xylene with 14% carbon yield by the addition of methanol and using La_2_O_3_/H-ZSM-5 as catalysts.^805^ This methodology could be used for the synthesis of terephthalic acid (PTA).^825^

#### 3.5.2 Deep Hydrodeoxygenation

As discussed in section 3.4.2.1, cyclohexanes can be obtained by deep hydrodeoxygenation of lignin oil. They can also be directly obtained from lignin content in the lignocellulosic biomass, for example a Ru/C catalyst (partially oxidized and then reduced) can effectively convert cornstalk to alkylcyclohexanes (from lignin) and polyols (from cellulose and hemicellulose).^268^ Another study showed that the combination of Pt/HAP and Ni/ASA resulted in 42 wt% yield of cycloalkanes from an organosolv lignin.^826^

**Scheme 157:**
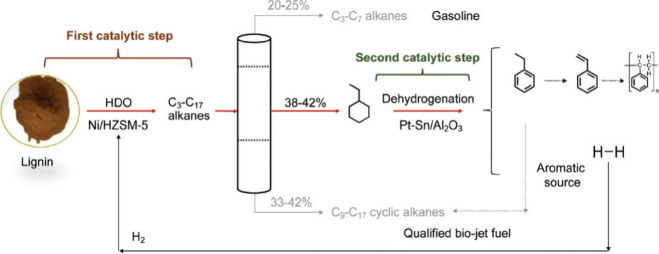
Two-Step Catalytic Process for the Selective Conversion of Lignin to Ethylbenzene Reproduced with permission from ref (827). Copyright 2020 Royal Society of Chemistry.

Additionally, aromatics can be produced by dehydrogenation of lignin-derived cyclohexanes. A two-step catalytic process (Scheme 157) was developed by Zhao and co-workers^827^ to convert hydrolysis lignin to a single aromatic product (viz. ethylbenzene) with a yield as high as 14%. The two-step catalytic process involves hydrodeoxygenation and dehydrogenation. In the first step, the hydrolysis lignin is converted into ethylcyclohexane with high selectivity (42%) using a Ni/Silicalite-1 catalyst at 300 °C, 6.0 MPa initial H_2_ pressure, and 2 h batch time. After distillation, the obtained ethylcyclohexane is dehydrogenated to ethylbenzene with a yield of 99.3% using a PtSn/Al_2_O_3_ catalyst.

#### 3.5.3 Other Catalytic Methods

Li and co-workers reported the ethanolysis of Kraft lignin to aromatics at 280 °C for 6 h employing supported molybdenum-based catalysts.^828^ Using Mo/Al_2_O_3_, 332 mg/g_lignin_ aromatics (note that significant amounts of weight are added due to alkylation), of which 162 mg/g_lignin_ were benzyl alcohols. With α-MoC_1–x_/AC catalyst, selectivity could be shifted toward alkylated monophenols and arenes in 78 and 131 mg/g_lignin_, respectively. Mo_2_N/Al_2_O_3_ yielded 282 mg/g_lignin_ aromatics,^829^ Mo(OCH_2_CH_3_)*x*/NaCl yielded 303 mg/g_lignin_ aromatics.^830^ A catalyst consisting of α-MoC_1–x_ and β-Mo_2_C mixed crystal phases was able to depolymerize Kraft lignin to 516 mg/g_lignin_ of aromatics which are mostly alkylated arenes.^831^ These alkylated arenes are proposed to be formed via base-promoted transfer hydrogenation. β-Mo_2_C supported on macro-porous carbon was reported to yield aromatic mixtures of 543 mg/g_lignin_,^650,832^ while the combination with HZSM-22 under similar conditions resulted in 609 mg/g_lignin_.^833^ The same high yield of aromatic compounds (575 mg/g_lignin_) was achieved via the catalyst synthesized by supporting MoC_1–x_ on CuMgAl mixed oxides (CuMgAlO_*y*_).^834^ The CuMgAl mixed oxides itself was investigated by Hensen and co-workers for depolymerization of lignin to aromatics in supercritical ethanol.^835−838^ The highest yield of aromatic compounds is 39.2% (including 22.7% of oxygen-free aromatics) in this series of work.^835^ Yuen et al. reported 709 mg/g_lignin_ of arenes, mostly BTEX from Kraft lignin, utilizing Mo_2_C_1–*x*_N_*x*_@TiN as catalyst. They also reported that the basic ash contained in the feedstock plays a crucial role in the depolymerization.^839^ In addition to the aforementioned investigations employing molybdenum carbide catalysts, it is noteworthy that Ru-based catalysts, particularly Nb_2_O_5_ supported Ru catalysts, demonstrate effective conversion of organosolv lignins into C7–C9 arenes.^636,840^ Wang et al. found that the use of Ru/Nb_2_O_5_ resulted in a high yield of C7–C9 arenes (20.4 wt%), outperforming other supports like ZrO_2_, Al_2_O_3_, TiO_2_, H-ZSM-5, and activated carbon.^636^

**Scheme 158:**
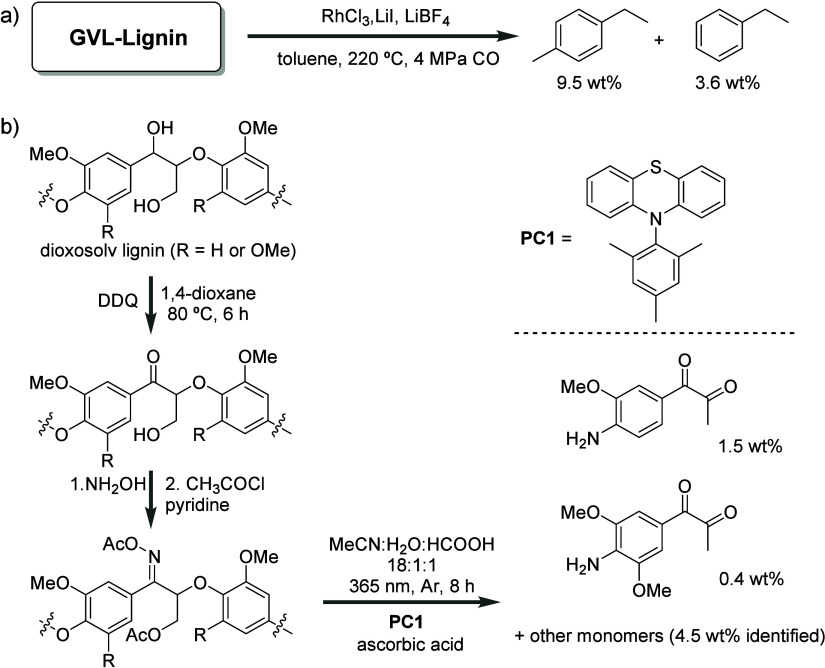
(a) Conversion of GVL-Extracted Lignin to Ethyltoluene^840^ and (b) Photocatalytic Depolymerization of Aminated Lignin to Aromatic Amines^844^

In many depolymerization methodologies that incorporate deoxygenation, the carbon from the aromatic methoxy groups is typically released into the gas phase as methane, methanol, carbon monoxide, or carbon dioxide. Building upon prior research focused on utilizing the methoxy groups to produce methyl iodide (MeI),^841,842^ Han et al. found that a catalytic system of RhCl_3_ in conjunction with LiI and LiBF_4_ under a CO atmosphere could convert mild GVL-extracted birch lignin to 4-ethyltoluene with a yield of 9.5 wt% (Scheme 158a).^843^ Ethylbenzene was the main side-product with 3.6 wt%. The method was also directly applied to poplar wood with claimed yields of 5.2 wt% ethyltoluene and 2.8 wt% ethylbenzene.

The group of Wang devised a strategy to access anilines from lignin, which they demonstrated first using β-O-4 model compounds.^844^ The strategy relies on installing an acetoxyimine functionality to the oxidized β-O-4 motifs which are cleaved to form an iminyl radical using photocatalysis (Scheme 158b). The amine is proposed to be formed by aryl migration to the formed iminyl radicals. When applied to dioxosolv lignin obtained via imine formation after DDQ oxidation,^608^ monomeric anilines were obtained in 1.9 wt% among other (acetylated) phenolic monomers. The poor yield was assumed to be due to the low percentage of α-oximated β-aryl ethers after modification and more restricted polymerized structures in real lignin.

#### 3.5.4 Biochemical

**Scheme 159:**
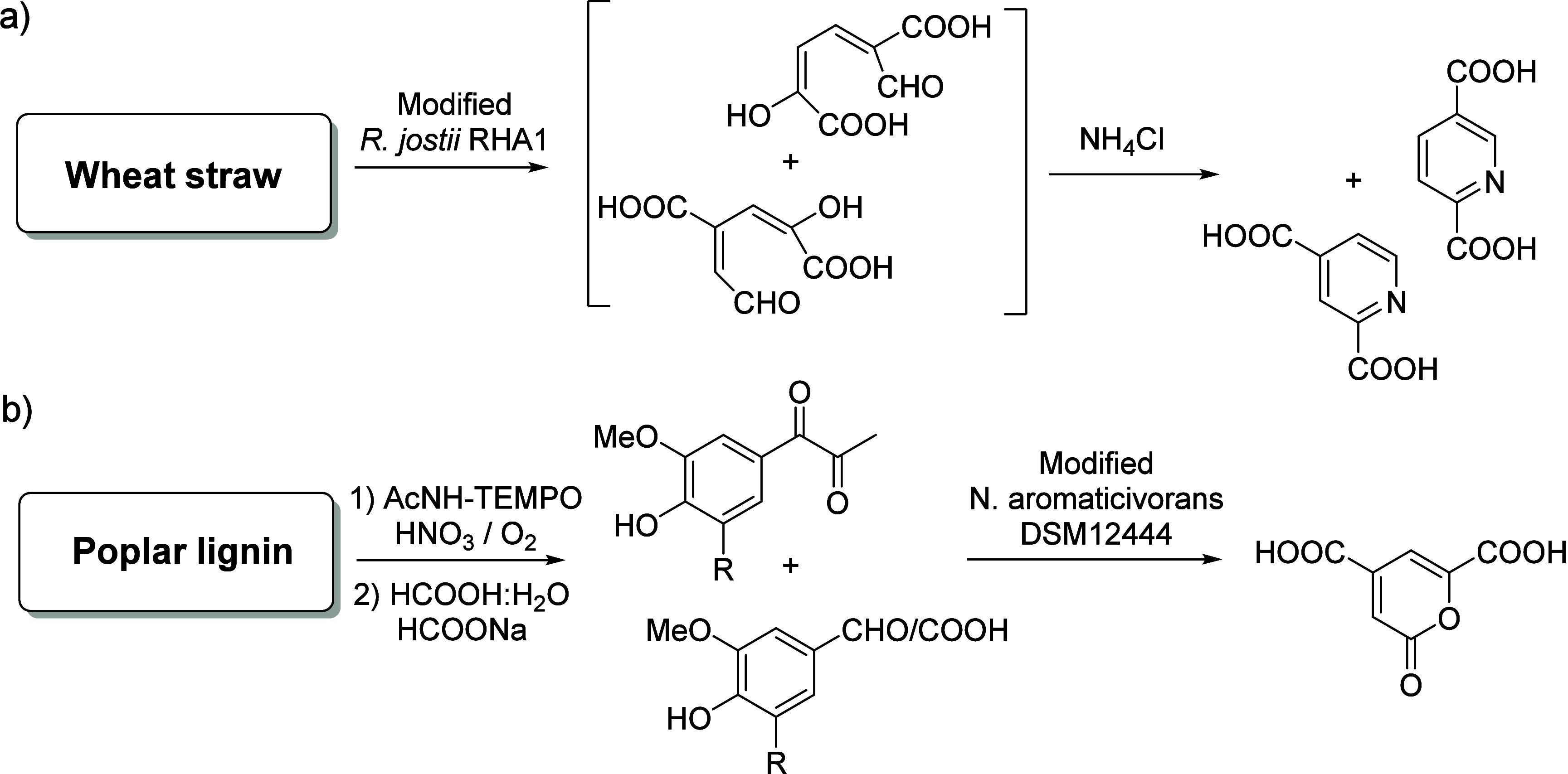
(a) Conversion of Lignin of Wheat Straw to Pyridine 2,4-Dicarboxylic Acid and Pyridine 2,5-Dicarboxylic Acid by Metabolic Engineering and Ammonia Cyclization and (b) Metabolic Conversion of Oxidative Depolymerized Lignin Products to 2-Pyrone-4,6-dicarboxylic Acid

Non-phenolic aromatics can also be obtained from lignin via biotechnological pathways. Bugg et al. reported that the phenolic metabolic pathway of *Rhodococcus jostii* RHA1 can be engineered to enable the direct synthesis of pyridine-2,4-dicarboxylic acid and pyridine-2,5-dicarboxylic acid from a medium containing 1% wheat straw (Scheme 159a).^845^ To achieve this, recombinant genes for protocatechuate 2,3- and 4,5-dioxygenases were inserted in *Rhodococcus jostii RHA1*. In the presence of ammonium chloride, the metabolic intermediates from aromatic ring cleavage rearomatize to the pyridine dicarboxylic acid products in 80–125 mg/L. When the necessary genes were inserted into the chromosome, the competing β-ketoadipate pathway was suppressed and a lignin degrading peroxidase was overexpressed, the yields for pyridine-2,4-dicarboxylic acid could be improved to 330 mg/L from wheat straw, which corresponds to approximately 16% of the lignin input.^846^ In addition, commercial soda lignin (Protobind) could be converted to yield pyridine-2,4-dicarboxylic acid in 240 mg/L. In contrast, Kraft lignin resulted in notably lower yields, primarily due to its condensed structure.

Goodell, Otsuka, and co-workers showed that 2-pyrone-4,6-dicarboxylic acid (PDC) can be accessed via metabolic conversion of monomers extracted from oxidative depolymerization lignin from cedar and birch. Transgenic *P. putida* PDHV85 allowed for efficient PDC accumulation of around 200–250 mg from around 500 mg raw extract.^847^ PDC was shown by Noguera et al. to be accessible via metabolic engineering of other lignin-derived degradation products (Scheme 159b).^848^*Novosphingobium aromaticivorans* DSM12444 was engineered via targeted gene-deletions that shunt the metabolic pathway toward PDC. The product is formed after ring opening of metabolic intermediates protocathechuic acid and 3,4-dihydroxy-5-methoxy-benzoic acid followed by cyclization. A mixture from oxidative depolymerized poplar lignin using a method by Stahl et al.^603^ yielded 0.49 mM PDC corresponding to a 59 mol% yield based on the observed monomer conversion.

### 3.6 Aromatics by Further Conversion of Monomeric Compounds Obtained from Lignin

#### 3.6.1 Aromatics from Cyclohexane

**Scheme 160:**
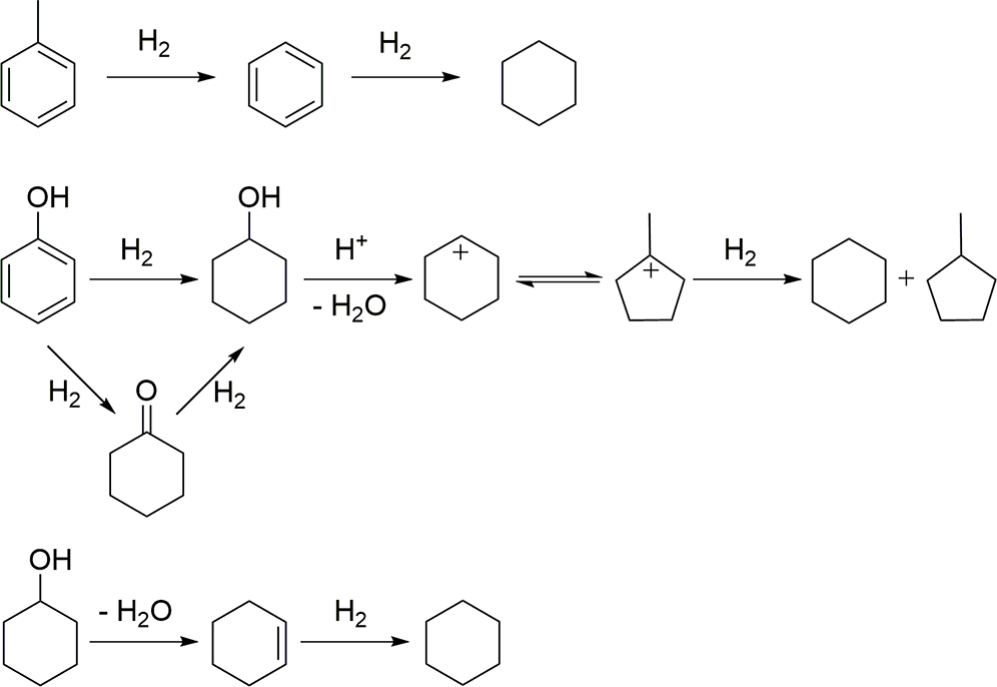
Proposed Reaction Pathways for the Hydrodeoxygenation of Phenols to Aliphatic Hydrocarbon (Cyclohexane and/or Methylclycopentane) Reproduced with permission from ref (849). Copyright 2013 Wiley.

The dehydrogenation of cyclohexane and its derivatives may be envisaged as a more facile approach to make aromatics compared to the linear alkanes/alkenes discussed above. The sustainable supply of cyclohexane is assumed to rely on lignin pyrolysis. The hydrodeoxygenation of phenols to cyclohexane have been already discussed for supported Ni catalysts (e.g., Ni/ZrO_2_-SiO_2_ catalysts were reported to produce cyclohexane with selectivity ≥90%).^849^ These authors proposed a plausible reaction pathway of the hydrodeoxygenation of phenol to cyclohexane which is summarized in Scheme 160.

**Figure 28:**
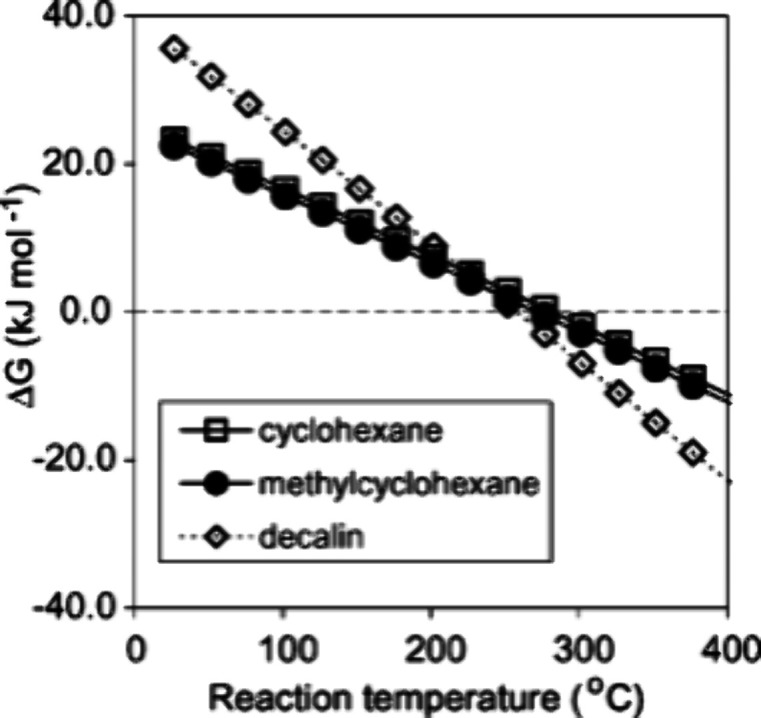
Free energy (*G*) of dehydrogenation of cyclohexane (C_6_H_12_ → C_6_H_6_ + 3H_2_), methylcyclohexane (C_7_H_14_ → C_7_H_8_ + 3H_2_), and decalin (C_10_H_18_ → C_10_H_8_ + 5H_2_) at various reaction temperatures. Reproduced with permission from ref (851). Copyright 2003 Elsevier.

In general, the cycloalkanes were primarily considered as a hydrogen carrier, where their dehydrogenation to aromatics is intended for the production of hydrogen. A typical catalyst for this conversion are oxide supported Pt nanoparticles. The non-oxidative dehydrogenation of cyclohexane is an endothermic process, and produces one molecule of benzene and three molecules of hydrogen (C_6_H_12_ → C_6_H_6_ + 3H_2_; Δ*H* = 205.9 KJ mol^–1^).^850^ This reaction, for different substrates, is thermodynamically feasible at temperatures ≥ 270 °C (see ΔG of dehydrogenation plotted as a function of reaction temperature for different cycloalkanes in Figure 28).^851^

Kaiya et al. studied systematically the dehydrogenation of different cyclic alkanes (cyclohexane, methylcyclohexane, tetralin and decalin) on anodized aluminum (alumite) supported Pt catalysts, including the Pt-bimetallic alloys (Pt-Rh, Pt-Pd, and Pt-Re) at a temperature of 375 °C.^850,851^ Unfortunately these authors focused only on the analysis of the product yield of hydrogen and did not report information on the quantification of aromatics, although the rates of hydrogen formation were related to the rates of benzene/aromatics formation. Akamatsu et al. developed membrane reactors to separate hydrogen from the aromatic yields (benzene) and /or cyclohexane adduct. They employed Pt/Al_2_O_3_ granules with different Pt loadings (2–4 wt%) packed inside the membrane for the dehydroaromatization of cyclohexane and used an amorphous silica membrane which is permeable for hydrogen. This way they could achieve conversions of cyclohexane equal/or higher than the equilibrium conversion (calculated from thermodynamics), almost close to 95% at around 300 °C, which is not achievable with the normal flow reactors,^852^ Coupling this engineering solution with the proper selection of catalytic materials is envisaged as one of the solutions for ameliorating the yields of these thermodynamically limited reactions.

More recently, Li et al. studied the non-oxidative dehydroaromatization of several alkanes (methane, propane, *n*-butane, *n*-hexane) including cyclohexane using gallium nitride catalysts. At 450 °C (using 20 mg of GaN; no information on GHSV) these authors studied the isothermal temporal evolution of catalytic activity of this catalyst for different substrates.^853^ Focusing on cyclohexane it was observed that the conversion of cyclohexane increases continuously with time on stream from about 1% (at the start of reaction, roughly at 20 min) and reaches about 10% after 1300 min. Selectivity for benzene increased more or less in a similar rate as the conversion between 0 and 900 min, reaching about 85% for benzene at 900 min. At extended reaction time (>900 min) no further changes in selectivity for benzene was observed (no further activation and no deactivation). Gallium doped MFI-type zeolites were also reported as active materials for the dehydrogenation of cyclohexane as well as the dehydroaromatization of Propane.^854^ With Ga loading of around 3 wt% maximum BTX yield of about 15% from the conversion of cyclohexane at 530 °C could be achieved (Note that there is no clear mention of the exact conversion and whether or not other products were detected).

## 4 Aromatics from Lignocellulosic Biomass via Catalytic Pyrolysis

**Figure 29:**
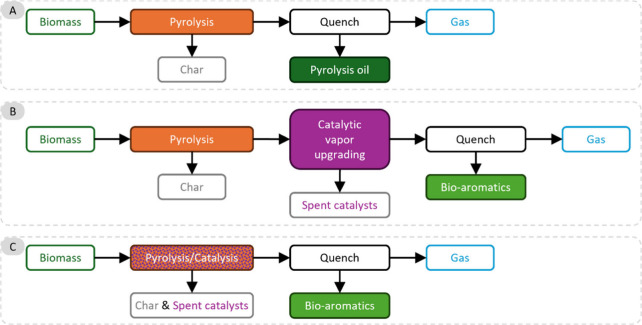
Scheme of pyrolysis of biomass to pyrolysis oil (A) and catalytic pyrolysis of biomass to bio-aromatics via *ex situ* (B) and *in situ* (C) approaches

Lignocellulosic biomass is identified as the cheapest and most abundant circular carbon source, moreover its usage as starting material to produce renewable biofuels/chemicals has many positive consequences in terms of scalability, economic viability, carbon neutrality when the appropriate technologies are used. Within this chapter a detailed overview on the conversion of lignocellulosic biomass to aromatics via catalytic pyrolysis is given. Catalytic pyrolysis of biomass^855−857^ has been developed^858,859^ to improve the property of biomass pyrolysis oil (or pyrolysis liquid, mainly oxygenates such as acids, carbonyls, furans, and phenols) to produce valuable biofuels and bio-based chemicals (e.g., bio-aromatics^860^ including benzene, toluene, and xylenes, abbreviated as BTX) using tailored catalysts (e.g., promoted ZSM-5 catalysts^10^). Different to the (catalytic) upgrading of pyrolysis oils by post treatment, e.g., co-processing the pyrolysis oil in the refineries via hydrotreatment (co-HDT) and fluid catalytic cracking (co-FCC),^861,862^ catalytic pyrolysis aims to convert primary pyrolysis vapor using catalysts present either in the pyrolysis reactor (*in situ* catalytic pyrolysis, Figure 29C) or in a separate reactor (*ex situ* catalytic pyrolysis, Figure 29B).^855,856,863^ Typical byproducts of the catalytic pyrolysis of biomass using aromatization catalysts are, besides the desired BTX, i) other monocyclic aromatics (MAHs) like alkylated benzenes, ii) higher (multiple ring aromatics like (substituted) naphthalenes and anthracenes, iii) coke and iv) gas phase components like CO, CO_2_, and C_1_-C_3_ hydrocarbons. Various reactors such as mg-scale analytical instruments (namely Pyroprobe,^864−868^ Tandem Microreactor (TMR),^869−872^ and Curie Point Pyrolyzer^872,873^) and mg-,^871,874−876^ g-^877−879^ or kg-scale^880^ fixed bed^880−882^ and fluidized bed^883−885^ reactors have been applied. Many biomass types such as woody biomass,^864,886−888^ herbaceous biomass,^889−893^ agricultural residues,^878,894−896^ and forest residues,^874,877,897,898^ have been studied using aromatization catalysts such as FCC catalyst,^894^ Al-SBA-15,^869^ Al_2_O_3_,^884^ LOSA-1,^894^ SAPO-34,^878^ β-type zeolite,^883,899^ Y-type zeolite^869,884,899^ and a dealuminated Y,^886^ MFI-type zeolite^867,869,900−906^ and the modified ZSM-5 with Co,^864^ Ni,^865^ Mo,^865^ Pt,^865^ Fe,^874^ Na,^884^ Ni,^874^ MoZn,^907^ CaO,^893^ and La_2_O_3_.^908^ The technology readiness level (TRL) of catalytic pyrolysis of biomass is about 5–6 considering pilot-scale demonstrations at two companies, BioBTX B.V. and Anellotech Inc.

Herein, we will discuss the effects of (i) reaction conditions (e.g., temperature and weight hourly space velocity (WHSV));^858,859,863,909,910^ (ii) the lignocellulosic biomass source,^807,911−913^ and (iii) the type of catalyst^890,914−930^ on catalytic performance related to BTX yields and we will provide catalyst structure–performance relations.^922−930^

### 4.1 Catalytic Pyrolysis Approaches

Pyrolysis of biomass is a promising thermochemical method to produce bio-based fuels and chemicals using abundantly available lignocellulosic biomass as starting material.^239,931^ Pyrolysis of biomass (Figure 29A) operates at temperature regime of 400–600 °C under a non-oxidizing reaction environment and the intermediately formed pyrolysis vapor is immediately quenched to obtain liquid products (known as pyrolysis oil or liquid).^858^ Pyrolysis technologies can be divided into two main categories based on the applied heating rate and residence time.^911^ Slow pyrolysis (SP) involves slow heating rates of 0.1–1 °C s^–1^ and long residence time varied from minutes to hours.^932^ In contrast to SP, one of the key advantages of fast pyrolysis (FP) is the high yield (up to 70 wt%) of pyrolysis oil, which can be obtained under the beneficial operating conditions namely rapid heating rate of 10–1000 °C s^–1^ and short residence time of <2 s. The primary product of biomass pyrolysis is the so-called pyrolysis oil, which is an extremely complex liquid, containing more than 400 oxygenated compounds that cannot be separated in an economically viable fashion (Table 33).^860,933,934^ The pyrolysis oil as such has limited applications due to its thermal lability, which is assumed to be due to the presence of small reactive aldehydes and ketones.

**Table 33:** Relevant Properties of Pyrolysis Oil and Crude Oil,^863,910,934^ and a Representative Example of a Pyrolysis Oil from the Fast Pyrolysis and Catalytic Fast Pyrolysis of Paper Sludge^935^

Properties and composition	Pyrolysis oil	Pyrolysis oil from fast pyrolysis of paper sludge	Pyrolysis oil from catalytic fast pyrolysis of paper sludge	Crude oil
Water (wt%)	15–30	67.0	0.7	0.1
pH	2.8–3.8	–	–	–
Density (kg L^–1^)	1.05–1.25	–	–	0.86–0.94
TAN (mg KOH g^–1^)	–	49.1	5.2	–
Viscosity (50 °C) (cP)^a^	40–100	–	–	180
HHV^b^ (MJ kg^–1^)	16–19	17.2	41.1	44
C (wt%)	55–65	16.7	85.9	83.86
O (wt%)	28–40	73.2	3.2	<1
H (wt%)	5–7	8.8	9.2	11–14
S (wt%)	<0.05	–	–	<4
N (wt%)	<0.4	1.3	1.7	<1
Ash (wt%)	<0.2	–	–	0.1
H/C	0.9–1.5	–	–	1.5–2.0
O/C	0.3–0.5	–	–	∼0
Hydrocarbons (wt%)	–	–	9.9	–
Aromatics (wt%)	–	5.1	19.7	–
Naphthalenes (wt%)	–	–	9.3	–

a cP = centipoise (dynamic viscosity unit.).

b HHV = higher heating value.

#### 4.1.1 *Ex Situ* Catalytic Pyrolysis

*Ex situ* catalytic pyrolysis involves separate reactors, one for the primary pyrolysis process, followed by vapour phase catalytic upgrading in a second reactor (Figure 29B).^855,856,863^ When aiming for aromatics, as is the prime focus of this chapter, the catalyst should be capable of aromatizing the primary pyrolysis vapors from the first reactor.^929,936,937^ A pilot-scale Integrated Cascading Catalytic Pyrolysis (ICCP) process^938^ for biomass pyrolysis has been operated by BioBTX BV, The Netherlands since 2018 using a continuous fluidized-bed reactor (feeding capacity of 100 kg h^–1^).^939^ Another pilot scale example to obtain bio-aromatics by *ex situ* catalytic fast pyrolysis is the Pyros pilot plant^940^ (ca. 11 kg h^–1^ input). It uses paper sludge as the feed and a fixed-bed catalytic vapor upgrading reactor with an appropriate catalyst.^941^ Relevant product properties are given in Table 33.^935^

#### 4.1.2 *In Situ* Catalytic Pyrolysis

Alternatively, the aromatization catalyst may also be present in the primary pyrolysis reactor, and as such biomass pyrolysis and pyrolysis vapor upgrading take place simultaneously at the same reaction conditions. This approach is known as *in situ* catalytic fast pyrolysis (Figure 29C).^855,856,863^ This technology has been scaled up to pilot scale at a scale of 0.5 ton day^–1^ (Bio-TCat process)^942^ by Anellotech Inc., in the US since 2018. The technology involves a fluidized bed reactor^943^ with continuous catalyst circulation. Bio-aromatics production from *in situ* catalytic fast pyrolysis of loblolly pine on a circulating fluidized bed reactor with a continuous biomass feeding of ca. 38 kg h^–1^ for 30 h was demonstrated by Mante et al.^944^ In situ catalytic fast pyrolysis of loblolly pine (feeding of ca. 1.14 kg h^–1^)^945^ and maize straw (feeding of ca. 5 kg h^–1^)^946^ has also been demonstrated recently.

#### 4.1.3 Reaction Parameters

Reaction temperature,^825,947−949^ weight-hourly-space-velocity (WHSV),^885,950,951^ and catalyst-to-biomass ratio^948,952−954^ are important factors that determine the liquid product composition^897,923,955^ and the extent of coke formation^892,897,950^ for catalytic pyrolysis. For example, Horne et al. reported a threefold increase in the yield of MAHs by increasing the residence time of the pyrolysis vapor in the catalyst bed.^956^ Carlson et al. reported that the yield and the selectivity of the desired products in a fluidized-bed reactor, using wood sawdust as feedstock and H-ZSM-5 as catalyst (*in situ* catalytic pyrolysis), could be controlled by the temperature and WHSV. For instance, the highest aromatic yield was 14 C% at a WHSV of less than 0.1 h^–1^ and decreased to 9.5 C% at a WHSV of 1.7 h^–1^ at 600 °C.^867^ In addition, a lower WHSV and temperature also resulted in higher yields to monocyclic aromatics and a reduced selectivity to polycyclic aromatics (PAHs).^867^ Wang et al. reported that the yield and the selectivity of the desired products from the *in situ* catalytic pyrolysis of organosolv lignin in a fixed-bed reactor (600 °C) using a WO-TiO_2_-Al_2_O_3_ catalyst not only be controlled by temperature but also by the catalyst-to-biomass ratio. The highest BTX yield from organosolv lignin was 1.6 wt% at catalyst-to-biomass ratio of 2, whereas it was reduced to 0.9 wt% at catalyst-to-biomass ratio of 0.6,^875^ higher reaction temperature promotes the conversion of MAHs to PAHs.^875^ Strong temperature effects were also observed for the *ex situ* catalytic pyrolysis of rape straw using red mud and H-ZSM-5 as the catalyst. For a pyrolysis temperature of 450 °C and catalytic vapor upgrading temperature of 550 °C a BTX yield of 10.3 wt% was reported, which is higher than found for a pyrolysis temperature of 450 °C and a catalytic temperature of 600 °C (9.1 wt%).^957^ The reduced yields at higher vapor upgrading temperatures were explained by excessive cracking.

### 4.2 Catalytic Pyrolysis of Lignocellulosic Biomass

#### 4.2.1 Effect of Biomass Source on BTX Yields

All forms of biomass that are suitable for pyrolysis can, in principle, also be considered for catalytic pyrolysis. However, some of them are more preferred based on economic (price and availability), sustainability, and ethical considerations (food versus fuel discussion). Lignocellulosic biomass is one of the most preferred feeds. It is mainly composed of cellulose, hemicellulose, lignin and some extractives like tannins, fatty acids and inorganic salts.^860,910^ Depending on the type of the biomass, the content of each substituents varies drastically. A typical composition of a woody biomass is about 40–47 wt% cellulose, 25–35 wt% hemicellulose, and about 16–31 wt% lignin.^860,910^ Cellulose is a linear polymer composed of 300–1700 glucose units. It forms extremely stable fibres as a result of extensive hydrogen bonding between the single strands. These fibres form the framework of biomass cell walls. The glucose units are linked via a β-1,4-glycosidic linkage. Crystalline and amorphous forms of cellulose exist; however, in nature most of them are highly crystalline.^934^ In contrast, hemicellulose is highly amorphous, which stems from its highly irregular structure. Hemicellulose contains both C5 and C6 sugars, like glucose, galactose, mannose, xylose, and arabinose. The third main element, lignin, is a three-dimensional polymer of propyl-phenol groups linked together with carbon–carbon and ether bonds.^910^ The monomers on which lignin is based are *p*-coumaryl alcohol, coniferyl alcohol, and sinapyl alcohol. They are linked in a radical process via single or multiple C–O and C–C bonds.^910,934,958^

A large number of lignocellulosic biomass sources have been studied for the catalytic pyrolysis to bio-BTX. We here only report experimental studies with quantified data, either on carbon (C%) or mass (wt%) basis and not on GC area percentages, as these are relative values only.^959−963^ The highest quantified BTX yields for various types of lignocellulosic biomass are shown in Tables 34 and 35. In general, the BTX yields from lignocellulosic biomass are lower than 20 C% (Table 34) or 10 wt% (Table 35). This is most likely related to the high content of oxygen in the biomass, which has to be removed by for instance deoxygenation reactions to form CO (via decarbonylation) and CO_2_ (via decarboxylation),^934^ inevitably leading to carbon losses. The highest reported BTX yield of ca. 40 C% on carbon basis was obtained from *in situ* catalytic pyrolysis of corn stover at 550 °C over an H-ZSM-5 catalyst in a fluidized bed reactor (Table 34, entry 1).^947^

**Table 34:** Quantified BTX Yields on Carbon Basis for Catalytic Pyrolysis of Lignocellulosic Biomass^a^

Entry	Feedstock	Catalyst	Reactor	Reaction conditions	Individual yield	BTX yield	Ref
1	Corn stover	H-ZSM-5	Fluidized bed, *in situ*, batch	Catalyst: 10 g, feed: 50 g, *T*: 550 °C, reaction time: 45 min	B: 7.2 C%	40.2 C% (2017)	(947)
					T: 11.2 C%		
					X: 21.8 C%		
2	Pine wood sawdust	Ga_2_O_3_/SiO_2_/H-ZSM-5	Fixed-bed and fixed-bed *ex situ*, continuous	Catalyst: 15 g, WHSV: 2 h^–1^, *T*_p_: 450 °C, *T*_c_: 450 °C, TOS: 30 min	B: 1.3 C%	31.1 C% (2022)	(825)
					T: 5.3 C%		
					X: 24.5 C%		
3	Sugarcane bagasse	H-ZSM-5 (23)	Frontier-TMR, *ex situ*, batch	Catalyst-to-feed ratio: 12, *T*_p_: 450 °C, *T*_c_: 450 °C	B: 5.9 C%	21.2 C% (2018)	(948)
					T: 10.5 C%		
					*p*-X: 4.8 C%		
4	Oak	ZSM-5	CDS-Pyroprobe, *in situ*, batch.	Catalyst: 4.76 mg, feed: 0.24 mg, *T*: 550 °C	B: 4.1 C%	18.9 C% (2017)	(917)
					T: 7.3 C%		
					X: 7.5 C%		
5	Corn fermentation residues	H-ZSM-5	CDS-Pyroprobe, *in situ*, batch	Catalyst: 60 mg, feed: 8.0 mg, *T*: 600 °C	B: 3.5 C%	16.3 C% (2016)	(964)
					T: 7.4 C%		
					X: 5.4 C%		
6	*Grindelia squarrosa*	ZSM-5	Fluidized bed, *in situ*, continuous	Catalyst: 148.5 g, WHSV: 1.0 h^–1^, *T*: 550 °C, TOS: 1 h	B: 6.1 C%	14.9 C% (2022)	(892)
					T: 6.3 C%		
					X: 2.5 C%		
7	*Platycladus orientalis* sawdust	ZSM-5 (12.5)	Fixed-bed, *in situ*, continuous	Catalyst: 4 g, WHSV: 1.5 h^–1^, *T*: 500 °C, TOS: 20 min	B: 5.0 C%	14.1 C% (2020)	(950)
					T: 6.7 C%		
					X: 2.4 C%		
8	Rice stalk	LOSA-1/Al_2_O_3_	Fluidized bed, *in situ*, continuous	Catalyst: 250 g, WHSV: 0.18 h^–1^, *T*: 550 °C, TOS: 30 min	B: 4.1 C%	13.2 C% (2013)	(965)
					T: 6.2 C%		
					X: 2.9 C%		
9	*Fagus sylvatica*	ZSM-5	Frontier-TMR, *in situ*, batch	Catalyst: 3.0 μg, feed: 0.3 μg, *T*: 500 °C	BTX: 11.7 C%.	11.7 C% (2022)	(952)
10	Eucalyptus trunks	Ga_6_-Ni_1_/H-ZSM-5	CDS-Pyroprobe, *ex situ*, batch	Catalyst: 0.36 mg, feed: 0.04 mg, *T*_p_: 600 °C, *T*_c_: 600 °C	B: 2.3 C%	10.2 C% (2017)	(923)
					T: 4.1 C%		
					X: 3.8 C%		
11	*Miscanthus x ganteus*	NiO_5_/ZSM-5	CDS-Pyroprobe, *in situ*, batch	Catalyst: 5 mg, feed: 1 mg, *T*: 600 °C	B: 2.0 C%	10.1 C% (2017)	(891)
					T: 4.0 C%		
					X: 4.1 C%		
12	Pinyon-juniper	H-ZSM-5	CDS-Pyroprobe, *ex situ*, batch	Catalyst: 60 mg, feed: 0.6 mg, *T*_p_: 475 °C, *T*_c_: 475 °C	B: 1.6 C%	8.6 C% (2014)	(889)
					T: 2.2 C%		
					X: 4.8 C%		
13	*Ageratina adenophora*	CaO/H-ZSM-5	Fixed-bed, *in situ*, batch	Catalyst: 2 g, feed: 1 g, *T*: 550 °C, reaction time: 45 min, microwave assisted	B: 2.7 C%	8.3 C% (2017)	(893)
					T: 3.3 C%		
					X: 2.3 C%		
14	Switchgrass	H-ZSM-5	CDS-Pyroprobe, *ex situ*, batch	Catalyst: 60 mg, feed: 0.6 mg, *T*_p_: 475 °C, *T*_c_: 475 °C	B: 1.2 C%	7.2 C% (2014)	(889)
					T: 1.9 C%		
					X: 4.1 C%		
15	Wheat straw	H-ZSM-5 (38)	Fluidized bed and fixed-bed, *ex situ*, continuous	Catalyst: 10 g, WHSV: 2 h^–1^, *T*_p_: 500 °C, *T*_c_: 500 °C, TOS: 0.1 h	B: 1.8 C%	6.9 C% (2018)	(966)
					T: 3.2 C%		
					X: 1.9 C%		
16	Poplar	H-ZSM-5	CDS-Pyroprobe, *ex situ*, batch	Catalyst: 60 mg, feed: 0.6 mg, *T*_p_: 475 °C, *T*_c_: 475 °C	B: 0.7 C%	5.1 C% (2014)	(889)
					T: 1.4 C%		
					X: 3.0 C%		
17	Citrus unshiu peel	H-ZSM-5 (23)	Frontier-TMR, *ex situ*, batch	Catalyst: 2 mg, feed: 2 mg, *T*_p_: 500 °C, *T*_c_: 600 °C	B: 0.9 C%	4.1 C% (2015)	(949)
					T: 2.0 C%		
					X: 1.2 C%		

a Abbreviated aromatics include benzene (B), toluene (T), and xylenes (X). *T*_p_: pyrolysis temperature, *T*_c_: catalytic vapor upgrading temperature.

**Table 35:** Quantified BTX Yields on Mass Basis for Catalytic Pyrolysis of Lignocellulosic Biomass^a^

Entry	Feedstock	Catalyst	Reactor	Reaction conditions	Individual yield	BTX yield	Ref
1	Cedar	H-ZSM-5 (40)	Fixed-bed, *in situ*, batch	Catalyst: 0.25 g, feed: 0.25 g, *T*: 500 °C, reaction time: 3 min	B: 3.9 wt%	14.4 wt% (2022)	(888)
					T: 4.4 wt%		
					X: 6.1 wt%		
2	Wood chips	Ni-Lamellar MFI	Fixed-bed and fixed-bed, *ex situ*, continuous	Catalyst: 5 g, feed: 1 g, *T*_p_: 450 °C, *T*_c_: 420 °C, TOS: 105 min	B: 0.4 wt%	11.6 wt% (2023)	(967)
					T: 1.8 wt%		
					X: 9.4 wt%		
3	Rape straw	Dual CaO/red mud and H-ZSM-5	Fixed-bed and fixed-bed, *ex situ*, batch	Catalyst: 8 g, feed: 4 g, *T*_p_: 450 °C, *T*_c_: 550 °C, reaction time: 45 min	B: 3.2 wt%	11.5 wt% (2023)	(896)
					T: 3.0 wt%		
					X: 5.3 wt%		
4	Poplar	H-ZSM-5 (30)	Frontier-TMR, *in situ*, batch	Catalyst: 6 mg, feed: 0.6 mg, *T*: 600 °C	B: 2.0 wt%	10.6 wt% (2017)	(968)
					T: 4.3 wt%		
					X: 4.3 wt%		
5	Corn stover	H-ZSM-5	CDS-Pyroprobe, *in situ*, batch	Catalyst: 5 mg, feed: 1 mg, *T*: 550 °C	B: 3.2 wt%	9.8 wt% (2011)	
					T: 2.2 wt%		
					X: 4.4 wt%		
6	Switchgrass	H-ZSM-5	CDS-Pyroprobe, *in situ*, batch	Catalyst: 5 mg, feed: 1 mg, *T*: 550 °C	B: 1.9 wt%	8.4 wt% (2011)	(890)
					T: 3.4 wt%		
					X: 3.1 wt%		
7	Pine	Ce Mo2N-H-ZSM-5	CDS-Pyroprobe, *ex situ*, batch	Catalyst: 1.5 mg, feed: 0.3 mg, *T*_p_: 750 °C, *T*_c_: 750 °C	B: 2.0 wt%	8.2 wt% (2018)	(954)
					T: 3.8 wt%		
					X: 2.4 wt%		
8	*Quercus mongolica*	Ga/H-ZSM-5	Fixed-bed and fixed-bed, *ex situ*, batch	Catalyst: 5 g, feed: 15 g, *T*_p_: 650 °C, *T*_c_: 550 °C, reaction time: 120 min	B: 0.5 wt%	7.2 wt% (2021)	(876)
					T: 3.0 wt%		
					*o*-X: 1.2 wt%		
					*p*-X: 2.5 wt%		
9	Bamboo residues	Fe-Zn/H-ZSM-5	CDS-Pyroprobe, *in situ*, batch	Catalyst: 0.67 mg, feed: 0.33 mg, *T*: 500 °C	B: 0.6 wt%	6.1 wt% (2023)	(898)
					T: 2.8 wt%		
					X: 2.7 wt%		
10	Rice straw	Ni/ZSM-5	CDS-Pyroprobe, *in situ*, batch	Catalyst: 0.08 g, feed: 0.02 g, *T*: 500 °C	B: 1.9 wt%	5.7 wt% (2023)	(969)
					T: 2.6 wt%		
					X: 2.2 wt%		
11	*Quercus variabilis*	H-ZSM-5	CDS-Pyroprobe, *in situ*, batch	Catalyst: 5 mg, feed: 1 mg, *T*: 600 °C	B: 1.5 wt%	5.4 wt% (2019)	(970)
					T: 3.0 wt%		
					X: 0.9 wt%		
12	Poplar sawdust	Fe-Ni/ZSM-5	Fixed-bed, *in situ*, batch	Catalyst: 0.5 mg, feed: 0.5 mg, *T*: 550 °C, reaction time: 60 min	B: 1.1 wt%	5.2 wt% (2022)	(971)
					T: 1.6 wt%		
					X: 2.5 wt%		
13	Palm kernel shell	Fe_1_/Hβ	Fixed-bed and fixed-bed, *ex situ*, continuous	Catalyst: 5 g, WHSV: 6 h^–1^, *T*_p_: 500 °C, *T*_c_: 500 °C, TOS: 1 h	B: 0.4 wt%	4.7 wt% (2016)	(972)
					T: 2.0 wt%		
					X: 2.3 wt%		
14	Oak	Hierarchical H-ZSM-5	Fluidized bed, *in situ*, batch	Catalyst: 5 g, feed: 4.25 g, *T*: 500 °C	B: 0.7 wt%	4.6 wt% (2016)	(919)
					T: 2.0 wt%		
					*p*-X: 1.9 wt%		
15	Miscanthus	ZSM-5	Spouted bed, *in situ*, continuous	Catalyst: 20 g, WHSV: 12 h^–1^, *T*: 600 °C	B: 1.2 wt%	3.9 wt% (2014)	(955)
					T: 1.6 wt%		
					X: 1.1 wt%		

a Abbreviated aromatics include benzene (B), toluene (T), and xylenes (X). *T*_p_: pyrolysis temperature, *T*_c_: catalytic vapor upgrading temperature.

**Figure 30:**
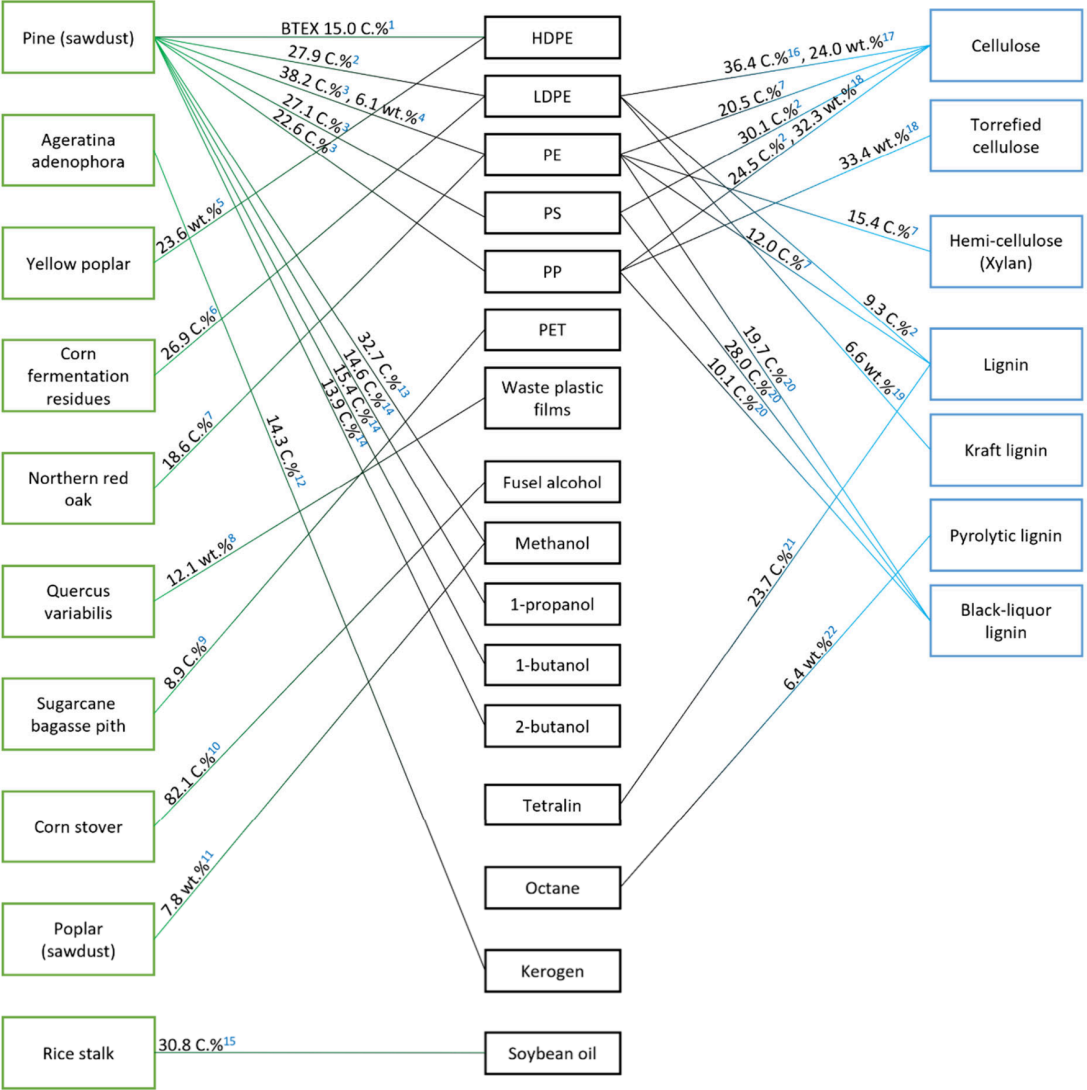
Quantified mass and carbon yields of BTX from catalytic co-pyrolysis of lignocellulosic biomass and co-feeds using various catalysts, including 1. Fe_4_Mo_4_/ZSM-5,^976^ 2. ZSM-5,^977^ 3. LOSA-1,^974^ Spent FCC,^974^ 4. H-ZSM-5,^866^ 5. H-ZSM-5(30),^968^ 6. H-ZSM-5,^964^ 7. H-ZSM-5,^978^ 8. H-ZSM-5,^970^ 9. Na_2_CO_3_/γ-Al_2_O_3_/H-ZSM-5(20/80),^979^ 10. H-ZSM-5,^947^ 11. H-ZSM-5(25),^951^ 12. CaO/H-ZSM-5(25/75),^893^ 13. Ga_2_O_3_-SiO_2_/H-ZSM-5,^825^ 14. H-ZSM-5,^931^ 15. ZSM-5,^980^ 16. ZSM-5,^981^ 17. H-ZSM-5,^982^ 18. H-ZSM-5,^983^ 19. MgO/C,^984^ 20. LOSA-1,^985^ Spent FCC,^985^ 21. HY(5.1),^807^ and 22. MoO_3_.^986^

It has been shown that the H/C_eff_ ratio of the feed is an important parameter that impacts the BTX yield, with higher values leading to better results. This effect of H/C_eff_ ratio has been systematically studied by Huber et al.,^239^ who investigated 10 different feedstocks such as glucose, glycerol, sorbitol, tetrahydrofuran, methanol and various hydrogenated pyrolysis oil fractions using an H-ZSM-5 catalyst. Lignocellulosic biomass has a relatively low H/C_eff_ ratio (less than 0.3) and as such co-feeding with a source with a higher H/C_eff_ may be an attractive approach to increase BTX yields. Co-feeding H-donors such as methanol,^912^ tetralin,^807^ and propylene/furan and propylene/2-methylfuran^283^ with the bio-based feedstock has been applied to increase the H/C_eff_ ratio of the feedstock for catalytic pyrolysis.^910,973^ The H/C_eff_ ratio can be also tuned changing the relative amounts of the co-feeds. Plastics and alcohols are the most-widely used co-feeds for the catalytic co-pyrolysis of lignocellulosic biomass. Waste plastics have been (co-)fed to the two pilot units originally designed for biomass pyrolysis (BioBTX B.V. and Anellotech Inc.). Quantified mass and carbon yields of BTX for the catalytic pyrolysis of lignocellulosic biomass with various co-feeds and catalysts are shown in Figure 30. The highest BTX yield of 82.1 C% was reported by Zhang et al., who co-fed fusel alcohol with corn stover in a 1:1 ratio (Figure 30, entry 10). As expected, this BTX yield is higher than that for the catalytic pyrolysis of corn stover alone (40.3 C%).^947^ Another example to illustrate positive effects of co-feeding is from Zhang et al. When co-feeding polyethylene with pine wood (1 to 1 ratio) in a fluidized-bed reactor using a spent FCC catalyst, a BTX yield of 23.7 C% was obtained, which is remarkably higher than that for the catalytic pyrolysis of pine wood alone (6.4 C%).^974^ This is also much higher than the calculated BTX yield (13.3 C%) based on the feed ratio and individual BTX yield, indicating synergistic effects. Such positive effects were also demonstrated for methanol as the co-feed. For instance, when using methanol and pine wood (23/15 ratio) as the feed, a BTX yield of 16.7 C% was obtained, which is also higher than that for the catalytic pyrolysis of pine wood alone (4.8 C%)^931^ and the calculated value of 6.4 C%. The amount of CO/CO2 in the off gas was reduced, the formation of hydrocarbons seemed to compete with decarbonylation and decarboxylation, while improved hydrocarbon conversion was detected.^912,975^

#### 4.2.2 Effect of the Constituents: Cellulose, Hemicellulose, and Lignin

**Figure 31:**
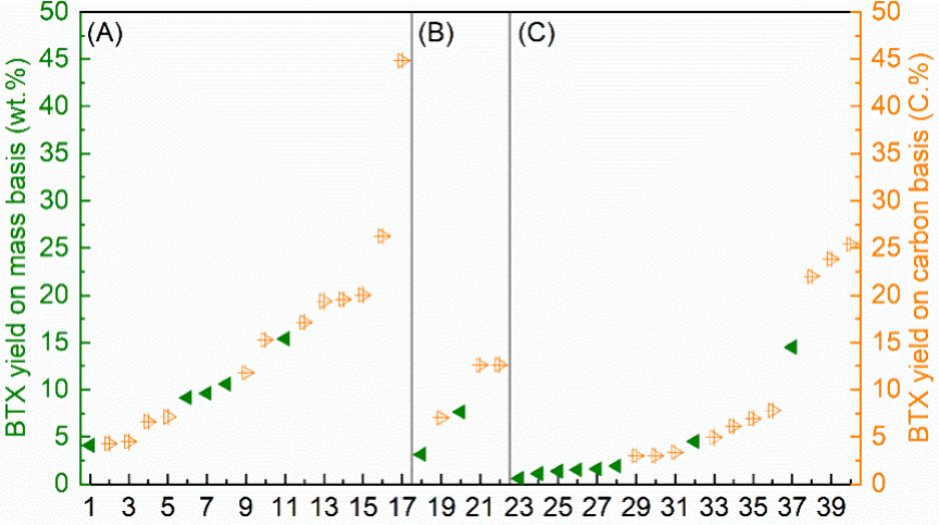
Quantified BTX yields for catalytic pyrolysis of cellulose (A), hemicellulose (B), and lignin (C) over various catalysts, including 1. HY,^987^ 2. MCM-41,^988^ 3. CaO,^988^ 4. ZSM-5/MCM-41(85/15),^988^ 5. ZSM-5/CaO(85/15),^988^ 6. Fe/Hβ,^972^ 7. Hβ,^972^ 8. H-ZSM-5,^987^ 9. MoO/H-ZSM-5,^989^ 10. B/H-ZSM-5,^873^ 11. H-ZSM-5/HY(70/30),^987^ 12. Fe_3_O_4_/SiO_2_/H-ZSM-5,^990^ 13. Mo_2_C/H-ZSM-5,^989^ 14. NaOH-treated H-ZSM-5,^864^ 15. NaOH-treated ZSM-5,^917^ 16. Pt/HF-ZSM-5,^991^ 17. ZSM-5,^977^ 18. Hβ,^890^ 19. MoO/H-ZSM-5,^989^ 20. H-ZSM-5(23),^890^ 21. H-ZSM-5,^989^ 22. Mo_2_C/H-ZSM-5,^989^ 23. HY,^992^ 24. MoO_*x*_-TiO_2_/Al_2_O_3_,^875^ 25. TiO_2_/Al_2_O_3_,^875^ 26. WO_*x*_/TiO_2_,^875^ 27. WO_*x*_-TiO_2_/Al_2_O_3_,^875^ 28. Fe/Hβ,^972^ 29. HY(5.1),^807^ 30. FCC,^985^ 31. MoO_3_/H-ZSM-5,^989^ 32. Hβ,^989^ 33. NaOH-treated ZSM-5,^917^ 34. Mo_2_C/H-ZSM-5,^989^ 35. Fe_3_O_4_-SiO_2_/H-ZSM-5,^990^ 36. NaOH-treated H-ZSM-5,^864^ 37. H-ZSM-5,^992^ 38. Re-Y,^824^ 39. H-ZSM-5(25),^824^ and 40. Re-Y/H-ZSM-5(25)(2:1)^824^ catalysts.

The molecular composition of the lignocellulosic biomass drastically affects the catalytic pyrolysis performance and the resulting BTX yield and distribution. Cellulose, hemicellulose, and lignin have been separately subjected to catalytic fast pyrolysis using various types of catalysts, of which the quantified BTX yields are summarized in Figure 31. The highest BTX yields have been reported for cellulose and lignin are about 25 C% (15 wt%). Compared to cellulose and lignin, hemicellulose shows lower BTX carbon yields, and all are below 15 C% (Figure 31B). A few studies have shown that cellulose–hemicellulose and cellulose–lignin interactions have a positive effect on BTX yields. For instance, Mihalcik et al. reported that the BTX yields for the catalytic co-pyrolysis of cellulose and lignin was 9.0 wt%, which is higher that calculated value (8.2 wt%) according to the co-feeding mass ratio (1:1) and the individual BTX yields (9.5 wt% for cellulose alone and 6.3 wt% for lignin alone).^890^

### 4.3 Heterogeneous Catalysts

A fundamental understanding of the reaction network and the action of the catalyst in the various reactions is key to rationally design of effective catalysts for the conversion of lignocellulosic biomass to BTX using the catalytic pyrolysis approach. Numerous catalysts have been investigated, such as metal oxides (e.g., CaO, Al_2_O_3_, and La_2_O_3_), zeolites (e.g., MFI, FER, FAU, BEA, MOR, ZSM-11, ZSM-23, MCM-22), metal-doped zeolites (e.g., Ce-, Fe-, Cu-, Ni-, Co-, Mo-, Pt-, and Na-), inorganic salt additives, and carbon-based catalysts.^864,865,874,884,890,893,908,914−930^ Among all these catalysts, zeolites show a good performance in deoxygenation efficiency and aromatic hydrocarbon production. This is associated with catalyst characteristics such as specific surface area, acidity, and microporosity, which control the reactions leading to the formation of aromatics, including dehydration, decarboxylation, cracking, C–C bond formation, isomerization, and dehydrogenation.^21,22,993−995^

#### 4.3.1 Zeolites

Zeolite-based catalysts are of great interest for catalytic pyrolysis of biomass due to their unique porous structure, shape selectivity, and tuneable acidity.^910,933^ Particularly their tuneable acidity (both the Brønsted and Lewis acidity) is a key feature of zeolites that can significantly determine their catalytic activity and selectivity in biomass conversions.^996−998^ Brønsted acid sites can be formed via the substitution of Si^4+^ by Al^3+^ in the tetrahedral framework of the zeolite.^999,1000^ The incorporation of Al^3+^ into the zeolite framework produces a negative charge, that needs to be compensated by cations to maintain the charge balance of the system.^933,1000^ Acid sites have high importance for example in cracking processes, where high acidity promotes the formation of lighter olefins.^910,933,934^ When using zeolites with high pore surface areas, the cracking, aromatization and isomerization rates are accelerated leading to higher amounts of the desired hydrocarbons. It is also known that the pore size affects the aromatic yield. For instance, small pore zeolites do not produce any aromatics (but more coke, CO, and CO_2_), whereas medium pore zeolites like ZSM-5 and ZSM-11 (with pore sizes in the range of 5.2–5.9 Å) give higher aromatic yields. Large pores have a negative effect and these zeolites promote the formation of coke, and only minor amounts of aromatics are formed.^21,910,933,934,1001^ Due to the kinetic diameters of the products and reactants, the main part of the aromatic products and the starting materials can fit inside the pores of medium and large pore zeolites (aromatics can form directly or via secondary reactions to smaller aromatics).^910,934^

#### 4.3.2 H-ZSM-5 and Modified H-ZSM-5 Catalysts

**Figure 32:**
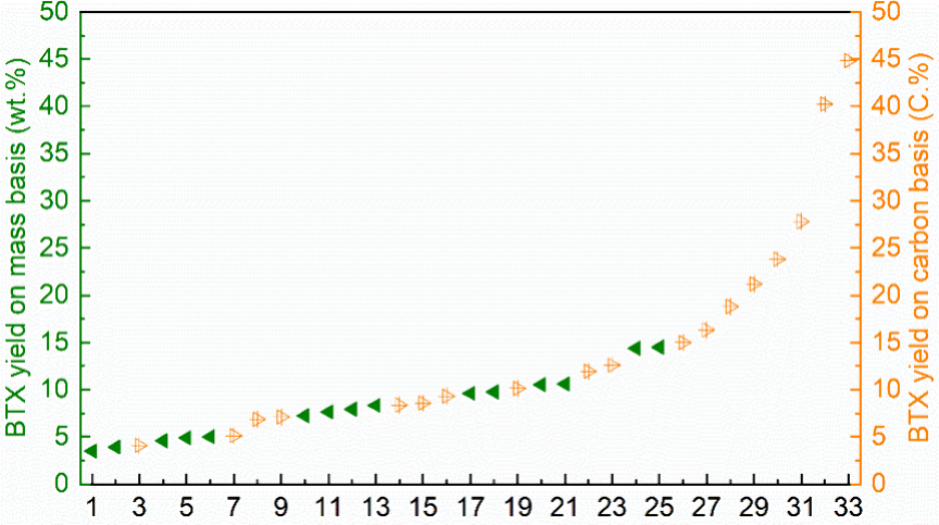
Quantified mass and carbon yields of BTX over non-modified H-ZSM-5 catalysts for the catalytic pyrolysis of various lignocellulosic biomass sources, including 1. Palm kernel shell,^972^ 2. Miscanthus *x* ganteus,^955^ 3. Citrus unshiu peel,^949^ 4. Oak,^919^ 5. Quercus,^876^ 6. Rice straw,^969^ 7. Hybrid poplar,^889^ 8. Wheat straw,^966^ 9. Switchgrass,^889^ 10. Pine,^953^ 11. HemiCellulose,^890^ 12. Wood chips,^967^ 13. Switchgrass,^890^ 14. Fagus sylvatica,^952^ 15. Pinyon-juniper,^889^ 16. Miscanthus *x* ganteus,^891^ 17. Corn cob,^890^ 18. Corn stover,^890^ 19. Eucalyptus trunks,^923^ 20. Yellow poplar (torrefied),^968^ 21. Cellulose,^987^ 22. Rice stalk,^980^ 23. Hemicellulose,^989^ 24. Cedar,^888^ 25. Lignin,^992^ 26. Grindelia squarrosa,^892^ 27. Corn fermentation residues,^964^ 28. Red oak,^917^ 29. Sugarcane bagasse,^948^ 30. Lignin,^824^ 31. Pine,^825^ 32. Corn stover,^947^ and 33. Cellulose.^977^

From the zeolite family, ZSM-5 (MFI-type zeolite) is the most efficient zeolite for producing aromatic hydrocarbons in general and BTX in particular,^867,869,900−906^ rationalized by considering pore characteristics (regular), acidity (tunable), and high thermal and hydrothermal stability.^1002−1004^ During catalytic pyrolysis, the structure of the zeolite may change by thermal distortion. For instance, it has been shown that pore enlargement by 2.5–3.4 Å occurs,^1005^ which may affect the BTX yield and selectivity by allowing molecules to enter the pores.^910,1005^ The quantified mass and carbon yields of BTX for the catalytic pyrolysis of various lignocellulosic biomasses and the three main constituents (cellulose, hemicellulose, and lignin) over non-modified H-ZSM-5 catalysts are shown in Figure 32. The BTX yield is typically between 10 and 20 C% (5–10 wt%), and these values represent the state-of-the-art performance of the catalytic pyrolysis of lignocellulosic biomass over a non-modified ZSM-5 zeolite. For exceptional cases, carbon yields of 45 C% have been reported (cellulose only, Figure 32, Entry 33).

**Figure 33:**
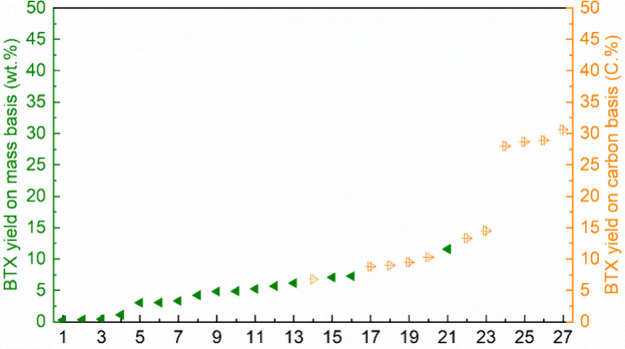
Quantified mass and carbon yields of BTX from catalytic pyrolysis of lignocellulosic biomass over metal-modified H-ZSM-5 catalysts by using various metals, including 1. Fe- (Poplar sawdust),^971^ 2. Fe-Ni- (Poplar sawdust),^971^ 3. Ni- (Poplar sawdust),^971^ 4. Zn- (Bamboo residues),^898^ 5. Ga- (Radiata pine),^937^ 6. Ni- (Poplar sawdust, regenerated),^971^ 7. Ni- (Poplar sawdust, regenerated),^971^ 8. Ni- (Quercus mongolica),^876^ 9. Fe (Bamboo residues),^898^ 10. Fe-Ni- (Poplar sawdust, regenerated),^971^ 11. Fe-Ni- (Poplar sawdust),^971^ 12. Ni- (Rice straw),^969^ 13. Fe-Zn- (Bamboo residues),^898^ 14. Ni- (Eucalyptus trunks),^923^ 15. Ga- (Quercus mongolica),^876^ 16. Ni- (Wood chips),^967^ 17. Ga- (Eucalyptus trunks),^923^ 18. Ni-(Miscanthus *x* ganteus),^891^ 19. Zn- (Eucalyptus trunks),^923^ 20. Ga- (Eucalyptus trunks),^923^ 21. Ni- (Wood chips),^967^ 22. Ga- (Pine wood),^1007^ 23. P-Ni- (Pine wood),^927^ 24. Ni- (Pine wood),^865^ 25. Co- (Pine wood),^865^ 26. Mo- (Pine wood),^865^ and 27 Pt- (Pine wood).^865^

Metal doping of zeolites, mainly via ion-exchange (complete/proportional) or impregnation),^1006^ is a straightforward way to improve catalytic performance of H-ZSM-5. The quantified mass and carbon yields of BTX for the catalytic pyrolysis of lignocellulosic biomass over metal-modified H-ZSM-5 catalysts by using various metals such as Co,^864,865^ Ni,^865,891,923,967,969,971^ Mo,^865^ Pt,^865^ Fe,^874,898,971^ Na,^884^ Ni,^874,876,971^ Zn,^898^ Ga,^876,923,937,1007^ are shown in Figure 33.

Typically, metal-modified zeolites facilitate the formation of single- and double-ring aromatics compared to non-modified H-ZSM-5 and the highest BTX yield of 30.6 C% was obtained on the Pt-modified ZSM-5 catalyst for catalytic pyrolysis of pine wood (Figure 33, entry 27). The incorporation of metals affects the BTX yield and selectivity for instance by (i) additional catalytic effects, (ii) changes in the pore volume/size (depending on metal loading) and specific surface area, and (iii) the acidity of the catalyst (both Brønsted and Lewis acidity). Furthermore, it was observed that metal-modification plays a role in slowing down the rate of coke formation (via favoring oxygen removal reactions in different forms such as decarboxylation and decarbonylation) and consequently reduce the extent of catalyst deactivation (vide infra). The amount of the metal typically needs to be tailored to the biomass feed, and a higher metal loading does not necessarily lead to improved catalyst performance.^973^

**Figure 34:**
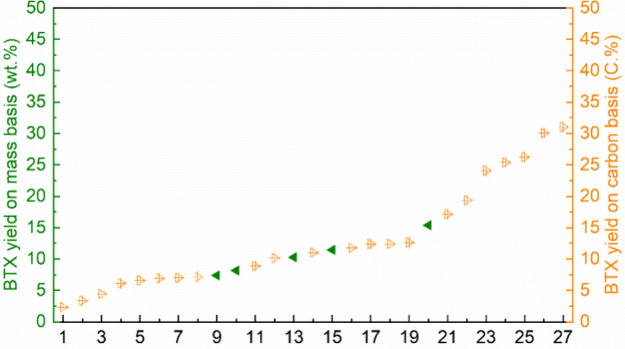
Quantified mass and carbon yields of BTX from catalytic pyrolysis of lignocellulosic biomass over mixed H-ZSM-5 catalysts, including 1. H-ZSM-5/Al_2_O_3_(90/10) (Pine),^1009^ 2. MoO_3_/H-ZSM-5 (Lignin),^989^ 3. H-ZSM-5/Al_2_O_3_(90/10) (Pine),^1009^ 4. Mo_2_C/H-ZSM-5 (Lignin),^989^ 5. ZSM-5/MCM-41(85/15) (Cellulose),^988^ 6. Fe_3_O_4_-SiO_2_/H-ZSM-5 (Lignin),^990^ 7. MoO/H-ZSM-5 (Hemicellulose),^989^ 8. ZSM-5/CaO(85/15) (Cellulose),^988^ 9. Mo_2_N/H-ZSM-5 (Pine),^1008^ 10. Ce-Mo_2_N-H-ZSM-5 (Pine),^954^ 11. H-ZSM-5/Al_2_O_3_(80/20) (Pine),^1009^ 12. NiO/ZSM-5 (Miscanthus x ganteus),^891^ 13. dual red mud and H-ZSM-5(50) (Rape straw),^957^ 14. H-ZSM-5/SiO_2_(80/20) (Pine),^1009^ 15. dual CaO/red mud and H-ZSM-5(50) (Rape straw),^896^ 16. MoO/H-ZSM-5 (Cellulose),^989^ 17. H-ZSM-5/clay(88/12) (Pine),^1009^ 18. Fe_3_O_4_/SiO_2_/H-ZSM-5 (Sawdust),^990^ 19. Mo_2_C/H-ZSM-5 (Hemicellulose),^989^ 20. H-ZSM-5/HY(70/30) (Cellulose),^987^ 21. Fe_3_O_4_/SiO_2_/H-ZSM-5 (Cellulose),^990^ 22. Mo_2_C/H-ZSM-5 (Cellulose),^989^ 23. SiO_2_/H-ZSM-5 (Pine wood sawdust),^825^ 24. Re-Y/H-ZSM-5(25)(2:1) (Lignin),^824^ 25. Pt/H-ZSM-5 (Cellulose),^991^ 26. Ga_2_O_3_/H-ZSM-5 (Pine wood sawdust),^825^ and 27. Ga_2_O_3_/SiO_2_/H-ZSM-5 (Pine wood sawdust).^825^

Besides the above mentioned zeolite modification by the introduction of metals, many studies have used H-ZSM-5-supported metal catalysts (e.g., MoO_3_/H-ZSM-5,^989^ Mo_2_C/H-ZSM-5,^989^ Ce-Mo_2_N-H-ZSM-5,^954^ Mo_2_N/H-ZSM-5 (Pine),^1008^ NiO_5_/ZSM-5,^891^ Ga_2_O_3_/H-ZSM-5^825^), H-ZSM-5 combined with binders (e.g., H-ZSM-5/Al_2_O_3_,^1009^ ZSM-5/CaO,^988^ H-ZSM-5/SiO_2_,^1009^ and H-ZSM-5/clay,^1009^ and H-ZSM-5/HY^987^), and two catalysts in sequential beds (e.g., red mud and H-ZSM-5^957^ and CaO/red mud and H-ZSM-5^896^) for catalytic pyrolysis of various lignocellulosic biomass and the quantified mass and carbon yields of BTX are shown in Figure 34. The highest BTX carbon yield of 31.1 C% was obtained for the catalytic pyrolysis of pine wood sawdust using a Ga_2_O_3_/SiO_2_/H-ZSM-5 catalyst (Figure 34, entry 27).^825^ These hybrid catalysts also affect the aromatics selectivity. For instance, Lu et al. observed that the amount of PAHs significantly decreased while the amount of monocyclic aromatics was increased when using a Mo_2_N/H-ZSM-5 catalyst (Figure 34, entry 9) compared to an un-modified H-ZSM-5.^1008^

#### 4.3.3 Catalyst Deactivation and Regeneration

**Figure 35:**
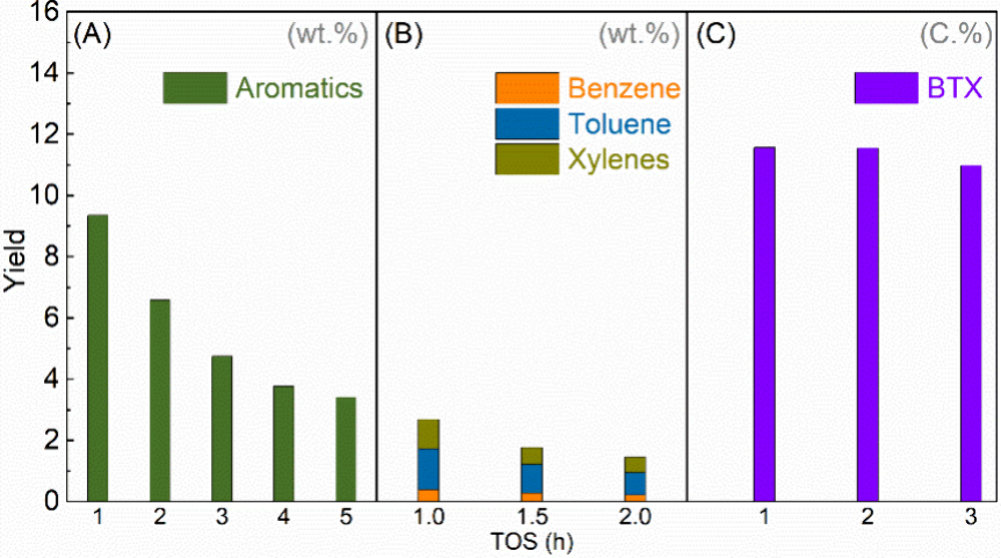
BTX yields versus time on stream for catalytic pyrolysis of rice stalk over ZSM-5^980^ (A), poplar over ZSM-5^951^ (B), and palm kernel shell over Fe/Hβ^972^ (C).

Coking and associated catalyst deactivation are typically observed in catalytic pyrolysis, and directly affect the catalyst life-time, as well as the selectivity of the reaction. Zhang et al. studied the catalytic pyrolysis of rice stalk in an internally interconnected fluidized-bed reactor using ZSM-5 type catalysts, and showed that the BTX yields decreased rapidly from 9.4 wt% at 1 h TOS to 4.7 wt% at a TOS of 3 h (Figure 35A).^980^ This rapid catalyst deactivation was also reported by Du et al., showing that BTX yields at a TOS of 2 h was halve of that at a TOS of 1 h (Figure 35B, poplar, H-ZSM-5).^951^ It was found that the degree of coking is connected to the topology and the acidity of the zeolite.^890,112,1010^

Due to rapid coking, zeolites must be frequently regenerated to maintain their catalytic activity. However, the regenerated catalyst often showed a lower BTX yield compared to the fresh catalyst. For example, a recent study by Li et al. on the catalytic pyrolysis of poplar showed that the BTX yields of a regenerated Ni/ZSM-5 (3.1 wt%) catalyst was only 8% of that of the fresh one. Similar results were found for the Fe-Ni/ZSM-5 catalyst.^971^ Irreversible catalyst deactivation is also observed; however, it has not been studied in detail yet.

### 4.4 Remarks and Outlook

Catalytic pyrolysis is a promising technology for converting lignocellulosic biomass to bio-based aromatics in general and BTX in particular. Significant progress has been made regarding (i) catalyst selection, (ii) selection of suitable lignocellulosic biomass sources, and (iii) the effects of co-feeds on the BTX yield and selectivity. Reaction parameters (e.g., temperatures of pyrolysis and catalytic vapor upgrading, weight hourly space velocity, and catalyst to biomass ratio), catalysts (e.g., types, modifications with metals and binders), and catalyst characteristics (e.g., surface area, acidity, and microporosity) have been widely investigated. The BTX yields for lignocellulosic biomass (alone) are typically lower than 20 C% (10 wt%) on feed. There are a few exceptions giving higher BTX yields, e.g., a value of 40.2 C% was reported for the catalytic pyrolysis of corn stover. Co-feeding biomass with, e.g., plastics or alcohols, shows synergistic effects and leads to enhanced BTX yields. The highest BTX yield (82.1 C%) was obtained for the catalytic co-pyrolysis of corn stover and fusel alcohol (1 to 1 mass ratio). Catalytic pyrolysis of lignocellulosic biomass seems economically feasible, particularly when green premium, carbon price, and CO_2_ emission factor are considered. Co-feeding the lignocellulosic biomass with cheap co-feeds, such as plastic waste, can significantly enhance the economic feasibility. This is currently applied by the refineries such as BioBTX B.V. and Anellotech Inc.

One of the major challenges for catalytic pyrolysis technology is the observation of fast catalyst deactivation due to coke formation. This requires the development of efficient catalyst regeneration protocols. Irreversible catalyst deactivation has also been reported but requires further quantification. Particularly the latter will strongly affect attainable catalyst turnover numbers (kg product per kg catalyst) and thus will have a major impact on the technoeconomic feasibility of the concept. In addition, rational reactor design and selection activities will be required to optimise the process. Inspiration for the latter may be obtained by considering existing commercially operated hydrocarbon processes using rapidly deactivating zeolite catalyst (e.g., FCC units).

## 5 Aromatics from Aldehydes

Aldehydes can be converted to aromatics in various ways. One bio-based aldehyde is acetaldehyde produced from the dehydrogenation of ethanol, which is a process that has been explored extensively.^1011^ This reaction plays an important role in both ethanol aromatization and acetaldehyde aromatization. Moreover, propanal can be obtained by hydrogenolysis of glycerol in up to 50% yields.^1012^

The conversion of acetaldehyde over ZSM-5 was initially described to proceed with low reactivities at 400 °C producing mainly olefins and only low single-digit selectivities to aromatics.^107^ The self-condensation of acetaldehyde can be significantly improved by the selection of a suitable catalyst. For example, Moteki et al. found a noteworthy increase of the yields in the reaction of acetaldehyde over calcium hydroxyapatite, particularly leading to the formation of methyl benzaldehydes. They used mixtures of ethanol (which gives acetaldehyde by dehydrogenation) with acetaldehyde (0.35 kPa C_2_H_4_O, 1 kPa C_2_H_5_OH, 100 kPa H_2_, 548 K) to form mainly 2-methylbenzaldehyde (S = 27%) in addition to 4-methylbenzaldehyde (S = 3%) at an acetaldehyde conversion of 55%.^1013^

Zhang et al. found that the conversion of acetaldehyde in ethanol (180 °C, ethanol:acetaldehyde = 10:1) using a magnesium oxide-catalyst gave the most promising yields (overall yield = 48.4%) of C_8_ compounds (2,4,6-octatrienal and tolualdehyde with S = 25.2%), although crotonaldehyde (*S* = 48%) was the main product. However, the crotonaldehyde can still be converted to C8 in a subsequent reaction, where the sweet point is at a temperature between 180 °C (X = 46.7%, *S*_C8_ = 73.9%) and 220 °C (X = 63%, S_C8_ = 36%).^1014^ An earlier study on the cyclodimerization of crotonaldehyde over solid acidic and basic catalysts already demonstrated the particular suitability of magnesia, but also of aluminum oxide and calcium oxide, to form the desired tolualdehydes or their precursors. For example, 19.2% yield of 6-methylcyclohexa-1,3-dienecarbaldehyde plus *o*-tolualdehyde were found using CaO at 350 °C in a pulse reactor.^1015^ The former compound can be converted into the latter by dehydrogenation.

**Scheme 161:**
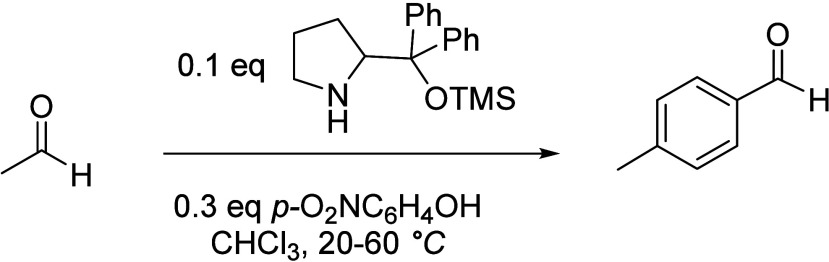
*p-*Methylbenzaldehyde from Acetaldehyde

However, the previous conversions of acetaldehyde are characterized by a complex reaction network that can release a variety of products. Li and co-workers therefore improved upon the previous heterogeneous-catalytic conversions by directing the condensation to aromatics with a homogeneous-catalytic approach. The synthesis of *p*-methylbenzaldehyde (*p*-MBA) from acetaldehyde can be carried out very selectively using diphenyl prolinol trimethylsilyl ether and *p*-nitrophenol as catalyst (Scheme 161). Selectivities for p-MBA of up to 90% (HPLC) with an acetaldehyde conversion of 99.8% have been reported.^1016^ A mechanism via enamine-iminium intermediates was proposed based on isotope labeling experiments. The condensation reactions lead to formation of the methyl-dihydro-benzaldehyde and the authors assume that the subsequent dehydrogenation is catalyzed by the base since the reaction takes place under argon. However, this is very unlikely at these low temperatures (20–60 °C). In addition, the authors were not able to prove the presence of hydrogen in the gas phase. It is more likely that the nitro group of the nitrophenol acts as the oxidant, or alternatively, the dihydrobenzene is oxidized by air during the work up or during the analysis.

**Scheme 162:**
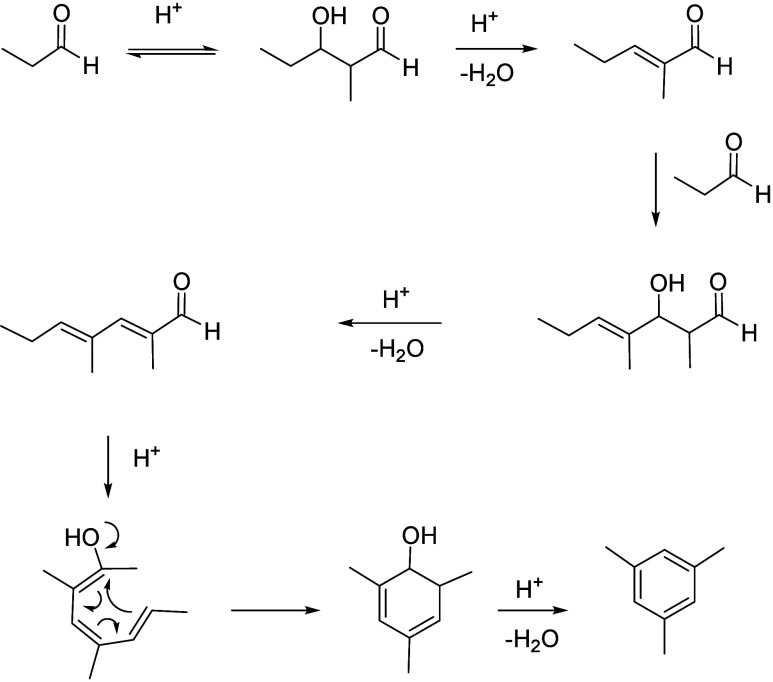
Aromatization of Propanal: Proposed Mechanism Leading to Trimethylbenzene via Aldol Condensation of Propanal^1017^

It was assumed that for aromatics formation, the aldehydes must first be deoxygenated and converted to an olefin before they can then react further to aromatics, as in the case of the conversion of saturated hydrocarbons. However, if one compares the reactivities of propylene and propanal, one comes to the conclusion that this is not necessarily the case. In a pivotal work in this field, Mallinson and co-workers^1017^ found that the conversion rates over H-ZSM-5 zeolites of propanal are significantly higher than those of propylene; thus, these two materials are probably converted via different pathways. In addition, a different aromatic product distribution was found (C_9_ aromatics). The authors concluded that the main pathway for aromatisation of aldehydes does not pass via a hydrocarbon pool as in the case of olefins but via an oxygenate pool. Using the formation of trimethylbenzene, the authors proposed a reaction chain that starts off with aldol condensation reactions (Scheme 162). Nevertheless, the authors did not exclude a contribution of a hydrocarbon pool.

Lin et al. attempted to elucidate the basic mechanistic steps of propanal deoxygenation at the Brønstedt acidic sites of the MFI zeolite. During their study they found that by increasing the reaction temperature from 473 to 548 K, the aromatic products increased from 13.1% to 51.4.^1018^ Regarding stability, smaller sized zeolites (0.2–0.5 μm vs 2–5 μm) show less deactivation due to reduced diffusion pathways and there can even be a significant increase in selectivity to C9 aromatics (0.88 vs 0.22 of the C9/(C8+C7) ratio at a conversion of about 80%).^1019^ Further developments in catalyst structuring followed. These include the development of Al-MFI nanosheets with Si/Al = 26, which are lamellar, ∼2 nm in (101) direction and 50–100 nm in (100) and (001) direction. Use of these catalysts resulted in a total aromatics yield of 58% and 24% for C9 trimethylbenzenes, respectively, at full conversion of propanal.^1020^ In addition, the Resasco team, with the participation of Mallinson, found that tailoring the zeolites with respect to the pore structure, and in particular the pore size, led to a significant increase in the yields of trimethylbenzenes.^1019^ Using the zeolite H-ZSM-5 (Si/Al = 76), the authors were able to achieve 12.8% m-xylene at T = 400 °C and W/F = 0.5 h (W/F = weight/flow ratio) with a conversion of 90% (aromatic content in the product spectrum of 40.2%). However, the desilylation used for the mesostructure formation must be carried out in a mild fashion (Si/Al = 59) to obtain a yield of aromatics of up to 42.2% at 90% conversion, since progressive destructuring of the zeolite lowers the microporosity and thus the reactivity. However, the increase in selectivity is only minor.

In conclusion, also based on the previous chapters, it becomes clear that for the aromatization of shorter-chain molecules, such as olefins, and here aldehydes, using high temperature methods, the desired products are often obtained at low selectivity. Indeed, a base-catalyzed low temperature condensation of propanal gave *p*-tolualdehyde in 90% selectivity. Here it is worth changing the perspective and asking what mixtures of aromatics will play a major role for in the future. For example, Yeboah et al. presented the idea of producing jet fuel aromatics in a targeted manner. They used tandem catalysis in which the catalyst bed upstream consisting of Cu/SiO_2_-TiO_2_ promotes propanal condensation and a second catalyst bed downstream consisting of Ni/H-ZSM-5 promotes sequential hydrodeoxygenation and aromatization to the desired fraction at 300 °C and atmospheric pressure.^1021,1022^ Carbon loss was minimal due to the formation of water for oxygen removal, so that a maximum of 86.1% jet fuel hydrocarbons with an aromatics content of 81.5% could be produced.

## 6 Aromatics from Fats and Oils

### 6.1 From Volatile Fatty Acids

In this section, the conversion of volatile fatty acids (acetic, propionic and butyric) to benzenoid aromatics will be discussed. Examples, where the yield of aromatics is below 20% were ignored.

Anaerobic fermentation of organic material leads to the formation of short-chain, volatile fatty acids (VFAs). These are normally further converted to methane and CO_2_ but by manipulation of the pH the process can be halted at the stage of the VFAs. Usually acids with C2–C6 are included in the definition of VFAs, although in most fermentations acetic, propionic and butyric acid are the major constituents. A tremendous amount of research has been performed on valorizing waste streams such as municipal solid or liquid waste, but also agro and food waste to obtain VFAs in view of the fact that most of these acids are already used industrially or can be converted further to useful products.^1023^ Conversion of VFA’s to aromatics is one possibility. Acetic acid can also be obtained via two-stage fermentation via ethanol.

Gayubo and co-workers studied the conversion of acetic acid in the presence of H-ZSM-5 as catalyst.^107^ The highest yield of aromatic compounds was achieved, when the reaction was initiated at a temperature of 250 °C, which was increased to 450 °C at a rate of 1 °C min^–1^. After reaching this temperature it was kept constant for a prolonged period. After 4 h the aromatic compounds started to form, and after a total of 6 h the yield of aromatics in the mixture reached 42% (Table 36, entry 1). Unfortunately, the authors did not provide any details on the product distribution. The group of Brown used H-ZSM-5 as catalyst for the pyrolysis of acetic acid at 600 °C.^23^ Total yield of benzenoid compounds corresponded to 27% (Table 36, entry 2). The main aromatic product was toluene (41% of the aromatic fraction) followed by xylene (28%). The group of Zhu reported the conversion of propionic acid over H-ZSM-5 in a continuous flow reactor.^1024^ After testing different WHSVs, the authors found that very high yields of aromatic compounds at complete conversion of propionic acid were achieved at WHSV = 3.37 h^–1^ (Table 36, entry 3). The major product is toluene (12% of the total yield). Triantafyllidis, Heracleous and co-workers reported the use of ZSM-5 for the ketonization of acetic acid to acetone and its further conversion to aromatics.^1025^ The selectivity toward aromatic compounds was 27% at 74% conversion of the substrate (Table 36, entry 4). The major products were xylenes and 2,5-dimethylphenol. Bruijnincx and co-workers studied H-ZSM-5, Ga-doped H-ZSM-5, TiO_2_ P25 and mixtures of P25 with the zeolite and Ga-doped zeolite for the conversion of butyric acid to valuable compounds.^136^ Ga-doped H-ZSM-5 and especially P25 + Ga/H-ZSM-5 allowed the formation of aromatic compounds, where the major components of the mixture were BTX (Table 36, entries 5 and 6). In both cases the major aromatic product was toluene.

**Table 36:** Conversion of Volatile Fatty Acids to Benzenoid Aromatics

Entry	Fatty acid	Catalyst	Conditions	*T* (°C)	Yield of benzenoid aromatic compounds (%)	Products	Ref
1	Acetic acid	H-ZSM-5	Continuous flow, fed with 50 wt% of H_2_O, TOS = 6 h	450	42^a^	No details provided	(107)
2	Acetic acid	H-ZSM-5	Gas-phase acetic acid, cat:AcOH = 20:1	600	27^a^	BTX, C_9+_ aromatics	(23)
3	Propionic acid	H-ZSM-5	Continuous flow, W/F = 3.37 h^–1^	350	86^a^	Benzene, alkylated benzenes and bicyclic hydrocarbons	(1024)
4	Acetic acid	ZSM-5	Continuous flow, WHSV = 48 h^–1^	500	20^a^	Phenols + other aromatics	(1025)
5	Butyric acid	Ga/H-ZSM-5	Continuous flow, WHSV = 1 h^–1^, TOS = 98 min	450	23^b^	BTX, ethyl-benzene and C_9_+ aromatics	(136)
6	Butyric acid	P25 + Ga/H-ZSM-5	Continuous flow, WHSV = 1 h^–1^, TOS = 49 min	450	50^b^	BTX, ethyl-benzene and C_9_+ aromatics	(136)

a Analysis by GC without use of internal standard.

b Analysis by GC with cyclooctane as an internal standard.

Many literature reports claim formation of aromatic compounds as side products during ketonization of these short-chain carboxylic acids. However, many of those do not report detailed data on the actual content of these aromatic compounds.

### 6.2 From Long-Chain Fatty Acids

Fatty acids are available via hydrolysis of fats and oils. These are current large-scale processes.^1026^

The group of Savage reported the conversion of palmitic acid in the presence of H-ZSM-5 as catalyst to toluene, 2-ethyltoluene, propylbenzene, xylene, and 1,2,4-trimethylbenzene.^1027^ The best results were achieved, when the reaction was run under 25 bar of hydrogen gas (Table 37, entry 1), where xylenes where the major products. The same group later studied the conversion of linoleic, oleic and stearic acids over ZSM-5 with a Si:Al ratio of 30 (Table 37, entries 2–4).^1028^ The best yield of aromatic compounds was achieved when stearic acid was used as the substrate (Table 37, entry 4), although the reported standard error was fairly high. The same alkylated benzenes were formed as in the previous case, and the major products in all cases were the xylenes. The authors reported the conversion of palmitic acid to aromatics in the presence of H-ZSM-5 catalyst under the same reaction conditions.^1029^ Aromatic compounds were formed in 42% yield (Table 37, entry 5). Li and co-workers used MZM-5-B mesoporous zeolite for the conversion of oleic acid to hydrocarbons.^1030^ While the liquid phase consisted of 97% of aromatic hydrocarbons (with *p*-xylene being the most abundant product, entry 6), the authors did not provide any details of actual yields of the liquid fraction. Zheng et al. studied Ni-Cu catalysts on different supports for catalytic pyrolysis of oleic acid.^1031^ Initially the supports were tested without copper and nickel. The results of those experiments, which led to the formation of more than 20% of aromatics are shown in Table 37, entries 7–10. H-ZSM-5 itself provided 65% yield of aromatics, and using the same catalyst as a support with Ni and Cu metals did not change the outcome. However, while Hβ and HUSY were not effective catalysts for the formation of aromatics from oleic acid, the use of these as supports for Ni-Cu catalysts allowed them to be effective in the desired transformation with aromatics yields of 74% and 59%, respectively. The formed products are benzene, alkylated benzenes and naphthalene. The group of Heeres reported the conversion of oleic acid in the presence of H-ZSM-5/Al_2_O_3_ catalyst.^1032^ The highest yield of 27% (22% BTX) was achieved after a time on stream of 1.5 h (Table 37, entry 11). The major product under these conditions is toluene. The authors have also demonstrated that the catalyst is active after regeneration. After the first cycle the BTX yield actually increased to 25%. However, further regeneration resulted in gradual reduction of the yield of BTX.

**Table 37:**
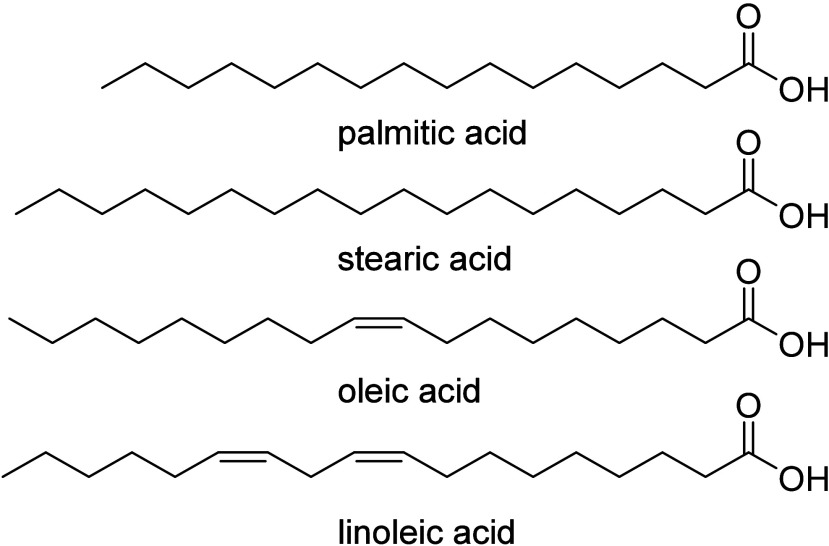
Conversion of Fatty Acids to Aromatic Compounds

Entry	Fatty acid	Catalyst (Si/Al ratio)	Conditions	*T* (°C)	Yield of benzenoid aromatic compounds (%)	Products	Ref
1	Palmitic acid	H-ZSM-5	Catalyst:substrate as 1:1 wt; water, 3 h, 25 bar H_2_	400	61 ± 9^a^	Alkylated benzenes	(1027)
2	Linoleic acid	ZSM-5(30)	Catalyst:substrate as 1:1 wt; water, 3 h	400	35 ± 12^a^	Alkylated benzenes	(1028)
3	Oleic acid	ZSM-5(30)	Catalyst:substrate as 1:1 wt; water, 3 h	400	63 ± 5^a^	Alkylated benzenes	(1028)
4	Stearic acid	ZSM-5(30)	Catalyst:substrate as 1:1 wt; water, 3 h	400	80 ± 33^a^	Alkylated benzenes	(1028)
5	Palmitic acid	H-ZSM-5	Catalyst:substrate as 1:1 wt; water, 3 h	400	42 ± 4^a^	Alkylated benzenes	(1029)
6	Oleic acid	MZM-5-B	Continuous flow, WHSV = 4.5 h^–1^	500	97% of the liquid fraction^b^	(alkylated) benzenes, indenes and naphthalenes	(1030)
7	Oleic acid	H-ZSM-5	Continuous flow, 1.2 g of catalyst, 0.25 mL min^–1^ of oleic acid, 30 min	500	65^a^	Alkylated benzenes	(1031)
8	Oleic acid	Ni-Cu/H-ZSM-5		500	65^a^	Alkylated benzenes	(1031)
9	Oleic acid	Ni-Cu/Hβ		500	74^a^	Alkylated benzenes	(1031)
10	Oleic acid	Ni-Cu/HUSY		500	59^a^	Alkylated benzenes	(1031)
11	Oleic acid	H-ZSM-5/Al_2_O_3_	Continuous flow, WHSV = 1 h^–1^, TOS = 1.5 h	550	27^c^	BTX, ethylbenzene, (alkylated)naphthalenes	(1032)

a Analysis by GC without use of internal standard.

b Yield of the liquid fraction was not reported.

c Analysis by GC with *n*-nonane an internal standard.

### 6.3 From Fats and Oils

The results of the catalytic conversion of fats and oils to aromatics are summarized in Table 38. In 1986 the group of Bakhshi reported the conversion of canola oil and mustard oil to hydrocarbons in the presence of H-ZSM-5 catalysts, which were subjected to steam treatment at different temperatures.^1033^ The best performing catalyst for the conversion of both oils was fresh non-treated by steam zeolite H-ZSM-5 (Table 38, entries 1 and 2). Unfortunately, no details on the exact aromatic products distributions were given. Later the same group reported the conversion of tall oil in the presence of H-ZSM-5 as catalyst.^1034^ The authors ran the reaction in the presence and absence of steam. The best result for the formation of aromatic compounds was achieved without any steam (Table 38, entry 3). The major aromatic product formed under these conditions is toluene, followed by benzene. This group also studied the conversion of canola oil over H-ZSM-5 as catalyst in a continuous flow at 500 °C.^1035^ The obtained products contained 23% of BTX (Table 38, entry 4). No further details on the exact portion of each aromatic compound were provided. The group of Bhatia reported the use of H-ZSM-5 and MCM-41 catalysts and their composites in the conversion of palm oil-based fatty acids residue.^1036^ The best results (27% aromatics yield, with a ratio of benzene:toluene:xylenes of 1:3.8:3.7) were achieved with a CMZ20 catalyst (Table 38, entry 5). In 2011, Hilten et al. reported the conversion of acidified peanut oil soap stock (an impure mixture of fatty acid sodium salts in water) in the presence of H-ZSM-5 as catalyst, where up to 33% yield of aromatic compounds was achieved (Table 38, entry 6).^1037^ The major component in the mixture was *m*-xylene. Bayat and Sadrameli also studied the conversion of canola oil and its methyl ester (the “ester” is the product of the reaction of the oil with methanol catalyzed by KOH).^1038^ In the presence of H-ZSM-5(50) as catalyst, canola oil was converted to 31% of aromatics (Table 38, entry 7) consisting of benzene (3.76%), toluene (11.51%), *p*-*m*-xylene (8.03%), *o*-xylene (2.46%), and C9-aromatics (5.31%). An even higher yield of 35% with a product composition of: benzene (3.48%), toluene (11.77%), *p*- and *m*-xylene (9.82%), *o*-xylene (3.24%), and C9-aromatics (6.53%) was achieved when the methyl ester of canola oil was subjected to the same reaction conditions (Table 38, entry 8).

**Table 38:** Conversion of Fats and Oils to Aromatic Compounds

Entry	Fat or oil used	Catalyst used (Si/Al ratio)	Conditions	*T* (°C)	Yield of benzenoid aromatic compounds (%)	Products	Ref
1	Canola oil	H-ZSM-5	Continuous flow, WHSV = 3.0 h^–1^, TOS = 1 h	370	37^a^	Hydrocarbons	(1033)
2	Mustard oil	H-ZSM-5	Continuous flow, WHSV = 3.0 h^–1^, TOS = 1 h	370	47^a^	Hydrocarbons	(1033)
3	Tall oil	H-ZSM-5	Continuous flow, WHSV = 3.6 h^–1^	405	45^b^	Benzene and alkylated benzenes	(1034)
4	Canola oil	H-ZSM-5	Continuous flow, WHSV = 12.1 h^–1^	500	23^b^	BTX	(1035)
5	Palm oil-based fatty acids residue	CMZ20	Continuous flow, WHSV = 2.5 h^–1^	450	27^b^	BTX	(1036)
6	Peanut oil soap stock	H-ZSM-5	Continuous flow, WHSV = 5.4 h^–1^	500	33	BTEX	(1037)
7	Canola oil	H-ZSM-5(50)	Continuous flow, WHSV = 2 h^–1^	450	31^b^	BTX and C9 aromatics	(1038)
8	Canola oil methyl ester	H-ZSM-5(50)	Continuous flow, WHSV = 2 h^–1^	450	35^b^	BTX and C9 aromatics	(1038)
9	Soybean oil	H-ZSM-5(50)	Catalyst:substrate as 1 g per 5.3 mL, H_2_ (14 bar), 1 h	430	28^c^	22% of monoaromatic compounds, 6% of polycyclic aromatics	(1039)
10	Rapeseed oil	Zn/H-ZSM-5	Continuous flow, WHSV = 7.6 h^–1^, 1 h	550	47^b^	C6-C13 aromatic hydrocarbons	(1040)
11	Coconut oil	H-ZSM-5	Catalyst:substrate as 1:1 wt; water, 3 h	400	31 ± 7^b^	Alkylated benzenes	(1029)
12	Peanut oil	H-ZSM-5	Catalyst:substrate as 1:1 wt; water, 3 h	400	52 ± 15^b^	Alkylated benzenes	(1029)
13	Lard	H-ZSM-5	Catalyst:substrate as 1:1 wt; water, 3 h	400	48 ± 4^b^	Alkylated benzenes	(1029)
14	Palm fatty acid distillate	5% Zn/ H-ZSM-5	Continuous flow, WHSV = 5 h^–1^	500	65^b^	BTEX	(1041)
15	Waste cooking oil	20 wt% 4AH-Z5	CDS Pyroprobe 5200, 20 seconds	600	58^b^	BTEX and naphthalene	(1042)
16	Rubber seed oil	Alkali treated ZSM-5	Continuous flow, WHSV = 5.48 h^–1^	550	>99% in the bio-oil phase^d^	BTX, other alkyl benzenes and polyaromatics	(1043)
17	Soybean oil	Si-MCM41	Continuous flow, WHSV = 6.69 h^–1^	460	25^b^	Mainly C_7_-C_13_ compounds	(1044)

a Unclear how the yield was measured.

b Analysis by GC without use of internal standard.

c Analysis by GC using 2-chlorotoluene as an internal standard.

d Yield of the liquid fraction was not reported.

Seames and co-workers reported the conversion of soybean oil at temperatures between 410 and 430 °C in the presence of H-ZSM-5 as the catalyst to a mixture of different compounds.^1039^ The selectivity toward monoaromatic compounds was up to 22%, and the combined yield of aromatic and polycyclic aromatic hydrocarbons did not exceed 28% yield (Table 38, entry 9). Unfortunately, no further details were provided on the exact yields of the separate aromatic compounds.

The group of Serrano studied the conversion of rapeseed oil in the presence of ZSM-5 catalysts modified with different metals.^1040^ The best yield of aromatic compounds was achieved with Zn-modified catalyst, where within the first hour the yield of aromatic compounds reached 47% (Table 38, entry 10). The aromatic hydrocarbons consist of different compounds containing 6–13 carbon atoms. No further details of the composition of these aromatic compounds were provided.

Savage and co-workers reported the conversions of coconut oil, peanut oil, and lard into benzenoids over ZSM-5 (Table 38, entries 11–13).^1029^ Using these starting materials they obtained alkylated benzenes, where xylenes were the major components in the aromatic fractions. Jongpatiwut and co-workers reported the conversion of palm fatty acid distillate (a waste stream containing mostly fatty acids) to aromatic compounds in the presence of H-ZSM-5 or doped versions of it with Ga or Zn.^1041^ The highest yield of benzenoids (65%) was achieved in the presence of 5% Zn/H-ZSM-5 as the catalyst (Table 38, entry 14). Want et al. reported the use of an alkali-treated ZSM-5 as the catalyst for the pyrolysis of waste cooking oil.^1042^ The best results were achieved with the catalyst treated with 0.2 M NaOH aqueous solution for 4 h (4AH-Z5) (Table 38, entry 15). The resulting mixture of products consisted of benzene, toluene, xylenes, ethyl benzene and naphthalene, where the major product was toluene. The group of Zheng reported the use of an alkali treated ZSM-5 catalyst for the conversion of rubber seed oil to an oil mixture of exclusively aromatics of which 78% BTX but without reporting a wt% yield (Table 38, entry 16).^1043^ Yu and co-workers studied La- and Fe-modified Si-MCM41 as the catalyst for the valorization of soybean oil.^1044^ The highest yield of aromatics was achieved with the non-modified Si-MCM41 catalyst (Table 38, entry 17), where the organic liquid fraction (67% yield) consisted of 38% of aromatic compounds. No details of the exact composition of this aromatics fraction were given. The majority of compounds contained 7–13 carbon atoms, and a small amount of phenol was present.

Although fatty acids and oils can indeed serve as feedstock to produce aromatic compounds, economic production will only be possible with waste streams. Exact data on carbon yields are lacking in many cases as often the yield of the liquid fraction is not mentioned and the GC values were often obtained without the use of an internal standard.

### 6.4 From Glycerol

Glycerol is currently mainly produced in the biodiesel industry in the form of crude glycerol, which is about 10 wt% of the biodiesel production.^1045^ Global biodiesel production has grown to 40.9 billion liters in 2019 and is expected to grow by ca. 4.5% annually. The booming of the biodiesel industry has resulted in a significant increase in the amount of crude glycerol, which is projected to be about 4 million tonnes by 2024.^1046^

**Figure 36:**
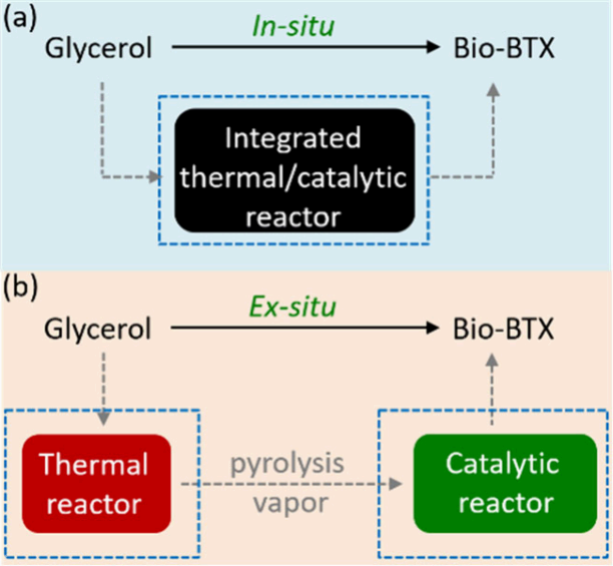
Scheme of the (a) *in situ* and (b) *ex situ* catalytic pyrolysis of glycerol to bio-BTX.

Catalytic pyrolysis of glycerol involves heating the feed to elevated temperatures (e.g., 450–550 °C) in combination with a catalyst. The catalyst is either present in the pyrolysis section (*in situ* catalytic pyrolysis, Figure 36a) or downstream and used to convert the vapor from the pyrolysis unit (*ex situ* catalytic pyrolysis, Figure 36b).^855^ Catalytic pyrolysis of glycerol^1047,1048^ (both with pure and crude glycerol^1049^) has been explored on a lab- and pilot-scale to obtain valuable bio-based aromatics (in particular bio-BTX).

#### 6.4.1 Catalytic Pyrolysis of Pure Glycerol

Thermal pyrolysis of glycerol leads to the formation of condensable products like dehydrated oxygenates (e.g., acrolein, acetaldehyde, acetol, and hydroxypropanal) and gas-phase components (e.g., CO, CH_4_, and C_2_H_4_),^1050^ following dehydration and C–C bond cleavage mechanisms.^1051^ The yields of the liquid oxygenates can be maximized by tuning the pyrolysis parameters such as the temperature and the residence time.^1050,1052^ These oxygenates can be further upgraded to valuable aromatics (in particular BTX) via catalytic pyrolysis^1053^ using dedicated catalysts (e.g., solid acid catalyst) operated at moderate temperatures (e.g., 400–600 °C) and under oxygen-free conditions.

##### 6.4.1.1 Catalysts

The most studied catalysts for the catalytic pyrolysis of pure glycerol are zeolites. Zeolites contain Brønsted acid sites^1054^ and also (in some cases) Lewis acid sites (e.g., extra-framework aluminium species^1055^) for dehydration/cracking reactions whereas the inherent micropores may be used to tune the product selectivity. The product selectivity for the catalytic conversion of glycerol using zeolites is a function of the zeolite structure and process conditions. For instance, it is possible to steer these variables in such a way as to obtain olefins,^1056,1057^ propenal (acrolein^1058,1059^), as well as aromatics. Castello et al.^1060^ investigated the possibility of using H-ZSM-5 for the catalytic conversion of glycerol to aromatics on a CDS Pyroprobe. Systematic studies to probe the effect of the structure of zeolite (including H-ZSM-5, HY, HNaMOR, and HZSM-22) on product composition were performed by Mallinson et al. at relatively low temperatures of 300–400 °C and a high pressure of 2 MPa in a fixed-bed reactor with a space-time of 0.5 h.^1061^ Hydrocarbons were not formed from glycerol when using zeolites with one-dimensional (1-D) pore channels (e.g., HNaMOR and H-ZSM-22). This is likely due to the presence of larger pores in HNaMOR which may promote condensation of small oxygenates to larger ones.^1061^ However, over zeolites with three-dimensional (3-D) pore channels (e.g., HY and H-ZSM-5, Table 39, entries 1 and 2), aromatics (including BTX, C_9_ and C_9_^+^ aromatics) were formed at the expense of oxygenates. Lower amounts of aromatics were produced over HY (containing large pores) when compared to H-ZSM-5 (containing medium pores) (Table 39, entry 1 vs 2). This is likely due to a higher rate of heavier oxygenate formation over HY (19%) than over H-ZSM-5 (5.6%). The yields of aromatics over H-ZSM-5 can be further increased by a tandem configuration, namely hydrodeoxygenation followed by aromatization. In contrast to the single catalyst bed (H-ZSM-5) system, the tandem approach (Pd/ZnO + H-ZSM-5) produces significantly higher amounts of aromatics (18.1% vs 7.4%, Table 39, entries 2 and 3) and particularly more xylenes.^1061^

**Table 39:** Aromatics Yields for Catalytic Pyrolysis of Pure Glycerol Using Various Catalysts^a^

Entry	Catalyst	Reaction conditions	Yields	BTX yield	Ref
1	HY	Fixed-bed reactor, *T*: 400 °C, *P*: 2 MPa, and W/F: 0.5 h	B: 0.3%, T: 0.9%, X: 1.9%	3.1%	(1061)
2	H-ZSM-5	Fixed-bed reactor, *T*: 400 °C, *P*: 2 MPa, and W/F: 0.5 h	B: 1.2%, T: 2.6%, X: 3.6%	7.4%	(1061)
3	Dual bed, Pd/ZnO and H-ZSM-5	Fixed-bed reactor, *T*: 400 °C, *P*: 2 MPa, and W/F: 0.5 h	B: 0.7%, T: 5.6%, X: 11.8%	18.1%	(1061)
4	H-ZSM-5(23)	Fixed-bed reactor, Catalyst: 1 g, *T*_p_: 400 °C, *T*_c_: 500 °C, *P*: atmospheric pressure, N_2_: 1.8 mL min^–1^, and WHSV: 1 h^–1^	B: 6.7 C%, T: 13.3 C%, X: 8.1 C%	28.1 C%	(1062)
5	H-ZSM-5(30)	Fixed-bed reactor, *T*: 400 °C, *P*: 20 bar, and WHSV: 1 h^–1^	B: 6.8 C%, T: 19.8 C%, X: 16.5 C%	39.2 C%	(1063)
6	Zn/H-ZSM-5(30)	Fixed-bed reactor, *T*: 400 °C, P: 20 bar, WHSV: 1 h^–1^, Zn loading 0.64%	B: 10.0 C%, T: 35.3 C%, X: 20.1 C%	65.4 C%	(1063)
7	H-ZSM-5(23)/SiO_2_	Fixed-bed reactor, Catalyst: 1 g, *T*_p_: 450 °C, *T*_c_: 500 °C, P: atmospheric pressure, N_2_: 1.8 mL min^–1^, WHSV: 1 h^–1^	B: 16.5 C%, T: 11.0 C%, X: 3.5 C%	31.0 C%	(1064)
8	Zn-ZSM-5/bentonite	Fixed-bed reactor, *T*: 400 °C, WHSV: 0.228 h^–1^	Aromatics: 9.1 wt%		(16)
9	Y-type FCC catalyst	MAT reactor, *T*: 500 °C	Aromatics: 12.7 C%		(238)
10	H-ZSM-5	Fixed-bed reactor, *T*: 400 °C, *P*: 1 atm, and W/F: 1 h	Aromatics: 36.8 C%		(1061)

a Abbreviated aromatics include benzene (B), toluene (T), and xylenes (X). *T*_p_: pyrolysis temperature, *T*_c_: catalytic vapor upgrading temperature.

Alumina can also be used as catalyst. Shahnazari et al.^1065^ studied the *in situ* catalytic pyrolysis of pure glycerol using alumina as the catalyst in a fixed-bed reactor at 470 °C and 1.3 bar. Considerable amounts of carbon (73%) end up as coke deposited on the alumina catalyst and the carbon selectivity toward aromatics is relatively low (17%). Furthermore, the BTX amount in the aromatics fraction was low and most of the aromatics are heavier cyclic compounds, e.g., C_10_, C_11_, and C_12_.

A Y-type FCC catalyst^238^ has also been investigated for the catalytic pyrolysis of pure glycerol in a micro activity test (MAT) reactor. Most of the glycerol was converted to coke and gases at typical FCC conditions (e.g., 500 °C and TOS of 30 s), resulting in a low carbon yield of aromatics (ca. 12.7 C%, Table 39, entry 9).

As shown above, H-ZSM-5 is the most promising catalyst for the catalytic pyrolysis of (pure) glycerol to produce aromatics. In the following, the performance of a series of unmodified H-ZSM-5 catalysts with different SiO_2_/Al_2_O_3_ ratios, modified H-ZSM-5 catalysts (e.g., with Zn), as well as H-ZSM-5 catalyst in combination with binders (e.g., Al_2_O_3_, SiO_2_, kaolinite, and bentonite), is provided.

The type and number of acidic sites (Lewis and Brønsted acid) in H-ZSM-5 is affected by the SiO_2_/Al_2_O_3_ ratio, leading to different product yields. An optimum was found at a ratio of 30, giving total aromatics yields of 52.2 C%, and 39.2 C% of BTX (Table 39, entry 5).^1063^ Thus, a high Brønsted acidity appears to be good for aromatic formation, but a too high value leads to a reduction in the yield. The latter is likely due to an increase in hydrophilicity,^1063^ which results in competitive absorption between water and oxygenates related to aromatics formation on the acid sites.

A recent benchmark has systematically studied the effects of reaction conditions on the catalytic pyrolysis of pure glycerol using an *ex situ* approach in a continuous set-up using H-ZSM-5 (SiO_2_/Al_2_O_3_ molar ratio of 23) and obtained a peak BTX yield of *ca*. 28.1 C% (Table 39, entry 4).^350^ The catalyst was deactivated at a time scale of hours. The overall BTX yield was 9.9 wt% (or 23.1 C%) and the BTX productivity was 398±55 mg_BTX_ g^–1^_catalyst_ for a catalyst life-time of 5 h. Major byproducts were higher aromatics (e.g., naphthalene and substituted naphthalenes) in ca. 7.0 wt% yield.

Catalytic pyrolysis of glycerol to aromatics over H-ZSM-5 catalyst also produces considerable amounts of olefins,^350^ particularly at high space times.^1063^ It is well known that olefins like ethylene and propylene may also be converted to aromatics when using certain modified zeolites.^1066,1067^ For instance, the aromatics yield was substantially enhanced when promoting an H-ZSM-5(30) catalyst with Zn.^1063^ Best results showed an aromatics (including BTX and C_9_^+^) carbon yield of 80.3% and a BTX yield of 65.4% (cf. the un-modified zeolite: carbon yields of 52.2% for aromatics and 39.2% for BTX, under the same reaction conditions, Table 39, entry 6). Apparently, Zn has a positive effect on catalyst performance, likely due to a combination of effects such as a reduction in Brønsted acidity (by the exchange of Zn^2+^ cations with the proton at Brønsted acid sites to form bivalent Zn cations at the exchanged site)^1063^ and promotion of the rates of dehydrogenation of hydrocarbon intermediates.^84^

**Figure 37:**
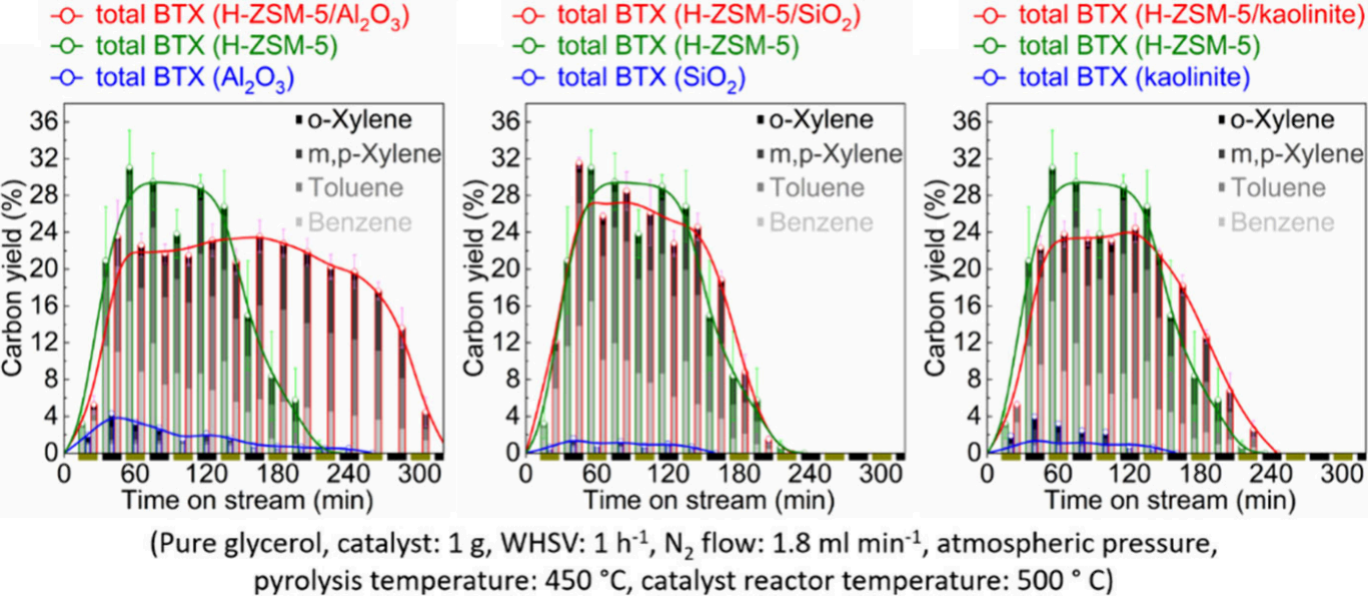
Carbon yields of the total and individual BTX versus TOS over H-ZSM-5, the three binders, and the three H-ZSM-5/binder (90/10 wt%) combinations. Reproduced with permission from ref (1064). Copyright 2021 Elsevier.

Typically zeolites, and particularly when used in continuous reactor configurations, are used in combination with binders such as alumina, silica, and clay.^1068^ Bentonite has been investigated as the binder for H-ZSM-5 catalysts (as such and exchanged with Zn and Mn) for the catalytic pyrolysis of pure glycerol.^16^ The best results were reported for the Zn-ZSM-5/bentonite catalyst (80/20, wt/wt), which gave an almost 3-fold increase in total aromatics yields compared to the parent H-ZSM-5/bentonite catalyst (80/20, wt/wt) (aromatics yields of 9.1 wt% (Table 39, entry 8) vs 3.0 wt%). A more detailed study using three binders, namely alumina, silica, and kaolinite, has been performed recently.^1064^ Among the three binders, Al_2_O_3_ showed the best binder performance such as a considerably prolonged catalyst life-time (320 vs 220 min) and a significantly enhanced total BTX productivity (518 vs 312 mg_BTX_ g^–1^_H-ZSM-5_), but lower peak BTX yield compared to H-ZSM-5 without a binder (Figure 37). The prolonged catalyst life-time is likely due to a higher coke accommodation capacity of the mesoporous Al_2_O_3_ binder than microporous H-ZSM-5, leading to a reduced coking rate for H-ZSM-5.^1064^ This binder effect was also observed for a technical H-ZSM-5/Al_2_O_3_ catalyst with a high binder content of 40 wt%. For this catalyst, the catalyst life-time was prolonged from 6.5 to 8.5 h and the total BTX productivity was increased from 556 to 710 mg_BTX_ g^–1^_H-ZSM-5_ compared to H-ZSM-5 without a binder.^1069^

##### 6.4.1.2 Catalyst Deactivation

It has been shown that zeolite-type catalysts like H-ZSM-5 deactivate considerably during the catalytic pyrolysis of pure glycerol.^350,1061,1064^ For instance, BTX formation was reduced significantly after a TOS of about 5 h when using an unmodified H-ZSM-5 (SiO_2_/Al_2_O_3_ ratio of 23) as the catalyst for the *ex situ* catalytic pyrolysis of glycerol in a fixed-bed reactor (Figure 37).^350^ Mallinson et al. reported that the aromatics yield decreased significantly after a TOS beyond 2 h, accompanied by a dramatic increase in the acrolein yield.^1061^ An even faster catalyst deactivation in terms of aromatics formation was also observed by Blass et al. during the catalytic pyrolysis of pure glycerol at harsher conditions (500 °C and a WHSV of 3.0 h^–1^).^1070^ The yield of aromatics was 20% at a TOS of 20 min, while negligible after a TOS of 1 h, with acrolein and acetaldehyde as the main liquid products.

**Figure 38:**
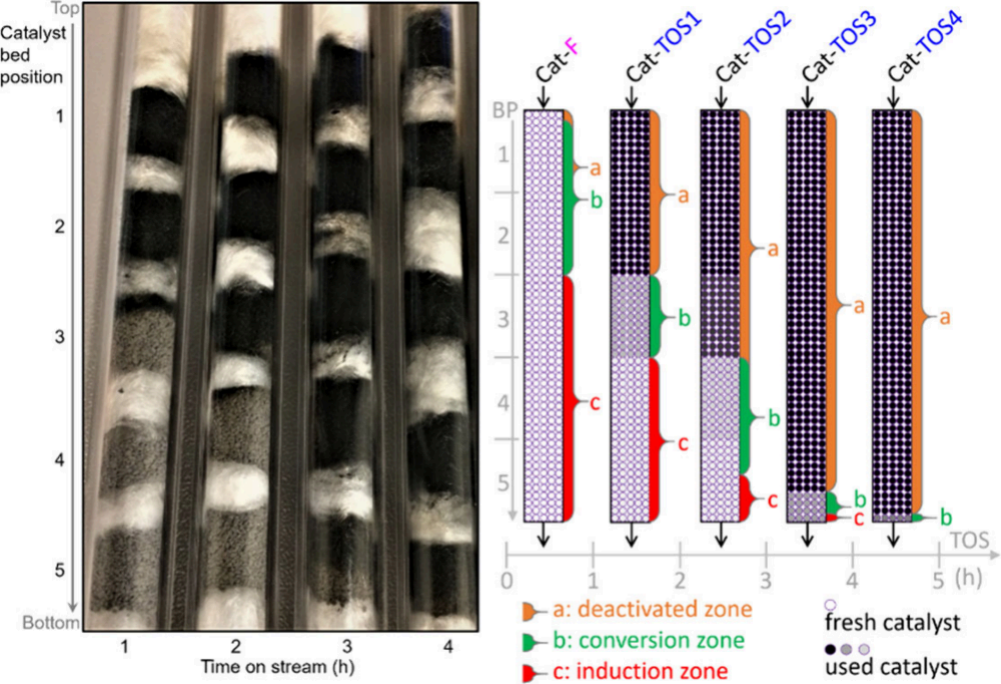
Overview image of the visual appearance of the catalyst in the reactor at various TOS (left, feed is added at the top of the bed) and a schematic representation of the three zones illustrating the conversion-zone migration model for glycerol conversion to aromatics over an H-ZSM-5 catalyst in a fixed-bed reactor (right). Reproduced with permission from ref (1071). Copyright 2022 Elsevier.

Characterization of the spent catalyst after reaction showed that deactivation at a time scale of hours was mainly due to the formation of “hard” coke.^350,1061^ A recent experimental study on the continuous deactivation of an unmodified H-ZSM-5 has visually confirmed the formation of coke at different TOS and positions in the reactor (Figure 38).^1071^ Based on the evolution of the catalyst performance and relevant catalyst characteristics, a conversion-zone migration model describing chemical reactions (Figure 38) and the time- and space-resolved catalyst deactivation process has been proposed.^1071^ According to this model, there are three zones in a fixed-bed reactor, namely a deactivated zone (with severely coked catalyst, near the feed entrance), a conversion zone (mainly for the BTX formation, in the middle of the bed), and an induction zone (for side reactions like (de-)alkylation, nears the exit of the reactor) (Figure 38). Only a minor part of the zeolite in the catalyst bed (conversion zone) is active for BTX formation and this zone migrates from the catalyst bed entrance to the exit with time on stream, ultimately leading to a fully deactivated catalyst bed for aromatics formation.^1071^

**Figure 39:**
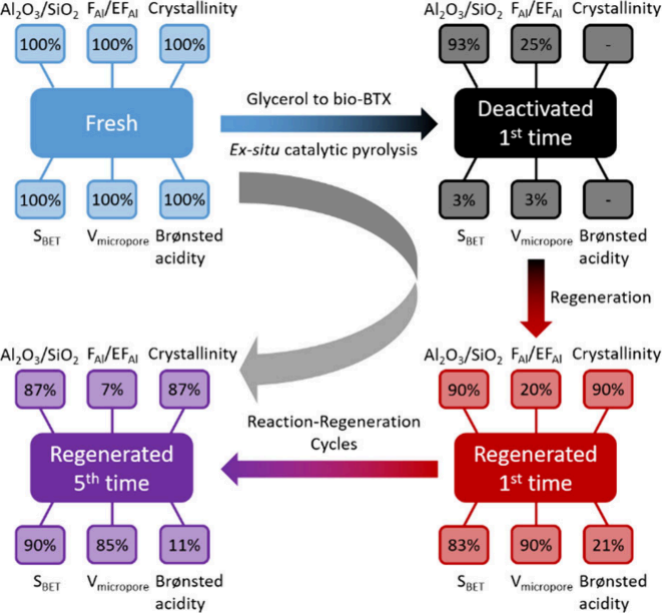
Changes in the H-ZSM-5(23) properties during reaction-regeneration cycles for the catalytic pyrolysis of pure glycerol. Reproduced with permission from ref (350). Copyright 2021 Elsevier.

Regeneration of the catalyst proved well possible by an oxidative treatment with air, though the peak yield of BTX when using regenerated catalysts was reduced with the number of reaction-regeneration cycles. As such, the total BTX productivity over the regenerated H-ZSM-5 catalyst is lowered by approximately 11–18% after each reaction–regeneration cycle,^350^ indicating irreversible deactivation of the catalyst. This is likely attributed to the dealumination of the H-ZSM-5 framework by steam.^350,1064,1069,1071^ Dealumination of the H-ZSM-5 framework occurred mainly during catalytic upgrading and less during oxidative catalyst regeneration.^350^ Dealumination was shown to lead to a significant decrease in the amount of Brønsted acid sites ever after one reaction–regeneration cycle (Figure 39).

**Figure 40:**
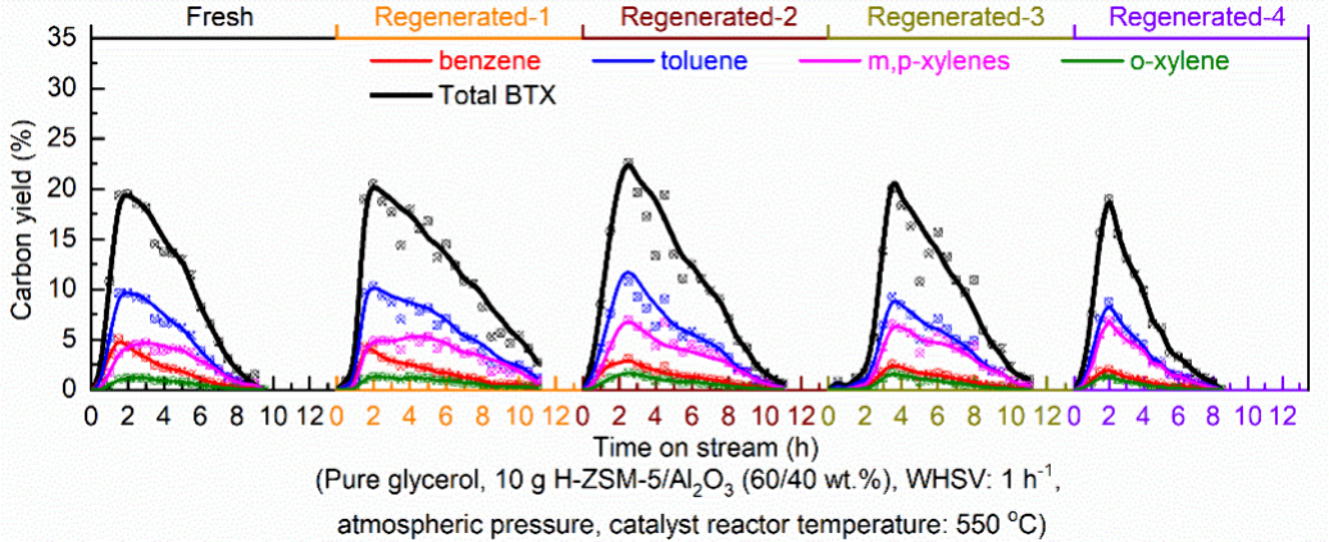
BTX yield versus TOS for recycling experiments for the catalytic pyrolysis of pure glycerol over an H-ZSM-5/Al_2_O_3_ (60/40 wt%) catalyst. Reproduced with permission from ref (1069). Copyright 2022 Elsevier.

Interestingly, when using Al_2_O_3_ as a binder for H-ZSM-5, a positive effect on catalyst stability was observed upon multiple reaction-regeneration cycles^1069^ (Figure 40). After 3 reaction-regeneration cycles, the regenerated H-ZSM-5/Al_2_O_3_ catalyst still shows good performance, which is comparable to the fresh catalyst. The improved catalyst performance is likely due to the transfer of coke from zeolite to the mesopores of Al_2_O_3_ and the newly formed ones upon catalyst synthesis, lowering the coking rate on the zeolite. In addition, the extent of dealumination of the H-ZSM-5 zeolite framework is reduced, likely due to the transfer of Al from Al_2_O_3_ to the framework.^1069^

##### 6.4.1.3 Mechanisms

**Figure 41:**
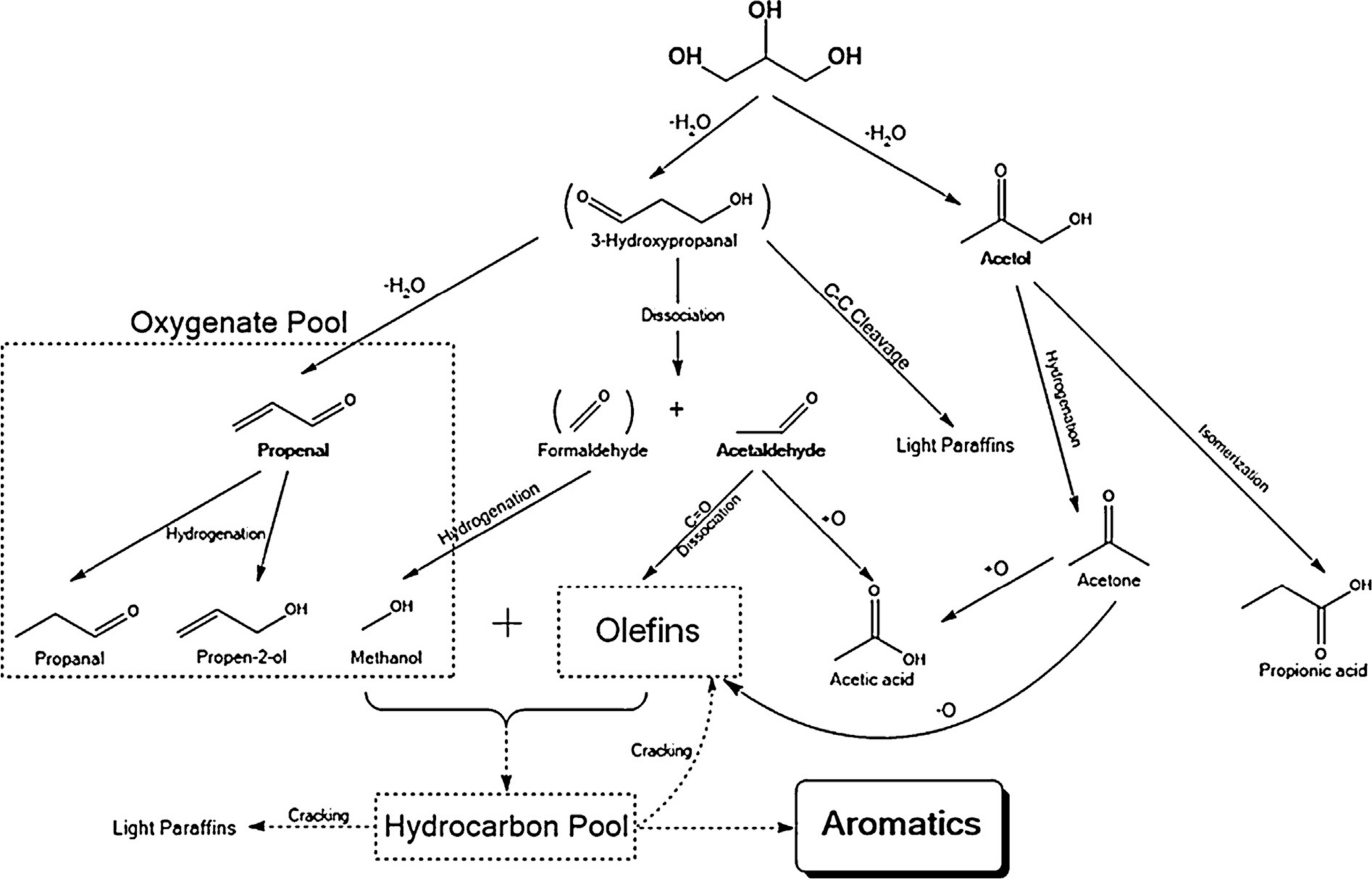
Reaction network for GTA. Reproduced with permission from ref (1063). Copyright 2015 Elsevier.

The catalytic pyrolysis of glycerol gives condensable and non-condensable products. Typical condensable products are oxygenates like 3-hydroxpropanal, acetol, acrolein, acetaldehyde, formaldehyde, propanal, propen-2-ol, methanol, acetone, acetic acid, and hydrocarbons like BTX, ethylbenzene, trimethylbenzenes (TMBs), and naphthalene. In addition, water is formed by various dehydration reactions.^238,1061,1063,1072^ Some of the oxygenates are also formed by thermal pyrolysis (without using a catalyst) and are prone to hydrogenation, de-hydrogenation, and C-C cleavage reactions, ultimately leading to a so-called “hydrocarbon pool” on the catalyst surface that is the source for aromatics formation by several (acid-catalysed) reaction pathways. A proposed reaction network is shown in Figure 41.

##### 6.4.1.4 Kinetics

The kinetics of the *in situ* catalytic pyrolysis of pure glycerol over H-ZSM-5 has been studied by thermogravimetry by Castello et al.^1073^ The activation energy for the overall catalytic pyrolysis of pure glycerol was calculated using the Kissinger method and found to be 77.4 kJ mol^–1^ and 65.2 kJ mol^–1^ when pure glycerol was mixed with 1 wt% and 5 wt% of ZSM-5 catalyst, respectively.^1073^ These values are much lower than those for the thermal pyrolysis of pure glycerol (ca. 105 kJ mol^–1^),^1074^ indicating the catalytic effect of ZSM-5.

Xiao et al. developed a lumped kinetic model (Table 40), including four lumped reactions (R1–R4), of which all involve more than one elementary reaction.^1075^ For instance, step R4 includes oligomerization, cyclization, and dehydrogenation. The fitting results indicated that the orders of Step R1, R2, R3, and R4 are 1, 2, 2, and 1, respectively (Table 40). The activation energy of Step R2 (121 kJ mol^–1^) is smaller than that of Step R3 (147 kJ mol^–1^), indicating that oxygenates tend to form gases (R2) rather than directly being converted to aromatics (R3).

**Table 40:**
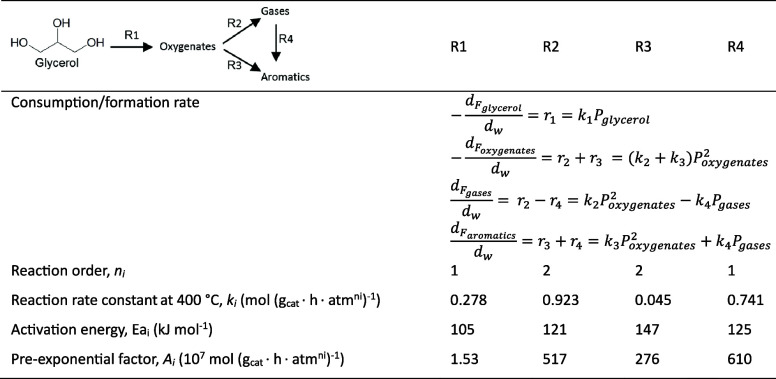
Reaction Rates and the Fitted Kinetic Parameters for the Catalytic Pyrolysis of Pure Glycerol over a Pd/H-ZSM-5 Catalyst^a^

a Data were taken from ref (1075).

#### 6.4.2 Catalytic Pyrolysis of Pure Glycerol with Co-feeds

Besides the focus on the catalytic conversion of pure glycerol using various catalysts, a lot of attention has been given to the conversion of glycerol in combination with other compounds, including water, alcohols, alkanes, and vegetable oils. The state-of-the-art performance of catalytic co-pyrolysis of glycerol with these co-feeds is summarized in Table 41.

**Table 41:** Aromatics Yields for Catalytic Co-pyrolysis of Pure Glycerol with Various Co-feeds

Entry	Co-feeds	Catalyst	Conditions	Result	Ref
1	Glycerol and H_2_O	15%CNT-OH/H-ZSM-5/Sono	Continuous fixed-bed reactor, glycerol/H_2_O 40/60 wt%, *T*: 400 °C, *P*: 1 atm, N_2_, and WHSV: 0.71 h^–1^	BTX peak carbon yield of 27.4 C%; catalyst lifetime of ca. 8.5 h	(1076)
2	Glycerol and alcohols	20Zn@Sn/H-ZSM-5	Continuous fixed-bed reactor, glycerol/CH_3_OH 40/60 wt%, *T*: 400 °C, *P*: 1 atm, N_2_, and WHSV: 0.71 h^–1^	BTX peak carbon yield of 38 C%; catalyst lifetime of ca. 10.5 h	(1077)
3	Glycerol and alkanes	Pd_1.1_-Zn_9.2_/Y_2_O_3_-Al_2_O_3_ extruded with H-ZSM-5 and bentonite	Continuous fixed-bed reactor, glycerol/hexane 50/50 wt%, *T*: 635 °C, *P*: 1 atm, steam, and WHSV: 1.5 h^–1^	BTX mass yield of 12.0 wt%	(1078)
4	Glycerol and vegetable oil	H-ZSM-5/Al_2_O_3_ (60/40 wt%)	Continuous fixed-bed reactor, glycerol/oleic acid 45/55 wt%, *T*: 550 °C, N_2_, *P*: 1 atm, and WHSV: 1 h^–1^	Peak BTX yield of 26.7 C%; BTX productivity of 834 mg_BTX_ g^–1^_catalyst_ during a catalyst lifetime of ca. 11 h	(1079)

The catalytic pyrolysis of aqueous glycerol has been studied using H-ZSM-5-based catalysts to investigate the effect of water on BTX yields.^239,1080,1081^ These studies were motivated by the fact that biomass-derived crude glycerol usually contains a certain amount of water.^1049^ In addition, glycerol itself is relatively viscous (e.g., 1.41 Pa·s at 20 °C),^1082^ and the addition of water lowers the viscosity which simplifies feeding at low temperatures to continuous units. Besides the use of standard batch and continuous reactors, also some research has been performed in a Riser Simulator^1083^ and micro activity test (MAT) reactors^238^ to assess the feasibility of co-processing aqueous glycerol in an FCC process.

Tarasov^1084^ studied the catalytic pyrolysis of glycerol-water mixtures (85 wt% glycerol) using ZSM-5 catalysts (SiO_2_/Al_2_O_3_ = 30) at a temperature of 340 °C in a fixed-bed reactor and obtained a glycerol conversion of 84.2% and an aromatic selectivity of 41.3%. Huber et al.^239^ reported the catalytic pyrolysis of glycerol/H_2_O (12.5/87.5 wt%) over a ZSM-5 catalyst (SiO_2_/Al_2_O_3_ = 30) in a fixed-bed reactor at higher temperature (600 °C) and a WHSV of 11.7 h^–1^. The products are CO, CO_2_, light olefins (including ethylene, propylene and butenes), aromatics (mainly BTX, of which toluene has the highest selectivity), and coke. The maximum carbon yield of total aromatics was 17.8 C%. Suh^1081^ reported a maximum carbon yield of total aromatics of 26.1% from glycerol/H_2_O mixtures (30/70 wt%) over a ZSM-5 catalyst. The effect of the weight ratio of glycerol and water on catalytic performance was also investigated.^1080,1081^ It was shown that higher amounts of glycerol in the feed led to a decreased carbon yield of the aromatics and also a different BTX selectivity.

Compared to the un-modified H-ZSM-5 above, modified H-ZSM-5 with various metals (including Ga, Zn, and Cu) only showed slight improvements when considering the carbon yields of BTX and total aromatics.^1080^ The best performance of catalytic pyrolysis of glycerol/H_2_O (30/70 wt%) was obtained over a Cu-ZSM-5 catalyst, showing a total aromatics carbon yield of 33.9%. A hierarchical H-ZSM-5 was even more efficient (catalytic activities and life-time) than the traditional microporous H-ZSM-5 for the conversion of glycerol to aromatics. For instance, the hierarchical 15% CNT-OH/H-ZSM-5/Sono catalyst showed nearly a doubling of the BTX peak carbon yield (27.4 (Table 41, entry 1) vs 14.1 C%) and catalyst life-time (8.5 vs 2.5 h), compared to microporous H-ZSM-5.^1076^ This is likely due to the improved diffusivity and enhanced access of substrates to the micropores and the acid sites.^1076,1085^

Co-feeding of glycerol with alcohols has been extended from methanol to a wide range of other alcohols such as ethanol, i-propanol, and i-butanol.^1080,1081,1086,1087^ Initially, the performance was tested for the individual alcohols over an H-ZSM-5 (SiO_2_/Al_2_O_3_ = 30) catalyst at 400 °C and a WHSV of 0.8 h^–1^. The carbon yields of total aromatics for MeOH, EtOH, and i-PrOH were about similar (31–32%), whereas that for i-BuOH was slightly higher (37%).^1080,1081^ MeOH mainly gives xylenes (carbon fraction of 44%) and TMBs (carbon fraction of 28%), whereas the other alcohols (ethanol, i-propanol, and i-butanol) are mostly converted into toluene (carbon fraction between 36 and 39%) and xylenes (carbon fraction between 31 and 34%).^1081^ Catalytic pyrolysis of glycerol/alcohol mixtures with glycerol concentration of 10–50 wt% gave higher carbon yields of total aromatics than found for the individual alcohol feeds.^1081^ This finding is of high interest as the glycerol byproduct from the biodiesel industry also contains methanol.^1088^ As such, when aiming for BTX formation, separation and purification of the glycerol is not required and actually is disfavored when considering BTX yields and purification costs. Product selectivity was found to be depending on the blend ratio.^1081^ When increasing the amount of mono-alcohol, more benzene and toluene were formed, and the carbon yield of xylenes and especially TMBs were significantly reduced.

**Figure 42:**
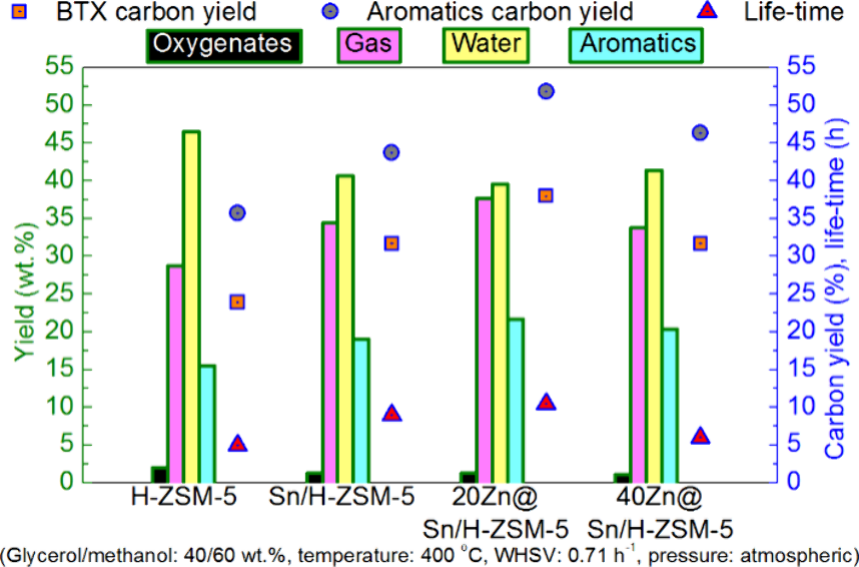
Product yields and catalyst life-time for the catalytic pyrolysis of glycerol/methanol mixtures over Zn- and Sn-modified H-ZSM-5 catalysts. Data were taken from Ref^1077^

Similar to the catalytic pyrolysis of pure glycerol (vide supra), the SiO_2_/Al_2_O_3_ ratio of the H-ZSM-5 zeolite has an effect on the yield of aromatics for the catalytic pyrolysis of glycerol/methanol blends.^1063^ Higher SiO_2_/Al_2_O_3_ ratios lead to a reduction of the aromatics yields, rationalized by considering the reduction in acidity when increasing the SiO_2_/Al_2_O_3_ ratio. Catalyst deactivation rates by coking show a correlation with the SiO_2_/Al_2_O_3_ ratio and higher rates are observed when the value is less than 30.^1080^ An optimal SiO_2_/Al_2_O_3_ ratio of 30 was found for H-ZSM-5, giving a BTX carbon yield of 26% and a total aromatics yield of 45%, for the catalytic pyrolysis of glycerol/methanol mixtures,^1080^ in line with optimum values found for the catalytic pyrolysis of pure glycerol.^1063^ Modifications of H-ZSM-5 by, e.g., steaming, acid leaching, alkaline leaching, successive acid leaching/steaming, and successive alkaline leaching/steaming have been studied by Wang et al.^1089,1090^ The highest BTX yield of ca. 32.5 C% was obtained for H-ZSM-5 by acid leaching/steaming, whereas the highest aromatics yield of ca. 43.5 C% and the longest lifetime of ca. 19 h was obtained on H-ZSM-5 by acid leaching/steaming. This is most likely related to the formation of a micro- and mesoporous structure with moderate acidity after modification.^1089,1090^ Alternatively, modifications of H-ZSM-5 by metals, e.g., Zn, Mo, Ag, Ni, and Sn, have been studied by Xiao et al.^1072,1077,1091−1093^ and Suh et al.^1080^ Both enhanced BTX yields (17.9 wt%) and a prolonged life-time (7.5 h) were found for the Sn/H-ZSM-5 catalyst.^1072^ The performance of Sn/H-ZSM-5 catalysts for catalytic pyrolysis of glycerol/methanol can be further improved by introducing Zn via atomic layer deposition (ALD).^1077^ For instance, a 20Zn@Sn/H-ZSM-5 catalyst (prepared by 20 cycles of ALD, Zn atomic loading is 1.23%) gives a higher carbon yield of total aromatics and BTX production (ca. 52% and 38%, respectively) than the parent Sn/H-ZSM-5 and H-ZSM-5 catalysts (Figure 42). This points toward a synergistic effect between Sn and Zn species. This may be facilitated by the preparation procedure using ALD, which is known to introduce Zn both on the external surface and in the micropore channels. The Zn species (mainly Zn(OH)+) inside the micropore channels provide the active sites for relevant reactions (e.g., dehydrogenation) to transform the intermediates to aromatics.

Alkanes such as dodecane,^1065^ hexadecane,^1065^ and hexane^1078^ have also been blended with glycerol to enhance aromatic yields for catalytic pyrolysis and to lower the rate of coke deposition. The effect on the blend ratio was studied by Shahnazari et al. in a fixed-bed reactor using an alumina catalyst.^1065^ Best results were obtained for a 1 to 1 feed ratio between glycerol and hexadecane at a temperature of 470 °C. Compared with the performance for pure glycerol, a remarkable improvement in organic product yield (14.4 wt% vs. 6.8 wt%) was observed, the amount of coke formation was reduced (7.1 wt% vs. 20.6 wt%), and higher carbon yield toward aromatics (36% vs. 17%) and BTX (14.3% vs. 0.7%) were obtained, showing the potential of co-feeding with long-chain alkanes.

Le Van Mao et al.^1078^ performed experiments with glycerol/n-hexane blends in combination with steam using hybrid catalysts. The catalysts were prepared by extruding Pd_1.1_-Zn_9.2_/Y_2_O_3_-Al_2_O_3_ (16.4 wt%), H-ZSM-5 (65.6 wt%, Si/Al = 50, and S_BET_ = 403 m^2^.g^–1^) and bentonite (18 wt%, as the binder). It was shown that the presence of glycerol has a positive effect on BTX and ethylene yields and resulted in a decrease in the amounts of propylene and C_4=_ yields (Table 42). Further improvements were possible by replacing Pd in the catalyst formulation by Ru (Table 42).

**Table 42:** Yields of the Products from Catalytic Pyrolysis of Glycerol/Hexane^a^^,^^1078^

	Pd_1.1_–Zn_9.2_/Y_2_O_3_–Al_2_O_3_ extruded with H-ZSM-5 and bentonite			Ru_0.5_–Pd_1.1_–Zn_9.2_/Y_2_O_3_–Al_2_O_3_ extruded with H-ZSM-5 and bentonite
Glycerol/Hexane (wt%/wt%)	0/100	30/70	50/50	30/70
CH_4_ (wt%)	no data	5.6	no data	4.6
C_2_–C_4_ paraffins (wt%)	no data	10.6	no data	10.8
C_2_^=^ (wt%)	13.0	14.9	15.5	12.9
C_3_^=^ (wt%)	26.0	23.8	18.7	21.9
C_4_^=^ (wt%)	7.7	6.6	4.9	5.8
BTX aromatics (wt%)	4.8	7.5	12.0	10.9
Coke (wt%)	0.5	1.1	2.1	1.3

a Reaction conditions: temperature of 635 °C, WHSV of glycerol/*n*-hexane of 1.5 h^–1^, steam/(glycerol/*n*-hexane) weight ratio of 0.5, and TOS of 4 h. Data were taken from Ref^1078^

**Figure 43:**
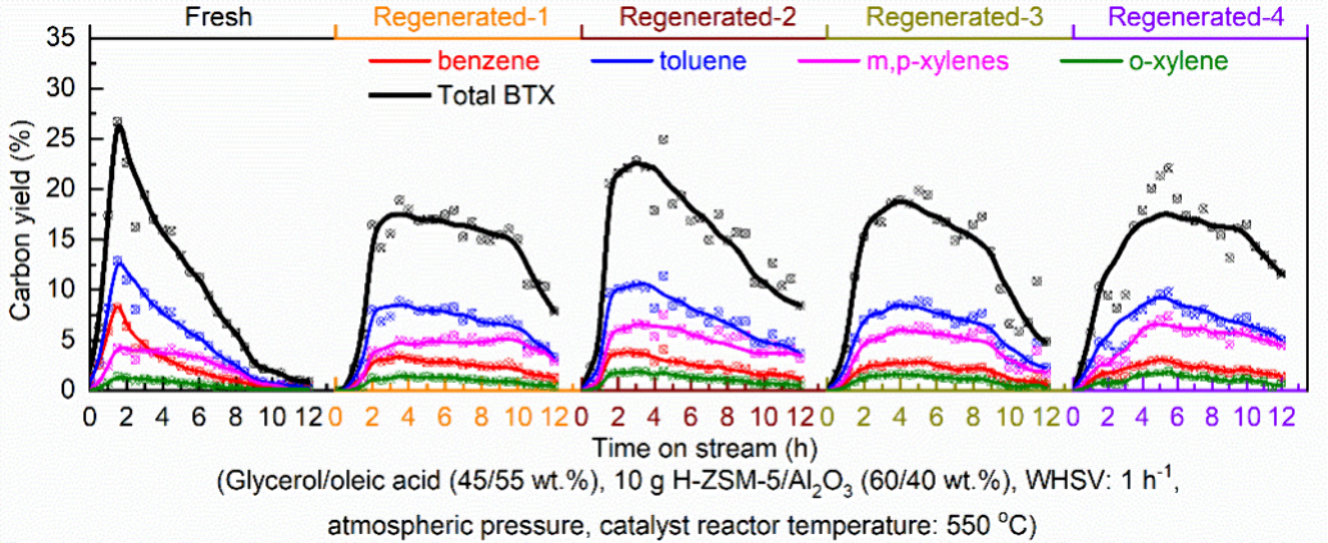
BTX yield *versus* TOS for recycling experiments for the catalytic co-conversion of glycerol/oleic acid (45/55 wt%) over an H-ZSM-5/Al_2_O_3_ (60/40 wt%) catalyst. Reproduced with permission from ref (1079). Copyright 2022 Elsevier.

Vegetable oils, particularly non-edible ones, are considered interesting bio-based sources for bio-aromatics (section 6.3) and are also present in crude glycerol.^1032,1049^ The co-feeding of glycerol and the model compound for vegetable oils–oleic acid with a mass ratio of 45/55 wt% has been studied.^1079^ It was shown that co-processing of glycerol with oleic acid (glycerol/oleic acid ratio of 45/55 wt%) resulted in improved catalyst stability. An extension of the catalyst life-time to ca. 11 h was observed (Figure 43), which is by far longer than found for glycerol alone (8.5 h) and oleic acid alone (6.5 h).^1032^ As a result, the total BTX productivity is significantly improved, from 834 mg_BTX_ gcat^–1^ for the co-conversion of glycerol/oleic acid, compared to 426 mg_BTX_ gcat^–1^ for glycerol alone and 739 mg_BTX_ gcat^–1^ for oleic acid alone.^1032^ More interestingly, catalyst regeneration and recycling studies showed that regenerated catalysts perform better than the fresh ones when considering catalyst life-time (Figure 43, > 12 h vs 11 h) and total BTX productivity (>1505 mg_BTX_ gcat^–1^ vs. 834 mg_BTX_ gcat^–1^).^1079^

**Figure 44:**
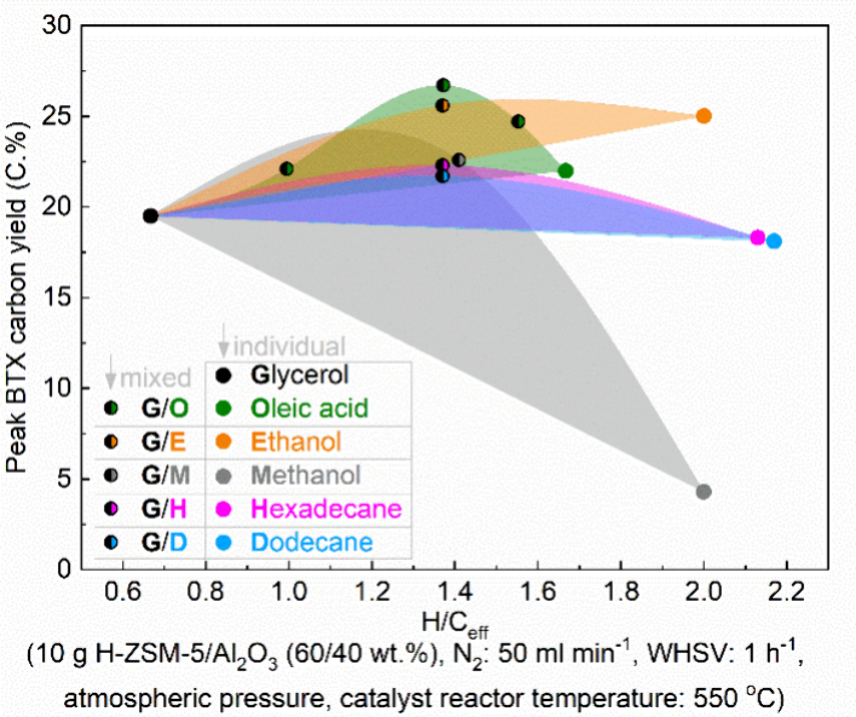
Peak BTX carbon yield versus the hydrogen to carbon effective ratio of the (co-)feed. Reproduced with permission from ref (1094). Copyright 2022 Royal Society of Chemistry.

The data shown above on the catalytic pyrolysis of glycerol with various co-feeds reveal that co-feeding has a positive effect on catalyst performance. Recently, Heeres et al. systematically studied the catalytic co-conversion of glycerol with fatty acids, alcohols, and alkanes and observed remarkable and unprecedented synergetic effects of co-feeding leading to (i) higher peak BTX carbon yields (Figure 44), (ii) longer catalyst life-times, (iii), higher total BTX productivity, and (iv) a reduction of the extent of irreversible deactivation.^1094^ The best results were obtained from the catalytic co-conversion of glycerol/oleic acid (45/55 wt%), showing a peak BTX carbon yield of 26.7 C%, a total BTX productivity of 834 mg_BTX_ g_catalys_t^–1^, and negligible irreversible catalyst deactivation after 5 reaction-regeneration cycles (Figure 43).^1079^

#### 6.4.3 Catalytic Pyrolysis of Crude Glycerol

The catalytic pyrolysis of crude glycerol is by far less studied than for pure glycerol (alone) and blended glycerol with co-feeds. Heeres et al. investigated the catalytic pyrolysis of crude glycerol (1 g) under N_2_ at 550 °C in a batch tandem fixed-bed reactor using H-ZSM-5/bentonite (60/40 wt%) as the catalyst (3 g).^1049^ Crude glycerol is converted mainly to a bio-liquid (33.5 wt%, <2% water) and 12.9 wt% of gas-phase components. The liquid mainly contains mono- and polycyclic aromatics, whereas the gas phase is mainly composed of alkanes.

**Figure 45:**
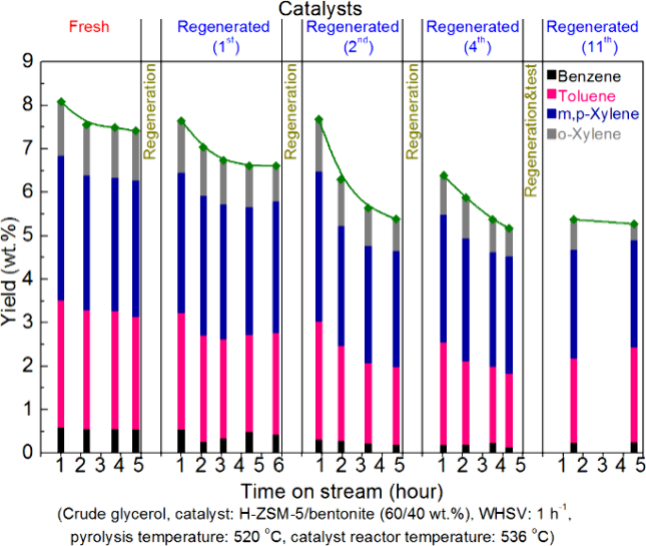
TOS-dependent BTX yield of the *ex situ* catalytic pyrolysis of crude bio-glycerol. Reproduced with permission from ref (1049) Copyright 2018 Elsevier.

The continuous *ex situ* catalytic pyrolysis of crude bio-glycerol was investigated in a fixed-bed reactor with the capacity of converting *ca*. 200 g crude glycerol h^–1^ using 200 g H-ZSM-5/bentonite (60/40 wt%) extrudates as catalyst.^1049^ A total BTX yield of 8.1 wt% (14.6 C%) was obtained over the fresh catalyst at a TOS of 1 h (Figure 45). After 4.7 h TOS, the total BTX yield decreased to 7.4 wt%, indicative of some catalyst deactivation. Moreover, after a TOS of 3.7 h, the product also contains (substituted) phenols and alkanes (e.g., C7–C11),^1049^ indicating that the deoxygenation and aromatization functions of the catalyst are deteriorating. The regenerated catalyst after coke removal by oxidation in air at 600 °C for 8 h was recycled and a total BTX yield of 7.7 wt% was obtained (Figure 45), which is ca. 95% of that over the fresh catalyst. This indicates that the deactivated catalyst can only be partially regenerated via oxidation in air, which is indicative for some irreversible deactivation. Detailed catalyst characterization studies showed that textural properties of the catalyst like pore structure (surface area and pore volume, ca. 90% of recovery) and acid sites (Lewis and Brønsted acid, ca. 28% of recovery) are changing upon regeneration.^1049^ After 11 cycles, the total BTX yield produced over the regenerated catalyst was decreased to 5.4 wt% (Figure 45), which is ca. 2/3 of that over the fresh catalyst. This indicates that irreversible catalyst deactivation is more severe after multiple reaction-regeneration cycles. It was shown by extensive catalyst characterization studies^1049^ that the bentonite structure was collapsed after 11 times of regeneration due to the removal of interlamellar water and dehydroxylation. Furthermore, ion exchange between the cations (e.g., Na) from the bentonite binder with the protons of H-ZSM-5 was observed. This led to a remarkable decrease in catalyst acidity.

#### 6.4.4 Conclusion and Perspective

Catalytic conversion of glycerol to bio-based aromatics is an attractive option to green-up the current petrochemical industry. Significant progress has been made in this respect and aromatics and BTX yields as high as 80.3 C% and 65.4 C%, respectively, have been reported using a Zn-modified H-ZSM-5 as the catalyst. However, the technology is still at a relatively low TRL level (max. 6) and to the best of our knowledge has only been demonstrated at a pilot scale with inputs in the order of tens of kgs per hour.

H-ZSM-5 is the most studied catalyst for the catalytic pyrolysis of glycerol to aromatics. Improvements in catalytic performance are possible by modifying H-ZSM-5 by using metal promotors (e.g., Zn and Sn) and the use of hierarchical H-ZSM-5 catalyst with micro and mesopores by for instance an alkali treatment. The blending of glycerol with co-feed with higher H/C_eff_ values such as alcohols, alkanes, and vegetable oils, has shown to be very beneficial when considering BTX yield and catalyst lifetime.

A major drawback of the technology is the rapid deactivation of the catalyst by coking on a time scale of hours. The highest total BTX productivity of 1390 mg_BTX_ g_H-ZSM-5_^–1^ was reported for a fresh technical H-ZSM-5/Al_2_O_3_ (60/40 wt%) catalyst for the conversion of the glycerol/oleic acid (45/55 wt%). Therefore, catalyst recycling after regeneration is of prime importance. Catalyst regeneration via coke removal by an oxidative treatment is well possible, though some irreversible deactivation of the zeolite typically occurs upon multiple regeneration/reuse cycles. This issue should be solved both on the molecular (proper catalyst modifications) and reactor/process level.

## 7 Aromatics from Terpenes

In this section one-step catalytic transformations of terpenes to benzenoid aromatic compounds will be discussed. It should be emphasized that currently not many terpenes are isolated from renewable resources. Many are produced synthetically from acetone and acetylene as the main building blocks. Nevertheless, the following terpenes are currently obtained from renewable resources: α- and β-pinene and 3-carene (from wood as side product from pulp and paper production),^1095^ α- and γ-terpinene (from pine oil),^1096^ and limonene (from orange peels). We found one earlier review on the conversion of terpenes to aromatics.^1097^

### 7.1 From Limonene

Transformation of limonene (**T1**) to *p*-cymene (T2) is the most reported transformation of limonene to a benzenoid aromatic compound. *p*-Cymene is a flavoring agent, a constituent of cough syrups and finds use as a ligand in transition metal complexes. The summary of all reported transformations is shown in Table 43. The reaction is in essence a double bond isomerization and a dehydrogenation. Palladium catalysts were often used for this reaction. Kou and co-workers found that up to 87% yield of **T2** could be achieved with Pd nanoparticles as the catalyst (Table 43, entry 1).^1098^ These nanoparticles were specially prepared using poly-(*N*-vinyl-2-pyrrolidone) with Mw of 630 kg mol^–1^ as the stabilizer. The group of Jaekel reported the use of Pd(OTFA)_2_ as the catalyst and CuCl_2_ as an additive.^1099^ Different additives were tested, and the highest yield of **T2** (67%) was achieved in the presence of Bu_4_NBF_4_ (Table 43, entry 2). Clark and co-workers reported a procedure catalyzed by Pd/C, where the authors isolated **T2** in 89% yield.^1100^ Hailes and co-workers used palladium trifluoroacetate as the catalyst in the presence of over stoichiometric amounts of CuCl_2_ and 2,6-lutidine as additives to afford a 30% yield of **T2** from **T1** (Table 43, entry 4).^1101^ Zhang and Zhao reported Pd-catalyzed dehydrogenation of **T1** to **T2** in 2015.^1102^ The highest yield of 81% was obtained when Pd on amorphous silica alumina was used as the catalyst (Table 43, entry 5). A year later the same group reported several Pd catalysts on zeolites for this transformation. Pd/H-ZSM-5 (197) catalyst (Si/Al ratio of 197) afforded the highest yield of **T2** of 83% (Table 43, entry 6).^1103^ Pd/Al_2_O_3_ as the catalyst was also reported to be efficient for the conversion of **T1** to **T2** in 82% yield by the group of Beekwilder (Table 43, entry 7).^1104^ F. Chemat, M. Touaibia and co-workers studied the hydrogenation of **T1** to menthane (1-isopropyl-4-methyl-cyclohexane) and menthene (1-isopropyl-4-methyl-cyclohexene).^1105^ They found, that in the presence of Pd/C as the catalyst up to 61% yield of **T2** can be achieved even under reductive conditions (Table 43, entry 8). In 2020, Mekkaoui, El Houssame and co-workers reported Pd catalysts supported on mesoporous natural phosphate for the dehydrogenation of **T1**. The authors obtained a 90% yield of **T2**, and isolated the desired product in 88% yield (Table 43, entry 9).^1106^ Metin and co-workers conducted dehydrogenation of **T1** using Cu_50_Pd_50_/m-gCN nanoparticles (Table 43, entry 10).^1107^ After a work-up the resulting mixture contained 90% of **T2**.

**Table 43:**
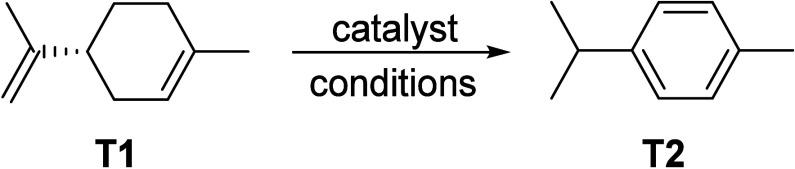
Conversion of Limonene to *p*-Cymene (In Some Cases Limonene Was Used as a Racemic Mixture)

Entry	Catalyst	Catalyst/metal loading	Additional data	*T* (°C)	*t* (h)	Yield of T2 (%) [isolated]	Ref
1	Pd nanoparticles	2 mol%	H_2_ gas (2 bar), H_2_O as solvent	150	3	87^a^	(1098)
2	Pd(OTFA)_2_	5 mol%	2 eq. CuCl_2_, 6 eq. Bu_4_NBF_4_, DMF as solvent	80	40	67^b^	(1099)
3	10 wt% Pd/C	1 mol%	K-10 montmorillonite clay was used	140	1	[89]^c^	(1100)
4	Pd(OTFA)_2_	5 mol%	CuCl_2_ (2 eq.), 2,6-lutidine (3 eq.), cyclomethyl-pentyl ether as a solvent	90	40	30^a^	(1101)
5	1 wt% Pd/ASA	0.03 mol%	N_2_ gas (8 bar), dodecane as the solvent	280	2	81^a^	(1102)
6	1 wt% Pd/H-ZSM-5 (197)	0.03 mol%	N_2_ gas (8 bar), dodecane as the solvent	260	2	83^a^	(1103)
7	5% Pd/Al_2_O_3_		Acetone as the solvent	125	1	82^a^	(1104)
8	10 wt% Pd/C	1.36 mol%	H_2_ gas (27.5 bar), solvent-free	RT	2	61^b^	(1105)
9	3.5 wt% Pd/NP	0.23 mol%	Solvent-free	176	24	90 [88]	(1106)
10	Cu_50_Pd_50_/m-gCN	3 mol%	Neat	180	12	90^d^	(1107)
11	H_5_PMo_10_V_2_O_40_	1 mol%	Tetraglyme (0.2 eq.), O_2_ gas (1 bar), dichloroethane as a solvent	70	20	98 [70]	(1108)
12	H_5_PV_2_Mo_10_O_40_	2 mol%	PEG-200 as solvent, O_2_ gas (2 bar)	100	16	74^e^	(1109)
13	Sepiolite/Ni	417 wt%	Microwave reaction	165	0.33	100^a^	(1110)
14	Sepiolite/Fe	417 wt%	Microwave reaction	165	0.33	100^a^	(1110)
15	Sepiolite/Mn	417 wt%	Microwave reaction	165	0.33	100^a^	(1110)
16	FeCl_3_	0.13 mol%	Na (0.2 eq.), NH_2_CH_2_CH_2_NH_2_ (0.7 eq.)	100	8	[99]	(1111)
17	Ni-zeolite A	6.5 g	Limonene:H_2_ = 1:3, reaction time = 3.6 seconds	180	Continuous flow	21^a^	(1112)
18	Dibromolimonene	3 mol%	AcOH as a solvent	174–178	4	45^f^	(1113)
19	I_2_	2 mol%	AcOH as a solvent	174–178	4	48^f^	(1113)
20	Cl_3_CCOOH	4 mol%	AcOH as a solvent	174–178	4	25^f^	(1113)
21	MgCl_2_	3 mol%	H_2_O as the solvent	300	3	20^g^	(1114)
22	I_2_	50 mol%	DDQ (0.5 eq.), toluene as a solvent	176	0.75	[82]	(1115)
23	[H(OEt_2_)_2_][BAr^F^_4_]	10 mol%	2,6-di-*t*Bu-4-(diphenylmethylene)cyclohexa-2,5-dien-1-one as an oxidant, C_2_H_4_Cl_2_ as a solvent	90	12	>99%^h^	(1116)
24	MV^2+^/Na-Y^I^	12 wt%	Dry hexane, argon, dark	RT	0.5	60^b^	(1117)
25	thionin/Na-Y	120,000 wt%	Hexane as a solvent	RT	0.5	29^b^	(1118)
26	TiO_2_	2.50 g	F = 7.6 g h^–1^, WHSV = 3.04 h^–1^	425	Continuous flow	65^a^	(1119)
27	30% ZnO/SiO_2_	0.2 g	WHSV = 0.080 h^–1^	300	4	98^a^	(1120)

a GC yield without use of internal standard.

b Analyzed by GC using dodecane as a standard.

c The purity of the isolated product was only 71%.

d ^1^H NMR yield after a work-up without use of any standard.

e GC yield using standard without providing details of what the standard is.

f Unclear how the yield was measured.

g Yield determined by ultraviolet absorption.

h The yield was determined by GC-MS using decane as internal standard.

I MV = methyl viologen.

Apart from Pd catalysts, other transition metal catalysts are also known for the conversion of **T1** to **T2**. In 1989 Neumann and Lissel achieved a 98% yield of **T2** (with 70% isolated yield) using H_5_PMo_10_V_2_O_40_ as a catalyst (Table 43, entry 11).^1108^ The same catalyst was applied for the same reaction by Haimov and Neumann, where 74% of **T2** was obtained (Table 43, entry 12).^1109^ Martin-Luengo et al. tested sepiolites doped with different metals for the dehydrogenation of **T1** to **T2** under dry media (large excess of solid over liquid) and solventless conditions.^1110^ These reaction afforded **T2** in 100% yields after 20 minutes with 3 different catalysts (Table 43, entries 13 – 15). Colonna and co-workers reported the iron-catalyzed synthesis of **T2** from **T1**.^1111^ The authors performed the reaction on a large scale (101 grams of limonene) and obtained 99% isolated yield of the desired product (Table 43, entry 16). Popov et al. used a zeolite A supported nickel catalyst for hydrogenation of **T1**.^1112^**T2** was formed as a side product in 21% yield (Table 43, entry 17).

Transition metal free catalysts have also been investigated in this transformation. Back in 1945 Ipatieff et al. reported the disproportionation of **T1** to **T2** and saturated cyclohexanes in the presence of different catalysts (Table 43, entries 18–20).^1113^**T2** was formed in 48% yield in the presence of iodine as the catalyst, while other catalysts afforded **T2** in lower yields. The same group also reported the formation of **T2** from **T1** in 20% yield in the presence of MgCl_2_ as catalyst (Table 43, entry 21).^1114^ Domingo et al. also reported the use of catalytic iodine for this reaction.^1115^ The authors used 2,3-dichloro-5,6-dicyano-1,4-benzoquinone (DDQ) as the hydrogen acceptor instead of using **T1** itself, and achieved an 82% isolated yield of **T2** (Table 43, entry 22). Fraser and Young studied dehydrogenation of different unsaturated molecules using 2,6-di-tBu-4-(diphenylmethylene)cyclohexa-2,5-dien-1-one as a hydrogen acceptor.^1116^ When **T1** was used as the substrate, quantitative conversion to **T2** was achieved (Table 43, entry 23). Stratakis and Stavroulakis used methyl viologen-supported zeolite NY to convert **T1** to *p*-cymene in 60% yield (Table 43, entry 24).^1117^ The authors also reported the use of thionin supported Na-Y zeolite for this transformation.^1118^ Under the same conditions a yield of 29% of **T2** was achieved (Table 43, entry 25). Running the reaction for another 2 h resulted in **T2** being the major product, but no further details about actual yield were provided. Borja-Thomas et al. reported a method for the dehydrogenation of **T1** in a flow reaction using simple TiO_2_ as the catalyst (Table 43, entry 26).^1119^ The yield of **T2** was 65%, while an extra 3% yield of isomers of *p*-cymene was reported. In 2021, Kozhevnikov and co-workers reported a highly efficient process for the conversion of **T1** to **T2** over ZnO/SiO_2_ catalysts calcined at 300 °C.^1120^ The best results (98% yield of **T2**) were achieved when 30% ZnO/SiO_2_ was used (Table 43, entry 27).

Double dehydrogenation of **T1** leads to the formation of 1-methyl-4-(prop-1-en-2-yl)benzene (**T3**). Very few reports describe this catalytic transformation as shown in Table 44. In 2003 Johnstone and co-workers reported dehydrogenation of various hydrocarbons by using acidic catalysts and nitrobenzene as the oxidant.^1121^ Complete conversion of **T1** to **T3** was observed after 12 minutes (Table 44, entry 1). Jaekel and co-workers reported Pd-catalyzed dehydrogenation of **T1** to **T3** in the presence of 2,6-^*t*^Bu_2_pyridine and CuCl_2_.^1099^ The desired product was obtained in 61% yield (Table 44, entry 2). The group of Hailes demonstrated the conversion of **T1** to **T3** by using Pd(OAc)_2_ as the catalyst and over stoichiometric amounts of CuCl_2_ and 2,6-lutidine.^1101^ The desired product was formed in 39% yield at 39% conversion of starting material, which makes this method absolutely selective toward **T3** (Table 44, entry 3).

**Table 44:**
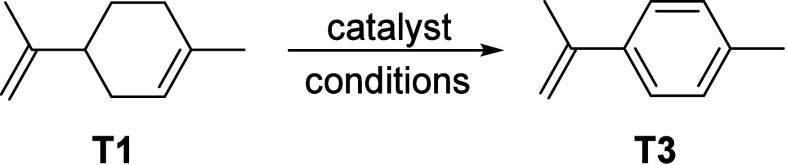
Conversion of Limonene to 1-Methyl-4-isopropenylbenzene

Entry	Catalyst	Catalyst/metal loading	Additional data	*T* (°C)	*t* (h)	Yield of T2 (%)	Ref
1	CF_3_SO_3_H	30 mol%	Excess of C_6_H_5_NO_2_ as oxidant	25	0.2	>99^a^	(1121)
2	Pd(OTFA)_2_	5 mol%	1.5 eq. 2,6-^*t*^Bu_2_pyridine, 2 eq. CuCl_2_, DMF as solvent	80	16	61^b^	(1099)
3	Pd(OAc)_2_	10 mol%	4 eq. CuCl_2_, 9 eq. 2,6-lutidine, DMF as solvent	90	3	39^a^	(1101)

a GC yield without use of internal standard.

b Analyzed by GC using dodecane as a standard.

### 7.2 From Pinene

The conversion of α-pinene (**T4**) to p-cymene (**T2**) has also been extensively investigated. The results are summarized in Table 45. Bazhenov et al. used 50% decationized zeolite Y as catalyst for the conversion of **T4** to **T2**.^1122^ Although the experimental procedure does only provides a range of catalyst loadings, the authors isolated the product in 80% yield by fractional distillation under reduced pressure (Table 45, entry 1). Simakova et al. have shown that full conversion of α-pinene with up to 19% selectivity toward **T2** can be achieved in the presence of 2.2 wt% Au/γ-Al_2_O_3_ catalyst (Table 45, entry 2).^1123^ Golets, Mikkola, and co-workers tested the conversion of **T4** to **T2** at different WHSV values and obtained up to 76% yield of **T2** after 1 h TOS with WHSV = 6.192 h^–1^ (Table 45, entry 3).^1124^ In 2014 Linnekoski and co-workers reported the use of Faujasite Y zeolite (FAU Y) in the aromatization of α-pinene, where a 23% yield of **T2** was achieved with a total yield of cymenes of 34% (Table 45, entry 4).^1125^ The group of Kozhevnikov reported an efficient method for the conversion of **T4** to **T2** over 10% ZnO/SiO_2_ calcined at 300 °C.^1120^ The authors reported an average yield of **T2** of 89% over the course of 6 h (Table 45, entry 5).

**Table 45:**
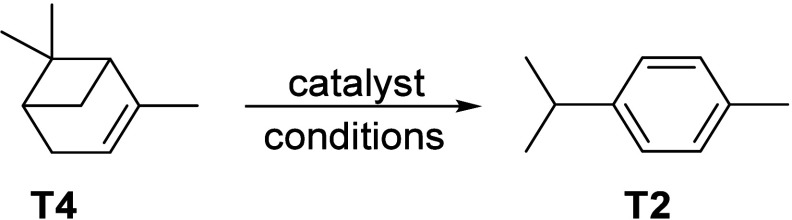
Conversion of α-Pinene to *p*-Cymene

Entry	Catalyst	Catalyst/metal loading	Conditions	*T* (°C)	*t* (h)	Yield of T2 (%) [isolated]	Ref
1	NaAY	1–10 wt%	N_2_ gas	150	2	[80]	(1122)
2	2.2 wt% Au/γ-Al_2_O_3_	0.2 g	0.4 vol.% of pinene in octane, H_2_ atmosphere, SV = 2200 h^–1^	200	0.3	19^a^	(1123)
3	5 wt% Pd-Zn/Al-SBA15	0.25 g	WHSV = 6.192 h^–1^	300	1	76^a^	(1124)
4	FAU Y	6 g	Flow rate of substrate = 10 g/h	300		23^a^	(1125)
5	10% ZnO/SiO_2_	0.4 g	WHSV = 0.020 h^–1^	370	6	89^a^	(1120)

a GC yield without use of internal standard.

**Scheme 163:**
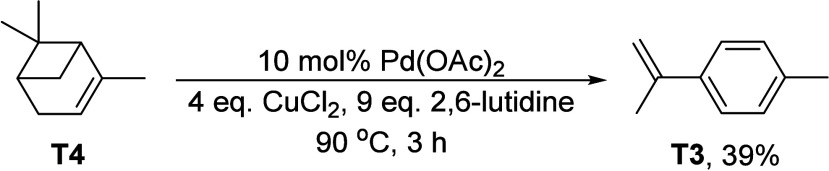
Conversion of α-Pinene to T3

There is only one example of catalytic conversion of α-pinene to **T3** (Scheme 163).^1126^ The authors achieved 39% yield of the desired product by using a Pd catalyst and copper chloride and 2,6-lutidine as additives.

### 7.3 From Other Terpenes

**Scheme 164:**
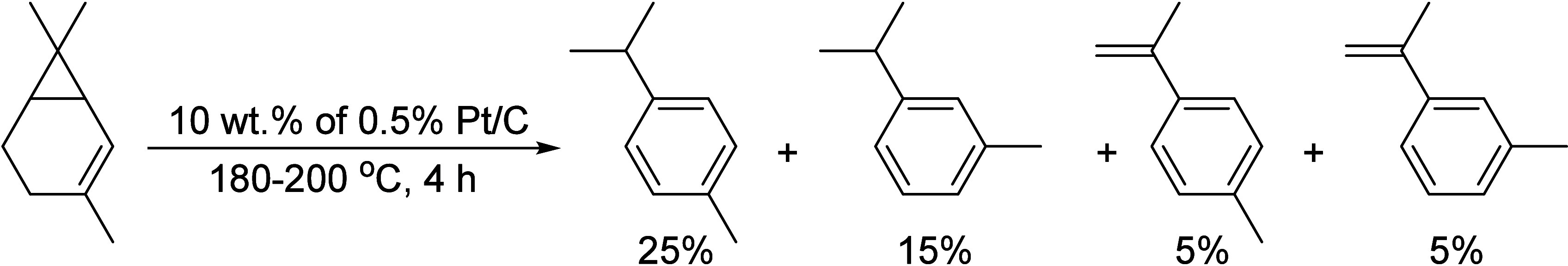
Catalytic Disproportionation of 3-Carene to Aromatic Compounds

Manukov et al. performed the Pt-catalyzed conversion of 3-carene to a mixture of cymenes and isopropenyltoluenes (Scheme 164).^1127^ The authors achieved a 50% yield of aromatic compounds. The remaining 50% correspond to saturated products such as caranes, 2-carene, trimethylcycloheptanes, and menthanes.

**Scheme 165:**
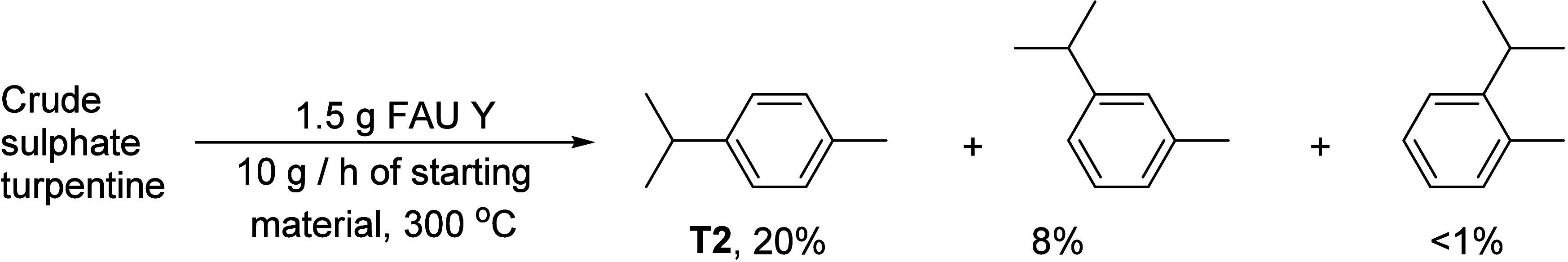
Conversion of Crude Sulfate Turpentine to Cymenes

Linnekoski and co-workers reported aromatization of crude sulphate turpentine, which is a mixture of terpenes, where the main components are α-pinene (65%), 3-carene (24%) and other terpene isomers with MW of 136 (7%).^1125^ Using Faujasite Y as the catalyst, a 28% yield of cymenes was achieved (Scheme 165).

Terpinenes were also converted to **T2**. Table 46 shows catalytic transformations of α-terpinene (**T5**) to **T2**. Barton and Wang investigated oxidation of **T5** in the presence of iron nitrate as the catalyst.^1128^ They achieved a 78% yield of **T2** (Table 46, entry 1). Barton, Smith and co-workers also used the homogeneous manganese catalyst developed by Hage and co-workers for textile bleaching and epoxidation^1129^ for the oxidation of α-terpinene to **T2** by periodic acid and compared it to the iron-catalyzed version of this reaction in the presence of hydrogen peroxide.^1130^ The manganese-catalyzed procedure afforded >95% yield of the product (Table 46, entry 2) while the iron catalyst only afforded 55% yield of the product at 50 mol% catalyst loading (Table 46, entry 3). Reducing the iron loading resulted in a lower yield of the product, but in a higher TON (e.g., 29% yield of **T2** in the presence of 10 mol% of FeCl_3_). Stratakis and Stavroulakis reported the use of viologen supported Na-Y zeolite for the oxidation of α-terpinene.^1117^ After running the reaction for 30 minutes in dry hexane in the dark under inert atmosphere 80% of **T2** was formed (Table 46, entry 4). The authors mentioned that running the reaction for another 30 minutes makes **T2** to be the only product, although no yield was given. The same group used thionin supported Na-Y zeolite for the oxidation of **T5** to **T2**.^1118^ After 30 minutes 36% of **T2** was formed (Table 46, entry 5). The authors also mentioned that leaving the reaction for a further 2 h results in **T2** being the major product, although no further details were provided. Haimov and Neumann reported the use of H_5_PV_2_Mo_10_O_40_ for the oxidation of α-terpinene to **T2**.^1109^ Full selectivity at 100% conversion was achieved under the reaction conditions (Table 46, entry 6). Johnstone and co-workers performed the oxidation of **T5** to **T2** using nitrobenzene as an oxidant and CF_3_SO_3_H as the catalyst (Table 46, entry 7).^1121^ Complete conversion to **T2** was achieved in 5 minutes. The group of Gonsalves studied photocatalytic oxidation of **T5** by oxygen in the presence of supported porphyrins and sunlight.^1131^ The highest yield of **T2** (64%) was achieved in the presence of a Merrifield polymer-supported porphyrin, connected to the support via a -CH_2_-NH-C_12_H_24_-NH-SO_2_-C_6_H_4_- linker (PS1, Table 46, entry 8). The analysis of the product mixture was done by ^1^H NMR after the removal of the catalyst by filtration. The group of Belgsir used an electrocatalytic method with TEMPO as the catalyst for the conversion of **T5** to **T2**.^1132^ The authors obtained an excellent GC yield (96%), and also isolated the product in 63% yield (Table 46, entry 9). Lacombe and co-workers studied the use of photosynthesizers for the oxidation of α-terpinene.^1133^ Use of silica-supported anthraquinone (ANTH-Si) afforded an 80% yield of aromatic compounds (70% of **T2** and 10% of *p*-isopropylbenzaldehyde) under irradiation (Table 46, entry 10). Oliver-Tomas, Renz and Corma reported TiO_2_ catalyzed formation of **T2** from α-terpinene (**T5**).^1119^ Running the reaction in the presence of 2-pentanone as an oxidant improved the yield of **T2** from 68% to 86% (Table 46, entries 11 and 12). Karakhanov et al. prepared Pt and Pd containing phenol-formaldehyde polymers as heterogeneous catalysts for the hydrogenation of terpenes.^1134^ The platinum catalyst (MPF-SO_3_H-Pt-c) appeared to be an efficient dehydrogenation catalyst of **T5** even in the presence of hydrogen pressure, affording a 60% yield of **T2** (Table 46, entry 13). Poliakoff, George and co-workers designed a vortex reactor for thermal and photochemical reactions.^1135^ The goal of the authors was to perform a cycloaddition reaction on α-terpinene with singlet oxygen, generated photocatalytically using Rose Bengal as sensitizer, to obtain ascaridole (the cyclic peroxide). However, at higher rotation speeds of the cylinder up to 24% of **T2** was formed (Table 46, entry 14). Taboonpong and Chavasiri developed a protocol with a recyclable catalyst, which was tested for the aromatization of different cyclic dienes.^1136^ α-Terpinene was converted to **T2** in 47% yield using *t*-BuOOH as oxidant and CuCl_2_ as catalyst in an ionic liquid as solvent (Table 46, entry 15). Fraser and Young performed the acid-catalyzed oxidation of **T5** via hydride transfer in the presence of 2,6-di-tBu-4-(diphenylmethylene)cyclohexa-2,5-dien-1-one as the oxidant resulting in a quantitative yield of **T2** (Table 46, entry 16).^1116^ The group of Jiang studied the photocatalytic oxidation of **T5** to **T2** in the presence of the nickel containing porphyrinic covalent organic framework DhaTph-Ni as the catalyst and oxygen as an oxidant.^1137^ The authors reached a total yield of aromatic compounds of 71%, where **T2** was the major product (62%) (Table 46, entry 17). Tang, Kang, Li and co-workers reported the photocatalytic oxidation of **T5** to **T2** in the presence of copper-bridged tetrakis(4-ethynylphenyl)ethene aggregates (T_4_EPE-Cu_4_)_n_ as catalyst.^1138^ The total yield of aromatic compounds was 45%, with the major aromatic product being **T2** (Table 46, entry 18). Metin and co-workers reported quantitative conversion of α-terpinene to **T2** catalyzed by CuPd nanoparticles that were anchored on mesoporous graphitic carbon nitride (Cu_50_Pd_50_/m-gCN) under neat conditions at 120 °C (Table 46, entry 19).^1107^ After the work-up the authors isolated **T2** in 99% yield. Jing, Duang, and co-workers studied the photo-catalyzed oxidation of **T5** by oxygen in the presence of dye-functionalized indium metal organic frameworks (MOF’s) with irradiation at 455 nm.^1139^ The best results were achieved with a 2-fold interpenetrated In-TPBD-20, where a total yield of aromatics was 85% (83% yield of **T2**, Table 46, entry 20). Zhou and co-workers described electro-catalytic oxidation of **T5** to **T2**.^1140^ Quantitative conversion to the desired product was achieved under the conditions (Table 46, entry 21). Reducing the loading of TEMPO resulted in a slight reduction of the yield.

**Table 46:**
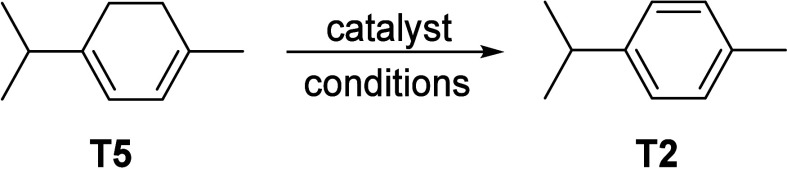
Conversion of α-Terpinene to *p*-Cymene

Entry	Catalyst	Catalyst/metal loading	Conditions	*T* (°C)	*t* (h)	Yield of **T2** (%) [isolated]	Ref
1	Fe(NO_3_)_3_·9H_2_O	10 mol%	30 mol% picolinic acid, 1 equiv ^*t*^Bu-hydroperoxide, acetic acid:pyridine (1:10) as solvent	20	0.5	78^a^	(1128)
2	[Mn^lV^-Mn^lV^ O_3_(tmtacn)_2_]PF_6_^b^	2–5 mol%	H_5_IO_6_ as oxidant, pyridine as solvent	RT	1	>95^c^	(1130)
3	FeCl_3_	50 mol%	H_2_O_2_ as oxidant, pyridine as solvent	0	1	55^c^	(1130)
4	MV^2+^/Na-Y	12 wt%	Dry hexane, argon, dark	RT	0.5	80^d^	(1117)
5	Thionin^e^/Na-Y	120,000 wt%	Hexane as solvent	RT	0.5	36^d^	(1118)
6	H_5_PV_2_Mo_10_O_40_	2 mol%	PEG-200 as solvent, O_2_ gas (2 bar)	100	16	>99^f^	(1109)
7	CF_3_SO_3_H	30 mol%	C_6_H_5_NO_2_ as a solvent and as an oxidant	25	0.08	>99^g^	(1121)
8	PS1	0.0017 mol%	O_2_ gas, CHCl_3_ as solvent, sunlight	RT	8	64^h^	(1131)
9	TEMPO	5 mol%	Electromediated oxidation	RT		96 [63]	(1132)
10	ANTH-Si	3.3 mol%	MeCN as the solvent, O_2_, irradiation λ_max_ = 419 nm	30	1.33	70^g^	(1133)
11	TiO_2_	2.50 g	F = 7.6 g h^–1^, WHSV = 3.04 h^–1^	425	Continuous flow	68^g^	(1119)
12	TiO_2_	2.50 g	F = 7.35 g h^–1^, WHSV = 2.94 h^–1^, 1 equiv of 2-pentanone	425	Continuous flow	86^g^	(1119)
13	MPF-SO_3_H-Pt-c	0.025 mol%	H_2_ gas (40 bar)	300	1	60^j^	(1134)
14	Rose Bengal	2 mol%	0.1 M in EtOH, 0.5 mL min^–1^, LED, air, 4000 rpm	RT	Continuous flow	24^k^	(1135)
15	CuCl_2_	5 mol%	6 eq. ^*t*^BuOOH, 1-hexyl-3-methyl-imidazolium bromide as solvent	RT	0.25	47^a^	(1136)
16	[H(OEt_2_)_2_][BAr^F^_4_]	10 mol%	2,6-di-^*t*^Bu-4-(diphenylmethylene)cyclohexa-2,5-dien-1-one as an oxidant, C_2_H_4_Cl_2_ as a solvent	90	12	>99%^i^	(1116)
17	DhaTph-Ni	73 wt%	O_2_ gas, visible light, MeCN as solvent	RT	6	62^d^	(1137)
18	(T_4_EPE-Cu_4_)_*n*_	76 wt%	Air, λ_max_ = 380 nm, MeCN as solvent	20	2	41	(1138)
19	Cu_50_Pd_50_/m-gCN	3 mol%	Neat	120	12	[99]	(1107)
20	In-TPBD-20	2.5 mol%	O_2_ gas, 455 nm LED, MeCN as solvent	RT	4	83^h^	(1139)
21	TEMPO	20 mol%	Pt cathode and anode, 2,6-lutidine, n-Bu_4_NClO_4_, wet MeCN	RT		99^f^	(1140)

a GC yield using naphthalene as internal standard.

b tmatcn = *N,N′,N″*-trimethyl-1,4,7-triazacyclononane.

c GC yield after a work-up using naphthalene as an internal standard.

d GC yield using n-dodecane as internal standard.

e Thionin = 3,7-diamino-5-phenothiazinium acetate

f GC yield using internal standard without providing details of what the standard is.

g GC yield without using internal standard.

h Determined by 1H NMR without internal standard.

i The yield determined by GC-MS using decane as internal standard.

j GLC yield without using internal standard.

k Determined by ^1^H NMR using biphenyl as internal standard.

Dehydrogenation of γ-terpinene (**T6**) to **T2** is summarized in Table 47. Fox et al. studied photocatalytic oxidation of different dienes in the presence of titanium dioxide.^1141^ This protocol resulted in a 21% yield of **T2** under the reaction conditions shown in Table 1, entry 1. Barton and Wang used Fe(NO_3_)_3_.9H2O as the catalyst for this reaction and T2 was formed in 86% yield (Table 47, entry 2).^1128^ The other product formed during the reaction was 4-isopropylbenzoic acid (14%). Very similar results with slightly better yield were reported by Barton and Chavasiri the same year.^1142^ Barton, Smith and co-workers also tested Mn and Fe catalysts for the oxidation of α-terpinene to T2.^1130^ The Mn-catalyzed procedure required a much lower catalyst loading and afforded an excellent 90% yield of **T2** (Table 47, entry 3). Use of the Fe catalyst resulted in a low 31% yield of the product (Table 47, entry 4), while it required H_2_O_2_ as an oxidant in contrast to periodic acid in case of the manganese-catalyzed protocol. Stratakis and Stavroulakis used viologen supported Na-Y zeolite for the oxidation of α-terpinene.^1117^ A yield of 47% of the desired product (72% selectivity at 65% conversion) was achieved (Table 47, entry 5). Continuing the reaction for another 30 minutes resulted in **T2** being the only product, although no further details were provided. Belgsir and co-workers reported an electrocatalytic method using TEMPO as the catalyst for the conversion of **T6** to **T2**.^1132^ An excellent GC yield of 94% resulting in 60% isolated yield of the product was achieved (Table 47, entry 6). Taboonpong and Chavasiri reported the use of CuCl_2_ as catalyst in acetonitrile or ionic liquids as solvents for the aromatization of cyclic dienes.^1136^ A quantitative yield of **T2** from **T6** was achieved in acetonitrile in just 15 minutes (Table 47, entry 7). If 6 equiv of *tert*-butylperoxide were used instead of 1.5 equiv, the same result was achieved in 1-hexyl-3-methylimidazolium bromide as the solvent. Metin and co-workers obtained a 90% yield of **T2** after a work-up of the reaction mixture obtained according to the reaction conditions in Table 47, entry 8.^1107^ Zhou and co-workers reported electro-catalytic oxidation of **T6** to **T2**.^1140^ An excellent yield of 93% of *p*-cymene was achieved under the reaction conditions shown in Table 47, entry 9.

**Table 47:**
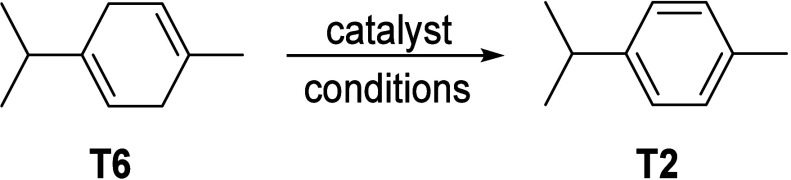
Conversion of γ-Terpinene to *p*-Cymene

Entry	Catalyst	Metal loading	Conditions	*T* (°C)	*t* (h)	Yield of T2 (%)[isolated]	Ref
1	TiO_2_	5–12.5 mol%	MeCN as solvent, λ = 350 nm	15–20	16–48	21^a^	(1141)
2	Fe(NO_3_)_3_·9H_2_O	10 mol%	30 mol% picolinic acid, 1 equiv ^*t*^Bu-hydroperoxide, acetic acid:pyridine (1:10) as solvent	20	1	86^b^	(1128)
3	[Mn_2_O_3_(C_9_H_21_N_3_)_2_] (PF_6_)_2_	2–5 mol%	H_5_IO_6_ as oxidant, pyridine as solvent	RT	3	90^b^	(1130)
4	FeCl_3_	10 mol%	H_2_O_2_ as oxidant, pyridine as solvent	0	1	31^b^	(1130)
5	MV^2+^/Na-Y	12 wt%	Dry hexane, argon, dark	RT	0.5	47^c^	(1117)
6	TEMPO	5 mol%	Electromediated oxidation	RT		94, [60]	(1132)
7	CuCl_2_	5 mol%	1.5 eq. ^*t*^BuOOH, MeCN as solvent	RT	0.25	>99^b^	(1136)
8	Cu_50_Pd_50_/m-gCN	3 mol%	Neat	120	18	90^d^	(1107)
9	TEMPO	20 mol%	Pt cathode and anode, 2,6-lutidine, n-Bu_4_NClO_4_, wet MeCN	RT		93^e^	(1140)

a GLC yield using pentamethylbenzene as an internal standard.

b GC yield using naphthalene as internal standard.

c GC yield using dodecane as internal standard.

d 1H NMR yield after a work-up without use of any standard.

e GC yield using internal standard without providing details of what the standard is.

Overall, naturally occurring terpenes, which are isolated and used in industry, can only be converted to cymenes or 1-methyl-4-isopropenylbenzene, although examples of the latter transformation are rare. Some literature examples provide protocols with excellent and even quantitative isolated yields, although, unfortunately, isolation of the products was rarely reported. In a number of cases *p*-cymene was seen as an undesired side product during other targeted reactions.

## 8 Aromatics from Methane

Methane can serve as a sustainable platform chemical for the production of benzenoid aromatics. For this purpose the renewable supply of methane can be secured through the Sabatier reaction (i.e., methanation) using anthropogenic CO_2_, resulting from domestic and industrial activities and green hydrogen generated from water electrolysis by renewable electricity (power-to-gas concepts).^1143,1144^ On the other hand methane can be generated via the hydrogenation of CO in syngas generated from biomass gassification.^1145^ A third source is via anaerobic fermentation of biomass, which delivers a 50/50 mixture of CH_4_ and CO_2_ (biogas).^1146^ Considering these aspects, the valorization of methane to value-added products such as aromatics, olefins and alcohols is deemed a significant area of research which has been critically discussed in the literature.^1147−1149^ The oxidative coupling of methane has been intensively studied for over 4 decades for the production of C2+ hydrocarbons including aromatics.^1150,1151^ The selectivity of aromatics from this process is, however, very low compared to lower hydrocarbons which are produced in significantly higher yields. The non-oxidative dehydroaromatization of methane was later proposed by several groups in the early nineties of the last century as a possibly more efficient approach for producing benzenoid aromatics form methane with higher aromatics selectivity compared to lower hydrocarbons (e.g., alkanes).^1152^

**Figure 46:**
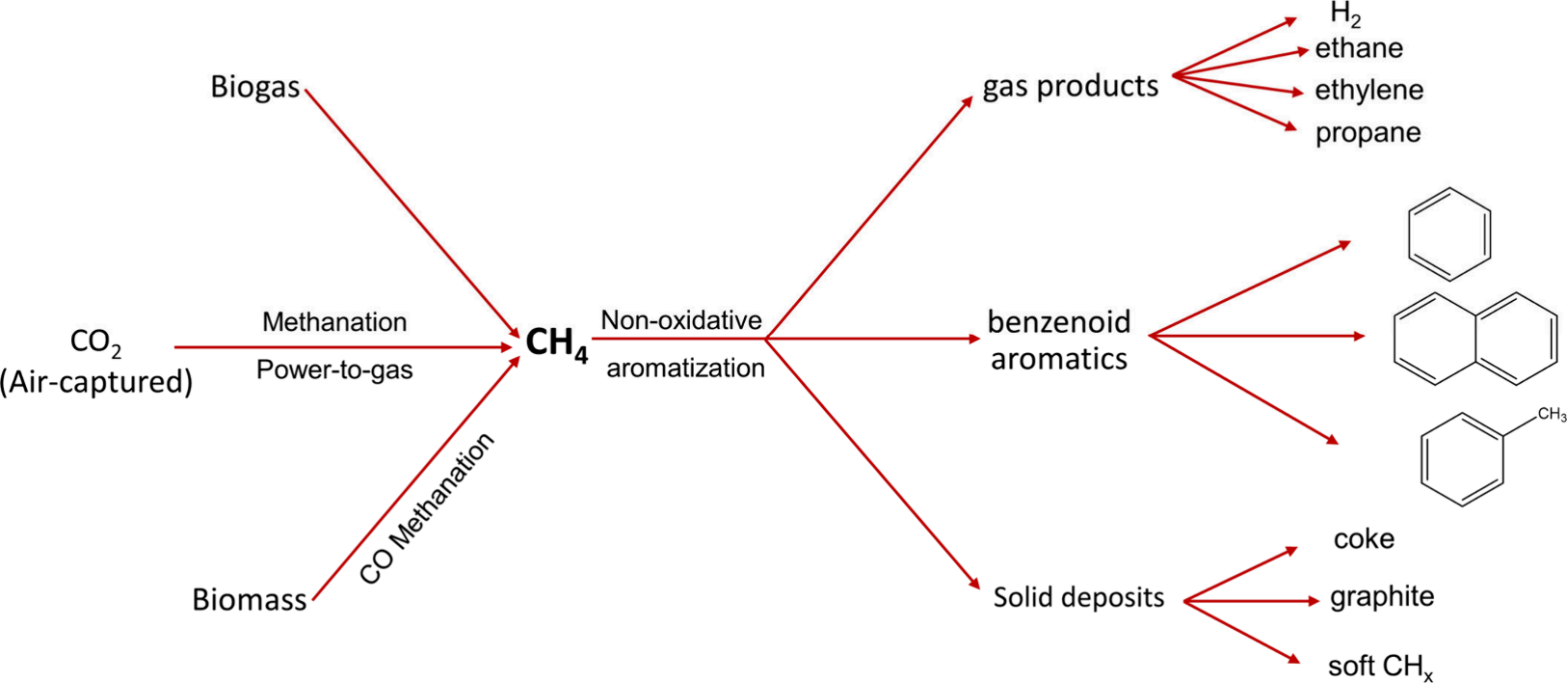
Schematic representation of the possible sources of renewable methane and the products in the non-oxidative conversion of methane above its decomposition temperature (>700 °C).

Methane dehydroaromatization (MDA) is a proposed process for converting methane to value-added aromatics and clean hydrogen as a byproduct from the methane dehydrogenation.^1147,1149,1153^ As an example, the conversion of 6 molecules of methane to benzene (considering for simplicity no other hydrocarbon products forming) would result in the formation of 9 molecules of H_2_ (see eq. 1). This way the successful implementation of the non-oxidative methane dehydroaromatization would make methane, an ideal hydrogen carrier and at the same time a source of basic chemicals (see this concept summarized in Figure 46).
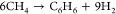
1MDA is, however, a highly endothermic reaction (^D^*H*_r_^s^ = +531 kJ mol^–1^) which is caused by the high activation barrier for breaking the C–H bond in the methane molecule. Thermodynamic numerical analysis studies indicate that formation of aromatics and olefins from non-oxidative activation of methane is favored at elevated temperatures and low reaction pressures.^1154^ Typical temperatures for the MDA reaction are far above 700 °C to achieve measurable methane conversion. The selected working temperature depends decisively on the employed catalysts. Upon reaching a temperature of 700–800 °C the thermodynamically controlled conversion of methane is roughly located in the range between 12 and 17%.^69^ Considering other limitations resulting from deactivation by coke formation under these harsh conditions, several studies over the past 20 years indicated that methane conversion remains far below these thermodynamic limits even upon applying the most active catalysts.^1155−1164^ Note that during the non-oxidative conversion of methane to aromatics other undesired byproducts are expected to form (see scheme in Figure 46, e.g., solid carbon deposits). The main products of this reaction can be divided into three basic categories: (i) gas products including ethane, ethylene, propane, etc.; (ii) solid products including coke and higher hydrocarbons; and (iii) benzenoid aromatics, basically benzene, toluene, and naphthalene.

Standard catalysts which have been widely studied so far for the methane dehydroaromatization are bifunctional catalysts. These catalysts are based on the use of highly oxidizable metals, used as single metal catalysts (arranged according to importance: Mo,^1162,1165−1168^ Fe,^1163,1169−1171^ Zn,^1172−1174^ Mn,^1175^ Re^1176^ and Ga^1177,1178^) or in combination with other metals (e.g., Mo/Fe,^1179−1181^ Mo/Zn,^1181^ Mo/Ru,^1156,1182,1183^ Mo/Pt,^1184^ Mn/W,^1185^ Mo/Zr^1186^) with an acidic support, basically a zeolitic material (arranged according to their importance: H-ZSM-5,^1167,1187,1188^ ZSM-11,^1178^ HMCM-49,^1187,1189^ MCM-22,^1190,1191^ and MFI-based zeolites^1176,1192−1194^), acidic oxides (sulfated zirconia^1195^), or nitrides (e.g., gallium nitrides^1196^).

Among all these materials the combination of MoO_*x*_ with the zeolite H-ZSM-5 showed by far the highest activity toward total methane conversion,^1162,1165−1168^ and more importantly the highest selectivity toward benzenoid aromatics, particularly benzene (on average >45% in the majority of cases). Fe single site or Fe-oxide clusters supported on H-ZSM-5 have also been considered as very promising for this reaction. For quantitative comparison, the catalytic and structural data of the supported Mo catalysts are summarized in Table 48. Results with other metals supported on H-ZSM-5 and other metals including samples promoted by a guest element are also included in Table 48.

**Table 48:** Performance of Catalysts in Methane Dehydroaromatization

Entry	Catalyst (metal loading)	Structural features	Reaction conditions (gas mixture, temperature, space velocity, or flow rate)	Conv (%)	Product sel. (%)	Analysis	Ref
1	Mo/H-ZSM-5 (2.0 wt%)	SiO_2_/Al_2_O_3_ = 25.5	100% CH_4_; 2 g cat.; 1440 ml g^–1^ h^–1^; 700 °C	7.2 (170 min)	100% benzene	MS	(1153)
2	Zn/H-ZSM-5 (2.0 wt%)	SiO_2_/Al_2_O_3_ = 25.5	100% CH_4_; 2 g cat.; 1440 ml g^–1^ h^–1^; 700 °C	3.0 (170 min)	100% benzene	MS	(1153)
3	MoO_3_/ZSM-5 (2.0 wt%)	Si/Al = 55.0	100% CH_4_; 0.5 g cat.; 12 ml min^–1^; 700 °C	4.15 (60 min)	Ethane: 2.92	GC	(1166)
					Ethylene: 5.52		
					Benzene: 36.8		
					Toluene: 2.76		
					Coke: 50.5		
4	Mo/H-ZSM-5 (3.5 wt%)	SiO_2_/Al_2_O_3_ = 50–70	100% CH_4_; 0.5 g cat.; 600 h^–1^; 700 °C	7.5 (60 min)	Ethane: 1.3	GC-FID	(69)
					Ethylene: 2.9		
					Benzene: 89.8		
					Toluene: 6.0		
					Coke: NM		
5	Zn-modified Mo/H-ZSM-5 (3.5wt%)	SiO_2_/Al_2_O_3_ = 50–70	100% CH_4_; 0.5 g cat.; 600 h^–1^; 700 °C	10.9 (60 min)	Ethane: 1.0	GC-FID	(69)
		For Mo/Zn = 0.03 (mol ratio)			Ethylene: 2.9		
					Benzene: 90.0		
					Toluene: 6.7		
					Coke: NM		
6	Fe/H-ZSM-5 (2.0 wt%)	Si/Al = 25	90% CH_4_ /N_2_; 1.0 g cat.; 800 h^–1^; 700 °C	14 (> 240 min)	CO: <1	GC	(1170)
					C2-C3: 2.7		
					Benzene: 48		
					Toluene: 2.5		
					Naphthalene: 6.5		
					Coke: 10% of CH_4_		
7	Mn/H-ZSM-5 (4.0 wt%)	Si/Al = 25	100% CH_4_; 0.5 g cat.; GHSV = 1600 mL h^–1^ g^–1^; 700, 750, and 800 °C	2.1% (700 °C);	78.6% benzene @ 700 °C	GC	(1175)
				4.4%(750 °C)	76.1% at750 °C		
				6.9%(800 °C) [360 min]	75.6% at 800 °C		
8	MO/H-ZSM-5 (0.13 wt% Ru-X wt% Mo; X= 1–3 wt)	Si/Al = 15	90% CH_4_/Ar; 600 °C; 270 cm^3^ h^–1^ g^–1^	6.4% at 600 °C (0.13% Ru–1.5% Mo/H-ZSM-5	65% benzene at 600 °C (for 6.4% CH_4_ conversion)	GC	(1164)
9	Re/H-ZSM-5	Si/Al = 28	16% CH_4_/Ar; 333 mL min^–1^; 700 °C		95.4% (1 min) to 50.5% (60) benzene	GC	(1176)
	Re/CaZSM-5						
	(3.9 / 3.95 wt%)						
10	Mo/MCM-2	Si/Al = 64	90% CH_4_, 2% CO_2_, and 8% Ar; 1500 mL g _cat_^–1^ h^–1^; 720 °C	14.5 (1 min) to 5.5 (100 h)	Benzene: ∼87 to 81%	´GC	(1190)
	(6 wt%)				Naphthalene: ∼7 to 18		
11	Mo/H-ZSM-5	Si/Al = 15	90% CH_4_, 10% N_2_, 15 SCCM; 3200 SCC g_cat_^–1^ h^–1^	10 (1 min) to -4 (360 min	Benzene: ∼60%	GC (GC-Mass)	(1197)
	(6 wt%)				Naphthalene: ∼15–7%		
					Ethylene: ∼0–15%		

These catalysts suffer, however, from a continuous deactivation with time on stream, quantitatively losing about 40–60% of their initial activity within a period of 250 to 350 min.^1198,1199^ Although, the physical reasons underlying the deactivation of these catalysts have been intensively studied over years, a consensus on the dominant mechanism remains elusive. Possible reasons for the deactivation are coke deposition, agglomeration of active MoOx species, or changes in the zeolite matrix (e.g., dealumination). For a full description, see the review by Spivey and Hutchings.^1149^

Interestingly, addition of a catalyst promoter such as Ru was reported to enhance the time-on-stream stability of the catalyst for over 1500 min at temperatures in the range from 600 to 700 °C.^1156^ Ru doping in particular proved beneficial for relatively low temperature applications (CH_4_ aromatization at <700 °C). As an example, limited doping of the Mo/ZSM-5 catalyst by only 0.13 wt% Ru resulted in rather high activity at 600 °C.^1164^ Note that these authors showed that the change of the loading of Mo with fixed Ru loading also had significant impact on activity and selectivity. The highest conversion of 6.4% was obtained for 1.5 wt% Mo with 65% benzene selectivity while the highest selectivity of 82% was recorded with 1 wt% Mo but at a much lower CH_4_ conversion of 2.4% (Table 48, entry 9). Finally, it should be noted that based on the results summarized in Table 48 the reliability of the reported product selectivities is some cases is a matter of discussion and sometimes is not realistic. As an example, in Table 48, entries 1 and 2, a benzene selectivity of 100% contradicts the other reported results. In these two cases mass spectrometry was applied for quantification which is less sensitive to small concentrations of other products formed during the reaction. In the rest of the examples discussed in Table 48 (entries 3–10) instead GC and/or GC-mass measurements were applied which is somewhat more reliable although internal standards were not always used. In none of the cases were products isolated.

Another decisive parameter to control the catalytic performance of these catalysts, and thus the enhancement of the reaction yield and selectivity to aromatics, is the reaction gas composition. Other constituents can be added to the methane such as aliphatic hydrocarbons, hydrogen, or water vapor.^1192,1200−1203^ For example, the cofeeding of ethane with methane was reported to enhance the selectivity toward benzene, although the methane conversion was negatively affected.^1200,1203^ As reported by Ma et al., the rate of benzene formation at 725 °C and a pressure of 3 atm over a 6.0 wt% Mo/H-ZSM-5 (Si/Al = 40) catalyst was enhanced by about 3-fold upon increasing the ethane concentration from 1 to 16%. Activation of methane over Co-Zn/H-ZSM-5 at 600 °C could be enhanced by adding propane to the feed.^1201,1202,1204^ On a 2% Co–2% Zn/H-ZSM-5 catalyst at 600 °C, the controlled variation of the ratio of CH_4_: C_3_H_8_ from 0.4 to 1.0 resulted in a continuous increase of the methane conversion from 26.2% to 36.7%, while at the same time the propane conversion increased from 70.1% to 79.9%. These variations in C1/C3 ratio correlated to an increase of the benzene selectivity from 24.2% to 28.5%. Total aromatics decreased, however, from 90.7 to 88.7 over the same range. As an explanation of the promotional effect of propane on methane activation, Liu et al al.^1202^ suggested that the methane activation step, which is considered as the rate-limiting step, proceeds via a hydrogen-transfer reaction between methane and propene. This example demonstrates the high potential of cofeeding higher hydrocarbons with methane especially for possible low-temperature aromatization. In addition, it demonstrated the need for systematic studies on the impact of reaction gas composition on the catalytic performance of these catalysts.

**Figure 47:**
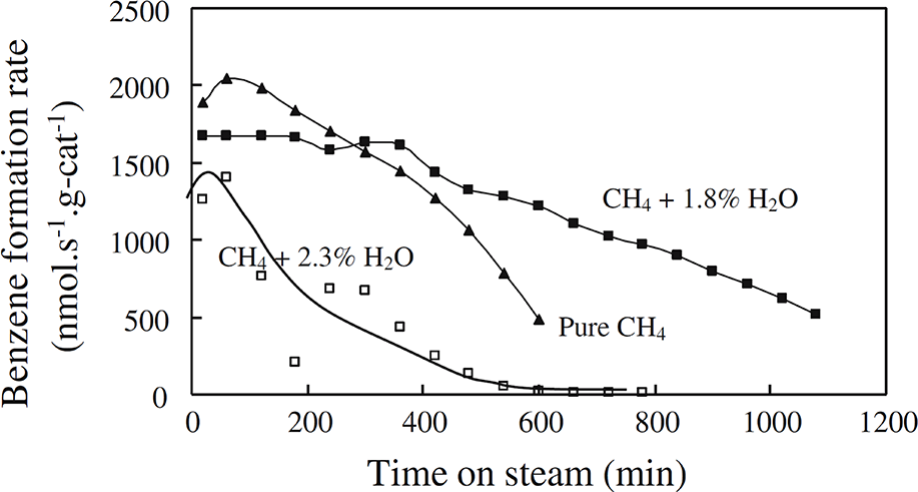
Rate of formation of benzene from methane under continuous-flow conditions in the presence of different amounts of steam at 750 °C over a 6.0 Mo/H-ZSM-5 catalyst. Reproduced with permission from ref (1205). Copyright 2005 Springer.

The influence of steam, and hydrogen cofeeds on the activation of methane to aromatics was also studied by several groups.^1154,1205−1207^ Ma et al. showed that the addition of a controlled amounts of H_2_ (5.3%) and H_2_O (1.8%) to the reaction feed had a positive effect on the stability of the 6.0 Mo/H-ZSM-5 catalysts during methane dehydroaromatization at 750 °C.^1205^ Based on these findings it was inferred that the cofeeding of H_2_O and H_2_ with methane has a synergistic effect on the catalyst stability, while the cofeeding of either H_2_ or steam separately with methane apparently had a negative effect on the catalyst stability. Exceeding a steam amount of more than 1.8% in the feed gas results in an accelerated deactivation of the catalyst (based on the decay of benzene yield with time on stream) compared to a steam-free methane feed gas (Figure 47). The presence of H_2_/H_2_O in the feed gas was found to suppress the buildup of coke which prevents the deactivation of these catalysts during the reaction. Recent findings reported by Çaǧlayan et al.^1207^ focused on the understanding of the impact of steam on the coke formation on 2 wt% Mo/H-ZSM-5 catalysts employing an array of characterization methods including spectroscopy (EPR, NMR, and XPS), high-resolution electron microscopy, porosity measurements, and X-ray diffraction. On 2 wt% Mo/H-ZSM-5, the addition of steam to methane during methane activation at 725 °C activated a steam reforming pathway of both the building coke and the methane, in parallel to the main course MDA reaction. This seems a plausible explanation for the coke decomposition in the presence of steam.

**Figure 48:**
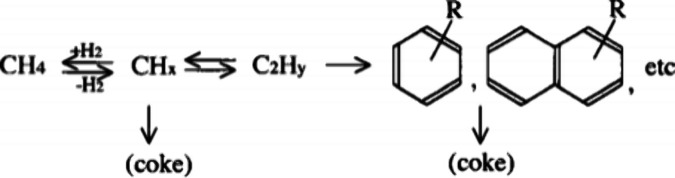
Proposed bifunctional mechanism of the non-oxidative dehydroaromatization of methane to aromatic products such as benzene and naphthalene via the surface hydrocarbon species on Mo/H-ZSM-5 catalyst. Reproduced with permission from ref (1157). Copyright Elsevier 1999.

Despite intensive studies of these metal supported zeolites, in particular the investigations on Mo/H-ZSM-5, the molecular reaction mechanism and the origin of the deactivation of these catalysts is not fully resolved. There is, however, consensus on a bigger picture of the reaction scheme which had been proposed earlier by Ohnishi et al.^1157^ These authors proposed that the C–H bond of methane is first activated on the MoO_*x*_ sites to form a CH*x* pool of different species and also even carbides (see scheme in Figure 48). These species can oligomerize in a subsequent step on the acid sites of the H-ZSMS-5 to higher hydrocarbons and eventually to benzenoids. Coke formation would build up in both steps. The elementary steps of this scheme and the underlying active sites are, however, controversially debated. As an example, the nature of the coke species and their reactivity is a matter of controversy. It may be further hydrogenated and participate in the reaction or act as catalyst poisoning species, depending on their stability (see further discussion in Refs.^1147,1149^).

In recent studies Abdel-Mageed and co-workers shed light on the nature of the active species in these Mo/H-ZSM-5 catalysts and the origin of the continuous deactivation with time-on-stream. In their approach they employed a combination of near-ambient pressure X-ray photoelectron and X-ray absorption spectroscopic measurements (NAP-XPS and XAS),^1197,1208^ MAS NMR spectroscopy,^1197,1209^ together with high-resolution electron microscopy to probe the different structural and electronic properties of the Mo species and the acid / base properties. Their results indicated that there is basically an irreversible agglomeration of MoOx during the reaction. In addition, there is little correlation between the deactivation of the catalyst and the loss (changes) in the concentration of Brønsted acid, which in contrast can be explained by the accumulation of coke at the external openings of the H-ZSM-5 framework. Finally, it was inferred that the active sites are comprised of highly dispersed mononuclear and / or tiny subnanometer oxy (oxycarbidic) clusters distributed uniformly on the micropores on (or in the vicinity of) the Brønsted acid sites.

In view of the high reaction temperatures, the low conversions, the low selectivities and the poor catalyst lifetime methane to aromatics does not seem feasible for large scale production.

## 9 Aromatics from Renewable Methanol

Fossil methanol is produced via aqueous reforming of methane to produce syngas. The syngas is then converted in a process catalyzed by copper–zinc oxide–aluminum oxide catalysts to methanol. Industrial production plants have capacities of up to 1.5 million metric tons of methanol per year.^1210^ Renewable methanol can be produced from a number of different resources.^1211^ Biogas is produced from the fermentation of renewable biogenic raw materials, mostly agro waste. It is a roughly 50/50 mixture of CH_4_ and CO_2_ and provides a large quantity of the total amount of sustainably available carbon.^1212^ Synthesis gas can be produced by reforming biogas or gasifying and reforming lignocellulose or by reduction of CO_2_. However, CO_2_ can also be directly hydrogenated to methanol.^1213,1214^ The source of CO_2_ can be either a cleaned industrial flue gas or concentrated atmospheric CO_2_^1215^ One of the best-known existing plants that produces methanol directly from CO_2_ is that of Carbon Recycling International (CRI) Iceland. In 2014, it produced 4000 t of methanol per year based on the process developed by Olah.^1216^ This plant demonstrated the possibility of upscaling, which is now being realized in a capacity of 110,000 t per year in the Shunli plant in Anyang, Henan province, China.^1217^

Another possible route based on biomass is via the OxFa process where lignocellulose is oxidized to formic acid (liquid phase),^1218^ which in turn can be reduced to methanol.

The production cost of bio-methanol is estimated between 1.5 and 4 times higher than the cost of natural gas-based methanol, which at current fossil fuel prices ranges from €200/t to €250/t.

In this section the conversion of methanol to aromatics (MTA) is reviewed. One earlier review was found on this topic.^1219^

### 9.1 Catalysts

All research groups used mainly ZSM-5 as the catalyst to produce benzene, toluene and xylene (BTX) from methanol in relatively high yields. The authors applied different ways to modify the ZSM-5 to decrease its acidity, which is generally seen as the main origin of coke formation, in order to prolongate the catalyst lifetime. Several different metals such as Ga, Zn, Ag, Cu, and Ni have been used in preparation of ZSM-5 with different crystallite size and pore sizes by applying various methods of preparation such as dealumination, wet impregnation, ion exchange, ball milling, and hydrothermal treatment as will be discussed below. Generally, the reaction was performed in fixed-bed reactors except in rare cases where micro reactors were used.

The effect of preparing zeolites with meso pores to prolongate the lifetime of the catalyst, increase the reaction rate and the selectivity to aromatics has been investigated by several groups. According to the literature, H-ZSM-5 zeolites have their strongly acidic sites (bridging hydroxyl groups) in the internal cavities of the zeolites. Therefore, Asghari et al. prepared a hierarchical H-ZSM-5 from natural kaolin with different pore sizes and Si/Al ratios. The dealuminated ZSM-5 with a Si/Al ratio of 41.6 exhibited superior methanol conversion (over 100 h up to 90%) and BTX selectivity (20%) in the methanol conversion to aromatic hydrocarbon products at 390 °C (Table 49, entry 1).^1220^ The productivity of BTX was found to be 0.8 g_BTX_/(g_cat._.h).

**Table 49:** Methanol to Aromatics: Catalytic results on zeolites with different pore size, crystallite size and acidity

								Selectivity (%)			
Entry	Catalyst (Si/Al ratio)	WHSV (h^–1^)^b^	Reactor	TOS (h)	*T* ( °C)	Analytic method	Selectivity to aromatics (C%)	Benzene	Toluene	Xylene	Ref
1	ZSM-5 (41.6)^a^	10	Fixed-bed	100	390	GC-MS	79	0.5	No data	18.8	(1220)
2	Si@Z5-Na + TP^c^	3.16	Fixed-bed	3	400	GC	69.5	11.9	6.4	21.3	(1221)
3	H-ZSM-5-A (Al 15)	1.5	Fixed-bed	6	450	GC	59.5^d^	5.7	25	28.8	(1222)
4	ZSM-5/NH_3_·H_2_O	1.5	Fixed-bed	120	380	GC	40.3^d^	–	–	–	(1223)
5	NSHZ	1.2	Fixed-bed	100	400	GC	23^d^	–	–	–	(1224)
6	ZSM-5/SAPO-34	1.5	Fixed-bed	12	450	GC	34.3	9.1	11.6	13.5	(1225)

a Hierarchical ZSM-5.

b WHSV = weight hour space velocity.

c Modified H-ZSM-5 with NaOH and TPAOH.

d BTX selectivity.

Chunhui et al. used an ingenious protective desiliconization method with a mixed solution of sodium hydroxide and tetrapropylammonium hydroxide (TPAOH) as a desiliconization agent and silica to create hierarchical porous systems without destruction of the main structure of the molecular sieve. This modification leads to the formation of Si@Z5-Na+TP with a mesoporous volume of 0.38 cm^3^·g^–1^ and strongly acidic centers of 0.341 mmol g^–1^. The selectivity to BTX in aromatics was 63.32% at 100% methanol conversion (Table 49, entry 2). The catalyst was stable over 110 h and the conversion of methanol remained 100% over 80h then it decreased to 90% after prolonged TOS. After 100 h, the strongly acidic sites were covered by carbon deposition and only the weakly acidic sites were exposed on the catalyst surface, which is the active site to form light olefins. Thus, the selectivity to light olefins (C_1_–C_5_) was sharply increased and the selectivity to aromatics largely decreased accordingly.^1221^ These results were higher compared to what was reported by Jin et al., who prepared hollow ZSM-5 catalysts using the treatment with NaOH and TPAOH. However, use of the H-ZSM-5-A (treated with NaOH) resulted in a higher BTX selectivity (above 40%) than that of the HZSM5-T (treated with TPAOH) until TOS of 18 h (Table 49 entry 3). The results were explained to be due to the thinner shell and larger mesopore volumes of the H-ZSM-5-A catalyst, which promoted more primary products to enter into the cavity which were converted to BTX.^1222^

Feng et al. studied the effect of weak base modification (NaHCO_3_, Na_2_CO_3_ and ammonia solution) on the catalytic performance of ZSM-5 in the conversion of methanol to aromatics. Due to the treatment of ZSM-5 with ammonia (ZSM-5/NH_3_·H_2_O) the Brønsted to Lewis ratio (B/L= 7.35) slightly increased but the amount of acid sites decreased, resulting in the formation of a pore structure with micropores and mesopores, which contributed to improving the diffusion of reactants and products, giving a methanol conversion above 80% over 120h and a BTX selectivity of 40.3% (Table 49 entry 4).^1223^

Jia et al. prepared nanocrystalline self-assembled hierarchical ZSM-5 zeolite microspheres (NSHZ) prepared by a simple hydrothermal synthesis in the presence of 3-glycidoxypropyltrimethoxysilane (KH-560). Use of the NSHZ catalyst resulted in a conversion 95% over 100h which decreased to 80% after 136 h. The yield of BTX in hydrocarbons reached 36.6%, even after 16 h. However, the selectivity to BTX was only 23% over 65 h which is low compared to the previous studies (Table 49, entry 5).^1224^

Jin also prepared a series of core-shell ZSM-5/SAPO-34 composite catalysts with different molar ratios (0.5, 1, 2) using a hydrothermal method. The highest selectivity of 34.3% of BTX was obtained over the core-shell ZSM-5/SAPO-34 catalyst with a molar ratio of 2 due to the hierarchical structure and moderate acid sites (Table 49, entry 6). The activity decreased within 12 h TOS.^1225^

### 9.2 Effect of Metal Addition and Acidity

The effect of metal addition (mostly Zn and Ga) to ZSM-5 was investigated extensively. Zn and Ga were chosen due to their Lewis acidity. Niu et al. studied different parameters; catalyst preparation methods and the influence of the crystallite size on the catalytic conversion of methanol to BTX. The results indicated that the way ZnO is introduced to ZSM-5 via impregnation (IM), ion exchange (IE), physical mixing (PM) and direct synthesis (DS) forms different ZnO species according to the preparation method applied. In their work they concluded the following: the introduction of zinc species to H-ZSM-5 is effective in enhancing the aromatization activity for MTA through two approaches: on the one hand, ZnOH^+^ species are formed, which are active for the dehydrogenation of light hydrocarbons; on the other hand, zinc cations may reduce the Brønsted acid sites, which is helpful in suppressing the formation of alkanes by inhibiting the hydrogen transfer reaction. The enhancement of the selectivity to aromatics is linearly related to the amount of ZnOH^+^ species in the Zn-containing H-ZSM-5 zeolites, no matter what method is used to introduce the zinc species. Zn(IE)/ZSM-5 prepared by ion exchange is provided with the highest fraction of surface ZnOH^+^ species and gives the highest selectivity to aromatics (46.9%) during the first 12.5h TOS at methanol complete conversion.^1226^ (Table 50, entry 1). Additionally, it was verified that a linear correlation is observed between the crystallite size, the amount of ZnOH^+^ species and the selectivity to aromatics. As a result, small crystal Zn/H-ZSM-5 (0.25 μm) with large portion of ZnOH^+^ species exhibits high selectivity to aromatics (46%) and long lifetime (80h) with 99% methanol conversion (Table 50, entry 2).^1227^ Shen et al. prepared ZnO-containing MFI zeolite catalysts with bimodal and trimodal hierarchical pore structures. It was found that alkaline treatment favors the formation of large ZnO particles while fluoride enhances the ZnO dispersion. The combination of alkaline and fluoride treatments resulted in a trimodal pore structure which affects the dispersion of the ZnO particles. The increase in pore hierarchy suppressed the coke deposition inside the micropores and increased the coke tolerance. Thus, the BTX selectivity was 62% at complete methanol conversion (Table 50, entry 3). Aromatics may be formed via the hydrogen-transfer route over the H-ZSM-5 catalyst without ZnO modification or with large ZnO particles.^1228^ In another approach, nano-sized H-ZSM-5 zeolites (NZ2, NZ3 and NZ4 catalysts) were modified by adding different amounts of ZnSiF_6_ to lower the total amount of acidic centers. The results revealed that, the amount of Lewis acid sites (L acid sites) (^⊖^ZO···H···O–Zn^⊕^ species) increased, particularly for NZ3, whereas the amount of Brønsted acid sites (B acid sites) obviously decreased with the introduction of the zinc species. The new Zn-Lewis acid sites are active for the dehydroaromatization. This explains why use of NZ3 results in complete methanol conversion in the first 174 h, and then gradually declines to 39% after 234 h TOS. The highest selectivity to BTX over the NZ3 catalyst was 48% (20h), then drops to 17% at 200 h TOS at 400 °C. The effect of temperature was also studied and the highest selectivity to BTX (51%) was achieved at 425 °C on NZ3^1229^ (Table 50, entry 4).

**Table 50:** Methanol to Aromatics: Catalytic Results on Zeolites Modified with Various Metals

								Selectivity (%)^j^			
Entry	Catalyst (Si/Al ratio)	WHSV (h^–1^)^b^	Reactor	TOS (h)	*T* ( °C)	Analytical method	BTX selectivity (C%)	Benzene	Toluene	Xylene	Ref
1	5% Zn(IE)/ZSM-5 (45)	3.2	Fixed-bed	12.5	390	GC	46.9^e^	–	–	–	(1226)
2	5% Zn/HZ-0.25 (45)	3.2	Fixed-bed	12.5	390	GC	46.0^e^	–	–	–	(1227)
3	0.2% ZnO/H-ZSM-5 (25)^a^	25^c^	Fixed-bed	2	460	GC	62	9.3	34.0	19.0	(1228)
4	NZ3 (51.6)^a^	0.8	Fixed-bed	6	400	GC	48.3	4.3	16.3	27.6	(1229)
5	2% Zn@ZSM-5^a^	1	Fixed-bed		430	GC	65^e^	–	–	–	(1230)
6	0.8% Zn-SH-H-ZSM-5 (25)	1	Fixed-bed	1	440	GC	54.5^f^,^g^	8.4	0.50	18	(1231)
7	H-GaMFI (Si/Ga = 33)		Fixed-bed	–	600	GC	–	12	50	39	(1232)
8	1% Ga-ZSM-5 (11.1)	2.4	Fixed-bed		500	GC	60^e^	–	–	–	(1233)
9	1.5% Mg–1% Zn/ZSM-5 (30)	1.0	Fixed-bed	0.5	460	GC	57^e^	14.4	17.8	21.9	(1234)
10	0.8% Zn/0.6% La/H-ZSM-5 (50)		Fixed-bed	4	437	GC	56.6	4.7	22.9	29.0	(1235)
11	2% Ga/ZSM-5 (25)	5.3	Fixed-bed	3	450	GC	20.0 (3.2 g_carbon_/g_catalyst_)	–	–	–	(1236)
12	0.02% Ca 2% Ga/ZSM-5 (25)	5.3	Fixed-bed	3	450	GC	17.8 (4 g_carbon_/g_catalyst_)	–	–	–	(1236)
13	0.75% Zn 1% PHZ (100)	2	Fixed-bed	1	430	GC	84.7^h^	4.8^h^	22.4^h^	23.4^h^	(1237)
14	1% Zn (2% P)ZSM-5 (30)	4.7	Fixed-bed	13	480	GC	60.0^h^	2.0^h^	20^h^	30^h^	(1238)
15	Zn-2P/H-ZSM-5^i^	0.7	Fixed-bed	6	400	GC	46.8^h^	3.5^h^	16.3^h^	27.0^h^	(1239)
16	3Zn/ZSM-5	1.5	Fixed-bed	240	430	GC	<10^h^	1.0^g^	3.0^g^	7^g^	(1240)
17	1%Ag/ZSM-5 (3 0)	0.32	Fixed-bed	12	450	GC	21.5^h^	2.7^h^	10.2^h^	17.6^h^	(1240)
18	2% Cr 1% Zn/HZ (50)	1.2^d^	Fixed-bed	10	430	GC	45.5^g^	–	–	–	(1241)
19	1%Sn1%Zn/ZSM-5 (50)	0.8	Fixed-bed	0.5	450	GC	64.1^g^	8.0	29.7	26.3	(1242)
20	5% Mo_2_C/ZSM-5(80)	1.1	Fixed-bed	75	500	GC	62.8^e^^,^^h^	2.5	9.0	22.3	(1243)
21	Zn-ZSM5 Si-Al15-32Zn	5	Fixed-bed	20	400	GC	18	1	5	12	(1244)

a Hierarchical ZSM-5.

b WHSV (weight hour space velocity).

c Flow rate in mL/min.

d Liquid hour space velocity.

e Aromatic selectivity.

f Total weight/volume.

g BTX yield.

h BTX selectivity calculated as relative wt%.

i Nanosized zeolites modified with 0.05 mol/L of ZnSiF_6_·6H_2_O.

j Benzene (B), Toluene (T), xylene (X)

In order to synthesize a mesoporous Zn-ZSM-5 through alkali treatment Liu et al. introduced Zn to the hierarchical ZSM-5 and this catalyst was mixed with SiO_2_-sol and shaped to be (Zn@ZSM-5). Zn@ZSM-5 led to dramatic increase in aromatics selectivity to reach 65%, but also suffered a tremendous decrease in lifetime to 40 h compared to non-modified H-ZSM-5 (140 h). However, dry gel conversion benefits the catalytic performance, as over Zn@ZSM-5 the higher portion of ZnOH^+^ which was also found by Niu et al. also contributed to the increase in aromatics selectivity as it improved the hydrogen transfer/dehydrogenation properties of the catalyst, which benefit the aromatics selectivity (Table 50, entry 5).^1227,1230^

Gong et al. tried to introduce new surface Lewis acid sites by preparing a Zn-modified nanosheet-H-ZSM-5 zeolite modified with 0.8% Zn (Zn-SH-H-ZSM-5) (Table 50, entry 6). A BTX selectivity of 54% was achieved at 440 °C at complete conversion.^1231^ Xylenes were formed with a selectivity of 18% over Zn-SH-H-ZSM-5. These data indicate that xylene can be formed much more easily over the nanosheet samples with much shortened diffusion-reaction path compared to other similar aromatic compounds. Li et al. have pointed out that *p*-xylene (p-X) may be preferentially generated from the dehydrogenation and hydrogen transfer of a C_8_ cyclic olefin intermediate formed by oligomerization of light olefins (butylene, ethylene) and subsequent cyclization in the MTA reaction. Then, a part of *p*-X is converted into *m*- and *o*-xylene through isomerization, while benzene and toluene are produced by the dealkylation of xylene at 400 °C.^1245^

Zn is well known to sublimate at high temperatures, which limits its utilization. Instead, Gallium, due to its acidity, can be used as promotor. Choudhary and Kinage noticed that the conversion of methanol over H-gallosilicate (H-GaMFI) is influenced strongly by the Si/Ga ratio, degree of H^+^ exchange, calcination temperature, and hydrothermal treatments of the zeolite at 600 °C. Using the best ratio, Si/Ga = 33 (X = 100%), the distribution of the aromatics (toluene, xylene, and benzene) was 50, 39, and 12%, respectively (Table 50, entry 7).^1232^ Lai et al. investigated a series of desilicated H-ZSM-5 catalysts with 1 wt% Ga, in MTA. A moderate alkalinity (NaOH = 0.05 M) in the Si extraction resulted in a maximum aromatics selectivity of 60% at 500 °C. According to Lai, the enhancement of the microporosity increased the number of (GaO)^+^ Brønsted acid sites and also leads to an increase in the outward diffusion of products from the ZSM-5 matrix leading to high aromatic yields (60%). Confined micropores at 0.55 nm was found to be the optimum to improve *o*- and *m*-xylene selectivity and further increased aromatics yields^1233^ (Table 50, entry 8). Liu et al. also studied the effect of the Ga loading (1–3 wt%) supported on ZSM-5. The highest BTX yield was 24% after 3 h TOS with 2.5% wt% Ga loading at 100% methanol conversion (Table 50, entry 11). They found that increasing the Ga content higher than 2% resulted in the agglomeration of Ga and a decrease in the yield of BTX.^1236^

Other authors studied the addition of different basic promotors to adjust the acidity and to modify the pore size to avoid blocking due to coking by adding basic oxides such as MgO, CaO and La_2_O_3_ to the Zn. Li et al. used 1.5% Mg modification which selectively reduced the density of the strongly acidic sites in the channels of Zn–Si-H-ZSM-5, effectively improving the catalyst stability. The selectivity to aromatics was 57% and the highest selectivity to xylene was 21.9% at complete conversion (460 °C and WHSV of 1 h^–1^) (Table 50, entry 9). The stability of the catalyst Mg–Zn–Si-H-ZSM-5 increased (12h TOS complete conversion of methanol) compared to the modified Zn catalyst (2h 10% methanol conversion).^1234^ Addition of La without Zn did not improve H-ZSM-5 for the MTA reaction, but in combination with Zn a 0.8% Zn/0.6% La/H-ZSM-5 catalyst showed high BTX selectivity of 56.6% compared to Zn/H-ZSM-5 catalyst at 437 °C and WHSV=0.8 h^–1^ (Table 50, entry 10).^1235^ Addition of minute amounts of Ca (0.02%) as a promotor to Ga/H-ZSM-5 proved to increase the total output from 16 to 23 g_carbon_/g_catalyst_ and the BTX increased also from 3 to 4 g_carbon_/g_catalyst_ (Table 50, entry 12)_._^1236^

It is generally accepted that phosphorus inhibits the dealumination of zeolites, which, in turn, increases their hydrothermal stability and catalytic selectivity. Thus, Qiao et al. examined the effect of adding phosphorus species as additives to a Zn/H-ZSM-5 zeolite and found excellent selectivity (85%) to BTX in the aromatics fraction at complete conversion of methanol at 430 °C (Table 50, entry 13). The catalyst showed high stability as it was possible to regenerate it *in situ* for 5 runs, each run is for 4h TOS, simulating a fluidized bed reactor.^1237^ Li et al. also found that modifying H-ZSM-5 with Zn and P 1%Zn(2%P)-30 led to the enhancement of the BTX selectivity (60%) (Table 50, entry 14). They explained this by assuming that both Zn and P decrease the acidic strength and increase the Lewis/Brønsted acid ratio of H-ZSM-5, which is beneficial for the aromatization of methanol rather than the hydrogen transfer reaction.^1238^

Similar results were found by Jai et al., who modified H-ZSM-5 using both ZnSiF_6_H_2_O and H_3_PO_4_. At the beginning of the reaction, the highest conversions of methanol over the 2P/H-ZSM-5 catalyst was 75.26% which decreased to 70% after 462 h TOS. The BTX selectivity was 46% during the first 6 h (Table 50, entry 15) but reduced to 19.4% after 222 h. In the presence of Zn and P, the BTX selectivity declined but then remained stable at 20% over 330 TOS. The same conclusion was reached as Qiao et al.: the main role of P and Zn is to decrease the Brønsted acid sites and increase the Lewis /Brønsted ratio which had impact on the selectivity of the BTX.^1239^

Several papers were published on the modification of ZSM-5 using transition metals such as Zn, Cu, Ag, Pd, Ir, Ru and Ni. Ji et al. found that from the elements Cu, Ag and Zn, 3 wt% Zn-modified ZSM-5 showed the highest catalytic performance (100% methanol conversion and a BTX selectivity of around 10%) and stability over 11 days at 430 °C and WHSV = 1.5 h^–1^ (Table 50, entry 16).^1240^ Conte et al. studied the effect of impregnating ZSM-5 with precious and non-precious metals including Ag, Cu, Ni, Pd, Ir, and Ru. Precious metals did not result in enhancement of the selectivity toward aromatics but addition of Ag enhanced the selectivity to C_6_–C_8_ (21.5%) with the highest product distribution of 14% of p-X (Table 50, entry 17).^1240^ Liu et al. studied the effect of the addition of a second metal (Zr, Ce, Mo, and Cr) to Zn-modified ZSM-5 and found that addition of Cr resulted in the highest BTX yield of 45.5% (Table 50, entry 18) at 100% methanol conversion. Hence, the effect of Cr loading was examined, and the optimum was 2% Cr to obtain the highest BTX yield (51%). Unfortunately, addition of Cr reduced the stability on TOS (22h) compared to non-modified ZSM-5 (32h) but it increased the BTX yield.^1241^ Xin et al., investigating the effect of adding Sn as a promotor to Zn on H-ZSM-5, found complete methanol conversion over 28 h but on addition of 2% Zn the stability of the catalyst decreased, and conversion of methanol decreased to 72% in 8h. Addition of Sn to Zn prolongates the catalyst’s lifetime to 24 h but then it deactivates. This in turn has an impact on the BTX yield (64%) as 1% Sn added to Zn in 30 min and decreased to 45% in 12 h (Table 50, entry 19). Sn was thought tyo play a role in accelerating the dehydrogenation of lower alkanes to olefins.^1242^

Mo is well known as a promotor for the aromatization of methane; therefore, Barthos et al. supported Mo_2_C on ZSM-5 with 2 and 10 wt% with various S/Al ratios (30, 80, and 280). The sample with 5% Mo_2_C/ZSM-5 (80) showed the highest yield of aromatics (63%) with the highest content of xylenes (22.3%) compared to other catalysts at 500 °C at complete methanol conversion (Table 50, entry 20). It was assumed that Mo_2_C accelerates the decomposition of methanol to CO, H_2_ and CH_4_ when using SiO_2_ as a support. The optimum amount of Mo_2_C was 5% as further increase of Mo_2_C decreased the ZSM-5 acidity which lowered aromatics selectivity.^1243^

Pinilla-Herrero et al. systematically investigated the effect of the Zn/Al ratio of the Zn-ZSM-5 catalytic systems on the selectivity to aromatics and found that high Zn/Al ratios enhance dehydrogenation vs hydrogen transfer reactions in methanol conversion to aromatics.^1244^

According to these studies it seems difficult to obtain an active and stable catalyst for the synthesis of aromatics from methanol. Therefore, Shao et al. prepared different zeolites with a high Si/Al ratio 220 (HS/Z5) to convert methanol to light alkenes which can be converted to aromatics on a second catalyst bed loaded with metal-modified zeolites with high Si/Al ratio 30 (Zn/Z5). The effect of the ratio of the catalysts added was also studied so 1HS/Z5 /1Zn/Z5 means 1 g of the catalyst HS/Z5 was added in the upper stream and 1 g of Zn/S5 was added in the downstream. The results showed that using this dual bed principle the catalytic lifetime of an individual catalyst (2HS/Z5) is indeed longer, but the hydrocarbon yield is lower (5.4%) than the dual bed (11.5%). All the catalysts showed complete conversion of methanol. Interestingly, the BTX selectivity in aromatics reached 71.7% for this two-step method using 1HS/Z5 /1Zn/Z5 catalyst (Table 51, entry 1).^1246^ By changing the catalysts amounts it was possible to increase the yield of hydrocarbons to 19.7% on 0.25HS/Z5-1.75Zn/Z5 but at the expense of the lifetime of the catalyst (40 h). Fu et al. examined the same catalysts but with different catalysts ratios: 10ZnZ30, 5Zn220/5ZnZ30, 8Zn220/2ZnZ30, 8.5Zn220/1.5ZnZ30, 9Zn220/1ZnZ30, and 10Z220. Rational mixing of two kinds of catalysts (8Zn220/2ZnZ30) led to an increase in aromatic selectivity to 39.9% and the selectivity of BTX to 64% in total and 22.5% BTX in aromatics (Table 51, entry 2). Nevertheless, 9Zn220/1ZnZ30 showed the longest lifetime (219h).^1247^ The same group examined different acidic properties of ZSM-5 for the aromatization of light hydrocarbons. Zeolites with different Si/Al ratios in the range of 30–70 were used for alkenes production from methanol and Zn-ZSM-5 (70) was used for the aromatization of the alkenes. Decreasing acid density by increasing SiO_2_/Al_2_O_3_ ratios from 36 to 70 (0.25HZ/1.75LZ70) decelerated coke formation and significantly extended the catalytic lifetime from 46.5 to 190.8 h with a BTX selectivity of 23% in aromatics at complete methanol conversion (Table 51, entry 3). Further modification with low Zn loadings (0.60HZ/1.75/0.5ZnLZ70) decreased the lifetime of the catalyst to 120h compared to 0.25HZ/1.75LZ70 with an increase in aromatics selectivity from 27.5% to 31.4% and a BTX selectivity of 25% (Table 51, entry 4). The decreased catalytic stability after introducing Zn species was attributed to the coke coverage on acid sites but not the final blockage of pore channels and the optimum amount of Zn was found to be 0.5%.^1248^ They also investigated the use of four layers of nanosized ZSM-5 with gradient increase in the acidity. However, aromatics selectivity did not exceed 32.6% using the sequence beds, and the BTX selectivity was not more than 17.6% With a four-bed reactor the conversion of methanol was 100% over 150 h and selectivity to BTX was 17% (Table 51, entry 5).^1249^

**Table 51:** Methanol to Aromatics: Catalytic Results at Different Catalytic Reaction Conditions

								Selectivity (%)			
Entry	Catalyst (Si/Al ratio)	WHSV (h^–1^)^a^	Reactor	Reaction time (h)	*T* ( °C)	Analytical method	BTX selectivity (C%)	Benzene	Toluene	Xylene	Ref
1	1HS/Z5 (220)–1Zn/Z5 (30)	2	Fixed-bed	12	430	GC	71.7^c^	2.8^c^	30.1^c^	38.8^c^	(1246)
2	8Zn220/2ZnZ30	2	Fixed-bed	39	420	GC, GC/MS	64.0^c^	–	–	–	(1247)
3	0.25HZ–1.75LZ70	2	Fixed-bed	20	430	GC	23^c^				(1248)
4	0.60HZ-1.75/0. 5ZnLZ70	2	Fixed-bed	20	430	GC	25^c^				(1248)
5	Z480/Z240/Z120/Zn60	0.135^b^	Fixed-bed		430	GC	17%				(1249)
6	SZ-170 (122)–Zn/NZ (160)	1	Fixed-bed	12 h (260)	450	GC	45.8 (>20%)	4.3	22.7	18.8	(1250)
7	3% Zn-ZSM-5 (38)	0.35	3-stage Fluidized bed	10	470–550–470	GC	62^e^	9.8^f^	24^f^	22.2^f^	(1251)
8	3% Zn-ZSM-5 (38)	0.5	2-stage Fluidized bed	11	470	GC	62^e^	3–4^f^	15–16^f^	35–39^f^	(1252)
9	2% NiO-Z (45)	1^g^	Fixed-bed	1	510	GC	36.6	3.8	12.5	20.3	(955)
10	2% NiO-Z (45)	1^h^	Fixed-bed	1	510	GC	24.6	2.0	7.6	14.4	(955)
11	2% Zn-ZSM-5 (40)	6	Fixed-bed	1.5	450	GC, GC/MS	70^d^	10	<5		(1256)

a WHSV (weight hour space velocity).

b Flow rate= 25 cm^3^/min.

c BTX selectivity in aromatics.

d Selectivity of aromatics.

e Aromatics yield.

f Benzene (B), toluene (T), xylene (X) yields.

g Additional CO_2_.

h N_2_ is added to the feed.

Wang et al. investigated methanol aromatization over a tandem catalyst. SZ-170-Zn/NZ had the highest catalytic stability, and the methanol conversion was 100% during the first stage of the reaction then it started to decline; however, the rate was still higher than 80% after 260 h and BTX selectivity (45.8% 12 h) decreased with TOS but was still above 20% after 260 h (Table 51, entry 6).^1250^ Chen et al. studied the aromatization of methanol on the Zn impregnated ZSM-5 using a 3 staged fluidized bed reactor. The first stage served for the aromatization of methanol at 470 °C (near the bottom), the second stage served the high-temperature (550 °C) aromatization and dehydrogenation of light hydrocarbon (middle of the bed) and the last stage for the aromatization of olefins near the exit at 470 °C. The set-up showed an increase in the aromatic yield reaching 62% after the third stage and the yield of both benzene and toluene increased to reach 9.8 and 24%, while the xylene decreased to 22.2% (Table 51, entry 7).^1251^ Also, the type and the amount of coke formed on the catalyst differs according to the temperature zone. This ensures that the third stage has the lowest amount of coke, which in turn affects the stability and selectivity toward aromatics.^1251^ The same group investigated a two-stage fluidized bed with decentralized methanol feed; i.e., each bed had its own methanol feed. Generally, methanol was completely converted. The yield of the aromatics decreased with time on stream due to coke deposition. The effect of using decentralized feed was not significant for the yield of benzene and toluene, as they remain between 3–4% and 15–16%, respectively (Table 51, entry 8) and the total amount of aromatics remained 62% as found by three-stage steps. The only enhancement for this method was with regards to the xylene yields which was improved to 35–39% using a methanol flow of 0.94 g/min on both stages and/or 1.24 first and 0.64 second stage. The *p*-xylene in xylenes reached to 29%.^1252^

Li et al. investigated the aromatization of methanol over parent H-ZSM-5 and a modified NiO-H-ZSM-5 in a fixed-bed reactor under CO_2_ and N_2_ flow. Total aromatic yield and BTX selectivity increased with NiO loadings below 2.0 wt% (36.6%), especially in a CO_2_ atmosphere (Table 51, entry 9) in comparison with a N_2_ atmosphere (24.0%) (Table 51, entry 10). Increasing the NiO loading lowered the acidity and therefore the aromatic yield. This is attributed to the cooperation of acid sites with the activated CO_2_ (over NiO species), which not only can effectively promote dehydrogenation of alkanes to form olefin intermediates, but also can accelerate dehydrogenation in the conversion of olefin intermediates to aromatics.^1253^

Alkylation of benzene with methanol over ZSM-5 and Zn-ZSM-5 was independently investigated by Hu et al. and Meng et al., who performed the methanol reaction with benzene to increase the yield of xylenes. Hu et al. used different gas atmospheres N_2_ and/or H_2_ and ZnO content. With increase of the ZnO content from 0 to 1 wt%, the conversion of benzene increased from 51.6 to 53.6% in H_2_ and to 51.8% in N_2_. The modified catalyst could suppress the formation of ethylbenzene under both atmospheres. In general, the increase of benzene conversion meant the alkylation of aromatics with methanol was promoted and the side reaction of methanol to olefins was suppressed.^1254^

Meng et al. synthesized H-ZSM-5, hierarchical IM zeolite (H-IM-5), and desilication by NaOH of IM zeolite H-IM-5(DS) zeolites as highly active and shape selective catalysts for the alkylation of benzene with methanol. The optimum reaction conditions was reaction temperature: 450 °C, reaction pressure: atmospheric pressure, WHSV = 2 h^–1^, the benzene conversion was 78% and the xylene selectivity was 44%.^1255^

Ni et al. reported that HCHO, which is formed by methanol dehydrogenation over Zn/H-ZSM-5 prepared by Zn impregnation, can participate in the synthesis of aromatics. The selectivity of aromatics reaches above 70% in the initial 1.5 h (Table 51, entry 11). After that, the formation of aromatics declines rapidly, whereas CO, CO_2_ and H_2_ all increased quickly. According to the previous studies, the latter three products could be formed by methanol decomposition (CH_3_OH → CO + 2H_2_) and water gas shift reaction (CO + H_2_O → CO_2_ + H_2_), respectively. It is worth noting that HCHO, the product of methanol dehydrogenation (CH_3_OH → HCHO + H_2_), started to appear after 10.5 h TOS, and then continues to grow, even reaching 30.7% after 18.5 h TOS. This suggests that methanol is dehydrogenated forming HCHO which coupled with methanol to form aromatics.^1256^

**Figure 49:**
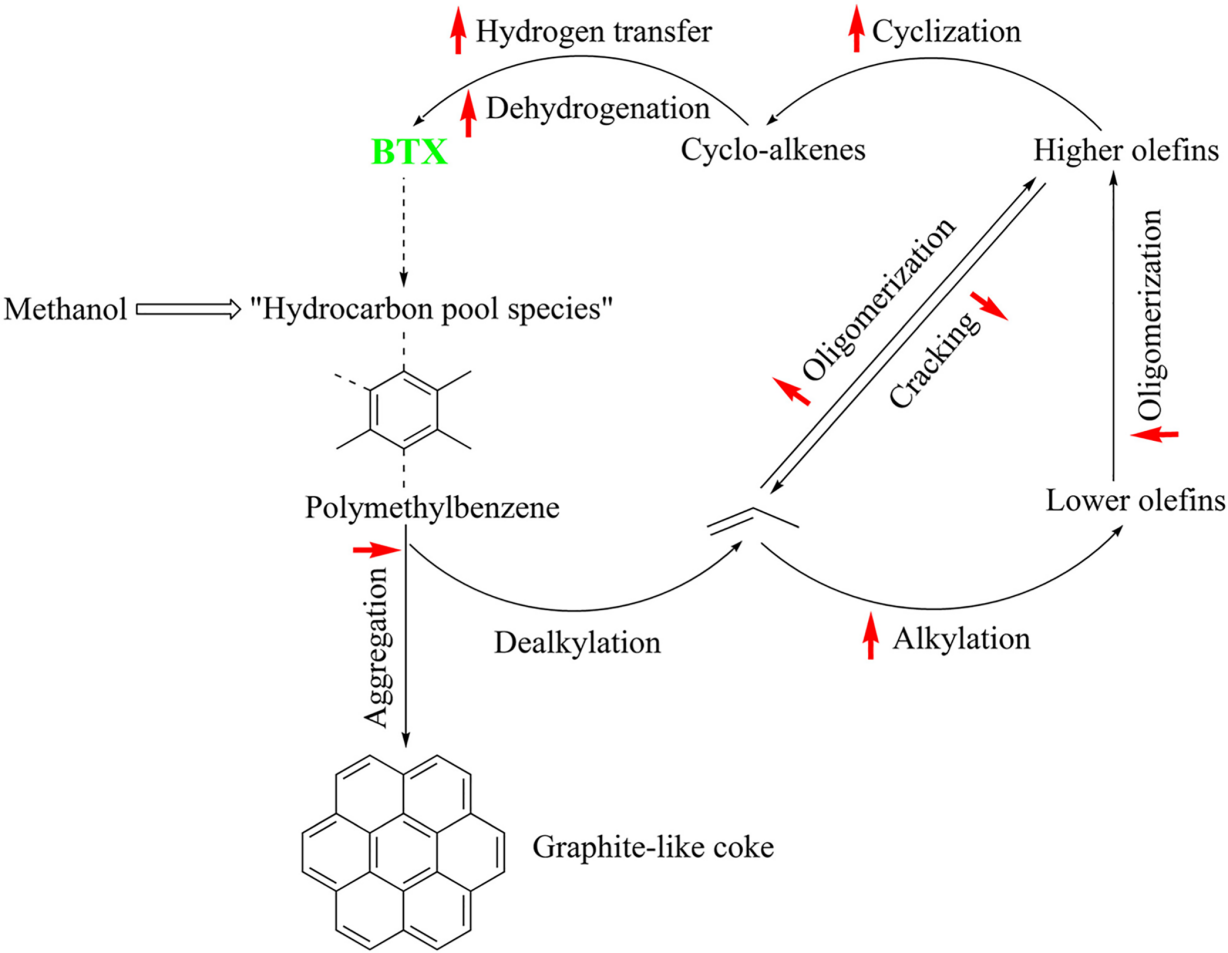
Methanol pathway conversion on H-ZSM-5 catalysts. Reproduced with permission from ref (1239). Copyright 2020 John Wiley & Sons, Ltd.

The catalyst deactivation is related to coking or products adsorption. Smaller crystal size, diffusion, and acidity are the main reasons causing the deactivation due to coke. Strongly acidic sites of the zeolites are the active sites for the production of aromatics; however, the increase of the strongly acidic sites is also favorable to the coke formation, which leads to the rapid deactivation of the catalyst.^1257^ Another factor is the concentration of the reactants. Li et al. studied, using modified ZSM-5 with 1%ZnO-2%P-ZSM-5 (30), the effect of different reaction parameters such as temperature and methanol partial pressure on the rate of deactivation.^1258^ The lifetime of the catalyst decreases with increasing temperature due to coke that is formed from the dehydrogenation and oligomerization of light hydrocarbon which became favorable at higher temperatures. As reported in the literature, the cracking, oligomerization, and hydride transfer reactions mainly occur on Brønsted acid sites. However, the alkylation and dehydrogenation reactions largely occur on the Lewis acid sites (Figure 49). Hence, the synergistic effect between Brønsted and Lewis acid is responsible for improving the selectivity to BTX in the methanol to aromatics conversion and the stability of the catalyst.^1229,1239,1257^ Zhang et al. investigated the regeneration of H-ZSM-5 and/or modified Zn catalyst and found that high air flow rates facilitated the recovery of the catalyst as it provides sufficient air for the combustion of coke, which enhanced the recovery of the deactivated catalyst.^1257^

### 9.3 Reaction Network

**Figure 50:**
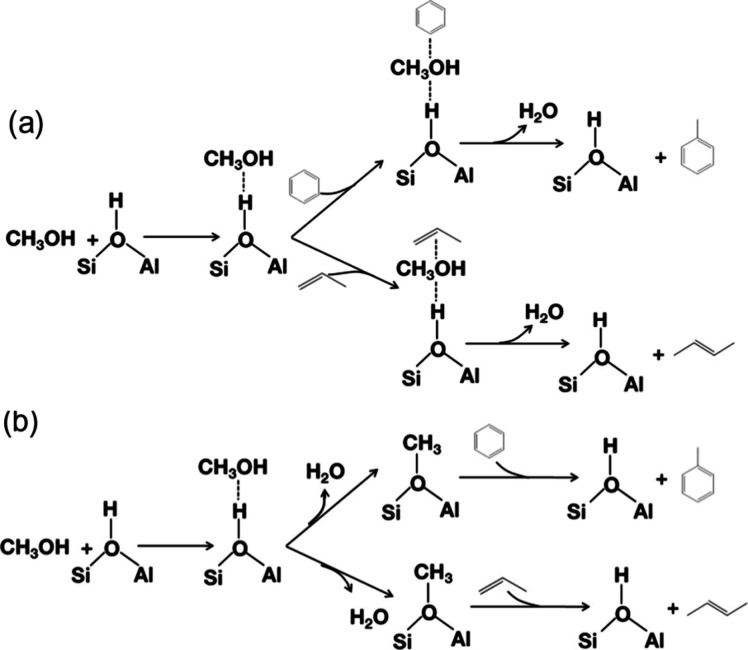
Generally accepted mechanisms for the methylation of aromatics or olefins, in which a gas-phase olefin or aromatic molecule reacts with a meth-oxonium ion (a) or methyl carbenium ion (b). Reproduced with permission from ref (1262). Copyright 2014 Elsevier Inc.

**Figure 51:**
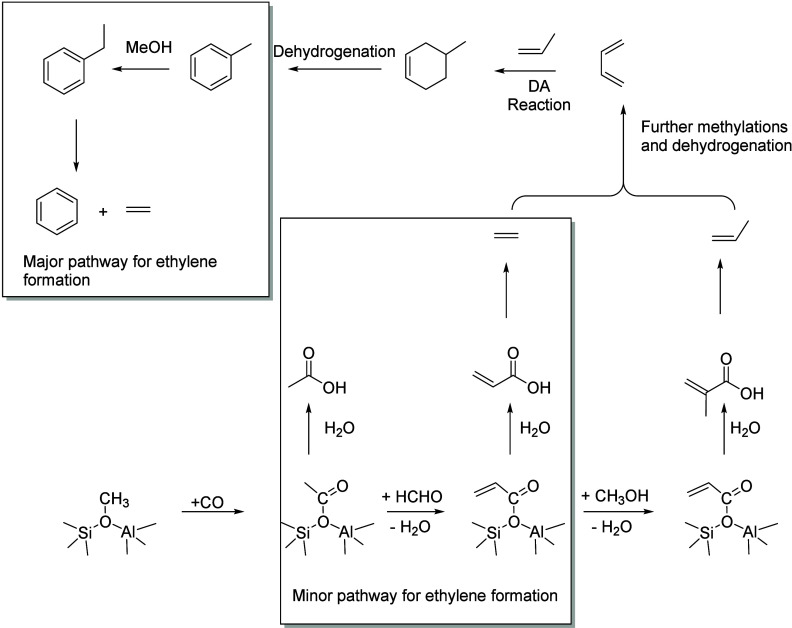
Proposed reaction mechanism for the formation of aromatics from methanol over H-ZSM-5 based on the findings of Haw, Nicholas, Bjørgen, and Lercher.

There has been a long debate on how methanol converts to ethylene as a first step. It is now clear from the work of Haw, Nicholas, and co-workers as well as from Bjørgen and co-workers that the major route is via alkylation of methylated benzenes, forming ethylbenzenes which may undergo a retro Friedel–Crafts alkylation to form ethylene.^1259,1260^ However, this begs the question on how the first aromatics are formed from methanol. For this, Lercher proposed a (minor) pathway by which methanol forms CO by dehydrogenation, which reacts with methanol to acetic acid, which may react with formaldehyde, formed from methanol dehydrogenation to form acrylic acid which decarboxylates to form ethylene.^1261^ Next, ethylene and other alkenes are methylated, either via Prins reaction with formaldehyde or via methylation with methanol. This can go through different pathways via autocatalysis where the hydrocarbon interacts with a chemisorbed methanol and a methylated hydrocarbon is formed and desorbed. The formed higher hydrocarbon molecules may react with other chemisorbed methanol molecules until the higher homologue has reached the size for which the rates of cracking on the acid site are higher than the rates of methylation (Figure 50a).^1262^ The other pathway of oligomerization is based on the hydrocarbon pool. It was discussed that the activated CH_2_ reacts with the hydrocarbon forming homologues as products (Figure 50b). Next, cyclization of olefins and dehydrogenation of the generated cyclic hydrocarbons produces aromatics. The dehydrogenation can occur via hydride transfer reaction and/or in presence of Zn or Ga through direct dehydrogenation (see Figure 51).

### 9.4 Pilot Plant for Methanol to Aromatics

Although methanol to gasoline (MTG) has been commercialized, this is not (yet) the case for methanol to aromatics (MTA). Nevertheless, pilot plants are in operation in China, based on a process developed by the group of Wei from Tsinghua University.^1263^ This process is based on the use of ZSM-5 catalysts modified with silver. Similar to MTG the technology uses fluidized bed reactors which has a number of advantages. They allow better control of the reaction temperature and hence coking is reduced. In addition, the separation of the catalyst for its regeneration is easier than in the case of a packed bed reactor. One reason for the relatively low selectivity to aromatics is the formation of lower hydrocarbons, in particular propane. This was tackled by this group by separating the light hydrocarbons from the aromatics and feeding them into an additional reactor for the conversion of the light hydrocarbons to aromatics (LHTA) at 520–550 °C. The pristine catalyst is first used in the LHTA reactor and after deactivation it is shifted to the MTA reactor, run at 475 °C, for which this catalyst has still sufficient reactivity. The spent catalyst is then regenerated oxidatively at 600–630 °C in a third reactor. An industrial scale reactor with a capacity of 30 kT/a was built based on this technology and has been running uninterrupted for up to 443 h. Methanol conversion is close to 100%. The catalyst consumption is 0.22 kg/ton of methanol. The yield of aromatics is 74.5%.

In conclusion, it is clear that the MTA technology is nearing the stage where it will be implemented. If the technology will be utilized for the conversion of biomethanol will depend on the availability and the price of the biomethanol.

## 10 Aromatics from Chitin

**Scheme 166:**
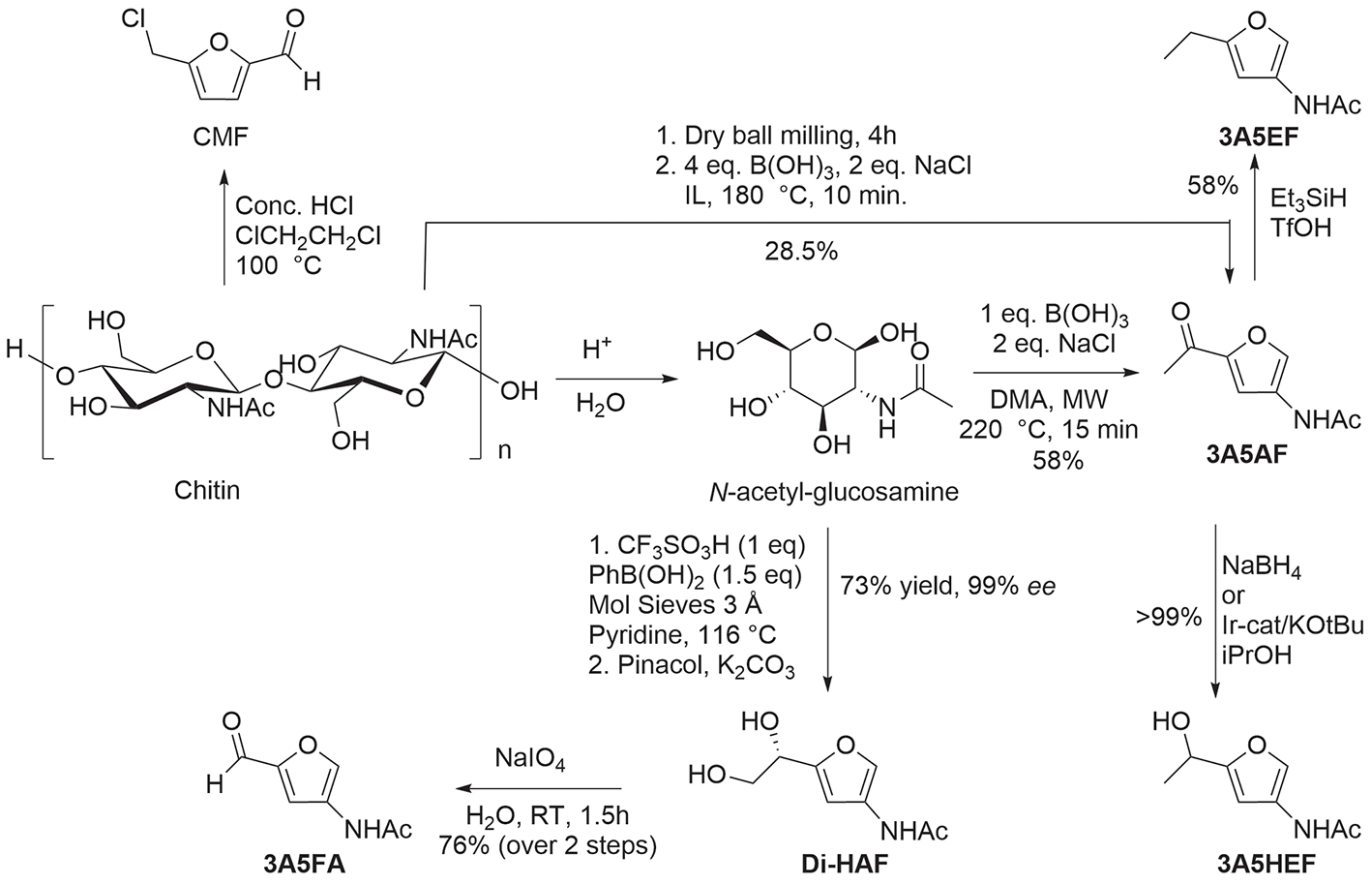
Furans from Chitin

Chitin, a polymer of *N-*acetylglucosamine is the second most abundant polysaccharide in nature after cellulose (Scheme 166). It is estimated that roughly one billion tons of chitin are produced on a yearly basis in nature, mostly by crustaceans, but also by fungi and insects. A large amount of chitin is available as waste from the food industry in the form of shells from shrimps and crabs. Mascal and co-workers were able to convert chitin into 5-chloromethyl-furfural (CMF) in 42% yield using a biphasic system of concentrated HCl and ClCH_2_CH_2_Cl (for the conversion of CMF into aromatics see Scheme 129 in section 2.5.4).^1264^*N*-acetyl-glucosamine is produced by acid hydrolysis of chitin.^1265^ Kerton and co-workers investigated the conversion of *N*-acetylglucosamine to 3-acetamido-5-acetylfuran(**3A5AF**). Using boric acid (1 equiv) in the presence of 2 equiv of NaCl in NMP as solvent the carbohydrate was converted into **3A5AF** within 15 min in 58% yield. This was later improved by Chen and co-workers, who performed the dehydration of *N*-acetylglucosamine in γ-valerolactone using HCl and NH_4_SCN as catalyst at 140 °C during 2h and obtained **3A5AF** in 75% HPLC yield.^1266^ It is also possible to produce **3A5AF** from chitin directly using a procedure similar as used by Kerton, but then the yield is only 7.5%.^1267^ One problem in this reaction is the poor solubility of chitin, which is related to its high degree of crystallinity; this makes it resistant against hydrolysis. Thus, Yan and co-workers examined a number of methods to reduce crystallinity and particle size.^1268^ Well-known methods such as wet or dry ball milling, steam explosion, acid or alkaline impregnation, and treatment with an ionic liquid were all examined. The pretreated samples were next subjected to the dehydration reaction at 180 °C using boric acid (400 mol%) and HCl (100 mol%), either in NMP or in an ionic liquid. In all cases, the yields obtained with ionic liquid were highest. Best results (28.5%) were obtained with dry ball milling followed by dehydration in the ionic liquid for 10 minutes. The **3A5AF** was isolated by chromatography in 20% yield.

**Scheme 167:**
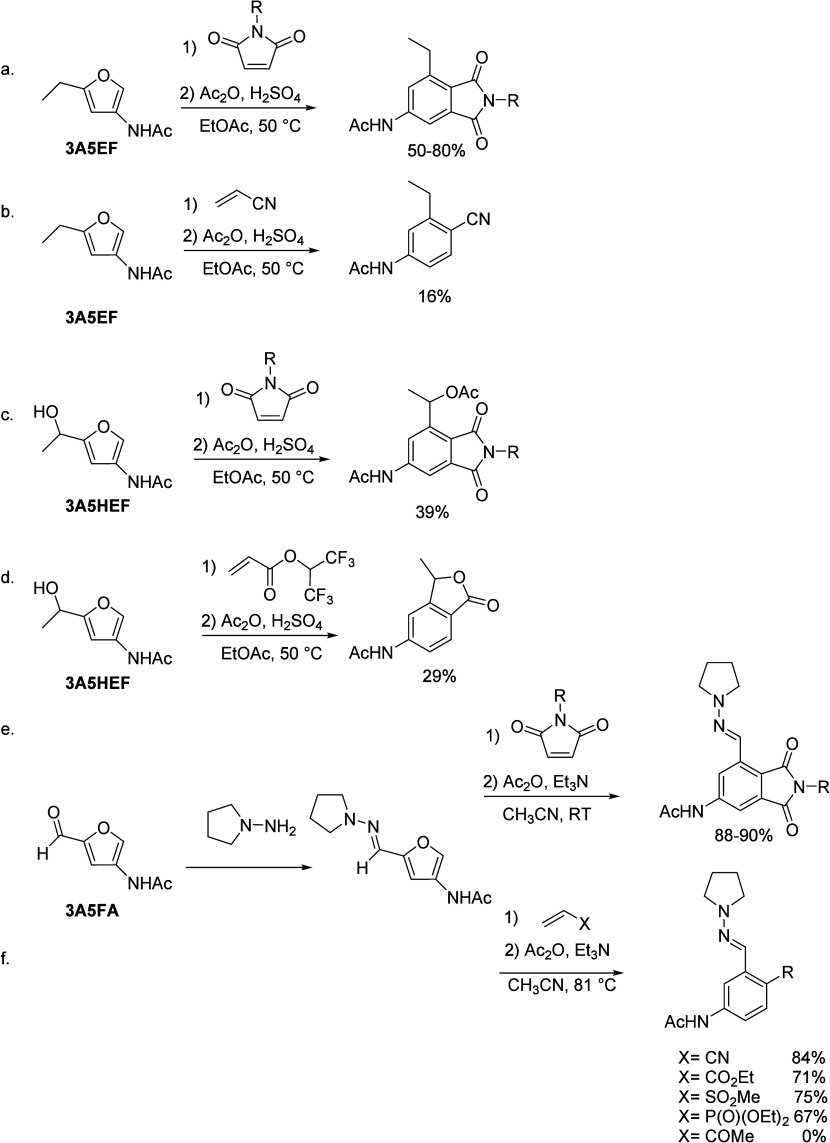
DA Reactions on 3-Acetamidofurans to Obtain Acylated Anilines

The ketone of **3A5AF** can be reduced to the alcohol, 3-acetamido-5-(1-hydroxy)ethylfuran (**3A5HEF**) in virtually quantitative yield either with NaBH_4_ or catalytically using 1 mol% of IrH_2_Cl[(*i*Pr_2_PC_2_H_4_)2NH in a transfer hydrogenation with isopropanol as reductant.^1269^ Sperry and co-workers reduced the ketone to 3-acetamido-5-ethylfuran (**3A5EF**) using Et_3_SiH/TfOH in 58% yield.^1270^ Minnaard and co-workers were able to find conditions were dehydration of the side chain diol to the acetyl group is prevented by buffering off a mixture of the acid catalysts CF_3_SO_3_H and PhB(OH)_2_ with pyridine. In this way he was able to isolate 5-(1,2-dihydroxyethyl)-3-acetamidofuran (**Di-HAF**) in 73% yield with full retention of the chirality in the chiral alcohol group.^1271^ Afonso and co-workers were able to convert **Di-HAF** with NaIO_4_ to the furfuraldehyde **3A5FA** in 76% yield (over the 2 steps).^1272^ The 3-acetamido-furans that were obtained in this way (Scheme 166) have been used in Diels–Alder reactions with activated alkenes to obtain acetylated anilines (Scheme 167).

Gomes and co-workers reported the DA rection of **3A5AF** and **3A5HEF** with a range of *N*-substituted maleimides to the oxanorbornene compounds, but failed to induce aromatization via dehydration under acidic or basic conditions.^1273^ Pastre and co-workers subjected **3A5EF** to the DA reaction with N-substituted maleimides and managed to induce dehydration to the acylated anilines in decent yields (50–80%) using a mixture of Ac_2_O/H_2_SO_4_ (Scheme 167a). Reaction with acrylonitrile gave the aromatic product in only 16% yield Scheme 167b). Reaction of **3A5HEF** with maleimide followed by dehydration led to the acylated aniline in which the alcohol group was acetylated (Scheme 167c). Reaction of **3A5HEF** with hexafluoroisopropyl acrylate led to formation of the lactone in 29% yield (Scheme 167d).

Minnaard and co-workers examined the possibility of performing DA reactions on **3A5FA**.^1274^ Fearing that the formyl group would deactivate the furan ring for DA reactions, they converted it into a hydrazone. Reaction with dimethylhydrazine worked in mediocre yield and the hydrazone underwent the DA reaction with *N*-methyl-maleimide also in mediocre yield. Much better results were obtained by using the more nucleophilic pyrrolidine hydrazine. With this compound the hydrazone of **3A5FA** was obtained in 82% isolated yield and this compound underwent the DA reaction smoothly, not only with substituted maleimides (Scheme 167e), but also with fumaronitrile, diethyl maleate, acrylate esters, and acrylonitrile (Scheme 167f). The same procedure could also be applied to **3A5AF**. The hydrazones could be deprotected to the aldehydes by reaction with ozone. Alternatively, oxidation with *m*-chloroperbenzoic acid converted the hydrazone into a nitrile.

In conclusion, even though chitin is a neglected renewable resource, the currently known routes from chitin to aromatics suffer from overall low selectivities, which makes the implementation of such routes unlikely.

## 11 Conclusions

**Scheme 168:**
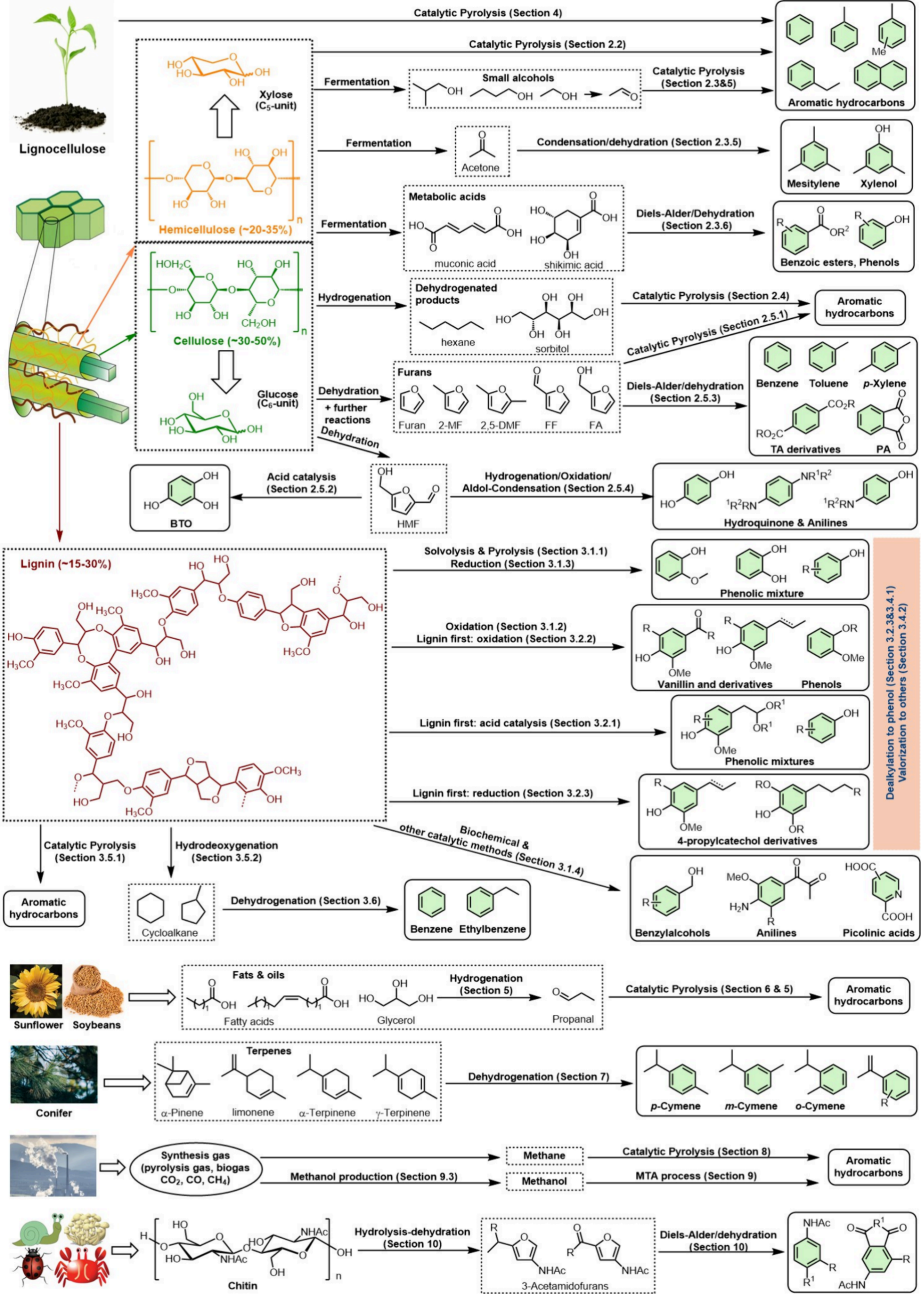
Summary of Methods Reported for the Conversion of Renewable Resources into Benzenoid Aromatics

The many methods that have been reported for the conversion of renewable resources into benzenoid aromatics are summarized in Scheme 168.

There are, roughly speaking, two different ways to convert renewable resources into benzenoid aromatics. One method that has been worked on by a vast army of researchers, is catalytic fast pyrolysis, which can be used on practically all carbon sources, perhaps with the exception of CO_2_. It seems that use of the zeolite H-ZSM-5 modified with zinc, silver, or gallium leads to the best results. Temperatures depend very much on the raw material used and are between 300 and 700 °C. These reactions lead to the formation of carbon black, gases, and an oil (bio-oil) which contains hydrocarbons, among which are benzenoid aromatics. Unfortunately, it is not easy to get a clear insight into the value of the published methodologies. In almost all publications, no information is given on how many grams of the oil is obtained from how many grams of renewable resource. The obtained oil is usually analyzed with GC. In the majority of cases, no internal standard was used and no dose–response curves were made for the compounds of interest, such as BTX. Products are almost never isolated as an independent means of establishing the yields. It remains unclear, in most cases, if the percentages of BTX given are the percentages found in the bio-oil or if they are real carbon yields based on biorenewable used. A recurring theme is the poor lifetime of the catalyst, which is real, but on the other hand (methane-based), methanol to gasoline (MTG) and methanol to olefins (MTO) as well as C_4_–C_5_ alkenes to aromatics are commercial processes, and thus these problems seem surmountable. Indeed, a 30 kt/year pilot plant is already in operation for conversion of methanol to aromatics in China. The two pilot plants for conversion of lignocellulose to aromatics have not led to commercial plants. A major problem is, of course, the weight loss that occurs on converting highly oxygenated biomass to hydrocarbons. It seems that both pilot plants are now focusing on converting waste plastics or a mixture of biomass and waste plastics to aromatics, and this may well be the future for this technology.

The low-temperature methods, which often are based on the use of so-called platform chemicals, seem at first less advantageous, as these usually need several steps to arrive at the desired aromatic product. Nevertheless, they have the great advantage that the selectivity of the individual steps and even of the overall process is much higher than that of the high-temperature methods. Indeed, Origin Materials’ plant that converts lignocellulose into xylene in three separate steps has already started operations last year. This is the conventional wisdom that today’s captains of industry, who focus solely on (short-term) shareholder value and demand a very fast break-even point for their new investments, have forgotten: in the end, the cost of a bulk chemical is determined largely by the cost of the raw material and the selectivities of the conversions, as most plants are written off after 10 years but keep operating for 50 years or more.

Nevertheless, as the price of fossil resources increases, more and more of these methods will become feasible.
